# Supplementary material for: The frequent complete subgraphs in the human connectome
Source: PLoS One. 2020 Aug 20;15(8):e0236883. doi: 10.1371/journal.pone.0236883 (PMC7444532; doi:10.1371/journal.pone.0236883)
Supplement: S1 Table — (PDF) [file pone.0236883.s001.pdf]

	frequency	name
2	1	(Left-Caudate)(Left-Pallidum)(Left-Putamen)(Left-Thalamus-Proper)
3	1	(Left-Caudate)(Left-Pallidum)(Left-Putamen)
4	1	(Left-Caudate)(Left-Pallidum)(Left-Thalamus-Proper)
5	1	(Left-Caudate)(Left-Pallidum)
6	1	(Left-Caudate)(Left-Putamen)(Left-Thalamus-Proper)
7	1	(Left-Caudate)(Left-Putamen)
8	1	(Left-Caudate)(Left-Thalamus-Proper)
9	1	(Left-Caudate)(lh.caudalanteriorcingulate_2)
10	1	(Left-Caudate)(lh.caudalmiddlefrontal_6)
11	1	(Left-Caudate)(lh.posteriorcingulate_1)
12	1	(Left-Caudate)(lh.posteriorcingulate_3)
13	1	(Left-Hippocampus)(Left-Putamen)(Left-Thalamus-Proper)
14	1	(Left-Hippocampus)(Left-Putamen)
15	1	(Left-Hippocampus)(Left-Thalamus-Proper)
16	1	(Left-Hippocampus)(lh.isthmuscingulate_3)
17	1	(Left-Pallidum)(Left-Putamen)(Left-Thalamus-Proper)
18	1	(Left-Pallidum)(Left-Putamen)
19	1	(Left-Pallidum)(Left-Thalamus-Proper)
20	1	(Left-Putamen)(Left-Thalamus-Proper)(lh.insula_1)
21	1	(Left-Putamen)(Left-Thalamus-Proper)
22	1	(Left-Putamen)(lh.insula_1)
23	1	(Left-Putamen)(lh.insula_2)
24	1	(Left-Putamen)(lh.insula_3)
25	1	(Left-Putamen)(lh.insula_6)
26	1	(Left-Putamen)(lh.precentral_11)
27	1	(Left-Thalamus-Proper)(lh.insula_1)
28	1	(Left-Thalamus-Proper)(Right-Thalamus-Proper)
29	1	(lh.caudalmiddlefrontal_5)(lh.caudalmiddlefrontal_6)
30	1	(lh.fusiform_1)(lh.fusiform_5)
31	1	(lh.insula_1)(lh.supramarginal_1)
32	1	(lh.insula_6)(lh.parsopercularis_3)
33	1	(lh.parsopercularis_1)(lh.parsopercularis_3)
34	1	(lh.parsopercularis_1)(lh.parstriangularis_3)
35	1	(lh.parstriangularis_3)(lh.rostralmiddlefrontal_1)
36	1	(lh.postcentral_14)(lh.precentral_16)
37	1	(lh.precentral_11)(lh.precentral_9)
38	1	(lh.superiorfrontal_10)(lh.superiorfrontal_8)
39	1	(lh.superiorfrontal_11)(lh.superiorfrontal_8)
40	1	(lh.superiorfrontal_14)(lh.superiorfrontal_15)
41	1	(lh.superiorfrontal_14)(lh.superiorfrontal_8)
42	1	(lh.superiorfrontal_15)(lh.superiorfrontal_18)
43	1	(lh.superiorfrontal_4)(lh.superiorfrontal_7)
44	1	(lh.superiorfrontal_4)(lh.superiorfrontal_8)
45	1	(rh.caudalmiddlefrontal_4)(rh.caudalmiddlefrontal_5)
46	1	(rh.fusiform_1)(rh.lateraloccipital_10)
47	1	(rh.fusiform_1)(rh.lateraloccipital_8)
48	1	(rh.fusiform_3)(rh.lateraloccipital_10)
49	1	(rh.insula_1)(rh.supramarginal_9)
50	1	(rh.paracentral_5)(rh.precentral_15)

51	1	(rh.parsopercularis_2)(rh.parsopercularis_4)
52	1	(rh.precentral_10)(rh.precentral_7)
53	1	(rh.precentral_13)(rh.precentral_7)
54	1	(rh.superiorfrontal_11)(rh.superiorfrontal_8)
55	1	(rh.superiorfrontal_16)(rh.superiorfrontal_17)
56	1	(rh.superiorfrontal_5)(rh.superiorfrontal_8)
57	1	(rh.superiorfrontal_7)(rh.superiorfrontal_8)(rh.superiorfrontal_9)
58	1	(rh.superiorfrontal_7)(rh.superiorfrontal_8)
59	1	(rh.superiorfrontal_7)(rh.superiorfrontal_9)
60	1	(rh.superiorfrontal_8)(rh.superiorfrontal_9)
61	1	(rh.superiorparietal_1)(rh.superiorparietal_7)
62	1	(rh.supramarginal_8)(rh.supramarginal_9)
63	1	(Right-Caudate)(rh.caudalanteriorcingulate_2)
64	1	(Right-Caudate)(rh.caudalanteriorcingulate_3)
65	1	(Right-Caudate)(rh.posteriorcingulate_1)
66	1	(Right-Caudate)(rh.posteriorcingulate_2)
67	1	(Right-Caudate)(rh.posteriorcingulate_3)
68	1	(Right-Caudate)(rh.precentral_13)
69	1	(Right-Caudate)(Right-Pallidum)(Right-Putamen)(Right-Thalamus-Proper)
70	1	(Right-Caudate)(Right-Pallidum)(Right-Putamen)
71	1	(Right-Caudate)(Right-Pallidum)(Right-Thalamus-Proper)
72	1	(Right-Caudate)(Right-Pallidum)
73	1	(Right-Caudate)(Right-Putamen)(Right-Thalamus-Proper)
74	1	(Right-Caudate)(Right-Putamen)
75	1	(Right-Caudate)(Right-Thalamus-Proper)
76	1	(Right-Hippocampus)(rh.isthmuscingulate_2)
77	1	(Right-Hippocampus)(Right-Putamen)(Right-Thalamus-Proper)
78	1	(Right-Hippocampus)(Right-Putamen)
79	1	(Right-Hippocampus)(Right-Thalamus-Proper)
80	1	(Right-Pallidum)(Right-Putamen)(Right-Thalamus-Proper)
81	1	(Right-Pallidum)(Right-Putamen)
82	1	(Right-Pallidum)(Right-Thalamus-Proper)
83	1	(Right-Putamen)(rh.caudalmiddlefrontal_4)
84	1	(Right-Putamen)(rh.insula_1)
85	1	(Right-Putamen)(rh.insula_2)
86	1	(Right-Putamen)(rh.insula_3)
87	1	(Right-Putamen)(rh.insula_4)
88	1	(Right-Putamen)(rh.insula_6)
89	1	(Right-Putamen)(rh.insula_7)
90	1	(Right-Putamen)(rh.precentral_7)
91	1	(Right-Putamen)(Right-Thalamus-Proper)(rh.insula_2)
92	1	(Right-Putamen)(Right-Thalamus-Proper)
93	1	(Right-Thalamus-Proper)(rh.insula_2)
94	0.99758	(Left-Caudate)(lh.caudalanteriorcingulate_1)(lh.caudalanteriorcingulate_2)
95	0.99758	(Left-Caudate)(lh.caudalanteriorcingulate_1)
96	0.99758	(Left-Caudate)(Right-Caudate)
97	0.99758	(Left-Hippocampus)(lh.superiortemporal_2)
98	0.99758	(Left-Putamen)(lh.caudalmiddlefrontal_3)
99	0.99758	(Left-Putamen)(lh.caudalmiddlefrontal_4)
100	0.99758	(lh.caudalanteriorcingulate_1)(lh.caudalanteriorcingulate_2)

101	0.99758	(lh.caudalmiddlefrontal_4)(lh.caudalmiddlefrontal_6)
102	0.99758	(lh.inferiorparietal_8)(lh.inferiorparietal_9)
103	0.99758	(lh.lingual_6)(lh.lingual_8)
104	0.99758	(lh.paracentral_4)(lh.paracentral_5)
105	0.99758	(lh.paracentral_4)(lh.precentral_2)
106	0.99758	(lh.paracentral_4)(lh.precentral_3)
107	0.99758	(lh.paracentral_5)(lh.precentral_2)
108	0.99758	(lh.rostralmiddlefrontal_1)(lh.rostralmiddlefrontal_4)
109	0.99758	(lh.superiorfrontal_10)(lh.superiorfrontal_4)(lh.superiorfrontal_8)
110	0.99758	(lh.superiorfrontal_10)(lh.superiorfrontal_4)
111	0.99758	(lh.superiorfrontal_10)(lh.superiorfrontal_7)
112	0.99758	(lh.superiorfrontal_11)(lh.superiorfrontal_4)(lh.superiorfrontal_8)
113	0.99758	(lh.superiorfrontal_11)(lh.superiorfrontal_4)
114	0.99758	(lh.superiorfrontal_12)(lh.superiorfrontal_14)
115	0.99758	(lh.superiorfrontal_13)(lh.superiorfrontal_14)
116	0.99758	(lh.superiorfrontal_14)(lh.superiorfrontal_17)
117	0.99758	(lh.superiorfrontal_15)(lh.superiorfrontal_16)
118	0.99758	(lh.superiorfrontal_4)(lh.superiorfrontal_5)
119	0.99758	(lh.superiorfrontal_4)(lh.superiorfrontal_7)(lh.superiorfrontal_8)
120	0.99758	(lh.superiorfrontal_7)(lh.superiorfrontal_8)
121	0.99758	(lh.superiorfrontal_7)(lh.superiorfrontal_9)
122	0.99758	(rh.caudalmiddlefrontal_4)(rh.insula_3)
123	0.99758	(rh.inferiorparietal_1)(rh.inferiorparietal_4)
124	0.99758	(rh.lateraloccipital_7)(rh.lingual_2)
125	0.99758	(rh.lingual_5)(rh.lingual_7)
126	0.99758	(rh.precentral_11)(rh.precentral_13)
127	0.99758	(rh.precuneus_1)(rh.superiorparietal_11)
128	0.99758	(rh.superiorfrontal_11)(rh.superiorfrontal_13)
129	0.99758	(rh.superiorfrontal_11)(rh.superiorfrontal_8)(rh.superiorfrontal_9)
130	0.99758	(rh.superiorfrontal_11)(rh.superiorfrontal_9)
131	0.99758	(rh.superiorfrontal_5)(rh.superiorfrontal_6)
132	0.99758	(rh.superiorfrontal_5)(rh.superiorfrontal_7)(rh.superiorfrontal_8)
133	0.99758	(rh.superiorfrontal_5)(rh.superiorfrontal_7)
134	0.99758	(Right-Caudate)(rh.caudalmiddlefrontal_4)
135	0.99758	(Right-Caudate)(Right-Putamen)(rh.caudalmiddlefrontal_4)
136	0.99758	(Right-Caudate)(Right-Putamen)(rh.precentral_13)
137	0.99758	(Right-Hippocampus)(rh.bankssts_2)
138	0.99758	(Right-Hippocampus)(rh.insula_2)
139	0.99758	(Right-Hippocampus)(rh.lingual_7)
140	0.99758	(Right-Hippocampus)(Right-Pallidum)(Right-Putamen)(Right-Thalamus-Proper)
141	0.99758	(Right-Hippocampus)(Right-Pallidum)(Right-Putamen)
142	0.99758	(Right-Hippocampus)(Right-Pallidum)(Right-Thalamus-Proper)
143	0.99758	(Right-Hippocampus)(Right-Pallidum)
144	0.99758	(Right-Hippocampus)(Right-Putamen)(rh.insula_2)
145	0.99758	(Right-Hippocampus)(Right-Putamen)(Right-Thalamus-Proper)(rh.insula_2)
146	0.99758	(Right-Hippocampus)(Right-Thalamus-Proper)(rh.insula_2)
147	0.99758	(Right-Putamen)(rh.caudalmiddlefrontal_4)(rh.insula_3)
148	0.99758	(Right-Putamen)(rh.insula_1)(rh.supramarginal_9)
149	0.99758	(Right-Putamen)(rh.parsopercularis_4)
150	0.99758	(Right-Putamen)(rh.precentral_13)(rh.precentral_7)

151	0.99758	(Right-Putamen)(rh.precentral_13)
152	0.99758	(Right-Putamen)(rh.precentral_5)
153	0.99758	(Right-Putamen)(rh.supramarginal_9)
154	0.99517	(Left-Caudate)(Left-Putamen)(lh.caudalmiddlefrontal_3)
155	0.99517	(Left-Caudate)(Left-Putamen)(lh.precentral_11)
156	0.99517	(Left-Caudate)(lh.caudalanteriorcingulate_2)(lh.posteriorcingulate_1)
157	0.99517	(Left-Caudate)(lh.caudalmiddlefrontal_3)
158	0.99517	(Left-Caudate)(lh.posteriorcingulate_2)
159	0.99517	(Left-Caudate)(lh.precentral_11)
160	0.99517	(Left-Caudate)(lh.superiorfrontal_12)
161	0.99517	(Left-Hippocampus)(Left-Putamen)(lh.insula_2)
162	0.99517	(Left-Hippocampus)(lh.insula_2)
163	0.99517	(Left-Putamen)(lh.insula_2)(lh.precentral_11)
164	0.99517	(Left-Putamen)(lh.precentral_11)(lh.precentral_9)
165	0.99517	(Left-Putamen)(lh.precentral_9)
166	0.99517	(lh.caudalanteriorcingulate_2)(lh.posteriorcingulate_1)
167	0.99517	(lh.caudalmiddlefrontal_4)(lh.caudalmiddlefrontal_5)
168	0.99517	(lh.fusiform_1)(lh.fusiform_2)
169	0.99517	(lh.fusiform_2)(lh.lateraloccipital_6)
170	0.99517	(lh.insula_2)(lh.precentral_11)
171	0.99517	(lh.isthmuscingulate_3)(lh.precuneus_11)
172	0.99517	(lh.lateraloccipital_6)(lh.pericalcarine_3)
173	0.99517	(lh.superiorfrontal_10)(lh.superiorfrontal_11)(lh.superiorfrontal_8)
174	0.99517	(lh.superiorfrontal_10)(lh.superiorfrontal_11)
175	0.99517	(lh.superiorfrontal_10)(lh.superiorfrontal_4)(lh.superiorfrontal_7)
176	0.99517	(lh.superiorfrontal_10)(lh.superiorfrontal_7)(lh.superiorfrontal_8)
177	0.99517	(lh.superiorfrontal_3)(lh.superiorfrontal_4)
178	0.99517	(lh.superiorfrontal_4)(lh.superiorfrontal_6)
179	0.99517	(rh.fusiform_1)(rh.lateraloccipital_10)(rh.lateraloccipital_8)
180	0.99517	(rh.fusiform_1)(rh.lingual_2)
181	0.99517	(rh.fusiform_2)(rh.lateraloccipital_10)
182	0.99517	(rh.inferiorparietal_11)(rh.inferiorparietal_12)
183	0.99517	(rh.inferiorparietal_12)(rh.inferiorparietal_8)
184	0.99517	(rh.insula_1)(rh.insula_3)
185	0.99517	(rh.insula_2)(rh.insula_4)
186	0.99517	(rh.lateraloccipital_10)(rh.lateraloccipital_8)
187	0.99517	(rh.lateraloccipital_4)(rh.lateraloccipital_8)
188	0.99517	(rh.parsopercularis_2)(rh.parsopercularis_3)
189	0.99517	(rh.precuneus_4)(rh.precuneus_7)
190	0.99517	(rh.superiorfrontal_10)(rh.superiorfrontal_11)
191	0.99517	(rh.superiorfrontal_6)(rh.superiorfrontal_8)
192	0.99517	(Right-Caudate)(rh.caudalanteriorcingulate_1)
193	0.99517	(Right-Caudate)(rh.parsopercularis_4)
194	0.99517	(Right-Putamen)(rh.insula_1)(rh.insula_3)
195	0.99517	(Right-Putamen)(rh.insula_2)(rh.insula_4)
196	0.99517	(Right-Putamen)(rh.insula_5)
197	0.99275	(Left-Amygdala)(Left-Putamen)(Left-Thalamus-Proper)
198	0.99275	(Left-Amygdala)(Left-Putamen)
199	0.99275	(Left-Amygdala)(Left-Thalamus-Proper)
200	0.99275	(Left-Caudate)(lh.precentral_4)

201	0.99275	(Left-Putamen)(lh.insula_1)(lh.precentral_11)
202	0.99275	(Left-Putamen)(lh.insula_6)(lh.parsopercularis_3)
203	0.99275	(Left-Putamen)(lh.parsopercularis_3)
204	0.99275	(lh.caudalanteriorcingulate_2)(lh.rostralanteriorcingulate_2)
205	0.99275	(lh.caudalmiddlefrontal_4)(lh.caudalmiddlefrontal_5)(lh.caudalmiddlefrontal_6)
206	0.99275	(lh.cuneus_1)(lh.superiorparietal_12)
207	0.99275	(lh.fusiform_2)(lh.lingual_8)
208	0.99275	(lh.insula_1)(lh.precentral_11)
209	0.99275	(lh.isthmuscingulate_2)(lh.isthmuscingulate_3)
210	0.99275	(lh.lateraloccipital_2)(lh.pericalcarine_2)
211	0.99275	(lh.paracentral_1)(lh.paracentral_4)
212	0.99275	(lh.paracentral_4)(lh.paracentral_5)(lh.precentral_2)
213	0.99275	(lh.posteriorcingulate_1)(lh.posteriorcingulate_2)
214	0.99275	(lh.precentral_6)(lh.precentral_7)
215	0.99275	(lh.precuneus_9)(lh.superiorparietal_11)
216	0.99275	(lh.superiorfrontal_10)(lh.superiorfrontal_4)(lh.superiorfrontal_7)(lh.superiorfrontal_8)
217	0.99275	(lh.superiorfrontal_11)(lh.superiorfrontal_12)
218	0.99275	(lh.superiorfrontal_14)(lh.superiorfrontal_9)
219	0.99275	(lh.superiorfrontal_16)(lh.superiorfrontal_18)
220	0.99275	(lh.superiorfrontal_3)(lh.superiorfrontal_4)(lh.superiorfrontal_5)
221	0.99275	(lh.superiorfrontal_3)(lh.superiorfrontal_5)
222	0.99275	(lh.superiorfrontal_6)(lh.superiorfrontal_7)
223	0.99275	(lh.supramarginal_6)(lh.supramarginal_9)
224	0.99275	(rh.fusiform_1)(rh.fusiform_3)(rh.lateraloccipital_10)
225	0.99275	(rh.fusiform_1)(rh.fusiform_3)
226	0.99275	(rh.fusiform_1)(rh.lateraloccipital_7)
227	0.99275	(rh.fusiform_1)(rh.lingual_5)
228	0.99275	(rh.isthmuscingulate_2)(rh.precuneus_3)
229	0.99275	(rh.lateraloccipital_3)(rh.lateraloccipital_4)
230	0.99275	(rh.lateraloccipital_7)(rh.lateraloccipital_8)
231	0.99275	(rh.lingual_7)(rh.precuneus_2)
232	0.99275	(rh.rostralmiddlefrontal_3)(rh.rostralmiddlefrontal_5)
233	0.99275	(rh.superiorfrontal_5)(rh.superiorfrontal_6)(rh.superiorfrontal_8)
234	0.99275	(rh.superiorfrontal_6)(rh.superiorfrontal_7)
235	0.99275	(Right-Caudate)(rh.insula_1)
236	0.99275	(Right-Caudate)(rh.posteriorcingulate_4)
237	0.99275	(Right-Caudate)(Right-Putamen)(rh.insula_1)
238	0.99275	(Right-Caudate)(Right-Putamen)(rh.parsopercularis_4)
239	0.99034	(Left-Accumbens-area)(Left-Pallidum)(Left-Thalamus-Proper)
240	0.99034	(Left-Accumbens-area)(Left-Pallidum)
241	0.99034	(Left-Accumbens-area)(Left-Thalamus-Proper)
242	0.99034	(Left-Amygdala)(Left-Hippocampus)(Left-Putamen)(Left-Thalamus-Proper)
243	0.99034	(Left-Amygdala)(Left-Hippocampus)(Left-Putamen)
244	0.99034	(Left-Amygdala)(Left-Hippocampus)(Left-Thalamus-Proper)
245	0.99034	(Left-Amygdala)(Left-Hippocampus)
246	0.99034	(Left-Amygdala)(Left-Pallidum)(Left-Putamen)(Left-Thalamus-Proper)
247	0.99034	(Left-Amygdala)(Left-Pallidum)(Left-Putamen)
248	0.99034	(Left-Amygdala)(Left-Pallidum)(Left-Thalamus-Proper)
249	0.99034	(Left-Amygdala)(Left-Pallidum)
250	0.99034	(Left-Caudate)(Left-Putamen)(lh.caudalanteriorcingulate_2)

251	0.99034	(Left-Caudate)(lh.rostralanteriorcingulate_1)
252	0.99034	(Left-Caudate)(lh.rostralanteriorcingulate_2)
253	0.99034	(Left-Caudate)(lh.superiorfrontal_11)
254	0.99034	(Left-Hippocampus)(lh.fusiform_5)
255	0.99034	(Left-Putamen)(lh.caudalanteriorcingulate_2)
256	0.99034	(Left-Putamen)(lh.insula_5)
257	0.99034	(Left-Putamen)(lh.insula_7)
258	0.99034	(Left-Putamen)(lh.lateralorbitofrontal_4)
259	0.99034	(Left-Putamen)(lh.postcentral_8)
260	0.99034	(Left-Putamen)(lh.precentral_14)
261	0.99034	(Left-Putamen)(lh.precentral_7)
262	0.99034	(lh.caudalanteriorcingulate_2)(lh.superiorfrontal_11)
263	0.99034	(lh.caudalanteriorcingulate_2)(lh.superiorfrontal_14)
264	0.99034	(lh.caudalanteriorcingulate_2)(lh.superiorfrontal_8)
265	0.99034	(lh.caudalmiddlefrontal_3)(lh.caudalmiddlefrontal_4)
266	0.99034	(lh.caudalmiddlefrontal_4)(lh.precentral_11)
267	0.99034	(lh.fusiform_2)(lh.lateraloccipital_10)
268	0.99034	(lh.fusiform_4)(lh.fusiform_5)
269	0.99034	(lh.lateraloccipital_3)(lh.pericalcarine_2)
270	0.99034	(lh.lingual_1)(lh.pericalcarine_2)
271	0.99034	(lh.parsopercularis_3)(lh.parsopercularis_4)
272	0.99034	(lh.postcentral_14)(lh.supramarginal_2)
273	0.99034	(lh.precentral_11)(lh.precentral_4)
274	0.99034	(lh.precentral_11)(lh.precentral_7)
275	0.99034	(lh.precuneus_10)(lh.precuneus_9)
276	0.99034	(lh.precuneus_6)(lh.superiorparietal_6)
277	0.99034	(lh.precuneus_8)(lh.precuneus_9)
278	0.99034	(lh.rostralmiddlefrontal_8)(lh.superiorfrontal_4)
279	0.99034	(lh.superiorfrontal_10)(lh.superiorfrontal_11)(lh.superiorfrontal_4)(lh.superiorfrontal_8)
280	0.99034	(lh.superiorfrontal_10)(lh.superiorfrontal_11)(lh.superiorfrontal_4)
281	0.99034	(lh.superiorfrontal_12)(lh.superiorfrontal_8)
282	0.99034	(lh.superiorfrontal_13)(lh.superiorfrontal_9)
283	0.99034	(lh.superiorfrontal_15)(lh.superiorfrontal_16)(lh.superiorfrontal_18)
284	0.99034	(lh.superiorparietal_5)(lh.superiorparietal_6)
285	0.99034	(rh.bankssts_2)(rh.inferiorparietal_4)
286	0.99034	(rh.insula_2)(rh.insula_3)
287	0.99034	(rh.insula_3)(rh.parsopercularis_4)
288	0.99034	(rh.lateraloccipital_3)(rh.superiorparietal_11)
289	0.99034	(rh.paracentral_4)(rh.precentral_14)
290	0.99034	(rh.paracentral_4)(rh.precentral_15)
291	0.99034	(rh.precentral_5)(rh.precentral_6)
292	0.99034	(rh.precuneus_10)(rh.superiorparietal_7)
293	0.99034	(rh.precuneus_8)(rh.superiorparietal_8)
294	0.99034	(rh.superiorfrontal_4)(rh.superiorfrontal_5)
295	0.99034	(rh.superiorparietal_1)(rh.supramarginal_9)
296	0.99034	(rh.superiorparietal_10)(rh.superiorparietal_7)
297	0.99034	(rh.superiorparietal_7)(rh.superiorparietal_8)
298	0.99034	(rh.supramarginal_6)(rh.supramarginal_8)
299	0.99034	(Right-Amygdala)(rh.insula_5)
300	0.99034	(Right-Amygdala)(Right-Thalamus-Proper)

301	0.99034	(Right-Putamen)(rh.insula_2)(rh.insula_3)
302	0.98792	(Left-Caudate)(Left-Putamen)(Left-Thalamus-Proper)(lh.insula_1)
303	0.98792	(Left-Caudate)(Left-Putamen)(lh.insula_1)
304	0.98792	(Left-Caudate)(Left-Putamen)(lh.insula_6)
305	0.98792	(Left-Caudate)(Left-Thalamus-Proper)(lh.insula_1)
306	0.98792	(Left-Caudate)(lh.caudalanteriorcingulate_2)(lh.caudalmiddlefrontal_3)
307	0.98792	(Left-Caudate)(lh.insula_1)
308	0.98792	(Left-Caudate)(lh.insula_6)
309	0.98792	(Left-Caudate)(lh.posteriorcingulate_1)(lh.posteriorcingulate_2)
310	0.98792	(Left-Caudate)(lh.rostralmiddlefrontal_4)
311	0.98792	(Left-Hippocampus)(Left-Pallidum)(Left-Putamen)(Left-Thalamus-Proper)
312	0.98792	(Left-Hippocampus)(Left-Pallidum)(Left-Putamen)
313	0.98792	(Left-Hippocampus)(Left-Pallidum)(Left-Thalamus-Proper)
314	0.98792	(Left-Hippocampus)(Left-Pallidum)
315	0.98792	(Left-Putamen)(lh.caudalmiddlefrontal_4)(lh.precentral_11)
316	0.98792	(Left-Putamen)(lh.precentral_4)
317	0.98792	(Left-Putamen)(lh.rostralmiddlefrontal_4)
318	0.98792	(lh.caudalanteriorcingulate_2)(lh.caudalmiddlefrontal_3)
319	0.98792	(lh.caudalmiddlefrontal_3)(lh.rostralmiddlefrontal_1)
320	0.98792	(lh.fusiform_1)(lh.lingual_8)
321	0.98792	(lh.fusiform_2)(lh.lingual_1)
322	0.98792	(lh.fusiform_5)(lh.lingual_8)
323	0.98792	(lh.lingual_6)(lh.precuneus_11)
324	0.98792	(lh.parstriangularis_3)(lh.rostralmiddlefrontal_1)(lh.rostralmiddlefrontal_9)
325	0.98792	(lh.parstriangularis_3)(lh.rostralmiddlefrontal_9)
326	0.98792	(lh.postcentral_12)(lh.precentral_16)
327	0.98792	(lh.postcentral_13)(lh.postcentral_14)
328	0.98792	(lh.precentral_11)(lh.precentral_14)
329	0.98792	(lh.rostralmiddlefrontal_1)(lh.rostralmiddlefrontal_9)
330	0.98792	(lh.superiorfrontal_12)(lh.superiorfrontal_14)(lh.superiorfrontal_8)
331	0.98792	(lh.superiorfrontal_4)(lh.superiorfrontal_6)(lh.superiorfrontal_7)
332	0.98792	(rh.bankssts_2)(rh.superiortemporal_3)
333	0.98792	(rh.fusiform_2)(rh.fusiform_3)
334	0.98792	(rh.fusiform_3)(rh.fusiform_4)
335	0.98792	(rh.inferiorparietal_10)(rh.inferiorparietal_8)
336	0.98792	(rh.inferiortemporal_7)(rh.middletemporal_1)
337	0.98792	(rh.lateraloccipital_10)(rh.lateraloccipital_9)
338	0.98792	(rh.paracentral_4)(rh.superiorfrontal_16)
339	0.98792	(rh.paracentral_6)(rh.superiorparietal_2)
340	0.98792	(rh.rostralmiddlefrontal_5)(rh.rostralmiddlefrontal_8)
341	0.98792	(rh.superiorfrontal_2)(rh.superiorfrontal_5)
342	0.98792	(rh.superiorfrontal_5)(rh.superiorfrontal_6)(rh.superiorfrontal_7)
343	0.98792	(rh.superiorfrontal_5)(rh.superiorfrontal_8)(rh.superiorfrontal_9)
344	0.98792	(rh.superiorfrontal_5)(rh.superiorfrontal_9)
345	0.98792	(rh.superiorfrontal_6)(rh.superiorfrontal_7)(rh.superiorfrontal_8)
346	0.98792	(rh.supramarginal_1)(rh.supramarginal_8)
347	0.98792	(Right-Amygdala)(Right-Pallidum)(Right-Putamen)(Right-Thalamus-Proper)
348	0.98792	(Right-Amygdala)(Right-Pallidum)(Right-Putamen)
349	0.98792	(Right-Amygdala)(Right-Pallidum)(Right-Thalamus-Proper)
350	0.98792	(Right-Amygdala)(Right-Pallidum)

351	0.98792	(Right-Amygdala)(Right-Putamen)(rh.insula_5)
352	0.98792	(Right-Amygdala)(Right-Putamen)(Right-Thalamus-Proper)
353	0.98792	(Right-Amygdala)(Right-Putamen)
354	0.98792	(Right-Caudate)(rh.rostralanteriorcingulate_2)
355	0.98792	(Right-Hippocampus)(rh.precuneus_2)
356	0.98792	(Right-Hippocampus)(rh.superiortemporal_3)
357	0.98792	(Right-Putamen)(rh.insula_3)(rh.parsopercularis_4)
358	0.98792	(Right-Putamen)(rh.lateralorbitofrontal_2)
359	0.98792	(Right-Putamen)(rh.parstriangularis_1)
360	0.98792	(Right-Putamen)(rh.precentral_10)(rh.precentral_7)
361	0.98792	(Right-Putamen)(rh.precentral_10)
362	0.98792	(Right-Putamen)(Right-Thalamus-Proper)(rh.insula_1)
363	0.98792	(Right-Thalamus-Proper)(rh.insula_1)
364	0.98551	(Left-Amygdala)(lh.insula_4)
365	0.98551	(Left-Caudate)(Left-Putamen)(lh.caudalmiddlefrontal_6)
366	0.98551	(Left-Caudate)(Left-Putamen)(lh.rostralanteriorcingulate_1)
367	0.98551	(Left-Caudate)(lh.caudalanteriorcingulate_2)(lh.rostralanteriorcingulate_2)
368	0.98551	(Left-Caudate)(lh.caudalmiddlefrontal_4)
369	0.98551	(Left-Caudate)(lh.posteriorcingulate_1)(lh.posteriorcingulate_3)
370	0.98551	(Left-Hippocampus)(lh.lingual_8)
371	0.98551	(Left-Putamen)(lh.caudalmiddlefrontal_3)(lh.caudalmiddlefrontal_4)
372	0.98551	(Left-Putamen)(lh.caudalmiddlefrontal_6)
373	0.98551	(Left-Putamen)(lh.rostralanteriorcingulate_1)
374	0.98551	(lh.caudalanteriorcingulate_1)(lh.caudalanteriorcingulate_2)(lh.rostralanteriorcingulate_2)
375	0.98551	(lh.caudalanteriorcingulate_1)(lh.rostralanteriorcingulate_2)
376	0.98551	(lh.fusiform_1)(lh.lateraloccipital_9)
377	0.98551	(lh.fusiform_2)(lh.lateraloccipital_9)
378	0.98551	(lh.lateraloccipital_11)(lh.lateraloccipital_6)
379	0.98551	(lh.lateraloccipital_6)(lh.lateraloccipital_7)
380	0.98551	(lh.lateraloccipital_6)(lh.lingual_1)
381	0.98551	(lh.lingual_1)(lh.lingual_4)
382	0.98551	(lh.parsopercularis_1)(lh.parstriangularis_3)(lh.rostralmiddlefrontal_1)
383	0.98551	(lh.parsopercularis_1)(lh.rostralmiddlefrontal_1)
384	0.98551	(lh.parstriangularis_2)(lh.parstriangularis_3)
385	0.98551	(lh.pericalcarine_2)(lh.pericalcarine_3)
386	0.98551	(lh.posteriorcingulate_1)(lh.posteriorcingulate_3)
387	0.98551	(lh.precuneus_10)(lh.precuneus_6)
388	0.98551	(lh.precuneus_6)(lh.precuneus_9)
389	0.98551	(lh.rostralmiddlefrontal_4)(lh.rostralmiddlefrontal_9)
390	0.98551	(lh.superiorfrontal_11)(lh.superiorfrontal_9)
391	0.98551	(lh.superiorfrontal_14)(lh.superiorfrontal_15)(lh.superiorfrontal_18)
392	0.98551	(lh.superiorfrontal_14)(lh.superiorfrontal_18)
393	0.98551	(lh.supramarginal_1)(lh.supramarginal_2)
394	0.98551	(rh.caudalmiddlefrontal_4)(rh.superiorfrontal_13)
395	0.98551	(rh.fusiform_1)(rh.lateraloccipital_7)(rh.lateraloccipital_8)
396	0.98551	(rh.fusiform_1)(rh.lateraloccipital_7)(rh.lingual_2)
397	0.98551	(rh.inferiorparietal_10)(rh.lateraloccipital_10)
398	0.98551	(rh.inferiortemporal_6)(rh.inferiortemporal_7)
399	0.98551	(rh.isthmuscingulate_2)(rh.precuneus_2)
400	0.98551	(rh.lateraloccipital_1)(rh.lateraloccipital_3)

401	0.98551	(rh.postcentral_1)(rh.postcentral_4)
402	0.98551	(rh.precentral_13)(rh.precentral_15)
403	0.98551	(rh.precentral_4)(rh.precentral_6)
404	0.98551	(rh.precuneus_10)(rh.superiorparietal_1)
405	0.98551	(rh.superiorfrontal_5)(rh.superiorfrontal_7)(rh.superiorfrontal_8)(rh.superiorfrontal_9)
406	0.98551	(rh.superiorfrontal_5)(rh.superiorfrontal_7)(rh.superiorfrontal_9)
407	0.98551	(rh.superiorparietal_1)(rh.supramarginal_1)
408	0.98551	(Right-Accumbens-area)(Right-Thalamus-Proper)
409	0.98551	(Right-Amygdala)(Right-Hippocampus)(Right-Pallidum)(Right-Putamen)(Right-Thalamus-Proper)
410	0.98551	(Right-Amygdala)(Right-Hippocampus)(Right-Pallidum)(Right-Putamen)
411	0.98551	(Right-Amygdala)(Right-Hippocampus)(Right-Pallidum)(Right-Thalamus-Proper)
412	0.98551	(Right-Amygdala)(Right-Hippocampus)(Right-Pallidum)
413	0.98551	(Right-Amygdala)(Right-Hippocampus)(Right-Putamen)(Right-Thalamus-Proper)
414	0.98551	(Right-Amygdala)(Right-Hippocampus)(Right-Putamen)
415	0.98551	(Right-Amygdala)(Right-Hippocampus)(Right-Thalamus-Proper)
416	0.98551	(Right-Amygdala)(Right-Hippocampus)
417	0.98551	(Right-Caudate)(rh.superiorfrontal_11)
418	0.98551	(Right-Caudate)(Right-Hippocampus)(Right-Putamen)(Right-Thalamus-Proper)
419	0.98551	(Right-Caudate)(Right-Hippocampus)(Right-Putamen)
420	0.98551	(Right-Caudate)(Right-Hippocampus)(Right-Thalamus-Proper)
421	0.98551	(Right-Caudate)(Right-Hippocampus)
422	0.98551	(Right-Hippocampus)(rh.inferiorparietal_10)
423	0.98551	(Right-Putamen)(rh.precentral_11)
424	0.98551	(Right-Putamen)(rh.rostralmiddlefrontal_8)
425	0.98309	(Left-Amygdala)(Left-Hippocampus)(Left-Pallidum)(Left-Putamen)(Left-Thalamus-Proper)
426	0.98309	(Left-Amygdala)(Left-Hippocampus)(Left-Pallidum)(Left-Putamen)
427	0.98309	(Left-Amygdala)(Left-Hippocampus)(Left-Pallidum)(Left-Thalamus-Proper)
428	0.98309	(Left-Amygdala)(Left-Hippocampus)(Left-Pallidum)
429	0.98309	(Left-Caudate)(Left-Putamen)(lh.caudalmiddlefrontal_4)
430	0.98309	(Left-Caudate)(Left-Putamen)(lh.posteriorcingulate_1)
431	0.98309	(Left-Caudate)(Left-Putamen)(lh.precentral_4)
432	0.98309	(Left-Caudate)(Left-Thalamus-Proper)(lh.posteriorcingulate_3)
433	0.98309	(Left-Caudate)(lh.caudalanteriorcingulate_2)(lh.superiorfrontal_11)
434	0.98309	(Left-Caudate)(lh.caudalmiddlefrontal_4)(lh.caudalmiddlefrontal_6)
435	0.98309	(Left-Caudate)(lh.caudalmiddlefrontal_5)(lh.caudalmiddlefrontal_6)
436	0.98309	(Left-Caudate)(lh.caudalmiddlefrontal_5)
437	0.98309	(Left-Caudate)(lh.paracentral_5)
438	0.98309	(Left-Caudate)(lh.rostralmiddlefrontal_1)
439	0.98309	(Left-Caudate)(lh.superiorfrontal_14)
440	0.98309	(Left-Hippocampus)(lh.lingual_6)
441	0.98309	(Left-Hippocampus)(lh.superiortemporal_3)
442	0.98309	(Left-Putamen)(lh.posteriorcingulate_1)
443	0.98309	(Left-Putamen)(lh.rostralanteriorcingulate_2)
444	0.98309	(Left-Thalamus-Proper)(lh.posteriorcingulate_3)
445	0.98309	(lh.fusiform_2)(lh.lateraloccipital_7)
446	0.98309	(lh.inferiorparietal_6)(lh.inferiorparietal_8)
447	0.98309	(lh.insula_6)(lh.rostralmiddlefrontal_1)
448	0.98309	(lh.isthmuscingulate_3)(lh.precuneus_10)
449	0.98309	(lh.lateraloccipital_9)(lh.lingual_8)
450	0.98309	(lh.lingual_1)(lh.pericalcarine_3)

451	0.98309	(lh.lingual_4)(lh.lingual_8)
452	0.98309	(lh.parsopercularis_2)(lh.parstriangularis_3)
453	0.98309	(lh.precuneus_6)(lh.precuneus_8)
454	0.98309	(lh.rostralmiddlefrontal_1)(lh.rostralmiddlefrontal_4)(lh.rostralmiddlefrontal_9)
455	0.98309	(lh.rostralmiddlefrontal_3)(lh.rostralmiddlefrontal_4)
456	0.98309	(lh.rostralmiddlefrontal_4)(lh.superiorfrontal_4)
457	0.98309	(lh.rostralmiddlefrontal_6)(lh.rostralmiddlefrontal_9)
458	0.98309	(lh.superiorfrontal_11)(lh.superiorfrontal_12)(lh.superiorfrontal_8)
459	0.98309	(lh.superiorfrontal_11)(lh.superiorfrontal_4)(lh.superiorfrontal_7)
460	0.98309	(lh.superiorfrontal_11)(lh.superiorfrontal_7)
461	0.98309	(lh.superiorfrontal_14)(lh.superiorfrontal_16)
462	0.98309	(lh.superiorfrontal_2)(lh.superiorfrontal_3)
463	0.98309	(lh.superiorfrontal_2)(lh.superiorfrontal_4)
464	0.98309	(rh.fusiform_1)(rh.fusiform_2)
465	0.98309	(rh.fusiform_2)(rh.fusiform_3)(rh.lateraloccipital_10)
466	0.98309	(rh.insula_1)(rh.precentral_7)
467	0.98309	(rh.parsopercularis_4)(rh.rostralmiddlefrontal_3)
468	0.98309	(rh.parstriangularis_1)(rh.parstriangularis_2)
469	0.98309	(rh.parstriangularis_1)(rh.rostralmiddlefrontal_8)
470	0.98309	(rh.postcentral_1)(rh.precentral_1)
471	0.98309	(rh.precuneus_2)(rh.superiorparietal_11)
472	0.98309	(rh.precuneus_4)(rh.superiorparietal_10)
473	0.98309	(rh.rostralmiddlefrontal_8)(rh.superiorfrontal_2)
474	0.98309	(rh.superiorfrontal_10)(rh.superiorfrontal_9)
475	0.98309	(rh.superiorfrontal_13)(rh.superiorfrontal_9)
476	0.98309	(rh.superiorfrontal_2)(rh.superiorfrontal_4)
477	0.98309	(rh.superiorfrontal_5)(rh.superiorfrontal_6)(rh.superiorfrontal_7)(rh.superiorfrontal_8)
478	0.98309	(Right-Caudate)(rh.rostralanteriorcingulate_1)
479	0.98309	(Right-Caudate)(Right-Hippocampus)(Right-Pallidum)(Right-Putamen)(Right-Thalamus-Proper)
480	0.98309	(Right-Caudate)(Right-Hippocampus)(Right-Pallidum)(Right-Putamen)
481	0.98309	(Right-Caudate)(Right-Hippocampus)(Right-Pallidum)(Right-Thalamus-Proper)
482	0.98309	(Right-Caudate)(Right-Hippocampus)(Right-Pallidum)
483	0.98309	(Right-Hippocampus)(rh.insula_5)
484	0.98309	(Right-Hippocampus)(rh.parahippocampal_2)
485	0.98309	(Right-Hippocampus)(rh.superiortemporal_9)
486	0.98309	(Right-Hippocampus)(rh.supramarginal_9)
487	0.98309	(Right-Hippocampus)(Right-Putamen)(rh.insula_5)
488	0.98309	(Right-Putamen)(rh.insula_1)(rh.precentral_7)
489	0.98309	(Right-Putamen)(rh.precentral_11)(rh.precentral_13)
490	0.98068	(Left-Accumbens-area)(Left-Caudate)
491	0.98068	(Left-Amygdala)(lh.insula_5)
492	0.98068	(Left-Hippocampus)(lh.isthmuscingulate_2)
493	0.98068	(Left-Hippocampus)(lh.parahippocampal_3)
494	0.98068	(Left-Putamen)(lh.caudalmiddlefrontal_4)(lh.caudalmiddlefrontal_6)
495	0.98068	(Left-Putamen)(lh.parstriangularis_3)
496	0.98068	(Left-Putamen)(lh.precentral_11)(lh.precentral_7)
497	0.98068	(Left-Putamen)(lh.rostralmiddlefrontal_1)
498	0.98068	(Left-Putamen)(lh.rostralmiddlefrontal_9)
499	0.98068	(lh.caudalanteriorcingulate_2)(lh.superiorfrontal_11)(lh.superiorfrontal_8)
500	0.98068	(lh.caudalanteriorcingulate_2)(lh.superiorfrontal_12)

501	0.98068	(lh.caudalanteriorcingulate_2)(lh.superiorfrontal_14)(lh.superiorfrontal_8)
502	0.98068	(lh.caudalmiddlefrontal_3)(lh.insula_6)
503	0.98068	(lh.inferiorparietal_7)(lh.inferiorparietal_8)
504	0.98068	(lh.insula_6)(lh.parsopercularis_1)(lh.parsopercularis_3)
505	0.98068	(lh.insula_6)(lh.parsopercularis_1)
506	0.98068	(lh.insula_6)(lh.parstriangularis_3)
507	0.98068	(lh.lateraloccipital_2)(lh.lateraloccipital_3)
508	0.98068	(lh.paracentral_5)(lh.precentral_4)
509	0.98068	(lh.parsopercularis_1)(lh.parsopercularis_4)
510	0.98068	(lh.precentral_4)(lh.precentral_7)
511	0.98068	(lh.precuneus_6)(lh.superiorparietal_9)
512	0.98068	(lh.rostralanteriorcingulate_2)(lh.superiorfrontal_4)
513	0.98068	(lh.superiorfrontal_1)(lh.superiorfrontal_3)
514	0.98068	(lh.superiorfrontal_11)(lh.superiorfrontal_4)(lh.superiorfrontal_7)(lh.superiorfrontal_8)
515	0.98068	(lh.superiorfrontal_11)(lh.superiorfrontal_7)(lh.superiorfrontal_8)
516	0.98068	(lh.superiorfrontal_13)(lh.superiorfrontal_14)(lh.superiorfrontal_9)
517	0.98068	(lh.superiorfrontal_14)(lh.superiorfrontal_15)(lh.superiorfrontal_16)
518	0.98068	(rh.caudalmiddlefrontal_3)(rh.caudalmiddlefrontal_4)
519	0.98068	(rh.insula_1)(rh.supramarginal_1)
520	0.98068	(rh.isthmuscingulate_2)(rh.precuneus_7)
521	0.98068	(rh.lateraloccipital_4)(rh.lateraloccipital_7)
522	0.98068	(rh.lateraloccipital_8)(rh.lateraloccipital_9)
523	0.98068	(rh.postcentral_1)(rh.postcentral_3)
524	0.98068	(rh.precentral_1)(rh.precentral_6)
525	0.98068	(rh.precuneus_3)(rh.precuneus_7)
526	0.98068	(rh.precuneus_4)(rh.precuneus_5)
527	0.98068	(rh.rostralmiddlefrontal_10)(rh.superiorfrontal_2)
528	0.98068	(rh.rostralmiddlefrontal_3)(rh.rostralmiddlefrontal_8)
529	0.98068	(rh.superiorfrontal_1)(rh.superiorfrontal_2)
530	0.98068	(rh.superiorfrontal_3)(rh.superiorfrontal_4)
531	0.98068	(rh.supramarginal_1)(rh.supramarginal_9)
532	0.98068	(Right-Amygdala)(rh.insula_6)
533	0.98068	(Right-Amygdala)(Right-Putamen)(rh.insula_6)
534	0.98068	(Right-Caudate)(Right-Putamen)(rh.posteriorcingulate_2)
535	0.98068	(Right-Caudate)(Right-Putamen)(Right-Thalamus-Proper)(rh.insula_1)
536	0.98068	(Right-Caudate)(Right-Thalamus-Proper)(rh.insula_1)
537	0.98068	(Right-Hippocampus)(rh.inferiorparietal_4)
538	0.98068	(Right-Hippocampus)(Right-Putamen)(rh.supramarginal_9)
539	0.98068	(Right-Putamen)(rh.lateralorbitofrontal_6)
540	0.98068	(Right-Putamen)(rh.postcentral_1)
541	0.98068	(Right-Putamen)(rh.posteriorcingulate_2)
542	0.97826	(Left-Accumbens-area)(Left-Caudate)(Left-Pallidum)(Left-Thalamus-Proper)
543	0.97826	(Left-Accumbens-area)(Left-Caudate)(Left-Pallidum)
544	0.97826	(Left-Accumbens-area)(Left-Caudate)(Left-Thalamus-Proper)
545	0.97826	(Left-Accumbens-area)(Left-Pallidum)(Left-Putamen)(Left-Thalamus-Proper)
546	0.97826	(Left-Accumbens-area)(Left-Pallidum)(Left-Putamen)
547	0.97826	(Left-Accumbens-area)(Left-Putamen)(Left-Thalamus-Proper)
548	0.97826	(Left-Accumbens-area)(Left-Putamen)
549	0.97826	(Left-Amygdala)(Left-Putamen)(lh.insula_5)
550	0.97826	(Left-Caudate)(Left-Putamen)(lh.caudalanteriorcingulate_2)(lh.caudalmiddlefrontal_3)

551	0.97826	(Left-Caudate)(Left-Putamen)(lh.rostralmiddlefrontal_4)
552	0.97826	(Left-Caudate)(lh.caudalanteriorcingulate_1)(lh.caudalanteriorcingulate_2)(lh.rostralanteriorcingulate_2)
553	0.97826	(Left-Caudate)(lh.caudalanteriorcingulate_1)(lh.rostralanteriorcingulate_2)
554	0.97826	(Left-Caudate)(lh.caudalanteriorcingulate_2)(lh.superiorfrontal_12)
555	0.97826	(Left-Caudate)(lh.posteriorcingulate_4)
556	0.97826	(Left-Caudate)(lh.precentral_11)(lh.precentral_4)
557	0.97826	(Left-Caudate)(lh.rostralmiddlefrontal_9)
558	0.97826	(Left-Caudate)(lh.superiorfrontal_11)(lh.superiorfrontal_12)
559	0.97826	(Left-Hippocampus)(lh.inferiorparietal_5)
560	0.97826	(Left-Putamen)(lh.caudalanteriorcingulate_2)(lh.caudalmiddlefrontal_3)
561	0.97826	(Left-Putamen)(lh.caudalmiddlefrontal_3)(lh.insula_6)
562	0.97826	(Left-Putamen)(lh.precentral_11)(lh.precentral_14)
563	0.97826	(Left-Putamen)(lh.precentral_11)(lh.precentral_4)
564	0.97826	(lh.caudalmiddlefrontal_1)(lh.caudalmiddlefrontal_3)
565	0.97826	(lh.fusiform_2)(lh.pericalcarine_3)
566	0.97826	(lh.fusiform_5)(lh.fusiform_6)
567	0.97826	(lh.fusiform_5)(lh.lateraloccipital_9)
568	0.97826	(lh.inferiorparietal_6)(lh.inferiorparietal_9)
569	0.97826	(lh.medialorbitofrontal_2)(lh.superiorfrontal_3)
570	0.97826	(lh.paracentral_5)(lh.superiorfrontal_14)
571	0.97826	(lh.parsopercularis_1)(lh.parsopercularis_2)
572	0.97826	(lh.postcentral_8)(lh.supramarginal_9)
573	0.97826	(lh.precentral_5)(lh.precentral_7)
574	0.97826	(lh.rostralanteriorcingulate_1)(lh.rostralanteriorcingulate_2)
575	0.97826	(lh.rostralanteriorcingulate_1)(lh.rostralmiddlefrontal_9)
576	0.97826	(lh.superiorfrontal_10)(lh.superiorfrontal_11)(lh.superiorfrontal_7)
577	0.97826	(lh.superiorfrontal_11)(lh.superiorfrontal_14)(lh.superiorfrontal_8)
578	0.97826	(lh.superiorfrontal_11)(lh.superiorfrontal_14)
579	0.97826	(lh.superiorfrontal_8)(lh.superiorfrontal_9)
580	0.97826	(rh.bankssts_2)(rh.bankssts_3)
581	0.97826	(rh.cuneus_3)(rh.pericalcarine_2)
582	0.97826	(rh.fusiform_1)(rh.fusiform_2)(rh.lateraloccipital_10)
583	0.97826	(rh.inferiorparietal_10)(rh.inferiorparietal_12)
584	0.97826	(rh.inferiorparietal_3)(rh.inferiorparietal_4)
585	0.97826	(rh.inferiorparietal_4)(rh.inferiorparietal_8)
586	0.97826	(rh.insula_1)(rh.insula_2)
587	0.97826	(rh.insula_5)(rh.insula_6)
588	0.97826	(rh.parstriangularis_1)(rh.parstriangularis_4)
589	0.97826	(rh.parstriangularis_1)(rh.rostralmiddlefrontal_3)
590	0.97826	(rh.postcentral_1)(rh.precentral_6)
591	0.97826	(rh.precentral_14)(rh.precentral_15)
592	0.97826	(rh.precuneus_10)(rh.superiorparietal_1)(rh.superiorparietal_7)
593	0.97826	(rh.precuneus_7)(rh.superiorparietal_7)
594	0.97826	(rh.superiorfrontal_10)(rh.superiorfrontal_11)(rh.superiorfrontal_9)
595	0.97826	(rh.superiorfrontal_11)(rh.superiorfrontal_12)
596	0.97826	(rh.superiorfrontal_11)(rh.superiorfrontal_13)(rh.superiorfrontal_9)
597	0.97826	(rh.superiorfrontal_2)(rh.superiorfrontal_6)
598	0.97826	(Right-Accumbens-area)(Right-Pallidum)
599	0.97826	(Right-Amygdala)(Right-Hippocampus)(rh.insula_5)
600	0.97826	(Right-Amygdala)(Right-Hippocampus)(Right-Putamen)(rh.insula_5)

601	0.97826	(Right-Hippocampus)(rh.lingual_7)(rh.precuneus_2)
602	0.97826	(Right-Hippocampus)(rh.superiortemporal_1)
603	0.97826	(Right-Putamen)(rh.insula_1)(rh.insula_2)
604	0.97826	(Right-Putamen)(rh.insula_5)(rh.insula_6)
605	0.97826	(Right-Putamen)(rh.precentral_6)
606	0.97585	(Left-Caudate)(Left-Putamen)(Left-Thalamus-Proper)(lh.precentral_11)
607	0.97585	(Left-Caudate)(Left-Putamen)(lh.insula_1)(lh.precentral_11)
608	0.97585	(Left-Caudate)(Left-Putamen)(lh.rostralanteriorcingulate_2)
609	0.97585	(Left-Caudate)(Left-Thalamus-Proper)(lh.precentral_11)
610	0.97585	(Left-Caudate)(lh.caudalmiddlefrontal_1)
611	0.97585	(Left-Caudate)(lh.insula_1)(lh.precentral_11)
612	0.97585	(Left-Caudate)(lh.parsopercularis_3)
613	0.97585	(Left-Caudate)(lh.superiorfrontal_12)(lh.superiorfrontal_14)
614	0.97585	(Left-Hippocampus)(lh.bankssts_3)
615	0.97585	(Left-Hippocampus)(lh.fusiform_7)
616	0.97585	(Left-Hippocampus)(lh.isthmuscingulate_2)(lh.isthmuscingulate_3)
617	0.97585	(Left-Putamen)(Left-Thalamus-Proper)(lh.precentral_11)
618	0.97585	(Left-Putamen)(lh.insula_1)(lh.supramarginal_1)
619	0.97585	(Left-Putamen)(lh.supramarginal_1)
620	0.97585	(Left-Thalamus-Proper)(lh.precentral_11)
621	0.97585	(lh.caudalmiddlefrontal_3)(lh.rostralmiddlefrontal_3)
622	0.97585	(lh.fusiform_1)(lh.fusiform_2)(lh.lingual_8)
623	0.97585	(lh.fusiform_1)(lh.fusiform_5)(lh.lingual_8)
624	0.97585	(lh.inferiorparietal_1)(lh.pericalcarine_3)
625	0.97585	(lh.lateraloccipital_11)(lh.lingual_1)
626	0.97585	(lh.lateraloccipital_2)(lh.lateraloccipital_3)(lh.pericalcarine_2)
627	0.97585	(lh.paracentral_3)(lh.paracentral_5)
628	0.97585	(lh.pericalcarine_3)(lh.superiorparietal_12)
629	0.97585	(lh.postcentral_12)(lh.postcentral_14)
630	0.97585	(lh.precuneus_10)(lh.precuneus_11)
631	0.97585	(lh.precuneus_11)(lh.precuneus_9)
632	0.97585	(lh.precuneus_3)(lh.precuneus_6)
633	0.97585	(lh.superiorfrontal_10)(lh.superiorfrontal_11)(lh.superiorfrontal_4)(lh.superiorfrontal_7)
634	0.97585	(lh.superiorfrontal_10)(lh.superiorfrontal_11)(lh.superiorfrontal_7)(lh.superiorfrontal_8)
635	0.97585	(rh.caudalmiddlefrontal_4)(rh.parsopercularis_4)
636	0.97585	(rh.caudalmiddlefrontal_4)(rh.rostralmiddlefrontal_1)
637	0.97585	(rh.fusiform_3)(rh.inferiortemporal_7)
638	0.97585	(rh.inferiorparietal_10)(rh.lateraloccipital_8)
639	0.97585	(rh.insula_7)(rh.parsopercularis_4)
640	0.97585	(rh.lateraloccipital_1)(rh.pericalcarine_2)
641	0.97585	(rh.lateraloccipital_3)(rh.pericalcarine_2)
642	0.97585	(rh.lateralorbitofrontal_2)(rh.lateralorbitofrontal_6)
643	0.97585	(rh.lateralorbitofrontal_6)(rh.parstriangularis_1)
644	0.97585	(rh.postcentral_1)(rh.precentral_7)
645	0.97585	(rh.rostralmiddlefrontal_12)(rh.rostralmiddlefrontal_8)
646	0.97585	(rh.superiorfrontal_2)(rh.superiorfrontal_4)(rh.superiorfrontal_5)
647	0.97585	(rh.superiorfrontal_2)(rh.superiorfrontal_7)
648	0.97585	(rh.superiortemporal_3)(rh.superiortemporal_9)
649	0.97585	(rh.supramarginal_7)(rh.supramarginal_8)
650	0.97585	(Right-Accumbens-area)(Right-Pallidum)(Right-Thalamus-Proper)

651	0.97585	(Right-Hippocampus)(rh.bankssts_2)(rh.superiortemporal_3)
652	0.97585	(Right-Hippocampus)(rh.insula_4)
653	0.97585	(Right-Hippocampus)(Right-Putamen)(rh.insula_4)
654	0.97585	(Right-Putamen)(rh.lateralorbitofrontal_2)(rh.lateralorbitofrontal_6)
655	0.97343	(Left-Caudate)(Left-Putamen)(lh.caudalanteriorcingulate_2)(lh.posteriorcingulate_1)
656	0.97343	(Left-Caudate)(lh.caudalanteriorcingulate_2)(lh.posteriorcingulate_3)
657	0.97343	(Left-Caudate)(lh.caudalanteriorcingulate_2)(lh.superiorfrontal_14)
658	0.97343	(Left-Caudate)(lh.caudalmiddlefrontal_4)(lh.precentral_11)
659	0.97343	(Left-Caudate)(lh.medialorbitofrontal_2)
660	0.97343	(Left-Caudate)(lh.rostralanteriorcingulate_1)(lh.rostralanteriorcingulate_2)
661	0.97343	(Left-Caudate)(lh.superiorfrontal_4)
662	0.97343	(Left-Hippocampus)(lh.precuneus_11)
663	0.97343	(Left-Putamen)(lh.caudalanteriorcingulate_2)(lh.posteriorcingulate_1)
664	0.97343	(Left-Putamen)(lh.caudalmiddlefrontal_5)
665	0.97343	(Left-Putamen)(lh.insula_1)(lh.insula_6)
666	0.97343	(Left-Putamen)(lh.rostralanteriorcingulate_1)(lh.rostralmiddlefrontal_9)
667	0.97343	(Left-Thalamus-Proper)(lh.precentral_4)
668	0.97343	(lh.caudalanteriorcingulate_2)(lh.posteriorcingulate_3)
669	0.97343	(lh.fusiform_1)(lh.fusiform_5)(lh.lateraloccipital_9)
670	0.97343	(lh.fusiform_2)(lh.lateraloccipital_11)
671	0.97343	(lh.insula_1)(lh.insula_6)
672	0.97343	(lh.lateraloccipital_2)(lh.pericalcarine_3)
673	0.97343	(lh.lateralorbitofrontal_4)(lh.lateralorbitofrontal_6)
674	0.97343	(lh.lateralorbitofrontal_6)(lh.parstriangularis_3)
675	0.97343	(lh.lingual_4)(lh.pericalcarine_3)
676	0.97343	(lh.precentral_12)(lh.precentral_14)
677	0.97343	(lh.precentral_14)(lh.precentral_16)
678	0.97343	(lh.precuneus_3)(lh.superiorparietal_6)
679	0.97343	(lh.rostralanteriorcingulate_1)(lh.superiorfrontal_4)
680	0.97343	(lh.rostralmiddlefrontal_9)(lh.superiorfrontal_3)
681	0.97343	(lh.superiorfrontal_10)(lh.superiorfrontal_11)(lh.superiorfrontal_4)(lh.superiorfrontal_7)(lh.superiorfrontal_8)
682	0.97343	(lh.superiorfrontal_10)(lh.superiorfrontal_6)
683	0.97343	(lh.superiorfrontal_7)(lh.superiorfrontal_8)(lh.superiorfrontal_9)
684	0.97343	(lh.superiorparietal_11)(lh.superiorparietal_6)
685	0.97343	(lh.superiorparietal_3)(lh.superiorparietal_5)
686	0.97343	(lh.superiortemporal_6)(lh.superiortemporal_8)
687	0.97343	(rh.caudalmiddlefrontal_2)(rh.caudalmiddlefrontal_4)
688	0.97343	(rh.caudalmiddlefrontal_4)(rh.rostralmiddlefrontal_2)
689	0.97343	(rh.inferiorparietal_10)(rh.inferiorparietal_9)
690	0.97343	(rh.insula_2)(rh.superiortemporal_1)
691	0.97343	(rh.isthmuscingulate_2)(rh.precuneus_4)
692	0.97343	(rh.lateraloccipital_4)(rh.lateraloccipital_7)(rh.lateraloccipital_8)
693	0.97343	(rh.lingual_2)(rh.pericalcarine_3)
694	0.97343	(rh.parstriangularis_1)(rh.rostralmiddlefrontal_12)
695	0.97343	(rh.posteriorcingulate_2)(rh.precentral_13)
696	0.97343	(rh.precentral_11)(rh.precentral_12)
697	0.97343	(rh.precuneus_4)(rh.superiorparietal_11)
698	0.97343	(rh.rostralmiddlefrontal_3)(rh.rostralmiddlefrontal_5)(rh.rostralmiddlefrontal_8)
699	0.97343	(rh.superiorfrontal_10)(rh.superiorfrontal_8)
700	0.97343	(rh.superiorfrontal_2)(rh.superiorfrontal_5)(rh.superiorfrontal_6)

701	0.97343	(rh.superiorfrontal_4)(rh.superiorfrontal_6)
702	0.97343	(rh.superiorparietal_1)(rh.superiorparietal_2)
703	0.97343	(rh.supramarginal_5)(rh.supramarginal_8)
704	0.97343	(Right-Accumbens-area)(Right-Caudate)
705	0.97343	(Right-Caudate)(rh.posteriorcingulate_2)(rh.precentral_13)
706	0.97343	(Right-Caudate)(Right-Putamen)(rh.rostralanteriorcingulate_1)
707	0.97343	(Right-Hippocampus)(rh.insula_2)(rh.insula_4)
708	0.97343	(Right-Hippocampus)(rh.isthmuscingulate_2)(rh.precuneus_2)
709	0.97343	(Right-Hippocampus)(rh.precuneus_3)
710	0.97343	(Right-Hippocampus)(Right-Putamen)(rh.insula_2)(rh.insula_4)
711	0.97343	(Right-Putamen)(rh.caudalmiddlefrontal_4)(rh.parsopercularis_4)
712	0.97343	(Right-Putamen)(rh.insula_7)(rh.parsopercularis_4)
713	0.97343	(Right-Putamen)(rh.lateralorbitofrontal_4)
714	0.97343	(Right-Putamen)(rh.parstriangularis_1)(rh.rostralmiddlefrontal_8)
715	0.97343	(Right-Putamen)(rh.rostralanteriorcingulate_1)
716	0.97343	(Right-Putamen)(rh.superiorparietal_1)
717	0.97101	(Left-Amygdala)(Left-Hippocampus)(lh.insula_4)
718	0.97101	(Left-Caudate)(Left-Putamen)(lh.caudalmiddlefrontal_4)(lh.precentral_11)
719	0.97101	(Left-Caudate)(Left-Putamen)(lh.parsopercularis_3)
720	0.97101	(Left-Caudate)(Left-Putamen)(lh.rostralmiddlefrontal_9)
721	0.97101	(Left-Caudate)(Left-Thalamus-Proper)(lh.precentral_4)
722	0.97101	(Left-Caudate)(lh.caudalmiddlefrontal_3)(lh.caudalmiddlefrontal_4)
723	0.97101	(Left-Caudate)(lh.caudalmiddlefrontal_3)(lh.rostralmiddlefrontal_1)
724	0.97101	(Left-Caudate)(lh.parstriangularis_3)
725	0.97101	(Left-Caudate)(lh.rostralmiddlefrontal_1)(lh.rostralmiddlefrontal_4)
726	0.97101	(Left-Caudate)(lh.superiorfrontal_10)
727	0.97101	(Left-Hippocampus)(lh.insula_4)
728	0.97101	(Left-Putamen)(lh.insula_1)(lh.insula_3)
729	0.97101	(Left-Thalamus-Proper)(lh.precentral_7)
730	0.97101	(Left-Thalamus-Proper)(lh.transversetemporal_2)
731	0.97101	(lh.caudalmiddlefrontal_6)(lh.precentral_11)
732	0.97101	(lh.caudalmiddlefrontal_6)(lh.precentral_9)
733	0.97101	(lh.fusiform_2)(lh.lateraloccipital_6)(lh.lingual_1)
734	0.97101	(lh.insula_1)(lh.insula_3)
735	0.97101	(lh.lateralorbitofrontal_6)(lh.rostralmiddlefrontal_9)
736	0.97101	(lh.parsopercularis_1)(lh.parsopercularis_3)(lh.parsopercularis_4)
737	0.97101	(lh.precuneus_8)(lh.superiorparietal_11)
738	0.97101	(lh.rostralmiddlefrontal_1)(lh.rostralmiddlefrontal_3)
739	0.97101	(lh.rostralmiddlefrontal_8)(lh.superiorfrontal_3)
740	0.97101	(lh.superiorfrontal_10)(lh.superiorfrontal_4)(lh.superiorfrontal_6)
741	0.97101	(lh.superiorfrontal_11)(lh.superiorfrontal_12)(lh.superiorfrontal_14)
742	0.97101	(lh.superiorfrontal_14)(lh.superiorfrontal_8)(lh.superiorfrontal_9)
743	0.97101	(lh.superiorfrontal_2)(lh.superiorfrontal_3)(lh.superiorfrontal_4)
744	0.97101	(rh.bankssts_1)(rh.bankssts_2)
745	0.97101	(rh.insula_1)(rh.superiorparietal_1)
746	0.97101	(rh.insula_2)(rh.precentral_7)
747	0.97101	(rh.lateraloccipital_10)(rh.lateraloccipital_8)(rh.lateraloccipital_9)
748	0.97101	(rh.precentral_10)(rh.precentral_13)(rh.precentral_7)
749	0.97101	(rh.precentral_10)(rh.precentral_13)
750	0.97101	(rh.rostralmiddlefrontal_11)(rh.superiorfrontal_2)

751	0.97101	(rh.rostralmiddlefrontal_2)(rh.rostralmiddlefrontal_3)
752	0.97101	(rh.rostralmiddlefrontal_3)(rh.rostralmiddlefrontal_4)
753	0.97101	(rh.superiorfrontal_10)(rh.superiorfrontal_11)(rh.superiorfrontal_8)
754	0.97101	(rh.superiorfrontal_12)(rh.superiorfrontal_15)
755	0.97101	(rh.supramarginal_1)(rh.supramarginal_8)(rh.supramarginal_9)
756	0.97101	(Right-Accumbens-area)(Right-Caudate)(Right-Thalamus-Proper)
757	0.97101	(Right-Amygdala)(rh.insula_5)(rh.insula_6)
758	0.97101	(Right-Amygdala)(Right-Putamen)(rh.insula_5)(rh.insula_6)
759	0.97101	(Right-Caudate)(Right-Putamen)(rh.rostralanteriorcingulate_2)
760	0.97101	(Right-Putamen)(rh.insula_2)(rh.precentral_7)
761	0.97101	(Right-Putamen)(rh.rostralanteriorcingulate_2)
762	0.97101	(Right-Putamen)(rh.rostralmiddlefrontal_12)
763	0.9686	(Left-Caudate)(Left-Putamen)(lh.caudalanteriorcingulate_1)
764	0.9686	(Left-Caudate)(Left-Putamen)(lh.caudalmiddlefrontal_3)(lh.caudalmiddlefrontal_4)
765	0.9686	(Left-Caudate)(Left-Putamen)(lh.precentral_11)(lh.precentral_4)
766	0.9686	(Left-Caudate)(lh.medialorbitofrontal_4)
767	0.9686	(Left-Caudate)(lh.postcentral_8)
768	0.9686	(Left-Caudate)(lh.rostralanteriorcingulate_1)(lh.rostralmiddlefrontal_9)
769	0.9686	(Left-Hippocampus)(Left-Putamen)(Left-Thalamus-Proper)(lh.insula_1)
770	0.9686	(Left-Hippocampus)(Left-Putamen)(lh.insula_1)
771	0.9686	(Left-Hippocampus)(Left-Thalamus-Proper)(lh.insula_1)
772	0.9686	(Left-Hippocampus)(Left-Thalamus-Proper)(lh.isthmuscingulate_3)
773	0.9686	(Left-Hippocampus)(lh.insula_1)
774	0.9686	(Left-Hippocampus)(lh.isthmuscingulate_3)(lh.precuneus_11)
775	0.9686	(Left-Hippocampus)(lh.lingual_6)(lh.lingual_8)
776	0.9686	(Left-Putamen)(Left-Thalamus-Proper)(lh.insula_1)(lh.precentral_11)
777	0.9686	(Left-Putamen)(Left-Thalamus-Proper)(lh.precentral_4)
778	0.9686	(Left-Putamen)(Left-Thalamus-Proper)(lh.precentral_7)
779	0.9686	(Left-Putamen)(lh.caudalanteriorcingulate_1)
780	0.9686	(Left-Putamen)(lh.caudalanteriorcingulate_2)(lh.rostralanteriorcingulate_2)
781	0.9686	(Left-Putamen)(lh.caudalmiddlefrontal_3)(lh.rostralmiddlefrontal_1)
782	0.9686	(Left-Putamen)(lh.lateralorbitofrontal_3)
783	0.9686	(Left-Putamen)(lh.rostralmiddlefrontal_1)(lh.rostralmiddlefrontal_4)
784	0.9686	(Left-Putamen)(lh.rostralmiddlefrontal_4)(lh.rostralmiddlefrontal_9)
785	0.9686	(Left-Thalamus-Proper)(lh.insula_1)(lh.precentral_11)
786	0.9686	(Left-Thalamus-Proper)(lh.isthmuscingulate_3)
787	0.9686	(lh.bankssts_2)(lh.bankssts_3)
788	0.9686	(lh.caudalanteriorcingulate_2)(lh.superiorfrontal_12)(lh.superiorfrontal_14)
789	0.9686	(lh.cuneus_1)(lh.pericalcarine_3)
790	0.9686	(lh.fusiform_2)(lh.lateraloccipital_6)(lh.pericalcarine_3)
791	0.9686	(lh.lateraloccipital_11)(lh.pericalcarine_2)
792	0.9686	(lh.lateralorbitofrontal_6)(lh.parstriangularis_3)(lh.rostralmiddlefrontal_9)
793	0.9686	(lh.lateralorbitofrontal_6)(lh.rostralmiddlefrontal_6)
794	0.9686	(lh.medialorbitofrontal_2)(lh.rostralanteriorcingulate_1)
795	0.9686	(lh.medialorbitofrontal_2)(lh.rostralmiddlefrontal_12)
796	0.9686	(lh.paracentral_1)(lh.precentral_3)
797	0.9686	(lh.parsopercularis_3)(lh.rostralmiddlefrontal_1)
798	0.9686	(lh.parstriangularis_3)(lh.rostralmiddlefrontal_6)
799	0.9686	(lh.pericalcarine_1)(lh.pericalcarine_2)
800	0.9686	(lh.precentral_11)(lh.precentral_4)(lh.precentral_7)

801	0.9686	(lh.precuneus_10)(lh.precuneus_6)(lh.precuneus_9)
802	0.9686	(lh.rostralmiddlefrontal_12)(lh.rostralmiddlefrontal_9)
803	0.9686	(lh.rostralmiddlefrontal_8)(lh.superiorfrontal_3)(lh.superiorfrontal_4)
804	0.9686	(lh.superiorfrontal_10)(lh.superiorfrontal_6)(lh.superiorfrontal_7)
805	0.9686	(lh.superiorfrontal_11)(lh.superiorfrontal_7)(lh.superiorfrontal_9)
806	0.9686	(rh.caudalmiddlefrontal_4)(rh.superiorfrontal_11)
807	0.9686	(rh.fusiform_1)(rh.lingual_4)
808	0.9686	(rh.fusiform_1)(rh.lingual_7)
809	0.9686	(rh.inferiorparietal_7)(rh.inferiorparietal_8)
810	0.9686	(rh.lateraloccipital_4)(rh.pericalcarine_1)
811	0.9686	(rh.lateraloccipital_4)(rh.pericalcarine_3)
812	0.9686	(rh.lateralorbitofrontal_2)(rh.parstriangularis_1)
813	0.9686	(rh.lateralorbitofrontal_6)(rh.rostralmiddlefrontal_12)
814	0.9686	(rh.paracentral_4)(rh.precentral_13)
815	0.9686	(rh.postcentral_1)(rh.supramarginal_7)
816	0.9686	(rh.precentral_11)(rh.precentral_13)(rh.precentral_7)
817	0.9686	(rh.precentral_11)(rh.precentral_7)
818	0.9686	(rh.rostralmiddlefrontal_1)(rh.rostralmiddlefrontal_5)
819	0.9686	(rh.rostralmiddlefrontal_6)(rh.rostralmiddlefrontal_8)
820	0.9686	(rh.superiorfrontal_11)(rh.superiorfrontal_5)(rh.superiorfrontal_8)
821	0.9686	(rh.superiorfrontal_11)(rh.superiorfrontal_5)
822	0.9686	(rh.superiorfrontal_4)(rh.superiorfrontal_5)(rh.superiorfrontal_6)
823	0.9686	(rh.superiortemporal_3)(rh.superiortemporal_6)
824	0.9686	(rh.supramarginal_2)(rh.supramarginal_8)
825	0.9686	(Right-Accumbens-area)(Right-Caudate)(Right-Pallidum)
826	0.9686	(Right-Caudate)(rh.caudalmiddlefrontal_4)(rh.parsopercularis_4)
827	0.9686	(Right-Caudate)(rh.medialorbitofrontal_2)
828	0.9686	(Right-Caudate)(Right-Putamen)(rh.caudalmiddlefrontal_4)(rh.parsopercularis_4)
829	0.9686	(Right-Caudate)(Right-Putamen)(Right-Thalamus-Proper)(rh.precentral_13)
830	0.9686	(Right-Caudate)(Right-Thalamus-Proper)(rh.precentral_13)
831	0.9686	(Right-Hippocampus)(rh.bankssts_2)(rh.inferiorparietal_4)
832	0.9686	(Right-Putamen)(rh.lateralorbitofrontal_6)(rh.parstriangularis_1)
833	0.9686	(Right-Putamen)(Right-Thalamus-Proper)(rh.precentral_13)
834	0.9686	(Right-Thalamus-Proper)(rh.precentral_13)
835	0.96618	(Left-Accumbens-area)(Left-Caudate)(Left-Pallidum)(Left-Putamen)(Left-Thalamus-Proper)
836	0.96618	(Left-Accumbens-area)(Left-Caudate)(Left-Pallidum)(Left-Putamen)
837	0.96618	(Left-Accumbens-area)(Left-Caudate)(Left-Putamen)(Left-Thalamus-Proper)
838	0.96618	(Left-Accumbens-area)(Left-Caudate)(Left-Putamen)
839	0.96618	(Left-Caudate)(Left-Putamen)(Left-Thalamus-Proper)(lh.precentral_4)
840	0.96618	(Left-Caudate)(Left-Putamen)(lh.caudalmiddlefrontal_3)(lh.insula_6)
841	0.96618	(Left-Caudate)(Left-Putamen)(lh.caudalmiddlefrontal_4)(lh.caudalmiddlefrontal_6)
842	0.96618	(Left-Caudate)(Left-Putamen)(lh.rostralmiddlefrontal_1)
843	0.96618	(Left-Caudate)(lh.caudalmiddlefrontal_3)(lh.insula_6)
844	0.96618	(Left-Caudate)(lh.caudalmiddlefrontal_6)(lh.precentral_11)
845	0.96618	(Left-Caudate)(lh.precentral_7)
846	0.96618	(Left-Caudate)(lh.precentral_9)
847	0.96618	(Left-Hippocampus)(lh.fusiform_5)(lh.lingual_8)
848	0.96618	(Left-Putamen)(Left-Thalamus-Proper)(lh.insula_2)
849	0.96618	(Left-Putamen)(lh.caudalmiddlefrontal_4)(lh.caudalmiddlefrontal_5)
850	0.96618	(Left-Putamen)(lh.lateralorbitofrontal_1)

851	0.96618	(Left-Putamen)(lh.lateralorbitofrontal_2)
852	0.96618	(Left-Putamen)(lh.lateralorbitofrontal_6)
853	0.96618	(Left-Putamen)(lh.parsopercularis_1)
854	0.96618	(Left-Putamen)(lh.precentral_4)(lh.precentral_7)
855	0.96618	(Left-Thalamus-Proper)(lh.insula_2)
856	0.96618	(lh.cuneus_2)(lh.pericalcarine_1)
857	0.96618	(lh.fusiform_1)(lh.fusiform_2)(lh.lateraloccipital_9)
858	0.96618	(lh.inferiorparietal_5)(lh.inferiorparietal_6)
859	0.96618	(lh.inferiorparietal_6)(lh.inferiorparietal_7)
860	0.96618	(lh.inferiortemporal_6)(lh.inferiortemporal_7)
861	0.96618	(lh.inferiortemporal_7)(lh.inferiortemporal_8)
862	0.96618	(lh.lateraloccipital_1)(lh.lateraloccipital_2)
863	0.96618	(lh.lateraloccipital_1)(lh.pericalcarine_1)
864	0.96618	(lh.lateralorbitofrontal_4)(lh.parstriangularis_3)
865	0.96618	(lh.medialorbitofrontal_2)(lh.rostralmiddlefrontal_9)
866	0.96618	(lh.postcentral_12)(lh.postcentral_14)(lh.precentral_16)
867	0.96618	(lh.posteriorcingulate_3)(lh.precentral_4)
868	0.96618	(lh.precuneus_6)(lh.precuneus_8)(lh.precuneus_9)
869	0.96618	(lh.rostralmiddlefrontal_12)(lh.superiorfrontal_3)
870	0.96618	(lh.superiorfrontal_10)(lh.superiorfrontal_4)(lh.superiorfrontal_6)(lh.superiorfrontal_7)
871	0.96618	(lh.supramarginal_1)(lh.supramarginal_6)
872	0.96618	(rh.caudalmiddlefrontal_1)(rh.caudalmiddlefrontal_3)
873	0.96618	(rh.fusiform_1)(rh.lateraloccipital_9)
874	0.96618	(rh.fusiform_2)(rh.lingual_7)
875	0.96618	(rh.inferiorparietal_1)(rh.superiorparietal_1)
876	0.96618	(rh.inferiortemporal_4)(rh.middletemporal_4)
877	0.96618	(rh.insula_2)(rh.supramarginal_9)
878	0.96618	(rh.insula_3)(rh.insula_7)
879	0.96618	(rh.paracentral_6)(rh.postcentral_11)
880	0.96618	(rh.paracentral_6)(rh.precentral_15)
881	0.96618	(rh.parstriangularis_2)(rh.parstriangularis_4)
882	0.96618	(rh.precentral_10)(rh.precentral_8)
883	0.96618	(rh.precuneus_10)(rh.superiorparietal_8)
884	0.96618	(rh.superiorfrontal_2)(rh.superiorfrontal_5)(rh.superiorfrontal_7)
885	0.96618	(rh.superiorparietal_11)(rh.superiorparietal_12)
886	0.96618	(rh.supramarginal_7)(rh.supramarginal_9)
887	0.96618	(Right-Accumbens-area)(Right-Caudate)(Right-Pallidum)(Right-Thalamus-Proper)
888	0.96618	(Right-Accumbens-area)(Right-Pallidum)(Right-Putamen)
889	0.96618	(Right-Accumbens-area)(Right-Putamen)
890	0.96618	(Right-Hippocampus)(rh.isthmuscingulate_2)(rh.precuneus_3)
891	0.96618	(Right-Putamen)(rh.insula_2)(rh.supramarginal_9)
892	0.96618	(Right-Putamen)(rh.insula_3)(rh.insula_7)
893	0.96618	(Right-Putamen)(rh.medialorbitofrontal_2)
894	0.96618	(Right-Putamen)(rh.precentral_5)(rh.precentral_6)
895	0.96618	(Right-Putamen)(rh.superiorfrontal_2)
896	0.96618	(Right-Putamen)(Right-Thalamus-Proper)(rh.insula_1)(rh.insula_2)
897	0.96618	(Right-Thalamus-Proper)(rh.insula_1)(rh.insula_2)
898	0.96377	(Left-Caudate)(Left-Putamen)(lh.caudalanteriorcingulate_2)(lh.rostralanteriorcingulate_2)
899	0.96377	(Left-Caudate)(Left-Putamen)(lh.postcentral_8)
900	0.96377	(Left-Caudate)(Left-Putamen)(lh.precentral_9)

901	0.96377	(Left-Caudate)(Left-Putamen)(lh.rostralanteriorcingulate_1)(lh.rostralmiddlefrontal_9)
902	0.96377	(Left-Caudate)(lh.caudalanteriorcingulate_1)(lh.rostralanteriorcingulate_1)
903	0.96377	(Left-Caudate)(lh.caudalmiddlefrontal_4)(lh.caudalmiddlefrontal_5)
904	0.96377	(Left-Caudate)(lh.insula_6)(lh.parsopercularis_3)
905	0.96377	(Left-Caudate)(lh.rostralmiddlefrontal_1)(lh.rostralmiddlefrontal_9)
906	0.96377	(Left-Caudate)(lh.rostralmiddlefrontal_4)(lh.rostralmiddlefrontal_9)
907	0.96377	(Left-Caudate)(lh.superiorfrontal_11)(lh.superiorfrontal_4)
908	0.96377	(Left-Putamen)(lh.insula_6)(lh.rostralmiddlefrontal_1)
909	0.96377	(Left-Putamen)(lh.lateralorbitofrontal_4)(lh.lateralorbitofrontal_6)
910	0.96377	(Left-Putamen)(lh.parstriangularis_3)(lh.rostralmiddlefrontal_1)
911	0.96377	(Left-Putamen)(lh.parstriangularis_3)(lh.rostralmiddlefrontal_9)
912	0.96377	(lh.caudalanteriorcingulate_1)(lh.rostralanteriorcingulate_1)
913	0.96377	(lh.caudalanteriorcingulate_2)(lh.superiorfrontal_11)(lh.superiorfrontal_12)
914	0.96377	(lh.caudalanteriorcingulate_2)(lh.superiorfrontal_11)(lh.superiorfrontal_14)
915	0.96377	(lh.caudalanteriorcingulate_2)(lh.superiorfrontal_12)(lh.superiorfrontal_8)
916	0.96377	(lh.cuneus_1)(lh.superiorparietal_13)
917	0.96377	(lh.cuneus_2)(lh.superiorparietal_14)
918	0.96377	(lh.fusiform_2)(lh.lateraloccipital_6)(lh.lateraloccipital_7)
919	0.96377	(lh.fusiform_6)(lh.fusiform_7)
920	0.96377	(lh.insula_6)(lh.parstriangularis_3)(lh.rostralmiddlefrontal_1)
921	0.96377	(lh.lateraloccipital_3)(lh.pericalcarine_3)
922	0.96377	(lh.lateraloccipital_6)(lh.lingual_1)(lh.pericalcarine_3)
923	0.96377	(lh.lateralorbitofrontal_6)(lh.rostralmiddlefrontal_6)(lh.rostralmiddlefrontal_9)
924	0.96377	(lh.parstriangularis_3)(lh.rostralmiddlefrontal_1)(lh.rostralmiddlefrontal_4)
925	0.96377	(lh.parstriangularis_3)(lh.rostralmiddlefrontal_4)
926	0.96377	(lh.parstriangularis_3)(lh.rostralmiddlefrontal_6)(lh.rostralmiddlefrontal_9)
927	0.96377	(lh.precentral_2)(lh.precentral_4)
928	0.96377	(lh.precuneus_2)(lh.superiorparietal_2)
929	0.96377	(lh.precuneus_6)(lh.superiorparietal_5)
930	0.96377	(lh.superiorfrontal_11)(lh.superiorfrontal_8)(lh.superiorfrontal_9)
931	0.96377	(lh.superiorfrontal_14)(lh.superiorfrontal_15)(lh.superiorfrontal_17)
932	0.96377	(lh.superiorfrontal_14)(lh.superiorfrontal_16)(lh.superiorfrontal_18)
933	0.96377	(lh.superiorfrontal_15)(lh.superiorfrontal_17)
934	0.96377	(rh.caudalmiddlefrontal_4)(rh.insula_3)(rh.parsopercularis_4)
935	0.96377	(rh.fusiform_1)(rh.fusiform_2)(rh.fusiform_3)
936	0.96377	(rh.inferiorparietal_1)(rh.inferiorparietal_6)
937	0.96377	(rh.inferiorparietal_12)(rh.lateraloccipital_8)
938	0.96377	(rh.inferiortemporal_4)(rh.inferiortemporal_5)
939	0.96377	(rh.insula_1)(rh.insula_2)(rh.insula_3)
940	0.96377	(rh.insula_1)(rh.superiorparietal_1)(rh.supramarginal_9)
941	0.96377	(rh.insula_1)(rh.supramarginal_1)(rh.supramarginal_9)
942	0.96377	(rh.insula_4)(rh.insula_6)
943	0.96377	(rh.lateraloccipital_3)(rh.pericalcarine_4)
944	0.96377	(rh.lateraloccipital_8)(rh.lingual_7)
945	0.96377	(rh.lateralorbitofrontal_7)(rh.medialorbitofrontal_2)
946	0.96377	(rh.lingual_2)(rh.lingual_4)
947	0.96377	(rh.medialorbitofrontal_2)(rh.rostralmiddlefrontal_11)
948	0.96377	(rh.paracentral_4)(rh.precentral_14)(rh.precentral_15)
949	0.96377	(rh.paracentral_4)(rh.superiorfrontal_17)
950	0.96377	(rh.parstriangularis_1)(rh.rostralmiddlefrontal_12)(rh.rostralmiddlefrontal_8)

951	0.96377	(rh.parstriangularis_2)(rh.rostralmiddlefrontal_3)
952	0.96377	(rh.rostralmiddlefrontal_10)(rh.rostralmiddlefrontal_8)
953	0.96377	(rh.superiorfrontal_10)(rh.superiorfrontal_8)(rh.superiorfrontal_9)
954	0.96377	(rh.superiorfrontal_11)(rh.superiorfrontal_16)
955	0.96377	(rh.superiorfrontal_12)(rh.superiorfrontal_13)
956	0.96377	(Right-Accumbens-area)(Right-Pallidum)(Right-Putamen)(Right-Thalamus-Proper)
957	0.96377	(Right-Accumbens-area)(Right-Putamen)(Right-Thalamus-Proper)
958	0.96377	(Right-Caudate)(rh.caudalmiddlefrontal_4)(rh.caudalmiddlefrontal_5)
959	0.96377	(Right-Caudate)(rh.caudalmiddlefrontal_5)
960	0.96377	(Right-Caudate)(rh.precentral_11)(rh.precentral_13)
961	0.96377	(Right-Caudate)(rh.precentral_11)
962	0.96377	(Right-Caudate)(rh.precentral_5)
963	0.96377	(Right-Caudate)(rh.superiorfrontal_7)
964	0.96377	(Right-Hippocampus)(rh.superiortemporal_3)(rh.superiortemporal_9)
965	0.96377	(Right-Putamen)(rh.insula_1)(rh.insula_2)(rh.insula_3)
966	0.96377	(Right-Putamen)(rh.insula_4)(rh.insula_6)
967	0.96377	(Right-Putamen)(rh.lateralorbitofrontal_2)(rh.parstriangularis_1)
968	0.96377	(Right-Putamen)(rh.rostralmiddlefrontal_12)(rh.rostralmiddlefrontal_8)
969	0.96377	(Right-Putamen)(rh.superiorparietal_1)(rh.supramarginal_9)
970	0.96377	(Right-Putamen)(rh.supramarginal_1)
971	0.96377	(Right-Thalamus-Proper)(rh.supramarginal_9)
972	0.96135	(Left-Caudate)(Left-Putamen)(lh.caudalanteriorcingulate_1)(lh.caudalanteriorcingulate_2)
973	0.96135	(Left-Caudate)(lh.caudalanteriorcingulate_2)(lh.posteriorcingulate_1)(lh.posteriorcingulate_3)
974	0.96135	(Left-Caudate)(lh.caudalmiddlefrontal_4)(lh.caudalmiddlefrontal_5)(lh.caudalmiddlefrontal_6)
975	0.96135	(Left-Caudate)(lh.medialorbitofrontal_2)(lh.rostralanteriorcingulate_1)
976	0.96135	(Left-Caudate)(lh.posteriorcingulate_3)(lh.precentral_4)
977	0.96135	(Left-Caudate)(lh.precentral_11)(lh.precentral_9)
978	0.96135	(Left-Caudate)(lh.superiorfrontal_10)(lh.superiorfrontal_11)
979	0.96135	(Left-Hippocampus)(Left-Putamen)(Left-Thalamus-Proper)(lh.insula_2)
980	0.96135	(Left-Hippocampus)(Left-Thalamus-Proper)(lh.insula_2)
981	0.96135	(Left-Putamen)(lh.caudalanteriorcingulate_1)(lh.caudalanteriorcingulate_2)
982	0.96135	(Left-Putamen)(lh.caudalmiddlefrontal_1)
983	0.96135	(Left-Putamen)(lh.caudalmiddlefrontal_5)(lh.caudalmiddlefrontal_6)
984	0.96135	(Left-Putamen)(lh.insula_6)(lh.parstriangularis_3)
985	0.96135	(Left-Putamen)(lh.rostralanteriorcingulate_1)(lh.rostralanteriorcingulate_2)
986	0.96135	(Left-Putamen)(lh.rostralmiddlefrontal_1)(lh.rostralmiddlefrontal_9)
987	0.96135	(Left-Putamen)(lh.rostralmiddlefrontal_12)
988	0.96135	(Left-Putamen)(lh.superiorfrontal_12)
989	0.96135	(lh.caudalanteriorcingulate_2)(lh.posteriorcingulate_1)(lh.posteriorcingulate_3)
990	0.96135	(lh.caudalmiddlefrontal_1)(lh.caudalmiddlefrontal_4)
991	0.96135	(lh.caudalmiddlefrontal_4)(lh.caudalmiddlefrontal_6)(lh.precentral_11)
992	0.96135	(lh.cuneus_1)(lh.cuneus_2)
993	0.96135	(lh.fusiform_1)(lh.fusiform_3)
994	0.96135	(lh.fusiform_2)(lh.lateraloccipital_9)(lh.lingual_8)
995	0.96135	(lh.fusiform_2)(lh.lingual_1)(lh.pericalcarine_3)
996	0.96135	(lh.inferiortemporal_6)(lh.middletemporal_3)
997	0.96135	(lh.insula_1)(lh.postcentral_8)
998	0.96135	(lh.insula_1)(lh.supramarginal_2)
999	0.96135	(lh.insula_2)(lh.superiortemporal_3)
1000	0.96135	(lh.insula_6)(lh.parsopercularis_1)(lh.parstriangularis_3)

1001	0.96135	(lh.lateralorbitofrontal_7)(lh.medialorbitofrontal_2)
1002	0.96135	(lh.lingual_3)(lh.pericalcarine_3)
1003	0.96135	(lh.lingual_8)(lh.pericalcarine_3)
1004	0.96135	(lh.medialorbitofrontal_2)(lh.rostralmiddlefrontal_12)(lh.superiorfrontal_3)
1005	0.96135	(lh.paracentral_4)(lh.superiorfrontal_16)
1006	0.96135	(lh.parsopercularis_1)(lh.parsopercularis_2)(lh.parstriangularis_3)
1007	0.96135	(lh.parstriangularis_3)(lh.rostralmiddlefrontal_12)
1008	0.96135	(lh.pericalcarine_1)(lh.superiorparietal_12)
1009	0.96135	(lh.precuneus_3)(lh.superiorparietal_5)
1010	0.96135	(lh.rostralmiddlefrontal_1)(lh.rostralmiddlefrontal_5)
1011	0.96135	(lh.superiorfrontal_11)(lh.superiorfrontal_12)(lh.superiorfrontal_14)(lh.superiorfrontal_8)
1012	0.96135	(lh.superiorfrontal_14)(lh.superiorfrontal_15)(lh.superiorfrontal_16)(lh.superiorfrontal_18)
1013	0.96135	(lh.superiorfrontal_5)(lh.superiorfrontal_6)
1014	0.96135	(lh.superiortemporal_3)(lh.superiortemporal_8)
1015	0.96135	(lh.supramarginal_2)(lh.supramarginal_4)
1016	0.96135	(rh.caudalmiddlefrontal_5)(rh.rostralmiddlefrontal_1)
1017	0.96135	(rh.cuneus_3)(rh.superiorparietal_13)
1018	0.96135	(rh.inferiorparietal_10)(rh.inferiorparietal_12)(rh.inferiorparietal_8)
1019	0.96135	(rh.inferiortemporal_4)(rh.middletemporal_6)
1020	0.96135	(rh.inferiortemporal_5)(rh.inferiortemporal_6)
1021	0.96135	(rh.isthmuscingulate_2)(rh.precuneus_3)(rh.precuneus_7)
1022	0.96135	(rh.lateraloccipital_3)(rh.pericalcarine_3)
1023	0.96135	(rh.lateraloccipital_4)(rh.superiorparietal_11)
1024	0.96135	(rh.lateraloccipital_5)(rh.lateraloccipital_7)
1025	0.96135	(rh.lateralorbitofrontal_6)(rh.lateralorbitofrontal_7)
1026	0.96135	(rh.posteriorcingulate_1)(rh.posteriorcingulate_3)
1027	0.96135	(Right-Caudate)(rh.medialorbitofrontal_3)
1028	0.96135	(Right-Caudate)(rh.posteriorcingulate_1)(rh.posteriorcingulate_3)
1029	0.96135	(Right-Caudate)(Right-Putamen)(rh.precentral_5)
1030	0.96135	(Right-Hippocampus)(Right-Thalamus-Proper)(rh.isthmuscingulate_2)
1031	0.96135	(Right-Putamen)(rh.caudalmiddlefrontal_4)(rh.insula_3)(rh.parsopercularis_4)
1032	0.96135	(Right-Putamen)(rh.parstriangularis_1)(rh.rostralmiddlefrontal_12)
1033	0.96135	(Right-Putamen)(rh.rostralmiddlefrontal_5)
1034	0.96135	(Right-Putamen)(Right-Thalamus-Proper)(rh.supramarginal_9)
1035	0.96135	(Right-Thalamus-Proper)(rh.isthmuscingulate_2)
1036	0.95894	(Left-Caudate)(Left-Putamen)(lh.caudalmiddlefrontal_5)
1037	0.95894	(Left-Caudate)(Left-Putamen)(lh.insula_6)(lh.parsopercularis_3)
1038	0.95894	(Left-Caudate)(Left-Putamen)(lh.posteriorcingulate_3)
1039	0.95894	(Left-Caudate)(Left-Putamen)(lh.precentral_11)(lh.precentral_9)
1040	0.95894	(Left-Caudate)(Left-Putamen)(lh.rostralanteriorcingulate_1)(lh.rostralanteriorcingulate_2)
1041	0.95894	(Left-Caudate)(lh.medialorbitofrontal_5)
1042	0.95894	(Left-Hippocampus)(lh.entorhinal_1)
1043	0.95894	(Left-Hippocampus)(lh.superiortemporal_6)
1044	0.95894	(Left-Putamen)(lh.lateralorbitofrontal_7)
1045	0.95894	(Left-Putamen)(lh.medialorbitofrontal_2)
1046	0.95894	(Left-Putamen)(lh.parsopercularis_1)(lh.parsopercularis_3)
1047	0.95894	(Left-Putamen)(lh.posteriorcingulate_3)
1048	0.95894	(Left-Putamen)(lh.rostralmiddlefrontal_12)(lh.rostralmiddlefrontal_9)
1049	0.95894	(Left-Putamen)(lh.superiorfrontal_11)
1050	0.95894	(lh.caudalanteriorcingulate_1)(lh.rostralanteriorcingulate_1)(lh.rostralanteriorcingulate_2)

1051	0.95894	(lh.caudalmiddlefrontal_1)(lh.caudalmiddlefrontal_5)
1052	0.95894	(lh.caudalmiddlefrontal_2)(lh.caudalmiddlefrontal_3)
1053	0.95894	(lh.fusiform_1)(lh.lateraloccipital_9)(lh.lingual_8)
1054	0.95894	(lh.fusiform_2)(lh.fusiform_3)
1055	0.95894	(lh.fusiform_2)(lh.lingual_4)
1056	0.95894	(lh.fusiform_5)(lh.inferiortemporal_8)
1057	0.95894	(lh.fusiform_7)(lh.inferiortemporal_3)
1058	0.95894	(lh.inferiorparietal_1)(lh.lateraloccipital_6)
1059	0.95894	(lh.inferiorparietal_10)(lh.inferiorparietal_9)
1060	0.95894	(lh.inferiorparietal_4)(lh.lateraloccipital_9)
1061	0.95894	(lh.inferiorparietal_6)(lh.inferiorparietal_8)(lh.inferiorparietal_9)
1062	0.95894	(lh.inferiortemporal_3)(lh.middletemporal_4)
1063	0.95894	(lh.insula_1)(lh.supramarginal_9)
1064	0.95894	(lh.isthmuscingulate_3)(lh.lingual_6)
1065	0.95894	(lh.isthmuscingulate_3)(lh.precuneus_10)(lh.precuneus_11)
1066	0.95894	(lh.lateraloccipital_2)(lh.pericalcarine_2)(lh.pericalcarine_3)
1067	0.95894	(lh.lateraloccipital_7)(lh.pericalcarine_3)
1068	0.95894	(lh.lateralorbitofrontal_4)(lh.lateralorbitofrontal_6)(lh.parstriangularis_3)
1069	0.95894	(lh.lateralorbitofrontal_4)(lh.rostralmiddlefrontal_9)
1070	0.95894	(lh.lateralorbitofrontal_6)(lh.rostralmiddlefrontal_12)
1071	0.95894	(lh.lateralorbitofrontal_7)(lh.rostralmiddlefrontal_12)
1072	0.95894	(lh.lingual_1)(lh.lingual_3)
1073	0.95894	(lh.lingual_1)(lh.pericalcarine_2)(lh.pericalcarine_3)
1074	0.95894	(lh.medialorbitofrontal_2)(lh.rostralmiddlefrontal_9)(lh.superiorfrontal_3)
1075	0.95894	(lh.medialorbitofrontal_2)(lh.superiorfrontal_1)(lh.superiorfrontal_3)
1076	0.95894	(lh.medialorbitofrontal_2)(lh.superiorfrontal_1)
1077	0.95894	(lh.middletemporal_4)(lh.middletemporal_5)
1078	0.95894	(lh.paracentral_1)(lh.paracentral_4)(lh.precentral_3)
1079	0.95894	(lh.paracentral_3)(lh.paracentral_4)
1080	0.95894	(lh.paracentral_5)(lh.postcentral_3)
1081	0.95894	(lh.paracentral_5)(lh.precentral_3)
1082	0.95894	(lh.paracentral_5)(lh.superiorfrontal_15)
1083	0.95894	(lh.parsopercularis_4)(lh.precentral_16)
1084	0.95894	(lh.postcentral_8)(lh.precentral_7)
1085	0.95894	(lh.precuneus_6)(lh.superiorparietal_11)
1086	0.95894	(lh.rostralanteriorcingulate_1)(lh.superiorfrontal_3)
1087	0.95894	(lh.rostralmiddlefrontal_1)(lh.rostralmiddlefrontal_3)(lh.rostralmiddlefrontal_4)
1088	0.95894	(lh.rostralmiddlefrontal_4)(lh.superiorfrontal_10)
1089	0.95894	(lh.superiorfrontal_4)(lh.superiorfrontal_5)(lh.superiorfrontal_6)
1090	0.95894	(rh.caudalmiddlefrontal_4)(rh.rostralmiddlefrontal_3)
1091	0.95894	(rh.caudalmiddlefrontal_4)(rh.superiorfrontal_7)
1092	0.95894	(rh.fusiform_1)(rh.fusiform_2)(rh.fusiform_3)(rh.lateraloccipital_10)
1093	0.95894	(rh.fusiform_1)(rh.lingual_5)(rh.lingual_7)
1094	0.95894	(rh.inferiorparietal_10)(rh.lateraloccipital_10)(rh.lateraloccipital_8)
1095	0.95894	(rh.inferiorparietal_11)(rh.lateraloccipital_8)
1096	0.95894	(rh.inferiorparietal_12)(rh.lateraloccipital_4)
1097	0.95894	(rh.inferiorparietal_12)(rh.superiorparietal_11)
1098	0.95894	(rh.lateraloccipital_8)(rh.pericalcarine_1)
1099	0.95894	(rh.lateralorbitofrontal_2)(rh.lateralorbitofrontal_6)(rh.parstriangularis_1)
1100	0.95894	(rh.lateralorbitofrontal_2)(rh.rostralmiddlefrontal_12)

1101	0.95894	(rh.lateralorbitofrontal_5)(rh.lateralorbitofrontal_7)
1102	0.95894	(rh.lateralorbitofrontal_6)(rh.parstriangularis_1)(rh.rostralmiddlefrontal_12)
1103	0.95894	(rh.paracentral_4)(rh.paracentral_5)
1104	0.95894	(rh.parstriangularis_4)(rh.rostralmiddlefrontal_12)
1105	0.95894	(rh.parstriangularis_4)(rh.rostralmiddlefrontal_3)
1106	0.95894	(rh.precentral_5)(rh.precentral_7)
1107	0.95894	(rh.precuneus_4)(rh.superiorparietal_7)
1108	0.95894	(rh.precuneus_7)(rh.superiorparietal_8)
1109	0.95894	(rh.rostralmiddlefrontal_12)(rh.superiorfrontal_2)
1110	0.95894	(rh.rostralmiddlefrontal_3)(rh.rostralmiddlefrontal_6)
1111	0.95894	(rh.superiorfrontal_10)(rh.superiorfrontal_11)(rh.superiorfrontal_8)(rh.superiorfrontal_9)
1112	0.95894	(rh.superiorfrontal_11)(rh.superiorfrontal_5)(rh.superiorfrontal_8)(rh.superiorfrontal_9)
1113	0.95894	(rh.superiorfrontal_11)(rh.superiorfrontal_5)(rh.superiorfrontal_9)
1114	0.95894	(rh.superiorfrontal_2)(rh.superiorfrontal_6)(rh.superiorfrontal_7)
1115	0.95894	(rh.superiortemporal_1)(rh.superiortemporal_3)
1116	0.95894	(Right-Caudate)(Right-Putamen)(rh.medialorbitofrontal_2)
1117	0.95894	(Right-Hippocampus)(rh.fusiform_8)
1118	0.95894	(Right-Putamen)(rh.caudalmiddlefrontal_4)(rh.caudalmiddlefrontal_5)
1119	0.95894	(Right-Putamen)(rh.caudalmiddlefrontal_5)
1120	0.95894	(Right-Putamen)(rh.lateralorbitofrontal_6)(rh.rostralmiddlefrontal_12)
1121	0.95894	(Right-Putamen)(rh.lateralorbitofrontal_7)
1122	0.95652	(Left-Amygdala)(lh.temporalpole_1)
1123	0.95652	(Left-Caudate)(Left-Putamen)(Left-Thalamus-Proper)(lh.insula_1)(lh.precentral_11)
1124	0.95652	(Left-Caudate)(Left-Putamen)(lh.medialorbitofrontal_2)
1125	0.95652	(Left-Caudate)(Left-Putamen)(lh.precentral_7)
1126	0.95652	(Left-Caudate)(Left-Putamen)(lh.superiorfrontal_11)
1127	0.95652	(Left-Caudate)(Left-Putamen)(lh.superiorfrontal_12)
1128	0.95652	(Left-Caudate)(Left-Thalamus-Proper)(lh.insula_1)(lh.precentral_11)
1129	0.95652	(Left-Caudate)(lh.caudalmiddlefrontal_1)(lh.caudalmiddlefrontal_3)
1130	0.95652	(Left-Caudate)(lh.paracentral_5)(lh.precentral_4)
1131	0.95652	(Left-Caudate)(lh.parstriangularis_3)(lh.rostralmiddlefrontal_1)
1132	0.95652	(Left-Hippocampus)(lh.parahippocampal_1)
1133	0.95652	(Left-Putamen)(lh.caudalmiddlefrontal_6)(lh.precentral_11)
1134	0.95652	(Left-Putamen)(lh.caudalmiddlefrontal_6)(lh.precentral_9)
1135	0.95652	(Left-Putamen)(lh.lateralorbitofrontal_4)(lh.rostralmiddlefrontal_9)
1136	0.95652	(Left-Putamen)(lh.lateralorbitofrontal_6)(lh.rostralmiddlefrontal_9)
1137	0.95652	(Left-Thalamus-Proper)(lh.isthmuscingulate_2)
1138	0.95652	(lh.caudalmiddlefrontal_3)(lh.caudalmiddlefrontal_5)
1139	0.95652	(lh.fusiform_5)(lh.lateraloccipital_9)(lh.lingual_8)
1140	0.95652	(lh.inferiortemporal_8)(lh.lateraloccipital_9)
1141	0.95652	(lh.isthmuscingulate_2)(lh.precuneus_10)
1142	0.95652	(lh.lateraloccipital_1)(lh.pericalcarine_2)
1143	0.95652	(lh.lateralorbitofrontal_6)(lh.lateralorbitofrontal_7)
1144	0.95652	(lh.lateralorbitofrontal_6)(lh.parsorbitalis_1)
1145	0.95652	(lh.lateralorbitofrontal_7)(lh.parstriangularis_3)
1146	0.95652	(lh.lingual_6)(lh.pericalcarine_3)
1147	0.95652	(lh.medialorbitofrontal_2)(lh.rostralmiddlefrontal_12)(lh.rostralmiddlefrontal_9)
1148	0.95652	(lh.parstriangularis_3)(lh.rostralmiddlefrontal_12)(lh.rostralmiddlefrontal_9)
1149	0.95652	(lh.precuneus_8)(lh.precuneus_9)(lh.superiorparietal_11)
1150	0.95652	(lh.rostralanteriorcingulate_1)(lh.rostralanteriorcingulate_2)(lh.superiorfrontal_4)

1151	0.95652	(lh.rostralanteriorcingulate_1)(lh.rostralmiddlefrontal_12)
1152	0.95652	(lh.rostralmiddlefrontal_4)(lh.rostralmiddlefrontal_5)
1153	0.95652	(lh.superiorfrontal_11)(lh.superiorfrontal_14)(lh.superiorfrontal_9)
1154	0.95652	(rh.caudalanteriorcingulate_3)(rh.posteriorcingulate_1)
1155	0.95652	(rh.fusiform_1)(rh.lateraloccipital_10)(rh.lateraloccipital_9)
1156	0.95652	(rh.fusiform_2)(rh.lingual_5)
1157	0.95652	(rh.inferiorparietal_10)(rh.inferiorparietal_11)
1158	0.95652	(rh.isthmuscingulate_2)(rh.precuneus_4)(rh.precuneus_7)
1159	0.95652	(rh.lateraloccipital_4)(rh.lingual_2)
1160	0.95652	(rh.lateralorbitofrontal_7)(rh.rostralmiddlefrontal_12)
1161	0.95652	(rh.paracentral_3)(rh.precentral_15)
1162	0.95652	(rh.parsopercularis_4)(rh.parstriangularis_2)
1163	0.95652	(rh.parstriangularis_1)(rh.parstriangularis_2)(rh.parstriangularis_4)
1164	0.95652	(rh.parstriangularis_1)(rh.rostralmiddlefrontal_3)(rh.rostralmiddlefrontal_8)
1165	0.95652	(rh.pericalcarine_2)(rh.superiorparietal_11)
1166	0.95652	(rh.precentral_5)(rh.precentral_8)
1167	0.95652	(rh.precuneus_7)(rh.superiorparietal_1)
1168	0.95652	(rh.superiorparietal_1)(rh.supramarginal_1)(rh.supramarginal_9)
1169	0.95652	(rh.superiorparietal_1)(rh.supramarginal_8)
1170	0.95652	(rh.superiorparietal_10)(rh.superiorparietal_8)
1171	0.95652	(Right-Accumbens-area)(Right-Caudate)(Right-Pallidum)(Right-Putamen)
1172	0.95652	(Right-Accumbens-area)(Right-Caudate)(Right-Putamen)
1173	0.95652	(Right-Amygdala)(rh.inferiortemporal_1)
1174	0.95652	(Right-Caudate)(rh.caudalanteriorcingulate_3)(rh.posteriorcingulate_1)
1175	0.95652	(Right-Caudate)(rh.precentral_13)(rh.precentral_7)
1176	0.95652	(Right-Caudate)(rh.precentral_7)
1177	0.95652	(Right-Caudate)(Right-Putamen)(rh.precentral_7)
1178	0.95652	(Right-Hippocampus)(rh.middletemporal_4)
1179	0.95652	(Right-Putamen)(rh.insula_1)(rh.supramarginal_1)
1180	0.95652	(Right-Putamen)(rh.lateralorbitofrontal_6)(rh.lateralorbitofrontal_7)
1181	0.95652	(Right-Putamen)(rh.lateralorbitofrontal_7)(rh.medialorbitofrontal_2)
1182	0.95652	(Right-Putamen)(rh.postcentral_1)(rh.precentral_7)
1183	0.95652	(Right-Putamen)(rh.precentral_10)(rh.precentral_13)(rh.precentral_7)
1184	0.95652	(Right-Putamen)(rh.precentral_10)(rh.precentral_13)
1185	0.95652	(Right-Putamen)(rh.precentral_11)(rh.precentral_7)
1186	0.95652	(Right-Putamen)(rh.precentral_5)(rh.precentral_7)
1187	0.95411	(Left-Caudate)(Left-Putamen)(lh.caudalmiddlefrontal_3)(lh.rostralmiddlefrontal_1)
1188	0.95411	(Left-Caudate)(Left-Putamen)(lh.parstriangularis_3)
1189	0.95411	(Left-Caudate)(lh.caudalanteriorcingulate_1)(lh.rostralanteriorcingulate_1)(lh.rostralanteriorcingulate_2)
1190	0.95411	(Left-Caudate)(lh.caudalanteriorcingulate_2)(lh.superiorfrontal_11)(lh.superiorfrontal_12)
1191	0.95411	(Left-Caudate)(lh.insula_6)(lh.rostralmiddlefrontal_1)
1192	0.95411	(Left-Caudate)(lh.lateralorbitofrontal_7)
1193	0.95411	(Left-Caudate)(lh.precentral_11)(lh.precentral_7)
1194	0.95411	(Left-Caudate)(lh.superiorfrontal_11)(lh.superiorfrontal_14)
1195	0.95411	(Left-Caudate)(Right-Caudate)(lh.caudalanteriorcingulate_1)
1196	0.95411	(Left-Hippocampus)(lh.superiortemporal_8)
1197	0.95411	(Left-Putamen)(lh.medialorbitofrontal_5)
1198	0.95411	(Left-Putamen)(lh.parsopercularis_1)(lh.parstriangularis_3)
1199	0.95411	(Left-Putamen)(lh.precentral_11)(lh.precentral_4)(lh.precentral_7)
1200	0.95411	(Left-Putamen)(lh.superiorfrontal_4)

1201	0.95411	(lh.bankssts_2)(lh.inferiortemporal_6)
1202	0.95411	(lh.caudalanteriorcingulate_2)(lh.superiorfrontal_11)(lh.superiorfrontal_14)(lh.superiorfrontal_8)
1203	0.95411	(lh.caudalanteriorcingulate_2)(lh.superiorfrontal_12)(lh.superiorfrontal_14)(lh.superiorfrontal_8)
1204	0.95411	(lh.caudalmiddlefrontal_3)(lh.insula_6)(lh.rostralmiddlefrontal_1)
1205	0.95411	(lh.caudalmiddlefrontal_3)(lh.superiorfrontal_11)
1206	0.95411	(lh.cuneus_1)(lh.pericalcarine_1)
1207	0.95411	(lh.fusiform_2)(lh.lateraloccipital_11)(lh.lateraloccipital_6)
1208	0.95411	(lh.fusiform_3)(lh.fusiform_5)
1209	0.95411	(lh.inferiorparietal_1)(lh.inferiorparietal_3)
1210	0.95411	(lh.lateraloccipital_11)(lh.lateraloccipital_6)(lh.lingual_1)
1211	0.95411	(lh.lateralorbitofrontal_6)(lh.parstriangularis_3)(lh.rostralmiddlefrontal_12)
1212	0.95411	(lh.lateralorbitofrontal_7)(lh.medialorbitofrontal_2)(lh.rostralmiddlefrontal_12)
1213	0.95411	(lh.paracentral_4)(lh.paracentral_5)(lh.precentral_3)
1214	0.95411	(lh.parsopercularis_1)(lh.parsopercularis_3)(lh.rostralmiddlefrontal_1)
1215	0.95411	(lh.parstriangularis_3)(lh.rostralmiddlefrontal_1)(lh.rostralmiddlefrontal_4)(lh.rostralmiddlefrontal_9)
1216	0.95411	(lh.parstriangularis_3)(lh.rostralmiddlefrontal_4)(lh.rostralmiddlefrontal_9)
1217	0.95411	(lh.precuneus_9)(lh.superiorparietal_12)
1218	0.95411	(lh.rostralmiddlefrontal_1)(lh.rostralmiddlefrontal_6)
1219	0.95411	(lh.rostralmiddlefrontal_12)(lh.rostralmiddlefrontal_9)(lh.superiorfrontal_3)
1220	0.95411	(lh.superiorparietal_11)(lh.superiorparietal_5)
1221	0.95411	(rh.caudalmiddlefrontal_4)(rh.superiorfrontal_11)(rh.superiorfrontal_13)
1222	0.95411	(rh.inferiorparietal_12)(rh.precuneus_2)
1223	0.95411	(rh.inferiortemporal_2)(rh.middletemporal_7)
1224	0.95411	(rh.inferiortemporal_7)(rh.middletemporal_2)
1225	0.95411	(rh.insula_3)(rh.precentral_6)
1226	0.95411	(rh.insula_3)(rh.precentral_7)
1227	0.95411	(rh.isthmuscingulate_1)(rh.isthmuscingulate_2)
1228	0.95411	(rh.lateralorbitofrontal_6)(rh.parstriangularis_4)
1229	0.95411	(rh.medialorbitofrontal_2)(rh.rostralmiddlefrontal_13)
1230	0.95411	(rh.middletemporal_4)(rh.middletemporal_7)
1231	0.95411	(rh.posteriorcingulate_1)(rh.posteriorcingulate_2)
1232	0.95411	(rh.precuneus_3)(rh.precuneus_4)
1233	0.95411	(rh.rostralmiddlefrontal_12)(rh.rostralmiddlefrontal_7)
1234	0.95411	(rh.rostralmiddlefrontal_9)(rh.superiorfrontal_2)
1235	0.95411	(rh.superiorfrontal_13)(rh.superiorfrontal_14)
1236	0.95411	(rh.superiorfrontal_14)(rh.superiorfrontal_17)
1237	0.95411	(rh.superiorparietal_10)(rh.superiorparietal_11)
1238	0.95411	(rh.supramarginal_6)(rh.supramarginal_7)
1239	0.95411	(Right-Accumbens-area)(Right-Caudate)(Right-Pallidum)(Right-Putamen)(Right-Thalamus-Proper)
1240	0.95411	(Right-Accumbens-area)(Right-Caudate)(Right-Putamen)(Right-Thalamus-Proper)
1241	0.95411	(Right-Caudate)(lh.caudalanteriorcingulate_1)
1242	0.95411	(Right-Caudate)(rh.caudalmiddlefrontal_4)(rh.superiorfrontal_11)
1243	0.95411	(Right-Caudate)(rh.posteriorcingulate_1)(rh.posteriorcingulate_2)
1244	0.95411	(Right-Caudate)(rh.supramarginal_9)
1245	0.95411	(Right-Caudate)(Right-Putamen)(rh.precentral_13)(rh.precentral_7)
1246	0.95411	(Right-Hippocampus)(rh.parahippocampal_3)
1247	0.95411	(Right-Hippocampus)(Right-Thalamus-Proper)(rh.supramarginal_9)
1248	0.95411	(Right-Putamen)(rh.insula_3)(rh.precentral_7)
1249	0.95411	(Right-Putamen)(rh.lateralorbitofrontal_2)(rh.lateralorbitofrontal_6)(rh.parstriangularis_1)
1250	0.95411	(Right-Putamen)(rh.lateralorbitofrontal_2)(rh.rostralmiddlefrontal_12)

1251	0.95411	(Right-Putamen)(rh.precentral_11)(rh.precentral_13)(rh.precentral_7)
1252	0.95411	(Right-Putamen)(rh.rostralmiddlefrontal_8)(rh.superiorfrontal_2)
1253	0.95411	(Right-Thalamus-Proper)(rh.insula_1)(rh.supramarginal_9)
1254	0.95169	(Left-Caudate)(Left-Putamen)(lh.caudalmiddlefrontal_1)
1255	0.95169	(Left-Caudate)(Left-Putamen)(lh.caudalmiddlefrontal_6)(lh.precentral_11)
1256	0.95169	(Left-Caudate)(lh.medialorbitofrontal_2)(lh.rostralmiddlefrontal_9)
1257	0.95169	(Left-Hippocampus)(lh.transversetemporal_2)
1258	0.95169	(Left-Putamen)(lh.caudalmiddlefrontal_4)(lh.caudalmiddlefrontal_5)(lh.caudalmiddlefrontal_6)
1259	0.95169	(Left-Putamen)(lh.insula_1)(lh.postcentral_8)
1260	0.95169	(lh.fusiform_4)(lh.fusiform_6)
1261	0.95169	(lh.fusiform_7)(lh.inferiortemporal_6)
1262	0.95169	(lh.inferiorparietal_1)(lh.lateraloccipital_7)
1263	0.95169	(lh.insula_6)(lh.parsopercularis_1)(lh.rostralmiddlefrontal_1)
1264	0.95169	(lh.insula_6)(lh.parsopercularis_3)(lh.rostralmiddlefrontal_1)
1265	0.95169	(lh.insula_6)(lh.precentral_14)
1266	0.95169	(lh.isthmuscingulate_3)(lh.precuneus_6)
1267	0.95169	(lh.lateralorbitofrontal_4)(lh.lateralorbitofrontal_6)(lh.rostralmiddlefrontal_9)
1268	0.95169	(lh.lateralorbitofrontal_6)(lh.parstriangularis_1)
1269	0.95169	(lh.lateralorbitofrontal_6)(lh.parstriangularis_3)(lh.rostralmiddlefrontal_6)
1270	0.95169	(lh.lingual_3)(lh.lingual_4)
1271	0.95169	(lh.paracentral_4)(lh.superiorfrontal_15)
1272	0.95169	(lh.parsopercularis_1)(lh.parsopercularis_3)(lh.parstriangularis_3)
1273	0.95169	(lh.parsopercularis_3)(lh.parstriangularis_3)
1274	0.95169	(lh.parstriangularis_1)(lh.parstriangularis_3)
1275	0.95169	(lh.posteriorcingulate_3)(lh.superiorfrontal_14)
1276	0.95169	(lh.precentral_12)(lh.precentral_16)
1277	0.95169	(lh.precuneus_10)(lh.precuneus_11)(lh.precuneus_9)
1278	0.95169	(lh.precuneus_11)(lh.precuneus_6)
1279	0.95169	(lh.precuneus_3)(lh.precuneus_6)(lh.superiorparietal_6)
1280	0.95169	(lh.rostralanteriorcingulate_2)(lh.rostralmiddlefrontal_4)
1281	0.95169	(lh.rostralmiddlefrontal_8)(lh.rostralmiddlefrontal_9)
1282	0.95169	(lh.rostralmiddlefrontal_9)(lh.superiorfrontal_4)
1283	0.95169	(lh.superiorparietal_5)(lh.superiorparietal_7)
1284	0.95169	(rh.caudalanteriorcingulate_1)(rh.caudalanteriorcingulate_2)
1285	0.95169	(rh.fusiform_1)(rh.lateraloccipital_8)(rh.lateraloccipital_9)
1286	0.95169	(rh.inferiorparietal_1)(rh.superiorparietal_7)
1287	0.95169	(rh.inferiortemporal_5)(rh.middletemporal_2)
1288	0.95169	(rh.insula_1)(rh.precentral_11)
1289	0.95169	(rh.insula_4)(rh.insula_5)
1290	0.95169	(rh.paracentral_4)(rh.paracentral_5)(rh.precentral_15)
1291	0.95169	(rh.paracentral_4)(rh.superiorfrontal_16)(rh.superiorfrontal_17)
1292	0.95169	(rh.parstriangularis_1)(rh.parstriangularis_3)
1293	0.95169	(rh.precentral_12)(rh.precentral_13)
1294	0.95169	(rh.precuneus_1)(rh.superiorparietal_10)
1295	0.95169	(rh.rostralmiddlefrontal_10)(rh.rostralmiddlefrontal_8)(rh.superiorfrontal_2)
1296	0.95169	(rh.rostralmiddlefrontal_5)(rh.rostralmiddlefrontal_6)
1297	0.95169	(rh.superiorfrontal_12)(rh.superiorfrontal_16)
1298	0.95169	(rh.superiorfrontal_2)(rh.superiorfrontal_5)(rh.superiorfrontal_6)(rh.superiorfrontal_7)
1299	0.95169	(rh.superiortemporal_6)(rh.superiortemporal_7)
1300	0.95169	(Right-Caudate)(rh.caudalanteriorcingulate_1)(rh.caudalanteriorcingulate_2)

1301	0.95169	(Right-Caudate)(Right-Pallidum)(rh.precentral_13)
1302	0.95169	(Right-Caudate)(Right-Pallidum)(Right-Putamen)(rh.precentral_13)
1303	0.95169	(Right-Caudate)(Right-Putamen)(rh.posteriorcingulate_2)(rh.precentral_13)
1304	0.95169	(Right-Caudate)(Right-Putamen)(rh.precentral_11)
1305	0.95169	(Right-Caudate)(Right-Putamen)(rh.supramarginal_9)
1306	0.95169	(Right-Hippocampus)(rh.insula_2)(rh.superiortemporal_1)
1307	0.95169	(Right-Hippocampus)(Right-Putamen)(Right-Thalamus-Proper)(rh.supramarginal_9)
1308	0.95169	(Right-Pallidum)(rh.precentral_13)
1309	0.95169	(Right-Pallidum)(Right-Putamen)(rh.precentral_13)
1310	0.95169	(Right-Putamen)(rh.posteriorcingulate_2)(rh.precentral_13)
1311	0.95169	(Right-Putamen)(rh.rostralmiddlefrontal_3)
1312	0.95169	(Right-Putamen)(rh.rostralmiddlefrontal_5)(rh.rostralmiddlefrontal_8)
1313	0.95169	(Right-Putamen)(Right-Thalamus-Proper)(rh.insula_1)(rh.supramarginal_9)
1314	0.94928	(Left-Caudate)(Left-Putamen)(Left-Thalamus-Proper)(lh.posteriorcingulate_3)
1315	0.94928	(Left-Caudate)(Left-Putamen)(lh.insula_1)(lh.insula_6)
1316	0.94928	(Left-Caudate)(Left-Putamen)(lh.rostralmiddlefrontal_4)(lh.rostralmiddlefrontal_9)
1317	0.94928	(Left-Caudate)(Left-Thalamus-Proper)(lh.precentral_7)
1318	0.94928	(Left-Caudate)(lh.caudalanteriorcingulate_2)(lh.superiorfrontal_12)(lh.superiorfrontal_14)
1319	0.94928	(Left-Caudate)(lh.insula_1)(lh.insula_6)
1320	0.94928	(Left-Caudate)(lh.parstriangularis_3)(lh.rostralmiddlefrontal_9)
1321	0.94928	(Left-Caudate)(lh.rostralanteriorcingulate_1)(lh.superiorfrontal_4)
1322	0.94928	(Left-Caudate)(lh.rostralanteriorcingulate_2)(lh.superiorfrontal_4)
1323	0.94928	(Left-Caudate)(lh.rostralmiddlefrontal_1)(lh.rostralmiddlefrontal_4)(lh.rostralmiddlefrontal_9)
1324	0.94928	(Left-Caudate)(lh.superiorfrontal_8)
1325	0.94928	(Left-Hippocampus)(lh.fusiform_8)
1326	0.94928	(Left-Hippocampus)(lh.lingual_6)(lh.precuneus_11)
1327	0.94928	(Left-Putamen)(Left-Thalamus-Proper)(lh.posteriorcingulate_3)
1328	0.94928	(Left-Putamen)(lh.lateralorbitofrontal_4)(lh.parstriangularis_3)
1329	0.94928	(Left-Putamen)(lh.lateralorbitofrontal_6)(lh.parstriangularis_3)
1330	0.94928	(Left-Putamen)(lh.lateralorbitofrontal_7)(lh.rostralmiddlefrontal_12)
1331	0.94928	(Left-Putamen)(lh.parsopercularis_3)(lh.rostralmiddlefrontal_1)
1332	0.94928	(Left-Putamen)(lh.rostralmiddlefrontal_1)(lh.rostralmiddlefrontal_4)(lh.rostralmiddlefrontal_9)
1333	0.94928	(Left-Putamen)(lh.superiorparietal_3)
1334	0.94928	(Left-Thalamus-Proper)(lh.posteriorcingulate_2)
1335	0.94928	(lh.cuneus_1)(lh.superiorparietal_14)
1336	0.94928	(lh.inferiorparietal_1)(lh.inferiorparietal_4)
1337	0.94928	(lh.inferiorparietal_1)(lh.precuneus_11)
1338	0.94928	(lh.inferiortemporal_3)(lh.inferiortemporal_6)
1339	0.94928	(lh.insula_1)(lh.supramarginal_1)(lh.supramarginal_2)
1340	0.94928	(lh.lateralorbitofrontal_3)(lh.lateralorbitofrontal_4)
1341	0.94928	(lh.lateralorbitofrontal_6)(lh.rostralmiddlefrontal_12)(lh.rostralmiddlefrontal_9)
1342	0.94928	(lh.medialorbitofrontal_2)(lh.rostralanteriorcingulate_1)(lh.rostralmiddlefrontal_9)
1343	0.94928	(lh.paracentral_5)(lh.precentral_2)(lh.precentral_4)
1344	0.94928	(lh.parstriangularis_3)(lh.rostralanteriorcingulate_1)
1345	0.94928	(lh.precuneus_2)(lh.precuneus_3)
1346	0.94928	(lh.precuneus_6)(lh.superiorparietal_5)(lh.superiorparietal_6)
1347	0.94928	(lh.rostralanteriorcingulate_1)(lh.rostralmiddlefrontal_12)(lh.rostralmiddlefrontal_9)
1348	0.94928	(lh.rostralmiddlefrontal_1)(lh.rostralmiddlefrontal_6)(lh.rostralmiddlefrontal_9)
1349	0.94928	(lh.rostralmiddlefrontal_2)(lh.rostralmiddlefrontal_3)
1350	0.94928	(lh.superiorfrontal_4)(lh.superiorfrontal_5)(lh.superiorfrontal_7)

1351	0.94928	(lh.superiorfrontal_5)(lh.superiorfrontal_7)
1352	0.94928	(lh.superiortemporal_3)(lh.superiortemporal_6)
1353	0.94928	(rh.bankssts_1)(rh.middletemporal_1)
1354	0.94928	(rh.bankssts_3)(rh.superiortemporal_2)
1355	0.94928	(rh.cuneus_2)(rh.pericalcarine_2)
1356	0.94928	(rh.inferiortemporal_5)(rh.middletemporal_6)
1357	0.94928	(rh.inferiortemporal_7)(rh.lateraloccipital_10)
1358	0.94928	(rh.insula_1)(rh.supramarginal_8)(rh.supramarginal_9)
1359	0.94928	(rh.insula_1)(rh.supramarginal_8)
1360	0.94928	(rh.insula_5)(rh.superiortemporal_10)
1361	0.94928	(rh.lateraloccipital_3)(rh.lateraloccipital_4)(rh.superiorparietal_11)
1362	0.94928	(rh.lateraloccipital_8)(rh.lingual_2)
1363	0.94928	(rh.lateralorbitofrontal_2)(rh.lateralorbitofrontal_7)
1364	0.94928	(rh.lateralorbitofrontal_4)(rh.lateralorbitofrontal_5)
1365	0.94928	(rh.lateralorbitofrontal_6)(rh.lateralorbitofrontal_7)(rh.rostralmiddlefrontal_12)
1366	0.94928	(rh.lateralorbitofrontal_6)(rh.rostralmiddlefrontal_13)
1367	0.94928	(rh.middletemporal_1)(rh.middletemporal_2)
1368	0.94928	(rh.paracentral_6)(rh.postcentral_10)
1369	0.94928	(rh.parstriangularis_1)(rh.parstriangularis_4)(rh.rostralmiddlefrontal_12)
1370	0.94928	(rh.precuneus_10)(rh.superiorparietal_7)(rh.superiorparietal_8)
1371	0.94928	(rh.precuneus_2)(rh.precuneus_4)
1372	0.94928	(rh.rostralmiddlefrontal_12)(rh.rostralmiddlefrontal_8)(rh.superiorfrontal_2)
1373	0.94928	(rh.superiorfrontal_14)(rh.superiorfrontal_16)
1374	0.94928	(rh.superiortemporal_3)(rh.transversetemporal_1)
1375	0.94928	(Right-Caudate)(rh.lateralorbitofrontal_4)
1376	0.94928	(Right-Caudate)(Right-Pallidum)(rh.caudalmiddlefrontal_4)
1377	0.94928	(Right-Caudate)(Right-Pallidum)(Right-Putamen)(rh.caudalmiddlefrontal_4)
1378	0.94928	(Right-Caudate)(Right-Putamen)(rh.lateralorbitofrontal_4)
1379	0.94928	(Right-Caudate)(Right-Putamen)(rh.precentral_11)(rh.precentral_13)
1380	0.94928	(Right-Pallidum)(rh.caudalmiddlefrontal_4)
1381	0.94928	(Right-Pallidum)(Right-Putamen)(rh.caudalmiddlefrontal_4)
1382	0.94928	(Right-Putamen)(rh.caudalmiddlefrontal_1)
1383	0.94928	(Right-Putamen)(rh.insula_1)(rh.superiorparietal_1)
1384	0.94928	(Right-Putamen)(rh.insula_4)(rh.insula_5)
1385	0.94928	(Right-Putamen)(rh.lateralorbitofrontal_5)
1386	0.94928	(Right-Putamen)(rh.lateralorbitofrontal_7)(rh.rostralmiddlefrontal_12)
1387	0.94928	(Right-Putamen)(rh.parsopercularis_2)
1388	0.94928	(Right-Putamen)(rh.parstriangularis_1)(rh.rostralmiddlefrontal_12)(rh.rostralmiddlefrontal_8)
1389	0.94928	(Right-Putamen)(rh.supramarginal_1)(rh.supramarginal_9)
1390	0.94686	(Left-Caudate)(Left-Putamen)(Left-Thalamus-Proper)(lh.precentral_7)
1391	0.94686	(Left-Caudate)(Left-Putamen)(lh.caudalmiddlefrontal_5)(lh.caudalmiddlefrontal_6)
1392	0.94686	(Left-Caudate)(Left-Putamen)(lh.medialorbitofrontal_5)
1393	0.94686	(Left-Caudate)(Left-Thalamus-Proper)(lh.posteriorcingulate_2)
1394	0.94686	(Left-Caudate)(lh.caudalmiddlefrontal_3)(lh.superiorfrontal_11)
1395	0.94686	(Left-Caudate)(lh.precentral_4)(lh.precentral_7)
1396	0.94686	(Left-Caudate)(lh.rostralmiddlefrontal_4)(lh.superiorfrontal_4)
1397	0.94686	(Left-Caudate)(lh.superiorfrontal_10)(lh.superiorfrontal_4)
1398	0.94686	(Left-Hippocampus)(lh.insula_2)(lh.superiortemporal_3)
1399	0.94686	(Left-Hippocampus)(lh.parahippocampal_2)
1400	0.94686	(Left-Putamen)(lh.caudalanteriorcingulate_1)(lh.rostralanteriorcingulate_2)

1401	0.94686	(Left-Putamen)(lh.caudalanteriorcingulate_2)(lh.superiorfrontal_11)
1402	0.94686	(Left-Putamen)(lh.insula_6)(lh.parsopercularis_1)
1403	0.94686	(Left-Putamen)(lh.insula_6)(lh.precentral_14)
1404	0.94686	(Left-Putamen)(lh.lateralorbitofrontal_3)(lh.lateralorbitofrontal_4)
1405	0.94686	(Left-Putamen)(lh.medialorbitofrontal_2)(lh.rostralanteriorcingulate_1)
1406	0.94686	(Left-Putamen)(lh.medialorbitofrontal_2)(lh.rostralmiddlefrontal_9)
1407	0.94686	(Left-Putamen)(lh.parstriangularis_3)(lh.rostralmiddlefrontal_1)(lh.rostralmiddlefrontal_9)
1408	0.94686	(Left-Putamen)(lh.precentral_12)
1409	0.94686	(Left-Putamen)(lh.rostralanteriorcingulate_1)(lh.rostralmiddlefrontal_12)
1410	0.94686	(lh.caudalanteriorcingulate_2)(lh.caudalmiddlefrontal_3)(lh.superiorfrontal_11)
1411	0.94686	(lh.caudalanteriorcingulate_2)(lh.superiorfrontal_11)(lh.superiorfrontal_12)(lh.superiorfrontal_8)
1412	0.94686	(lh.caudalmiddlefrontal_3)(lh.caudalmiddlefrontal_4)(lh.caudalmiddlefrontal_5)
1413	0.94686	(lh.fusiform_4)(lh.inferiortemporal_8)
1414	0.94686	(lh.insula_1)(lh.superiorparietal_3)
1415	0.94686	(lh.isthmuscingulate_2)(lh.isthmuscingulate_3)(lh.precuneus_10)
1416	0.94686	(lh.lateraloccipital_1)(lh.lateraloccipital_3)
1417	0.94686	(lh.lateraloccipital_2)(lh.lateraloccipital_6)
1418	0.94686	(lh.lateraloccipital_3)(lh.lingual_1)
1419	0.94686	(lh.lateraloccipital_7)(lh.lateraloccipital_9)
1420	0.94686	(lh.lateralorbitofrontal_4)(lh.parstriangularis_3)(lh.rostralmiddlefrontal_9)
1421	0.94686	(lh.lateralorbitofrontal_6)(lh.lateralorbitofrontal_7)(lh.parstriangularis_3)
1422	0.94686	(lh.lateralorbitofrontal_6)(lh.parstriangularis_3)(lh.rostralmiddlefrontal_6)(lh.rostralmiddlefrontal_9)
1423	0.94686	(lh.lingual_1)(lh.lingual_4)(lh.pericalcarine_3)
1424	0.94686	(lh.paracentral_5)(lh.precuneus_2)
1425	0.94686	(lh.parsorbitalis_1)(lh.parstriangularis_3)
1426	0.94686	(lh.parstriangularis_3)(lh.rostralanteriorcingulate_1)(lh.rostralmiddlefrontal_9)
1427	0.94686	(lh.postcentral_14)(lh.supramarginal_1)
1428	0.94686	(lh.postcentral_14)(lh.supramarginal_4)
1429	0.94686	(lh.posteriorcingulate_4)(lh.superiorparietal_2)
1430	0.94686	(lh.precuneus_5)(lh.precuneus_8)
1431	0.94686	(lh.precuneus_8)(lh.superiorparietal_10)
1432	0.94686	(lh.rostralanteriorcingulate_1)(lh.superiorfrontal_3)(lh.superiorfrontal_4)
1433	0.94686	(lh.rostralmiddlefrontal_7)(lh.rostralmiddlefrontal_9)
1434	0.94686	(rh.bankssts_2)(rh.middletemporal_4)
1435	0.94686	(rh.caudalmiddlefrontal_1)(rh.caudalmiddlefrontal_4)
1436	0.94686	(rh.caudalmiddlefrontal_4)(rh.precentral_7)
1437	0.94686	(rh.fusiform_1)(rh.lateraloccipital_4)
1438	0.94686	(rh.fusiform_1)(rh.lateraloccipital_8)(rh.lingual_2)
1439	0.94686	(rh.inferiorparietal_4)(rh.supramarginal_8)
1440	0.94686	(rh.insula_1)(rh.insula_2)(rh.supramarginal_9)
1441	0.94686	(rh.lateraloccipital_4)(rh.lateraloccipital_5)
1442	0.94686	(rh.lateraloccipital_8)(rh.lingual_5)
1443	0.94686	(rh.lateralorbitofrontal_2)(rh.lateralorbitofrontal_6)(rh.lateralorbitofrontal_7)
1444	0.94686	(rh.lateralorbitofrontal_2)(rh.lateralorbitofrontal_6)(rh.rostralmiddlefrontal_12)
1445	0.94686	(rh.paracentral_3)(rh.paracentral_4)
1446	0.94686	(rh.paracentral_4)(rh.precentral_13)(rh.precentral_15)
1447	0.94686	(rh.postcentral_1)(rh.supramarginal_9)
1448	0.94686	(rh.postcentral_5)(rh.precentral_13)
1449	0.94686	(rh.postcentral_5)(rh.supramarginal_1)
1450	0.94686	(rh.superiorfrontal_11)(rh.superiorfrontal_12)(rh.superiorfrontal_13)

1451	0.94686	(rh.superiorfrontal_2)(rh.superiorfrontal_4)(rh.superiorfrontal_6)
1452	0.94686	(rh.superiorparietal_1)(rh.supramarginal_8)(rh.supramarginal_9)
1453	0.94686	(rh.supramarginal_7)(rh.supramarginal_8)(rh.supramarginal_9)
1454	0.94686	(Right-Caudate)(rh.insula_1)(rh.supramarginal_9)
1455	0.94686	(Right-Hippocampus)(rh.insula_2)(rh.supramarginal_9)
1456	0.94686	(Right-Hippocampus)(Right-Putamen)(rh.insula_2)(rh.supramarginal_9)
1457	0.94686	(Right-Putamen)(rh.caudalmiddlefrontal_4)(rh.precentral_7)
1458	0.94686	(Right-Putamen)(rh.insula_1)(rh.insula_2)(rh.supramarginal_9)
1459	0.94686	(Right-Putamen)(rh.insula_1)(rh.precentral_11)
1460	0.94686	(Right-Putamen)(rh.lateralorbitofrontal_6)(rh.parstriangularis_1)(rh.rostralmiddlefrontal_12)
1461	0.94686	(Right-Putamen)(rh.parsopercularis_2)(rh.parsopercularis_4)
1462	0.94444	(Left-Caudate)(Left-Hippocampus)(Left-Putamen)(Left-Thalamus-Proper)
1463	0.94444	(Left-Caudate)(Left-Hippocampus)(Left-Putamen)
1464	0.94444	(Left-Caudate)(Left-Hippocampus)(Left-Thalamus-Proper)
1465	0.94444	(Left-Caudate)(Left-Hippocampus)
1466	0.94444	(Left-Caudate)(Left-Putamen)(lh.caudalanteriorcingulate_2)(lh.superiorfrontal_11)
1467	0.94444	(Left-Caudate)(Left-Putamen)(lh.lateralorbitofrontal_2)
1468	0.94444	(Left-Caudate)(Left-Putamen)(lh.lateralorbitofrontal_7)
1469	0.94444	(Left-Caudate)(Left-Putamen)(lh.medialorbitofrontal_2)(lh.rostralanteriorcingulate_1)
1470	0.94444	(Left-Caudate)(Left-Putamen)(lh.precentral_11)(lh.precentral_7)
1471	0.94444	(Left-Caudate)(Left-Putamen)(lh.rostralmiddlefrontal_1)(lh.rostralmiddlefrontal_4)
1472	0.94444	(Left-Caudate)(Left-Putamen)(lh.superiorfrontal_4)
1473	0.94444	(Left-Caudate)(lh.caudalmiddlefrontal_4)(lh.caudalmiddlefrontal_6)(lh.precentral_11)
1474	0.94444	(Left-Caudate)(lh.lateralorbitofrontal_2)
1475	0.94444	(Left-Caudate)(lh.paracentral_5)(lh.superiorfrontal_14)
1476	0.94444	(Left-Hippocampus)(lh.isthmuscingulate_3)(lh.lingual_6)
1477	0.94444	(Left-Hippocampus)(lh.lingual_7)
1478	0.94444	(Left-Pallidum)(Left-Putamen)(Left-Thalamus-Proper)(lh.insula_1)
1479	0.94444	(Left-Pallidum)(Left-Putamen)(lh.insula_1)
1480	0.94444	(Left-Pallidum)(Left-Thalamus-Proper)(lh.insula_1)
1481	0.94444	(Left-Pallidum)(lh.insula_1)
1482	0.94444	(Left-Putamen)(lh.caudalmiddlefrontal_1)(lh.caudalmiddlefrontal_3)
1483	0.94444	(Left-Putamen)(lh.caudalmiddlefrontal_4)(lh.caudalmiddlefrontal_6)(lh.precentral_11)
1484	0.94444	(Left-Putamen)(lh.lateralorbitofrontal_6)(lh.rostralmiddlefrontal_12)
1485	0.94444	(Left-Putamen)(lh.lateralorbitofrontal_7)(lh.medialorbitofrontal_2)
1486	0.94444	(Left-Putamen)(lh.medialorbitofrontal_2)(lh.rostralmiddlefrontal_12)
1487	0.94444	(Left-Putamen)(lh.rostralanteriorcingulate_1)(lh.rostralmiddlefrontal_12)(lh.rostralmiddlefrontal_9)
1488	0.94444	(lh.bankssts_3)(lh.inferiorparietal_5)
1489	0.94444	(lh.caudalmiddlefrontal_3)(lh.rostralmiddlefrontal_4)
1490	0.94444	(lh.caudalmiddlefrontal_6)(lh.precentral_11)(lh.precentral_9)
1491	0.94444	(lh.inferiorparietal_5)(lh.inferiorparietal_9)
1492	0.94444	(lh.insula_1)(lh.postcentral_14)
1493	0.94444	(lh.isthmuscingulate_3)(lh.lingual_6)(lh.precuneus_11)
1494	0.94444	(lh.lateraloccipital_11)(lh.lingual_1)(lh.pericalcarine_2)
1495	0.94444	(lh.lateraloccipital_3)(lh.pericalcarine_2)(lh.pericalcarine_3)
1496	0.94444	(lh.lateralorbitofrontal_6)(lh.parstriangularis_3)(lh.rostralmiddlefrontal_12)(lh.rostralmiddlefrontal_9)
1497	0.94444	(lh.paracentral_1)(lh.precentral_2)
1498	0.94444	(lh.paracentral_5)(lh.superiorfrontal_14)(lh.superiorfrontal_15)
1499	0.94444	(lh.pericalcarine_3)(lh.precuneus_11)
1500	0.94444	(lh.postcentral_6)(lh.postcentral_8)

1501	0.94444	(lh.precentral_11)(lh.precentral_13)
1502	0.94444	(lh.precuneus_10)(lh.precuneus_8)
1503	0.94444	(lh.rostralmiddlefrontal_4)(lh.rostralmiddlefrontal_8)
1504	0.94444	(lh.rostralmiddlefrontal_4)(lh.superiorfrontal_10)(lh.superiorfrontal_4)
1505	0.94444	(lh.superiorfrontal_11)(lh.superiorfrontal_7)(lh.superiorfrontal_8)(lh.superiorfrontal_9)
1506	0.94444	(lh.superiorfrontal_13)(lh.superiorfrontal_14)(lh.superiorfrontal_8)
1507	0.94444	(lh.superiorfrontal_13)(lh.superiorfrontal_8)
1508	0.94444	(lh.supramarginal_1)(lh.supramarginal_9)
1509	0.94444	(rh.bankssts_2)(rh.superiortemporal_1)
1510	0.94444	(rh.caudalmiddlefrontal_4)(rh.insula_7)
1511	0.94444	(rh.fusiform_1)(rh.lateraloccipital_10)(rh.lateraloccipital_8)(rh.lateraloccipital_9)
1512	0.94444	(rh.fusiform_1)(rh.lateraloccipital_4)(rh.lateraloccipital_8)
1513	0.94444	(rh.inferiorparietal_10)(rh.inferiorparietal_4)
1514	0.94444	(rh.insula_1)(rh.insula_2)(rh.precentral_7)
1515	0.94444	(rh.insula_1)(rh.insula_3)(rh.precentral_7)
1516	0.94444	(rh.insula_7)(rh.parstriangularis_1)
1517	0.94444	(rh.lateraloccipital_1)(rh.lateraloccipital_3)(rh.pericalcarine_2)
1518	0.94444	(rh.lateraloccipital_7)(rh.pericalcarine_3)
1519	0.94444	(rh.lateralorbitofrontal_6)(rh.parstriangularis_1)(rh.parstriangularis_4)
1520	0.94444	(rh.lateralorbitofrontal_6)(rh.parstriangularis_3)
1521	0.94444	(rh.lateralorbitofrontal_7)(rh.rostralmiddlefrontal_13)
1522	0.94444	(rh.middletemporal_4)(rh.middletemporal_6)
1523	0.94444	(rh.parsopercularis_2)(rh.parstriangularis_2)
1524	0.94444	(rh.parstriangularis_4)(rh.rostralmiddlefrontal_8)
1525	0.94444	(rh.postcentral_1)(rh.precentral_1)(rh.precentral_6)
1526	0.94444	(rh.precuneus_1)(rh.precuneus_4)
1527	0.94444	(rh.precuneus_1)(rh.superiorparietal_12)
1528	0.94444	(rh.precuneus_4)(rh.superiorparietal_1)
1529	0.94444	(rh.precuneus_4)(rh.superiorparietal_10)(rh.superiorparietal_7)
1530	0.94444	(rh.precuneus_7)(rh.superiorparietal_1)(rh.superiorparietal_7)
1531	0.94444	(rh.superiorfrontal_15)(rh.superiorfrontal_16)
1532	0.94444	(rh.superiorparietal_10)(rh.superiorparietal_7)(rh.superiorparietal_8)
1533	0.94444	(rh.superiortemporal_3)(rh.superiortemporal_4)
1534	0.94444	(Right-Caudate)(rh.insula_7)
1535	0.94444	(Right-Caudate)(rh.superiorfrontal_13)
1536	0.94444	(Right-Caudate)(rh.superiorfrontal_2)
1537	0.94444	(Right-Caudate)(Right-Putamen)(rh.insula_1)(rh.supramarginal_9)
1538	0.94444	(Right-Caudate)(Right-Putamen)(rh.insula_7)
1539	0.94444	(Right-Putamen)(rh.caudalmiddlefrontal_4)(rh.insula_7)
1540	0.94444	(Right-Putamen)(rh.insula_1)(rh.insula_2)(rh.precentral_7)
1541	0.94444	(Right-Putamen)(rh.insula_1)(rh.insula_3)(rh.precentral_7)
1542	0.94444	(Right-Putamen)(rh.lateralorbitofrontal_1)
1543	0.94444	(Right-Putamen)(rh.lateralorbitofrontal_2)(rh.lateralorbitofrontal_7)
1544	0.94444	(Right-Putamen)(rh.superiorfrontal_7)
1545	0.94203	(Left-Amygdala)(Left-Putamen)(lh.insula_7)
1546	0.94203	(Left-Amygdala)(lh.insula_7)
1547	0.94203	(Left-Caudate)(Left-Putamen)(lh.caudalanteriorcingulate_1)(lh.rostralanteriorcingulate_2)
1548	0.94203	(Left-Caudate)(Left-Putamen)(lh.lateralorbitofrontal_4)
1549	0.94203	(Left-Caudate)(lh.caudalanteriorcingulate_2)(lh.superiorfrontal_11)(lh.superiorfrontal_14)
1550	0.94203	(Left-Caudate)(lh.caudalmiddlefrontal_3)(lh.caudalmiddlefrontal_5)

1551	0.94203	(Left-Caudate)(lh.insula_6)(lh.parstriangularis_3)
1552	0.94203	(Left-Caudate)(lh.lateralorbitofrontal_4)
1553	0.94203	(Left-Caudate)(lh.lateralorbitofrontal_7)(lh.medialorbitofrontal_2)
1554	0.94203	(Left-Caudate)(lh.superiorfrontal_11)(lh.superiorfrontal_12)(lh.superiorfrontal_14)
1555	0.94203	(Left-Pallidum)(Left-Putamen)(lh.precentral_11)
1556	0.94203	(Left-Pallidum)(lh.precentral_11)
1557	0.94203	(Left-Putamen)(lh.lateralorbitofrontal_4)(lh.lateralorbitofrontal_7)
1558	0.94203	(Left-Putamen)(lh.lateralorbitofrontal_6)(lh.lateralorbitofrontal_7)
1559	0.94203	(Left-Putamen)(lh.lateralorbitofrontal_6)(lh.parstriangularis_3)(lh.rostralmiddlefrontal_9)
1560	0.94203	(Left-Thalamus-Proper)(lh.precentral_11)(lh.precentral_4)
1561	0.94203	(lh.caudalanteriorcingulate_2)(lh.posteriorcingulate_2)
1562	0.94203	(lh.caudalmiddlefrontal_1)(lh.caudalmiddlefrontal_3)(lh.caudalmiddlefrontal_4)
1563	0.94203	(lh.inferiorparietal_5)(lh.superiortemporal_2)
1564	0.94203	(lh.inferiorparietal_6)(lh.inferiorparietal_7)(lh.inferiorparietal_8)
1565	0.94203	(lh.inferiorparietal_8)(lh.superiorparietal_11)
1566	0.94203	(lh.lateralorbitofrontal_2)(lh.lateralorbitofrontal_7)
1567	0.94203	(lh.lateralorbitofrontal_4)(lh.lateralorbitofrontal_7)
1568	0.94203	(lh.lateralorbitofrontal_4)(lh.rostralmiddlefrontal_12)
1569	0.94203	(lh.lateralorbitofrontal_6)(lh.lateralorbitofrontal_7)(lh.rostralmiddlefrontal_12)
1570	0.94203	(lh.lateralorbitofrontal_7)(lh.parstriangularis_3)(lh.rostralmiddlefrontal_12)
1571	0.94203	(lh.lateralorbitofrontal_7)(lh.rostralmiddlefrontal_9)
1572	0.94203	(lh.medialorbitofrontal_2)(lh.rostralanteriorcingulate_1)(lh.rostralmiddlefrontal_12)
1573	0.94203	(lh.medialorbitofrontal_2)(lh.rostralanteriorcingulate_1)(lh.superiorfrontal_3)
1574	0.94203	(lh.medialorbitofrontal_2)(lh.rostralmiddlefrontal_12)(lh.rostralmiddlefrontal_9)(lh.superiorfrontal_3)
1575	0.94203	(lh.paracentral_3)(lh.paracentral_4)(lh.paracentral_5)
1576	0.94203	(lh.paracentral_5)(lh.superiorfrontal_12)
1577	0.94203	(lh.postcentral_8)(lh.precentral_4)
1578	0.94203	(lh.rostralanteriorcingulate_1)(lh.rostralmiddlefrontal_9)(lh.superiorfrontal_3)
1579	0.94203	(lh.superiorparietal_5)(lh.supramarginal_6)
1580	0.94203	(rh.bankssts_1)(rh.inferiortemporal_6)
1581	0.94203	(rh.bankssts_1)(rh.middletemporal_4)
1582	0.94203	(rh.caudalmiddlefrontal_4)(rh.caudalmiddlefrontal_5)(rh.rostralmiddlefrontal_1)
1583	0.94203	(rh.fusiform_1)(rh.lateraloccipital_8)(rh.lingual_5)
1584	0.94203	(rh.lateraloccipital_1)(rh.lateraloccipital_4)
1585	0.94203	(rh.lateraloccipital_4)(rh.lateraloccipital_7)(rh.lingual_2)
1586	0.94203	(rh.lateraloccipital_7)(rh.lateraloccipital_8)(rh.lingual_2)
1587	0.94203	(rh.lateralorbitofrontal_7)(rh.medialorbitofrontal_2)(rh.rostralmiddlefrontal_11)
1588	0.94203	(rh.lateralorbitofrontal_7)(rh.rostralmiddlefrontal_11)
1589	0.94203	(rh.paracentral_3)(rh.precentral_16)
1590	0.94203	(rh.parsopercularis_3)(rh.parsopercularis_4)
1591	0.94203	(rh.postcentral_4)(rh.supramarginal_7)
1592	0.94203	(rh.precentral_1)(rh.precentral_4)
1593	0.94203	(rh.precentral_11)(rh.supramarginal_1)
1594	0.94203	(rh.precentral_13)(rh.superiorfrontal_14)
1595	0.94203	(rh.precuneus_4)(rh.precuneus_7)(rh.superiorparietal_7)
1596	0.94203	(rh.rostralmiddlefrontal_11)(rh.superiorfrontal_1)
1597	0.94203	(rh.rostralmiddlefrontal_4)(rh.rostralmiddlefrontal_5)
1598	0.94203	(rh.superiortemporal_10)(rh.superiortemporal_7)
1599	0.94203	(Right-Caudate)(rh.parstriangularis_1)
1600	0.94203	(Right-Caudate)(Right-Putamen)(rh.posteriorcingulate_1)

1601	0.94203	(Right-Putamen)(rh.insula_1)(rh.superiorparietal_1)(rh.supramarginal_9)
1602	0.94203	(Right-Putamen)(rh.insula_1)(rh.supramarginal_1)(rh.supramarginal_9)
1603	0.94203	(Right-Putamen)(rh.insula_7)(rh.parstriangularis_1)
1604	0.94203	(Right-Putamen)(rh.lateralorbitofrontal_2)(rh.lateralorbitofrontal_6)(rh.lateralorbitofrontal_7)
1605	0.94203	(Right-Putamen)(rh.lateralorbitofrontal_2)(rh.lateralorbitofrontal_6)(rh.rostralmiddlefrontal_12)
1606	0.94203	(Right-Putamen)(rh.lateralorbitofrontal_6)(rh.lateralorbitofrontal_7)(rh.rostralmiddlefrontal_12)
1607	0.94203	(Right-Putamen)(rh.posteriorcingulate_1)
1608	0.93961	(Left-Caudate)(Left-Pallidum)(Left-Putamen)(lh.precentral_11)
1609	0.93961	(Left-Caudate)(Left-Pallidum)(lh.precentral_11)
1610	0.93961	(Left-Caudate)(Left-Putamen)(lh.rostralmiddlefrontal_1)(lh.rostralmiddlefrontal_9)
1611	0.93961	(Left-Caudate)(Left-Thalamus-Proper)(lh.precentral_11)(lh.precentral_4)
1612	0.93961	(Left-Caudate)(lh.caudalanteriorcingulate_2)(lh.caudalmiddlefrontal_3)(lh.superiorfrontal_11)
1613	0.93961	(Left-Caudate)(lh.caudalanteriorcingulate_2)(lh.superiorfrontal_8)
1614	0.93961	(Left-Caudate)(lh.caudalmiddlefrontal_6)(lh.precentral_9)
1615	0.93961	(Left-Caudate)(lh.superiorfrontal_11)(lh.superiorfrontal_8)
1616	0.93961	(Left-Hippocampus)(Left-Thalamus-Proper)(lh.isthmuscingulate_2)
1617	0.93961	(Left-Hippocampus)(lh.bankssts_2)
1618	0.93961	(Left-Putamen)(Left-Thalamus-Proper)(lh.insula_2)(lh.precentral_11)
1619	0.93961	(Left-Putamen)(lh.caudalanteriorcingulate_1)(lh.caudalanteriorcingulate_2)(lh.rostralanteriorcingulate_2)
1620	0.93961	(Left-Putamen)(lh.caudalanteriorcingulate_2)(lh.superiorfrontal_12)
1621	0.93961	(Left-Putamen)(lh.caudalmiddlefrontal_3)(lh.caudalmiddlefrontal_5)
1622	0.93961	(Left-Putamen)(lh.insula_6)(lh.parsopercularis_1)(lh.parsopercularis_3)
1623	0.93961	(Left-Putamen)(lh.lateralorbitofrontal_4)(lh.lateralorbitofrontal_6)(lh.rostralmiddlefrontal_9)
1624	0.93961	(Left-Putamen)(lh.lateralorbitofrontal_6)(lh.rostralmiddlefrontal_12)(lh.rostralmiddlefrontal_9)
1625	0.93961	(Left-Putamen)(lh.parstriangularis_3)(lh.rostralmiddlefrontal_4)
1626	0.93961	(Left-Putamen)(lh.postcentral_8)(lh.precentral_7)
1627	0.93961	(Left-Thalamus-Proper)(lh.insula_2)(lh.precentral_11)
1628	0.93961	(Left-Thalamus-Proper)(lh.precentral_11)(lh.precentral_7)
1629	0.93961	(lh.caudalanteriorcingulate_2)(lh.superiorfrontal_10)
1630	0.93961	(lh.caudalmiddlefrontal_1)(lh.rostralmiddlefrontal_4)
1631	0.93961	(lh.caudalmiddlefrontal_3)(lh.rostralmiddlefrontal_1)(lh.rostralmiddlefrontal_3)
1632	0.93961	(lh.cuneus_1)(lh.pericalcarine_3)(lh.superiorparietal_12)
1633	0.93961	(lh.fusiform_1)(lh.fusiform_5)(lh.lateraloccipital_9)(lh.lingual_8)
1634	0.93961	(lh.fusiform_2)(lh.lateraloccipital_6)(lh.lingual_1)(lh.pericalcarine_3)
1635	0.93961	(lh.fusiform_2)(lh.lingual_1)(lh.lingual_4)
1636	0.93961	(lh.fusiform_2)(lh.lingual_4)(lh.lingual_8)
1637	0.93961	(lh.fusiform_2)(lh.lingual_8)(lh.pericalcarine_3)
1638	0.93961	(lh.insula_1)(lh.superiorparietal_5)
1639	0.93961	(lh.lateraloccipital_6)(lh.lateraloccipital_7)(lh.pericalcarine_3)
1640	0.93961	(lh.lateralorbitofrontal_4)(lh.lateralorbitofrontal_6)(lh.parstriangularis_3)(lh.rostralmiddlefrontal_9)
1641	0.93961	(lh.lateralorbitofrontal_6)(lh.parstriangularis_3)(lh.rostralmiddlefrontal_1)
1642	0.93961	(lh.lateralorbitofrontal_6)(lh.rostralmiddlefrontal_1)
1643	0.93961	(lh.lingual_6)(lh.lingual_7)
1644	0.93961	(lh.paracentral_5)(lh.superiorparietal_2)
1645	0.93961	(lh.precuneus_6)(lh.precuneus_9)(lh.superiorparietal_11)
1646	0.93961	(lh.rostralmiddlefrontal_12)(lh.rostralmiddlefrontal_6)
1647	0.93961	(lh.superiorfrontal_12)(lh.superiorfrontal_14)(lh.superiorfrontal_15)
1648	0.93961	(lh.superiorfrontal_12)(lh.superiorfrontal_15)
1649	0.93961	(rh.bankssts_1)(rh.inferiortemporal_5)
1650	0.93961	(rh.caudalanteriorcingulate_1)(rh.rostralanteriorcingulate_2)

1651	0.93961	(rh.cuneus_4)(rh.lateraloccipital_1)
1652	0.93961	(rh.fusiform_2)(rh.lateraloccipital_8)
1653	0.93961	(rh.fusiform_8)(rh.inferiortemporal_1)
1654	0.93961	(rh.inferiorparietal_6)(rh.inferiorparietal_8)
1655	0.93961	(rh.inferiortemporal_3)(rh.inferiortemporal_4)
1656	0.93961	(rh.insula_1)(rh.superiorparietal_1)(rh.supramarginal_1)
1657	0.93961	(rh.lateraloccipital_10)(rh.lingual_5)
1658	0.93961	(rh.lateraloccipital_4)(rh.pericalcarine_4)
1659	0.93961	(rh.lateralorbitofrontal_2)(rh.parstriangularis_1)(rh.rostralmiddlefrontal_12)
1660	0.93961	(rh.medialorbitofrontal_2)(rh.superiorfrontal_2)
1661	0.93961	(rh.parsopercularis_2)(rh.parsopercularis_3)(rh.parsopercularis_4)
1662	0.93961	(rh.pericalcarine_1)(rh.superiorparietal_11)
1663	0.93961	(rh.rostralmiddlefrontal_1)(rh.rostralmiddlefrontal_2)
1664	0.93961	(rh.superiorfrontal_2)(rh.superiorfrontal_4)(rh.superiorfrontal_5)(rh.superiorfrontal_6)
1665	0.93961	(rh.superiorfrontal_3)(rh.superiorfrontal_5)
1666	0.93961	(Right-Hippocampus)(rh.entorhinal_1)
1667	0.93961	(Right-Putamen)(rh.lateralorbitofrontal_5)(rh.lateralorbitofrontal_7)
1668	0.9372	(Left-Caudate)(Left-Putamen)(lh.caudalanteriorcingulate_2)(lh.superiorfrontal_12)
1669	0.9372	(Left-Caudate)(Left-Putamen)(lh.caudalmiddlefrontal_4)(lh.caudalmiddlefrontal_5)
1670	0.9372	(Left-Caudate)(Left-Putamen)(lh.insula_6)(lh.rostralmiddlefrontal_1)
1671	0.9372	(Left-Caudate)(lh.caudalanteriorcingulate_2)(lh.posteriorcingulate_2)
1672	0.9372	(Left-Caudate)(lh.caudalanteriorcingulate_2)(lh.rostralanteriorcingulate_1)
1673	0.9372	(Left-Caudate)(lh.parstriangularis_3)(lh.rostralmiddlefrontal_1)(lh.rostralmiddlefrontal_9)
1674	0.9372	(Left-Caudate)(lh.posteriorcingulate_3)(lh.superiorfrontal_14)
1675	0.9372	(Left-Caudate)(lh.rostralanteriorcingulate_2)(lh.rostralmiddlefrontal_4)
1676	0.9372	(Left-Caudate)(lh.rostralmiddlefrontal_12)
1677	0.9372	(Left-Caudate)(lh.superiorfrontal_12)(lh.superiorfrontal_8)
1678	0.9372	(Left-Caudate)(lh.superiorfrontal_14)(lh.superiorfrontal_8)
1679	0.9372	(Left-Putamen)(Left-Thalamus-Proper)(lh.precentral_11)(lh.precentral_4)
1680	0.9372	(Left-Putamen)(Left-Thalamus-Proper)(lh.precentral_11)(lh.precentral_7)
1681	0.9372	(Left-Putamen)(lh.lateralorbitofrontal_2)(lh.lateralorbitofrontal_7)
1682	0.9372	(Left-Putamen)(lh.lateralorbitofrontal_4)(lh.rostralmiddlefrontal_12)
1683	0.9372	(Left-Putamen)(lh.parstriangularis_3)(lh.rostralmiddlefrontal_12)
1684	0.9372	(Left-Thalamus-Proper)(lh.paracentral_5)
1685	0.9372	(Left-Thalamus-Proper)(lh.posteriorcingulate_4)
1686	0.9372	(Left-Thalamus-Proper)(lh.precentral_4)(lh.precentral_7)
1687	0.9372	(lh.bankssts_3)(lh.superiortemporal_2)
1688	0.9372	(lh.caudalanteriorcingulate_2)(lh.rostralanteriorcingulate_1)(lh.rostralanteriorcingulate_2)
1689	0.9372	(lh.caudalanteriorcingulate_2)(lh.rostralanteriorcingulate_1)
1690	0.9372	(lh.caudalanteriorcingulate_2)(lh.superiorfrontal_11)(lh.superiorfrontal_12)(lh.superiorfrontal_14)
1691	0.9372	(lh.fusiform_2)(lh.lateraloccipital_11)(lh.lingual_1)
1692	0.9372	(lh.fusiform_7)(lh.middletemporal_4)
1693	0.9372	(lh.inferiorparietal_1)(lh.inferiorparietal_8)
1694	0.9372	(lh.insula_6)(lh.parsopercularis_3)(lh.parstriangularis_3)
1695	0.9372	(lh.isthmuscingulate_2)(lh.precuneus_6)
1696	0.9372	(lh.lateraloccipital_3)(lh.lateraloccipital_6)
1697	0.9372	(lh.lateraloccipital_7)(lh.lateraloccipital_8)
1698	0.9372	(lh.lateralorbitofrontal_4)(lh.lateralorbitofrontal_6)(lh.rostralmiddlefrontal_12)
1699	0.9372	(lh.lateralorbitofrontal_6)(lh.parsorbitalis_1)(lh.parstriangularis_3)
1700	0.9372	(lh.lateralorbitofrontal_7)(lh.medialorbitofrontal_4)

1701	0.9372	(lh.medialorbitofrontal_1)(lh.medialorbitofrontal_2)
1702	0.9372	(lh.postcentral_8)(lh.precentral_14)
1703	0.9372	(lh.postcentral_8)(lh.supramarginal_4)
1704	0.9372	(lh.precentral_2)(lh.precentral_3)
1705	0.9372	(lh.rostralmiddlefrontal_1)(lh.rostralmiddlefrontal_7)
1706	0.9372	(lh.rostralmiddlefrontal_4)(lh.superiorfrontal_11)
1707	0.9372	(lh.superiorfrontal_11)(lh.superiorfrontal_14)(lh.superiorfrontal_8)(lh.superiorfrontal_9)
1708	0.9372	(lh.supramarginal_4)(lh.supramarginal_9)
1709	0.9372	(rh.bankssts_2)(rh.superiortemporal_9)
1710	0.9372	(rh.caudalmiddlefrontal_1)(rh.caudalmiddlefrontal_2)
1711	0.9372	(rh.fusiform_3)(rh.fusiform_6)
1712	0.9372	(rh.inferiorparietal_4)(rh.supramarginal_9)
1713	0.9372	(rh.inferiortemporal_2)(rh.middletemporal_5)
1714	0.9372	(rh.inferiortemporal_6)(rh.middletemporal_2)
1715	0.9372	(rh.lateraloccipital_1)(rh.superiorparietal_13)
1716	0.9372	(rh.lateraloccipital_3)(rh.pericalcarine_2)(rh.superiorparietal_11)
1717	0.9372	(rh.lateraloccipital_7)(rh.lingual_1)
1718	0.9372	(rh.lateralorbitofrontal_5)(rh.medialorbitofrontal_2)
1719	0.9372	(rh.medialorbitofrontal_2)(rh.rostralmiddlefrontal_12)
1720	0.9372	(rh.parsorbitalis_1)(rh.parstriangularis_1)
1721	0.9372	(rh.parstriangularis_1)(rh.parstriangularis_2)(rh.rostralmiddlefrontal_3)
1722	0.9372	(rh.postcentral_10)(rh.superiorparietal_1)
1723	0.9372	(rh.precentral_6)(rh.precentral_8)
1724	0.9372	(rh.precuneus_3)(rh.precuneus_4)(rh.precuneus_7)
1725	0.9372	(rh.precuneus_7)(rh.superiorparietal_10)
1726	0.9372	(rh.precuneus_7)(rh.superiorparietal_7)(rh.superiorparietal_8)
1727	0.9372	(rh.rostralmiddlefrontal_8)(rh.rostralmiddlefrontal_9)
1728	0.9372	(rh.rostralmiddlefrontal_9)(rh.superiorfrontal_7)
1729	0.9372	(Right-Caudate)(rh.caudalanteriorcingulate_1)(rh.rostralanteriorcingulate_2)
1730	0.9372	(Right-Caudate)(rh.caudalmiddlefrontal_4)(rh.superiorfrontal_13)
1731	0.9372	(Right-Caudate)(Right-Pallidum)(Right-Putamen)(Right-Thalamus-Proper)(rh.precentral_13)
1732	0.9372	(Right-Caudate)(Right-Pallidum)(Right-Thalamus-Proper)(rh.precentral_13)
1733	0.9372	(Right-Pallidum)(Right-Putamen)(Right-Thalamus-Proper)(rh.precentral_13)
1734	0.9372	(Right-Pallidum)(Right-Thalamus-Proper)(rh.precentral_13)
1735	0.9372	(Right-Putamen)(rh.lateralorbitofrontal_4)(rh.lateralorbitofrontal_5)
1736	0.9372	(Right-Putamen)(rh.parsopercularis_4)(rh.rostralmiddlefrontal_3)
1737	0.9372	(Right-Putamen)(rh.postcentral_1)(rh.precentral_6)
1738	0.93478	(Left-Caudate)(Left-Hippocampus)(Left-Pallidum)(Left-Putamen)(Left-Thalamus-Proper)
1739	0.93478	(Left-Caudate)(Left-Hippocampus)(Left-Pallidum)(Left-Putamen)
1740	0.93478	(Left-Caudate)(Left-Hippocampus)(Left-Pallidum)(Left-Thalamus-Proper)
1741	0.93478	(Left-Caudate)(Left-Hippocampus)(Left-Pallidum)
1742	0.93478	(Left-Caudate)(Left-Pallidum)(Left-Putamen)(Left-Thalamus-Proper)(lh.insula_1)
1743	0.93478	(Left-Caudate)(Left-Pallidum)(Left-Putamen)(lh.insula_1)
1744	0.93478	(Left-Caudate)(Left-Pallidum)(Left-Thalamus-Proper)(lh.insula_1)
1745	0.93478	(Left-Caudate)(Left-Pallidum)(lh.insula_1)
1746	0.93478	(Left-Caudate)(Left-Putamen)(Left-Thalamus-Proper)(lh.precentral_11)(lh.precentral_4)
1747	0.93478	(Left-Caudate)(Left-Putamen)(lh.caudalanteriorcingulate_1)(lh.caudalanteriorcingulate_2)(lh.rostralanteriorcingulate_2)
1748	0.93478	(Left-Caudate)(Left-Putamen)(lh.medialorbitofrontal_2)(lh.rostralmiddlefrontal_9)
1749	0.93478	(Left-Caudate)(Left-Putamen)(lh.medialorbitofrontal_4)
1750	0.93478	(Left-Caudate)(Left-Putamen)(lh.posteriorcingulate_1)(lh.posteriorcingulate_3)

1751	0.93478	(Left-Caudate)(Left-Putamen)(lh.precentral_4)(lh.precentral_7)
1752	0.93478	(Left-Caudate)(Left-Thalamus-Proper)(Right-Caudate)
1753	0.93478	(Left-Caudate)(lh.caudalanteriorcingulate_2)(lh.rostralanteriorcingulate_1)(lh.rostralanteriorcingulate_2)
1754	0.93478	(Left-Caudate)(lh.caudalmiddlefrontal_1)(lh.caudalmiddlefrontal_4)
1755	0.93478	(Left-Caudate)(lh.medialorbitofrontal_2)(lh.rostralanteriorcingulate_1)(lh.rostralmiddlefrontal_9)
1756	0.93478	(Left-Caudate)(lh.superiorfrontal_10)(lh.superiorfrontal_11)(lh.superiorfrontal_4)
1757	0.93478	(Left-Putamen)(Left-Thalamus-Proper)(lh.precentral_4)(lh.precentral_7)
1758	0.93478	(Left-Putamen)(lh.caudalmiddlefrontal_3)(lh.insula_6)(lh.rostralmiddlefrontal_1)
1759	0.93478	(Left-Putamen)(lh.insula_1)(lh.insula_2)
1760	0.93478	(Left-Putamen)(lh.insula_4)
1761	0.93478	(Left-Putamen)(lh.lateralorbitofrontal_4)(lh.lateralorbitofrontal_6)(lh.parstriangularis_3)
1762	0.93478	(Left-Putamen)(lh.lateralorbitofrontal_7)(lh.medialorbitofrontal_2)(lh.rostralmiddlefrontal_12)
1763	0.93478	(Left-Putamen)(lh.medialorbitofrontal_2)(lh.rostralmiddlefrontal_12)(lh.rostralmiddlefrontal_9)
1764	0.93478	(Left-Putamen)(lh.medialorbitofrontal_4)
1765	0.93478	(Left-Putamen)(lh.parsopercularis_1)(lh.rostralmiddlefrontal_1)
1766	0.93478	(Left-Putamen)(lh.parstriangularis_3)(lh.rostralanteriorcingulate_1)
1767	0.93478	(Left-Putamen)(lh.parstriangularis_3)(lh.rostralmiddlefrontal_12)(lh.rostralmiddlefrontal_9)
1768	0.93478	(Left-Putamen)(lh.posteriorcingulate_1)(lh.posteriorcingulate_3)
1769	0.93478	(Left-Thalamus-Proper)(lh.posteriorcingulate_3)(lh.precentral_4)
1770	0.93478	(Left-Thalamus-Proper)(Right-Caudate)(Right-Thalamus-Proper)
1771	0.93478	(Left-Thalamus-Proper)(Right-Caudate)
1772	0.93478	(lh.insula_1)(lh.insula_2)
1773	0.93478	(lh.lateraloccipital_2)(lh.pericalcarine_1)
1774	0.93478	(lh.lateraloccipital_3)(lh.lingual_1)(lh.pericalcarine_2)
1775	0.93478	(lh.lateraloccipital_6)(lh.pericalcarine_2)
1776	0.93478	(lh.lateralorbitofrontal_4)(lh.lateralorbitofrontal_6)(lh.lateralorbitofrontal_7)
1777	0.93478	(lh.lateralorbitofrontal_6)(lh.parstriangularis_3)(lh.rostralmiddlefrontal_1)(lh.rostralmiddlefrontal_9)
1778	0.93478	(lh.lateralorbitofrontal_6)(lh.rostralmiddlefrontal_1)(lh.rostralmiddlefrontal_9)
1779	0.93478	(lh.paracentral_1)(lh.paracentral_4)(lh.precentral_2)
1780	0.93478	(lh.paracentral_4)(lh.precentral_2)(lh.precentral_3)
1781	0.93478	(lh.parstriangularis_3)(lh.rostralmiddlefrontal_1)(lh.rostralmiddlefrontal_6)
1782	0.93478	(lh.pericalcarine_3)(lh.precuneus_9)
1783	0.93478	(lh.rostralanteriorcingulate_2)(lh.rostralmiddlefrontal_8)
1784	0.93478	(lh.rostralmiddlefrontal_10)(lh.rostralmiddlefrontal_6)
1785	0.93478	(lh.rostralmiddlefrontal_12)(lh.rostralmiddlefrontal_6)(lh.rostralmiddlefrontal_9)
1786	0.93478	(lh.rostralmiddlefrontal_9)(lh.superiorfrontal_3)(lh.superiorfrontal_4)
1787	0.93478	(lh.superiorfrontal_14)(lh.superiorfrontal_7)
1788	0.93478	(lh.superiorparietal_5)(lh.supramarginal_9)
1789	0.93478	(lh.supramarginal_1)(lh.supramarginal_4)
1790	0.93478	(rh.bankssts_1)(rh.middletemporal_2)
1791	0.93478	(rh.caudalanteriorcingulate_2)(rh.caudalanteriorcingulate_3)
1792	0.93478	(rh.caudalmiddlefrontal_2)(rh.caudalmiddlefrontal_3)
1793	0.93478	(rh.fusiform_1)(rh.lateraloccipital_10)(rh.lingual_5)
1794	0.93478	(rh.fusiform_1)(rh.lateraloccipital_8)(rh.lingual_7)
1795	0.93478	(rh.fusiform_4)(rh.lateraloccipital_10)
1796	0.93478	(rh.fusiform_5)(rh.lateraloccipital_10)
1797	0.93478	(rh.inferiorparietal_1)(rh.supramarginal_9)
1798	0.93478	(rh.inferiorparietal_2)(rh.inferiorparietal_4)
1799	0.93478	(rh.inferiortemporal_2)(rh.middletemporal_4)
1800	0.93478	(rh.inferiortemporal_6)(rh.middletemporal_1)

1801	0.93478	(rh.lateraloccipital_3)(rh.lateraloccipital_4)(rh.pericalcarine_3)
1802	0.93478	(rh.lateraloccipital_4)(rh.lateraloccipital_8)(rh.pericalcarine_1)
1803	0.93478	(rh.lateralorbitofrontal_4)(rh.lateralorbitofrontal_6)
1804	0.93478	(rh.lateralorbitofrontal_6)(rh.parstriangularis_4)(rh.rostralmiddlefrontal_12)
1805	0.93478	(rh.lateralorbitofrontal_7)(rh.medialorbitofrontal_2)(rh.rostralmiddlefrontal_13)
1806	0.93478	(rh.parstriangularis_1)(rh.parstriangularis_4)(rh.rostralmiddlefrontal_3)
1807	0.93478	(rh.precentral_11)(rh.precentral_12)(rh.precentral_13)
1808	0.93478	(rh.precuneus_5)(rh.precuneus_8)
1809	0.93478	(rh.rostralmiddlefrontal_11)(rh.superiorfrontal_1)(rh.superiorfrontal_2)
1810	0.93478	(rh.superiorfrontal_13)(rh.superiorfrontal_8)
1811	0.93478	(Right-Caudate)(rh.caudalanteriorcingulate_2)(rh.caudalanteriorcingulate_3)
1812	0.93478	(Right-Caudate)(rh.rostralmiddlefrontal_8)
1813	0.93478	(Right-Caudate)(Right-Putamen)(rh.caudalmiddlefrontal_4)(rh.caudalmiddlefrontal_5)
1814	0.93478	(Right-Caudate)(Right-Putamen)(rh.caudalmiddlefrontal_5)
1815	0.93478	(Right-Caudate)(Right-Putamen)(rh.parstriangularis_1)
1816	0.93478	(Right-Hippocampus)(rh.bankssts_2)(rh.superiortemporal_9)
1817	0.93478	(Right-Hippocampus)(rh.insula_4)(rh.insula_5)
1818	0.93478	(Right-Hippocampus)(rh.precuneus_7)
1819	0.93478	(Right-Hippocampus)(Right-Putamen)(rh.insula_4)(rh.insula_5)
1820	0.93478	(Right-Putamen)(rh.insula_3)(rh.precentral_6)
1821	0.93478	(Right-Putamen)(rh.lateralorbitofrontal_4)(rh.lateralorbitofrontal_6)
1822	0.93237	(Left-Amygdala)(lh.entorhinal_1)
1823	0.93237	(Left-Caudate)(Left-Putamen)(lh.caudalanteriorcingulate_1)(lh.rostralanteriorcingulate_1)
1824	0.93237	(Left-Caudate)(Left-Putamen)(lh.caudalmiddlefrontal_1)(lh.caudalmiddlefrontal_3)
1825	0.93237	(Left-Caudate)(Left-Thalamus-Proper)(lh.posteriorcingulate_3)(lh.precentral_4)
1826	0.93237	(Left-Caudate)(lh.parsopercularis_3)(lh.rostralmiddlefrontal_1)
1827	0.93237	(Left-Hippocampus)(Left-Thalamus-Proper)(lh.transversetemporal_2)
1828	0.93237	(Left-Putamen)(lh.caudalanteriorcingulate_1)(lh.rostralanteriorcingulate_1)
1829	0.93237	(Left-Putamen)(lh.caudalmiddlefrontal_3)(lh.rostralmiddlefrontal_4)
1830	0.93237	(Left-Putamen)(lh.insula_6)(lh.parsopercularis_3)(lh.rostralmiddlefrontal_1)
1831	0.93237	(Left-Putamen)(lh.lateralorbitofrontal_7)(lh.parstriangularis_3)
1832	0.93237	(Left-Putamen)(lh.lateralorbitofrontal_7)(lh.rostralmiddlefrontal_9)
1833	0.93237	(Left-Putamen)(lh.rostralmiddlefrontal_4)(lh.superiorfrontal_4)
1834	0.93237	(lh.caudalanteriorcingulate_2)(lh.posteriorcingulate_1)(lh.posteriorcingulate_2)
1835	0.93237	(lh.caudalanteriorcingulate_2)(lh.superiorfrontal_10)(lh.superiorfrontal_8)
1836	0.93237	(lh.caudalmiddlefrontal_3)(lh.rostralmiddlefrontal_1)(lh.rostralmiddlefrontal_4)
1837	0.93237	(lh.fusiform_1)(lh.fusiform_2)(lh.lateraloccipital_9)(lh.lingual_8)
1838	0.93237	(lh.inferiorparietal_1)(lh.lateraloccipital_6)(lh.pericalcarine_3)
1839	0.93237	(lh.inferiorparietal_9)(lh.supramarginal_10)
1840	0.93237	(lh.insula_6)(lh.parsopercularis_1)(lh.parstriangularis_3)(lh.rostralmiddlefrontal_1)
1841	0.93237	(lh.insula_6)(lh.rostralmiddlefrontal_4)
1842	0.93237	(lh.isthmuscingulate_3)(lh.precuneus_10)(lh.precuneus_6)
1843	0.93237	(lh.lateraloccipital_1)(lh.lateraloccipital_2)(lh.pericalcarine_2)
1844	0.93237	(lh.lateraloccipital_2)(lh.lateraloccipital_3)(lh.pericalcarine_3)
1845	0.93237	(lh.lateralorbitofrontal_6)(lh.parstriangularis_1)(lh.parstriangularis_3)
1846	0.93237	(lh.lateralorbitofrontal_6)(lh.parstriangularis_2)
1847	0.93237	(lh.lateralorbitofrontal_7)(lh.parstriangularis_3)(lh.rostralmiddlefrontal_9)
1848	0.93237	(lh.medialorbitofrontal_4)(lh.medialorbitofrontal_5)
1849	0.93237	(lh.parstriangularis_3)(lh.rostralmiddlefrontal_1)(lh.rostralmiddlefrontal_6)(lh.rostralmiddlefrontal_9)
1850	0.93237	(lh.precentral_13)(lh.precentral_14)

1851	0.93237	(lh.precentral_15)(lh.precentral_16)
1852	0.93237	(lh.precuneus_10)(lh.precuneus_8)(lh.precuneus_9)
1853	0.93237	(lh.precuneus_3)(lh.superiorparietal_5)(lh.superiorparietal_6)
1854	0.93237	(lh.precuneus_6)(lh.superiorparietal_11)(lh.superiorparietal_6)
1855	0.93237	(lh.rostralmiddlefrontal_1)(lh.rostralmiddlefrontal_4)(lh.rostralmiddlefrontal_5)
1856	0.93237	(lh.rostralmiddlefrontal_4)(lh.rostralmiddlefrontal_9)(lh.superiorfrontal_4)
1857	0.93237	(lh.rostralmiddlefrontal_9)(lh.superiorfrontal_1)
1858	0.93237	(lh.superiorfrontal_14)(lh.superiorfrontal_7)(lh.superiorfrontal_8)
1859	0.93237	(lh.superiorparietal_3)(lh.supramarginal_9)
1860	0.93237	(rh.caudalmiddlefrontal_4)(rh.superiorfrontal_9)
1861	0.93237	(rh.fusiform_1)(rh.fusiform_2)(rh.lingual_5)
1862	0.93237	(rh.fusiform_1)(rh.lateraloccipital_7)(rh.lateraloccipital_8)(rh.lingual_2)
1863	0.93237	(rh.fusiform_2)(rh.lateraloccipital_10)(rh.lateraloccipital_8)
1864	0.93237	(rh.inferiorparietal_10)(rh.inferiorparietal_11)(rh.inferiorparietal_12)
1865	0.93237	(rh.inferiorparietal_11)(rh.inferiorparietal_12)(rh.lateraloccipital_8)
1866	0.93237	(rh.inferiortemporal_2)(rh.middletemporal_8)
1867	0.93237	(rh.insula_2)(rh.insula_3)(rh.precentral_7)
1868	0.93237	(rh.insula_3)(rh.insula_7)(rh.parsopercularis_4)
1869	0.93237	(rh.insula_7)(rh.rostralmiddlefrontal_3)
1870	0.93237	(rh.lateralorbitofrontal_7)(rh.superiorfrontal_2)
1871	0.93237	(rh.lingual_5)(rh.lingual_6)
1872	0.93237	(rh.medialorbitofrontal_2)(rh.rostralmiddlefrontal_11)(rh.superiorfrontal_2)
1873	0.93237	(rh.parsopercularis_2)(rh.precentral_1)
1874	0.93237	(rh.postcentral_1)(rh.postcentral_2)
1875	0.93237	(rh.precentral_13)(rh.precentral_8)
1876	0.93237	(rh.precuneus_5)(rh.superiorparietal_10)
1877	0.93237	(rh.rostralmiddlefrontal_1)(rh.superiorfrontal_7)
1878	0.93237	(rh.rostralmiddlefrontal_3)(rh.rostralmiddlefrontal_6)(rh.rostralmiddlefrontal_8)
1879	0.93237	(rh.superiorfrontal_11)(rh.superiorfrontal_13)(rh.superiorfrontal_8)
1880	0.93237	(rh.superiorparietal_1)(rh.supramarginal_1)(rh.supramarginal_8)
1881	0.93237	(rh.supramarginal_6)(rh.supramarginal_9)
1882	0.93237	(Right-Caudate)(lh.rostralanteriorcingulate_1)
1883	0.93237	(Right-Caudate)(rh.insula_1)(rh.precentral_7)
1884	0.93237	(Right-Caudate)(rh.superiorfrontal_11)(rh.superiorfrontal_13)
1885	0.93237	(Right-Caudate)(Right-Putamen)(rh.insula_1)(rh.precentral_7)
1886	0.93237	(Right-Caudate)(Right-Putamen)(rh.rostralmiddlefrontal_8)
1887	0.93237	(Right-Hippocampus)(rh.bankssts_1)
1888	0.93237	(Right-Hippocampus)(rh.lingual_5)
1889	0.93237	(Right-Putamen)(rh.insula_2)(rh.insula_3)(rh.precentral_7)
1890	0.93237	(Right-Putamen)(rh.medialorbitofrontal_2)(rh.rostralmiddlefrontal_12)
1891	0.92995	(Left-Caudate)(Left-Putamen)(lh.lateralorbitofrontal_7)(lh.medialorbitofrontal_2)
1892	0.92995	(Left-Caudate)(Left-Thalamus-Proper)(lh.posteriorcingulate_4)
1893	0.92995	(Left-Caudate)(lh.caudalmiddlefrontal_3)(lh.rostralmiddlefrontal_4)
1894	0.92995	(Left-Caudate)(lh.parstriangularis_3)(lh.rostralmiddlefrontal_4)
1895	0.92995	(Left-Caudate)(lh.precentral_11)(lh.precentral_4)(lh.precentral_7)
1896	0.92995	(Left-Caudate)(lh.precentral_14)
1897	0.92995	(Left-Caudate)(lh.rostralanteriorcingulate_1)(lh.rostralanteriorcingulate_2)(lh.superiorfrontal_4)
1898	0.92995	(Left-Caudate)(lh.superiorfrontal_4)(lh.superiorfrontal_8)
1899	0.92995	(Left-Caudate)(Right-Caudate)(lh.rostralanteriorcingulate_1)
1900	0.92995	(Left-Putamen)(lh.caudalmiddlefrontal_6)(lh.precentral_11)(lh.precentral_9)

1901	0.92995	(Left-Putamen)(lh.lateralorbitofrontal_4)(lh.lateralorbitofrontal_6)(lh.lateralorbitofrontal_7)
1902	0.92995	(Left-Putamen)(lh.medialorbitofrontal_2)(lh.rostralanteriorcingulate_1)(lh.rostralmiddlefrontal_9)
1903	0.92995	(Left-Putamen)(lh.parstriangularis_3)(lh.rostralanteriorcingulate_1)(lh.rostralmiddlefrontal_9)
1904	0.92995	(Left-Putamen)(lh.rostralanteriorcingulate_2)(lh.superiorfrontal_4)
1905	0.92995	(lh.caudalmiddlefrontal_6)(lh.superiorfrontal_12)
1906	0.92995	(lh.fusiform_4)(lh.fusiform_5)(lh.fusiform_6)
1907	0.92995	(lh.inferiortemporal_3)(lh.middletemporal_5)
1908	0.92995	(lh.insula_4)(lh.superiortemporal_11)
1909	0.92995	(lh.lateraloccipital_3)(lh.pericalcarine_1)
1910	0.92995	(lh.lateralorbitofrontal_6)(lh.lateralorbitofrontal_7)(lh.parstriangularis_3)(lh.rostralmiddlefrontal_12)
1911	0.92995	(lh.lateralorbitofrontal_6)(lh.parstriangularis_2)(lh.parstriangularis_3)
1912	0.92995	(lh.lateralorbitofrontal_7)(lh.medialorbitofrontal_2)(lh.rostralmiddlefrontal_9)
1913	0.92995	(lh.lateralorbitofrontal_7)(lh.rostralanteriorcingulate_1)
1914	0.92995	(lh.lateralorbitofrontal_7)(lh.rostralmiddlefrontal_12)(lh.rostralmiddlefrontal_9)
1915	0.92995	(lh.middletemporal_3)(lh.middletemporal_4)
1916	0.92995	(lh.precuneus_10)(lh.superiorparietal_11)
1917	0.92995	(lh.rostralanteriorcingulate_1)(lh.rostralmiddlefrontal_9)(lh.superiorfrontal_4)
1918	0.92995	(lh.rostralmiddlefrontal_12)(lh.superiorfrontal_1)
1919	0.92995	(lh.superiorfrontal_14)(lh.superiorfrontal_7)(lh.superiorfrontal_9)
1920	0.92995	(rh.fusiform_1)(rh.lingual_2)(rh.lingual_4)
1921	0.92995	(rh.fusiform_1)(rh.pericalcarine_1)
1922	0.92995	(rh.fusiform_3)(rh.inferiortemporal_7)(rh.lateraloccipital_10)
1923	0.92995	(rh.fusiform_8)(rh.inferiortemporal_2)
1924	0.92995	(rh.inferiortemporal_1)(rh.insula_5)
1925	0.92995	(rh.inferiortemporal_2)(rh.superiortemporal_6)
1926	0.92995	(rh.insula_3)(rh.precentral_10)
1927	0.92995	(rh.lateralorbitofrontal_1)(rh.lateralorbitofrontal_4)
1928	0.92995	(rh.lateralorbitofrontal_2)(rh.lateralorbitofrontal_7)(rh.rostralmiddlefrontal_12)
1929	0.92995	(rh.lateralorbitofrontal_5)(rh.lateralorbitofrontal_7)(rh.medialorbitofrontal_2)
1930	0.92995	(rh.lingual_2)(rh.pericalcarine_1)
1931	0.92995	(rh.parsopercularis_3)(rh.precentral_1)
1932	0.92995	(rh.parstriangularis_1)(rh.parstriangularis_4)(rh.rostralmiddlefrontal_8)
1933	0.92995	(rh.precuneus_10)(rh.precuneus_7)
1934	0.92995	(rh.precuneus_4)(rh.precuneus_7)(rh.superiorparietal_10)
1935	0.92995	(rh.rostralmiddlefrontal_1)(rh.rostralmiddlefrontal_3)
1936	0.92995	(rh.superiorfrontal_3)(rh.superiorfrontal_6)
1937	0.92995	(rh.superiorparietal_11)(rh.superiorparietal_13)
1938	0.92995	(rh.supramarginal_6)(rh.supramarginal_8)(rh.supramarginal_9)
1939	0.92995	(Right-Caudate)(rh.caudalmiddlefrontal_1)
1940	0.92995	(Right-Caudate)(rh.lateralorbitofrontal_2)
1941	0.92995	(Right-Caudate)(rh.lateralorbitofrontal_7)
1942	0.92995	(Right-Caudate)(rh.medialorbitofrontal_5)
1943	0.92995	(Right-Caudate)(Right-Hippocampus)(rh.supramarginal_9)
1944	0.92995	(Right-Caudate)(Right-Putamen)(rh.lateralorbitofrontal_7)
1945	0.92995	(Right-Hippocampus)(rh.superiortemporal_1)(rh.superiortemporal_3)
1946	0.92995	(Right-Putamen)(rh.insula_3)(rh.insula_7)(rh.parsopercularis_4)
1947	0.92995	(Right-Putamen)(rh.lateralorbitofrontal_2)(rh.parstriangularis_1)(rh.rostralmiddlefrontal_12)
1948	0.92995	(Right-Putamen)(rh.postcentral_1)(rh.supramarginal_9)
1949	0.92995	(Right-Putamen)(rh.posteriorcingulate_4)
1950	0.92995	(Right-Putamen)(rh.precentral_1)

1951	0.92995	(Right-Putamen)(rh.rostralmiddlefrontal_3)(rh.rostralmiddlefrontal_8)
1952	0.92995	(Right-Putamen)(Right-Thalamus-Proper)(rh.insula_2)(rh.supramarginal_9)
1953	0.92995	(Right-Thalamus-Proper)(rh.insula_2)(rh.supramarginal_9)
1954	0.92754	(Left-Caudate)(Left-Putamen)(lh.caudalanteriorcingulate_2)(lh.rostralanteriorcingulate_1)
1955	0.92754	(Left-Caudate)(Left-Putamen)(lh.caudalmiddlefrontal_3)(lh.caudalmiddlefrontal_5)
1956	0.92754	(Left-Caudate)(Left-Putamen)(lh.caudalmiddlefrontal_4)(lh.caudalmiddlefrontal_6)(lh.precentral_11)
1957	0.92754	(Left-Caudate)(Left-Putamen)(lh.caudalmiddlefrontal_6)(lh.precentral_9)
1958	0.92754	(Left-Caudate)(Left-Putamen)(lh.parstriangularis_3)(lh.rostralmiddlefrontal_1)
1959	0.92754	(Left-Caudate)(Left-Putamen)(lh.parstriangularis_3)(lh.rostralmiddlefrontal_9)
1960	0.92754	(Left-Caudate)(Left-Putamen)(lh.rostralmiddlefrontal_12)
1961	0.92754	(Left-Caudate)(lh.caudalanteriorcingulate_2)(lh.posteriorcingulate_1)(lh.posteriorcingulate_2)
1962	0.92754	(Left-Caudate)(lh.caudalmiddlefrontal_6)(lh.superiorfrontal_12)
1963	0.92754	(Left-Caudate)(lh.medialorbitofrontal_4)(lh.medialorbitofrontal_5)
1964	0.92754	(Left-Caudate)(lh.parstriangularis_3)(lh.rostralanteriorcingulate_1)
1965	0.92754	(Left-Putamen)(lh.caudalanteriorcingulate_2)(lh.rostralanteriorcingulate_1)
1966	0.92754	(Left-Putamen)(lh.caudalmiddlefrontal_3)(lh.caudalmiddlefrontal_4)(lh.caudalmiddlefrontal_5)
1967	0.92754	(Left-Putamen)(lh.insula_6)(lh.parstriangularis_3)(lh.rostralmiddlefrontal_1)
1968	0.92754	(Left-Putamen)(lh.insula_6)(lh.rostralmiddlefrontal_4)
1969	0.92754	(Left-Putamen)(lh.lateralorbitofrontal_4)(lh.parstriangularis_3)(lh.rostralmiddlefrontal_9)
1970	0.92754	(Left-Putamen)(lh.lateralorbitofrontal_6)(lh.lateralorbitofrontal_7)(lh.rostralmiddlefrontal_12)
1971	0.92754	(Left-Putamen)(lh.parsopercularis_3)(lh.parstriangularis_3)
1972	0.92754	(Left-Putamen)(lh.postcentral_3)
1973	0.92754	(Left-Putamen)(lh.rostralanteriorcingulate_1)(lh.superiorfrontal_4)
1974	0.92754	(Left-Putamen)(lh.rostralanteriorcingulate_2)(lh.rostralmiddlefrontal_4)
1975	0.92754	(Left-Thalamus-Proper)(lh.isthmuscingulate_2)(lh.isthmuscingulate_3)
1976	0.92754	(lh.bankssts_3)(lh.fusiform_5)
1977	0.92754	(lh.caudalanteriorcingulate_2)(lh.superiorfrontal_10)(lh.superiorfrontal_11)
1978	0.92754	(lh.fusiform_1)(lh.fusiform_2)(lh.fusiform_3)
1979	0.92754	(lh.fusiform_2)(lh.lateraloccipital_7)(lh.pericalcarine_3)
1980	0.92754	(lh.fusiform_5)(lh.fusiform_7)
1981	0.92754	(lh.inferiortemporal_3)(lh.middletemporal_3)
1982	0.92754	(lh.insula_1)(lh.supramarginal_4)
1983	0.92754	(lh.lateraloccipital_1)(lh.lateraloccipital_2)(lh.lateraloccipital_3)
1984	0.92754	(lh.lateralorbitofrontal_4)(lh.parstriangularis_3)(lh.rostralmiddlefrontal_12)
1985	0.92754	(lh.lateralorbitofrontal_4)(lh.rostralmiddlefrontal_12)(lh.rostralmiddlefrontal_9)
1986	0.92754	(lh.lateralorbitofrontal_6)(lh.lateralorbitofrontal_7)(lh.rostralmiddlefrontal_9)
1987	0.92754	(lh.lateralorbitofrontal_6)(lh.rostralmiddlefrontal_12)(lh.rostralmiddlefrontal_6)
1988	0.92754	(lh.medialorbitofrontal_2)(lh.rostralanteriorcingulate_1)(lh.rostralmiddlefrontal_12)(lh.rostralmiddlefrontal_9)
1989	0.92754	(lh.parsopercularis_3)(lh.parstriangularis_3)(lh.rostralmiddlefrontal_1)
1990	0.92754	(lh.parsorbitalis_1)(lh.parstriangularis_1)
1991	0.92754	(lh.pericalcarine_1)(lh.pericalcarine_3)
1992	0.92754	(lh.postcentral_14)(lh.supramarginal_1)(lh.supramarginal_2)
1993	0.92754	(lh.rostralanteriorcingulate_1)(lh.rostralmiddlefrontal_12)(lh.superiorfrontal_3)
1994	0.92754	(lh.rostralanteriorcingulate_2)(lh.rostralmiddlefrontal_4)(lh.superiorfrontal_4)
1995	0.92754	(lh.rostralmiddlefrontal_8)(lh.rostralmiddlefrontal_9)(lh.superiorfrontal_3)
1996	0.92754	(lh.rostralmiddlefrontal_9)(lh.superiorfrontal_1)(lh.superiorfrontal_3)
1997	0.92754	(rh.bankssts_2)(rh.superiortemporal_3)(rh.superiortemporal_9)
1998	0.92754	(rh.fusiform_1)(rh.fusiform_2)(rh.lingual_7)
1999	0.92754	(rh.fusiform_2)(rh.inferiorparietal_10)
2000	0.92754	(rh.fusiform_2)(rh.lingual_5)(rh.lingual_7)

2001	0.92754	(rh.fusiform_4)(rh.fusiform_6)
2002	0.92754	(rh.inferiortemporal_4)(rh.middletemporal_7)
2003	0.92754	(rh.lateraloccipital_10)(rh.middletemporal_1)
2004	0.92754	(rh.lateraloccipital_7)(rh.lingual_2)(rh.pericalcarine_3)
2005	0.92754	(rh.lateralorbitofrontal_2)(rh.lateralorbitofrontal_4)
2006	0.92754	(rh.lateralorbitofrontal_2)(rh.lateralorbitofrontal_6)(rh.parstriangularis_1)(rh.rostralmiddlefrontal_12)
2007	0.92754	(rh.lateralorbitofrontal_6)(rh.lateralorbitofrontal_7)(rh.rostralmiddlefrontal_13)
2008	0.92754	(rh.lateralorbitofrontal_7)(rh.parstriangularis_1)
2009	0.92754	(rh.middletemporal_5)(rh.middletemporal_7)
2010	0.92754	(rh.precentral_8)(rh.precentral_9)
2011	0.92754	(rh.superiorfrontal_10)(rh.superiorfrontal_13)
2012	0.92754	(rh.superiorfrontal_3)(rh.superiorfrontal_4)(rh.superiorfrontal_5)
2013	0.92754	(rh.supramarginal_6)(rh.supramarginal_7)(rh.supramarginal_8)
2014	0.92754	(Right-Caudate)(rh.isthmuscingulate_1)
2015	0.92754	(Right-Caudate)(rh.superiorfrontal_5)
2016	0.92754	(Right-Caudate)(Right-Hippocampus)(Right-Putamen)(rh.supramarginal_9)
2017	0.92754	(Right-Caudate)(Right-Putamen)(rh.lateralorbitofrontal_2)
2018	0.92754	(Right-Caudate)(Right-Putamen)(rh.posteriorcingulate_4)
2019	0.92754	(Right-Hippocampus)(rh.inferiortemporal_2)
2020	0.92754	(Right-Hippocampus)(rh.isthmuscingulate_2)(rh.precuneus_7)
2021	0.92754	(Right-Hippocampus)(rh.lingual_5)(rh.lingual_7)
2022	0.92512	(Left-Accumbens-area)(lh.medialorbitofrontal_5)
2023	0.92512	(Left-Accumbens-area)(lh.rostralanteriorcingulate_1)
2024	0.92512	(Left-Caudate)(Left-Putamen)(lh.caudalanteriorcingulate_2)(lh.posteriorcingulate_3)
2025	0.92512	(Left-Caudate)(Left-Putamen)(lh.insula_6)(lh.parstriangularis_3)
2026	0.92512	(Left-Caudate)(Left-Putamen)(lh.precentral_14)
2027	0.92512	(Left-Caudate)(lh.caudalanteriorcingulate_1)(lh.caudalanteriorcingulate_2)(lh.rostralanteriorcingulate_1)
2028	0.92512	(Left-Caudate)(lh.caudalmiddlefrontal_3)(lh.insula_6)(lh.rostralmiddlefrontal_1)
2029	0.92512	(Left-Caudate)(lh.medialorbitofrontal_2)(lh.rostralmiddlefrontal_12)
2030	0.92512	(Left-Caudate)(lh.rostralanteriorcingulate_1)(lh.rostralmiddlefrontal_12)
2031	0.92512	(Left-Caudate)(lh.rostralmiddlefrontal_12)(lh.rostralmiddlefrontal_9)
2032	0.92512	(Left-Caudate)(lh.rostralmiddlefrontal_4)(lh.superiorfrontal_10)
2033	0.92512	(Left-Hippocampus)(lh.inferiortemporal_1)
2034	0.92512	(Left-Putamen)(lh.caudalanteriorcingulate_2)(lh.posteriorcingulate_3)
2035	0.92512	(Left-Putamen)(lh.caudalmiddlefrontal_1)(lh.caudalmiddlefrontal_4)
2036	0.92512	(Left-Putamen)(lh.lateralorbitofrontal_4)(lh.lateralorbitofrontal_6)(lh.rostralmiddlefrontal_12)
2037	0.92512	(Left-Putamen)(lh.lateralorbitofrontal_4)(lh.rostralmiddlefrontal_12)(lh.rostralmiddlefrontal_9)
2038	0.92512	(Left-Putamen)(lh.lateralorbitofrontal_6)(lh.parstriangularis_3)(lh.rostralmiddlefrontal_12)
2039	0.92512	(Left-Putamen)(lh.parstriangularis_3)(lh.rostralmiddlefrontal_4)(lh.rostralmiddlefrontal_9)
2040	0.92512	(Left-Putamen)(lh.superiorfrontal_14)
2041	0.92512	(Left-Thalamus-Proper)(lh.postcentral_8)
2042	0.92512	(lh.bankssts_2)(lh.superiortemporal_6)
2043	0.92512	(lh.caudalanteriorcingulate_1)(lh.caudalanteriorcingulate_2)(lh.rostralanteriorcingulate_1)(lh.rostralanteriorcingulate_2)
2044	0.92512	(lh.caudalanteriorcingulate_1)(lh.caudalanteriorcingulate_2)(lh.rostralanteriorcingulate_1)
2045	0.92512	(lh.insula_1)(lh.precentral_7)
2046	0.92512	(lh.insula_4)(lh.temporalpole_1)
2047	0.92512	(lh.isthmuscingulate_2)(lh.precuneus_11)
2048	0.92512	(lh.lateralorbitofrontal_1)(lh.lateralorbitofrontal_3)
2049	0.92512	(lh.lateralorbitofrontal_4)(lh.lateralorbitofrontal_7)(lh.parstriangularis_3)
2050	0.92512	(lh.lingual_4)(lh.lingual_8)(lh.pericalcarine_3)

2051	0.92512	(lh.parstriangularis_3)(lh.rostralanteriorcingulate_1)(lh.rostralmiddlefrontal_12)
2052	0.92512	(lh.pericalcarine_1)(lh.superiorparietal_14)
2053	0.92512	(lh.precentral_4)(lh.superiorfrontal_14)
2054	0.92512	(lh.precuneus_11)(lh.superiorparietal_11)
2055	0.92512	(lh.rostralanteriorcingulate_2)(lh.rostralmiddlefrontal_9)
2056	0.92512	(lh.rostralmiddlefrontal_1)(lh.rostralmiddlefrontal_2)
2057	0.92512	(lh.rostralmiddlefrontal_12)(lh.superiorfrontal_1)(lh.superiorfrontal_3)
2058	0.92512	(lh.rostralmiddlefrontal_4)(lh.rostralmiddlefrontal_8)(lh.superiorfrontal_4)
2059	0.92512	(lh.superiorfrontal_13)(lh.superiorfrontal_8)(lh.superiorfrontal_9)
2060	0.92512	(rh.bankssts_2)(rh.inferiorparietal_10)
2061	0.92512	(rh.caudalmiddlefrontal_3)(rh.caudalmiddlefrontal_5)
2062	0.92512	(rh.caudalmiddlefrontal_4)(rh.parsopercularis_4)(rh.rostralmiddlefrontal_3)
2063	0.92512	(rh.fusiform_1)(rh.fusiform_2)(rh.lateraloccipital_8)
2064	0.92512	(rh.fusiform_1)(rh.lateraloccipital_4)(rh.lateraloccipital_7)
2065	0.92512	(rh.fusiform_3)(rh.fusiform_4)(rh.lateraloccipital_10)
2066	0.92512	(rh.fusiform_6)(rh.fusiform_7)
2067	0.92512	(rh.inferiorparietal_1)(rh.superiorparietal_1)(rh.superiorparietal_7)
2068	0.92512	(rh.insula_1)(rh.postcentral_1)
2069	0.92512	(rh.isthmuscingulate_2)(rh.precuneus_3)(rh.precuneus_4)
2070	0.92512	(rh.lateraloccipital_1)(rh.lateraloccipital_3)(rh.lateraloccipital_4)
2071	0.92512	(rh.lateraloccipital_1)(rh.pericalcarine_4)
2072	0.92512	(rh.lateralorbitofrontal_4)(rh.lateralorbitofrontal_7)
2073	0.92512	(rh.lateralorbitofrontal_6)(rh.lateralorbitofrontal_7)(rh.parstriangularis_1)
2074	0.92512	(rh.lateralorbitofrontal_6)(rh.parsorbitalis_2)
2075	0.92512	(rh.lateralorbitofrontal_7)(rh.medialorbitofrontal_2)(rh.rostralmiddlefrontal_12)
2076	0.92512	(rh.middletemporal_6)(rh.middletemporal_7)
2077	0.92512	(rh.parsorbitalis_2)(rh.rostralmiddlefrontal_12)
2078	0.92512	(rh.pericalcarine_2)(rh.superiorparietal_13)
2079	0.92512	(rh.precuneus_1)(rh.superiorparietal_11)(rh.superiorparietal_12)
2080	0.92512	(rh.precuneus_4)(rh.precuneus_6)
2081	0.92512	(rh.rostralmiddlefrontal_11)(rh.rostralmiddlefrontal_8)
2082	0.92512	(rh.rostralmiddlefrontal_3)(rh.rostralmiddlefrontal_5)(rh.rostralmiddlefrontal_6)
2083	0.92512	(rh.rostralmiddlefrontal_5)(rh.rostralmiddlefrontal_6)(rh.rostralmiddlefrontal_8)
2084	0.92512	(rh.rostralmiddlefrontal_9)(rh.superiorfrontal_5)
2085	0.92512	(rh.superiorfrontal_6)(rh.superiorfrontal_9)
2086	0.92512	(Right-Caudate)(rh.lateralorbitofrontal_5)
2087	0.92512	(Right-Caudate)(rh.lateralorbitofrontal_6)
2088	0.92512	(Right-Caudate)(Right-Putamen)(rh.lateralorbitofrontal_6)
2089	0.92512	(Right-Caudate)(Right-Putamen)(rh.superiorfrontal_2)
2090	0.92512	(Right-Caudate)(Right-Thalamus-Proper)(rh.posteriorcingulate_3)
2091	0.92512	(Right-Hippocampus)(rh.fusiform_7)
2092	0.92512	(Right-Putamen)(rh.lateralorbitofrontal_2)(rh.lateralorbitofrontal_4)
2093	0.92512	(Right-Putamen)(rh.parstriangularis_1)(rh.rostralmiddlefrontal_3)
2094	0.92512	(Right-Putamen)(rh.rostralmiddlefrontal_12)(rh.superiorfrontal_2)
2095	0.92512	(Right-Putamen)(rh.superiorparietal_1)(rh.supramarginal_1)
2096	0.92512	(Right-Thalamus-Proper)(rh.posteriorcingulate_3)
2097	0.92271	(Left-Amygdala)(Left-Hippocampus)(lh.entorhinal_1)
2098	0.92271	(Left-Amygdala)(Left-Putamen)(lh.insula_4)
2099	0.92271	(Left-Caudate)(Left-Putamen)(lh.caudalmiddlefrontal_3)(lh.rostralmiddlefrontal_4)
2100	0.92271	(Left-Caudate)(Left-Putamen)(lh.caudalmiddlefrontal_4)(lh.caudalmiddlefrontal_5)(lh.caudalmiddlefrontal_6)

2101	0.92271	(Left-Caudate)(Left-Thalamus-Proper)(lh.paracentral_5)
2102	0.92271	(Left-Caudate)(Left-Thalamus-Proper)(lh.precentral_11)(lh.precentral_7)
2103	0.92271	(Left-Caudate)(lh.caudalanteriorcingulate_1)(lh.caudalanteriorcingulate_2)(lh.rostralanteriorcingulate_1)(lh.rostralanteriorcingulate_2)
2104	0.92271	(Left-Caudate)(lh.caudalanteriorcingulate_2)(lh.superiorfrontal_11)(lh.superiorfrontal_8)
2105	0.92271	(Left-Caudate)(lh.isthmuscingulate_1)
2106	0.92271	(Left-Caudate)(lh.lateralorbitofrontal_6)
2107	0.92271	(Left-Caudate)(lh.lateralorbitofrontal_7)(lh.medialorbitofrontal_4)
2108	0.92271	(Left-Caudate)(lh.paracentral_5)(lh.superiorfrontal_12)
2109	0.92271	(Left-Caudate)(lh.rostralmiddlefrontal_4)(lh.superiorfrontal_11)
2110	0.92271	(Left-Caudate)(lh.superiorfrontal_10)(lh.superiorfrontal_8)
2111	0.92271	(Left-Caudate)(lh.superiorfrontal_11)(lh.superiorfrontal_12)(lh.superiorfrontal_8)
2112	0.92271	(Left-Caudate)(lh.superiorfrontal_12)(lh.superiorfrontal_14)(lh.superiorfrontal_8)
2113	0.92271	(Left-Putamen)(Left-Thalamus-Proper)(lh.postcentral_8)
2114	0.92271	(Left-Putamen)(lh.insula_1)(lh.insula_2)(lh.precentral_11)
2115	0.92271	(Left-Putamen)(lh.insula_1)(lh.precentral_7)
2116	0.92271	(Left-Putamen)(lh.lateralorbitofrontal_7)(lh.rostralanteriorcingulate_1)
2117	0.92271	(Left-Putamen)(lh.lateralorbitofrontal_7)(lh.rostralmiddlefrontal_12)(lh.rostralmiddlefrontal_9)
2118	0.92271	(Left-Putamen)(lh.medialorbitofrontal_2)(lh.rostralanteriorcingulate_1)(lh.rostralmiddlefrontal_12)
2119	0.92271	(Left-Putamen)(lh.parsopercularis_1)(lh.parstriangularis_3)(lh.rostralmiddlefrontal_1)
2120	0.92271	(Left-Putamen)(lh.parstriangularis_3)(lh.rostralmiddlefrontal_1)(lh.rostralmiddlefrontal_4)
2121	0.92271	(Left-Putamen)(lh.posteriorcingulate_3)(lh.precentral_4)
2122	0.92271	(lh.caudalanteriorcingulate_2)(lh.posteriorcingulate_3)(lh.superiorfrontal_14)
2123	0.92271	(lh.caudalanteriorcingulate_2)(lh.superiorfrontal_11)(lh.superiorfrontal_12)(lh.superiorfrontal_14)(lh.superiorfrontal_8)
2124	0.92271	(lh.caudalmiddlefrontal_1)(lh.caudalmiddlefrontal_2)
2125	0.92271	(lh.caudalmiddlefrontal_1)(lh.rostralmiddlefrontal_3)
2126	0.92271	(lh.caudalmiddlefrontal_3)(lh.superiorfrontal_12)
2127	0.92271	(lh.fusiform_1)(lh.fusiform_3)(lh.fusiform_5)
2128	0.92271	(lh.fusiform_2)(lh.lateraloccipital_7)(lh.lateraloccipital_9)
2129	0.92271	(lh.inferiorparietal_9)(lh.supramarginal_9)
2130	0.92271	(lh.inferiortemporal_6)(lh.inferiortemporal_8)
2131	0.92271	(lh.insula_1)(lh.insula_2)(lh.precentral_11)
2132	0.92271	(lh.isthmuscingulate_2)(lh.isthmuscingulate_3)(lh.precuneus_11)
2133	0.92271	(lh.lateraloccipital_1)(lh.lateraloccipital_3)(lh.pericalcarine_2)
2134	0.92271	(lh.lateralorbitofrontal_4)(lh.lateralorbitofrontal_6)(lh.parstriangularis_3)(lh.rostralmiddlefrontal_12)
2135	0.92271	(lh.lateralorbitofrontal_6)(lh.rostralmiddlefrontal_11)
2136	0.92271	(lh.lateralorbitofrontal_7)(lh.medialorbitofrontal_1)
2137	0.92271	(lh.medialorbitofrontal_2)(lh.superiorfrontal_3)(lh.superiorfrontal_4)
2138	0.92271	(lh.medialorbitofrontal_2)(lh.superiorfrontal_4)
2139	0.92271	(lh.paracentral_5)(lh.superiorfrontal_12)(lh.superiorfrontal_14)
2140	0.92271	(lh.precuneus_10)(lh.precuneus_11)(lh.precuneus_6)
2141	0.92271	(lh.precuneus_11)(lh.precuneus_6)(lh.precuneus_9)
2142	0.92271	(lh.rostralanteriorcingulate_2)(lh.superiorfrontal_8)
2143	0.92271	(lh.superiorfrontal_10)(lh.superiorfrontal_3)(lh.superiorfrontal_4)
2144	0.92271	(lh.superiorfrontal_10)(lh.superiorfrontal_3)
2145	0.92271	(lh.superiorfrontal_10)(lh.superiorfrontal_7)(lh.superiorfrontal_9)
2146	0.92271	(lh.superiorfrontal_10)(lh.superiorfrontal_9)
2147	0.92271	(lh.superiorfrontal_11)(lh.superiorfrontal_13)
2148	0.92271	(lh.superiorfrontal_14)(lh.superiorfrontal_4)(lh.superiorfrontal_8)
2149	0.92271	(lh.superiorfrontal_14)(lh.superiorfrontal_4)
2150	0.92271	(lh.superiorparietal_11)(lh.superiorparietal_5)(lh.superiorparietal_6)

2151	0.92271	(rh.bankssts_3)(rh.superiortemporal_3)
2152	0.92271	(rh.fusiform_1)(rh.inferiorparietal_10)
2153	0.92271	(rh.inferiorparietal_12)(rh.lateraloccipital_4)(rh.lateraloccipital_8)
2154	0.92271	(rh.inferiortemporal_2)(rh.middletemporal_9)
2155	0.92271	(rh.inferiortemporal_4)(rh.inferiortemporal_5)(rh.middletemporal_6)
2156	0.92271	(rh.insula_3)(rh.precentral_5)
2157	0.92271	(rh.insula_7)(rh.lateralorbitofrontal_2)
2158	0.92271	(rh.lateralorbitofrontal_2)(rh.lateralorbitofrontal_6)(rh.lateralorbitofrontal_7)(rh.rostralmiddlefrontal_12)
2159	0.92271	(rh.lateralorbitofrontal_6)(rh.parstriangularis_1)(rh.parstriangularis_4)(rh.rostralmiddlefrontal_12)
2160	0.92271	(rh.lingual_4)(rh.lingual_5)
2161	0.92271	(rh.lingual_4)(rh.pericalcarine_1)
2162	0.92271	(rh.paracentral_6)(rh.precentral_13)
2163	0.92271	(rh.parsopercularis_4)(rh.parstriangularis_1)
2164	0.92271	(rh.parstriangularis_4)(rh.rostralmiddlefrontal_12)(rh.rostralmiddlefrontal_8)
2165	0.92271	(rh.posteriorcingulate_2)(rh.superiorfrontal_11)
2166	0.92271	(rh.precuneus_10)(rh.precuneus_8)
2167	0.92271	(rh.precuneus_4)(rh.superiorparietal_10)(rh.superiorparietal_11)
2168	0.92271	(rh.rostralmiddlefrontal_12)(rh.rostralmiddlefrontal_3)
2169	0.92271	(rh.rostralmiddlefrontal_7)(rh.rostralmiddlefrontal_8)
2170	0.92271	(rh.superiorfrontal_11)(rh.superiorfrontal_7)(rh.superiorfrontal_8)(rh.superiorfrontal_9)
2171	0.92271	(rh.superiorfrontal_11)(rh.superiorfrontal_7)(rh.superiorfrontal_8)
2172	0.92271	(rh.superiorfrontal_11)(rh.superiorfrontal_7)(rh.superiorfrontal_9)
2173	0.92271	(rh.superiorfrontal_11)(rh.superiorfrontal_7)
2174	0.92271	(rh.superiorfrontal_13)(rh.superiorfrontal_7)
2175	0.92271	(rh.superiorfrontal_13)(rh.superiorfrontal_8)(rh.superiorfrontal_9)
2176	0.92271	(rh.superiorparietal_1)(rh.superiorparietal_10)
2177	0.92271	(Right-Amygdala)(rh.inferiortemporal_1)(rh.insula_5)
2178	0.92271	(Right-Caudate)(rh.caudalmiddlefrontal_4)(rh.superiorfrontal_7)
2179	0.92271	(Right-Caudate)(rh.lateralorbitofrontal_7)(rh.medialorbitofrontal_2)
2180	0.92271	(Right-Caudate)(rh.rostralmiddlefrontal_5)
2181	0.92271	(Right-Hippocampus)(rh.bankssts_2)(rh.superiortemporal_1)
2182	0.92271	(Right-Putamen)(rh.insula_3)(rh.precentral_10)
2183	0.92271	(Right-Putamen)(rh.insula_3)(rh.precentral_5)
2184	0.92271	(Right-Putamen)(rh.insula_7)(rh.lateralorbitofrontal_2)
2185	0.92271	(Right-Putamen)(rh.lateralorbitofrontal_2)(rh.lateralorbitofrontal_7)(rh.rostralmiddlefrontal_12)
2186	0.92271	(Right-Putamen)(rh.supramarginal_8)(rh.supramarginal_9)
2187	0.92271	(Right-Putamen)(rh.supramarginal_8)
2188	0.92271	(Right-Thalamus-Proper)(rh.precentral_11)
2189	0.92029	(Left-Caudate)(Left-Pallidum)(Left-Putamen)(Left-Thalamus-Proper)(lh.precentral_11)
2190	0.92029	(Left-Caudate)(Left-Pallidum)(Left-Thalamus-Proper)(lh.precentral_11)
2191	0.92029	(Left-Caudate)(Left-Putamen)(Left-Thalamus-Proper)(lh.precentral_11)(lh.precentral_7)
2192	0.92029	(Left-Caudate)(Left-Putamen)(lh.rostralanteriorcingulate_1)(lh.superiorfrontal_4)
2193	0.92029	(Left-Caudate)(Left-Putamen)(lh.rostralmiddlefrontal_12)(lh.rostralmiddlefrontal_9)
2194	0.92029	(Left-Caudate)(lh.caudalmiddlefrontal_1)(lh.rostralmiddlefrontal_4)
2195	0.92029	(Left-Caudate)(lh.lateralorbitofrontal_7)(lh.parstriangularis_3)
2196	0.92029	(Left-Caudate)(lh.lateralorbitofrontal_7)(lh.rostralmiddlefrontal_9)
2197	0.92029	(Left-Caudate)(lh.superiorfrontal_11)(lh.superiorfrontal_4)(lh.superiorfrontal_8)
2198	0.92029	(Left-Caudate)(lh.supramarginal_1)
2199	0.92029	(Left-Caudate)(rh.caudalanteriorcingulate_1)
2200	0.92029	(Left-Hippocampus)(lh.inferiorparietal_5)(lh.superiortemporal_2)

2201	0.92029	(Left-Pallidum)(Left-Putamen)(Left-Thalamus-Proper)(lh.precentral_11)
2202	0.92029	(Left-Pallidum)(Left-Thalamus-Proper)(lh.precentral_11)
2203	0.92029	(Left-Pallidum)(lh.precentral_4)
2204	0.92029	(Left-Putamen)(lh.caudalanteriorcingulate_1)(lh.rostralanteriorcingulate_1)(lh.rostralanteriorcingulate_2)
2205	0.92029	(Left-Putamen)(lh.lateralorbitofrontal_1)(lh.lateralorbitofrontal_3)
2206	0.92029	(Left-Putamen)(lh.lateralorbitofrontal_6)(lh.parstriangularis_3)(lh.rostralmiddlefrontal_12)(lh.rostralmiddlefrontal_9)
2207	0.92029	(Left-Putamen)(lh.postcentral_8)(lh.precentral_4)
2208	0.92029	(Left-Putamen)(lh.superiorfrontal_11)(lh.superiorfrontal_4)
2209	0.92029	(lh.caudalanteriorcingulate_2)(lh.superiorfrontal_10)(lh.superiorfrontal_11)(lh.superiorfrontal_8)
2210	0.92029	(lh.caudalanteriorcingulate_2)(lh.superiorfrontal_4)
2211	0.92029	(lh.caudalmiddlefrontal_1)(lh.caudalmiddlefrontal_4)(lh.caudalmiddlefrontal_5)
2212	0.92029	(lh.caudalmiddlefrontal_3)(lh.rostralmiddlefrontal_3)(lh.rostralmiddlefrontal_4)
2213	0.92029	(lh.cuneus_1)(lh.pericalcarine_1)(lh.superiorparietal_12)
2214	0.92029	(lh.insula_6)(lh.parsopercularis_1)(lh.parsopercularis_3)(lh.rostralmiddlefrontal_1)
2215	0.92029	(lh.isthmuscingulate_2)(lh.superiorparietal_5)
2216	0.92029	(lh.lateraloccipital_2)(lh.lateraloccipital_3)(lh.pericalcarine_2)(lh.pericalcarine_3)
2217	0.92029	(lh.lateraloccipital_2)(lh.lateraloccipital_6)(lh.pericalcarine_3)
2218	0.92029	(lh.lateraloccipital_6)(lh.lingual_1)(lh.pericalcarine_2)
2219	0.92029	(lh.lateraloccipital_7)(lh.lingual_8)
2220	0.92029	(lh.lateralorbitofrontal_2)(lh.medialorbitofrontal_2)
2221	0.92029	(lh.lateralorbitofrontal_6)(lh.lateralorbitofrontal_7)(lh.parstriangularis_3)(lh.rostralmiddlefrontal_9)
2222	0.92029	(lh.lateralorbitofrontal_7)(lh.medialorbitofrontal_2)(lh.rostralanteriorcingulate_1)
2223	0.92029	(lh.lateralorbitofrontal_7)(lh.medialorbitofrontal_2)(lh.rostralmiddlefrontal_12)(lh.rostralmiddlefrontal_9)
2224	0.92029	(lh.lingual_1)(lh.lingual_3)(lh.pericalcarine_3)
2225	0.92029	(lh.lingual_6)(lh.lingual_8)(lh.pericalcarine_3)
2226	0.92029	(lh.parsopercularis_2)(lh.rostralmiddlefrontal_1)
2227	0.92029	(lh.parstriangularis_3)(lh.rostralanteriorcingulate_1)(lh.rostralmiddlefrontal_12)(lh.rostralmiddlefrontal_9)
2228	0.92029	(lh.parstriangularis_3)(lh.rostralmiddlefrontal_12)(lh.rostralmiddlefrontal_6)
2229	0.92029	(lh.postcentral_8)(lh.precentral_11)
2230	0.92029	(lh.precuneus_10)(lh.precuneus_6)(lh.precuneus_8)
2231	0.92029	(lh.precuneus_10)(lh.precuneus_9)(lh.superiorparietal_11)
2232	0.92029	(lh.precuneus_6)(lh.precuneus_8)(lh.superiorparietal_11)
2233	0.92029	(lh.precuneus_7)(lh.precuneus_8)
2234	0.92029	(lh.rostralmiddlefrontal_4)(lh.superiorfrontal_11)(lh.superiorfrontal_4)
2235	0.92029	(lh.superiorfrontal_1)(lh.superiorfrontal_2)(lh.superiorfrontal_3)
2236	0.92029	(lh.superiorfrontal_1)(lh.superiorfrontal_2)
2237	0.92029	(lh.superiorfrontal_6)(lh.superiorfrontal_8)
2238	0.92029	(rh.caudalmiddlefrontal_5)(rh.superiorfrontal_7)
2239	0.92029	(rh.fusiform_3)(rh.fusiform_5)
2240	0.92029	(rh.inferiorparietal_10)(rh.inferiorparietal_12)(rh.lateraloccipital_8)
2241	0.92029	(rh.inferiorparietal_10)(rh.insula_2)
2242	0.92029	(rh.insula_1)(rh.supramarginal_1)(rh.supramarginal_8)
2243	0.92029	(rh.lateraloccipital_8)(rh.lingual_5)(rh.lingual_7)
2244	0.92029	(rh.lateralorbitofrontal_2)(rh.rostralmiddlefrontal_8)
2245	0.92029	(rh.middletemporal_9)(rh.superiortemporal_10)
2246	0.92029	(rh.pericalcarine_2)(rh.precuneus_1)
2247	0.92029	(rh.precentral_13)(rh.superiorfrontal_17)
2248	0.92029	(rh.superiorfrontal_10)(rh.superiorfrontal_11)(rh.superiorfrontal_13)
2249	0.92029	(rh.superiorfrontal_5)(rh.superiorfrontal_6)(rh.superiorfrontal_9)
2250	0.92029	(rh.superiorfrontal_6)(rh.superiorfrontal_7)(rh.superiorfrontal_9)

2251	0.92029	(rh.superiorfrontal_6)(rh.superiorfrontal_8)(rh.superiorfrontal_9)
2252	0.92029	(rh.superiorparietal_1)(rh.superiorparietal_6)
2253	0.92029	(Right-Caudate)(Right-Putamen)(rh.lateralorbitofrontal_7)(rh.medialorbitofrontal_2)
2254	0.92029	(Right-Hippocampus)(rh.parahippocampal_1)
2255	0.92029	(Right-Hippocampus)(rh.superiortemporal_10)
2256	0.92029	(Right-Hippocampus)(rh.superiortemporal_6)
2257	0.92029	(Right-Putamen)(rh.lateralorbitofrontal_2)(rh.rostralmiddlefrontal_8)
2258	0.92029	(Right-Putamen)(rh.lateralorbitofrontal_4)(rh.lateralorbitofrontal_7)
2259	0.92029	(Right-Putamen)(rh.lateralorbitofrontal_5)(rh.medialorbitofrontal_2)
2260	0.92029	(Right-Putamen)(rh.lateralorbitofrontal_7)(rh.medialorbitofrontal_2)(rh.rostralmiddlefrontal_12)
2261	0.92029	(Right-Putamen)(rh.rostralmiddlefrontal_13)
2262	0.91787	(Left-Accumbens-area)(Left-Caudate)(lh.rostralanteriorcingulate_1)
2263	0.91787	(Left-Amygdala)(lh.insula_4)(lh.temporalpole_1)
2264	0.91787	(Left-Caudate)(Left-Putamen)(lh.caudalanteriorcingulate_1)(lh.rostralanteriorcingulate_1)(lh.rostralanteriorcingulate_2)
2265	0.91787	(Left-Caudate)(Left-Putamen)(lh.medialorbitofrontal_2)(lh.rostralanteriorcingulate_1)(lh.rostralmiddlefrontal_9)
2266	0.91787	(Left-Caudate)(Left-Putamen)(lh.posteriorcingulate_3)(lh.precentral_4)
2267	0.91787	(Left-Caudate)(Left-Putamen)(lh.precentral_11)(lh.precentral_4)(lh.precentral_7)
2268	0.91787	(Left-Caudate)(Left-Putamen)(lh.rostralmiddlefrontal_1)(lh.rostralmiddlefrontal_4)(lh.rostralmiddlefrontal_9)
2269	0.91787	(Left-Caudate)(Left-Putamen)(lh.superiorfrontal_14)
2270	0.91787	(Left-Caudate)(Left-Thalamus-Proper)(lh.precentral_4)(lh.precentral_7)
2271	0.91787	(Left-Caudate)(lh.caudalanteriorcingulate_2)(lh.superiorfrontal_14)(lh.superiorfrontal_8)
2272	0.91787	(Left-Caudate)(lh.caudalmiddlefrontal_1)(lh.caudalmiddlefrontal_5)
2273	0.91787	(Left-Caudate)(lh.caudalmiddlefrontal_3)(lh.caudalmiddlefrontal_4)(lh.caudalmiddlefrontal_5)
2274	0.91787	(Left-Caudate)(lh.caudalmiddlefrontal_3)(lh.superiorfrontal_12)
2275	0.91787	(Left-Caudate)(lh.insula_1)(lh.postcentral_8)
2276	0.91787	(Left-Caudate)(Right-Caudate)(rh.caudalanteriorcingulate_1)
2277	0.91787	(Left-Hippocampus)(lh.bankssts_3)(lh.superiortemporal_2)
2278	0.91787	(Left-Hippocampus)(lh.superiortemporal_3)(lh.superiortemporal_8)
2279	0.91787	(Left-Pallidum)(Left-Putamen)(lh.precentral_4)
2280	0.91787	(Left-Putamen)(lh.caudalmiddlefrontal_3)(lh.superiorfrontal_11)
2281	0.91787	(Left-Putamen)(lh.lateralorbitofrontal_6)(lh.lateralorbitofrontal_7)(lh.parstriangularis_3)
2282	0.91787	(Left-Putamen)(lh.lateralorbitofrontal_7)(lh.parstriangularis_3)(lh.rostralmiddlefrontal_12)
2283	0.91787	(Left-Putamen)(lh.postcentral_8)(lh.precentral_14)
2284	0.91787	(Left-Putamen)(lh.precentral_12)(lh.precentral_14)
2285	0.91787	(Left-Putamen)(lh.rostralmiddlefrontal_8)
2286	0.91787	(Left-Putamen)(lh.superiorfrontal_11)(lh.superiorfrontal_12)
2287	0.91787	(Left-Thalamus-Proper)(lh.precentral_9)
2288	0.91787	(lh.bankssts_2)(lh.middletemporal_3)
2289	0.91787	(lh.fusiform_2)(lh.lingual_4)(lh.pericalcarine_3)
2290	0.91787	(lh.inferiorparietal_9)(lh.superiorparietal_11)
2291	0.91787	(lh.insula_6)(lh.parsopercularis_1)(lh.parsopercularis_3)(lh.parstriangularis_3)
2292	0.91787	(lh.lateraloccipital_2)(lh.superiorparietal_12)
2293	0.91787	(lh.lateraloccipital_6)(lh.pericalcarine_2)(lh.pericalcarine_3)
2294	0.91787	(lh.lateralorbitofrontal_4)(lh.lateralorbitofrontal_6)(lh.lateralorbitofrontal_7)(lh.parstriangularis_3)
2295	0.91787	(lh.lateralorbitofrontal_4)(lh.lateralorbitofrontal_6)(lh.rostralmiddlefrontal_12)(lh.rostralmiddlefrontal_9)
2296	0.91787	(lh.lateralorbitofrontal_6)(lh.rostralmiddlefrontal_12)(lh.rostralmiddlefrontal_6)(lh.rostralmiddlefrontal_9)
2297	0.91787	(lh.lingual_7)(lh.lingual_8)
2298	0.91787	(lh.medialorbitofrontal_2)(lh.rostralanteriorcingulate_1)(lh.rostralmiddlefrontal_9)(lh.superiorfrontal_3)
2299	0.91787	(lh.paracentral_4)(lh.superiorfrontal_15)(lh.superiorfrontal_16)
2300	0.91787	(lh.precuneus_3)(lh.precuneus_6)(lh.superiorparietal_5)

2301	0.91787	(lh.rostralmiddlefrontal_2)(lh.rostralmiddlefrontal_4)
2302	0.91787	(lh.superiorfrontal_13)(lh.superiorfrontal_14)(lh.superiorfrontal_8)(lh.superiorfrontal_9)
2303	0.91787	(lh.superiorfrontal_2)(lh.superiorfrontal_5)
2304	0.91787	(lh.superiorfrontal_4)(lh.superiorfrontal_6)(lh.superiorfrontal_8)
2305	0.91787	(rh.bankssts_2)(rh.insula_2)
2306	0.91787	(rh.caudalmiddlefrontal_4)(rh.insula_3)(rh.insula_7)
2307	0.91787	(rh.cuneus_4)(rh.pericalcarine_2)
2308	0.91787	(rh.fusiform_1)(rh.fusiform_2)(rh.lateraloccipital_10)(rh.lateraloccipital_8)
2309	0.91787	(rh.fusiform_1)(rh.lateraloccipital_4)(rh.lateraloccipital_7)(rh.lateraloccipital_8)
2310	0.91787	(rh.fusiform_8)(rh.insula_5)
2311	0.91787	(rh.inferiorparietal_10)(rh.inferiorparietal_4)(rh.inferiorparietal_8)
2312	0.91787	(rh.inferiorparietal_10)(rh.lingual_7)
2313	0.91787	(rh.inferiortemporal_2)(rh.inferiortemporal_4)
2314	0.91787	(rh.inferiortemporal_4)(rh.middletemporal_4)(rh.middletemporal_6)
2315	0.91787	(rh.insula_1)(rh.insula_3)(rh.supramarginal_9)
2316	0.91787	(rh.insula_3)(rh.supramarginal_9)
2317	0.91787	(rh.lateraloccipital_10)(rh.lateraloccipital_7)
2318	0.91787	(rh.lateralorbitofrontal_6)(rh.parstriangularis_1)(rh.parstriangularis_3)
2319	0.91787	(rh.lateralorbitofrontal_7)(rh.parstriangularis_1)(rh.rostralmiddlefrontal_12)
2320	0.91787	(rh.parsopercularis_1)(rh.parstriangularis_2)
2321	0.91787	(rh.parstriangularis_2)(rh.parstriangularis_3)
2322	0.91787	(rh.parstriangularis_2)(rh.parstriangularis_4)(rh.rostralmiddlefrontal_3)
2323	0.91787	(rh.parstriangularis_3)(rh.parstriangularis_4)
2324	0.91787	(rh.postcentral_10)(rh.postcentral_8)
2325	0.91787	(rh.precentral_13)(rh.precentral_14)
2326	0.91787	(rh.precuneus_1)(rh.precuneus_4)(rh.superiorparietal_11)
2327	0.91787	(rh.precuneus_7)(rh.superiorparietal_10)(rh.superiorparietal_7)
2328	0.91787	(rh.superiorfrontal_11)(rh.superiorfrontal_13)(rh.superiorfrontal_8)(rh.superiorfrontal_9)
2329	0.91787	(rh.superiorparietal_1)(rh.superiorparietal_10)(rh.superiorparietal_7)
2330	0.91787	(Right-Caudate)(Right-Thalamus-Proper)(rh.supramarginal_9)
2331	0.91787	(Right-Hippocampus)(rh.bankssts_2)(rh.superiortemporal_3)(rh.superiortemporal_9)
2332	0.91787	(Right-Hippocampus)(rh.isthmuscingulate_1)
2333	0.91787	(Right-Hippocampus)(Right-Putamen)(Right-Thalamus-Proper)(rh.insula_2)(rh.supramarginal_9)
2334	0.91787	(Right-Hippocampus)(Right-Thalamus-Proper)(rh.insula_2)(rh.supramarginal_9)
2335	0.91787	(Right-Putamen)(rh.caudalmiddlefrontal_4)(rh.insula_3)(rh.insula_7)
2336	0.91787	(Right-Putamen)(rh.insula_1)(rh.insula_3)(rh.supramarginal_9)
2337	0.91787	(Right-Putamen)(rh.insula_3)(rh.supramarginal_9)
2338	0.91787	(Right-Putamen)(rh.lateralorbitofrontal_2)(rh.lateralorbitofrontal_6)(rh.parstriangularis_1)(rh.rostralmiddlefrontal_12)
2339	0.91787	(Right-Putamen)(rh.lateralorbitofrontal_7)(rh.parstriangularis_1)
2340	0.91787	(Right-Putamen)(rh.rostralmiddlefrontal_1)
2341	0.91787	(Right-Putamen)(rh.superiorfrontal_13)
2342	0.91546	(Left-Accumbens-area)(Left-Putamen)(lh.rostralanteriorcingulate_1)
2343	0.91546	(Left-Caudate)(Left-Pallidum)(lh.precentral_4)
2344	0.91546	(Left-Caudate)(Left-Putamen)(Left-Thalamus-Proper)(lh.precentral_4)(lh.precentral_7)
2345	0.91546	(Left-Caudate)(Left-Putamen)(lh.caudalmiddlefrontal_3)(lh.superiorfrontal_11)
2346	0.91546	(Left-Caudate)(Left-Putamen)(lh.parsopercularis_3)(lh.rostralmiddlefrontal_1)
2347	0.91546	(Left-Caudate)(Left-Putamen)(lh.parstriangularis_3)(lh.rostralanteriorcingulate_1)
2348	0.91546	(Left-Caudate)(Left-Putamen)(lh.rostralanteriorcingulate_1)(lh.rostralmiddlefrontal_12)
2349	0.91546	(Left-Caudate)(Left-Putamen)(lh.rostralanteriorcingulate_2)(lh.rostralmiddlefrontal_4)
2350	0.91546	(Left-Caudate)(Left-Putamen)(lh.rostralanteriorcingulate_2)(lh.superiorfrontal_4)

2351	0.91546	(Left-Caudate)(Left-Putamen)(lh.rostralmiddlefrontal_4)(lh.superiorfrontal_4)
2352	0.91546	(Left-Caudate)(lh.caudalmiddlefrontal_1)(lh.caudalmiddlefrontal_3)(lh.caudalmiddlefrontal_4)
2353	0.91546	(Left-Caudate)(lh.lateralorbitofrontal_7)(lh.rostralanteriorcingulate_1)
2354	0.91546	(Left-Caudate)(lh.lateralorbitofrontal_7)(lh.rostralmiddlefrontal_12)
2355	0.91546	(Left-Caudate)(lh.parstriangularis_3)(lh.rostralanteriorcingulate_1)(lh.rostralmiddlefrontal_9)
2356	0.91546	(Left-Caudate)(lh.parstriangularis_3)(lh.rostralmiddlefrontal_1)(lh.rostralmiddlefrontal_4)
2357	0.91546	(Left-Caudate)(lh.postcentral_3)
2358	0.91546	(Left-Caudate)(lh.precentral_11)(lh.precentral_14)
2359	0.91546	(Left-Caudate)(lh.rostralmiddlefrontal_8)
2360	0.91546	(Left-Caudate)(lh.rostralmiddlefrontal_9)(lh.superiorfrontal_4)
2361	0.91546	(Left-Caudate)(Right-Caudate)(lh.caudalanteriorcingulate_2)
2362	0.91546	(Left-Putamen)(Left-Thalamus-Proper)(lh.precentral_9)
2363	0.91546	(Left-Putamen)(lh.caudalanteriorcingulate_2)(lh.rostralanteriorcingulate_1)(lh.rostralanteriorcingulate_2)
2364	0.91546	(Left-Putamen)(lh.insula_1)(lh.superiorparietal_3)
2365	0.91546	(Left-Putamen)(lh.insula_6)(lh.parsopercularis_1)(lh.parstriangularis_3)
2366	0.91546	(Left-Putamen)(lh.lateralorbitofrontal_7)(lh.medialorbitofrontal_2)(lh.rostralmiddlefrontal_9)
2367	0.91546	(lh.caudalanteriorcingulate_2)(lh.superiorfrontal_4)(lh.superiorfrontal_8)
2368	0.91546	(lh.lateraloccipital_1)(lh.pericalcarine_1)(lh.pericalcarine_2)
2369	0.91546	(lh.lateraloccipital_11)(lh.lateraloccipital_3)
2370	0.91546	(lh.lateraloccipital_6)(lh.lingual_4)
2371	0.91546	(lh.lateralorbitofrontal_4)(lh.lateralorbitofrontal_7)(lh.rostralmiddlefrontal_12)
2372	0.91546	(lh.lateralorbitofrontal_7)(lh.parstriangularis_3)(lh.rostralmiddlefrontal_12)(lh.rostralmiddlefrontal_9)
2373	0.91546	(lh.medialorbitofrontal_1)(lh.medialorbitofrontal_3)
2374	0.91546	(lh.parstriangularis_3)(lh.rostralmiddlefrontal_12)(lh.rostralmiddlefrontal_6)(lh.rostralmiddlefrontal_9)
2375	0.91546	(lh.precuneus_6)(lh.superiorparietal_7)
2376	0.91546	(lh.rostralanteriorcingulate_1)(lh.rostralmiddlefrontal_4)
2377	0.91546	(lh.rostralanteriorcingulate_2)(lh.rostralmiddlefrontal_8)(lh.superiorfrontal_4)
2378	0.91546	(lh.rostralanteriorcingulate_2)(lh.superiorfrontal_10)
2379	0.91546	(lh.superiorfrontal_2)(lh.superiorfrontal_3)(lh.superiorfrontal_5)
2380	0.91546	(lh.superiorfrontal_3)(lh.superiorfrontal_6)
2381	0.91546	(lh.superiorfrontal_5)(lh.superiorfrontal_6)(lh.superiorfrontal_7)
2382	0.91546	(lh.superiorfrontal_6)(lh.superiorfrontal_7)(lh.superiorfrontal_8)
2383	0.91546	(rh.caudalmiddlefrontal_1)(rh.precentral_10)
2384	0.91546	(rh.fusiform_7)(rh.middletemporal_4)
2385	0.91546	(rh.inferiorparietal_4)(rh.supramarginal_4)
2386	0.91546	(rh.inferiortemporal_6)(rh.inferiortemporal_7)(rh.middletemporal_1)
2387	0.91546	(rh.insula_1)(rh.superiorparietal_1)(rh.supramarginal_1)(rh.supramarginal_9)
2388	0.91546	(rh.insula_5)(rh.middletemporal_9)
2389	0.91546	(rh.isthmuscingulate_2)(rh.lingual_7)
2390	0.91546	(rh.isthmuscingulate_2)(rh.precuneus_2)(rh.precuneus_4)
2391	0.91546	(rh.lateraloccipital_10)(rh.lingual_7)
2392	0.91546	(rh.lateraloccipital_3)(rh.lateraloccipital_4)(rh.pericalcarine_4)
2393	0.91546	(rh.lateraloccipital_3)(rh.pericalcarine_1)
2394	0.91546	(rh.lateraloccipital_4)(rh.lateraloccipital_8)(rh.lingual_2)
2395	0.91546	(rh.lateralorbitofrontal_6)(rh.medialorbitofrontal_2)
2396	0.91546	(rh.lingual_4)(rh.pericalcarine_3)
2397	0.91546	(rh.parsopercularis_1)(rh.parsopercularis_2)
2398	0.91546	(rh.postcentral_2)(rh.postcentral_3)
2399	0.91546	(rh.precuneus_4)(rh.superiorparietal_1)(rh.superiorparietal_7)
2400	0.91546	(rh.rostralmiddlefrontal_5)(rh.superiorfrontal_2)

2401	0.91546	(rh.superiorfrontal_1)(rh.superiorfrontal_4)
2402	0.91546	(rh.superiorfrontal_5)(rh.superiorfrontal_6)(rh.superiorfrontal_8)(rh.superiorfrontal_9)
2403	0.91546	(rh.superiorfrontal_6)(rh.superiorfrontal_7)(rh.superiorfrontal_8)(rh.superiorfrontal_9)
2404	0.91546	(rh.supramarginal_3)(rh.supramarginal_8)
2405	0.91546	(Right-Accumbens-area)(Right-Amygdala)(Right-Thalamus-Proper)
2406	0.91546	(Right-Accumbens-area)(Right-Amygdala)
2407	0.91546	(Right-Accumbens-area)(Right-Hippocampus)(Right-Pallidum)(Right-Thalamus-Proper)
2408	0.91546	(Right-Accumbens-area)(Right-Hippocampus)(Right-Pallidum)
2409	0.91546	(Right-Accumbens-area)(Right-Hippocampus)(Right-Thalamus-Proper)
2410	0.91546	(Right-Accumbens-area)(Right-Hippocampus)
2411	0.91546	(Right-Caudate)(lh.caudalanteriorcingulate_2)
2412	0.91546	(Right-Caudate)(rh.insula_1)(rh.precentral_11)
2413	0.91546	(Right-Caudate)(rh.insula_7)(rh.parsopercularis_4)
2414	0.91546	(Right-Caudate)(rh.rostralmiddlefrontal_3)
2415	0.91546	(Right-Caudate)(Right-Putamen)(Right-Thalamus-Proper)(rh.supramarginal_9)
2416	0.91546	(Right-Hippocampus)(rh.bankssts_2)(rh.insula_2)
2417	0.91546	(Right-Hippocampus)(rh.inferiorparietal_10)(rh.inferiorparietal_4)
2418	0.91546	(Right-Putamen)(rh.caudalmiddlefrontal_4)(rh.rostralmiddlefrontal_3)
2419	0.91546	(Right-Putamen)(rh.lateralorbitofrontal_1)(rh.lateralorbitofrontal_4)
2420	0.91546	(Right-Putamen)(rh.lateralorbitofrontal_2)(rh.lateralorbitofrontal_6)(rh.lateralorbitofrontal_7)(rh.rostralmiddlefrontal_12)
2421	0.91546	(Right-Putamen)(rh.lateralorbitofrontal_6)(rh.lateralorbitofrontal_7)(rh.parstriangularis_1)
2422	0.91546	(Right-Putamen)(rh.precentral_8)
2423	0.91304	(Left-Amygdala)(lh.fusiform_8)
2424	0.91304	(Left-Caudate)(Left-Pallidum)(Left-Putamen)(lh.precentral_4)
2425	0.91304	(Left-Caudate)(Left-Putamen)(lh.caudalanteriorcingulate_2)(lh.rostralanteriorcingulate_1)(lh.rostralanteriorcingulate_2)
2426	0.91304	(Left-Caudate)(Left-Putamen)(lh.insula_1)(lh.postcentral_8)
2427	0.91304	(Left-Caudate)(lh.caudalanteriorcingulate_2)(lh.superiorfrontal_10)
2428	0.91304	(Left-Caudate)(lh.caudalanteriorcingulate_2)(lh.superiorfrontal_11)(lh.superiorfrontal_12)(lh.superiorfrontal_14)
2429	0.91304	(Left-Caudate)(lh.caudalanteriorcingulate_2)(lh.superiorfrontal_12)(lh.superiorfrontal_8)
2430	0.91304	(Left-Caudate)(lh.insula_6)(lh.parstriangularis_3)(lh.rostralmiddlefrontal_1)
2431	0.91304	(Left-Caudate)(lh.lateralorbitofrontal_2)(lh.lateralorbitofrontal_7)
2432	0.91304	(Left-Caudate)(lh.rostralanteriorcingulate_1)(lh.rostralmiddlefrontal_12)(lh.rostralmiddlefrontal_9)
2433	0.91304	(Left-Caudate)(lh.rostralanteriorcingulate_2)(lh.rostralmiddlefrontal_9)
2434	0.91304	(Left-Caudate)(lh.superiorfrontal_10)(lh.superiorfrontal_11)(lh.superiorfrontal_8)
2435	0.91304	(Left-Hippocampus)(Left-Thalamus-Proper)(lh.isthmuscingulate_2)(lh.isthmuscingulate_3)
2436	0.91304	(Left-Hippocampus)(lh.fusiform_6)
2437	0.91304	(Left-Hippocampus)(lh.superiortemporal_6)(lh.superiortemporal_8)
2438	0.91304	(Left-Putamen)(lh.insula_3)(lh.precentral_11)
2439	0.91304	(Left-Putamen)(lh.insula_6)(lh.parsopercularis_3)(lh.parstriangularis_3)
2440	0.91304	(Left-Putamen)(lh.lateralorbitofrontal_4)(lh.lateralorbitofrontal_6)(lh.parstriangularis_3)(lh.rostralmiddlefrontal_9)
2441	0.91304	(Left-Putamen)(lh.lateralorbitofrontal_4)(lh.lateralorbitofrontal_7)(lh.rostralmiddlefrontal_12)
2442	0.91304	(Left-Putamen)(lh.lateralorbitofrontal_6)(lh.lateralorbitofrontal_7)(lh.rostralmiddlefrontal_9)
2443	0.91304	(Left-Putamen)(lh.postcentral_8)(lh.precentral_11)
2444	0.91304	(Left-Putamen)(lh.superiorfrontal_3)
2445	0.91304	(Left-Thalamus-Proper)(lh.insula_1)(lh.supramarginal_1)
2446	0.91304	(Left-Thalamus-Proper)(lh.isthmuscingulate_1)
2447	0.91304	(Left-Thalamus-Proper)(lh.supramarginal_1)
2448	0.91304	(lh.caudalanteriorcingulate_2)(lh.caudalmiddlefrontal_1)
2449	0.91304	(lh.fusiform_2)(lh.lateraloccipital_11)(lh.lateraloccipital_6)(lh.lingual_1)
2450	0.91304	(lh.fusiform_5)(lh.inferiortemporal_8)(lh.lateraloccipital_9)

2451	0.91304	(lh.fusiform_7)(lh.inferiortemporal_1)
2452	0.91304	(lh.inferiortemporal_3)(lh.inferiortemporal_4)
2453	0.91304	(lh.insula_1)(lh.supramarginal_1)(lh.supramarginal_9)
2454	0.91304	(lh.insula_3)(lh.precentral_11)
2455	0.91304	(lh.insula_6)(lh.rostralmiddlefrontal_1)(lh.rostralmiddlefrontal_4)
2456	0.91304	(lh.lateralorbitofrontal_4)(lh.parstriangularis_3)(lh.rostralmiddlefrontal_12)(lh.rostralmiddlefrontal_9)
2457	0.91304	(lh.lateralorbitofrontal_6)(lh.parsorbitalis_1)(lh.parstriangularis_1)
2458	0.91304	(lh.lateralorbitofrontal_7)(lh.rostralanteriorcingulate_1)(lh.rostralmiddlefrontal_12)
2459	0.91304	(lh.lingual_3)(lh.lingual_4)(lh.pericalcarine_3)
2460	0.91304	(lh.medialorbitofrontal_2)(lh.rostralanteriorcingulate_1)(lh.rostralmiddlefrontal_12)(lh.superiorfrontal_3)
2461	0.91304	(lh.medialorbitofrontal_2)(lh.rostralmiddlefrontal_12)(lh.superiorfrontal_1)
2462	0.91304	(lh.paracentral_4)(lh.paracentral_5)(lh.superiorfrontal_15)
2463	0.91304	(lh.parsopercularis_1)(lh.parsopercularis_3)(lh.parstriangularis_3)(lh.rostralmiddlefrontal_1)
2464	0.91304	(lh.parsopercularis_2)(lh.parstriangularis_3)(lh.rostralmiddlefrontal_1)
2465	0.91304	(lh.postcentral_14)(lh.supramarginal_2)(lh.supramarginal_4)
2466	0.91304	(lh.precuneus_3)(lh.superiorparietal_7)
2467	0.91304	(lh.precuneus_9)(lh.superiorparietal_6)
2468	0.91304	(lh.rostralanteriorcingulate_1)(lh.rostralanteriorcingulate_2)(lh.rostralmiddlefrontal_9)
2469	0.91304	(lh.rostralanteriorcingulate_1)(lh.rostralmiddlefrontal_12)(lh.rostralmiddlefrontal_9)(lh.superiorfrontal_3)
2470	0.91304	(lh.rostralanteriorcingulate_2)(lh.superiorfrontal_4)(lh.superiorfrontal_8)
2471	0.91304	(lh.rostralmiddlefrontal_5)(lh.rostralmiddlefrontal_7)
2472	0.91304	(lh.superiorfrontal_10)(lh.superiorfrontal_4)(lh.superiorfrontal_5)
2473	0.91304	(lh.superiorfrontal_10)(lh.superiorfrontal_5)
2474	0.91304	(lh.superiorfrontal_3)(lh.superiorfrontal_4)(lh.superiorfrontal_6)
2475	0.91304	(lh.superiorfrontal_4)(lh.superiorfrontal_5)(lh.superiorfrontal_6)(lh.superiorfrontal_7)
2476	0.91304	(lh.superiorfrontal_4)(lh.superiorfrontal_6)(lh.superiorfrontal_7)(lh.superiorfrontal_8)
2477	0.91304	(lh.supramarginal_1)(lh.supramarginal_6)(lh.supramarginal_9)
2478	0.91304	(rh.bankssts_3)(rh.inferiorparietal_4)
2479	0.91304	(rh.caudalmiddlefrontal_4)(rh.rostralmiddlefrontal_2)(rh.rostralmiddlefrontal_3)
2480	0.91304	(rh.fusiform_1)(rh.inferiorparietal_10)(rh.lateraloccipital_8)
2481	0.91304	(rh.fusiform_1)(rh.lateraloccipital_10)(rh.lateraloccipital_7)
2482	0.91304	(rh.inferiorparietal_12)(rh.pericalcarine_1)
2483	0.91304	(rh.inferiorparietal_5)(rh.inferiorparietal_8)
2484	0.91304	(rh.insula_4)(rh.insula_5)(rh.insula_6)
2485	0.91304	(rh.lateralorbitofrontal_7)(rh.rostralmiddlefrontal_11)(rh.superiorfrontal_2)
2486	0.91304	(rh.precuneus_5)(rh.precuneus_7)
2487	0.91304	(rh.rostralmiddlefrontal_12)(rh.rostralmiddlefrontal_3)(rh.rostralmiddlefrontal_8)
2488	0.91304	(rh.rostralmiddlefrontal_5)(rh.rostralmiddlefrontal_9)
2489	0.91304	(rh.superiorfrontal_5)(rh.superiorfrontal_6)(rh.superiorfrontal_7)(rh.superiorfrontal_9)
2490	0.91304	(Right-Accumbens-area)(Right-Amygdala)(Right-Pallidum)(Right-Thalamus-Proper)
2491	0.91304	(Right-Accumbens-area)(Right-Amygdala)(Right-Pallidum)
2492	0.91304	(Right-Caudate)(rh.posteriorcingulate_2)(rh.superiorfrontal_11)
2493	0.91304	(Right-Caudate)(Right-Putamen)(rh.caudalanteriorcingulate_1)
2494	0.91304	(Right-Caudate)(Right-Putamen)(rh.insula_7)(rh.parsopercularis_4)
2495	0.91304	(Right-Caudate)(Right-Putamen)(rh.lateralorbitofrontal_5)
2496	0.91304	(Right-Hippocampus)(rh.bankssts_2)(rh.inferiorparietal_10)
2497	0.91304	(Right-Hippocampus)(rh.isthmuscingulate_2)(rh.lingual_7)
2498	0.91304	(Right-Hippocampus)(rh.precuneus_4)
2499	0.91304	(Right-Putamen)(rh.caudalanteriorcingulate_1)
2500	0.91304	(Right-Putamen)(rh.insula_4)(rh.insula_5)(rh.insula_6)

2501	0.91304	(Right-Putamen)(rh.lateralorbitofrontal_6)(rh.medialorbitofrontal_2)
2502	0.91304	(Right-Putamen)(rh.lateralorbitofrontal_6)(rh.rostralmiddlefrontal_13)
2503	0.91304	(Right-Putamen)(rh.medialorbitofrontal_2)(rh.superiorfrontal_2)
2504	0.91304	(Right-Putamen)(rh.rostralmiddlefrontal_12)(rh.rostralmiddlefrontal_8)(rh.superiorfrontal_2)
2505	0.91304	(Right-Putamen)(Right-Thalamus-Proper)(rh.precentral_11)
2506	0.91063	(Left-Accumbens-area)(Right-Caudate)
2507	0.91063	(Left-Amygdala)(Left-Hippocampus)(Left-Putamen)(lh.insula_4)
2508	0.91063	(Left-Caudate)(Left-Putamen)(lh.lateralorbitofrontal_7)(lh.rostralmiddlefrontal_9)
2509	0.91063	(Left-Caudate)(Left-Putamen)(lh.medialorbitofrontal_2)(lh.rostralmiddlefrontal_12)
2510	0.91063	(Left-Caudate)(Left-Putamen)(lh.precentral_11)(lh.precentral_14)
2511	0.91063	(Left-Caudate)(Left-Putamen)(lh.superiorfrontal_11)(lh.superiorfrontal_12)
2512	0.91063	(Left-Caudate)(Left-Putamen)(lh.superiorfrontal_11)(lh.superiorfrontal_4)
2513	0.91063	(Left-Caudate)(Left-Thalamus-Proper)(lh.precentral_9)
2514	0.91063	(Left-Caudate)(lh.insula_6)(lh.rostralmiddlefrontal_4)
2515	0.91063	(Left-Caudate)(lh.medialorbitofrontal_2)(lh.rostralmiddlefrontal_12)(lh.rostralmiddlefrontal_9)
2516	0.91063	(Left-Caudate)(lh.parstriangularis_3)(lh.rostralmiddlefrontal_4)(lh.rostralmiddlefrontal_9)
2517	0.91063	(Left-Caudate)(lh.rostralanteriorcingulate_1)(lh.rostralmiddlefrontal_4)
2518	0.91063	(Left-Hippocampus)(Left-Putamen)(lh.insula_4)
2519	0.91063	(Left-Hippocampus)(lh.isthmuscingulate_3)(lh.lingual_6)(lh.precuneus_11)
2520	0.91063	(Left-Putamen)(lh.lateralorbitofrontal_4)(lh.lateralorbitofrontal_6)(lh.rostralmiddlefrontal_12)(lh.rostralmiddlefrontal_9)
2521	0.91063	(Left-Putamen)(lh.lateralorbitofrontal_4)(lh.lateralorbitofrontal_7)(lh.parstriangularis_3)
2522	0.91063	(Left-Putamen)(lh.lateralorbitofrontal_6)(lh.rostralmiddlefrontal_1)
2523	0.91063	(Left-Putamen)(lh.medialorbitofrontal_2)(lh.rostralanteriorcingulate_1)(lh.rostralmiddlefrontal_12)(lh.rostralmiddlefrontal_9)
2524	0.91063	(Left-Thalamus-Proper)(lh.postcentral_3)
2525	0.91063	(lh.bankssts_2)(lh.fusiform_7)
2526	0.91063	(lh.caudalanteriorcingulate_2)(lh.rostralanteriorcingulate_2)(lh.superiorfrontal_8)
2527	0.91063	(lh.caudalanteriorcingulate_2)(lh.superiorfrontal_11)(lh.superiorfrontal_4)
2528	0.91063	(lh.cuneus_1)(lh.precuneus_9)
2529	0.91063	(lh.fusiform_2)(lh.lingual_6)
2530	0.91063	(lh.fusiform_4)(lh.fusiform_7)
2531	0.91063	(lh.inferiorparietal_7)(lh.inferiorparietal_9)
2532	0.91063	(lh.inferiortemporal_5)(lh.inferiortemporal_6)
2533	0.91063	(lh.inferiortemporal_8)(lh.middletemporal_1)
2534	0.91063	(lh.insula_1)(lh.precentral_11)(lh.precentral_7)
2535	0.91063	(lh.insula_6)(lh.rostralmiddlefrontal_9)
2536	0.91063	(lh.isthmuscingulate_3)(lh.precuneus_11)(lh.precuneus_6)
2537	0.91063	(lh.lateraloccipital_10)(lh.lateraloccipital_11)
2538	0.91063	(lh.lateraloccipital_2)(lh.pericalcarine_1)(lh.pericalcarine_2)
2539	0.91063	(lh.lateralorbitofrontal_6)(lh.lateralorbitofrontal_7)(lh.rostralmiddlefrontal_12)(lh.rostralmiddlefrontal_9)
2540	0.91063	(lh.lateralorbitofrontal_6)(lh.parstriangularis_3)(lh.rostralmiddlefrontal_12)(lh.rostralmiddlefrontal_6)
2541	0.91063	(lh.lingual_1)(lh.lingual_3)(lh.lingual_4)
2542	0.91063	(lh.medialorbitofrontal_1)(lh.rostralmiddlefrontal_12)
2543	0.91063	(lh.medialorbitofrontal_2)(lh.medialorbitofrontal_4)
2544	0.91063	(lh.medialorbitofrontal_2)(lh.rostralmiddlefrontal_9)(lh.superiorfrontal_1)
2545	0.91063	(lh.precuneus_11)(lh.precuneus_9)(lh.superiorparietal_11)
2546	0.91063	(lh.rostralanteriorcingulate_2)(lh.superiorfrontal_11)
2547	0.91063	(lh.rostralmiddlefrontal_4)(lh.superiorfrontal_3)
2548	0.91063	(lh.rostralmiddlefrontal_8)(lh.superiorfrontal_2)
2549	0.91063	(lh.superiorfrontal_1)(lh.superiorfrontal_4)
2550	0.91063	(lh.superiorfrontal_11)(lh.superiorfrontal_13)(lh.superiorfrontal_14)

2551	0.91063	(rh.caudalmiddlefrontal_4)(rh.insula_3)(rh.precentral_7)
2552	0.91063	(rh.caudalmiddlefrontal_4)(rh.rostralmiddlefrontal_5)
2553	0.91063	(rh.caudalmiddlefrontal_4)(rh.superiorfrontal_13)(rh.superiorfrontal_9)
2554	0.91063	(rh.caudalmiddlefrontal_4)(rh.superiorfrontal_7)(rh.superiorfrontal_9)
2555	0.91063	(rh.fusiform_1)(rh.lateraloccipital_4)(rh.lingual_2)
2556	0.91063	(rh.fusiform_2)(rh.inferiorparietal_10)(rh.lateraloccipital_10)
2557	0.91063	(rh.fusiform_5)(rh.inferiorparietal_10)
2558	0.91063	(rh.inferiorparietal_10)(rh.middletemporal_1)
2559	0.91063	(rh.inferiorparietal_11)(rh.lateraloccipital_10)
2560	0.91063	(rh.inferiorparietal_12)(rh.inferiorparietal_7)
2561	0.91063	(rh.lateraloccipital_4)(rh.lateraloccipital_7)(rh.pericalcarine_3)
2562	0.91063	(rh.lateraloccipital_4)(rh.lingual_2)(rh.pericalcarine_3)
2563	0.91063	(rh.lateraloccipital_5)(rh.lateraloccipital_8)
2564	0.91063	(rh.lateralorbitofrontal_4)(rh.lateralorbitofrontal_5)(rh.lateralorbitofrontal_7)
2565	0.91063	(rh.lateralorbitofrontal_6)(rh.lateralorbitofrontal_7)(rh.medialorbitofrontal_2)
2566	0.91063	(rh.lateralorbitofrontal_6)(rh.lateralorbitofrontal_7)(rh.parstriangularis_1)(rh.rostralmiddlefrontal_12)
2567	0.91063	(rh.precuneus_10)(rh.superiorparietal_2)
2568	0.91063	(rh.precuneus_10)(rh.superiorparietal_4)
2569	0.91063	(rh.precuneus_5)(rh.superiorparietal_9)
2570	0.91063	(rh.rostralmiddlefrontal_11)(rh.rostralmiddlefrontal_8)(rh.superiorfrontal_2)
2571	0.91063	(rh.rostralmiddlefrontal_2)(rh.rostralmiddlefrontal_4)
2572	0.91063	(rh.rostralmiddlefrontal_3)(rh.rostralmiddlefrontal_4)(rh.rostralmiddlefrontal_5)
2573	0.91063	(rh.superiorfrontal_2)(rh.superiorfrontal_8)
2574	0.91063	(Right-Caudate)(Right-Putamen)(rh.insula_1)(rh.precentral_11)
2575	0.91063	(Right-Caudate)(Right-Putamen)(rh.superiorfrontal_7)
2576	0.91063	(Right-Caudate)(Right-Thalamus-Proper)(rh.posteriorcingulate_2)
2577	0.91063	(Right-Hippocampus)(rh.inferiortemporal_1)
2578	0.91063	(Right-Putamen)(rh.caudalmiddlefrontal_4)(rh.insula_3)(rh.precentral_7)
2579	0.91063	(Right-Putamen)(rh.caudalmiddlefrontal_4)(rh.superiorfrontal_13)
2580	0.91063	(Right-Putamen)(rh.caudalmiddlefrontal_4)(rh.superiorfrontal_7)
2581	0.91063	(Right-Putamen)(rh.lateralorbitofrontal_5)(rh.lateralorbitofrontal_7)(rh.medialorbitofrontal_2)
2582	0.91063	(Right-Putamen)(rh.lateralorbitofrontal_7)(rh.rostralmiddlefrontal_13)
2583	0.91063	(Right-Putamen)(rh.parsopercularis_4)(rh.parstriangularis_1)
2584	0.91063	(Right-Putamen)(rh.postcentral_4)
2585	0.91063	(Right-Putamen)(rh.postcentral_5)
2586	0.91063	(Right-Putamen)(rh.rostralmiddlefrontal_3)(rh.rostralmiddlefrontal_5)
2587	0.91063	(Right-Thalamus-Proper)(rh.posteriorcingulate_2)
2588	0.90821	(Left-Accumbens-area)(Left-Caudate)(Left-Putamen)(lh.rostralanteriorcingulate_1)
2589	0.90821	(Left-Accumbens-area)(Left-Putamen)(lh.medialorbitofrontal_5)
2590	0.90821	(Left-Caudate)(Left-Hippocampus)(Left-Putamen)(Left-Thalamus-Proper)(lh.insula_1)
2591	0.90821	(Left-Caudate)(Left-Hippocampus)(Left-Putamen)(lh.insula_1)
2592	0.90821	(Left-Caudate)(Left-Hippocampus)(Left-Thalamus-Proper)(lh.insula_1)
2593	0.90821	(Left-Caudate)(Left-Hippocampus)(lh.insula_1)
2594	0.90821	(Left-Caudate)(Left-Putamen)(Left-Thalamus-Proper)(lh.precentral_9)
2595	0.90821	(Left-Caudate)(Left-Putamen)(lh.caudalmiddlefrontal_1)(lh.caudalmiddlefrontal_4)
2596	0.90821	(Left-Caudate)(Left-Putamen)(lh.caudalmiddlefrontal_3)(lh.insula_6)(lh.rostralmiddlefrontal_1)
2597	0.90821	(Left-Caudate)(Left-Putamen)(lh.insula_6)(lh.rostralmiddlefrontal_4)
2598	0.90821	(Left-Caudate)(Left-Putamen)(lh.lateralorbitofrontal_2)(lh.lateralorbitofrontal_7)
2599	0.90821	(Left-Caudate)(Left-Putamen)(lh.lateralorbitofrontal_6)
2600	0.90821	(Left-Caudate)(Left-Putamen)(lh.lateralorbitofrontal_7)(lh.rostralanteriorcingulate_1)

2601	0.90821	(Left-Caudate)(Left-Putamen)(lh.lateralorbitofrontal_7)(lh.rostralmiddlefrontal_12)
2602	0.90821	(Left-Caudate)(Left-Putamen)(lh.parstriangularis_3)(lh.rostralmiddlefrontal_4)
2603	0.90821	(Left-Caudate)(Left-Putamen)(lh.rostralanteriorcingulate_1)(lh.rostralmiddlefrontal_12)(lh.rostralmiddlefrontal_9)
2604	0.90821	(Left-Caudate)(lh.caudalanteriorcingulate_2)(lh.caudalmiddlefrontal_1)
2605	0.90821	(Left-Caudate)(lh.caudalanteriorcingulate_2)(lh.posteriorcingulate_3)(lh.superiorfrontal_14)
2606	0.90821	(Left-Caudate)(lh.caudalmiddlefrontal_6)(lh.precentral_11)(lh.precentral_9)
2607	0.90821	(Left-Caudate)(lh.insula_1)(lh.supramarginal_1)
2608	0.90821	(Left-Caudate)(lh.medialorbitofrontal_2)(lh.medialorbitofrontal_4)
2609	0.90821	(Left-Caudate)(lh.medialorbitofrontal_2)(lh.rostralanteriorcingulate_1)(lh.rostralmiddlefrontal_12)
2610	0.90821	(Left-Caudate)(lh.postcentral_8)(lh.precentral_4)
2611	0.90821	(Left-Caudate)(lh.superiorfrontal_11)(lh.superiorfrontal_14)(lh.superiorfrontal_8)
2612	0.90821	(Left-Hippocampus)(lh.superiortemporal_11)
2613	0.90821	(Left-Hippocampus)(lh.superiortemporal_3)(lh.superiortemporal_6)
2614	0.90821	(Left-Pallidum)(Left-Thalamus-Proper)(lh.precentral_4)
2615	0.90821	(Left-Putamen)(lh.caudalanteriorcingulate_2)(lh.superiorfrontal_14)
2616	0.90821	(Left-Putamen)(lh.caudalmiddlefrontal_1)(lh.caudalmiddlefrontal_3)(lh.caudalmiddlefrontal_4)
2617	0.90821	(Left-Putamen)(lh.caudalmiddlefrontal_1)(lh.rostralmiddlefrontal_4)
2618	0.90821	(Left-Putamen)(lh.caudalmiddlefrontal_3)(lh.rostralmiddlefrontal_1)(lh.rostralmiddlefrontal_4)
2619	0.90821	(Left-Putamen)(lh.insula_1)(lh.precentral_11)(lh.precentral_7)
2620	0.90821	(Left-Putamen)(lh.lateralorbitofrontal_7)(lh.parstriangularis_3)(lh.rostralmiddlefrontal_9)
2621	0.90821	(Left-Putamen)(lh.parstriangularis_3)(lh.rostralmiddlefrontal_1)(lh.rostralmiddlefrontal_4)(lh.rostralmiddlefrontal_9)
2622	0.90821	(Left-Putamen)(lh.precentral_16)
2623	0.90821	(lh.caudalmiddlefrontal_4)(lh.precentral_9)
2624	0.90821	(lh.cuneus_3)(lh.lateraloccipital_1)
2625	0.90821	(lh.fusiform_1)(lh.fusiform_4)
2626	0.90821	(lh.fusiform_3)(lh.lateraloccipital_9)
2627	0.90821	(lh.fusiform_5)(lh.inferiorparietal_4)
2628	0.90821	(lh.inferiorparietal_2)(lh.inferiorparietal_3)
2629	0.90821	(lh.insula_2)(lh.postcentral_14)
2630	0.90821	(lh.lateraloccipital_3)(lh.pericalcarine_1)(lh.pericalcarine_2)
2631	0.90821	(lh.lateralorbitofrontal_6)(lh.rostralmiddlefrontal_1)(lh.rostralmiddlefrontal_6)
2632	0.90821	(lh.lingual_1)(lh.lingual_2)
2633	0.90821	(lh.medialorbitofrontal_2)(lh.rostralanteriorcingulate_1)(lh.superiorfrontal_4)
2634	0.90821	(lh.medialorbitofrontal_2)(lh.rostralmiddlefrontal_12)(lh.superiorfrontal_1)(lh.superiorfrontal_3)
2635	0.90821	(lh.parahippocampal_1)(lh.parahippocampal_2)
2636	0.90821	(lh.parsorbitalis_1)(lh.rostralmiddlefrontal_6)
2637	0.90821	(lh.parstriangularis_2)(lh.rostralmiddlefrontal_1)
2638	0.90821	(lh.precentral_7)(lh.precentral_9)
2639	0.90821	(lh.rostralmiddlefrontal_8)(lh.rostralmiddlefrontal_9)(lh.superiorfrontal_4)
2640	0.90821	(lh.superiorfrontal_1)(lh.superiorfrontal_3)(lh.superiorfrontal_4)
2641	0.90821	(lh.superiorfrontal_10)(lh.superiorfrontal_11)(lh.superiorfrontal_9)
2642	0.90821	(lh.superiorfrontal_11)(lh.superiorfrontal_13)(lh.superiorfrontal_9)
2643	0.90821	(lh.superiorfrontal_4)(lh.superiorfrontal_7)(lh.superiorfrontal_9)
2644	0.90821	(lh.superiorfrontal_4)(lh.superiorfrontal_9)
2645	0.90821	(lh.superiorparietal_2)(lh.superiorparietal_3)
2646	0.90821	(lh.superiorparietal_5)(lh.supramarginal_1)
2647	0.90821	(lh.superiortemporal_3)(lh.superiortemporal_6)(lh.superiortemporal_8)
2648	0.90821	(rh.bankssts_1)(rh.inferiortemporal_4)
2649	0.90821	(rh.bankssts_1)(rh.inferiortemporal_7)
2650	0.90821	(rh.caudalmiddlefrontal_3)(rh.caudalmiddlefrontal_4)(rh.caudalmiddlefrontal_5)

2651	0.90821	(rh.fusiform_1)(rh.inferiorparietal_10)(rh.lateraloccipital_10)
2652	0.90821	(rh.fusiform_8)(rh.superiortemporal_10)
2653	0.90821	(rh.inferiorparietal_12)(rh.lingual_7)
2654	0.90821	(rh.inferiorparietal_12)(rh.precuneus_2)(rh.superiorparietal_11)
2655	0.90821	(rh.insula_1)(rh.precentral_11)(rh.precentral_7)
2656	0.90821	(rh.insula_1)(rh.supramarginal_1)(rh.supramarginal_8)(rh.supramarginal_9)
2657	0.90821	(rh.lateraloccipital_10)(rh.lateraloccipital_7)(rh.lateraloccipital_8)
2658	0.90821	(rh.lateralorbitofrontal_6)(rh.parsorbitalis_1)
2659	0.90821	(rh.lateralorbitofrontal_7)(rh.rostralmiddlefrontal_12)(rh.superiorfrontal_2)
2660	0.90821	(rh.parsopercularis_2)(rh.parsopercularis_4)(rh.parstriangularis_2)
2661	0.90821	(rh.parstriangularis_1)(rh.parstriangularis_4)(rh.rostralmiddlefrontal_12)(rh.rostralmiddlefrontal_8)
2662	0.90821	(rh.parstriangularis_1)(rh.rostralmiddlefrontal_12)(rh.rostralmiddlefrontal_3)
2663	0.90821	(rh.parstriangularis_4)(rh.rostralmiddlefrontal_3)(rh.rostralmiddlefrontal_8)
2664	0.90821	(rh.postcentral_1)(rh.postcentral_4)(rh.supramarginal_7)
2665	0.90821	(rh.precentral_1)(rh.precentral_4)(rh.precentral_6)
2666	0.90821	(rh.precentral_15)(rh.precentral_16)
2667	0.90821	(rh.precuneus_1)(rh.superiorparietal_10)(rh.superiorparietal_11)
2668	0.90821	(rh.precuneus_2)(rh.precuneus_4)(rh.superiorparietal_11)
2669	0.90821	(rh.precuneus_4)(rh.precuneus_5)(rh.superiorparietal_10)
2670	0.90821	(rh.rostralmiddlefrontal_1)(rh.rostralmiddlefrontal_3)(rh.rostralmiddlefrontal_5)
2671	0.90821	(rh.rostralmiddlefrontal_12)(rh.rostralmiddlefrontal_7)(rh.rostralmiddlefrontal_8)
2672	0.90821	(rh.rostralmiddlefrontal_5)(rh.rostralmiddlefrontal_8)(rh.superiorfrontal_2)
2673	0.90821	(rh.superiorfrontal_13)(rh.superiorfrontal_16)
2674	0.90821	(rh.superiorfrontal_13)(rh.superiorfrontal_7)(rh.superiorfrontal_9)
2675	0.90821	(rh.superiorfrontal_2)(rh.superiorfrontal_3)
2676	0.90821	(rh.superiorfrontal_3)(rh.superiorfrontal_4)(rh.superiorfrontal_6)
2677	0.90821	(rh.superiorfrontal_5)(rh.superiorfrontal_6)(rh.superiorfrontal_7)(rh.superiorfrontal_8)(rh.superiorfrontal_9)
2678	0.90821	(Right-Caudate)(Right-Putamen)(rh.rostralmiddlefrontal_5)
2679	0.90821	(Right-Hippocampus)(rh.bankssts_2)(rh.middletemporal_4)
2680	0.90821	(Right-Hippocampus)(rh.inferiorparietal_10)(rh.lingual_7)
2681	0.90821	(Right-Hippocampus)(rh.precuneus_3)(rh.precuneus_7)
2682	0.90821	(Right-Putamen)(rh.caudalmiddlefrontal_1)(rh.caudalmiddlefrontal_4)
2683	0.90821	(Right-Putamen)(rh.insula_1)(rh.postcentral_1)
2684	0.90821	(Right-Putamen)(rh.medialorbitofrontal_2)(rh.rostralmiddlefrontal_13)
2685	0.90821	(Right-Putamen)(rh.parstriangularis_1)(rh.rostralmiddlefrontal_3)(rh.rostralmiddlefrontal_8)
2686	0.90821	(Right-Putamen)(rh.superiorfrontal_11)
2687	0.9058	(Left-Accumbens-area)(Left-Caudate)(lh.medialorbitofrontal_5)
2688	0.9058	(Left-Caudate)(Left-Pallidum)(Left-Thalamus-Proper)(lh.precentral_4)
2689	0.9058	(Left-Caudate)(Left-Putamen)(lh.caudalanteriorcingulate_2)(lh.posteriorcingulate_1)(lh.posteriorcingulate_3)
2690	0.9058	(Left-Caudate)(Left-Thalamus-Proper)(lh.postcentral_8)
2691	0.9058	(Left-Caudate)(lh.caudalmiddlefrontal_3)(lh.rostralmiddlefrontal_1)(lh.rostralmiddlefrontal_4)
2692	0.9058	(Left-Caudate)(lh.lateralorbitofrontal_7)(lh.medialorbitofrontal_2)(lh.rostralmiddlefrontal_12)
2693	0.9058	(Left-Hippocampus)(Left-Pallidum)(Left-Putamen)(Left-Thalamus-Proper)(lh.insula_1)
2694	0.9058	(Left-Hippocampus)(Left-Pallidum)(Left-Putamen)(lh.insula_1)
2695	0.9058	(Left-Hippocampus)(Left-Pallidum)(Left-Thalamus-Proper)(lh.insula_1)
2696	0.9058	(Left-Hippocampus)(Left-Pallidum)(lh.insula_1)
2697	0.9058	(Left-Pallidum)(Left-Putamen)(Left-Thalamus-Proper)(lh.precentral_4)
2698	0.9058	(Left-Putamen)(lh.caudalanteriorcingulate_2)(lh.caudalmiddlefrontal_3)(lh.superiorfrontal_11)
2699	0.9058	(Left-Putamen)(lh.caudalanteriorcingulate_2)(lh.posteriorcingulate_1)(lh.posteriorcingulate_3)
2700	0.9058	(Left-Putamen)(lh.caudalmiddlefrontal_4)(lh.precentral_9)

2701	0.9058	(Left-Putamen)(lh.lateralorbitofrontal_4)(lh.parstriangularis_3)(lh.rostralmiddlefrontal_12)
2702	0.9058	(Left-Putamen)(lh.lateralorbitofrontal_7)(lh.medialorbitofrontal_2)(lh.rostralmiddlefrontal_12)(lh.rostralmiddlefrontal_9)
2703	0.9058	(Left-Putamen)(lh.lateralorbitofrontal_7)(lh.medialorbitofrontal_4)
2704	0.9058	(Left-Putamen)(lh.lateralorbitofrontal_7)(lh.rostralanteriorcingulate_1)(lh.rostralmiddlefrontal_12)
2705	0.9058	(Left-Putamen)(lh.rostralanteriorcingulate_1)(lh.rostralanteriorcingulate_2)(lh.superiorfrontal_4)
2706	0.9058	(Left-Putamen)(lh.rostralanteriorcingulate_1)(lh.rostralmiddlefrontal_4)
2707	0.9058	(Left-Putamen)(lh.rostralanteriorcingulate_2)(lh.rostralmiddlefrontal_9)
2708	0.9058	(Left-Putamen)(lh.rostralmiddlefrontal_9)(lh.superiorfrontal_3)
2709	0.9058	(lh.caudalanteriorcingulate_2)(lh.caudalmiddlefrontal_3)(lh.superiorfrontal_12)
2710	0.9058	(lh.caudalanteriorcingulate_2)(lh.superiorfrontal_11)(lh.superiorfrontal_4)(lh.superiorfrontal_8)
2711	0.9058	(lh.caudalmiddlefrontal_1)(lh.caudalmiddlefrontal_3)(lh.caudalmiddlefrontal_5)
2712	0.9058	(lh.fusiform_4)(lh.fusiform_5)(lh.inferiortemporal_8)
2713	0.9058	(lh.fusiform_7)(lh.inferiortemporal_3)(lh.inferiortemporal_6)
2714	0.9058	(lh.inferiorparietal_1)(lh.lateraloccipital_6)(lh.lateraloccipital_7)
2715	0.9058	(lh.inferiortemporal_2)(lh.inferiortemporal_3)
2716	0.9058	(lh.insula_3)(lh.postcentral_12)
2717	0.9058	(lh.lateralorbitofrontal_4)(lh.lateralorbitofrontal_5)
2718	0.9058	(lh.lateralorbitofrontal_4)(lh.lateralorbitofrontal_6)(lh.lateralorbitofrontal_7)(lh.rostralmiddlefrontal_12)
2719	0.9058	(lh.medialorbitofrontal_2)(lh.rostralmiddlefrontal_9)(lh.superiorfrontal_1)(lh.superiorfrontal_3)
2720	0.9058	(lh.paracentral_5)(lh.postcentral_4)
2721	0.9058	(lh.parstriangularis_2)(lh.parstriangularis_3)(lh.rostralmiddlefrontal_1)
2722	0.9058	(lh.postcentral_3)(lh.posteriorcingulate_4)
2723	0.9058	(lh.precentral_12)(lh.precentral_14)(lh.precentral_16)
2724	0.9058	(lh.rostralanteriorcingulate_1)(lh.rostralmiddlefrontal_4)(lh.rostralmiddlefrontal_9)
2725	0.9058	(lh.superiorfrontal_11)(lh.superiorfrontal_6)
2726	0.9058	(lh.superiorfrontal_14)(lh.superiorfrontal_7)(lh.superiorfrontal_8)(lh.superiorfrontal_9)
2727	0.9058	(lh.superiorfrontal_2)(lh.superiorfrontal_4)(lh.superiorfrontal_5)
2728	0.9058	(lh.supramarginal_10)(lh.supramarginal_9)
2729	0.9058	(rh.caudalanteriorcingulate_3)(rh.caudalmiddlefrontal_4)
2730	0.9058	(rh.caudalmiddlefrontal_2)(rh.caudalmiddlefrontal_3)(rh.caudalmiddlefrontal_4)
2731	0.9058	(rh.caudalmiddlefrontal_4)(rh.rostralmiddlefrontal_1)(rh.rostralmiddlefrontal_2)
2732	0.9058	(rh.cuneus_2)(rh.superiorparietal_11)
2733	0.9058	(rh.inferiorparietal_1)(rh.superiorparietal_11)
2734	0.9058	(rh.inferiorparietal_4)(rh.insula_2)
2735	0.9058	(rh.inferiortemporal_3)(rh.middletemporal_4)
2736	0.9058	(rh.insula_2)(rh.insula_5)
2737	0.9058	(rh.insula_6)(rh.insula_7)
2738	0.9058	(rh.lateraloccipital_4)(rh.lateraloccipital_5)(rh.lateraloccipital_7)
2739	0.9058	(rh.lateralorbitofrontal_7)(rh.medialorbitofrontal_3)
2740	0.9058	(rh.lateralorbitofrontal_7)(rh.rostralmiddlefrontal_8)
2741	0.9058	(rh.middletemporal_9)(rh.superiortemporal_7)
2742	0.9058	(rh.paracentral_3)(rh.precentral_14)
2743	0.9058	(rh.parsorbitalis_2)(rh.rostralmiddlefrontal_13)
2744	0.9058	(rh.precentral_7)(rh.precentral_8)
2745	0.9058	(rh.precuneus_1)(rh.precuneus_4)(rh.superiorparietal_10)
2746	0.9058	(rh.precuneus_10)(rh.precuneus_7)(rh.superiorparietal_7)
2747	0.9058	(rh.superiorfrontal_14)(rh.superiorfrontal_16)(rh.superiorfrontal_17)
2748	0.9058	(rh.superiorfrontal_2)(rh.superiorfrontal_5)(rh.superiorfrontal_8)
2749	0.9058	(rh.superiorfrontal_4)(rh.superiorfrontal_7)
2750	0.9058	(rh.superiorparietal_1)(rh.supramarginal_1)(rh.supramarginal_8)(rh.supramarginal_9)

2751	0.9058	(Right-Accumbens-area)(Right-Hippocampus)(Right-Pallidum)(Right-Putamen)(Right-Thalamus-Proper)
2752	0.9058	(Right-Accumbens-area)(Right-Hippocampus)(Right-Pallidum)(Right-Putamen)
2753	0.9058	(Right-Accumbens-area)(Right-Hippocampus)(Right-Putamen)(Right-Thalamus-Proper)
2754	0.9058	(Right-Accumbens-area)(Right-Hippocampus)(Right-Putamen)
2755	0.9058	(Right-Amygdala)(Right-Hippocampus)(rh.inferiortemporal_1)
2756	0.9058	(Right-Caudate)(rh.caudalanteriorcingulate_3)(rh.caudalmiddlefrontal_4)
2757	0.9058	(Right-Caudate)(rh.caudalmiddlefrontal_4)(rh.precentral_7)
2758	0.9058	(Right-Caudate)(rh.medialorbitofrontal_4)
2759	0.9058	(Right-Caudate)(rh.superiorfrontal_9)
2760	0.9058	(Right-Caudate)(Right-Putamen)(rh.caudalmiddlefrontal_4)(rh.precentral_7)
2761	0.9058	(Right-Caudate)(Right-Thalamus-Proper)(rh.precentral_11)
2762	0.9058	(Right-Hippocampus)(rh.fusiform_5)
2763	0.9058	(Right-Hippocampus)(rh.inferiorparietal_4)(rh.supramarginal_9)
2764	0.9058	(Right-Putamen)(rh.insula_2)(rh.insula_5)
2765	0.9058	(Right-Putamen)(rh.insula_6)(rh.insula_7)
2766	0.9058	(Right-Putamen)(rh.lateralorbitofrontal_7)(rh.parstriangularis_1)(rh.rostralmiddlefrontal_12)
2767	0.9058	(Right-Putamen)(rh.precentral_15)
2768	0.90338	(Left-Accumbens-area)(Left-Caudate)(Right-Caudate)
2769	0.90338	(Left-Amygdala)(lh.inferiortemporal_1)
2770	0.90338	(Left-Caudate)(Left-Pallidum)(Left-Putamen)(Left-Thalamus-Proper)(lh.precentral_4)
2771	0.90338	(Left-Caudate)(Left-Pallidum)(Left-Putamen)(lh.caudalmiddlefrontal_3)
2772	0.90338	(Left-Caudate)(Left-Pallidum)(lh.caudalmiddlefrontal_3)
2773	0.90338	(Left-Caudate)(Left-Putamen)(Left-Thalamus-Proper)(lh.postcentral_8)
2774	0.90338	(Left-Caudate)(Left-Putamen)(lh.caudalanteriorcingulate_2)(lh.caudalmiddlefrontal_3)(lh.superiorfrontal_11)
2775	0.90338	(Left-Caudate)(Left-Putamen)(lh.parstriangularis_3)(lh.rostralmiddlefrontal_1)(lh.rostralmiddlefrontal_9)
2776	0.90338	(Left-Caudate)(lh.caudalanteriorcingulate_2)(lh.caudalmiddlefrontal_3)(lh.superiorfrontal_12)
2777	0.90338	(Left-Caudate)(lh.insula_6)(lh.parsopercularis_3)(lh.rostralmiddlefrontal_1)
2778	0.90338	(Left-Caudate)(lh.lateralorbitofrontal_6)(lh.rostralmiddlefrontal_9)
2779	0.90338	(Left-Caudate)(lh.lateralorbitofrontal_7)(lh.medialorbitofrontal_2)(lh.rostralmiddlefrontal_9)
2780	0.90338	(Left-Caudate)(lh.parsopercularis_3)(lh.parstriangularis_3)
2781	0.90338	(Left-Caudate)(lh.postcentral_8)(lh.precentral_7)
2782	0.90338	(Left-Caudate)(lh.precentral_4)(lh.superiorfrontal_14)
2783	0.90338	(Left-Caudate)(lh.superiorfrontal_10)(lh.superiorfrontal_4)(lh.superiorfrontal_8)
2784	0.90338	(Left-Caudate)(Right-Accumbens-area)
2785	0.90338	(Left-Hippocampus)(lh.bankssts_3)(lh.inferiorparietal_5)
2786	0.90338	(Left-Hippocampus)(lh.fusiform_5)(lh.fusiform_7)
2787	0.90338	(Left-Hippocampus)(lh.lateraloccipital_9)
2788	0.90338	(Left-Pallidum)(Left-Putamen)(lh.caudalmiddlefrontal_3)
2789	0.90338	(Left-Pallidum)(lh.caudalmiddlefrontal_3)
2790	0.90338	(Left-Putamen)(Left-Thalamus-Proper)(lh.insula_1)(lh.precentral_7)
2791	0.90338	(Left-Putamen)(lh.insula_6)(lh.rostralmiddlefrontal_9)
2792	0.90338	(Left-Putamen)(lh.lateralorbitofrontal_2)(lh.medialorbitofrontal_2)
2793	0.90338	(Left-Putamen)(lh.lateralorbitofrontal_6)(lh.rostralmiddlefrontal_1)(lh.rostralmiddlefrontal_9)
2794	0.90338	(Left-Putamen)(lh.lateralorbitofrontal_7)(lh.medialorbitofrontal_2)(lh.rostralanteriorcingulate_1)
2795	0.90338	(Left-Putamen)(lh.parsopercularis_1)(lh.parsopercularis_3)(lh.rostralmiddlefrontal_1)
2796	0.90338	(Left-Putamen)(lh.parstriangularis_3)(lh.rostralanteriorcingulate_1)(lh.rostralmiddlefrontal_12)
2797	0.90338	(Left-Thalamus-Proper)(lh.insula_1)(lh.precentral_7)
2798	0.90338	(Left-Thalamus-Proper)(lh.precentral_11)(lh.precentral_4)(lh.precentral_7)
2799	0.90338	(lh.bankssts_2)(lh.inferiortemporal_7)
2800	0.90338	(lh.fusiform_2)(lh.lateraloccipital_6)(lh.lateraloccipital_7)(lh.pericalcarine_3)

2801	0.90338	(lh.fusiform_2)(lh.lateraloccipital_7)(lh.lingual_8)
2802	0.90338	(lh.fusiform_2)(lh.lingual_6)(lh.lingual_8)
2803	0.90338	(lh.inferiortemporal_3)(lh.middletemporal_4)(lh.middletemporal_5)
2804	0.90338	(lh.lateraloccipital_11)(lh.lateraloccipital_6)(lh.pericalcarine_2)
2805	0.90338	(lh.lateraloccipital_11)(lh.pericalcarine_3)
2806	0.90338	(lh.lateraloccipital_6)(lh.lingual_1)(lh.lingual_4)
2807	0.90338	(lh.lateralorbitofrontal_2)(lh.lateralorbitofrontal_7)(lh.medialorbitofrontal_2)
2808	0.90338	(lh.lateralorbitofrontal_3)(lh.lateralorbitofrontal_6)
2809	0.90338	(lh.lateralorbitofrontal_4)(lh.lateralorbitofrontal_6)(lh.parstriangularis_3)(lh.rostralmiddlefrontal_12)(lh.rostralmiddlefrontal_9)
2810	0.90338	(lh.lateralorbitofrontal_4)(lh.lateralorbitofrontal_7)(lh.rostralmiddlefrontal_9)
2811	0.90338	(lh.lateralorbitofrontal_6)(lh.rostralmiddlefrontal_1)(lh.rostralmiddlefrontal_6)(lh.rostralmiddlefrontal_9)
2812	0.90338	(lh.lateralorbitofrontal_7)(lh.medialorbitofrontal_2)(lh.rostralanteriorcingulate_1)(lh.rostralmiddlefrontal_12)
2813	0.90338	(lh.lateralorbitofrontal_7)(lh.rostralanteriorcingulate_1)(lh.rostralmiddlefrontal_9)
2814	0.90338	(lh.medialorbitofrontal_4)(lh.rostralanteriorcingulate_1)
2815	0.90338	(lh.parstriangularis_3)(lh.rostralanteriorcingulate_2)
2816	0.90338	(lh.precuneus_10)(lh.precuneus_6)(lh.precuneus_8)(lh.precuneus_9)
2817	0.90338	(lh.rostralanteriorcingulate_2)(lh.superiorfrontal_10)(lh.superiorfrontal_4)
2818	0.90338	(lh.rostralmiddlefrontal_1)(lh.rostralmiddlefrontal_7)(lh.rostralmiddlefrontal_9)
2819	0.90338	(lh.rostralmiddlefrontal_4)(lh.superiorfrontal_10)(lh.superiorfrontal_11)
2820	0.90338	(lh.superiorfrontal_11)(lh.superiorfrontal_14)(lh.superiorfrontal_7)
2821	0.90338	(lh.superiorfrontal_11)(lh.superiorfrontal_4)(lh.superiorfrontal_6)
2822	0.90338	(lh.superiorfrontal_2)(lh.superiorfrontal_3)(lh.superiorfrontal_4)(lh.superiorfrontal_5)
2823	0.90338	(rh.bankssts_2)(rh.bankssts_3)(rh.superiortemporal_3)
2824	0.90338	(rh.fusiform_1)(rh.lateraloccipital_10)(rh.lateraloccipital_7)(rh.lateraloccipital_8)
2825	0.90338	(rh.fusiform_1)(rh.lateraloccipital_8)(rh.pericalcarine_1)
2826	0.90338	(rh.inferiortemporal_4)(rh.middletemporal_4)(rh.middletemporal_7)
2827	0.90338	(rh.inferiortemporal_7)(rh.middletemporal_1)(rh.middletemporal_2)
2828	0.90338	(rh.isthmuscingulate_2)(rh.precuneus_3)(rh.precuneus_4)(rh.precuneus_7)
2829	0.90338	(rh.lateraloccipital_6)(rh.lateraloccipital_7)
2830	0.90338	(rh.lateralorbitofrontal_2)(rh.lateralorbitofrontal_7)(rh.parstriangularis_1)
2831	0.90338	(rh.middletemporal_7)(rh.middletemporal_8)
2832	0.90338	(rh.paracentral_3)(rh.paracentral_4)(rh.precentral_15)
2833	0.90338	(rh.parsopercularis_4)(rh.parstriangularis_2)(rh.rostralmiddlefrontal_3)
2834	0.90338	(rh.parsorbitalis_2)(rh.parstriangularis_1)
2835	0.90338	(rh.precentral_7)(rh.supramarginal_1)
2836	0.90338	(rh.precuneus_2)(rh.precuneus_7)
2837	0.90338	(rh.precuneus_4)(rh.precuneus_7)(rh.superiorparietal_1)
2838	0.90338	(rh.rostralanteriorcingulate_1)(rh.rostralanteriorcingulate_2)
2839	0.90338	(rh.rostralmiddlefrontal_11)(rh.rostralmiddlefrontal_12)
2840	0.90338	(rh.rostralmiddlefrontal_8)(rh.rostralmiddlefrontal_9)(rh.superiorfrontal_2)
2841	0.90338	(rh.superiorfrontal_1)(rh.superiorfrontal_2)(rh.superiorfrontal_4)
2842	0.90338	(rh.supramarginal_1)(rh.supramarginal_6)
2843	0.90338	(Right-Accumbens-area)(Right-Amygdala)(Right-Pallidum)(Right-Putamen)(Right-Thalamus-Proper)
2844	0.90338	(Right-Accumbens-area)(Right-Amygdala)(Right-Pallidum)(Right-Putamen)
2845	0.90338	(Right-Accumbens-area)(Right-Amygdala)(Right-Putamen)(Right-Thalamus-Proper)
2846	0.90338	(Right-Accumbens-area)(Right-Amygdala)(Right-Putamen)
2847	0.90338	(Right-Caudate)(rh.parsopercularis_4)(rh.rostralmiddlefrontal_3)
2848	0.90338	(Right-Caudate)(rh.rostralanteriorcingulate_1)(rh.rostralanteriorcingulate_2)
2849	0.90338	(Right-Hippocampus)(rh.bankssts_1)(rh.bankssts_2)
2850	0.90338	(Right-Hippocampus)(rh.inferiorparietal_10)(rh.insula_2)

2851	0.90338	(Right-Hippocampus)(rh.isthmuscingulate_2)(rh.precuneus_4)
2852	0.90338	(Right-Putamen)(rh.insula_1)(rh.precentral_11)(rh.precentral_7)
2853	0.90338	(Right-Putamen)(rh.insula_7)(rh.rostralmiddlefrontal_3)
2854	0.90338	(Right-Putamen)(rh.lateralorbitofrontal_6)(rh.lateralorbitofrontal_7)(rh.medialorbitofrontal_2)
2855	0.90338	(Right-Putamen)(rh.rostralmiddlefrontal_9)
2856	0.90338	(Right-Putamen)(rh.superiorfrontal_2)(rh.superiorfrontal_7)
2857	0.90338	(Right-Putamen)(Right-Thalamus-Proper)(rh.insula_1)(rh.insula_2)(rh.supramarginal_9)
2858	0.90338	(Right-Thalamus-Proper)(rh.insula_1)(rh.insula_2)(rh.supramarginal_9)
2859	0.90338	(Right-Thalamus-Proper)(rh.isthmuscingulate_1)
2860	0.90097	(Left-Caudate)(Left-Putamen)(lh.caudalanteriorcingulate_2)(lh.superiorfrontal_14)
2861	0.90097	(Left-Caudate)(Left-Putamen)(lh.caudalmiddlefrontal_3)(lh.caudalmiddlefrontal_4)(lh.caudalmiddlefrontal_5)
2862	0.90097	(Left-Caudate)(Left-Putamen)(lh.parstriangularis_3)(lh.rostralanteriorcingulate_1)(lh.rostralmiddlefrontal_9)
2863	0.90097	(Left-Caudate)(Left-Putamen)(lh.rostralanteriorcingulate_1)(lh.rostralmiddlefrontal_4)
2864	0.90097	(Left-Caudate)(Left-Putamen)(lh.supramarginal_1)
2865	0.90097	(Left-Caudate)(lh.lateralorbitofrontal_2)(lh.medialorbitofrontal_2)
2866	0.90097	(Left-Caudate)(lh.lateralorbitofrontal_4)(lh.rostralmiddlefrontal_9)
2867	0.90097	(Left-Caudate)(lh.lateralorbitofrontal_7)(lh.medialorbitofrontal_2)(lh.rostralanteriorcingulate_1)
2868	0.90097	(Left-Caudate)(lh.medialorbitofrontal_4)(lh.rostralanteriorcingulate_1)
2869	0.90097	(Left-Caudate)(lh.parstriangularis_3)(lh.rostralmiddlefrontal_12)
2870	0.90097	(Left-Caudate)(lh.rostralanteriorcingulate_1)(lh.rostralanteriorcingulate_2)(lh.rostralmiddlefrontal_9)
2871	0.90097	(Left-Hippocampus)(Left-Putamen)(lh.insula_1)(lh.insula_2)
2872	0.90097	(Left-Hippocampus)(lh.bankssts_3)(lh.fusiform_5)
2873	0.90097	(Left-Hippocampus)(lh.insula_1)(lh.insula_2)
2874	0.90097	(Left-Putamen)(Left-Thalamus-Proper)(lh.insula_1)(lh.insula_2)
2875	0.90097	(Left-Putamen)(Left-Thalamus-Proper)(lh.posteriorcingulate_3)(lh.precentral_4)
2876	0.90097	(Left-Putamen)(Left-Thalamus-Proper)(lh.precentral_11)(lh.precentral_4)(lh.precentral_7)
2877	0.90097	(Left-Putamen)(lh.insula_6)(lh.parsopercularis_1)(lh.rostralmiddlefrontal_1)
2878	0.90097	(Left-Putamen)(lh.lateralorbitofrontal_4)(lh.lateralorbitofrontal_6)(lh.lateralorbitofrontal_7)(lh.parstriangularis_3)
2879	0.90097	(Left-Putamen)(lh.lateralorbitofrontal_4)(lh.lateralorbitofrontal_7)(lh.rostralmiddlefrontal_9)
2880	0.90097	(Left-Putamen)(lh.lateralorbitofrontal_6)(lh.lateralorbitofrontal_7)(lh.parstriangularis_3)(lh.rostralmiddlefrontal_12)
2881	0.90097	(Left-Putamen)(lh.lateralorbitofrontal_6)(lh.lateralorbitofrontal_7)(lh.rostralmiddlefrontal_12)(lh.rostralmiddlefrontal_9)
2882	0.90097	(Left-Putamen)(lh.parsopercularis_1)(lh.parsopercularis_3)(lh.parstriangularis_3)
2883	0.90097	(Left-Putamen)(lh.parstriangularis_3)(lh.rostralanteriorcingulate_1)(lh.rostralmiddlefrontal_12)(lh.rostralmiddlefrontal_9)
2884	0.90097	(Left-Thalamus-Proper)(lh.insula_1)(lh.insula_2)
2885	0.90097	(Left-Thalamus-Proper)(lh.paracentral_5)(lh.precentral_4)
2886	0.90097	(Left-Thalamus-Proper)(lh.precentral_11)(lh.precentral_9)
2887	0.90097	(lh.bankssts_2)(lh.inferiortemporal_8)
2888	0.90097	(lh.caudalanteriorcingulate_2)(lh.rostralanteriorcingulate_2)(lh.superiorfrontal_11)
2889	0.90097	(lh.caudalanteriorcingulate_2)(lh.rostralanteriorcingulate_2)(lh.superiorfrontal_4)
2890	0.90097	(lh.caudalmiddlefrontal_4)(lh.precentral_11)(lh.precentral_9)
2891	0.90097	(lh.cuneus_1)(lh.lateraloccipital_2)
2892	0.90097	(lh.inferiorparietal_4)(lh.lingual_8)
2893	0.90097	(lh.inferiorparietal_5)(lh.inferiorparietal_6)(lh.inferiorparietal_9)
2894	0.90097	(lh.inferiorparietal_5)(lh.supramarginal_7)
2895	0.90097	(lh.insula_1)(lh.postcentral_14)(lh.supramarginal_2)
2896	0.90097	(lh.insula_1)(lh.postcentral_8)(lh.supramarginal_9)
2897	0.90097	(lh.insula_3)(lh.precentral_16)
2898	0.90097	(lh.insula_6)(lh.rostralmiddlefrontal_1)(lh.rostralmiddlefrontal_9)
2899	0.90097	(lh.lateraloccipital_1)(lh.lateraloccipital_2)(lh.lateraloccipital_3)(lh.pericalcarine_2)
2900	0.90097	(lh.lateraloccipital_11)(lh.lateraloccipital_3)(lh.pericalcarine_2)

2901	0.90097	(lh.lateraloccipital_6)(lh.lingual_8)
2902	0.90097	(lh.lateralorbitofrontal_6)(lh.lateralorbitofrontal_7)(lh.parstriangularis_3)(lh.rostralmiddlefrontal_12)(lh.rostralmiddlefrontal_9)
2903	0.90097	(lh.lateralorbitofrontal_6)(lh.parstriangularis_3)(lh.rostralmiddlefrontal_12)(lh.rostralmiddlefrontal_6)(lh.rostralmiddlefrontal_9)
2904	0.90097	(lh.lateralorbitofrontal_7)(lh.medialorbitofrontal_1)(lh.medialorbitofrontal_2)
2905	0.90097	(lh.lateralorbitofrontal_7)(lh.parstriangularis_3)(lh.rostralanteriorcingulate_1)
2906	0.90097	(lh.lingual_1)(lh.lingual_8)
2907	0.90097	(lh.lingual_3)(lh.pericalcarine_2)
2908	0.90097	(lh.lingual_4)(lh.lingual_5)
2909	0.90097	(lh.middletemporal_7)(lh.superiortemporal_11)
2910	0.90097	(lh.parstriangularis_2)(lh.rostralmiddlefrontal_9)
2911	0.90097	(lh.precentral_11)(lh.precentral_8)
2912	0.90097	(lh.precuneus_5)(lh.precuneus_6)
2913	0.90097	(lh.precuneus_6)(lh.precuneus_9)(lh.superiorparietal_6)
2914	0.90097	(lh.rostralmiddlefrontal_4)(lh.rostralmiddlefrontal_8)(lh.rostralmiddlefrontal_9)
2915	0.90097	(lh.rostralmiddlefrontal_8)(lh.superiorfrontal_2)(lh.superiorfrontal_4)
2916	0.90097	(lh.superiorfrontal_10)(lh.superiorfrontal_4)(lh.superiorfrontal_6)(lh.superiorfrontal_8)
2917	0.90097	(lh.superiorfrontal_10)(lh.superiorfrontal_6)(lh.superiorfrontal_8)
2918	0.90097	(lh.superiorfrontal_10)(lh.superiorfrontal_8)(lh.superiorfrontal_9)
2919	0.90097	(lh.superiorfrontal_11)(lh.superiorfrontal_14)(lh.superiorfrontal_4)(lh.superiorfrontal_8)
2920	0.90097	(lh.superiorfrontal_11)(lh.superiorfrontal_14)(lh.superiorfrontal_4)
2921	0.90097	(lh.superiorfrontal_11)(lh.superiorfrontal_14)(lh.superiorfrontal_7)(lh.superiorfrontal_8)
2922	0.90097	(lh.supramarginal_1)(lh.supramarginal_2)(lh.supramarginal_4)
2923	0.90097	(lh.supramarginal_5)(lh.supramarginal_9)
2924	0.90097	(rh.bankssts_2)(rh.middletemporal_1)
2925	0.90097	(rh.caudalanteriorcingulate_3)(rh.posteriorcingulate_2)
2926	0.90097	(rh.caudalmiddlefrontal_1)(rh.caudalmiddlefrontal_3)(rh.caudalmiddlefrontal_4)
2927	0.90097	(rh.caudalmiddlefrontal_4)(rh.superiorfrontal_11)(rh.superiorfrontal_9)
2928	0.90097	(rh.cuneus_3)(rh.lateraloccipital_1)
2929	0.90097	(rh.insula_1)(rh.insula_2)(rh.insula_3)(rh.precentral_7)
2930	0.90097	(rh.lateraloccipital_1)(rh.lateraloccipital_3)(rh.pericalcarine_4)
2931	0.90097	(rh.lateraloccipital_7)(rh.pericalcarine_4)
2932	0.90097	(rh.lateralorbitofrontal_2)(rh.lateralorbitofrontal_6)(rh.lateralorbitofrontal_7)(rh.parstriangularis_1)
2933	0.90097	(rh.lateralorbitofrontal_7)(rh.medialorbitofrontal_2)(rh.superiorfrontal_2)
2934	0.90097	(rh.lateralorbitofrontal_7)(rh.rostralmiddlefrontal_12)(rh.rostralmiddlefrontal_8)
2935	0.90097	(rh.postcentral_1)(rh.supramarginal_7)(rh.supramarginal_9)
2936	0.90097	(rh.postcentral_2)(rh.postcentral_4)
2937	0.90097	(rh.precentral_13)(rh.superiorparietal_1)
2938	0.90097	(rh.rostralmiddlefrontal_9)(rh.superiorfrontal_2)(rh.superiorfrontal_5)
2939	0.90097	(rh.superiorfrontal_11)(rh.superiorfrontal_5)(rh.superiorfrontal_7)(rh.superiorfrontal_8)
2940	0.90097	(rh.superiorfrontal_11)(rh.superiorfrontal_5)(rh.superiorfrontal_7)
2941	0.90097	(Right-Accumbens-area)(Right-Caudate)(Right-Hippocampus)(Right-Pallidum)(Right-Thalamus-Proper)
2942	0.90097	(Right-Accumbens-area)(Right-Caudate)(Right-Hippocampus)(Right-Pallidum)
2943	0.90097	(Right-Accumbens-area)(Right-Caudate)(Right-Hippocampus)(Right-Thalamus-Proper)
2944	0.90097	(Right-Accumbens-area)(Right-Caudate)(Right-Hippocampus)
2945	0.90097	(Right-Caudate)(rh.caudalanteriorcingulate_3)(rh.posteriorcingulate_2)
2946	0.90097	(Right-Caudate)(rh.precentral_11)(rh.precentral_13)(rh.precentral_7)
2947	0.90097	(Right-Caudate)(rh.precentral_11)(rh.precentral_7)
2948	0.90097	(Right-Caudate)(rh.rostralmiddlefrontal_1)
2949	0.90097	(Right-Caudate)(Right-Hippocampus)(Right-Thalamus-Proper)(rh.supramarginal_9)
2950	0.90097	(Right-Caudate)(Right-Putamen)(rh.caudalmiddlefrontal_1)

2951	0.90097	(Right-Caudate)(Right-Putamen)(Right-Thalamus-Proper)(rh.posteriorcingulate_2)
2952	0.90097	(Right-Caudate)(Right-Thalamus-Proper)(rh.insula_1)(rh.supramarginal_9)
2953	0.90097	(Right-Hippocampus)(rh.fusiform_8)(rh.insula_5)
2954	0.90097	(Right-Hippocampus)(rh.insula_2)(rh.insula_5)
2955	0.90097	(Right-Hippocampus)(rh.supramarginal_8)
2956	0.90097	(Right-Hippocampus)(Right-Putamen)(rh.insula_2)(rh.insula_5)
2957	0.90097	(Right-Putamen)(rh.insula_1)(rh.insula_2)(rh.insula_3)(rh.precentral_7)
2958	0.90097	(Right-Putamen)(rh.lateralorbitofrontal_6)(rh.lateralorbitofrontal_7)(rh.rostralmiddlefrontal_13)
2959	0.90097	(Right-Putamen)(rh.lateralorbitofrontal_7)(rh.medialorbitofrontal_2)(rh.rostralmiddlefrontal_13)
2960	0.90097	(Right-Putamen)(rh.lateralorbitofrontal_7)(rh.superiorfrontal_2)
2961	0.90097	(Right-Putamen)(rh.postcentral_1)(rh.precentral_1)
2962	0.90097	(Right-Putamen)(rh.precentral_1)(rh.precentral_6)
2963	0.90097	(Right-Putamen)(rh.rostralmiddlefrontal_11)
2964	0.90097	(Right-Putamen)(rh.superiorparietal_1)(rh.supramarginal_1)(rh.supramarginal_9)
2965	0.90097	(Right-Putamen)(Right-Thalamus-Proper)(rh.posteriorcingulate_2)
2966	0.89855	(Left-Amygdala)(Left-Hippocampus)(Left-Putamen)(lh.insula_2)
2967	0.89855	(Left-Amygdala)(Left-Hippocampus)(lh.insula_2)
2968	0.89855	(Left-Amygdala)(Left-Putamen)(lh.insula_2)
2969	0.89855	(Left-Amygdala)(lh.insula_2)
2970	0.89855	(Left-Caudate)(Left-Putamen)(Left-Thalamus-Proper)(lh.posteriorcingulate_3)(lh.precentral_4)
2971	0.89855	(Left-Caudate)(Left-Putamen)(lh.lateralorbitofrontal_4)(lh.rostralmiddlefrontal_9)
2972	0.89855	(Left-Caudate)(Left-Putamen)(lh.lateralorbitofrontal_7)(lh.parstriangularis_3)
2973	0.89855	(Left-Caudate)(lh.caudalanteriorcingulate_2)(lh.superiorfrontal_10)(lh.superiorfrontal_11)
2974	0.89855	(Left-Caudate)(lh.medialorbitofrontal_2)(lh.superiorfrontal_4)
2975	0.89855	(Left-Caudate)(lh.parstriangularis_3)(lh.rostralmiddlefrontal_1)(lh.rostralmiddlefrontal_4)(lh.rostralmiddlefrontal_9)
2976	0.89855	(Left-Caudate)(lh.rostralanteriorcingulate_1)(lh.rostralmiddlefrontal_9)(lh.superiorfrontal_4)
2977	0.89855	(Left-Caudate)(lh.rostralanteriorcingulate_2)(lh.superiorfrontal_11)
2978	0.89855	(Left-Putamen)(Left-Thalamus-Proper)(lh.precentral_11)(lh.precentral_9)
2979	0.89855	(Left-Putamen)(lh.caudalmiddlefrontal_1)(lh.caudalmiddlefrontal_5)
2980	0.89855	(Left-Putamen)(lh.caudalmiddlefrontal_4)(lh.precentral_11)(lh.precentral_9)
2981	0.89855	(Left-Putamen)(lh.lateralorbitofrontal_3)(lh.lateralorbitofrontal_6)
2982	0.89855	(Left-Putamen)(lh.lateralorbitofrontal_4)(lh.lateralorbitofrontal_6)(lh.lateralorbitofrontal_7)(lh.rostralmiddlefrontal_12)
2983	0.89855	(Left-Putamen)(lh.lateralorbitofrontal_6)(lh.parstriangularis_3)(lh.rostralmiddlefrontal_1)
2984	0.89855	(Left-Putamen)(lh.medialorbitofrontal_2)(lh.superiorfrontal_3)
2985	0.89855	(Left-Putamen)(lh.rostralanteriorcingulate_1)(lh.rostralmiddlefrontal_4)(lh.rostralmiddlefrontal_9)
2986	0.89855	(Left-Putamen)(lh.rostralmiddlefrontal_4)(lh.superiorfrontal_11)
2987	0.89855	(Left-Putamen)(lh.superiorfrontal_10)
2988	0.89855	(lh.caudalmiddlefrontal_1)(lh.superiorfrontal_11)
2989	0.89855	(lh.cuneus_1)(lh.cuneus_2)(lh.pericalcarine_1)
2990	0.89855	(lh.fusiform_1)(lh.fusiform_4)(lh.fusiform_5)
2991	0.89855	(lh.fusiform_2)(lh.lateraloccipital_6)(lh.lingual_8)
2992	0.89855	(lh.fusiform_5)(lh.fusiform_6)(lh.fusiform_7)
2993	0.89855	(lh.insula_2)(lh.precentral_9)
2994	0.89855	(lh.insula_6)(lh.parsopercularis_3)(lh.parstriangularis_3)(lh.rostralmiddlefrontal_1)
2995	0.89855	(lh.lateraloccipital_3)(lh.lateraloccipital_6)(lh.pericalcarine_3)
2996	0.89855	(lh.lateraloccipital_3)(lh.lingual_1)(lh.pericalcarine_3)
2997	0.89855	(lh.lateralorbitofrontal_2)(lh.medialorbitofrontal_5)
2998	0.89855	(lh.lateralorbitofrontal_4)(lh.lateralorbitofrontal_7)(lh.parstriangularis_3)(lh.rostralmiddlefrontal_12)
2999	0.89855	(lh.lingual_6)(lh.pericalcarine_3)(lh.precuneus_11)
3000	0.89855	(lh.paracentral_4)(lh.superiorfrontal_18)

3001	0.89855	(lh.paracentral_5)(lh.precentral_2)(lh.precentral_3)
3002	0.89855	(lh.parsopercularis_1)(lh.precentral_14)
3003	0.89855	(lh.parsopercularis_1)(lh.precentral_16)
3004	0.89855	(lh.parstriangularis_2)(lh.parstriangularis_3)(lh.rostralmiddlefrontal_9)
3005	0.89855	(lh.postcentral_11)(lh.postcentral_14)
3006	0.89855	(lh.postcentral_12)(lh.precentral_14)
3007	0.89855	(lh.postcentral_3)(lh.supramarginal_9)
3008	0.89855	(lh.posteriorcingulate_4)(lh.precuneus_3)
3009	0.89855	(lh.precentral_11)(lh.precentral_7)(lh.precentral_9)
3010	0.89855	(lh.precuneus_6)(lh.precuneus_8)(lh.precuneus_9)(lh.superiorparietal_11)
3011	0.89855	(lh.rostralanteriorcingulate_1)(lh.rostralmiddlefrontal_4)(lh.superiorfrontal_4)
3012	0.89855	(lh.rostralanteriorcingulate_1)(lh.rostralmiddlefrontal_9)(lh.superiorfrontal_3)(lh.superiorfrontal_4)
3013	0.89855	(lh.rostralanteriorcingulate_2)(lh.superiorfrontal_11)(lh.superiorfrontal_4)
3014	0.89855	(lh.rostralmiddlefrontal_4)(lh.superiorfrontal_3)(lh.superiorfrontal_4)
3015	0.89855	(lh.superiorfrontal_10)(lh.superiorfrontal_7)(lh.superiorfrontal_8)(lh.superiorfrontal_9)
3016	0.89855	(lh.supramarginal_2)(lh.supramarginal_3)
3017	0.89855	(rh.bankssts_2)(rh.inferiorparietal_9)
3018	0.89855	(rh.fusiform_2)(rh.lateraloccipital_10)(rh.lingual_5)
3019	0.89855	(rh.inferiorparietal_10)(rh.inferiorparietal_11)(rh.lateraloccipital_8)
3020	0.89855	(rh.inferiorparietal_4)(rh.inferiorparietal_6)
3021	0.89855	(rh.inferiorparietal_4)(rh.supramarginal_8)(rh.supramarginal_9)
3022	0.89855	(rh.inferiortemporal_2)(rh.middletemporal_4)(rh.middletemporal_7)
3023	0.89855	(rh.inferiortemporal_6)(rh.inferiortemporal_7)(rh.middletemporal_2)
3024	0.89855	(rh.isthmuscingulate_2)(rh.lingual_7)(rh.precuneus_2)
3025	0.89855	(rh.lateraloccipital_3)(rh.lateraloccipital_8)
3026	0.89855	(rh.lateralorbitofrontal_2)(rh.lateralorbitofrontal_4)(rh.lateralorbitofrontal_6)
3027	0.89855	(rh.paracentral_6)(rh.precuneus_10)
3028	0.89855	(rh.parahippocampal_2)(rh.parahippocampal_3)
3029	0.89855	(rh.parstriangularis_1)(rh.parstriangularis_2)(rh.parstriangularis_3)
3030	0.89855	(rh.parstriangularis_1)(rh.parstriangularis_2)(rh.parstriangularis_4)(rh.rostralmiddlefrontal_3)
3031	0.89855	(rh.pericalcarine_1)(rh.pericalcarine_3)
3032	0.89855	(rh.precentral_10)(rh.precentral_6)
3033	0.89855	(rh.precuneus_4)(rh.precuneus_5)(rh.precuneus_7)
3034	0.89855	(rh.rostralmiddlefrontal_3)(rh.rostralmiddlefrontal_5)(rh.rostralmiddlefrontal_6)(rh.rostralmiddlefrontal_8)
3035	0.89855	(rh.superiorfrontal_10)(rh.superiorfrontal_13)(rh.superiorfrontal_9)
3036	0.89855	(rh.superiorfrontal_4)(rh.superiorfrontal_5)(rh.superiorfrontal_7)
3037	0.89855	(Right-Amygdala)(rh.insula_4)
3038	0.89855	(Right-Amygdala)(Right-Caudate)(Right-Pallidum)(Right-Putamen)(Right-Thalamus-Proper)
3039	0.89855	(Right-Amygdala)(Right-Caudate)(Right-Pallidum)(Right-Putamen)
3040	0.89855	(Right-Amygdala)(Right-Caudate)(Right-Pallidum)(Right-Thalamus-Proper)
3041	0.89855	(Right-Amygdala)(Right-Caudate)(Right-Pallidum)
3042	0.89855	(Right-Amygdala)(Right-Caudate)(Right-Putamen)(Right-Thalamus-Proper)
3043	0.89855	(Right-Amygdala)(Right-Caudate)(Right-Putamen)
3044	0.89855	(Right-Amygdala)(Right-Caudate)(Right-Thalamus-Proper)
3045	0.89855	(Right-Amygdala)(Right-Caudate)
3046	0.89855	(Right-Amygdala)(Right-Putamen)(rh.insula_4)
3047	0.89855	(Right-Caudate)(rh.caudalmiddlefrontal_4)(rh.insula_7)
3048	0.89855	(Right-Caudate)(rh.caudalmiddlefrontal_4)(rh.superiorfrontal_11)(rh.superiorfrontal_13)
3049	0.89855	(Right-Caudate)(rh.lateralorbitofrontal_4)(rh.lateralorbitofrontal_5)
3050	0.89855	(Right-Caudate)(rh.superiorfrontal_2)(rh.superiorfrontal_7)

3051	0.89855	(Right-Caudate)(Right-Hippocampus)(Right-Putamen)(Right-Thalamus-Proper)(rh.supramarginal_9)
3052	0.89855	(Right-Caudate)(Right-Putamen)(rh.caudalmiddlefrontal_4)(rh.insula_7)
3053	0.89855	(Right-Caudate)(Right-Putamen)(Right-Thalamus-Proper)(rh.insula_1)(rh.supramarginal_9)
3054	0.89855	(Right-Hippocampus)(rh.middletemporal_1)
3055	0.89855	(Right-Hippocampus)(rh.superiortemporal_3)(rh.superiortemporal_6)
3056	0.89855	(Right-Putamen)(rh.caudalmiddlefrontal_4)(rh.rostralmiddlefrontal_1)
3057	0.89855	(Right-Putamen)(rh.insula_1)(rh.superiorparietal_1)(rh.supramarginal_1)
3058	0.89855	(Right-Putamen)(rh.lateralorbitofrontal_2)(rh.lateralorbitofrontal_4)(rh.lateralorbitofrontal_6)
3059	0.89855	(Right-Putamen)(rh.lateralorbitofrontal_6)(rh.lateralorbitofrontal_7)(rh.parstriangularis_1)(rh.rostralmiddlefrontal_12)
3060	0.89855	(Right-Putamen)(rh.lateralorbitofrontal_7)(rh.rostralmiddlefrontal_8)
3061	0.89855	(Right-Putamen)(rh.postcentral_8)
3062	0.89855	(Right-Putamen)(rh.rostralmiddlefrontal_3)(rh.rostralmiddlefrontal_5)(rh.rostralmiddlefrontal_8)
3063	0.89855	(Right-Putamen)(rh.superiorfrontal_14)
3064	0.89855	(Right-Thalamus-Proper)(rh.precentral_11)(rh.precentral_13)
3065	0.89614	(Left-Caudate)(Left-Putamen)(lh.caudalmiddlefrontal_1)(lh.rostralmiddlefrontal_4)
3066	0.89614	(Left-Caudate)(Left-Putamen)(lh.caudalmiddlefrontal_6)(lh.precentral_11)(lh.precentral_9)
3067	0.89614	(Left-Caudate)(Left-Putamen)(lh.medialorbitofrontal_2)(lh.rostralmiddlefrontal_12)(lh.rostralmiddlefrontal_9)
3068	0.89614	(Left-Caudate)(Left-Putamen)(lh.rostralanteriorcingulate_1)(lh.rostralanteriorcingulate_2)(lh.superiorfrontal_4)
3069	0.89614	(Left-Caudate)(Left-Putamen)(lh.rostralanteriorcingulate_2)(lh.rostralmiddlefrontal_9)
3070	0.89614	(Left-Caudate)(Left-Thalamus-Proper)(lh.precentral_11)(lh.precentral_9)
3071	0.89614	(Left-Caudate)(lh.caudalanteriorcingulate_2)(lh.superiorfrontal_4)
3072	0.89614	(Left-Hippocampus)(lh.fusiform_5)(lh.fusiform_6)
3073	0.89614	(Left-Putamen)(Left-Thalamus-Proper)(lh.insula_1)(lh.supramarginal_1)
3074	0.89614	(Left-Putamen)(Left-Thalamus-Proper)(lh.supramarginal_1)
3075	0.89614	(Left-Putamen)(lh.insula_2)(lh.precentral_9)
3076	0.89614	(Left-Putamen)(lh.lateralorbitofrontal_4)(lh.lateralorbitofrontal_6)(lh.parstriangularis_3)(lh.rostralmiddlefrontal_12)
3077	0.89614	(Left-Putamen)(lh.lateralorbitofrontal_4)(lh.rostralanteriorcingulate_1)
3078	0.89614	(Left-Putamen)(lh.medialorbitofrontal_4)(lh.medialorbitofrontal_5)
3079	0.89614	(Left-Putamen)(lh.precentral_7)(lh.precentral_9)
3080	0.89614	(Left-Putamen)(lh.rostralanteriorcingulate_1)(lh.rostralanteriorcingulate_2)(lh.rostralmiddlefrontal_9)
3081	0.89614	(Left-Putamen)(lh.rostralmiddlefrontal_12)(lh.superiorfrontal_3)
3082	0.89614	(Left-Putamen)(lh.rostralmiddlefrontal_9)(lh.superiorfrontal_4)
3083	0.89614	(Left-Putamen)(lh.superiorfrontal_12)(lh.superiorfrontal_14)
3084	0.89614	(lh.caudalmiddlefrontal_3)(lh.parstriangularis_3)
3085	0.89614	(lh.cuneus_3)(lh.pericalcarine_1)
3086	0.89614	(lh.fusiform_2)(lh.lingual_1)(lh.lingual_4)(lh.pericalcarine_3)
3087	0.89614	(lh.fusiform_7)(lh.inferiortemporal_3)(lh.middletemporal_4)
3088	0.89614	(lh.inferiorparietal_8)(lh.superiorparietal_6)
3089	0.89614	(lh.insula_6)(lh.parstriangularis_3)(lh.rostralmiddlefrontal_9)
3090	0.89614	(lh.isthmuscingulate_1)(lh.posteriorcingulate_2)
3091	0.89614	(lh.lateralorbitofrontal_4)(lh.rostralanteriorcingulate_1)
3092	0.89614	(lh.lateralorbitofrontal_6)(lh.parsorbitalis_1)(lh.rostralmiddlefrontal_6)
3093	0.89614	(lh.medialorbitofrontal_2)(lh.parstriangularis_3)
3094	0.89614	(lh.parsopercularis_2)(lh.parsopercularis_3)
3095	0.89614	(lh.parsorbitalis_1)(lh.parstriangularis_1)(lh.parstriangularis_3)
3096	0.89614	(lh.postcentral_8)(lh.precentral_4)(lh.precentral_7)
3097	0.89614	(lh.precentral_4)(lh.precentral_6)
3098	0.89614	(lh.precuneus_10)(lh.precuneus_11)(lh.precuneus_6)(lh.precuneus_9)
3099	0.89614	(lh.precuneus_10)(lh.precuneus_6)(lh.superiorparietal_11)
3100	0.89614	(lh.precuneus_9)(lh.superiorparietal_5)

3101	0.89614	(lh.superiorfrontal_10)(lh.superiorfrontal_14)(lh.superiorfrontal_8)
3102	0.89614	(lh.superiorfrontal_10)(lh.superiorfrontal_14)
3103	0.89614	(lh.superiorfrontal_10)(lh.superiorfrontal_4)(lh.superiorfrontal_6)(lh.superiorfrontal_7)(lh.superiorfrontal_8)
3104	0.89614	(lh.superiorfrontal_10)(lh.superiorfrontal_6)(lh.superiorfrontal_7)(lh.superiorfrontal_8)
3105	0.89614	(lh.superiorfrontal_11)(lh.superiorfrontal_6)(lh.superiorfrontal_7)
3106	0.89614	(lh.superiorfrontal_17)(lh.superiorfrontal_18)
3107	0.89614	(rh.bankssts_2)(rh.superiortemporal_1)(rh.superiortemporal_3)
3108	0.89614	(rh.caudalmiddlefrontal_4)(rh.insula_7)(rh.parsopercularis_4)
3109	0.89614	(rh.caudalmiddlefrontal_4)(rh.rostralmiddlefrontal_1)(rh.rostralmiddlefrontal_3)
3110	0.89614	(rh.fusiform_1)(rh.inferiorparietal_10)(rh.lateraloccipital_10)(rh.lateraloccipital_8)
3111	0.89614	(rh.fusiform_1)(rh.lateraloccipital_10)(rh.lingual_7)
3112	0.89614	(rh.fusiform_6)(rh.inferiortemporal_6)
3113	0.89614	(rh.insula_7)(rh.parsopercularis_4)(rh.rostralmiddlefrontal_3)
3114	0.89614	(rh.lateraloccipital_3)(rh.lateraloccipital_4)(rh.pericalcarine_1)
3115	0.89614	(rh.lateraloccipital_4)(rh.lateraloccipital_7)(rh.lateraloccipital_8)(rh.lingual_2)
3116	0.89614	(rh.lateraloccipital_5)(rh.lateraloccipital_6)
3117	0.89614	(rh.lateralorbitofrontal_4)(rh.lateralorbitofrontal_6)(rh.lateralorbitofrontal_7)
3118	0.89614	(rh.lateralorbitofrontal_7)(rh.medialorbitofrontal_4)
3119	0.89614	(rh.lingual_1)(rh.lingual_2)
3120	0.89614	(rh.medialorbitofrontal_1)(rh.rostralmiddlefrontal_11)
3121	0.89614	(rh.middletemporal_4)(rh.middletemporal_5)
3122	0.89614	(rh.parsopercularis_2)(rh.parstriangularis_1)
3123	0.89614	(rh.parstriangularis_1)(rh.rostralmiddlefrontal_5)
3124	0.89614	(rh.precentral_5)(rh.precentral_6)(rh.precentral_8)
3125	0.89614	(rh.superiorfrontal_1)(rh.superiorfrontal_3)
3126	0.89614	(rh.superiorfrontal_11)(rh.superiorfrontal_12)(rh.superiorfrontal_16)
3127	0.89614	(rh.superiorfrontal_11)(rh.superiorfrontal_14)
3128	0.89614	(rh.superiorfrontal_2)(rh.superiorfrontal_7)(rh.superiorfrontal_8)
3129	0.89614	(rh.superiorfrontal_2)(rh.superiorfrontal_9)
3130	0.89614	(Right-Caudate)(Right-Pallidum)(rh.posteriorcingulate_2)
3131	0.89614	(Right-Caudate)(Right-Putamen)(rh.superiorfrontal_11)
3132	0.89614	(Right-Caudate)(Right-Putamen)(Right-Thalamus-Proper)(rh.precentral_11)
3133	0.89614	(Right-Pallidum)(rh.posteriorcingulate_2)
3134	0.89614	(Right-Putamen)(rh.caudalmiddlefrontal_4)(rh.insula_7)(rh.parsopercularis_4)
3135	0.89614	(Right-Putamen)(rh.lateralorbitofrontal_4)(rh.lateralorbitofrontal_5)(rh.lateralorbitofrontal_7)
3136	0.89614	(Right-Putamen)(rh.precentral_11)(rh.supramarginal_1)
3137	0.89372	(Left-Accumbens-area)(lh.medialorbitofrontal_4)
3138	0.89372	(Left-Caudate)(Left-Putamen)(Left-Thalamus-Proper)(lh.precentral_11)(lh.precentral_9)
3139	0.89372	(Left-Caudate)(Left-Putamen)(lh.lateralorbitofrontal_7)(lh.medialorbitofrontal_2)(lh.rostralmiddlefrontal_12)
3140	0.89372	(Left-Caudate)(Left-Putamen)(lh.lateralorbitofrontal_7)(lh.medialorbitofrontal_2)(lh.rostralmiddlefrontal_9)
3141	0.89372	(Left-Caudate)(Left-Putamen)(lh.medialorbitofrontal_2)(lh.rostralanteriorcingulate_1)(lh.rostralmiddlefrontal_12)
3142	0.89372	(Left-Caudate)(Left-Putamen)(lh.postcentral_8)(lh.precentral_4)
3143	0.89372	(Left-Caudate)(lh.insula_6)(lh.rostralmiddlefrontal_9)
3144	0.89372	(Left-Caudate)(lh.lateralorbitofrontal_4)(lh.parstriangularis_3)
3145	0.89372	(Left-Caudate)(lh.lateralorbitofrontal_6)(lh.parstriangularis_3)
3146	0.89372	(Left-Caudate)(lh.rostralmiddlefrontal_8)(lh.superiorfrontal_4)
3147	0.89372	(Left-Caudate)(lh.superiorfrontal_14)(lh.superiorfrontal_4)
3148	0.89372	(Left-Hippocampus)(lh.bankssts_2)(lh.bankssts_3)
3149	0.89372	(Left-Putamen)(lh.insula_6)(lh.rostralmiddlefrontal_1)(lh.rostralmiddlefrontal_4)
3150	0.89372	(Left-Putamen)(lh.lateralorbitofrontal_4)(lh.parstriangularis_3)(lh.rostralmiddlefrontal_12)(lh.rostralmiddlefrontal_9)

3151	0.89372	(Left-Putamen)(lh.lateralorbitofrontal_7)(lh.parstriangularis_3)(lh.rostralmiddlefrontal_12)(lh.rostralmiddlefrontal_9)
3152	0.89372	(Left-Putamen)(lh.lateralorbitofrontal_7)(lh.rostralanteriorcingulate_1)(lh.rostralmiddlefrontal_9)
3153	0.89372	(Left-Putamen)(lh.rostralanteriorcingulate_1)(lh.superiorfrontal_3)
3154	0.89372	(lh.caudalanteriorcingulate_1)(lh.superiorfrontal_10)
3155	0.89372	(lh.caudalanteriorcingulate_2)(lh.caudalmiddlefrontal_5)
3156	0.89372	(lh.caudalmiddlefrontal_2)(lh.caudalmiddlefrontal_4)
3157	0.89372	(lh.cuneus_1)(lh.cuneus_2)(lh.superiorparietal_14)
3158	0.89372	(lh.cuneus_2)(lh.pericalcarine_1)(lh.superiorparietal_14)
3159	0.89372	(lh.fusiform_2)(lh.lateraloccipital_8)
3160	0.89372	(lh.inferiorparietal_1)(lh.lateraloccipital_2)
3161	0.89372	(lh.inferiorparietal_1)(lh.lateraloccipital_7)(lh.pericalcarine_3)
3162	0.89372	(lh.inferiorparietal_7)(lh.inferiorparietal_8)(lh.inferiorparietal_9)
3163	0.89372	(lh.inferiortemporal_5)(lh.middletemporal_3)
3164	0.89372	(lh.inferiortemporal_8)(lh.middletemporal_2)
3165	0.89372	(lh.insula_1)(lh.postcentral_14)(lh.supramarginal_1)
3166	0.89372	(lh.insula_1)(lh.supramarginal_1)(lh.supramarginal_4)
3167	0.89372	(lh.insula_2)(lh.precentral_11)(lh.precentral_9)
3168	0.89372	(lh.lateraloccipital_2)(lh.lateraloccipital_3)(lh.lateraloccipital_6)
3169	0.89372	(lh.lateraloccipital_6)(lh.lateraloccipital_9)
3170	0.89372	(lh.lateraloccipital_6)(lh.lingual_4)(lh.pericalcarine_3)
3171	0.89372	(lh.lateralorbitofrontal_4)(lh.lateralorbitofrontal_6)(lh.lateralorbitofrontal_7)(lh.rostralmiddlefrontal_9)
3172	0.89372	(lh.lateralorbitofrontal_7)(lh.medialorbitofrontal_2)(lh.medialorbitofrontal_4)
3173	0.89372	(lh.lingual_5)(lh.lingual_8)
3174	0.89372	(lh.medialorbitofrontal_1)(lh.superiorfrontal_1)
3175	0.89372	(lh.paracentral_4)(lh.paracentral_5)(lh.precentral_2)(lh.precentral_3)
3176	0.89372	(lh.pericalcarine_2)(lh.superiorparietal_12)
3177	0.89372	(lh.rostralmiddlefrontal_4)(lh.rostralmiddlefrontal_9)(lh.superiorfrontal_3)
3178	0.89372	(lh.rostralmiddlefrontal_9)(lh.superiorfrontal_2)
3179	0.89372	(lh.superiorfrontal_10)(lh.superiorfrontal_11)(lh.superiorfrontal_7)(lh.superiorfrontal_9)
3180	0.89372	(lh.superiorfrontal_11)(lh.superiorfrontal_4)(lh.superiorfrontal_6)(lh.superiorfrontal_7)
3181	0.89372	(lh.superiorfrontal_11)(lh.superiorfrontal_4)(lh.superiorfrontal_9)
3182	0.89372	(rh.bankssts_2)(rh.bankssts_3)(rh.inferiorparietal_4)
3183	0.89372	(rh.fusiform_3)(rh.lateraloccipital_10)(rh.lateraloccipital_8)
3184	0.89372	(rh.fusiform_3)(rh.lateraloccipital_8)
3185	0.89372	(rh.inferiorparietal_10)(rh.lingual_5)
3186	0.89372	(rh.inferiorparietal_10)(rh.superiorparietal_11)
3187	0.89372	(rh.inferiorparietal_12)(rh.lateraloccipital_4)(rh.superiorparietal_11)
3188	0.89372	(rh.inferiortemporal_3)(rh.middletemporal_6)
3189	0.89372	(rh.inferiortemporal_3)(rh.middletemporal_7)
3190	0.89372	(rh.lateraloccipital_1)(rh.superiorparietal_11)
3191	0.89372	(rh.lateraloccipital_4)(rh.pericalcarine_1)(rh.superiorparietal_11)
3192	0.89372	(rh.lateraloccipital_5)(rh.lateraloccipital_7)(rh.lateraloccipital_8)
3193	0.89372	(rh.lateralorbitofrontal_2)(rh.parstriangularis_1)(rh.rostralmiddlefrontal_8)
3194	0.89372	(rh.lingual_7)(rh.pericalcarine_1)
3195	0.89372	(rh.medialorbitofrontal_2)(rh.rostralmiddlefrontal_12)(rh.superiorfrontal_2)
3196	0.89372	(rh.paracentral_4)(rh.superiorfrontal_14)
3197	0.89372	(rh.parstriangularis_1)(rh.parstriangularis_3)(rh.parstriangularis_4)
3198	0.89372	(rh.parstriangularis_1)(rh.rostralmiddlefrontal_12)(rh.rostralmiddlefrontal_3)(rh.rostralmiddlefrontal_8)
3199	0.89372	(rh.parstriangularis_1)(rh.rostralmiddlefrontal_5)(rh.rostralmiddlefrontal_8)
3200	0.89372	(rh.rostralmiddlefrontal_12)(rh.rostralmiddlefrontal_5)

3201	0.89372	(rh.rostralmiddlefrontal_9)(rh.superiorfrontal_2)(rh.superiorfrontal_7)
3202	0.89372	(rh.superiorfrontal_2)(rh.superiorfrontal_3)(rh.superiorfrontal_4)
3203	0.89372	(rh.superiorfrontal_2)(rh.superiorfrontal_6)(rh.superiorfrontal_8)
3204	0.89372	(Right-Caudate)(rh.insula_2)
3205	0.89372	(Right-Caudate)(rh.lateralorbitofrontal_5)(rh.lateralorbitofrontal_7)
3206	0.89372	(Right-Caudate)(rh.superiorfrontal_11)(rh.superiorfrontal_9)
3207	0.89372	(Right-Caudate)(rh.superiorfrontal_7)(rh.superiorfrontal_9)
3208	0.89372	(Right-Caudate)(Right-Pallidum)(Right-Putamen)(rh.posteriorcingulate_2)
3209	0.89372	(Right-Caudate)(Right-Putamen)(rh.insula_2)
3210	0.89372	(Right-Caudate)(Right-Putamen)(Right-Thalamus-Proper)(rh.insula_2)
3211	0.89372	(Right-Caudate)(Right-Thalamus-Proper)(rh.insula_2)
3212	0.89372	(Right-Hippocampus)(rh.inferiorparietal_4)(rh.insula_2)
3213	0.89372	(Right-Hippocampus)(rh.insula_5)(rh.superiortemporal_10)
3214	0.89372	(Right-Hippocampus)(rh.isthmuscingulate_2)(rh.precuneus_3)(rh.precuneus_7)
3215	0.89372	(Right-Pallidum)(Right-Putamen)(rh.posteriorcingulate_2)
3216	0.89372	(Right-Putamen)(rh.caudalmiddlefrontal_4)(rh.rostralmiddlefrontal_5)
3217	0.89372	(Right-Putamen)(rh.caudalmiddlefrontal_4)(rh.superiorfrontal_11)
3218	0.89372	(Right-Putamen)(rh.lateralorbitofrontal_2)(rh.lateralorbitofrontal_7)(rh.parstriangularis_1)
3219	0.89372	(Right-Putamen)(rh.lateralorbitofrontal_4)(rh.lateralorbitofrontal_6)(rh.lateralorbitofrontal_7)
3220	0.89372	(Right-Putamen)(rh.postcentral_9)
3221	0.8913	(Left-Accumbens-area)(Left-Amygdala)(Left-Pallidum)(Left-Thalamus-Proper)
3222	0.8913	(Left-Accumbens-area)(Left-Amygdala)(Left-Pallidum)
3223	0.8913	(Left-Accumbens-area)(Left-Amygdala)(Left-Thalamus-Proper)
3224	0.8913	(Left-Accumbens-area)(Left-Amygdala)
3225	0.8913	(Left-Amygdala)(Left-Hippocampus)(lh.fusiform_8)
3226	0.8913	(Left-Caudate)(Left-Putamen)(lh.lateralorbitofrontal_6)(lh.rostralmiddlefrontal_9)
3227	0.8913	(Left-Caudate)(Left-Putamen)(lh.lateralorbitofrontal_7)(lh.medialorbitofrontal_4)
3228	0.8913	(Left-Caudate)(Left-Putamen)(lh.medialorbitofrontal_4)(lh.medialorbitofrontal_5)
3229	0.8913	(Left-Caudate)(lh.caudalanteriorcingulate_2)(lh.rostralanteriorcingulate_2)(lh.superiorfrontal_11)
3230	0.8913	(Left-Caudate)(lh.caudalanteriorcingulate_2)(lh.superiorfrontal_11)(lh.superiorfrontal_12)(lh.superiorfrontal_8)
3231	0.8913	(Left-Caudate)(lh.caudalanteriorcingulate_2)(lh.superiorfrontal_12)(lh.superiorfrontal_14)(lh.superiorfrontal_8)
3232	0.8913	(Left-Caudate)(lh.lateralorbitofrontal_7)(lh.parstriangularis_3)(lh.rostralmiddlefrontal_9)
3233	0.8913	(Left-Caudate)(lh.medialorbitofrontal_2)(lh.rostralanteriorcingulate_1)(lh.rostralmiddlefrontal_12)(lh.rostralmiddlefrontal_9)
3234	0.8913	(Left-Caudate)(lh.parstriangularis_3)(lh.rostralmiddlefrontal_12)(lh.rostralmiddlefrontal_9)
3235	0.8913	(Left-Caudate)(lh.rostralanteriorcingulate_1)(lh.rostralmiddlefrontal_4)(lh.rostralmiddlefrontal_9)
3236	0.8913	(Left-Caudate)(lh.rostralanteriorcingulate_2)(lh.superiorfrontal_10)
3237	0.8913	(Left-Caudate)(lh.rostralmiddlefrontal_4)(lh.rostralmiddlefrontal_9)(lh.superiorfrontal_4)
3238	0.8913	(Left-Caudate)(lh.rostralmiddlefrontal_4)(lh.superiorfrontal_10)(lh.superiorfrontal_4)
3239	0.8913	(Left-Caudate)(lh.superiorfrontal_10)(lh.superiorfrontal_11)(lh.superiorfrontal_4)(lh.superiorfrontal_8)
3240	0.8913	(Left-Caudate)(lh.superiorfrontal_11)(lh.superiorfrontal_12)(lh.superiorfrontal_14)(lh.superiorfrontal_8)
3241	0.8913	(Left-Caudate)(Right-Accumbens-area)(Right-Caudate)
3242	0.8913	(Left-Putamen)(lh.caudalanteriorcingulate_2)(lh.superiorfrontal_11)(lh.superiorfrontal_12)
3243	0.8913	(Left-Putamen)(lh.caudalmiddlefrontal_3)(lh.superiorfrontal_12)
3244	0.8913	(Left-Putamen)(lh.insula_2)(lh.precentral_11)(lh.precentral_9)
3245	0.8913	(Left-Putamen)(lh.lateralorbitofrontal_2)(lh.medialorbitofrontal_5)
3246	0.8913	(Left-Putamen)(lh.lateralorbitofrontal_6)(lh.lateralorbitofrontal_7)(lh.parstriangularis_3)(lh.rostralmiddlefrontal_9)
3247	0.8913	(Left-Putamen)(lh.lateralorbitofrontal_6)(lh.parstriangularis_3)(lh.rostralmiddlefrontal_1)(lh.rostralmiddlefrontal_9)
3248	0.8913	(Left-Putamen)(lh.parsopercularis_3)(lh.parstriangularis_3)(lh.rostralmiddlefrontal_1)
3249	0.8913	(Left-Putamen)(lh.rostralmiddlefrontal_12)(lh.rostralmiddlefrontal_9)(lh.superiorfrontal_3)
3250	0.8913	(Left-Putamen)(lh.supramarginal_9)

3251	0.8913	(lh.caudalanteriorcingulate_2)(lh.caudalmiddlefrontal_1)(lh.caudalmiddlefrontal_3)
3252	0.8913	(lh.caudalmiddlefrontal_3)(lh.rostralmiddlefrontal_1)(lh.rostralmiddlefrontal_3)(lh.rostralmiddlefrontal_4)
3253	0.8913	(lh.caudalmiddlefrontal_4)(lh.superiorfrontal_12)
3254	0.8913	(lh.caudalmiddlefrontal_5)(lh.precentral_9)
3255	0.8913	(lh.fusiform_2)(lh.lateraloccipital_10)(lh.lateraloccipital_11)
3256	0.8913	(lh.inferiorparietal_9)(lh.superiortemporal_2)
3257	0.8913	(lh.inferiortemporal_3)(lh.inferiortemporal_6)(lh.middletemporal_3)
3258	0.8913	(lh.insula_3)(lh.precentral_12)
3259	0.8913	(lh.isthmuscingulate_2)(lh.isthmuscingulate_3)(lh.precuneus_6)
3260	0.8913	(lh.isthmuscingulate_2)(lh.precuneus_10)(lh.precuneus_11)
3261	0.8913	(lh.isthmuscingulate_2)(lh.precuneus_10)(lh.precuneus_6)
3262	0.8913	(lh.lateraloccipital_1)(lh.superiorparietal_14)
3263	0.8913	(lh.lateraloccipital_11)(lh.lateraloccipital_6)(lh.pericalcarine_3)
3264	0.8913	(lh.lateraloccipital_2)(lh.lateraloccipital_6)(lh.pericalcarine_2)
3265	0.8913	(lh.lateraloccipital_8)(lh.lateraloccipital_9)
3266	0.8913	(lh.lateralorbitofrontal_1)(lh.lateralorbitofrontal_2)
3267	0.8913	(lh.lateralorbitofrontal_6)(lh.parstriangularis_3)(lh.rostralmiddlefrontal_1)(lh.rostralmiddlefrontal_6)
3268	0.8913	(lh.lateralorbitofrontal_7)(lh.medialorbitofrontal_3)
3269	0.8913	(lh.medialorbitofrontal_2)(lh.rostralanteriorcingulate_1)(lh.rostralmiddlefrontal_12)(lh.rostralmiddlefrontal_9)(lh.superiorfrontal_3)
3270	0.8913	(lh.parsopercularis_1)(lh.parsopercularis_2)(lh.rostralmiddlefrontal_1)
3271	0.8913	(lh.pericalcarine_1)(lh.pericalcarine_2)(lh.pericalcarine_3)
3272	0.8913	(lh.precuneus_3)(lh.precuneus_6)(lh.superiorparietal_5)(lh.superiorparietal_6)
3273	0.8913	(lh.precuneus_4)(lh.precuneus_6)
3274	0.8913	(lh.rostralmiddlefrontal_8)(lh.superiorfrontal_2)(lh.superiorfrontal_3)
3275	0.8913	(lh.superiorfrontal_4)(lh.superiorfrontal_8)(lh.superiorfrontal_9)
3276	0.8913	(rh.caudalanteriorcingulate_2)(rh.caudalmiddlefrontal_4)
3277	0.8913	(rh.caudalmiddlefrontal_5)(rh.rostralmiddlefrontal_2)
3278	0.8913	(rh.fusiform_4)(rh.inferiortemporal_7)
3279	0.8913	(rh.inferiorparietal_1)(rh.inferiorparietal_3)
3280	0.8913	(rh.inferiorparietal_1)(rh.superiorparietal_1)(rh.supramarginal_9)
3281	0.8913	(rh.inferiortemporal_5)(rh.inferiortemporal_6)(rh.middletemporal_2)
3282	0.8913	(rh.inferiortemporal_5)(rh.middletemporal_4)
3283	0.8913	(rh.insula_3)(rh.precentral_10)(rh.precentral_7)
3284	0.8913	(rh.lateraloccipital_3)(rh.lateraloccipital_4)(rh.lateraloccipital_8)
3285	0.8913	(rh.lingual_4)(rh.lingual_6)
3286	0.8913	(rh.postcentral_1)(rh.precentral_4)
3287	0.8913	(rh.precentral_6)(rh.precentral_7)
3288	0.8913	(rh.rostralmiddlefrontal_6)(rh.rostralmiddlefrontal_7)
3289	0.8913	(rh.superiorfrontal_10)(rh.superiorfrontal_11)(rh.superiorfrontal_13)(rh.superiorfrontal_9)
3290	0.8913	(rh.superiorfrontal_11)(rh.superiorfrontal_15)
3291	0.8913	(rh.superiorfrontal_11)(rh.superiorfrontal_5)(rh.superiorfrontal_7)(rh.superiorfrontal_8)(rh.superiorfrontal_9)
3292	0.8913	(rh.superiorfrontal_11)(rh.superiorfrontal_5)(rh.superiorfrontal_7)(rh.superiorfrontal_9)
3293	0.8913	(rh.superiorfrontal_13)(rh.superiorfrontal_5)
3294	0.8913	(rh.superiorfrontal_2)(rh.superiorfrontal_5)(rh.superiorfrontal_6)(rh.superiorfrontal_8)
3295	0.8913	(rh.superiorfrontal_3)(rh.superiorfrontal_5)(rh.superiorfrontal_6)
3296	0.8913	(Right-Accumbens-area)(Right-Caudate)(Right-Hippocampus)(Right-Pallidum)(Right-Putamen)(Right-Thalamus-Proper)
3297	0.8913	(Right-Accumbens-area)(Right-Caudate)(Right-Hippocampus)(Right-Pallidum)(Right-Putamen)
3298	0.8913	(Right-Accumbens-area)(Right-Caudate)(Right-Hippocampus)(Right-Putamen)(Right-Thalamus-Proper)
3299	0.8913	(Right-Accumbens-area)(Right-Caudate)(Right-Hippocampus)(Right-Putamen)
3300	0.8913	(Right-Amygdala)(Right-Caudate)(Right-Hippocampus)(Right-Pallidum)(Right-Putamen)(Right-Thalamus-Proper)

3301	0.8913	(Right-Amygdala)(Right-Caudate)(Right-Hippocampus)(Right-Pallidum)(Right-Putamen)
3302	0.8913	(Right-Amygdala)(Right-Caudate)(Right-Hippocampus)(Right-Pallidum)(Right-Thalamus-Proper)
3303	0.8913	(Right-Amygdala)(Right-Caudate)(Right-Hippocampus)(Right-Pallidum)
3304	0.8913	(Right-Amygdala)(Right-Caudate)(Right-Hippocampus)(Right-Putamen)(Right-Thalamus-Proper)
3305	0.8913	(Right-Amygdala)(Right-Caudate)(Right-Hippocampus)(Right-Putamen)
3306	0.8913	(Right-Amygdala)(Right-Caudate)(Right-Hippocampus)(Right-Thalamus-Proper)
3307	0.8913	(Right-Amygdala)(Right-Caudate)(Right-Hippocampus)
3308	0.8913	(Right-Caudate)(rh.caudalanteriorcingulate_2)(rh.caudalmiddlefrontal_4)
3309	0.8913	(Right-Caudate)(rh.rostralmiddlefrontal_12)
3310	0.8913	(Right-Caudate)(rh.rostralmiddlefrontal_8)(rh.superiorfrontal_2)
3311	0.8913	(Right-Caudate)(rh.superiorfrontal_11)(rh.superiorfrontal_5)
3312	0.8913	(Right-Caudate)(rh.superiorfrontal_5)(rh.superiorfrontal_7)
3313	0.8913	(Right-Caudate)(Right-Putamen)(rh.lateralorbitofrontal_4)(rh.lateralorbitofrontal_5)
3314	0.8913	(Right-Caudate)(Right-Putamen)(rh.rostralmiddlefrontal_12)
3315	0.8913	(Right-Caudate)(Right-Thalamus-Proper)(rh.posteriorcingulate_4)
3316	0.8913	(Right-Putamen)(rh.lateralorbitofrontal_2)(rh.lateralorbitofrontal_6)(rh.lateralorbitofrontal_7)(rh.parstriangularis_1)
3317	0.8913	(Right-Putamen)(rh.lateralorbitofrontal_7)(rh.rostralmiddlefrontal_12)(rh.rostralmiddlefrontal_8)
3318	0.8913	(Right-Putamen)(rh.medialorbitofrontal_2)(rh.rostralmiddlefrontal_11)
3319	0.8913	(Right-Putamen)(rh.postcentral_1)(rh.postcentral_4)
3320	0.8913	(Right-Putamen)(rh.precentral_13)(rh.precentral_15)
3321	0.8913	(Right-Thalamus-Proper)(rh.posteriorcingulate_4)
3322	0.88889	(Left-Accumbens-area)(Left-Caudate)(Left-Putamen)(lh.medialorbitofrontal_5)
3323	0.88889	(Left-Caudate)(Left-Putamen)(lh.caudalanteriorcingulate_1)(lh.caudalanteriorcingulate_2)(lh.rostralanteriorcingulate_1)
3324	0.88889	(Left-Caudate)(Left-Putamen)(lh.caudalmiddlefrontal_1)(lh.caudalmiddlefrontal_3)(lh.caudalmiddlefrontal_4)
3325	0.88889	(Left-Caudate)(Left-Putamen)(lh.insula_1)(lh.supramarginal_1)
3326	0.88889	(Left-Caudate)(Left-Putamen)(lh.lateralorbitofrontal_7)(lh.medialorbitofrontal_2)(lh.rostralanteriorcingulate_1)
3327	0.88889	(Left-Caudate)(Left-Putamen)(lh.postcentral_8)(lh.precentral_7)
3328	0.88889	(Left-Caudate)(Left-Putamen)(lh.superiorfrontal_10)
3329	0.88889	(Left-Caudate)(lh.paracentral_5)(lh.superiorfrontal_12)(lh.superiorfrontal_14)
3330	0.88889	(Left-Caudate)(lh.rostralanteriorcingulate_2)(lh.rostralmiddlefrontal_4)(lh.superiorfrontal_4)
3331	0.88889	(Left-Hippocampus)(lh.fusiform_5)(lh.lateraloccipital_9)
3332	0.88889	(Left-Hippocampus)(lh.isthmuscingulate_2)(lh.isthmuscingulate_3)(lh.precuneus_11)
3333	0.88889	(Left-Hippocampus)(lh.isthmuscingulate_2)(lh.precuneus_11)
3334	0.88889	(Left-Pallidum)(Left-Putamen)(lh.insula_1)(lh.precentral_11)
3335	0.88889	(Left-Pallidum)(lh.insula_1)(lh.precentral_11)
3336	0.88889	(Left-Putamen)(lh.caudalanteriorcingulate_1)(lh.caudalanteriorcingulate_2)(lh.rostralanteriorcingulate_1)
3337	0.88889	(Left-Putamen)(lh.lateralorbitofrontal_1)(lh.lateralorbitofrontal_2)
3338	0.88889	(Left-Putamen)(lh.rostralmiddlefrontal_8)(lh.superiorfrontal_4)
3339	0.88889	(lh.caudalmiddlefrontal_1)(lh.caudalmiddlefrontal_3)(lh.rostralmiddlefrontal_3)
3340	0.88889	(lh.caudalmiddlefrontal_3)(lh.parstriangularis_3)(lh.rostralmiddlefrontal_1)
3341	0.88889	(lh.caudalmiddlefrontal_3)(lh.superiorfrontal_11)(lh.superiorfrontal_12)
3342	0.88889	(lh.fusiform_3)(lh.fusiform_4)
3343	0.88889	(lh.inferiorparietal_2)(lh.inferiorparietal_8)
3344	0.88889	(lh.inferiorparietal_4)(lh.lateraloccipital_7)
3345	0.88889	(lh.insula_6)(lh.parstriangularis_3)(lh.rostralmiddlefrontal_4)
3346	0.88889	(lh.lateraloccipital_10)(lh.lateraloccipital_6)
3347	0.88889	(lh.lateraloccipital_6)(lh.lingual_1)(lh.pericalcarine_2)(lh.pericalcarine_3)
3348	0.88889	(lh.lateralorbitofrontal_3)(lh.lateralorbitofrontal_4)(lh.lateralorbitofrontal_6)
3349	0.88889	(lh.lateralorbitofrontal_4)(lh.lateralorbitofrontal_6)(lh.lateralorbitofrontal_7)(lh.parstriangularis_3)(lh.rostralmiddlefrontal_12)
3350	0.88889	(lh.lateralorbitofrontal_4)(lh.lateralorbitofrontal_7)(lh.parstriangularis_3)(lh.rostralmiddlefrontal_9)

3351	0.88889	(lh.lateralorbitofrontal_7)(lh.rostralmiddlefrontal_6)
3352	0.88889	(lh.medialorbitofrontal_1)(lh.medialorbitofrontal_2)(lh.rostralmiddlefrontal_12)
3353	0.88889	(lh.medialorbitofrontal_2)(lh.rostralmiddlefrontal_11)
3354	0.88889	(lh.precuneus_3)(lh.superiorparietal_8)
3355	0.88889	(lh.precuneus_6)(lh.superiorparietal_11)(lh.superiorparietal_5)
3356	0.88889	(lh.rostralmiddlefrontal_1)(lh.rostralmiddlefrontal_12)
3357	0.88889	(lh.rostralmiddlefrontal_12)(lh.rostralmiddlefrontal_9)(lh.superiorfrontal_1)
3358	0.88889	(lh.rostralmiddlefrontal_12)(lh.superiorfrontal_4)
3359	0.88889	(lh.rostralmiddlefrontal_4)(lh.superiorfrontal_10)(lh.superiorfrontal_11)(lh.superiorfrontal_4)
3360	0.88889	(lh.superiorfrontal_11)(lh.superiorfrontal_13)(lh.superiorfrontal_14)(lh.superiorfrontal_9)
3361	0.88889	(lh.superiorfrontal_4)(lh.superiorfrontal_7)(lh.superiorfrontal_8)(lh.superiorfrontal_9)
3362	0.88889	(rh.bankssts_3)(rh.superiortemporal_1)
3363	0.88889	(rh.caudalmiddlefrontal_2)(rh.caudalmiddlefrontal_5)
3364	0.88889	(rh.entorhinal_1)(rh.parahippocampal_3)
3365	0.88889	(rh.fusiform_1)(rh.fusiform_3)(rh.lateraloccipital_10)(rh.lateraloccipital_8)
3366	0.88889	(rh.fusiform_1)(rh.fusiform_3)(rh.lateraloccipital_8)
3367	0.88889	(rh.fusiform_1)(rh.lateraloccipital_4)(rh.lateraloccipital_7)(rh.lingual_2)
3368	0.88889	(rh.fusiform_1)(rh.lateraloccipital_8)(rh.lingual_5)(rh.lingual_7)
3369	0.88889	(rh.fusiform_1)(rh.lingual_4)(rh.lingual_5)
3370	0.88889	(rh.inferiortemporal_2)(rh.inferiortemporal_3)
3371	0.88889	(rh.inferiortemporal_5)(rh.inferiortemporal_7)
3372	0.88889	(rh.insula_1)(rh.postcentral_1)(rh.precentral_7)
3373	0.88889	(rh.lateraloccipital_5)(rh.pericalcarine_4)
3374	0.88889	(rh.lateralorbitofrontal_6)(rh.parsorbitalis_2)(rh.rostralmiddlefrontal_12)
3375	0.88889	(rh.medialorbitofrontal_2)(rh.superiorfrontal_1)
3376	0.88889	(rh.pericalcarine_1)(rh.pericalcarine_2)
3377	0.88889	(rh.pericalcarine_2)(rh.precuneus_1)(rh.superiorparietal_11)
3378	0.88889	(rh.postcentral_10)(rh.supramarginal_1)
3379	0.88889	(rh.rostralmiddlefrontal_10)(rh.superiorfrontal_1)
3380	0.88889	(rh.rostralmiddlefrontal_12)(rh.rostralmiddlefrontal_5)(rh.rostralmiddlefrontal_8)
3381	0.88889	(rh.superiorfrontal_2)(rh.superiorfrontal_5)(rh.superiorfrontal_7)(rh.superiorfrontal_8)
3382	0.88889	(rh.superiorfrontal_2)(rh.superiorfrontal_5)(rh.superiorfrontal_9)
3383	0.88889	(rh.superiorfrontal_4)(rh.superiorfrontal_6)(rh.superiorfrontal_7)
3384	0.88889	(rh.supramarginal_1)(rh.supramarginal_6)(rh.supramarginal_8)
3385	0.88889	(Right-Caudate)(rh.caudalmiddlefrontal_1)(rh.caudalmiddlefrontal_4)
3386	0.88889	(Right-Caudate)(rh.lateralorbitofrontal_5)(rh.medialorbitofrontal_2)
3387	0.88889	(Right-Caudate)(rh.medialorbitofrontal_2)(rh.superiorfrontal_2)
3388	0.88889	(Right-Caudate)(rh.supramarginal_8)
3389	0.88889	(Right-Caudate)(Right-Putamen)(rh.caudalanteriorcingulate_2)
3390	0.88889	(Right-Caudate)(Right-Putamen)(rh.posteriorcingulate_1)(rh.posteriorcingulate_2)
3391	0.88889	(Right-Caudate)(Right-Putamen)(rh.precentral_11)(rh.precentral_7)
3392	0.88889	(Right-Caudate)(Right-Putamen)(rh.rostralmiddlefrontal_3)
3393	0.88889	(Right-Hippocampus)(rh.supramarginal_8)(rh.supramarginal_9)
3394	0.88889	(Right-Putamen)(rh.caudalanteriorcingulate_2)
3395	0.88889	(Right-Putamen)(rh.lateralorbitofrontal_2)(rh.parstriangularis_1)(rh.rostralmiddlefrontal_8)
3396	0.88889	(Right-Putamen)(rh.posteriorcingulate_1)(rh.posteriorcingulate_2)
3397	0.88889	(Right-Putamen)(Right-Thalamus-Proper)(rh.precentral_11)(rh.precentral_13)
3398	0.88647	(Left-Accumbens-area)(Left-Hippocampus)(Left-Thalamus-Proper)
3399	0.88647	(Left-Accumbens-area)(Left-Hippocampus)
3400	0.88647	(Left-Caudate)(Left-Putamen)(lh.caudalanteriorcingulate_2)(lh.superiorfrontal_11)(lh.superiorfrontal_12)

3401	0.88647	(Left-Caudate)(Left-Putamen)(lh.caudalmiddlefrontal_3)(lh.superiorfrontal_12)
3402	0.88647	(Left-Caudate)(Left-Putamen)(lh.insula_6)(lh.parsopercularis_3)(lh.rostralmiddlefrontal_1)
3403	0.88647	(Left-Caudate)(Left-Putamen)(lh.insula_6)(lh.rostralmiddlefrontal_9)
3404	0.88647	(Left-Caudate)(Left-Putamen)(lh.lateralorbitofrontal_2)(lh.medialorbitofrontal_2)
3405	0.88647	(Left-Caudate)(Left-Putamen)(lh.rostralanteriorcingulate_1)(lh.rostralanteriorcingulate_2)(lh.rostralmiddlefrontal_9)
3406	0.88647	(Left-Caudate)(Left-Putamen)(lh.rostralmiddlefrontal_4)(lh.superiorfrontal_11)
3407	0.88647	(Left-Caudate)(Left-Thalamus-Proper)(lh.precentral_11)(lh.precentral_4)(lh.precentral_7)
3408	0.88647	(Left-Caudate)(lh.caudalanteriorcingulate_2)(lh.caudalmiddlefrontal_1)(lh.caudalmiddlefrontal_3)
3409	0.88647	(Left-Caudate)(lh.caudalanteriorcingulate_2)(lh.superiorfrontal_11)(lh.superiorfrontal_14)(lh.superiorfrontal_8)
3410	0.88647	(Left-Caudate)(lh.insula_1)(lh.precentral_7)
3411	0.88647	(Left-Caudate)(lh.lateralorbitofrontal_3)
3412	0.88647	(Left-Caudate)(lh.lateralorbitofrontal_6)(lh.lateralorbitofrontal_7)
3413	0.88647	(Left-Caudate)(lh.postcentral_8)(lh.precentral_11)
3414	0.88647	(Left-Caudate)(lh.precentral_6)
3415	0.88647	(Left-Hippocampus)(Left-Thalamus-Proper)(lh.superiortemporal_2)
3416	0.88647	(Left-Hippocampus)(lh.lateraloccipital_9)(lh.lingual_8)
3417	0.88647	(Left-Putamen)(lh.lateralorbitofrontal_2)(lh.lateralorbitofrontal_7)(lh.medialorbitofrontal_2)
3418	0.88647	(Left-Putamen)(lh.lateralorbitofrontal_4)(lh.lateralorbitofrontal_6)(lh.lateralorbitofrontal_7)(lh.rostralmiddlefrontal_9)
3419	0.88647	(Left-Putamen)(lh.lateralorbitofrontal_7)(lh.medialorbitofrontal_2)(lh.rostralanteriorcingulate_1)(lh.rostralmiddlefrontal_12)
3420	0.88647	(Left-Putamen)(lh.precentral_11)(lh.precentral_7)(lh.precentral_9)
3421	0.88647	(Left-Thalamus-Proper)(lh.insula_1)(lh.postcentral_8)
3422	0.88647	(Left-Thalamus-Proper)(lh.superiortemporal_2)
3423	0.88647	(lh.bankssts_2)(lh.fusiform_4)
3424	0.88647	(lh.bankssts_2)(lh.inferiortemporal_3)
3425	0.88647	(lh.bankssts_3)(lh.fusiform_7)
3426	0.88647	(lh.fusiform_1)(lh.pericalcarine_3)
3427	0.88647	(lh.fusiform_2)(lh.lingual_1)(lh.lingual_8)
3428	0.88647	(lh.fusiform_4)(lh.inferiortemporal_7)
3429	0.88647	(lh.fusiform_4)(lh.lateraloccipital_9)
3430	0.88647	(lh.fusiform_7)(lh.fusiform_8)
3431	0.88647	(lh.inferiorparietal_9)(lh.superiorparietal_5)
3432	0.88647	(lh.inferiortemporal_7)(lh.middletemporal_3)
3433	0.88647	(lh.insula_1)(lh.supramarginal_2)(lh.supramarginal_4)
3434	0.88647	(lh.insula_6)(lh.parstriangularis_3)(lh.rostralmiddlefrontal_1)(lh.rostralmiddlefrontal_9)
3435	0.88647	(lh.isthmuscingulate_2)(lh.posteriorcingulate_4)
3436	0.88647	(lh.lateraloccipital_5)(lh.pericalcarine_2)
3437	0.88647	(lh.lateralorbitofrontal_6)(lh.parstriangularis_3)(lh.rostralmiddlefrontal_1)(lh.rostralmiddlefrontal_6)(lh.rostralmiddlefrontal_9)
3438	0.88647	(lh.lateralorbitofrontal_7)(lh.medialorbitofrontal_2)(lh.rostralanteriorcingulate_1)(lh.rostralmiddlefrontal_9)
3439	0.88647	(lh.paracentral_5)(lh.precentral_4)(lh.superiorfrontal_14)
3440	0.88647	(lh.pericalcarine_1)(lh.pericalcarine_3)(lh.superiorparietal_12)
3441	0.88647	(lh.postcentral_8)(lh.precentral_9)
3442	0.88647	(lh.rostralanteriorcingulate_1)(lh.rostralanteriorcingulate_2)(lh.rostralmiddlefrontal_4)
3443	0.88647	(lh.rostralmiddlefrontal_1)(lh.rostralmiddlefrontal_12)(lh.rostralmiddlefrontal_9)
3444	0.88647	(lh.rostralmiddlefrontal_8)(lh.rostralmiddlefrontal_9)(lh.superiorfrontal_3)(lh.superiorfrontal_4)
3445	0.88647	(lh.superiorfrontal_10)(lh.superiorfrontal_11)(lh.superiorfrontal_8)(lh.superiorfrontal_9)
3446	0.88647	(lh.superiorfrontal_11)(lh.superiorfrontal_14)(lh.superiorfrontal_7)(lh.superiorfrontal_9)
3447	0.88647	(rh.caudalmiddlefrontal_4)(rh.caudalmiddlefrontal_5)(rh.superiorfrontal_7)
3448	0.88647	(rh.caudalmiddlefrontal_4)(rh.superiorfrontal_13)(rh.superiorfrontal_7)
3449	0.88647	(rh.fusiform_2)(rh.lateraloccipital_10)(rh.lingual_7)
3450	0.88647	(rh.fusiform_7)(rh.parahippocampal_2)

3451	0.88647	(rh.inferiorparietal_11)(rh.lateraloccipital_4)
3452	0.88647	(rh.inferiorparietal_4)(rh.supramarginal_3)
3453	0.88647	(rh.insula_1)(rh.precentral_11)(rh.supramarginal_1)
3454	0.88647	(rh.insula_2)(rh.insula_3)(rh.supramarginal_9)
3455	0.88647	(rh.isthmuscingulate_2)(rh.precuneus_2)(rh.precuneus_7)
3456	0.88647	(rh.lateraloccipital_10)(rh.lateraloccipital_8)(rh.lingual_5)
3457	0.88647	(rh.lateralorbitofrontal_2)(rh.rostralmiddlefrontal_12)(rh.rostralmiddlefrontal_8)
3458	0.88647	(rh.lateralorbitofrontal_6)(rh.medialorbitofrontal_2)(rh.rostralmiddlefrontal_13)
3459	0.88647	(rh.lateralorbitofrontal_6)(rh.parsorbitalis_1)(rh.parstriangularis_1)
3460	0.88647	(rh.paracentral_6)(rh.superiorparietal_1)
3461	0.88647	(rh.precentral_13)(rh.precentral_14)(rh.precentral_15)
3462	0.88647	(rh.precuneus_10)(rh.superiorparietal_1)(rh.superiorparietal_2)
3463	0.88647	(rh.rostralmiddlefrontal_10)(rh.rostralmiddlefrontal_11)
3464	0.88647	(rh.superiorfrontal_11)(rh.superiorfrontal_13)(rh.superiorfrontal_16)
3465	0.88647	(rh.superiorfrontal_13)(rh.superiorfrontal_15)
3466	0.88647	(Right-Amygdala)(Right-Hippocampus)(rh.insula_4)
3467	0.88647	(Right-Amygdala)(Right-Hippocampus)(Right-Putamen)(rh.insula_4)
3468	0.88647	(Right-Caudate)(rh.caudalmiddlefrontal_4)(rh.rostralmiddlefrontal_1)
3469	0.88647	(Right-Caudate)(rh.caudalmiddlefrontal_4)(rh.rostralmiddlefrontal_3)
3470	0.88647	(Right-Caudate)(Right-Putamen)(rh.lateralorbitofrontal_5)(rh.lateralorbitofrontal_7)
3471	0.88647	(Right-Caudate)(Right-Putamen)(rh.precentral_11)(rh.precentral_13)(rh.precentral_7)
3472	0.88647	(Right-Hippocampus)(rh.insula_1)
3473	0.88647	(Right-Hippocampus)(rh.lateraloccipital_10)
3474	0.88647	(Right-Hippocampus)(Right-Putamen)(rh.insula_1)
3475	0.88647	(Right-Putamen)(rh.insula_1)(rh.supramarginal_8)(rh.supramarginal_9)
3476	0.88647	(Right-Putamen)(rh.insula_1)(rh.supramarginal_8)
3477	0.88647	(Right-Putamen)(rh.insula_2)(rh.insula_3)(rh.supramarginal_9)
3478	0.88406	(Left-Accumbens-area)(Left-Amygdala)(Left-Pallidum)(Left-Putamen)(Left-Thalamus-Proper)
3479	0.88406	(Left-Accumbens-area)(Left-Amygdala)(Left-Pallidum)(Left-Putamen)
3480	0.88406	(Left-Accumbens-area)(Left-Amygdala)(Left-Putamen)(Left-Thalamus-Proper)
3481	0.88406	(Left-Accumbens-area)(Left-Amygdala)(Left-Putamen)
3482	0.88406	(Left-Accumbens-area)(Left-Caudate)(lh.medialorbitofrontal_4)
3483	0.88406	(Left-Caudate)(Left-Putamen)(Left-Thalamus-Proper)(lh.precentral_11)(lh.precentral_4)(lh.precentral_7)
3484	0.88406	(Left-Caudate)(Left-Putamen)(lh.caudalmiddlefrontal_3)(lh.rostralmiddlefrontal_1)(lh.rostralmiddlefrontal_4)
3485	0.88406	(Left-Caudate)(Left-Putamen)(lh.insula_1)(lh.precentral_7)
3486	0.88406	(Left-Caudate)(Left-Putamen)(lh.insula_6)(lh.parstriangularis_3)(lh.rostralmiddlefrontal_1)
3487	0.88406	(Left-Caudate)(Left-Putamen)(lh.parstriangularis_3)(lh.rostralmiddlefrontal_4)(lh.rostralmiddlefrontal_9)
3488	0.88406	(Left-Caudate)(Left-Putamen)(lh.postcentral_8)(lh.precentral_11)
3489	0.88406	(Left-Caudate)(Left-Putamen)(lh.rostralanteriorcingulate_1)(lh.rostralmiddlefrontal_4)(lh.rostralmiddlefrontal_9)
3490	0.88406	(Left-Caudate)(Left-Putamen)(lh.superiorfrontal_12)(lh.superiorfrontal_14)
3491	0.88406	(Left-Caudate)(Left-Thalamus-Proper)(lh.paracentral_5)(lh.precentral_4)
3492	0.88406	(Left-Caudate)(lh.caudalanteriorcingulate_2)(lh.caudalmiddlefrontal_5)
3493	0.88406	(Left-Caudate)(lh.medialorbitofrontal_2)(lh.rostralanteriorcingulate_1)(lh.superiorfrontal_4)
3494	0.88406	(Left-Caudate)(lh.rostralmiddlefrontal_4)(lh.superiorfrontal_11)(lh.superiorfrontal_4)
3495	0.88406	(Left-Hippocampus)(lh.inferiorparietal_4)
3496	0.88406	(Left-Putamen)(Left-Thalamus-Proper)(lh.insula_1)(lh.postcentral_8)
3497	0.88406	(Left-Putamen)(lh.caudalmiddlefrontal_3)(lh.parstriangularis_3)
3498	0.88406	(Left-Putamen)(lh.lateralorbitofrontal_3)(lh.lateralorbitofrontal_4)(lh.lateralorbitofrontal_6)
3499	0.88406	(Left-Putamen)(lh.medialorbitofrontal_2)(lh.rostralmiddlefrontal_12)(lh.superiorfrontal_3)
3500	0.88406	(Left-Putamen)(lh.medialorbitofrontal_2)(lh.rostralmiddlefrontal_9)(lh.superiorfrontal_3)

3501	0.88406	(Left-Putamen)(lh.superiorfrontal_3)(lh.superiorfrontal_4)
3502	0.88406	(lh.caudalanteriorcingulate_1)(lh.rostralmiddlefrontal_4)
3503	0.88406	(lh.entorhinal_1)(lh.parahippocampal_1)
3504	0.88406	(lh.fusiform_2)(lh.lateraloccipital_10)(lh.lateraloccipital_6)
3505	0.88406	(lh.fusiform_2)(lh.lateraloccipital_6)(lh.lateraloccipital_9)
3506	0.88406	(lh.fusiform_7)(lh.middletemporal_3)
3507	0.88406	(lh.inferiorparietal_1)(lh.pericalcarine_3)(lh.precuneus_11)
3508	0.88406	(lh.inferiorparietal_8)(lh.superiorparietal_5)
3509	0.88406	(lh.insula_1)(lh.supramarginal_6)
3510	0.88406	(lh.insula_2)(lh.superiortemporal_2)
3511	0.88406	(lh.insula_6)(lh.lateralorbitofrontal_6)
3512	0.88406	(lh.isthmuscingulate_2)(lh.isthmuscingulate_3)(lh.precuneus_10)(lh.precuneus_11)
3513	0.88406	(lh.lateraloccipital_1)(lh.lateraloccipital_2)(lh.pericalcarine_1)
3514	0.88406	(lh.lateraloccipital_3)(lh.lateraloccipital_5)
3515	0.88406	(lh.lateraloccipital_3)(lh.lateraloccipital_6)(lh.lingual_1)
3516	0.88406	(lh.lateraloccipital_3)(lh.lateraloccipital_6)(lh.pericalcarine_2)
3517	0.88406	(lh.lateralorbitofrontal_6)(lh.lateralorbitofrontal_7)(lh.rostralmiddlefrontal_6)
3518	0.88406	(lh.lateralorbitofrontal_6)(lh.parsorbitalis_1)(lh.parstriangularis_1)(lh.parstriangularis_3)
3519	0.88406	(lh.lateralorbitofrontal_7)(lh.medialorbitofrontal_5)
3520	0.88406	(lh.lateralorbitofrontal_7)(lh.parstriangularis_3)(lh.rostralanteriorcingulate_1)(lh.rostralmiddlefrontal_12)
3521	0.88406	(lh.lateralorbitofrontal_7)(lh.rostralanteriorcingulate_1)(lh.rostralmiddlefrontal_12)(lh.rostralmiddlefrontal_9)
3522	0.88406	(lh.medialorbitofrontal_2)(lh.parstriangularis_3)(lh.rostralmiddlefrontal_9)
3523	0.88406	(lh.medialorbitofrontal_2)(lh.rostralanteriorcingulate_1)(lh.superiorfrontal_3)(lh.superiorfrontal_4)
3524	0.88406	(lh.medialorbitofrontal_2)(lh.rostralmiddlefrontal_9)(lh.superiorfrontal_4)
3525	0.88406	(lh.paracentral_5)(lh.posteriorcingulate_3)
3526	0.88406	(lh.parsopercularis_1)(lh.parsopercularis_2)(lh.parstriangularis_3)(lh.rostralmiddlefrontal_1)
3527	0.88406	(lh.postcentral_3)(lh.superiorparietal_3)
3528	0.88406	(lh.postcentral_8)(lh.precentral_6)
3529	0.88406	(lh.precentral_11)(lh.precentral_6)
3530	0.88406	(lh.precuneus_9)(lh.superiorparietal_11)(lh.superiorparietal_6)
3531	0.88406	(lh.rostralmiddlefrontal_8)(lh.superiorfrontal_2)(lh.superiorfrontal_3)(lh.superiorfrontal_4)
3532	0.88406	(lh.rostralmiddlefrontal_9)(lh.superiorfrontal_2)(lh.superiorfrontal_3)
3533	0.88406	(lh.superiorfrontal_11)(lh.superiorfrontal_13)(lh.superiorfrontal_8)
3534	0.88406	(lh.superiorfrontal_12)(lh.superiorfrontal_17)
3535	0.88406	(lh.superiorfrontal_12)(lh.superiorfrontal_4)
3536	0.88406	(lh.superiorfrontal_3)(lh.superiorfrontal_5)(lh.superiorfrontal_6)
3537	0.88406	(rh.caudalmiddlefrontal_3)(rh.precentral_10)
3538	0.88406	(rh.fusiform_1)(rh.fusiform_2)(rh.lingual_5)(rh.lingual_7)
3539	0.88406	(rh.fusiform_2)(rh.lateraloccipital_8)(rh.lingual_5)
3540	0.88406	(rh.fusiform_7)(rh.inferiortemporal_2)
3541	0.88406	(rh.fusiform_8)(rh.middletemporal_9)
3542	0.88406	(rh.inferiorparietal_12)(rh.inferiorparietal_7)(rh.inferiorparietal_8)
3543	0.88406	(rh.inferiortemporal_2)(rh.middletemporal_5)(rh.middletemporal_7)
3544	0.88406	(rh.inferiortemporal_2)(rh.superiortemporal_7)
3545	0.88406	(rh.insula_1)(rh.superiorparietal_1)(rh.supramarginal_8)
3546	0.88406	(rh.lateraloccipital_8)(rh.lingual_4)
3547	0.88406	(rh.lateralorbitofrontal_7)(rh.medialorbitofrontal_2)(rh.rostralmiddlefrontal_11)(rh.superiorfrontal_2)
3548	0.88406	(rh.middletemporal_4)(rh.superiortemporal_6)
3549	0.88406	(rh.paracentral_6)(rh.precentral_13)(rh.precentral_15)
3550	0.88406	(rh.parsopercularis_4)(rh.parstriangularis_1)(rh.rostralmiddlefrontal_3)

3551	0.88406	(rh.parstriangularis_1)(rh.parstriangularis_4)(rh.rostralmiddlefrontal_3)(rh.rostralmiddlefrontal_8)
3552	0.88406	(rh.postcentral_8)(rh.postcentral_9)
3553	0.88406	(rh.precentral_10)(rh.precentral_13)(rh.precentral_8)
3554	0.88406	(rh.precuneus_10)(rh.precuneus_7)(rh.superiorparietal_1)
3555	0.88406	(rh.precuneus_4)(rh.precuneus_7)(rh.superiorparietal_10)(rh.superiorparietal_7)
3556	0.88406	(rh.precuneus_4)(rh.precuneus_8)
3557	0.88406	(rh.precuneus_4)(rh.superiorparietal_1)(rh.superiorparietal_10)
3558	0.88406	(rh.rostralmiddlefrontal_10)(rh.superiorfrontal_1)(rh.superiorfrontal_2)
3559	0.88406	(rh.rostralmiddlefrontal_11)(rh.rostralmiddlefrontal_12)(rh.superiorfrontal_2)
3560	0.88406	(rh.rostralmiddlefrontal_2)(rh.rostralmiddlefrontal_5)
3561	0.88406	(rh.rostralmiddlefrontal_8)(rh.superiorfrontal_7)
3562	0.88406	(rh.rostralmiddlefrontal_9)(rh.superiorfrontal_5)(rh.superiorfrontal_7)
3563	0.88406	(rh.superiorfrontal_4)(rh.superiorfrontal_5)(rh.superiorfrontal_6)(rh.superiorfrontal_7)
3564	0.88406	(rh.superiorparietal_1)(rh.superiorparietal_8)
3565	0.88406	(Right-Amygdala)(rh.insula_2)
3566	0.88406	(Right-Amygdala)(Right-Putamen)(rh.insula_2)
3567	0.88406	(Right-Amygdala)(Right-Putamen)(Right-Thalamus-Proper)(rh.insula_2)
3568	0.88406	(Right-Amygdala)(Right-Thalamus-Proper)(rh.insula_2)
3569	0.88406	(Right-Caudate)(rh.lateralorbitofrontal_4)(rh.lateralorbitofrontal_7)
3570	0.88406	(Right-Caudate)(rh.precentral_5)(rh.precentral_7)
3571	0.88406	(Right-Caudate)(rh.superiorfrontal_2)(rh.superiorfrontal_5)
3572	0.88406	(Right-Caudate)(Right-Putamen)(rh.lateralorbitofrontal_4)(rh.lateralorbitofrontal_7)
3573	0.88406	(Right-Caudate)(Right-Putamen)(rh.rostralanteriorcingulate_1)(rh.rostralanteriorcingulate_2)
3574	0.88406	(Right-Hippocampus)(rh.inferiorparietal_9)
3575	0.88406	(Right-Hippocampus)(rh.isthmuscingulate_1)(rh.isthmuscingulate_2)
3576	0.88406	(Right-Hippocampus)(rh.isthmuscingulate_2)(rh.lingual_7)(rh.precuneus_2)
3577	0.88406	(Right-Hippocampus)(rh.parahippocampal_2)(rh.parahippocampal_3)
3578	0.88406	(Right-Putamen)(rh.caudalmiddlefrontal_2)
3579	0.88406	(Right-Putamen)(rh.caudalmiddlefrontal_4)(rh.parsopercularis_4)(rh.rostralmiddlefrontal_3)
3580	0.88406	(Right-Putamen)(rh.insula_3)(rh.precentral_10)(rh.precentral_7)
3581	0.88406	(Right-Putamen)(rh.rostralanteriorcingulate_1)(rh.rostralanteriorcingulate_2)
3582	0.88164	(Left-Amygdala)(Left-Hippocampus)(lh.inferiortemporal_1)
3583	0.88164	(Left-Caudate)(Left-Putamen)(lh.lateralorbitofrontal_4)(lh.lateralorbitofrontal_7)
3584	0.88164	(Left-Caudate)(Left-Putamen)(lh.parsopercularis_3)(lh.parstriangularis_3)
3585	0.88164	(Left-Caudate)(Left-Putamen)(lh.parstriangularis_3)(lh.rostralmiddlefrontal_1)(lh.rostralmiddlefrontal_4)
3586	0.88164	(Left-Caudate)(Left-Thalamus-Proper)(Right-Thalamus-Proper)
3587	0.88164	(Left-Caudate)(lh.caudalanteriorcingulate_2)(lh.superiorfrontal_11)(lh.superiorfrontal_4)
3588	0.88164	(Left-Caudate)(lh.caudalmiddlefrontal_1)(lh.caudalmiddlefrontal_4)(lh.caudalmiddlefrontal_5)
3589	0.88164	(Left-Caudate)(lh.lateralorbitofrontal_4)(lh.lateralorbitofrontal_7)
3590	0.88164	(Left-Caudate)(lh.lateralorbitofrontal_7)(lh.rostralanteriorcingulate_1)(lh.rostralmiddlefrontal_9)
3591	0.88164	(Left-Caudate)(lh.lateralorbitofrontal_7)(lh.rostralmiddlefrontal_12)(lh.rostralmiddlefrontal_9)
3592	0.88164	(Left-Caudate)(Right-Caudate)(lh.caudalanteriorcingulate_1)(lh.caudalanteriorcingulate_2)
3593	0.88164	(Left-Caudate)(Right-Caudate)(lh.caudalanteriorcingulate_1)(lh.rostralanteriorcingulate_1)
3594	0.88164	(Left-Caudate)(Right-Caudate)(Right-Thalamus-Proper)
3595	0.88164	(Left-Caudate)(Right-Thalamus-Proper)
3596	0.88164	(Left-Putamen)(lh.caudalanteriorcingulate_1)(lh.caudalanteriorcingulate_2)(lh.rostralanteriorcingulate_1)(lh.rostralanteriorcingulate_2)
3597	0.88164	(Left-Putamen)(lh.lateralorbitofrontal_4)(lh.lateralorbitofrontal_6)(lh.parstriangularis_3)(lh.rostralmiddlefrontal_12)(lh.rostralmiddlefrontal_9)
3598	0.88164	(Left-Putamen)(lh.lateralorbitofrontal_4)(lh.lateralorbitofrontal_7)(lh.parstriangularis_3)(lh.rostralmiddlefrontal_12)
3599	0.88164	(Left-Putamen)(lh.lateralorbitofrontal_7)(lh.parstriangularis_3)(lh.rostralanteriorcingulate_1)
3600	0.88164	(Left-Putamen)(lh.precentral_13)

3601	0.88164	(Left-Putamen)(lh.precentral_6)
3602	0.88164	(Left-Putamen)(lh.rostralanteriorcingulate_1)(lh.rostralmiddlefrontal_9)(lh.superiorfrontal_3)
3603	0.88164	(Left-Putamen)(lh.rostralanteriorcingulate_1)(lh.rostralmiddlefrontal_9)(lh.superiorfrontal_4)
3604	0.88164	(lh.caudalmiddlefrontal_1)(lh.rostralmiddlefrontal_1)
3605	0.88164	(lh.caudalmiddlefrontal_2)(lh.caudalmiddlefrontal_5)
3606	0.88164	(lh.caudalmiddlefrontal_5)(lh.superiorfrontal_12)
3607	0.88164	(lh.fusiform_1)(lh.lingual_6)
3608	0.88164	(lh.fusiform_2)(lh.lateraloccipital_6)(lh.lingual_4)
3609	0.88164	(lh.fusiform_7)(lh.inferiortemporal_2)
3610	0.88164	(lh.inferiorparietal_1)(lh.inferiorparietal_6)
3611	0.88164	(lh.inferiorparietal_8)(lh.precuneus_9)
3612	0.88164	(lh.inferiortemporal_1)(lh.insula_4)
3613	0.88164	(lh.inferiortemporal_6)(lh.inferiortemporal_7)(lh.inferiortemporal_8)
3614	0.88164	(lh.insula_1)(lh.postcentral_8)(lh.precentral_11)
3615	0.88164	(lh.isthmuscingulate_1)(lh.isthmuscingulate_2)
3616	0.88164	(lh.isthmuscingulate_3)(lh.lingual_7)
3617	0.88164	(lh.isthmuscingulate_3)(lh.precuneus_10)(lh.precuneus_11)(lh.precuneus_6)
3618	0.88164	(lh.lateralorbitofrontal_4)(lh.lateralorbitofrontal_7)(lh.rostralmiddlefrontal_12)(lh.rostralmiddlefrontal_9)
3619	0.88164	(lh.lingual_1)(lh.lingual_4)(lh.lingual_8)
3620	0.88164	(lh.medialorbitofrontal_3)(lh.medialorbitofrontal_4)
3621	0.88164	(lh.paracentral_1)(lh.precentral_2)(lh.precentral_3)
3622	0.88164	(lh.parsopercularis_1)(lh.parsopercularis_2)(lh.parsopercularis_3)
3623	0.88164	(lh.parstriangularis_3)(lh.rostralmiddlefrontal_1)(lh.rostralmiddlefrontal_12)
3624	0.88164	(lh.rostralanteriorcingulate_2)(lh.rostralmiddlefrontal_4)(lh.rostralmiddlefrontal_9)
3625	0.88164	(lh.rostralmiddlefrontal_12)(lh.superiorfrontal_3)(lh.superiorfrontal_4)
3626	0.88164	(lh.superiorfrontal_10)(lh.superiorfrontal_11)(lh.superiorfrontal_14)(lh.superiorfrontal_8)
3627	0.88164	(lh.superiorfrontal_10)(lh.superiorfrontal_11)(lh.superiorfrontal_14)
3628	0.88164	(lh.superiorfrontal_10)(lh.superiorfrontal_11)(lh.superiorfrontal_6)
3629	0.88164	(lh.superiorfrontal_10)(lh.superiorfrontal_4)(lh.superiorfrontal_5)(lh.superiorfrontal_7)
3630	0.88164	(lh.superiorfrontal_10)(lh.superiorfrontal_5)(lh.superiorfrontal_7)
3631	0.88164	(lh.superiorfrontal_11)(lh.superiorfrontal_4)(lh.superiorfrontal_7)(lh.superiorfrontal_9)
3632	0.88164	(lh.superiorfrontal_12)(lh.superiorfrontal_14)(lh.superiorfrontal_17)
3633	0.88164	(lh.superiorfrontal_3)(lh.superiorfrontal_4)(lh.superiorfrontal_5)(lh.superiorfrontal_6)
3634	0.88164	(lh.superiorparietal_3)(lh.supramarginal_1)
3635	0.88164	(lh.superiortemporal_4)(lh.superiortemporal_6)
3636	0.88164	(lh.superiortemporal_6)(lh.superiortemporal_7)
3637	0.88164	(lh.superiortemporal_8)(lh.superiortemporal_9)
3638	0.88164	(rh.bankssts_1)(rh.inferiortemporal_5)(rh.inferiortemporal_6)
3639	0.88164	(rh.bankssts_2)(rh.inferiortemporal_6)
3640	0.88164	(rh.caudalmiddlefrontal_4)(rh.caudalmiddlefrontal_5)(rh.rostralmiddlefrontal_2)
3641	0.88164	(rh.caudalmiddlefrontal_4)(rh.precentral_13)
3642	0.88164	(rh.caudalmiddlefrontal_4)(rh.rostralmiddlefrontal_1)(rh.superiorfrontal_7)
3643	0.88164	(rh.cuneus_3)(rh.pericalcarine_2)(rh.superiorparietal_13)
3644	0.88164	(rh.fusiform_1)(rh.lateraloccipital_10)(rh.lateraloccipital_8)(rh.lingual_5)
3645	0.88164	(rh.fusiform_3)(rh.fusiform_4)(rh.fusiform_6)
3646	0.88164	(rh.fusiform_3)(rh.inferiortemporal_6)
3647	0.88164	(rh.inferiorparietal_1)(rh.inferiorparietal_3)(rh.inferiorparietal_4)
3648	0.88164	(rh.inferiorparietal_10)(rh.lateraloccipital_8)(rh.lingual_7)
3649	0.88164	(rh.inferiortemporal_2)(rh.inferiortemporal_4)(rh.middletemporal_4)
3650	0.88164	(rh.inferiortemporal_2)(rh.superiortemporal_3)

3651	0.88164	(rh.insula_1)(rh.postcentral_1)(rh.supramarginal_9)
3652	0.88164	(rh.insula_1)(rh.supramarginal_7)
3653	0.88164	(rh.insula_6)(rh.lateralorbitofrontal_6)
3654	0.88164	(rh.lateraloccipital_8)(rh.superiorparietal_11)
3655	0.88164	(rh.lateralorbitofrontal_2)(rh.lateralorbitofrontal_7)(rh.parstriangularis_1)(rh.rostralmiddlefrontal_12)
3656	0.88164	(rh.middletemporal_4)(rh.middletemporal_6)(rh.middletemporal_7)
3657	0.88164	(rh.parsopercularis_2)(rh.rostralmiddlefrontal_3)
3658	0.88164	(rh.parsopercularis_4)(rh.parstriangularis_1)(rh.parstriangularis_2)
3659	0.88164	(rh.parstriangularis_4)(rh.rostralmiddlefrontal_12)(rh.rostralmiddlefrontal_3)
3660	0.88164	(rh.posteriorcingulate_4)(rh.supramarginal_8)
3661	0.88164	(rh.precentral_10)(rh.precentral_7)(rh.precentral_8)
3662	0.88164	(rh.precentral_13)(rh.superiorfrontal_16)
3663	0.88164	(rh.rostralmiddlefrontal_1)(rh.rostralmiddlefrontal_8)
3664	0.88164	(rh.superiorfrontal_12)(rh.superiorfrontal_15)(rh.superiorfrontal_16)
3665	0.88164	(rh.superiorfrontal_2)(rh.superiorfrontal_7)(rh.superiorfrontal_9)
3666	0.88164	(Right-Amygdala)(Right-Hippocampus)(rh.insula_2)
3667	0.88164	(Right-Amygdala)(Right-Hippocampus)(Right-Putamen)(rh.insula_2)
3668	0.88164	(Right-Amygdala)(Right-Hippocampus)(Right-Putamen)(Right-Thalamus-Proper)(rh.insula_2)
3669	0.88164	(Right-Amygdala)(Right-Hippocampus)(Right-Thalamus-Proper)(rh.insula_2)
3670	0.88164	(Right-Caudate)(lh.caudalanteriorcingulate_1)(lh.caudalanteriorcingulate_2)
3671	0.88164	(Right-Caudate)(lh.caudalanteriorcingulate_1)(lh.rostralanteriorcingulate_1)
3672	0.88164	(Right-Caudate)(rh.parstriangularis_1)(rh.rostralmiddlefrontal_8)
3673	0.88164	(Right-Caudate)(rh.superiorfrontal_14)
3674	0.88164	(Right-Caudate)(Right-Putamen)(rh.precentral_5)(rh.precentral_7)
3675	0.88164	(Right-Caudate)(Right-Putamen)(rh.superiorfrontal_13)
3676	0.88164	(Right-Caudate)(Right-Thalamus-Proper)(rh.precentral_11)(rh.precentral_13)
3677	0.88164	(Right-Hippocampus)(rh.inferiortemporal_1)(rh.insula_5)
3678	0.88164	(Right-Hippocampus)(Right-Putamen)(Right-Thalamus-Proper)(rh.insula_1)
3679	0.88164	(Right-Hippocampus)(Right-Thalamus-Proper)(rh.insula_1)
3680	0.88164	(Right-Putamen)(rh.insula_6)(rh.lateralorbitofrontal_6)
3681	0.88164	(Right-Putamen)(rh.lateralorbitofrontal_2)(rh.rostralmiddlefrontal_12)(rh.rostralmiddlefrontal_8)
3682	0.88164	(Right-Putamen)(rh.precentral_10)(rh.precentral_8)
3683	0.88164	(Right-Putamen)(rh.precentral_13)(rh.superiorparietal_1)
3684	0.88164	(Right-Putamen)(rh.rostralmiddlefrontal_10)
3685	0.88164	(Right-Putamen)(rh.supramarginal_1)(rh.supramarginal_8)
3686	0.87923	(Left-Accumbens-area)(Left-Hippocampus)(Left-Pallidum)(Left-Thalamus-Proper)
3687	0.87923	(Left-Accumbens-area)(Left-Hippocampus)(Left-Pallidum)
3688	0.87923	(Left-Accumbens-area)(Left-Hippocampus)(Left-Putamen)(Left-Thalamus-Proper)
3689	0.87923	(Left-Accumbens-area)(Left-Hippocampus)(Left-Putamen)
3690	0.87923	(Left-Amygdala)(lh.superiortemporal_11)
3691	0.87923	(Left-Caudate)(Left-Putamen)(lh.caudalanteriorcingulate_1)(lh.caudalanteriorcingulate_2)(lh.rostralanteriorcingulate_1)(lh.rostralanteriorcingulate_2)
3692	0.87923	(Left-Caudate)(Left-Putamen)(lh.lateralorbitofrontal_3)
3693	0.87923	(Left-Caudate)(Left-Putamen)(lh.lateralorbitofrontal_4)(lh.parstriangularis_3)
3694	0.87923	(Left-Caudate)(Left-Putamen)(lh.parstriangularis_3)(lh.rostralmiddlefrontal_12)
3695	0.87923	(Left-Caudate)(Left-Putamen)(lh.rostralmiddlefrontal_9)(lh.superiorfrontal_4)
3696	0.87923	(Left-Caudate)(lh.caudalmiddlefrontal_1)(lh.superiorfrontal_11)
3697	0.87923	(Left-Caudate)(lh.caudalmiddlefrontal_3)(lh.superiorfrontal_11)(lh.superiorfrontal_12)
3698	0.87923	(Left-Caudate)(lh.caudalmiddlefrontal_4)(lh.precentral_9)
3699	0.87923	(Left-Caudate)(lh.insula_6)(lh.parsopercularis_3)(lh.parstriangularis_3)
3700	0.87923	(Left-Caudate)(lh.lateralorbitofrontal_2)(lh.medialorbitofrontal_5)

3701	0.87923	(Left-Caudate)(lh.lateralorbitofrontal_6)(lh.parstriangularis_3)(lh.rostralmiddlefrontal_9)
3702	0.87923	(Left-Caudate)(lh.lateralorbitofrontal_7)(lh.parstriangularis_3)(lh.rostralmiddlefrontal_12)
3703	0.87923	(Left-Caudate)(lh.rostralanteriorcingulate_2)(lh.superiorfrontal_8)
3704	0.87923	(Left-Hippocampus)(lh.insula_2)(lh.superiortemporal_2)
3705	0.87923	(Left-Pallidum)(Left-Putamen)(lh.postcentral_8)
3706	0.87923	(Left-Pallidum)(Left-Putamen)(lh.precentral_9)
3707	0.87923	(Left-Pallidum)(lh.postcentral_8)
3708	0.87923	(Left-Pallidum)(lh.precentral_11)(lh.precentral_4)
3709	0.87923	(Left-Pallidum)(lh.precentral_9)
3710	0.87923	(Left-Putamen)(lh.caudalanteriorcingulate_2)(lh.caudalmiddlefrontal_1)
3711	0.87923	(Left-Putamen)(lh.caudalmiddlefrontal_6)(lh.superiorfrontal_12)
3712	0.87923	(Left-Putamen)(lh.insula_1)(lh.insula_3)(lh.precentral_11)
3713	0.87923	(Left-Putamen)(lh.insula_6)(lh.lateralorbitofrontal_6)
3714	0.87923	(Left-Putamen)(lh.lateralorbitofrontal_4)(lh.lateralorbitofrontal_7)(lh.rostralmiddlefrontal_12)(lh.rostralmiddlefrontal_9)
3715	0.87923	(Left-Putamen)(lh.lateralorbitofrontal_7)(lh.rostralanteriorcingulate_1)(lh.rostralmiddlefrontal_12)(lh.rostralmiddlefrontal_9)
3716	0.87923	(Left-Putamen)(lh.postcentral_8)(lh.precentral_9)
3717	0.87923	(Left-Putamen)(lh.precentral_14)(lh.precentral_16)
3718	0.87923	(Left-Putamen)(lh.precentral_6)(lh.precentral_7)
3719	0.87923	(lh.bankssts_2)(lh.fusiform_5)
3720	0.87923	(lh.caudalanteriorcingulate_2)(lh.superiorfrontal_10)(lh.superiorfrontal_4)
3721	0.87923	(lh.caudalmiddlefrontal_3)(lh.parsopercularis_3)
3722	0.87923	(lh.caudalmiddlefrontal_4)(lh.caudalmiddlefrontal_6)(lh.precentral_9)
3723	0.87923	(lh.fusiform_7)(lh.superiortemporal_6)
3724	0.87923	(lh.inferiortemporal_4)(lh.middletemporal_4)
3725	0.87923	(lh.insula_1)(lh.insula_3)(lh.precentral_11)
3726	0.87923	(lh.isthmuscingulate_2)(lh.precuneus_3)
3727	0.87923	(lh.lateraloccipital_6)(lh.lateraloccipital_8)
3728	0.87923	(lh.lateralorbitofrontal_4)(lh.lateralorbitofrontal_6)(lh.lateralorbitofrontal_7)(lh.parstriangularis_3)(lh.rostralmiddlefrontal_9)
3729	0.87923	(lh.lateralorbitofrontal_4)(lh.rostralanteriorcingulate_1)(lh.rostralmiddlefrontal_9)
3730	0.87923	(lh.lateralorbitofrontal_7)(lh.rostralmiddlefrontal_11)
3731	0.87923	(lh.paracentral_1)(lh.paracentral_5)
3732	0.87923	(lh.parstriangularis_3)(lh.rostralmiddlefrontal_1)(lh.rostralmiddlefrontal_12)(lh.rostralmiddlefrontal_9)
3733	0.87923	(lh.postcentral_8)(lh.precentral_11)(lh.precentral_7)
3734	0.87923	(lh.precentral_4)(lh.precentral_6)(lh.precentral_7)
3735	0.87923	(lh.precuneus_8)(lh.superiorparietal_6)
3736	0.87923	(lh.rostralanteriorcingulate_1)(lh.rostralmiddlefrontal_12)(lh.superiorfrontal_4)
3737	0.87923	(lh.rostralmiddlefrontal_12)(lh.rostralmiddlefrontal_9)(lh.superiorfrontal_1)(lh.superiorfrontal_3)
3738	0.87923	(lh.rostralmiddlefrontal_4)(lh.rostralmiddlefrontal_7)
3739	0.87923	(lh.rostralmiddlefrontal_6)(lh.rostralmiddlefrontal_7)
3740	0.87923	(lh.superiorfrontal_10)(lh.superiorfrontal_11)(lh.superiorfrontal_4)(lh.superiorfrontal_6)
3741	0.87923	(lh.superiorfrontal_11)(lh.superiorfrontal_12)(lh.superiorfrontal_4)
3742	0.87923	(lh.superiorfrontal_12)(lh.superiorfrontal_4)(lh.superiorfrontal_8)
3743	0.87923	(lh.superiorfrontal_14)(lh.superiorfrontal_17)(lh.superiorfrontal_18)
3744	0.87923	(lh.superiorparietal_5)(lh.supramarginal_6)(lh.supramarginal_9)
3745	0.87923	(rh.bankssts_1)(rh.bankssts_2)(rh.middletemporal_4)
3746	0.87923	(rh.fusiform_2)(rh.lateraloccipital_8)(rh.lingual_7)
3747	0.87923	(rh.inferiorparietal_12)(rh.precuneus_1)
3748	0.87923	(rh.inferiortemporal_3)(rh.inferiortemporal_5)
3749	0.87923	(rh.insula_1)(rh.precentral_7)(rh.supramarginal_1)
3750	0.87923	(rh.insula_2)(rh.precentral_13)

3751	0.87923	(rh.insula_3)(rh.insula_6)
3752	0.87923	(rh.lateraloccipital_1)(rh.lateraloccipital_3)(rh.superiorparietal_11)
3753	0.87923	(rh.lateraloccipital_10)(rh.lingual_5)(rh.lingual_7)
3754	0.87923	(rh.lateraloccipital_4)(rh.lateraloccipital_6)
3755	0.87923	(rh.lateraloccipital_4)(rh.lingual_2)(rh.pericalcarine_1)
3756	0.87923	(rh.lateraloccipital_4)(rh.pericalcarine_2)
3757	0.87923	(rh.lateralorbitofrontal_6)(rh.rostralmiddlefrontal_8)
3758	0.87923	(rh.paracentral_4)(rh.precentral_13)(rh.precentral_14)
3759	0.87923	(rh.paracentral_4)(rh.superiorfrontal_11)
3760	0.87923	(rh.paracentral_5)(rh.paracentral_6)
3761	0.87923	(rh.paracentral_5)(rh.postcentral_11)
3762	0.87923	(rh.parsopercularis_1)(rh.parsopercularis_4)
3763	0.87923	(rh.precentral_10)(rh.precentral_12)
3764	0.87923	(rh.precuneus_10)(rh.precuneus_8)(rh.superiorparietal_8)
3765	0.87923	(rh.precuneus_2)(rh.precuneus_4)(rh.precuneus_7)
3766	0.87923	(rh.rostralmiddlefrontal_12)(rh.rostralmiddlefrontal_6)
3767	0.87923	(rh.superiortemporal_10)(rh.superiortemporal_6)
3768	0.87923	(Right-Caudate)(rh.caudalmiddlefrontal_4)(rh.precentral_13)
3769	0.87923	(Right-Caudate)(rh.lateralorbitofrontal_6)(rh.lateralorbitofrontal_7)
3770	0.87923	(Right-Caudate)(rh.precentral_6)
3771	0.87923	(Right-Caudate)(rh.superiorfrontal_11)(rh.superiorfrontal_7)
3772	0.87923	(Right-Caudate)(Right-Putamen)(rh.caudalmiddlefrontal_4)(rh.superiorfrontal_11)
3773	0.87923	(Right-Caudate)(Right-Putamen)(rh.lateralorbitofrontal_6)(rh.lateralorbitofrontal_7)
3774	0.87923	(Right-Putamen)(rh.caudalmiddlefrontal_4)(rh.precentral_13)
3775	0.87923	(Right-Putamen)(rh.insula_2)(rh.precentral_13)
3776	0.87923	(Right-Putamen)(rh.insula_3)(rh.insula_6)
3777	0.87923	(Right-Putamen)(rh.precentral_5)(rh.precentral_8)
3778	0.87681	(Left-Caudate)(Left-Pallidum)(Left-Putamen)(lh.insula_1)(lh.precentral_11)
3779	0.87681	(Left-Caudate)(Left-Pallidum)(lh.insula_1)(lh.precentral_11)
3780	0.87681	(Left-Caudate)(Left-Putamen)(Left-Thalamus-Proper)(lh.insula_1)(lh.precentral_7)
3781	0.87681	(Left-Caudate)(Left-Putamen)(lh.caudalmiddlefrontal_4)(lh.precentral_9)
3782	0.87681	(Left-Caudate)(Left-Putamen)(lh.caudalmiddlefrontal_6)(lh.superiorfrontal_12)
3783	0.87681	(Left-Caudate)(Left-Putamen)(lh.lateralorbitofrontal_7)(lh.rostralmiddlefrontal_12)(lh.rostralmiddlefrontal_9)
3784	0.87681	(Left-Caudate)(Left-Putamen)(lh.medialorbitofrontal_2)(lh.rostralanteriorcingulate_1)(lh.rostralmiddlefrontal_12)(lh.rostralmiddlefrontal_9)
3785	0.87681	(Left-Caudate)(Left-Thalamus-Proper)(lh.insula_1)(lh.precentral_7)
3786	0.87681	(Left-Caudate)(lh.caudalanteriorcingulate_1)(lh.superiorfrontal_10)
3787	0.87681	(Left-Caudate)(lh.insula_6)(lh.rostralmiddlefrontal_1)(lh.rostralmiddlefrontal_4)
3788	0.87681	(Left-Caudate)(lh.lateralorbitofrontal_4)(lh.lateralorbitofrontal_6)
3789	0.87681	(Left-Caudate)(lh.lateralorbitofrontal_7)(lh.medialorbitofrontal_2)(lh.medialorbitofrontal_4)
3790	0.87681	(Left-Caudate)(lh.rostralanteriorcingulate_1)(lh.rostralanteriorcingulate_2)(lh.rostralmiddlefrontal_4)
3791	0.87681	(Left-Caudate)(lh.superiorfrontal_3)
3792	0.87681	(Left-Hippocampus)(lh.entorhinal_1)(lh.parahippocampal_1)
3793	0.87681	(Left-Pallidum)(Left-Putamen)(lh.precentral_11)(lh.precentral_4)
3794	0.87681	(Left-Putamen)(lh.caudalanteriorcingulate_2)(lh.superiorfrontal_4)
3795	0.87681	(Left-Putamen)(lh.lateralorbitofrontal_4)(lh.rostralanteriorcingulate_1)(lh.rostralmiddlefrontal_9)
3796	0.87681	(Left-Putamen)(lh.lateralorbitofrontal_6)(lh.lateralorbitofrontal_7)(lh.parstriangularis_3)(lh.rostralmiddlefrontal_12)(lh.rostralmiddlefrontal_9)
3797	0.87681	(Left-Putamen)(lh.parstriangularis_3)(lh.rostralanteriorcingulate_2)
3798	0.87681	(Left-Putamen)(lh.rostralmiddlefrontal_4)(lh.rostralmiddlefrontal_8)
3799	0.87681	(Left-Putamen)(lh.rostralmiddlefrontal_8)(lh.rostralmiddlefrontal_9)
3800	0.87681	(Left-Putamen)(lh.superiorfrontal_10)(lh.superiorfrontal_11)

3801	0.87681	(Left-Thalamus-Proper)(Right-Accumbens-area)
3802	0.87681	(lh.caudalmiddlefrontal_3)(lh.insula_6)(lh.parstriangularis_3)
3803	0.87681	(lh.cuneus_2)(lh.superiorparietal_12)
3804	0.87681	(lh.inferiorparietal_10)(lh.inferiorparietal_5)
3805	0.87681	(lh.inferiorparietal_5)(lh.supramarginal_10)
3806	0.87681	(lh.inferiortemporal_3)(lh.middletemporal_3)(lh.middletemporal_4)
3807	0.87681	(lh.lateraloccipital_11)(lh.lateraloccipital_6)(lh.lingual_1)(lh.pericalcarine_2)
3808	0.87681	(lh.lateraloccipital_11)(lh.lingual_1)(lh.pericalcarine_3)
3809	0.87681	(lh.lateraloccipital_2)(lh.lateraloccipital_3)(lh.pericalcarine_1)
3810	0.87681	(lh.lateraloccipital_3)(lh.lingual_1)(lh.pericalcarine_2)(lh.pericalcarine_3)
3811	0.87681	(lh.lateraloccipital_7)(lh.lingual_1)
3812	0.87681	(lh.lateralorbitofrontal_7)(lh.medialorbitofrontal_2)(lh.parstriangularis_3)
3813	0.87681	(lh.lateralorbitofrontal_7)(lh.parstriangularis_3)(lh.rostralanteriorcingulate_1)(lh.rostralmiddlefrontal_9)
3814	0.87681	(lh.medialorbitofrontal_2)(lh.rostralmiddlefrontal_9)(lh.superiorfrontal_3)(lh.superiorfrontal_4)
3815	0.87681	(lh.paracentral_5)(lh.precuneus_2)(lh.superiorparietal_2)
3816	0.87681	(lh.parstriangularis_3)(lh.rostralanteriorcingulate_1)(lh.rostralanteriorcingulate_2)
3817	0.87681	(lh.precentral_11)(lh.precentral_13)(lh.precentral_14)
3818	0.87681	(lh.precentral_11)(lh.precentral_6)(lh.precentral_7)
3819	0.87681	(lh.precuneus_10)(lh.precuneus_6)(lh.precuneus_9)(lh.superiorparietal_11)
3820	0.87681	(lh.precuneus_6)(lh.precuneus_7)
3821	0.87681	(lh.rostralanteriorcingulate_1)(lh.rostralmiddlefrontal_1)(lh.rostralmiddlefrontal_9)
3822	0.87681	(lh.rostralanteriorcingulate_1)(lh.rostralmiddlefrontal_1)
3823	0.87681	(lh.rostralanteriorcingulate_2)(lh.rostralmiddlefrontal_9)(lh.superiorfrontal_4)
3824	0.87681	(lh.superiorfrontal_11)(lh.superiorfrontal_4)(lh.superiorfrontal_8)(lh.superiorfrontal_9)
3825	0.87681	(lh.superiorfrontal_16)(lh.superiorfrontal_17)
3826	0.87681	(lh.superiorparietal_12)(lh.superiorparietal_13)
3827	0.87681	(lh.superiorparietal_2)(lh.superiorparietal_5)
3828	0.87681	(lh.supramarginal_2)(lh.supramarginal_6)
3829	0.87681	(rh.bankssts_2)(rh.inferiorparietal_10)(rh.inferiorparietal_4)
3830	0.87681	(rh.caudalanteriorcingulate_2)(rh.superiorfrontal_7)
3831	0.87681	(rh.caudalmiddlefrontal_4)(rh.rostralmiddlefrontal_3)(rh.rostralmiddlefrontal_5)
3832	0.87681	(rh.caudalmiddlefrontal_4)(rh.superiorfrontal_11)(rh.superiorfrontal_13)(rh.superiorfrontal_9)
3833	0.87681	(rh.fusiform_1)(rh.fusiform_2)(rh.lateraloccipital_10)(rh.lingual_5)
3834	0.87681	(rh.inferiorparietal_10)(rh.inferiorparietal_11)(rh.lateraloccipital_10)
3835	0.87681	(rh.inferiortemporal_4)(rh.middletemporal_6)(rh.middletemporal_7)
3836	0.87681	(rh.insula_1)(rh.superiorparietal_1)(rh.supramarginal_8)(rh.supramarginal_9)
3837	0.87681	(rh.lateraloccipital_10)(rh.lateraloccipital_8)(rh.lingual_7)
3838	0.87681	(rh.lateraloccipital_4)(rh.lateraloccipital_5)(rh.lateraloccipital_8)
3839	0.87681	(rh.lateralorbitofrontal_6)(rh.medialorbitofrontal_2)(rh.rostralmiddlefrontal_12)
3840	0.87681	(rh.lateralorbitofrontal_7)(rh.rostralmiddlefrontal_8)(rh.superiorfrontal_2)
3841	0.87681	(rh.lingual_7)(rh.superiorparietal_11)
3842	0.87681	(rh.postcentral_8)(rh.supramarginal_1)
3843	0.87681	(rh.precuneus_4)(rh.superiorparietal_8)
3844	0.87681	(rh.precuneus_5)(rh.superiorparietal_8)
3845	0.87681	(rh.rostralmiddlefrontal_10)(rh.rostralmiddlefrontal_11)(rh.superiorfrontal_2)
3846	0.87681	(rh.rostralmiddlefrontal_12)(rh.rostralmiddlefrontal_13)
3847	0.87681	(rh.superiorfrontal_2)(rh.superiorfrontal_4)(rh.superiorfrontal_7)
3848	0.87681	(rh.superiorfrontal_2)(rh.superiorfrontal_6)(rh.superiorfrontal_7)(rh.superiorfrontal_8)
3849	0.87681	(rh.superiorparietal_1)(rh.superiorparietal_7)(rh.superiorparietal_8)
3850	0.87681	(rh.supramarginal_6)(rh.supramarginal_7)(rh.supramarginal_9)

3851	0.87681	(Right-Amygdala)(Right-Hippocampus)(rh.inferiortemporal_1)(rh.insula_5)
3852	0.87681	(Right-Caudate)(rh.lateralorbitofrontal_4)(rh.lateralorbitofrontal_6)
3853	0.87681	(Right-Caudate)(Right-Hippocampus)(rh.insula_1)
3854	0.87681	(Right-Caudate)(Right-Hippocampus)(rh.insula_2)
3855	0.87681	(Right-Caudate)(Right-Hippocampus)(Right-Putamen)(rh.insula_1)
3856	0.87681	(Right-Caudate)(Right-Hippocampus)(Right-Putamen)(rh.insula_2)
3857	0.87681	(Right-Caudate)(Right-Hippocampus)(Right-Putamen)(Right-Thalamus-Proper)(rh.insula_2)
3858	0.87681	(Right-Caudate)(Right-Hippocampus)(Right-Thalamus-Proper)(rh.insula_2)
3859	0.87681	(Right-Caudate)(Right-Putamen)(rh.caudalmiddlefrontal_4)(rh.precentral_13)
3860	0.87681	(Right-Caudate)(Right-Putamen)(rh.caudalmiddlefrontal_4)(rh.superiorfrontal_13)
3861	0.87681	(Right-Caudate)(Right-Putamen)(rh.lateralorbitofrontal_4)(rh.lateralorbitofrontal_6)
3862	0.87681	(Right-Caudate)(Right-Putamen)(rh.lateralorbitofrontal_5)(rh.medialorbitofrontal_2)
3863	0.87681	(Right-Caudate)(Right-Putamen)(rh.parsopercularis_4)(rh.rostralmiddlefrontal_3)
3864	0.87681	(Right-Hippocampus)(rh.transversetemporal_1)
3865	0.87681	(Right-Putamen)(rh.insula_1)(rh.superiorparietal_1)(rh.supramarginal_1)(rh.supramarginal_9)
3866	0.87681	(Right-Putamen)(rh.lateralorbitofrontal_7)(rh.rostralmiddlefrontal_12)(rh.superiorfrontal_2)
3867	0.87681	(Right-Putamen)(rh.parstriangularis_1)(rh.rostralmiddlefrontal_5)
3868	0.87681	(Right-Putamen)(rh.precentral_10)(rh.precentral_6)
3869	0.87681	(Right-Putamen)(rh.precentral_7)(rh.supramarginal_1)
3870	0.87681	(Right-Putamen)(rh.rostralmiddlefrontal_12)(rh.rostralmiddlefrontal_3)
3871	0.8744	(Left-Caudate)(Left-Putamen)(lh.caudalanteriorcingulate_2)(lh.caudalmiddlefrontal_1)
3872	0.8744	(Left-Caudate)(Left-Putamen)(lh.caudalmiddlefrontal_1)(lh.caudalmiddlefrontal_5)
3873	0.8744	(Left-Caudate)(Left-Putamen)(lh.lateralorbitofrontal_6)(lh.lateralorbitofrontal_7)
3874	0.8744	(Left-Caudate)(Left-Putamen)(lh.lateralorbitofrontal_6)(lh.parstriangularis_3)
3875	0.8744	(Left-Caudate)(lh.caudalanteriorcingulate_1)(lh.rostralmiddlefrontal_4)
3876	0.8744	(Left-Caudate)(lh.caudalmiddlefrontal_4)(lh.superiorfrontal_12)
3877	0.8744	(Left-Caudate)(lh.insula_6)(lh.precentral_14)
3878	0.8744	(Left-Caudate)(lh.lateralorbitofrontal_2)(lh.lateralorbitofrontal_7)(lh.medialorbitofrontal_2)
3879	0.8744	(Left-Caudate)(lh.lateralorbitofrontal_7)(lh.rostralanteriorcingulate_1)(lh.rostralmiddlefrontal_12)
3880	0.8744	(Left-Caudate)(lh.parstriangularis_3)(lh.rostralanteriorcingulate_1)(lh.rostralmiddlefrontal_12)
3881	0.8744	(Left-Caudate)(lh.parstriangularis_3)(lh.rostralanteriorcingulate_2)
3882	0.8744	(Left-Caudate)(lh.rostralmiddlefrontal_3)
3883	0.8744	(Left-Caudate)(lh.rostralmiddlefrontal_8)(lh.rostralmiddlefrontal_9)
3884	0.8744	(Left-Hippocampus)(lh.bankssts_2)(lh.fusiform_7)
3885	0.8744	(Left-Hippocampus)(lh.fusiform_6)(lh.fusiform_7)
3886	0.8744	(Left-Putamen)(lh.insula_1)(lh.postcentral_8)(lh.precentral_11)
3887	0.8744	(Left-Putamen)(lh.insula_6)(lh.rostralmiddlefrontal_1)(lh.rostralmiddlefrontal_9)
3888	0.8744	(Left-Putamen)(lh.rostralmiddlefrontal_4)(lh.rostralmiddlefrontal_9)(lh.superiorfrontal_4)
3889	0.8744	(Left-Putamen)(lh.superiorfrontal_11)(lh.superiorfrontal_14)
3890	0.8744	(Left-Putamen)(lh.transversetemporal_2)
3891	0.8744	(Left-Thalamus-Proper)(lh.superiorparietal_3)
3892	0.8744	(Left-Thalamus-Proper)(Right-Accumbens-area)(Right-Thalamus-Proper)
3893	0.8744	(lh.bankssts_2)(lh.inferiortemporal_6)(lh.middletemporal_3)
3894	0.8744	(lh.bankssts_2)(lh.middletemporal_4)
3895	0.8744	(lh.caudalanteriorcingulate_2)(lh.superiorfrontal_10)(lh.superiorfrontal_4)(lh.superiorfrontal_8)
3896	0.8744	(lh.caudalmiddlefrontal_1)(lh.caudalmiddlefrontal_2)(lh.caudalmiddlefrontal_3)
3897	0.8744	(lh.caudalmiddlefrontal_1)(lh.caudalmiddlefrontal_3)(lh.rostralmiddlefrontal_4)
3898	0.8744	(lh.caudalmiddlefrontal_3)(lh.insula_6)(lh.rostralmiddlefrontal_4)
3899	0.8744	(lh.fusiform_1)(lh.lingual_6)(lh.lingual_8)
3900	0.8744	(lh.inferiorparietal_6)(lh.inferiorparietal_7)(lh.inferiorparietal_9)

3901	0.8744	(lh.insula_1)(lh.superiorparietal_3)(lh.superiorparietal_5)
3902	0.8744	(lh.insula_6)(lh.parsopercularis_4)
3903	0.8744	(lh.lateraloccipital_1)(lh.lateraloccipital_3)(lh.pericalcarine_1)
3904	0.8744	(lh.lateraloccipital_2)(lh.pericalcarine_3)(lh.superiorparietal_12)
3905	0.8744	(lh.medialorbitofrontal_2)(lh.parstriangularis_3)(lh.rostralmiddlefrontal_12)
3906	0.8744	(lh.paracentral_5)(lh.superiorfrontal_8)
3907	0.8744	(lh.parsorbitalis_1)(lh.parstriangularis_3)(lh.rostralmiddlefrontal_6)
3908	0.8744	(lh.pericalcarine_3)(lh.precuneus_9)(lh.superiorparietal_12)
3909	0.8744	(lh.precuneus_10)(lh.precuneus_7)
3910	0.8744	(lh.precuneus_2)(lh.precuneus_6)
3911	0.8744	(lh.rostralmiddlefrontal_8)(lh.superiorfrontal_10)
3912	0.8744	(lh.superiorfrontal_11)(lh.superiorfrontal_12)(lh.superiorfrontal_4)(lh.superiorfrontal_8)
3913	0.8744	(lh.superiortemporal_11)(lh.superiortemporal_9)
3914	0.8744	(rh.caudalanteriorcingulate_3)(rh.posteriorcingulate_3)
3915	0.8744	(rh.caudalmiddlefrontal_1)(rh.caudalmiddlefrontal_2)(rh.caudalmiddlefrontal_4)
3916	0.8744	(rh.caudalmiddlefrontal_4)(rh.rostralmiddlefrontal_1)(rh.rostralmiddlefrontal_5)
3917	0.8744	(rh.fusiform_1)(rh.lingual_2)(rh.pericalcarine_1)
3918	0.8744	(rh.fusiform_2)(rh.fusiform_4)
3919	0.8744	(rh.fusiform_6)(rh.inferiortemporal_7)
3920	0.8744	(rh.inferiorparietal_1)(rh.inferiorparietal_4)(rh.supramarginal_9)
3921	0.8744	(rh.inferiorparietal_10)(rh.lateraloccipital_4)
3922	0.8744	(rh.inferiorparietal_12)(rh.lingual_7)(rh.precuneus_2)
3923	0.8744	(rh.inferiortemporal_1)(rh.inferiortemporal_2)
3924	0.8744	(rh.inferiortemporal_3)(rh.inferiortemporal_4)(rh.middletemporal_4)
3925	0.8744	(rh.isthmuscingulate_1)(rh.posteriorcingulate_3)
3926	0.8744	(rh.lateralorbitofrontal_2)(rh.lateralorbitofrontal_6)(rh.lateralorbitofrontal_7)(rh.parstriangularis_1)(rh.rostralmiddlefrontal_12)
3927	0.8744	(rh.lateralorbitofrontal_7)(rh.rostralmiddlefrontal_11)(rh.rostralmiddlefrontal_12)
3928	0.8744	(rh.parsopercularis_2)(rh.parsopercularis_3)(rh.precentral_1)
3929	0.8744	(rh.parsorbitalis_1)(rh.parstriangularis_3)
3930	0.8744	(rh.precuneus_7)(rh.precuneus_8)
3931	0.8744	(rh.precuneus_7)(rh.superiorparietal_10)(rh.superiorparietal_8)
3932	0.8744	(Right-Caudate)(rh.caudalanteriorcingulate_3)(rh.posteriorcingulate_3)
3933	0.8744	(Right-Caudate)(rh.parsopercularis_4)(rh.parstriangularis_1)
3934	0.8744	(Right-Caudate)(rh.precentral_15)
3935	0.8744	(Right-Caudate)(Right-Putamen)(rh.caudalmiddlefrontal_4)(rh.superiorfrontal_7)
3936	0.8744	(Right-Caudate)(Right-Putamen)(rh.parstriangularis_1)(rh.rostralmiddlefrontal_8)
3937	0.8744	(Right-Caudate)(Right-Putamen)(rh.precentral_6)
3938	0.8744	(Right-Hippocampus)(rh.bankssts_3)
3939	0.8744	(Right-Hippocampus)(rh.fusiform_8)(rh.inferiortemporal_1)
3940	0.8744	(Right-Hippocampus)(rh.insula_1)(rh.supramarginal_9)
3941	0.8744	(Right-Hippocampus)(rh.middletemporal_9)
3942	0.8744	(Right-Putamen)(rh.lateralorbitofrontal_6)(rh.medialorbitofrontal_2)(rh.rostralmiddlefrontal_12)
3943	0.8744	(Right-Putamen)(rh.lateralorbitofrontal_6)(rh.rostralmiddlefrontal_8)
3944	0.8744	(Right-Putamen)(rh.lateralorbitofrontal_7)(rh.rostralmiddlefrontal_11)
3945	0.8744	(Right-Putamen)(rh.precentral_6)(rh.precentral_7)
3946	0.8744	(Right-Putamen)(rh.rostralmiddlefrontal_11)(rh.superiorfrontal_2)
3947	0.87198	(Left-Accumbens-area)(Left-Hippocampus)(Left-Pallidum)(Left-Putamen)(Left-Thalamus-Proper)
3948	0.87198	(Left-Accumbens-area)(Left-Hippocampus)(Left-Pallidum)(Left-Putamen)
3949	0.87198	(Left-Amygdala)(Left-Hippocampus)(Left-Putamen)(Left-Thalamus-Proper)(lh.insula_2)
3950	0.87198	(Left-Amygdala)(Left-Hippocampus)(Left-Thalamus-Proper)(lh.insula_2)

3951	0.87198	(Left-Amygdala)(Left-Putamen)(Left-Thalamus-Proper)(lh.insula_2)
3952	0.87198	(Left-Amygdala)(Left-Thalamus-Proper)(lh.insula_2)
3953	0.87198	(Left-Caudate)(Left-Pallidum)(Left-Putamen)(lh.postcentral_8)
3954	0.87198	(Left-Caudate)(Left-Pallidum)(lh.postcentral_8)
3955	0.87198	(Left-Caudate)(Left-Pallidum)(lh.posteriorcingulate_3)
3956	0.87198	(Left-Caudate)(Left-Pallidum)(lh.precentral_11)(lh.precentral_4)
3957	0.87198	(Left-Caudate)(Left-Putamen)(lh.lateralorbitofrontal_2)(lh.medialorbitofrontal_5)
3958	0.87198	(Left-Caudate)(Left-Putamen)(lh.lateralorbitofrontal_4)(lh.lateralorbitofrontal_6)
3959	0.87198	(Left-Caudate)(Left-Putamen)(lh.lateralorbitofrontal_7)(lh.rostralanteriorcingulate_1)(lh.rostralmiddlefrontal_9)
3960	0.87198	(Left-Caudate)(Left-Putamen)(lh.medialorbitofrontal_2)(lh.medialorbitofrontal_4)
3961	0.87198	(Left-Caudate)(Left-Putamen)(lh.parstriangularis_3)(lh.rostralmiddlefrontal_12)(lh.rostralmiddlefrontal_9)
3962	0.87198	(Left-Caudate)(Left-Putamen)(lh.rostralmiddlefrontal_8)
3963	0.87198	(Left-Caudate)(lh.caudalanteriorcingulate_2)(lh.rostralanteriorcingulate_2)(lh.superiorfrontal_4)
3964	0.87198	(Left-Caudate)(lh.caudalmiddlefrontal_1)(lh.caudalmiddlefrontal_3)(lh.caudalmiddlefrontal_5)
3965	0.87198	(Left-Caudate)(lh.caudalmiddlefrontal_4)(lh.precentral_11)(lh.precentral_9)
3966	0.87198	(Left-Caudate)(lh.lateralorbitofrontal_6)(lh.rostralmiddlefrontal_1)
3967	0.87198	(Left-Caudate)(lh.lateralorbitofrontal_6)(lh.rostralmiddlefrontal_12)
3968	0.87198	(Left-Caudate)(lh.paracentral_5)(lh.postcentral_3)
3969	0.87198	(Left-Caudate)(rh.caudalanteriorcingulate_3)
3970	0.87198	(Left-Caudate)(Right-Caudate)(rh.caudalanteriorcingulate_3)
3971	0.87198	(Left-Hippocampus)(lh.lingual_6)(lh.lingual_7)
3972	0.87198	(Left-Pallidum)(lh.posteriorcingulate_3)
3973	0.87198	(Left-Putamen)(Left-Thalamus-Proper)(lh.insula_1)(lh.insula_2)(lh.precentral_11)
3974	0.87198	(Left-Putamen)(lh.caudalanteriorcingulate_2)(lh.caudalmiddlefrontal_3)(lh.superiorfrontal_12)
3975	0.87198	(Left-Putamen)(lh.caudalmiddlefrontal_3)(lh.parsopercularis_3)
3976	0.87198	(Left-Putamen)(lh.insula_6)(lh.parstriangularis_3)(lh.rostralmiddlefrontal_9)
3977	0.87198	(Left-Putamen)(lh.lateralorbitofrontal_4)(lh.lateralorbitofrontal_7)(lh.parstriangularis_3)(lh.rostralmiddlefrontal_9)
3978	0.87198	(Left-Putamen)(lh.lateralorbitofrontal_7)(lh.medialorbitofrontal_2)(lh.rostralanteriorcingulate_1)(lh.rostralmiddlefrontal_9)
3979	0.87198	(Left-Putamen)(lh.medialorbitofrontal_2)(lh.medialorbitofrontal_4)
3980	0.87198	(Left-Putamen)(lh.medialorbitofrontal_2)(lh.parstriangularis_3)
3981	0.87198	(Left-Putamen)(lh.postcentral_8)(lh.precentral_4)(lh.precentral_7)
3982	0.87198	(Left-Putamen)(lh.rostralanteriorcingulate_1)(lh.rostralmiddlefrontal_12)(lh.superiorfrontal_3)
3983	0.87198	(Left-Putamen)(lh.rostralanteriorcingulate_2)(lh.rostralmiddlefrontal_4)(lh.superiorfrontal_4)
3984	0.87198	(Left-Thalamus-Proper)(lh.insula_1)(lh.insula_2)(lh.precentral_11)
3985	0.87198	(lh.caudalmiddlefrontal_1)(lh.caudalmiddlefrontal_3)(lh.caudalmiddlefrontal_4)(lh.caudalmiddlefrontal_5)
3986	0.87198	(lh.caudalmiddlefrontal_3)(lh.rostralmiddlefrontal_9)
3987	0.87198	(lh.cuneus_1)(lh.pericalcarine_1)(lh.pericalcarine_3)
3988	0.87198	(lh.cuneus_2)(lh.pericalcarine_2)
3989	0.87198	(lh.fusiform_1)(lh.fusiform_3)(lh.lateraloccipital_9)
3990	0.87198	(lh.fusiform_2)(lh.lingual_4)(lh.lingual_8)(lh.pericalcarine_3)
3991	0.87198	(lh.inferiorparietal_1)(lh.precuneus_9)
3992	0.87198	(lh.inferiorparietal_8)(lh.inferiorparietal_9)(lh.superiorparietal_11)
3993	0.87198	(lh.insula_6)(lh.parstriangularis_3)(lh.rostralmiddlefrontal_1)(lh.rostralmiddlefrontal_4)
3994	0.87198	(lh.lateraloccipital_11)(lh.pericalcarine_2)(lh.pericalcarine_3)
3995	0.87198	(lh.lateralorbitofrontal_7)(lh.medialorbitofrontal_1)(lh.rostralmiddlefrontal_12)
3996	0.87198	(lh.paracentral_1)(lh.paracentral_4)(lh.precentral_2)(lh.precentral_3)
3997	0.87198	(lh.parstriangularis_3)(lh.rostralmiddlefrontal_8)
3998	0.87198	(lh.pericalcarine_3)(lh.precuneus_11)(lh.precuneus_9)
3999	0.87198	(lh.posteriorcingulate_1)(lh.superiorfrontal_12)
4000	0.87198	(lh.precentral_11)(lh.precentral_12)

4001	0.87198	(lh.rostralanteriorcingulate_1)(lh.rostralmiddlefrontal_8)
4002	0.87198	(lh.superiorfrontal_10)(lh.superiorfrontal_11)(lh.superiorfrontal_6)(lh.superiorfrontal_7)
4003	0.87198	(lh.superiorfrontal_11)(lh.superiorfrontal_13)(lh.superiorfrontal_14)(lh.superiorfrontal_8)
4004	0.87198	(lh.supramarginal_10)(lh.supramarginal_6)
4005	0.87198	(rh.caudalmiddlefrontal_4)(rh.insula_7)(rh.rostralmiddlefrontal_3)
4006	0.87198	(rh.cuneus_3)(rh.lateraloccipital_1)(rh.pericalcarine_2)
4007	0.87198	(rh.fusiform_1)(rh.lateraloccipital_4)(rh.lateraloccipital_8)(rh.lingual_2)
4008	0.87198	(rh.fusiform_7)(rh.inferiortemporal_5)
4009	0.87198	(rh.inferiorparietal_4)(rh.superiorparietal_1)
4010	0.87198	(rh.inferiorparietal_4)(rh.superiortemporal_1)
4011	0.87198	(rh.inferiortemporal_7)(rh.lateraloccipital_10)(rh.middletemporal_1)
4012	0.87198	(rh.lateraloccipital_3)(rh.pericalcarine_1)(rh.superiorparietal_11)
4013	0.87198	(rh.lateralorbitofrontal_6)(rh.parstriangularis_1)(rh.rostralmiddlefrontal_8)
4014	0.87198	(rh.lateralorbitofrontal_6)(rh.rostralmiddlefrontal_7)
4015	0.87198	(rh.lateralorbitofrontal_7)(rh.parstriangularis_1)(rh.rostralmiddlefrontal_8)
4016	0.87198	(rh.medialorbitofrontal_2)(rh.rostralmiddlefrontal_11)(rh.superiorfrontal_1)
4017	0.87198	(rh.pericalcarine_3)(rh.superiorparietal_11)
4018	0.87198	(rh.precentral_11)(rh.superiorparietal_1)
4019	0.87198	(rh.rostralmiddlefrontal_10)(rh.rostralmiddlefrontal_9)
4020	0.87198	(rh.superiorfrontal_11)(rh.superiorfrontal_13)(rh.superiorfrontal_14)
4021	0.87198	(rh.superiorfrontal_2)(rh.superiorfrontal_5)(rh.superiorfrontal_6)(rh.superiorfrontal_7)(rh.superiorfrontal_8)
4022	0.87198	(rh.superiorfrontal_2)(rh.superiorfrontal_5)(rh.superiorfrontal_7)(rh.superiorfrontal_9)
4023	0.87198	(rh.superiortemporal_1)(rh.superiortemporal_9)
4024	0.87198	(Right-Caudate)(rh.insula_1)(rh.insula_2)
4025	0.87198	(Right-Caudate)(Right-Hippocampus)(Right-Putamen)(Right-Thalamus-Proper)(rh.insula_1)
4026	0.87198	(Right-Caudate)(Right-Hippocampus)(Right-Thalamus-Proper)(rh.insula_1)
4027	0.87198	(Right-Caudate)(Right-Putamen)(rh.caudalanteriorcingulate_3)
4028	0.87198	(Right-Caudate)(Right-Putamen)(rh.insula_1)(rh.insula_2)
4029	0.87198	(Right-Caudate)(Right-Putamen)(rh.rostralmiddlefrontal_8)(rh.superiorfrontal_2)
4030	0.87198	(Right-Caudate)(Right-Putamen)(Right-Thalamus-Proper)(rh.precentral_11)(rh.precentral_13)
4031	0.87198	(Right-Hippocampus)(rh.fusiform_8)(rh.inferiortemporal_2)
4032	0.87198	(Right-Hippocampus)(Right-Putamen)(rh.insula_1)(rh.supramarginal_9)
4033	0.87198	(Right-Putamen)(rh.caudalanteriorcingulate_3)
4034	0.87198	(Right-Putamen)(rh.caudalmiddlefrontal_2)(rh.caudalmiddlefrontal_4)
4035	0.87198	(Right-Putamen)(rh.insula_1)(rh.postcentral_1)(rh.precentral_7)
4036	0.87198	(Right-Putamen)(rh.lateralorbitofrontal_7)(rh.medialorbitofrontal_2)(rh.rostralmiddlefrontal_11)
4037	0.87198	(Right-Putamen)(rh.lateralorbitofrontal_7)(rh.medialorbitofrontal_2)(rh.superiorfrontal_2)
4038	0.87198	(Right-Putamen)(rh.parstriangularis_1)(rh.rostralmiddlefrontal_5)(rh.rostralmiddlefrontal_8)
4039	0.87198	(Right-Putamen)(rh.rostralmiddlefrontal_5)(rh.superiorfrontal_2)
4040	0.86957	(Left-Caudate)(Left-Pallidum)(Left-Putamen)(lh.precentral_11)(lh.precentral_4)
4041	0.86957	(Left-Caudate)(Left-Putamen)(lh.caudalanteriorcingulate_2)(lh.caudalmiddlefrontal_3)(lh.superiorfrontal_12)
4042	0.86957	(Left-Caudate)(Left-Putamen)(lh.caudalmiddlefrontal_4)(lh.precentral_11)(lh.precentral_9)
4043	0.86957	(Left-Caudate)(Left-Putamen)(lh.insula_6)(lh.precentral_14)
4044	0.86957	(Left-Caudate)(Left-Putamen)(lh.lateralorbitofrontal_7)(lh.parstriangularis_3)(lh.rostralmiddlefrontal_9)
4045	0.86957	(Left-Caudate)(lh.caudalmiddlefrontal_5)(lh.superiorfrontal_12)
4046	0.86957	(Left-Caudate)(lh.insula_1)(lh.precentral_11)(lh.precentral_7)
4047	0.86957	(Left-Caudate)(lh.insula_6)(lh.rostralmiddlefrontal_1)(lh.rostralmiddlefrontal_9)
4048	0.86957	(Left-Caudate)(lh.lateralorbitofrontal_7)(lh.medialorbitofrontal_2)(lh.rostralmiddlefrontal_12)(lh.rostralmiddlefrontal_9)
4049	0.86957	(Left-Caudate)(lh.lateralorbitofrontal_7)(lh.parstriangularis_3)(lh.rostralanteriorcingulate_1)
4050	0.86957	(Left-Caudate)(lh.medialorbitofrontal_2)(lh.superiorfrontal_3)

4051	0.86957	(Left-Caudate)(lh.posteriorcingulate_1)(lh.superiorfrontal_12)
4052	0.86957	(Left-Caudate)(lh.rostralanteriorcingulate_1)(lh.rostralmiddlefrontal_1)
4053	0.86957	(Left-Caudate)(lh.rostralanteriorcingulate_1)(lh.rostralmiddlefrontal_4)(lh.superiorfrontal_4)
4054	0.86957	(Left-Hippocampus)(lh.fusiform_7)(lh.inferiortemporal_1)
4055	0.86957	(Left-Putamen)(lh.caudalmiddlefrontal_5)(lh.precentral_9)
4056	0.86957	(Left-Putamen)(lh.insula_2)(lh.insula_3)
4057	0.86957	(Left-Putamen)(lh.insula_6)(lh.parsopercularis_1)(lh.parsopercularis_3)(lh.rostralmiddlefrontal_1)
4058	0.86957	(Left-Putamen)(lh.insula_6)(lh.parsopercularis_1)(lh.parstriangularis_3)(lh.rostralmiddlefrontal_1)
4059	0.86957	(Left-Putamen)(lh.insula_6)(lh.parstriangularis_3)(lh.rostralmiddlefrontal_4)
4060	0.86957	(Left-Putamen)(lh.lateralorbitofrontal_4)(lh.lateralorbitofrontal_6)(lh.lateralorbitofrontal_7)(lh.parstriangularis_3)(lh.rostralmiddlefrontal_12)
4061	0.86957	(Left-Putamen)(lh.lateralorbitofrontal_7)(lh.medialorbitofrontal_5)
4062	0.86957	(Left-Putamen)(lh.medialorbitofrontal_2)(lh.rostralanteriorcingulate_1)(lh.superiorfrontal_3)
4063	0.86957	(Left-Putamen)(lh.medialorbitofrontal_2)(lh.rostralmiddlefrontal_12)(lh.rostralmiddlefrontal_9)(lh.superiorfrontal_3)
4064	0.86957	(Left-Thalamus-Proper)(lh.postcentral_8)(lh.precentral_7)
4065	0.86957	(lh.caudalanteriorcingulate_2)(lh.caudalmiddlefrontal_3)(lh.superiorfrontal_11)(lh.superiorfrontal_12)
4066	0.86957	(lh.caudalanteriorcingulate_2)(lh.superiorfrontal_10)(lh.superiorfrontal_11)(lh.superiorfrontal_4)
4067	0.86957	(lh.caudalmiddlefrontal_1)(lh.rostralmiddlefrontal_3)(lh.rostralmiddlefrontal_4)
4068	0.86957	(lh.caudalmiddlefrontal_6)(lh.precentral_13)
4069	0.86957	(lh.fusiform_1)(lh.fusiform_2)(lh.pericalcarine_3)
4070	0.86957	(lh.fusiform_5)(lh.lingual_6)
4071	0.86957	(lh.inferiorparietal_3)(lh.lateraloccipital_7)
4072	0.86957	(lh.inferiortemporal_3)(lh.inferiortemporal_5)
4073	0.86957	(lh.insula_1)(lh.superiorparietal_5)(lh.supramarginal_1)
4074	0.86957	(lh.insula_2)(lh.insula_3)
4075	0.86957	(lh.lateraloccipital_11)(lh.lateraloccipital_3)(lh.lateraloccipital_6)
4076	0.86957	(lh.lateraloccipital_6)(lh.lingual_1)(lh.lingual_4)(lh.pericalcarine_3)
4077	0.86957	(lh.lateraloccipital_7)(lh.lingual_6)
4078	0.86957	(lh.lateralorbitofrontal_4)(lh.lateralorbitofrontal_6)(lh.lateralorbitofrontal_7)(lh.rostralmiddlefrontal_12)(lh.rostralmiddlefrontal_9)
4079	0.86957	(lh.lateralorbitofrontal_7)(lh.parstriangularis_3)(lh.rostralmiddlefrontal_6)
4080	0.86957	(lh.lateralorbitofrontal_7)(lh.superiorfrontal_3)
4081	0.86957	(lh.lingual_6)(lh.lingual_7)(lh.lingual_8)
4082	0.86957	(lh.medialorbitofrontal_2)(lh.rostralmiddlefrontal_12)(lh.rostralmiddlefrontal_9)(lh.superiorfrontal_1)
4083	0.86957	(lh.paracentral_1)(lh.paracentral_4)(lh.paracentral_5)
4084	0.86957	(lh.paracentral_4)(lh.precentral_4)
4085	0.86957	(lh.parstriangularis_3)(lh.rostralanteriorcingulate_1)(lh.rostralmiddlefrontal_4)
4086	0.86957	(lh.postcentral_8)(lh.precentral_11)(lh.precentral_4)
4087	0.86957	(lh.posteriorcingulate_3)(lh.superiorfrontal_8)
4088	0.86957	(lh.rostralanteriorcingulate_2)(lh.rostralmiddlefrontal_1)
4089	0.86957	(lh.rostralmiddlefrontal_1)(lh.superiorfrontal_4)
4090	0.86957	(lh.rostralmiddlefrontal_8)(lh.superiorfrontal_10)(lh.superiorfrontal_4)
4091	0.86957	(lh.rostralmiddlefrontal_8)(lh.superiorfrontal_5)
4092	0.86957	(lh.superiorfrontal_10)(lh.superiorfrontal_11)(lh.superiorfrontal_4)(lh.superiorfrontal_6)(lh.superiorfrontal_7)
4093	0.86957	(lh.superiorfrontal_10)(lh.superiorfrontal_11)(lh.superiorfrontal_7)(lh.superiorfrontal_8)(lh.superiorfrontal_9)
4094	0.86957	(lh.superiorfrontal_6)(lh.superiorfrontal_9)
4095	0.86957	(lh.superiorparietal_6)(lh.superiorparietal_7)
4096	0.86957	(rh.caudalmiddlefrontal_2)(rh.caudalmiddlefrontal_4)(rh.caudalmiddlefrontal_5)
4097	0.86957	(rh.fusiform_1)(rh.lateraloccipital_5)
4098	0.86957	(rh.fusiform_3)(rh.fusiform_5)(rh.lateraloccipital_10)
4099	0.86957	(rh.fusiform_3)(rh.middletemporal_1)
4100	0.86957	(rh.inferiorparietal_11)(rh.lateraloccipital_10)(rh.lateraloccipital_8)

4101	0.86957	(rh.inferiortemporal_4)(rh.inferiortemporal_5)(rh.middletemporal_4)
4102	0.86957	(rh.insula_2)(rh.insula_4)(rh.insula_5)
4103	0.86957	(rh.insula_3)(rh.precentral_5)(rh.precentral_6)
4104	0.86957	(rh.insula_7)(rh.parstriangularis_1)(rh.rostralmiddlefrontal_3)
4105	0.86957	(rh.insula_7)(rh.rostralmiddlefrontal_8)
4106	0.86957	(rh.lateraloccipital_1)(rh.pericalcarine_3)
4107	0.86957	(rh.lateraloccipital_3)(rh.lateraloccipital_4)(rh.pericalcarine_2)
4108	0.86957	(rh.lateraloccipital_3)(rh.precuneus_1)
4109	0.86957	(rh.lateraloccipital_5)(rh.pericalcarine_3)
4110	0.86957	(rh.lateraloccipital_8)(rh.lingual_2)(rh.pericalcarine_1)
4111	0.86957	(rh.lateralorbitofrontal_2)(rh.lateralorbitofrontal_4)(rh.lateralorbitofrontal_7)
4112	0.86957	(rh.lateralorbitofrontal_6)(rh.lateralorbitofrontal_7)(rh.medialorbitofrontal_2)(rh.rostralmiddlefrontal_13)
4113	0.86957	(rh.lateralorbitofrontal_6)(rh.parstriangularis_3)(rh.parstriangularis_4)
4114	0.86957	(rh.lateralorbitofrontal_6)(rh.rostralmiddlefrontal_12)(rh.rostralmiddlefrontal_8)
4115	0.86957	(rh.medialorbitofrontal_2)(rh.rostralmiddlefrontal_11)(rh.rostralmiddlefrontal_12)
4116	0.86957	(rh.parstriangularis_1)(rh.rostralmiddlefrontal_3)(rh.rostralmiddlefrontal_5)
4117	0.86957	(rh.parstriangularis_3)(rh.rostralmiddlefrontal_12)
4118	0.86957	(rh.postcentral_5)(rh.precentral_5)
4119	0.86957	(rh.posteriorcingulate_4)(rh.superiorparietal_1)
4120	0.86957	(rh.precuneus_4)(rh.precuneus_7)(rh.superiorparietal_1)(rh.superiorparietal_7)
4121	0.86957	(rh.precuneus_4)(rh.superiorparietal_9)
4122	0.86957	(rh.rostralmiddlefrontal_1)(rh.rostralmiddlefrontal_5)(rh.rostralmiddlefrontal_8)
4123	0.86957	(rh.superiorfrontal_13)(rh.superiorfrontal_5)(rh.superiorfrontal_9)
4124	0.86957	(rh.supramarginal_4)(rh.supramarginal_8)
4125	0.86957	(Right-Accumbens-area)(Right-Amygdala)(Right-Hippocampus)(Right-Pallidum)(Right-Thalamus-Proper)
4126	0.86957	(Right-Accumbens-area)(Right-Amygdala)(Right-Hippocampus)(Right-Pallidum)
4127	0.86957	(Right-Accumbens-area)(Right-Amygdala)(Right-Hippocampus)(Right-Thalamus-Proper)
4128	0.86957	(Right-Accumbens-area)(Right-Amygdala)(Right-Hippocampus)
4129	0.86957	(Right-Amygdala)(rh.insula_4)(rh.insula_6)
4130	0.86957	(Right-Amygdala)(Right-Putamen)(rh.insula_4)(rh.insula_6)
4131	0.86957	(Right-Caudate)(rh.lateralorbitofrontal_2)(rh.lateralorbitofrontal_6)
4132	0.86957	(Right-Caudate)(rh.lateralorbitofrontal_7)(rh.medialorbitofrontal_3)
4133	0.86957	(Right-Caudate)(rh.rostralmiddlefrontal_5)(rh.rostralmiddlefrontal_8)
4134	0.86957	(Right-Caudate)(rh.supramarginal_8)(rh.supramarginal_9)
4135	0.86957	(Right-Caudate)(Right-Putamen)(rh.lateralorbitofrontal_2)(rh.lateralorbitofrontal_6)
4136	0.86957	(Right-Caudate)(Right-Putamen)(rh.medialorbitofrontal_2)(rh.superiorfrontal_2)
4137	0.86957	(Right-Hippocampus)(rh.bankssts_2)(rh.superiortemporal_1)(rh.superiortemporal_3)
4138	0.86957	(Right-Hippocampus)(rh.precuneus_4)(rh.precuneus_7)
4139	0.86957	(Right-Pallidum)(rh.precentral_5)
4140	0.86957	(Right-Pallidum)(rh.precentral_7)
4141	0.86957	(Right-Pallidum)(Right-Putamen)(rh.precentral_5)
4142	0.86957	(Right-Pallidum)(Right-Putamen)(rh.precentral_7)
4143	0.86957	(Right-Putamen)(rh.insula_2)(rh.insula_4)(rh.insula_5)
4144	0.86957	(Right-Putamen)(rh.insula_7)(rh.parsopercularis_4)(rh.rostralmiddlefrontal_3)
4145	0.86957	(Right-Putamen)(rh.insula_7)(rh.rostralmiddlefrontal_8)
4146	0.86957	(Right-Putamen)(rh.lateralorbitofrontal_2)(rh.lateralorbitofrontal_7)(rh.parstriangularis_1)(rh.rostralmiddlefrontal_12)
4147	0.86957	(Right-Putamen)(rh.precentral_12)
4148	0.86957	(Right-Putamen)(rh.rostralmiddlefrontal_1)(rh.rostralmiddlefrontal_5)
4149	0.86957	(Right-Putamen)(rh.rostralmiddlefrontal_10)(rh.rostralmiddlefrontal_8)
4150	0.86957	(Right-Putamen)(rh.rostralmiddlefrontal_12)(rh.rostralmiddlefrontal_5)

4151	0.86957	(Right-Thalamus-Proper)(rh.insula_1)(rh.precentral_11)
4152	0.86715	(Left-Caudate)(Left-Putamen)(lh.caudalanteriorcingulate_2)(lh.superiorfrontal_4)
4153	0.86715	(Left-Caudate)(Left-Putamen)(lh.insula_1)(lh.precentral_11)(lh.precentral_7)
4154	0.86715	(Left-Caudate)(Left-Putamen)(lh.lateralorbitofrontal_7)(lh.rostralanteriorcingulate_1)(lh.rostralmiddlefrontal_12)
4155	0.86715	(Left-Caudate)(Left-Putamen)(lh.medialorbitofrontal_4)(lh.rostralanteriorcingulate_1)
4156	0.86715	(Left-Caudate)(Left-Putamen)(lh.rostralanteriorcingulate_1)(lh.rostralmiddlefrontal_9)(lh.superiorfrontal_4)
4157	0.86715	(Left-Caudate)(Left-Putamen)(lh.superiorfrontal_11)(lh.superiorfrontal_14)
4158	0.86715	(Left-Caudate)(Left-Thalamus-Proper)(lh.isthmuscingulate_1)
4159	0.86715	(Left-Caudate)(lh.caudalanteriorcingulate_2)(lh.rostralanteriorcingulate_2)(lh.superiorfrontal_8)
4160	0.86715	(Left-Caudate)(lh.caudalmiddlefrontal_3)(lh.parstriangularis_3)
4161	0.86715	(Left-Caudate)(lh.lateralorbitofrontal_4)(lh.parstriangularis_3)(lh.rostralmiddlefrontal_9)
4162	0.86715	(Left-Caudate)(lh.lateralorbitofrontal_7)(lh.medialorbitofrontal_5)
4163	0.86715	(Left-Caudate)(lh.paracentral_5)(lh.posteriorcingulate_3)
4164	0.86715	(Left-Caudate)(lh.parsopercularis_3)(lh.parstriangularis_3)(lh.rostralmiddlefrontal_1)
4165	0.86715	(Left-Caudate)(lh.rostralmiddlefrontal_4)(lh.superiorfrontal_10)(lh.superiorfrontal_11)
4166	0.86715	(Left-Hippocampus)(Left-Putamen)(Left-Thalamus-Proper)(lh.insula_1)(lh.insula_2)
4167	0.86715	(Left-Hippocampus)(Left-Thalamus-Proper)(lh.insula_1)(lh.insula_2)
4168	0.86715	(Left-Hippocampus)(lh.parahippocampal_1)(lh.parahippocampal_2)
4169	0.86715	(Left-Hippocampus)(lh.supramarginal_1)
4170	0.86715	(Left-Pallidum)(Left-Putamen)(Left-Thalamus-Proper)(lh.insula_1)(lh.precentral_11)
4171	0.86715	(Left-Pallidum)(Left-Thalamus-Proper)(lh.insula_1)(lh.precentral_11)
4172	0.86715	(Left-Putamen)(Left-Thalamus-Proper)(lh.insula_1)(lh.precentral_11)(lh.precentral_7)
4173	0.86715	(Left-Putamen)(lh.caudalanteriorcingulate_2)(lh.caudalmiddlefrontal_5)
4174	0.86715	(Left-Putamen)(lh.caudalmiddlefrontal_3)(lh.insula_6)(lh.rostralmiddlefrontal_4)
4175	0.86715	(Left-Putamen)(lh.caudalmiddlefrontal_3)(lh.rostralmiddlefrontal_9)
4176	0.86715	(Left-Putamen)(lh.caudalmiddlefrontal_4)(lh.caudalmiddlefrontal_6)(lh.precentral_9)
4177	0.86715	(Left-Putamen)(lh.insula_1)(lh.supramarginal_9)
4178	0.86715	(Left-Putamen)(lh.insula_6)(lh.parsopercularis_1)(lh.parsopercularis_3)(lh.parstriangularis_3)
4179	0.86715	(Left-Putamen)(lh.medialorbitofrontal_2)(lh.superiorfrontal_4)
4180	0.86715	(Left-Putamen)(lh.medialorbitofrontal_4)(lh.rostralanteriorcingulate_1)
4181	0.86715	(Left-Putamen)(lh.paracentral_5)
4182	0.86715	(Left-Putamen)(lh.rostralmiddlefrontal_1)(lh.rostralmiddlefrontal_12)
4183	0.86715	(Left-Thalamus-Proper)(lh.insula_1)(lh.precentral_11)(lh.precentral_7)
4184	0.86715	(Left-Thalamus-Proper)(lh.precentral_6)
4185	0.86715	(lh.bankssts_1)(lh.superiortemporal_6)
4186	0.86715	(lh.caudalmiddlefrontal_3)(lh.rostralmiddlefrontal_1)(lh.rostralmiddlefrontal_9)
4187	0.86715	(lh.cuneus_1)(lh.precuneus_9)(lh.superiorparietal_12)
4188	0.86715	(lh.fusiform_1)(lh.inferiortemporal_8)
4189	0.86715	(lh.fusiform_4)(lh.fusiform_5)(lh.lateraloccipital_9)
4190	0.86715	(lh.fusiform_7)(lh.inferiortemporal_7)
4191	0.86715	(lh.inferiorparietal_10)(lh.inferiorparietal_6)
4192	0.86715	(lh.inferiorparietal_3)(lh.inferiorparietal_4)
4193	0.86715	(lh.insula_1)(lh.postcentral_8)(lh.precentral_7)
4194	0.86715	(lh.insula_1)(lh.supramarginal_1)(lh.supramarginal_6)
4195	0.86715	(lh.insula_6)(lh.parsopercularis_1)(lh.parsopercularis_3)(lh.parstriangularis_3)(lh.rostralmiddlefrontal_1)
4196	0.86715	(lh.insula_6)(lh.parsopercularis_3)(lh.parsopercularis_4)
4197	0.86715	(lh.lateralorbitofrontal_4)(lh.lateralorbitofrontal_7)(lh.parstriangularis_3)(lh.rostralmiddlefrontal_12)(lh.rostralmiddlefrontal_9)
4198	0.86715	(lh.lateralorbitofrontal_7)(lh.medialorbitofrontal_2)(lh.rostralanteriorcingulate_1)(lh.rostralmiddlefrontal_12)(lh.rostralmiddlefrontal_9)
4199	0.86715	(lh.lateralorbitofrontal_7)(lh.rostralmiddlefrontal_12)(lh.rostralmiddlefrontal_6)
4200	0.86715	(lh.lingual_1)(lh.lingual_3)(lh.lingual_4)(lh.pericalcarine_3)

4201	0.86715	(lh.medialorbitofrontal_2)(lh.rostralanteriorcingulate_1)(lh.rostralmiddlefrontal_9)(lh.superiorfrontal_4)
4202	0.86715	(lh.paracentral_1)(lh.superiorfrontal_16)
4203	0.86715	(lh.paracentral_4)(lh.paracentral_5)(lh.precentral_4)
4204	0.86715	(lh.paracentral_5)(lh.precentral_6)
4205	0.86715	(lh.paracentral_5)(lh.superiorfrontal_14)(lh.superiorfrontal_8)
4206	0.86715	(lh.parstriangularis_2)(lh.rostralmiddlefrontal_6)
4207	0.86715	(lh.parstriangularis_3)(lh.rostralanteriorcingulate_1)(lh.rostralmiddlefrontal_1)(lh.rostralmiddlefrontal_9)
4208	0.86715	(lh.parstriangularis_3)(lh.rostralanteriorcingulate_1)(lh.rostralmiddlefrontal_1)
4209	0.86715	(lh.postcentral_12)(lh.precentral_14)(lh.precentral_16)
4210	0.86715	(lh.postcentral_14)(lh.precentral_11)
4211	0.86715	(lh.precentral_4)(lh.superiorfrontal_12)
4212	0.86715	(lh.precuneus_9)(lh.superiorparietal_11)(lh.superiorparietal_5)
4213	0.86715	(lh.rostralmiddlefrontal_1)(lh.rostralmiddlefrontal_2)(lh.rostralmiddlefrontal_4)
4214	0.86715	(lh.rostralmiddlefrontal_12)(lh.rostralmiddlefrontal_4)
4215	0.86715	(lh.superiorfrontal_14)(lh.superiorfrontal_16)(lh.superiorfrontal_17)
4216	0.86715	(lh.superiorfrontal_14)(lh.superiorfrontal_4)(lh.superiorfrontal_7)(lh.superiorfrontal_8)
4217	0.86715	(lh.superiorfrontal_14)(lh.superiorfrontal_4)(lh.superiorfrontal_7)
4218	0.86715	(lh.superiorfrontal_6)(lh.superiorfrontal_7)(lh.superiorfrontal_9)
4219	0.86715	(rh.bankssts_1)(rh.inferiortemporal_4)(rh.middletemporal_4)
4220	0.86715	(rh.caudalanteriorcingulate_2)(rh.rostralanteriorcingulate_2)
4221	0.86715	(rh.caudalmiddlefrontal_1)(rh.caudalmiddlefrontal_2)(rh.caudalmiddlefrontal_3)
4222	0.86715	(rh.cuneus_3)(rh.superiorparietal_11)
4223	0.86715	(rh.cuneus_4)(rh.lateraloccipital_1)(rh.pericalcarine_2)
4224	0.86715	(rh.fusiform_1)(rh.pericalcarine_3)
4225	0.86715	(rh.fusiform_3)(rh.fusiform_4)(rh.inferiortemporal_7)
4226	0.86715	(rh.inferiorparietal_1)(rh.superiorparietal_10)
4227	0.86715	(rh.inferiorparietal_11)(rh.inferiorparietal_12)(rh.lateraloccipital_4)
4228	0.86715	(rh.inferiorparietal_12)(rh.lateraloccipital_4)(rh.pericalcarine_1)
4229	0.86715	(rh.insula_1)(rh.insula_2)(rh.insula_3)(rh.supramarginal_9)
4230	0.86715	(rh.insula_3)(rh.postcentral_1)
4231	0.86715	(rh.lateraloccipital_5)(rh.lingual_2)
4232	0.86715	(rh.lateralorbitofrontal_6)(rh.lateralorbitofrontal_7)(rh.medialorbitofrontal_2)(rh.rostralmiddlefrontal_12)
4233	0.86715	(rh.lateralorbitofrontal_6)(rh.parsorbitalis_2)(rh.parstriangularis_1)
4234	0.86715	(rh.lateralorbitofrontal_6)(rh.rostralmiddlefrontal_3)
4235	0.86715	(rh.lateralorbitofrontal_7)(rh.parstriangularis_1)(rh.rostralmiddlefrontal_12)(rh.rostralmiddlefrontal_8)
4236	0.86715	(rh.lateralorbitofrontal_7)(rh.rostralmiddlefrontal_12)(rh.rostralmiddlefrontal_13)
4237	0.86715	(rh.lingual_6)(rh.lingual_7)
4238	0.86715	(rh.paracentral_4)(rh.paracentral_6)
4239	0.86715	(rh.parsopercularis_2)(rh.parsopercularis_4)(rh.rostralmiddlefrontal_3)
4240	0.86715	(rh.parsorbitalis_2)(rh.rostralmiddlefrontal_7)
4241	0.86715	(rh.parstriangularis_1)(rh.rostralmiddlefrontal_3)(rh.rostralmiddlefrontal_5)(rh.rostralmiddlefrontal_8)
4242	0.86715	(rh.parstriangularis_2)(rh.parstriangularis_3)(rh.parstriangularis_4)
4243	0.86715	(rh.precentral_13)(rh.superiorfrontal_13)
4244	0.86715	(rh.precuneus_1)(rh.precuneus_2)
4245	0.86715	(rh.precuneus_10)(rh.precuneus_7)(rh.superiorparietal_1)(rh.superiorparietal_7)
4246	0.86715	(rh.precuneus_10)(rh.precuneus_7)(rh.superiorparietal_8)
4247	0.86715	(rh.rostralmiddlefrontal_5)(rh.superiorfrontal_7)
4248	0.86715	(rh.rostralmiddlefrontal_8)(rh.superiorfrontal_2)(rh.superiorfrontal_7)
4249	0.86715	(rh.superiorfrontal_13)(rh.superiorfrontal_7)(rh.superiorfrontal_8)
4250	0.86715	(rh.superiorfrontal_2)(rh.superiorfrontal_4)(rh.superiorfrontal_5)(rh.superiorfrontal_7)

4251	0.86715	(rh.superiorfrontal_3)(rh.superiorfrontal_4)(rh.superiorfrontal_5)(rh.superiorfrontal_6)
4252	0.86715	(Right-Amygdala)(rh.fusiform_8)
4253	0.86715	(Right-Amygdala)(rh.insula_4)(rh.insula_5)
4254	0.86715	(Right-Amygdala)(Right-Putamen)(rh.insula_4)(rh.insula_5)
4255	0.86715	(Right-Caudate)(rh.caudalanteriorcingulate_2)(rh.rostralanteriorcingulate_2)
4256	0.86715	(Right-Caudate)(rh.lateralorbitofrontal_2)(rh.parstriangularis_1)
4257	0.86715	(Right-Caudate)(rh.lateralorbitofrontal_6)(rh.parstriangularis_1)
4258	0.86715	(Right-Caudate)(rh.precentral_10)
4259	0.86715	(Right-Caudate)(Right-Thalamus-Proper)(rh.posteriorcingulate_2)(rh.precentral_13)
4260	0.86715	(Right-Pallidum)(rh.insula_2)
4261	0.86715	(Right-Pallidum)(Right-Putamen)(rh.insula_2)
4262	0.86715	(Right-Pallidum)(Right-Putamen)(Right-Thalamus-Proper)(rh.insula_2)
4263	0.86715	(Right-Pallidum)(Right-Thalamus-Proper)(rh.insula_2)
4264	0.86715	(Right-Putamen)(rh.insula_1)(rh.insula_2)(rh.insula_3)(rh.supramarginal_9)
4265	0.86715	(Right-Putamen)(rh.insula_1)(rh.postcentral_1)(rh.supramarginal_9)
4266	0.86715	(Right-Putamen)(rh.lateralorbitofrontal_2)(rh.lateralorbitofrontal_4)(rh.lateralorbitofrontal_7)
4267	0.86715	(Right-Putamen)(rh.rostralmiddlefrontal_12)(rh.rostralmiddlefrontal_3)(rh.rostralmiddlefrontal_8)
4268	0.86715	(Right-Putamen)(rh.supramarginal_1)(rh.supramarginal_8)(rh.supramarginal_9)
4269	0.86715	(Right-Putamen)(Right-Thalamus-Proper)(rh.insula_1)(rh.precentral_11)
4270	0.86715	(Right-Thalamus-Proper)(rh.posteriorcingulate_2)(rh.precentral_13)
4271	0.86473	(Left-Caudate)(Left-Pallidum)(Left-Putamen)(lh.precentral_9)
4272	0.86473	(Left-Caudate)(Left-Pallidum)(lh.precentral_9)
4273	0.86473	(Left-Caudate)(Left-Putamen)(lh.superiorfrontal_10)(lh.superiorfrontal_11)
4274	0.86473	(Left-Caudate)(lh.caudalmiddlefrontal_3)(lh.rostralmiddlefrontal_9)
4275	0.86473	(Left-Caudate)(lh.lateralorbitofrontal_4)(lh.rostralmiddlefrontal_12)
4276	0.86473	(Left-Caudate)(lh.parstriangularis_3)(lh.rostralanteriorcingulate_1)(lh.rostralmiddlefrontal_12)(lh.rostralmiddlefrontal_9)
4277	0.86473	(Left-Caudate)(lh.precentral_6)(lh.precentral_7)
4278	0.86473	(Left-Caudate)(lh.rostralanteriorcingulate_2)(lh.superiorfrontal_11)(lh.superiorfrontal_4)
4279	0.86473	(Left-Caudate)(lh.superiorfrontal_11)(lh.superiorfrontal_14)(lh.superiorfrontal_4)
4280	0.86473	(Left-Hippocampus)(lh.fusiform_5)(lh.lateraloccipital_9)(lh.lingual_8)
4281	0.86473	(Left-Putamen)(Left-Thalamus-Proper)(lh.postcentral_8)(lh.precentral_7)
4282	0.86473	(Left-Putamen)(lh.caudalanteriorcingulate_2)(lh.superiorfrontal_12)(lh.superiorfrontal_14)
4283	0.86473	(Left-Putamen)(lh.caudalmiddlefrontal_3)(lh.insula_6)(lh.parstriangularis_3)
4284	0.86473	(Left-Putamen)(lh.caudalmiddlefrontal_4)(lh.superiorfrontal_12)
4285	0.86473	(Left-Putamen)(lh.rostralanteriorcingulate_1)(lh.rostralanteriorcingulate_2)(lh.rostralmiddlefrontal_4)
4286	0.86473	(Left-Putamen)(lh.rostralanteriorcingulate_1)(lh.rostralmiddlefrontal_1)
4287	0.86473	(Left-Putamen)(lh.rostralanteriorcingulate_1)(lh.rostralmiddlefrontal_12)(lh.rostralmiddlefrontal_9)(lh.superiorfrontal_3)
4288	0.86473	(Left-Putamen)(lh.rostralmiddlefrontal_1)(lh.rostralmiddlefrontal_12)(lh.rostralmiddlefrontal_9)
4289	0.86473	(lh.caudalanteriorcingulate_2)(lh.superiorfrontal_10)(lh.superiorfrontal_11)(lh.superiorfrontal_4)(lh.superiorfrontal_8)
4290	0.86473	(lh.caudalmiddlefrontal_1)(lh.caudalmiddlefrontal_3)(lh.rostralmiddlefrontal_1)
4291	0.86473	(lh.caudalmiddlefrontal_3)(lh.insula_6)(lh.parsopercularis_3)
4292	0.86473	(lh.caudalmiddlefrontal_5)(lh.caudalmiddlefrontal_6)(lh.precentral_9)
4293	0.86473	(lh.fusiform_2)(lh.fusiform_3)(lh.lateraloccipital_9)
4294	0.86473	(lh.fusiform_2)(lh.fusiform_5)
4295	0.86473	(lh.fusiform_2)(lh.lateraloccipital_11)(lh.pericalcarine_3)
4296	0.86473	(lh.fusiform_2)(lh.lingual_6)(lh.pericalcarine_3)
4297	0.86473	(lh.inferiorparietal_4)(lh.inferiorparietal_8)
4298	0.86473	(lh.inferiorparietal_9)(lh.supramarginal_6)
4299	0.86473	(lh.insula_6)(lh.lateralorbitofrontal_6)(lh.parstriangularis_3)
4300	0.86473	(lh.insula_6)(lh.parsopercularis_1)(lh.parsopercularis_4)

4301	0.86473	(lh.lateraloccipital_11)(lh.lateraloccipital_3)(lh.lingual_1)
4302	0.86473	(lh.lateraloccipital_2)(lh.pericalcarine_1)(lh.pericalcarine_3)
4303	0.86473	(lh.lateralorbitofrontal_4)(lh.parstriangularis_3)(lh.rostralanteriorcingulate_1)
4304	0.86473	(lh.lateralorbitofrontal_6)(lh.lateralorbitofrontal_7)(lh.parstriangularis_3)(lh.rostralmiddlefrontal_6)
4305	0.86473	(lh.lateralorbitofrontal_6)(lh.parstriangularis_2)(lh.rostralmiddlefrontal_9)
4306	0.86473	(lh.lateralorbitofrontal_7)(lh.medialorbitofrontal_2)(lh.superiorfrontal_3)
4307	0.86473	(lh.lateralorbitofrontal_7)(lh.rostralmiddlefrontal_6)(lh.rostralmiddlefrontal_9)
4308	0.86473	(lh.lingual_1)(lh.lingual_3)(lh.pericalcarine_2)
4309	0.86473	(lh.paracentral_4)(lh.superiorfrontal_16)(lh.superiorfrontal_18)
4310	0.86473	(lh.parahippocampal_2)(lh.parahippocampal_3)
4311	0.86473	(lh.parsopercularis_1)(lh.parstriangularis_2)(lh.parstriangularis_3)
4312	0.86473	(lh.parsopercularis_1)(lh.parstriangularis_2)
4313	0.86473	(lh.postcentral_8)(lh.superiorparietal_3)
4314	0.86473	(lh.posteriorcingulate_1)(lh.superiorfrontal_14)
4315	0.86473	(lh.precentral_10)(lh.precentral_11)
4316	0.86473	(lh.precentral_11)(lh.precentral_5)
4317	0.86473	(lh.precentral_13)(lh.precentral_9)
4318	0.86473	(lh.precuneus_6)(lh.precuneus_9)(lh.superiorparietal_5)
4319	0.86473	(lh.rostralanteriorcingulate_2)(lh.superiorfrontal_10)(lh.superiorfrontal_8)
4320	0.86473	(lh.rostralmiddlefrontal_12)(lh.rostralmiddlefrontal_4)(lh.rostralmiddlefrontal_9)
4321	0.86473	(lh.rostralmiddlefrontal_8)(lh.superiorfrontal_4)(lh.superiorfrontal_5)
4322	0.86473	(lh.superiorfrontal_11)(lh.superiorfrontal_14)(lh.superiorfrontal_7)(lh.superiorfrontal_8)(lh.superiorfrontal_9)
4323	0.86473	(lh.superiorfrontal_15)(lh.superiorfrontal_17)(lh.superiorfrontal_18)
4324	0.86473	(rh.bankssts_1)(rh.middletemporal_1)(rh.middletemporal_2)
4325	0.86473	(rh.caudalanteriorcingulate_1)(rh.caudalanteriorcingulate_3)
4326	0.86473	(rh.caudalmiddlefrontal_4)(rh.insula_3)(rh.insula_7)(rh.parsopercularis_4)
4327	0.86473	(rh.caudalmiddlefrontal_4)(rh.rostralmiddlefrontal_8)
4328	0.86473	(rh.caudalmiddlefrontal_4)(rh.superiorfrontal_11)(rh.superiorfrontal_7)
4329	0.86473	(rh.cuneus_4)(rh.lateraloccipital_3)
4330	0.86473	(rh.fusiform_1)(rh.fusiform_2)(rh.lateraloccipital_8)(rh.lingual_5)
4331	0.86473	(rh.fusiform_1)(rh.lateraloccipital_4)(rh.pericalcarine_1)
4332	0.86473	(rh.fusiform_1)(rh.lingual_6)
4333	0.86473	(rh.inferiorparietal_1)(rh.inferiorparietal_4)(rh.inferiorparietal_6)
4334	0.86473	(rh.inferiortemporal_3)(rh.inferiortemporal_4)(rh.middletemporal_6)
4335	0.86473	(rh.inferiortemporal_6)(rh.lateraloccipital_10)
4336	0.86473	(rh.inferiortemporal_6)(rh.middletemporal_1)(rh.middletemporal_2)
4337	0.86473	(rh.insula_7)(rh.lateralorbitofrontal_2)(rh.parstriangularis_1)
4338	0.86473	(rh.isthmuscingulate_1)(rh.superiorparietal_1)
4339	0.86473	(rh.lateralorbitofrontal_6)(rh.rostralmiddlefrontal_12)(rh.rostralmiddlefrontal_13)
4340	0.86473	(rh.lingual_2)(rh.lingual_4)(rh.pericalcarine_3)
4341	0.86473	(rh.middletemporal_8)(rh.superiortemporal_6)
4342	0.86473	(rh.parsorbitalis_2)(rh.parstriangularis_1)(rh.rostralmiddlefrontal_12)
4343	0.86473	(rh.parstriangularis_1)(rh.parstriangularis_4)(rh.rostralmiddlefrontal_12)(rh.rostralmiddlefrontal_3)
4344	0.86473	(rh.precentral_11)(rh.precentral_15)
4345	0.86473	(rh.superiorparietal_1)(rh.superiorparietal_11)
4346	0.86473	(rh.superiorparietal_6)(rh.superiorparietal_7)
4347	0.86473	(Right-Caudate)(rh.caudalanteriorcingulate_1)(rh.caudalanteriorcingulate_3)
4348	0.86473	(Right-Caudate)(rh.lateralorbitofrontal_6)(rh.medialorbitofrontal_2)
4349	0.86473	(Right-Caudate)(rh.medialorbitofrontal_2)(rh.rostralmiddlefrontal_12)
4350	0.86473	(Right-Caudate)(Right-Putamen)(rh.caudalmiddlefrontal_1)(rh.caudalmiddlefrontal_4)

4351	0.86473	(Right-Caudate)(Right-Putamen)(rh.parsopercularis_4)(rh.parstriangularis_1)
4352	0.86473	(Right-Caudate)(Right-Putamen)(rh.precentral_10)
4353	0.86473	(Right-Caudate)(Right-Putamen)(Right-Thalamus-Proper)(rh.insula_1)(rh.insula_2)
4354	0.86473	(Right-Caudate)(Right-Thalamus-Proper)(rh.insula_1)(rh.insula_2)
4355	0.86473	(Right-Hippocampus)(rh.fusiform_6)
4356	0.86473	(Right-Hippocampus)(rh.insula_1)(rh.insula_2)
4357	0.86473	(Right-Hippocampus)(Right-Putamen)(rh.insula_1)(rh.insula_2)
4358	0.86473	(Right-Putamen)(rh.caudalmiddlefrontal_4)(rh.insula_3)(rh.insula_7)(rh.parsopercularis_4)
4359	0.86473	(Right-Putamen)(rh.caudalmiddlefrontal_4)(rh.rostralmiddlefrontal_8)
4360	0.86473	(Right-Putamen)(rh.medialorbitofrontal_2)(rh.rostralmiddlefrontal_12)(rh.superiorfrontal_2)
4361	0.86473	(Right-Putamen)(rh.postcentral_10)
4362	0.86473	(Right-Putamen)(rh.rostralmiddlefrontal_12)(rh.rostralmiddlefrontal_5)(rh.rostralmiddlefrontal_8)
4363	0.86232	(Left-Accumbens-area)(lh.lateralorbitofrontal_2)
4364	0.86232	(Left-Caudate)(Left-Pallidum)(Left-Thalamus-Proper)(lh.posteriorcingulate_3)
4365	0.86232	(Left-Caudate)(Left-Putamen)(lh.lateralorbitofrontal_2)(lh.lateralorbitofrontal_7)(lh.medialorbitofrontal_2)
4366	0.86232	(Left-Caudate)(Left-Putamen)(lh.lateralorbitofrontal_6)(lh.rostralmiddlefrontal_12)
4367	0.86232	(Left-Caudate)(lh.caudalanteriorcingulate_2)(lh.caudalmiddlefrontal_3)(lh.superiorfrontal_11)(lh.superiorfrontal_12)
4368	0.86232	(Left-Caudate)(lh.lateralorbitofrontal_6)(lh.lateralorbitofrontal_7)(lh.parstriangularis_3)
4369	0.86232	(Left-Caudate)(lh.lateralorbitofrontal_7)(lh.medialorbitofrontal_2)(lh.rostralanteriorcingulate_1)(lh.rostralmiddlefrontal_12)
4370	0.86232	(Left-Caudate)(lh.rostralanteriorcingulate_1)(lh.rostralmiddlefrontal_1)(lh.rostralmiddlefrontal_9)
4371	0.86232	(Left-Caudate)(lh.rostralanteriorcingulate_2)(lh.rostralmiddlefrontal_4)(lh.rostralmiddlefrontal_9)
4372	0.86232	(Left-Caudate)(lh.superiorfrontal_12)(lh.superiorfrontal_4)
4373	0.86232	(Left-Caudate)(lh.superiorfrontal_3)(lh.superiorfrontal_4)
4374	0.86232	(Left-Hippocampus)(lh.bankssts_3)(lh.fusiform_7)
4375	0.86232	(Left-Pallidum)(Left-Thalamus-Proper)(lh.posteriorcingulate_3)
4376	0.86232	(Left-Putamen)(lh.caudalmiddlefrontal_1)(lh.caudalmiddlefrontal_4)(lh.caudalmiddlefrontal_5)
4377	0.86232	(Left-Putamen)(lh.caudalmiddlefrontal_3)(lh.parstriangularis_3)(lh.rostralmiddlefrontal_1)
4378	0.86232	(Left-Putamen)(lh.insula_6)(lh.parsopercularis_3)(lh.parstriangularis_3)(lh.rostralmiddlefrontal_1)
4379	0.86232	(Left-Putamen)(lh.lateralorbitofrontal_4)(lh.lateralorbitofrontal_6)(lh.lateralorbitofrontal_7)(lh.rostralmiddlefrontal_12)(lh.rostralmiddlefrontal_9)
4380	0.86232	(Left-Putamen)(lh.lateralorbitofrontal_7)(lh.parstriangularis_3)(lh.rostralanteriorcingulate_1)(lh.rostralmiddlefrontal_12)
4381	0.86232	(Left-Putamen)(lh.medialorbitofrontal_2)(lh.parstriangularis_3)(lh.rostralmiddlefrontal_9)
4382	0.86232	(Left-Putamen)(lh.postcentral_8)(lh.precentral_11)(lh.precentral_7)
4383	0.86232	(Left-Putamen)(lh.postcentral_8)(lh.supramarginal_9)
4384	0.86232	(Left-Putamen)(lh.rostralanteriorcingulate_1)(lh.rostralmiddlefrontal_1)(lh.rostralmiddlefrontal_9)
4385	0.86232	(Left-Putamen)(lh.rostralanteriorcingulate_2)(lh.superiorfrontal_11)
4386	0.86232	(Left-Putamen)(lh.rostralmiddlefrontal_4)(lh.superiorfrontal_10)
4387	0.86232	(Left-Putamen)(lh.superiorfrontal_10)(lh.superiorfrontal_4)
4388	0.86232	(Left-Thalamus-Proper)(lh.precentral_6)(lh.precentral_7)
4389	0.86232	(lh.bankssts_3)(lh.fusiform_4)
4390	0.86232	(lh.caudalanteriorcingulate_1)(lh.superiorfrontal_11)
4391	0.86232	(lh.caudalmiddlefrontal_2)(lh.caudalmiddlefrontal_3)(lh.caudalmiddlefrontal_4)
4392	0.86232	(lh.fusiform_1)(lh.fusiform_2)(lh.fusiform_5)
4393	0.86232	(lh.inferiorparietal_4)(lh.inferiorparietal_5)
4394	0.86232	(lh.lateraloccipital_10)(lh.lateraloccipital_7)
4395	0.86232	(lh.lateraloccipital_6)(lh.lingual_8)(lh.pericalcarine_3)
4396	0.86232	(lh.lateraloccipital_7)(lh.lateraloccipital_9)(lh.lingual_8)
4397	0.86232	(lh.lateraloccipital_9)(lh.lingual_6)
4398	0.86232	(lh.lateralorbitofrontal_1)(lh.lateralorbitofrontal_4)
4399	0.86232	(lh.lateralorbitofrontal_6)(lh.parsorbitalis_1)(lh.parstriangularis_3)(lh.rostralmiddlefrontal_6)
4400	0.86232	(lh.lateralorbitofrontal_6)(lh.parstriangularis_2)(lh.parstriangularis_3)(lh.rostralmiddlefrontal_9)

4401	0.86232	(lh.lateralorbitofrontal_7)(lh.medialorbitofrontal_2)(lh.parstriangularis_3)(lh.rostralmiddlefrontal_12)
4402	0.86232	(lh.lingual_1)(lh.lingual_8)(lh.pericalcarine_3)
4403	0.86232	(lh.medialorbitofrontal_1)(lh.medialorbitofrontal_2)(lh.superiorfrontal_1)
4404	0.86232	(lh.medialorbitofrontal_2)(lh.parstriangularis_3)(lh.rostralanteriorcingulate_1)
4405	0.86232	(lh.medialorbitofrontal_2)(lh.parstriangularis_3)(lh.rostralmiddlefrontal_12)(lh.rostralmiddlefrontal_9)
4406	0.86232	(lh.parstriangularis_3)(lh.rostralanteriorcingulate_1)(lh.rostralmiddlefrontal_4)(lh.rostralmiddlefrontal_9)
4407	0.86232	(lh.pericalcarine_2)(lh.pericalcarine_3)(lh.superiorparietal_12)
4408	0.86232	(lh.postcentral_4)(lh.superiorparietal_3)
4409	0.86232	(lh.postcentral_8)(lh.precentral_6)(lh.precentral_7)
4410	0.86232	(lh.postcentral_8)(lh.supramarginal_4)(lh.supramarginal_9)
4411	0.86232	(lh.precentral_4)(lh.superiorfrontal_15)
4412	0.86232	(lh.precuneus_2)(lh.superiorparietal_8)
4413	0.86232	(lh.precuneus_6)(lh.precuneus_8)(lh.superiorparietal_6)
4414	0.86232	(lh.precuneus_6)(lh.superiorparietal_11)(lh.superiorparietal_5)(lh.superiorparietal_6)
4415	0.86232	(lh.rostralanteriorcingulate_1)(lh.rostralmiddlefrontal_8)(lh.superiorfrontal_4)
4416	0.86232	(lh.rostralanteriorcingulate_2)(lh.superiorfrontal_11)(lh.superiorfrontal_8)
4417	0.86232	(lh.rostralmiddlefrontal_12)(lh.rostralmiddlefrontal_9)(lh.superiorfrontal_4)
4418	0.86232	(lh.rostralmiddlefrontal_2)(lh.rostralmiddlefrontal_3)(lh.rostralmiddlefrontal_4)
4419	0.86232	(lh.superiorfrontal_10)(lh.superiorfrontal_5)(lh.superiorfrontal_6)
4420	0.86232	(lh.superiorfrontal_11)(lh.superiorfrontal_4)(lh.superiorfrontal_7)(lh.superiorfrontal_8)(lh.superiorfrontal_9)
4421	0.86232	(rh.bankssts_1)(rh.inferiortemporal_4)(rh.inferiortemporal_5)
4422	0.86232	(rh.fusiform_1)(rh.lateraloccipital_8)(rh.lingual_4)
4423	0.86232	(rh.fusiform_2)(rh.inferiorparietal_10)(rh.lateraloccipital_8)
4424	0.86232	(rh.inferiorparietal_12)(rh.inferiorparietal_6)
4425	0.86232	(rh.inferiorparietal_12)(rh.lateraloccipital_8)(rh.pericalcarine_1)
4426	0.86232	(rh.inferiortemporal_2)(rh.inferiortemporal_4)(rh.middletemporal_7)
4427	0.86232	(rh.inferiortemporal_3)(rh.inferiortemporal_4)(rh.inferiortemporal_5)
4428	0.86232	(rh.insula_1)(rh.supramarginal_7)(rh.supramarginal_9)
4429	0.86232	(rh.insula_5)(rh.middletemporal_9)(rh.superiortemporal_10)
4430	0.86232	(rh.lateraloccipital_3)(rh.precuneus_1)(rh.superiorparietal_11)
4431	0.86232	(rh.lateraloccipital_4)(rh.lateraloccipital_7)(rh.lingual_2)(rh.pericalcarine_3)
4432	0.86232	(rh.lateralorbitofrontal_2)(rh.parstriangularis_1)(rh.rostralmiddlefrontal_12)(rh.rostralmiddlefrontal_8)
4433	0.86232	(rh.lateralorbitofrontal_6)(rh.parsorbitalis_2)(rh.rostralmiddlefrontal_13)
4434	0.86232	(rh.pericalcarine_1)(rh.precuneus_1)
4435	0.86232	(rh.precentral_13)(rh.superiorparietal_2)
4436	0.86232	(rh.rostralanteriorcingulate_2)(rh.rostralmiddlefrontal_8)
4437	0.86232	(rh.rostralmiddlefrontal_12)(rh.rostralmiddlefrontal_6)(rh.rostralmiddlefrontal_8)
4438	0.86232	(rh.rostralmiddlefrontal_2)(rh.rostralmiddlefrontal_3)(rh.rostralmiddlefrontal_4)
4439	0.86232	(rh.rostralmiddlefrontal_5)(rh.rostralmiddlefrontal_8)(rh.rostralmiddlefrontal_9)
4440	0.86232	(rh.superiorfrontal_11)(rh.superiorfrontal_12)(rh.superiorfrontal_15)
4441	0.86232	(rh.superiorfrontal_11)(rh.superiorfrontal_13)(rh.superiorfrontal_5)
4442	0.86232	(Right-Caudate)(rh.caudalmiddlefrontal_4)(rh.rostralmiddlefrontal_5)
4443	0.86232	(Right-Caudate)(rh.caudalmiddlefrontal_4)(rh.superiorfrontal_9)
4444	0.86232	(Right-Caudate)(rh.caudalmiddlefrontal_5)(rh.superiorfrontal_7)
4445	0.86232	(Right-Caudate)(rh.precentral_13)(rh.precentral_15)
4446	0.86232	(Right-Caudate)(Right-Putamen)(rh.lateralorbitofrontal_2)(rh.parstriangularis_1)
4447	0.86232	(Right-Caudate)(Right-Putamen)(rh.lateralorbitofrontal_6)(rh.medialorbitofrontal_2)
4448	0.86232	(Right-Caudate)(Right-Putamen)(rh.lateralorbitofrontal_6)(rh.parstriangularis_1)
4449	0.86232	(Right-Caudate)(Right-Putamen)(rh.medialorbitofrontal_2)(rh.rostralmiddlefrontal_12)
4450	0.86232	(Right-Caudate)(Right-Putamen)(Right-Thalamus-Proper)(rh.posteriorcingulate_4)

4451	0.86232	(Right-Hippocampus)(rh.entorhinal_1)(rh.parahippocampal_3)
4452	0.86232	(Right-Hippocampus)(rh.precuneus_3)(rh.precuneus_4)
4453	0.86232	(Right-Hippocampus)(rh.superiorparietal_1)
4454	0.86232	(Right-Hippocampus)(Right-Pallidum)(rh.insula_2)
4455	0.86232	(Right-Hippocampus)(Right-Pallidum)(Right-Putamen)(rh.insula_2)
4456	0.86232	(Right-Hippocampus)(Right-Pallidum)(Right-Putamen)(Right-Thalamus-Proper)(rh.insula_2)
4457	0.86232	(Right-Hippocampus)(Right-Pallidum)(Right-Thalamus-Proper)(rh.insula_2)
4458	0.86232	(Right-Putamen)(rh.insula_7)(rh.lateralorbitofrontal_2)(rh.parstriangularis_1)
4459	0.86232	(Right-Putamen)(rh.lateralorbitofrontal_2)(rh.lateralorbitofrontal_6)(rh.lateralorbitofrontal_7)(rh.parstriangularis_1)(rh.rostralmiddlefrontal_12)
4460	0.86232	(Right-Putamen)(rh.lateralorbitofrontal_6)(rh.lateralorbitofrontal_7)(rh.medialorbitofrontal_2)(rh.rostralmiddlefrontal_12)
4461	0.86232	(Right-Putamen)(rh.lateralorbitofrontal_6)(rh.parstriangularis_1)(rh.rostralmiddlefrontal_8)
4462	0.86232	(Right-Putamen)(rh.rostralmiddlefrontal_10)(rh.superiorfrontal_2)
4463	0.86232	(Right-Putamen)(rh.superiorparietal_1)(rh.supramarginal_8)
4464	0.86232	(Right-Putamen)(Right-Thalamus-Proper)(rh.posteriorcingulate_4)
4465	0.8599	(Left-Accumbens-area)(Left-Putamen)(lh.medialorbitofrontal_4)
4466	0.8599	(Left-Accumbens-area)(Right-Thalamus-Proper)
4467	0.8599	(Left-Caudate)(Left-Putamen)(lh.caudalmiddlefrontal_3)(lh.parstriangularis_3)
4468	0.8599	(Left-Caudate)(Left-Putamen)(lh.caudalmiddlefrontal_3)(lh.rostralmiddlefrontal_9)
4469	0.8599	(Left-Caudate)(Left-Putamen)(lh.insula_6)(lh.rostralmiddlefrontal_1)(lh.rostralmiddlefrontal_4)
4470	0.8599	(Left-Caudate)(Left-Putamen)(lh.lateralorbitofrontal_4)(lh.rostralmiddlefrontal_12)
4471	0.8599	(Left-Caudate)(Left-Putamen)(lh.lateralorbitofrontal_7)(lh.medialorbitofrontal_2)(lh.rostralmiddlefrontal_12)(lh.rostralmiddlefrontal_9)
4472	0.8599	(Left-Caudate)(Left-Putamen)(lh.medialorbitofrontal_2)(lh.superiorfrontal_4)
4473	0.8599	(Left-Caudate)(Left-Putamen)(lh.parstriangularis_3)(lh.rostralmiddlefrontal_1)(lh.rostralmiddlefrontal_4)(lh.rostralmiddlefrontal_9)
4474	0.8599	(Left-Caudate)(Left-Putamen)(lh.postcentral_3)
4475	0.8599	(Left-Caudate)(lh.caudalanteriorcingulate_1)(lh.superiorfrontal_11)
4476	0.8599	(Left-Caudate)(lh.lateralorbitofrontal_6)(lh.rostralmiddlefrontal_12)(lh.rostralmiddlefrontal_9)
4477	0.8599	(Left-Caudate)(lh.lateralorbitofrontal_7)(lh.medialorbitofrontal_2)(lh.rostralanteriorcingulate_1)(lh.rostralmiddlefrontal_9)
4478	0.8599	(Left-Caudate)(lh.medialorbitofrontal_2)(lh.parstriangularis_3)
4479	0.8599	(Left-Caudate)(lh.parsopercularis_1)
4480	0.8599	(Left-Caudate)(lh.rostralanteriorcingulate_1)(lh.superiorfrontal_3)
4481	0.8599	(Left-Caudate)(lh.rostralanteriorcingulate_2)(lh.superiorfrontal_10)(lh.superiorfrontal_4)
4482	0.8599	(Left-Caudate)(rh.rostralanteriorcingulate_1)
4483	0.8599	(Left-Hippocampus)(lh.lingual_7)(lh.lingual_8)
4484	0.8599	(Left-Putamen)(Left-Thalamus-Proper)(lh.postcentral_3)
4485	0.8599	(Left-Putamen)(Left-Thalamus-Proper)(lh.transversetemporal_2)
4486	0.8599	(Left-Putamen)(lh.caudalmiddlefrontal_1)(lh.caudalmiddlefrontal_3)(lh.caudalmiddlefrontal_5)
4487	0.8599	(Left-Putamen)(lh.lateralorbitofrontal_1)(lh.lateralorbitofrontal_4)
4488	0.8599	(Left-Putamen)(lh.lateralorbitofrontal_4)(lh.lateralorbitofrontal_6)(lh.lateralorbitofrontal_7)(lh.parstriangularis_3)(lh.rostralmiddlefrontal_9)
4489	0.8599	(Left-Putamen)(lh.posteriorcingulate_3)(lh.superiorfrontal_14)
4490	0.8599	(lh.caudalanteriorcingulate_1)(lh.superiorfrontal_8)
4491	0.8599	(lh.caudalanteriorcingulate_2)(lh.rostralanteriorcingulate_2)(lh.superiorfrontal_10)
4492	0.8599	(lh.caudalmiddlefrontal_3)(lh.rostralmiddlefrontal_4)(lh.superiorfrontal_11)
4493	0.8599	(lh.cuneus_1)(lh.lateraloccipital_2)(lh.pericalcarine_3)
4494	0.8599	(lh.fusiform_1)(lh.fusiform_5)(lh.inferiortemporal_8)
4495	0.8599	(lh.fusiform_1)(lh.lingual_8)(lh.pericalcarine_3)
4496	0.8599	(lh.fusiform_2)(lh.lateraloccipital_6)(lh.lingual_1)(lh.lingual_4)
4497	0.8599	(lh.fusiform_3)(lh.fusiform_5)(lh.lateraloccipital_9)
4498	0.8599	(lh.fusiform_5)(lh.inferiorparietal_4)(lh.lateraloccipital_9)
4499	0.8599	(lh.fusiform_5)(lh.lingual_6)(lh.lingual_8)
4500	0.8599	(lh.inferiorparietal_1)(lh.inferiorparietal_2)

4501	0.8599	(lh.inferiorparietal_1)(lh.lateraloccipital_2)(lh.pericalcarine_3)
4502	0.8599	(lh.lateraloccipital_10)(lh.lateraloccipital_9)
4503	0.8599	(lh.lateraloccipital_2)(lh.lateraloccipital_6)(lh.pericalcarine_2)(lh.pericalcarine_3)
4504	0.8599	(lh.lateraloccipital_6)(lh.lingual_3)
4505	0.8599	(lh.lateralorbitofrontal_6)(lh.lateralorbitofrontal_7)(lh.rostralmiddlefrontal_12)(lh.rostralmiddlefrontal_6)
4506	0.8599	(lh.lingual_3)(lh.pericalcarine_2)(lh.pericalcarine_3)
4507	0.8599	(lh.medialorbitofrontal_2)(lh.rostralmiddlefrontal_12)(lh.rostralmiddlefrontal_9)(lh.superiorfrontal_1)(lh.superiorfrontal_3)
4508	0.8599	(lh.medialorbitofrontal_5)(lh.rostralanteriorcingulate_1)
4509	0.8599	(lh.paracentral_4)(lh.superiorfrontal_15)(lh.superiorfrontal_18)
4510	0.8599	(lh.parsopercularis_2)(lh.parstriangularis_2)
4511	0.8599	(lh.postcentral_3)(lh.precentral_4)
4512	0.8599	(lh.postcentral_8)(lh.precentral_11)(lh.precentral_14)
4513	0.8599	(lh.precuneus_10)(lh.precuneus_11)(lh.superiorparietal_11)
4514	0.8599	(lh.rostralanteriorcingulate_1)(lh.rostralanteriorcingulate_2)(lh.rostralmiddlefrontal_4)(lh.superiorfrontal_4)
4515	0.8599	(lh.rostralanteriorcingulate_1)(lh.rostralanteriorcingulate_2)(lh.rostralmiddlefrontal_9)(lh.superiorfrontal_4)
4516	0.8599	(lh.rostralmiddlefrontal_1)(lh.rostralmiddlefrontal_8)
4517	0.8599	(lh.rostralmiddlefrontal_2)(lh.rostralmiddlefrontal_5)
4518	0.8599	(lh.rostralmiddlefrontal_5)(lh.rostralmiddlefrontal_9)
4519	0.8599	(lh.superiorfrontal_10)(lh.superiorfrontal_3)(lh.superiorfrontal_6)
4520	0.8599	(lh.superiorfrontal_10)(lh.superiorfrontal_4)(lh.superiorfrontal_5)(lh.superiorfrontal_6)
4521	0.8599	(lh.superiorfrontal_15)(lh.superiorfrontal_16)(lh.superiorfrontal_17)
4522	0.8599	(lh.superiorparietal_5)(lh.superiortemporal_2)
4523	0.8599	(rh.caudalmiddlefrontal_1)(rh.caudalmiddlefrontal_5)
4524	0.8599	(rh.cuneus_2)(rh.cuneus_3)
4525	0.8599	(rh.cuneus_2)(rh.superiorparietal_13)
4526	0.8599	(rh.fusiform_1)(rh.fusiform_2)(rh.inferiorparietal_10)
4527	0.8599	(rh.fusiform_8)(rh.inferiortemporal_1)(rh.insula_5)
4528	0.8599	(rh.inferiorparietal_10)(rh.inferiorparietal_12)(rh.superiorparietal_11)
4529	0.8599	(rh.inferiorparietal_5)(rh.inferiorparietal_6)
4530	0.8599	(rh.inferiortemporal_2)(rh.middletemporal_7)(rh.middletemporal_8)
4531	0.8599	(rh.insula_7)(rh.rostralmiddlefrontal_5)
4532	0.8599	(rh.lateraloccipital_1)(rh.pericalcarine_2)(rh.superiorparietal_13)
4533	0.8599	(rh.lateraloccipital_4)(rh.lateraloccipital_8)(rh.superiorparietal_11)
4534	0.8599	(rh.lateraloccipital_4)(rh.pericalcarine_1)(rh.pericalcarine_3)
4535	0.8599	(rh.lateralorbitofrontal_6)(rh.parstriangularis_1)(rh.rostralmiddlefrontal_12)(rh.rostralmiddlefrontal_8)
4536	0.8599	(rh.medialorbitofrontal_2)(rh.superiorfrontal_1)(rh.superiorfrontal_2)
4537	0.8599	(rh.paracentral_3)(rh.precentral_14)(rh.precentral_15)
4538	0.8599	(rh.postcentral_1)(rh.precentral_4)(rh.precentral_6)
4539	0.8599	(rh.postcentral_1)(rh.precentral_6)(rh.precentral_7)
4540	0.8599	(rh.rostralmiddlefrontal_11)(rh.rostralmiddlefrontal_12)(rh.rostralmiddlefrontal_8)
4541	0.8599	(rh.rostralmiddlefrontal_8)(rh.superiorfrontal_1)
4542	0.8599	(rh.superiorfrontal_11)(rh.superiorfrontal_13)(rh.superiorfrontal_7)
4543	0.8599	(Right-Accumbens-area)(Right-Amygdala)(Right-Hippocampus)(Right-Pallidum)(Right-Putamen)(Right-Thalamus-Proper)
4544	0.8599	(Right-Accumbens-area)(Right-Amygdala)(Right-Hippocampus)(Right-Pallidum)(Right-Putamen)
4545	0.8599	(Right-Accumbens-area)(Right-Amygdala)(Right-Hippocampus)(Right-Putamen)(Right-Thalamus-Proper)
4546	0.8599	(Right-Accumbens-area)(Right-Amygdala)(Right-Hippocampus)(Right-Putamen)
4547	0.8599	(Right-Caudate)(rh.lateralorbitofrontal_2)(rh.lateralorbitofrontal_7)
4548	0.8599	(Right-Caudate)(rh.lateralorbitofrontal_5)(rh.lateralorbitofrontal_7)(rh.medialorbitofrontal_2)
4549	0.8599	(Right-Caudate)(rh.parsopercularis_2)
4550	0.8599	(Right-Caudate)(Right-Putamen)(rh.caudalmiddlefrontal_4)(rh.rostralmiddlefrontal_3)

4551	0.8599	(Right-Caudate)(Right-Putamen)(rh.lateralorbitofrontal_2)(rh.lateralorbitofrontal_7)
4552	0.8599	(Right-Hippocampus)(rh.inferiorparietal_10)(rh.lateraloccipital_10)
4553	0.8599	(Right-Hippocampus)(rh.insula_2)(rh.insula_4)(rh.insula_5)
4554	0.8599	(Right-Hippocampus)(rh.precuneus_2)(rh.precuneus_4)
4555	0.8599	(Right-Hippocampus)(Right-Putamen)(rh.insula_2)(rh.insula_4)(rh.insula_5)
4556	0.8599	(Right-Hippocampus)(Right-Putamen)(Right-Thalamus-Proper)(rh.insula_1)(rh.insula_2)
4557	0.8599	(Right-Hippocampus)(Right-Thalamus-Proper)(rh.insula_1)(rh.insula_2)
4558	0.8599	(Right-Putamen)(rh.lateralorbitofrontal_6)(rh.rostralmiddlefrontal_12)(rh.rostralmiddlefrontal_8)
4559	0.8599	(Right-Putamen)(rh.lateralorbitofrontal_7)(rh.parstriangularis_1)(rh.rostralmiddlefrontal_8)
4560	0.8599	(Right-Putamen)(rh.parstriangularis_1)(rh.rostralmiddlefrontal_12)(rh.rostralmiddlefrontal_3)
4561	0.8599	(Right-Putamen)(rh.precentral_13)(rh.precentral_8)
4562	0.8599	(Right-Putamen)(rh.rostralmiddlefrontal_5)(rh.rostralmiddlefrontal_8)(rh.superiorfrontal_2)
4563	0.8599	(Right-Putamen)(rh.rostralmiddlefrontal_9)(rh.superiorfrontal_2)
4564	0.85749	(Left-Accumbens-area)(Left-Putamen)(lh.lateralorbitofrontal_2)
4565	0.85749	(Left-Accumbens-area)(Left-Thalamus-Proper)(Right-Thalamus-Proper)
4566	0.85749	(Left-Accumbens-area)(lh.lateralorbitofrontal_3)
4567	0.85749	(Left-Caudate)(Left-Pallidum)(Left-Putamen)(Left-Thalamus-Proper)(lh.insula_1)(lh.precentral_11)
4568	0.85749	(Left-Caudate)(Left-Pallidum)(Left-Thalamus-Proper)(lh.insula_1)(lh.precentral_11)
4569	0.85749	(Left-Caudate)(Left-Putamen)(lh.caudalanteriorcingulate_2)(lh.caudalmiddlefrontal_5)
4570	0.85749	(Left-Caudate)(Left-Putamen)(lh.insula_6)(lh.parsopercularis_3)(lh.parstriangularis_3)
4571	0.85749	(Left-Caudate)(Left-Putamen)(lh.lateralorbitofrontal_4)(lh.rostralanteriorcingulate_1)
4572	0.85749	(Left-Caudate)(Left-Putamen)(lh.lateralorbitofrontal_6)(lh.parstriangularis_3)(lh.rostralmiddlefrontal_9)
4573	0.85749	(Left-Caudate)(Left-Putamen)(lh.lateralorbitofrontal_7)(lh.parstriangularis_3)(lh.rostralmiddlefrontal_12)
4574	0.85749	(Left-Caudate)(Left-Putamen)(lh.paracentral_5)
4575	0.85749	(Left-Caudate)(Left-Putamen)(lh.rostralanteriorcingulate_1)(lh.rostralanteriorcingulate_2)(lh.rostralmiddlefrontal_4)
4576	0.85749	(Left-Caudate)(Left-Putamen)(lh.rostralanteriorcingulate_1)(lh.rostralmiddlefrontal_1)
4577	0.85749	(Left-Caudate)(Left-Putamen)(lh.rostralanteriorcingulate_2)(lh.superiorfrontal_11)
4578	0.85749	(Left-Caudate)(Left-Thalamus-Proper)(lh.supramarginal_1)
4579	0.85749	(Left-Caudate)(lh.caudalanteriorcingulate_1)(lh.caudalanteriorcingulate_2)(lh.posteriorcingulate_1)
4580	0.85749	(Left-Caudate)(lh.caudalanteriorcingulate_1)(lh.posteriorcingulate_1)
4581	0.85749	(Left-Caudate)(lh.caudalanteriorcingulate_2)(lh.superiorfrontal_10)(lh.superiorfrontal_8)
4582	0.85749	(Left-Caudate)(lh.caudalanteriorcingulate_2)(lh.superiorfrontal_11)(lh.superiorfrontal_12)(lh.superiorfrontal_14)(lh.superiorfrontal_8)
4583	0.85749	(Left-Caudate)(lh.caudalmiddlefrontal_1)(lh.caudalmiddlefrontal_3)(lh.rostralmiddlefrontal_4)
4584	0.85749	(Left-Caudate)(lh.caudalmiddlefrontal_1)(lh.rostralmiddlefrontal_1)
4585	0.85749	(Left-Caudate)(lh.caudalmiddlefrontal_5)(lh.precentral_9)
4586	0.85749	(Left-Caudate)(lh.isthmuscingulate_2)
4587	0.85749	(Left-Caudate)(lh.lateralorbitofrontal_4)(lh.rostralanteriorcingulate_1)
4588	0.85749	(Left-Caudate)(lh.lateralorbitofrontal_6)(lh.rostralmiddlefrontal_1)(lh.rostralmiddlefrontal_9)
4589	0.85749	(Left-Caudate)(lh.precentral_4)(lh.superiorfrontal_12)
4590	0.85749	(Left-Caudate)(lh.precentral_7)(lh.precentral_9)
4591	0.85749	(Left-Caudate)(lh.rostralanteriorcingulate_2)(lh.rostralmiddlefrontal_1)
4592	0.85749	(Left-Caudate)(lh.rostralmiddlefrontal_4)(lh.rostralmiddlefrontal_8)
4593	0.85749	(Left-Caudate)(lh.rostralmiddlefrontal_9)(lh.superiorfrontal_3)
4594	0.85749	(Left-Caudate)(lh.superiorfrontal_10)(lh.superiorfrontal_14)
4595	0.85749	(Left-Caudate)(Right-Caudate)(rh.rostralanteriorcingulate_1)
4596	0.85749	(Left-Hippocampus)(lh.bankssts_2)(lh.superiortemporal_6)
4597	0.85749	(Left-Hippocampus)(lh.fusiform_4)
4598	0.85749	(Left-Hippocampus)(lh.insula_4)(lh.superiortemporal_11)
4599	0.85749	(Left-Hippocampus)(lh.superiortemporal_9)
4600	0.85749	(Left-Putamen)(lh.caudalanteriorcingulate_2)(lh.caudalmiddlefrontal_1)(lh.caudalmiddlefrontal_3)

4601	0.85749	(Left-Putamen)(lh.caudalanteriorcingulate_2)(lh.superiorfrontal_11)(lh.superiorfrontal_14)
4602	0.85749	(Left-Putamen)(lh.caudalmiddlefrontal_3)(lh.insula_6)(lh.parsopercularis_3)
4603	0.85749	(Left-Putamen)(lh.lateralorbitofrontal_7)(lh.medialorbitofrontal_2)(lh.medialorbitofrontal_4)
4604	0.85749	(Left-Putamen)(lh.parsopercularis_1)(lh.precentral_14)
4605	0.85749	(Left-Putamen)(lh.rostralanteriorcingulate_1)(lh.superiorfrontal_3)(lh.superiorfrontal_4)
4606	0.85749	(Left-Putamen)(lh.rostralanteriorcingulate_2)(lh.rostralmiddlefrontal_4)(lh.rostralmiddlefrontal_9)
4607	0.85749	(Left-Putamen)(lh.rostralmiddlefrontal_3)
4608	0.85749	(lh.caudalanteriorcingulate_1)(lh.caudalanteriorcingulate_2)(lh.posteriorcingulate_1)
4609	0.85749	(lh.caudalanteriorcingulate_1)(lh.caudalanteriorcingulate_2)(lh.superiorfrontal_11)
4610	0.85749	(lh.caudalanteriorcingulate_1)(lh.posteriorcingulate_1)
4611	0.85749	(lh.caudalanteriorcingulate_2)(lh.posteriorcingulate_3)(lh.superiorfrontal_8)
4612	0.85749	(lh.caudalanteriorcingulate_2)(lh.rostralanteriorcingulate_1)(lh.superiorfrontal_4)
4613	0.85749	(lh.caudalmiddlefrontal_1)(lh.rostralmiddlefrontal_1)(lh.rostralmiddlefrontal_4)
4614	0.85749	(lh.cuneus_1)(lh.cuneus_2)(lh.superiorparietal_12)
4615	0.85749	(lh.fusiform_2)(lh.lateraloccipital_7)(lh.lingual_1)
4616	0.85749	(lh.fusiform_2)(lh.pericalcarine_2)
4617	0.85749	(lh.fusiform_4)(lh.fusiform_6)(lh.fusiform_7)
4618	0.85749	(lh.fusiform_4)(lh.inferiortemporal_6)
4619	0.85749	(lh.fusiform_5)(lh.inferiortemporal_7)
4620	0.85749	(lh.insula_1)(lh.supramarginal_4)(lh.supramarginal_9)
4621	0.85749	(lh.insula_6)(lh.parsopercularis_1)(lh.parsopercularis_3)(lh.parsopercularis_4)
4622	0.85749	(lh.lateraloccipital_2)(lh.lateraloccipital_3)(lh.pericalcarine_1)(lh.pericalcarine_2)
4623	0.85749	(lh.lateraloccipital_2)(lh.lateraloccipital_7)
4624	0.85749	(lh.lateraloccipital_6)(lh.lateraloccipital_7)(lh.lingual_1)
4625	0.85749	(lh.lateralorbitofrontal_4)(lh.parstriangularis_1)
4626	0.85749	(lh.lateralorbitofrontal_5)(lh.lateralorbitofrontal_6)
4627	0.85749	(lh.lateralorbitofrontal_6)(lh.lateralorbitofrontal_7)(lh.rostralmiddlefrontal_6)(lh.rostralmiddlefrontal_9)
4628	0.85749	(lh.lateralorbitofrontal_6)(lh.medialorbitofrontal_2)
4629	0.85749	(lh.lateralorbitofrontal_6)(lh.rostralmiddlefrontal_1)(lh.rostralmiddlefrontal_12)
4630	0.85749	(lh.lateralorbitofrontal_7)(lh.parstriangularis_3)(lh.rostralanteriorcingulate_1)(lh.rostralmiddlefrontal_12)(lh.rostralmiddlefrontal_9)
4631	0.85749	(lh.lateralorbitofrontal_7)(lh.rostralmiddlefrontal_12)(lh.superiorfrontal_3)
4632	0.85749	(lh.parsopercularis_1)(lh.parsopercularis_4)(lh.precentral_16)
4633	0.85749	(lh.postcentral_8)(lh.postcentral_9)
4634	0.85749	(lh.posteriorcingulate_3)(lh.posteriorcingulate_4)
4635	0.85749	(lh.precuneus_10)(lh.precuneus_8)(lh.superiorparietal_11)
4636	0.85749	(lh.precuneus_3)(lh.superiorparietal_5)(lh.superiorparietal_7)
4637	0.85749	(lh.precuneus_4)(lh.superiorparietal_8)
4638	0.85749	(lh.precuneus_6)(lh.superiorparietal_5)(lh.superiorparietal_7)
4639	0.85749	(lh.rostralanteriorcingulate_2)(lh.superiorfrontal_10)(lh.superiorfrontal_11)
4640	0.85749	(lh.rostralmiddlefrontal_1)(lh.rostralmiddlefrontal_5)(lh.rostralmiddlefrontal_7)
4641	0.85749	(lh.superiorfrontal_10)(lh.superiorfrontal_3)(lh.superiorfrontal_4)(lh.superiorfrontal_6)
4642	0.85749	(lh.superiorfrontal_11)(lh.superiorfrontal_13)(lh.superiorfrontal_8)(lh.superiorfrontal_9)
4643	0.85749	(lh.superiorfrontal_14)(lh.superiorfrontal_15)(lh.superiorfrontal_8)
4644	0.85749	(lh.superiorfrontal_15)(lh.superiorfrontal_8)
4645	0.85749	(lh.superiorfrontal_3)(lh.superiorfrontal_4)(lh.superiorfrontal_7)
4646	0.85749	(lh.superiorfrontal_3)(lh.superiorfrontal_7)
4647	0.85749	(rh.bankssts_1)(rh.inferiortemporal_7)(rh.middletemporal_1)
4648	0.85749	(rh.bankssts_2)(rh.inferiortemporal_7)
4649	0.85749	(rh.bankssts_2)(rh.insula_2)(rh.superiortemporal_1)
4650	0.85749	(rh.caudalmiddlefrontal_5)(rh.superiorfrontal_13)

4651	0.85749	(rh.fusiform_1)(rh.fusiform_2)(rh.lateraloccipital_10)(rh.lingual_7)
4652	0.85749	(rh.fusiform_1)(rh.lateraloccipital_10)(rh.lateraloccipital_8)(rh.lingual_7)
4653	0.85749	(rh.fusiform_7)(rh.inferiortemporal_6)
4654	0.85749	(rh.inferiorparietal_1)(rh.supramarginal_8)
4655	0.85749	(rh.inferiorparietal_10)(rh.inferiorparietal_11)(rh.inferiorparietal_12)(rh.lateraloccipital_8)
4656	0.85749	(rh.inferiorparietal_10)(rh.lateraloccipital_4)(rh.lateraloccipital_8)
4657	0.85749	(rh.lateraloccipital_3)(rh.lateraloccipital_5)
4658	0.85749	(rh.lateraloccipital_8)(rh.pericalcarine_3)
4659	0.85749	(rh.lateralorbitofrontal_7)(rh.medialorbitofrontal_1)
4660	0.85749	(rh.lingual_7)(rh.precuneus_2)(rh.superiorparietal_11)
4661	0.85749	(rh.middletemporal_2)(rh.middletemporal_6)
4662	0.85749	(rh.paracentral_3)(rh.paracentral_4)(rh.precentral_14)
4663	0.85749	(rh.paracentral_5)(rh.paracentral_6)(rh.precentral_15)
4664	0.85749	(rh.paracentral_6)(rh.superiorparietal_1)(rh.superiorparietal_2)
4665	0.85749	(rh.parstriangularis_2)(rh.rostralmiddlefrontal_8)
4666	0.85749	(rh.postcentral_2)(rh.supramarginal_7)
4667	0.85749	(rh.posteriorcingulate_4)(rh.precuneus_10)
4668	0.85749	(rh.precentral_14)(rh.superiorfrontal_17)
4669	0.85749	(rh.precuneus_4)(rh.superiorparietal_1)(rh.superiorparietal_10)(rh.superiorparietal_7)
4670	0.85749	(rh.superiorfrontal_2)(rh.superiorfrontal_3)(rh.superiorfrontal_5)
4671	0.85749	(Right-Caudate)(rh.lateralorbitofrontal_2)(rh.lateralorbitofrontal_4)
4672	0.85749	(Right-Caudate)(rh.lateralorbitofrontal_7)(rh.rostralmiddlefrontal_12)
4673	0.85749	(Right-Caudate)(rh.superiorfrontal_13)(rh.superiorfrontal_9)
4674	0.85749	(Right-Caudate)(Right-Pallidum)(rh.precentral_5)
4675	0.85749	(Right-Caudate)(Right-Pallidum)(Right-Putamen)(rh.precentral_5)
4676	0.85749	(Right-Caudate)(Right-Putamen)(rh.lateralorbitofrontal_2)(rh.lateralorbitofrontal_4)
4677	0.85749	(Right-Caudate)(Right-Putamen)(rh.lateralorbitofrontal_7)(rh.rostralmiddlefrontal_12)
4678	0.85749	(Right-Caudate)(Right-Putamen)(rh.rostralmiddlefrontal_1)
4679	0.85749	(Right-Caudate)(Right-Putamen)(Right-Thalamus-Proper)(rh.posteriorcingulate_2)(rh.precentral_13)
4680	0.85749	(Right-Hippocampus)(rh.bankssts_2)(rh.bankssts_3)
4681	0.85749	(Right-Putamen)(rh.caudalmiddlefrontal_1)(rh.precentral_10)
4682	0.85749	(Right-Putamen)(rh.insula_1)(rh.precentral_11)(rh.supramarginal_1)
4683	0.85749	(Right-Putamen)(rh.insula_1)(rh.precentral_7)(rh.supramarginal_1)
4684	0.85749	(Right-Putamen)(Right-Thalamus-Proper)(rh.insula_2)(rh.precentral_13)
4685	0.85749	(Right-Putamen)(Right-Thalamus-Proper)(rh.posteriorcingulate_2)(rh.precentral_13)
4686	0.85749	(Right-Thalamus-Proper)(rh.insula_2)(rh.precentral_13)
4687	0.85507	(Left-Amygdala)(lh.inferiortemporal_1)(lh.insula_4)
4688	0.85507	(Left-Caudate)(Left-Putamen)(lh.caudalanteriorcingulate_2)(lh.superiorfrontal_12)(lh.superiorfrontal_14)
4689	0.85507	(Left-Caudate)(Left-Putamen)(lh.parstriangularis_3)(lh.rostralanteriorcingulate_1)(lh.rostralmiddlefrontal_12)
4690	0.85507	(Left-Caudate)(Left-Putamen)(lh.posteriorcingulate_3)(lh.superiorfrontal_14)
4691	0.85507	(Left-Caudate)(Left-Thalamus-Proper)(lh.insula_1)(lh.postcentral_8)
4692	0.85507	(Left-Caudate)(lh.caudalanteriorcingulate_1)(lh.caudalanteriorcingulate_2)(lh.superiorfrontal_11)
4693	0.85507	(Left-Caudate)(lh.caudalmiddlefrontal_3)(lh.parsopercularis_3)
4694	0.85507	(Left-Caudate)(lh.insula_6)(lh.parstriangularis_3)(lh.rostralmiddlefrontal_9)
4695	0.85507	(Left-Caudate)(lh.medialorbitofrontal_2)(lh.rostralmiddlefrontal_9)(lh.superiorfrontal_4)
4696	0.85507	(Left-Caudate)(lh.parstriangularis_3)(lh.rostralanteriorcingulate_1)(lh.rostralanteriorcingulate_2)
4697	0.85507	(Left-Caudate)(lh.rostralanteriorcingulate_2)(lh.rostralmiddlefrontal_8)
4698	0.85507	(Left-Caudate)(lh.rostralanteriorcingulate_2)(lh.superiorfrontal_4)(lh.superiorfrontal_8)
4699	0.85507	(Left-Caudate)(lh.rostralmiddlefrontal_1)(lh.superiorfrontal_4)
4700	0.85507	(Left-Caudate)(lh.rostralmiddlefrontal_3)(lh.rostralmiddlefrontal_4)

4701	0.85507	(Left-Caudate)(lh.superiorfrontal_14)(lh.superiorfrontal_4)(lh.superiorfrontal_8)
4702	0.85507	(Left-Hippocampus)(lh.bankssts_2)(lh.fusiform_5)
4703	0.85507	(Left-Hippocampus)(lh.fusiform_7)(lh.fusiform_8)
4704	0.85507	(Left-Hippocampus)(lh.middletemporal_4)
4705	0.85507	(Left-Putamen)(lh.insula_1)(lh.postcentral_8)(lh.precentral_7)
4706	0.85507	(Left-Putamen)(lh.lateralorbitofrontal_7)(lh.medialorbitofrontal_2)(lh.parstriangularis_3)
4707	0.85507	(Left-Putamen)(lh.lateralorbitofrontal_7)(lh.medialorbitofrontal_2)(lh.rostralanteriorcingulate_1)(lh.rostralmiddlefrontal_12)(lh.rostralmiddlefrontal_9)
4708	0.85507	(Left-Putamen)(lh.lateralorbitofrontal_7)(lh.parstriangularis_3)(lh.rostralanteriorcingulate_1)(lh.rostralmiddlefrontal_9)
4709	0.85507	(Left-Putamen)(lh.rostralanteriorcingulate_2)(lh.rostralmiddlefrontal_8)
4710	0.85507	(Left-Putamen)(lh.rostralmiddlefrontal_12)(lh.rostralmiddlefrontal_4)
4711	0.85507	(lh.bankssts_2)(lh.fusiform_7)(lh.inferiortemporal_6)
4712	0.85507	(lh.caudalmiddlefrontal_1)(lh.caudalmiddlefrontal_3)(lh.superiorfrontal_11)
4713	0.85507	(lh.inferiorparietal_4)(lh.lateraloccipital_9)(lh.lingual_8)
4714	0.85507	(lh.inferiorparietal_5)(lh.insula_2)
4715	0.85507	(lh.inferiortemporal_6)(lh.inferiortemporal_7)(lh.middletemporal_3)
4716	0.85507	(lh.insula_1)(lh.superiorparietal_3)(lh.supramarginal_9)
4717	0.85507	(lh.insula_3)(lh.postcentral_14)
4718	0.85507	(lh.insula_6)(lh.parsopercularis_1)(lh.precentral_14)
4719	0.85507	(lh.insula_6)(lh.rostralanteriorcingulate_1)
4720	0.85507	(lh.lateraloccipital_11)(lh.lateraloccipital_6)(lh.lingual_1)(lh.pericalcarine_3)
4721	0.85507	(lh.lateraloccipital_6)(lh.lateraloccipital_7)(lh.lateraloccipital_9)
4722	0.85507	(lh.lateraloccipital_9)(lh.lingual_6)(lh.lingual_8)
4723	0.85507	(lh.lateraloccipital_9)(lh.pericalcarine_3)
4724	0.85507	(lh.lateralorbitofrontal_4)(lh.lateralorbitofrontal_6)(lh.lateralorbitofrontal_7)(lh.parstriangularis_3)(lh.rostralmiddlefrontal_12)(lh.rostralmiddlefrontal_9)
4725	0.85507	(lh.lateralorbitofrontal_4)(lh.rostralanteriorcingulate_1)(lh.rostralmiddlefrontal_12)
4726	0.85507	(lh.lateralorbitofrontal_6)(lh.parstriangularis_3)(lh.rostralmiddlefrontal_1)(lh.rostralmiddlefrontal_12)
4727	0.85507	(lh.lateralorbitofrontal_7)(lh.medialorbitofrontal_2)(lh.parstriangularis_3)(lh.rostralmiddlefrontal_9)
4728	0.85507	(lh.lateralorbitofrontal_7)(lh.medialorbitofrontal_2)(lh.rostralmiddlefrontal_12)(lh.superiorfrontal_3)
4729	0.85507	(lh.lingual_2)(lh.pericalcarine_2)
4730	0.85507	(lh.paracentral_5)(lh.superiorfrontal_12)(lh.superiorfrontal_15)
4731	0.85507	(lh.parsopercularis_2)(lh.parstriangularis_2)(lh.parstriangularis_3)
4732	0.85507	(lh.precentral_11)(lh.supramarginal_9)
4733	0.85507	(lh.precuneus_8)(lh.superiorparietal_9)
4734	0.85507	(lh.rostralanteriorcingulate_1)(lh.rostralmiddlefrontal_4)(lh.rostralmiddlefrontal_9)(lh.superiorfrontal_4)
4735	0.85507	(lh.rostralanteriorcingulate_2)(lh.superiorfrontal_10)(lh.superiorfrontal_4)(lh.superiorfrontal_8)
4736	0.85507	(lh.rostralmiddlefrontal_1)(lh.rostralmiddlefrontal_2)(lh.rostralmiddlefrontal_3)
4737	0.85507	(lh.rostralmiddlefrontal_1)(lh.rostralmiddlefrontal_4)(lh.rostralmiddlefrontal_7)
4738	0.85507	(lh.rostralmiddlefrontal_1)(lh.rostralmiddlefrontal_4)(lh.superiorfrontal_4)
4739	0.85507	(lh.rostralmiddlefrontal_4)(lh.rostralmiddlefrontal_8)(lh.superiorfrontal_3)
4740	0.85507	(lh.rostralmiddlefrontal_8)(lh.superiorfrontal_3)(lh.superiorfrontal_4)(lh.superiorfrontal_5)
4741	0.85507	(lh.rostralmiddlefrontal_8)(lh.superiorfrontal_3)(lh.superiorfrontal_5)
4742	0.85507	(lh.rostralmiddlefrontal_9)(lh.superiorfrontal_2)(lh.superiorfrontal_4)
4743	0.85507	(lh.superiorfrontal_1)(lh.superiorfrontal_2)(lh.superiorfrontal_3)(lh.superiorfrontal_4)
4744	0.85507	(lh.superiorfrontal_1)(lh.superiorfrontal_2)(lh.superiorfrontal_4)
4745	0.85507	(lh.superiorfrontal_10)(lh.superiorfrontal_3)(lh.superiorfrontal_4)(lh.superiorfrontal_5)
4746	0.85507	(lh.superiorfrontal_10)(lh.superiorfrontal_3)(lh.superiorfrontal_5)
4747	0.85507	(rh.bankssts_1)(rh.inferiortemporal_5)(rh.middletemporal_2)
4748	0.85507	(rh.caudalanteriorcingulate_2)(rh.superiorfrontal_11)
4749	0.85507	(rh.caudalmiddlefrontal_4)(rh.superiorfrontal_14)
4750	0.85507	(rh.fusiform_1)(rh.lateraloccipital_10)(rh.lingual_5)(rh.lingual_7)

4751	0.85507	(rh.inferiorparietal_11)(rh.lateraloccipital_4)(rh.lateraloccipital_8)
4752	0.85507	(rh.lateraloccipital_4)(rh.lateraloccipital_7)(rh.pericalcarine_4)
4753	0.85507	(rh.lateralorbitofrontal_6)(rh.parstriangularis_2)
4754	0.85507	(rh.lateralorbitofrontal_6)(rh.rostralmiddlefrontal_12)(rh.rostralmiddlefrontal_7)
4755	0.85507	(rh.lateralorbitofrontal_7)(rh.rostralmiddlefrontal_12)(rh.rostralmiddlefrontal_8)(rh.superiorfrontal_2)
4756	0.85507	(rh.paracentral_4)(rh.precentral_13)(rh.superiorfrontal_17)
4757	0.85507	(rh.parstriangularis_1)(rh.rostralanteriorcingulate_2)
4758	0.85507	(rh.postcentral_4)(rh.precentral_1)
4759	0.85507	(rh.precentral_13)(rh.precentral_6)
4760	0.85507	(rh.precuneus_4)(rh.superiorparietal_7)(rh.superiorparietal_8)
4761	0.85507	(rh.precuneus_7)(rh.superiorparietal_10)(rh.superiorparietal_7)(rh.superiorparietal_8)
4762	0.85507	(rh.rostralmiddlefrontal_1)(rh.superiorfrontal_5)
4763	0.85507	(rh.rostralmiddlefrontal_2)(rh.rostralmiddlefrontal_3)(rh.rostralmiddlefrontal_5)
4764	0.85507	(rh.superiorfrontal_13)(rh.superiorfrontal_7)(rh.superiorfrontal_8)(rh.superiorfrontal_9)
4765	0.85507	(rh.superiorfrontal_2)(rh.superiorfrontal_4)(rh.superiorfrontal_6)(rh.superiorfrontal_7)
4766	0.85507	(rh.supramarginal_6)(rh.supramarginal_7)(rh.supramarginal_8)(rh.supramarginal_9)
4767	0.85507	(Right-Amygdala)(Right-Hippocampus)(rh.insula_4)(rh.insula_5)
4768	0.85507	(Right-Amygdala)(Right-Hippocampus)(Right-Putamen)(rh.insula_4)(rh.insula_5)
4769	0.85507	(Right-Caudate)(rh.caudalmiddlefrontal_4)(rh.parsopercularis_4)(rh.rostralmiddlefrontal_3)
4770	0.85507	(Right-Caudate)(rh.lateralorbitofrontal_7)(rh.superiorfrontal_2)
4771	0.85507	(Right-Caudate)(rh.parsopercularis_2)(rh.parsopercularis_4)
4772	0.85507	(Right-Caudate)(rh.precentral_8)
4773	0.85507	(Right-Caudate)(rh.rostralmiddlefrontal_9)
4774	0.85507	(Right-Caudate)(Right-Pallidum)(rh.precentral_7)
4775	0.85507	(Right-Caudate)(Right-Pallidum)(Right-Putamen)(rh.precentral_7)
4776	0.85507	(Right-Caudate)(Right-Putamen)(rh.caudalmiddlefrontal_4)(rh.rostralmiddlefrontal_5)
4777	0.85507	(Right-Caudate)(Right-Putamen)(rh.rostralmiddlefrontal_5)(rh.rostralmiddlefrontal_8)
4778	0.85507	(Right-Hippocampus)(rh.inferiortemporal_2)(rh.middletemporal_4)
4779	0.85507	(Right-Hippocampus)(rh.isthmuscingulate_2)(rh.precuneus_4)(rh.precuneus_7)
4780	0.85507	(Right-Hippocampus)(rh.superiortemporal_1)(rh.superiortemporal_9)
4781	0.85507	(Right-Pallidum)(rh.parsopercularis_4)
4782	0.85507	(Right-Pallidum)(Right-Putamen)(rh.parsopercularis_4)
4783	0.85507	(Right-Putamen)(rh.caudalmiddlefrontal_5)(rh.rostralmiddlefrontal_1)
4784	0.85507	(Right-Putamen)(rh.postcentral_1)(rh.precentral_1)(rh.precentral_6)
4785	0.85507	(Right-Putamen)(rh.postcentral_5)(rh.precentral_13)
4786	0.85507	(Right-Putamen)(rh.superiorfrontal_5)
4787	0.85266	(Left-Caudate)(Left-Hippocampus)(Left-Pallidum)(Left-Putamen)(Left-Thalamus-Proper)(lh.insula_1)
4788	0.85266	(Left-Caudate)(Left-Hippocampus)(Left-Pallidum)(Left-Putamen)(lh.insula_1)
4789	0.85266	(Left-Caudate)(Left-Hippocampus)(Left-Pallidum)(Left-Thalamus-Proper)(lh.insula_1)
4790	0.85266	(Left-Caudate)(Left-Hippocampus)(Left-Pallidum)(lh.insula_1)
4791	0.85266	(Left-Caudate)(Left-Pallidum)(Left-Putamen)(lh.posteriorcingulate_3)
4792	0.85266	(Left-Caudate)(Left-Putamen)(Left-Thalamus-Proper)(lh.insula_1)(lh.postcentral_8)
4793	0.85266	(Left-Caudate)(Left-Putamen)(lh.caudalanteriorcingulate_2)(lh.caudalmiddlefrontal_1)(lh.caudalmiddlefrontal_3)
4794	0.85266	(Left-Caudate)(Left-Putamen)(lh.caudalmiddlefrontal_3)(lh.parsopercularis_3)
4795	0.85266	(Left-Caudate)(Left-Putamen)(lh.lateralorbitofrontal_6)(lh.rostralmiddlefrontal_12)(lh.rostralmiddlefrontal_9)
4796	0.85266	(Left-Caudate)(Left-Putamen)(lh.lateralorbitofrontal_7)(lh.medialorbitofrontal_5)
4797	0.85266	(Left-Caudate)(Left-Putamen)(lh.lateralorbitofrontal_7)(lh.parstriangularis_3)(lh.rostralanteriorcingulate_1)
4798	0.85266	(Left-Caudate)(Left-Putamen)(lh.rostralanteriorcingulate_2)(lh.rostralmiddlefrontal_4)(lh.superiorfrontal_4)
4799	0.85266	(Left-Caudate)(Left-Putamen)(lh.rostralmiddlefrontal_4)(lh.rostralmiddlefrontal_9)(lh.superiorfrontal_4)
4800	0.85266	(Left-Caudate)(lh.lateralorbitofrontal_6)(lh.parstriangularis_3)(lh.rostralmiddlefrontal_1)

4801	0.85266	(Left-Caudate)(lh.posteriorcingulate_1)(lh.superiorfrontal_14)
4802	0.85266	(Left-Caudate)(lh.rostralmiddlefrontal_1)(lh.rostralmiddlefrontal_12)
4803	0.85266	(Left-Caudate)(rh.rostralanteriorcingulate_2)
4804	0.85266	(Left-Caudate)(Right-Caudate)(rh.rostralanteriorcingulate_2)
4805	0.85266	(Left-Hippocampus)(lh.fusiform_5)(lh.lingual_6)
4806	0.85266	(Left-Hippocampus)(lh.fusiform_7)(lh.superiortemporal_6)
4807	0.85266	(Left-Hippocampus)(lh.inferiortemporal_1)(lh.insula_4)
4808	0.85266	(Left-Pallidum)(Left-Putamen)(lh.insula_7)
4809	0.85266	(Left-Pallidum)(Left-Putamen)(lh.posteriorcingulate_3)
4810	0.85266	(Left-Pallidum)(Left-Putamen)(lh.precentral_11)(lh.precentral_9)
4811	0.85266	(Left-Pallidum)(lh.insula_7)
4812	0.85266	(Left-Pallidum)(lh.precentral_11)(lh.precentral_9)
4813	0.85266	(Left-Pallidum)(lh.precentral_7)
4814	0.85266	(Left-Putamen)(lh.caudalanteriorcingulate_2)(lh.rostralanteriorcingulate_2)(lh.superiorfrontal_4)
4815	0.85266	(Left-Putamen)(lh.insula_6)(lh.rostralanteriorcingulate_1)
4816	0.85266	(Left-Putamen)(lh.medialorbitofrontal_2)(lh.parstriangularis_3)(lh.rostralmiddlefrontal_12)
4817	0.85266	(Left-Putamen)(lh.medialorbitofrontal_2)(lh.rostralanteriorcingulate_1)(lh.superiorfrontal_4)
4818	0.85266	(Left-Putamen)(lh.rostralmiddlefrontal_12)(lh.rostralmiddlefrontal_4)(lh.rostralmiddlefrontal_9)
4819	0.85266	(Left-Putamen)(lh.rostralmiddlefrontal_4)(lh.superiorfrontal_11)(lh.superiorfrontal_4)
4820	0.85266	(Left-Thalamus-Proper)(lh.postcentral_8)(lh.precentral_4)
4821	0.85266	(lh.caudalanteriorcingulate_2)(lh.caudalmiddlefrontal_3)(lh.caudalmiddlefrontal_5)
4822	0.85266	(lh.caudalanteriorcingulate_2)(lh.posteriorcingulate_1)(lh.superiorfrontal_12)
4823	0.85266	(lh.caudalanteriorcingulate_2)(lh.posteriorcingulate_1)(lh.superiorfrontal_14)
4824	0.85266	(lh.caudalmiddlefrontal_3)(lh.insula_6)(lh.parstriangularis_3)(lh.rostralmiddlefrontal_1)
4825	0.85266	(lh.caudalmiddlefrontal_3)(lh.rostralmiddlefrontal_2)
4826	0.85266	(lh.cuneus_1)(lh.inferiorparietal_1)
4827	0.85266	(lh.cuneus_1)(lh.superiorparietal_12)(lh.superiorparietal_13)
4828	0.85266	(lh.fusiform_2)(lh.fusiform_5)(lh.lingual_8)
4829	0.85266	(lh.fusiform_3)(lh.fusiform_4)(lh.fusiform_5)
4830	0.85266	(lh.fusiform_5)(lh.inferiorparietal_5)
4831	0.85266	(lh.inferiorparietal_6)(lh.inferiorparietal_7)(lh.inferiorparietal_8)(lh.inferiorparietal_9)
4832	0.85266	(lh.insula_1)(lh.superiorparietal_3)(lh.supramarginal_1)
4833	0.85266	(lh.isthmuscingulate_3)(lh.parahippocampal_3)
4834	0.85266	(lh.lateraloccipital_11)(lh.lateraloccipital_5)
4835	0.85266	(lh.lateraloccipital_3)(lh.superiorparietal_12)
4836	0.85266	(lh.lateraloccipital_7)(lh.lingual_8)(lh.pericalcarine_3)
4837	0.85266	(lh.lateralorbitofrontal_4)(lh.parstriangularis_3)(lh.rostralanteriorcingulate_1)(lh.rostralmiddlefrontal_9)
4838	0.85266	(lh.lateralorbitofrontal_4)(lh.rostralmiddlefrontal_6)
4839	0.85266	(lh.lateralorbitofrontal_6)(lh.lateralorbitofrontal_7)(lh.rostralmiddlefrontal_11)
4840	0.85266	(lh.lateralorbitofrontal_7)(lh.medialorbitofrontal_1)(lh.medialorbitofrontal_2)(lh.rostralmiddlefrontal_12)
4841	0.85266	(lh.lateralorbitofrontal_7)(lh.medialorbitofrontal_4)(lh.medialorbitofrontal_5)
4842	0.85266	(lh.lingual_6)(lh.parahippocampal_3)
4843	0.85266	(lh.paracentral_4)(lh.precentral_2)(lh.precentral_4)
4844	0.85266	(lh.paracentral_4)(lh.superiorfrontal_14)
4845	0.85266	(lh.parstriangularis_2)(lh.parstriangularis_3)(lh.rostralmiddlefrontal_6)
4846	0.85266	(lh.posteriorcingulate_3)(lh.precentral_11)
4847	0.85266	(lh.precentral_7)(lh.supramarginal_9)
4848	0.85266	(lh.precuneus_2)(lh.superiorparietal_5)
4849	0.85266	(lh.precuneus_3)(lh.superiorparietal_2)
4850	0.85266	(lh.precuneus_4)(lh.superiorparietal_9)

4851	0.85266	(lh.rostralanteriorcingulate_2)(lh.rostralmiddlefrontal_4)(lh.rostralmiddlefrontal_8)
4852	0.85266	(lh.rostralanteriorcingulate_2)(lh.superiorfrontal_11)(lh.superiorfrontal_4)(lh.superiorfrontal_8)
4853	0.85266	(lh.superiorfrontal_11)(lh.superiorfrontal_4)(lh.superiorfrontal_6)(lh.superiorfrontal_8)
4854	0.85266	(lh.superiorfrontal_11)(lh.superiorfrontal_6)(lh.superiorfrontal_8)
4855	0.85266	(lh.superiorfrontal_14)(lh.superiorfrontal_4)(lh.superiorfrontal_9)
4856	0.85266	(rh.bankssts_1)(rh.bankssts_2)(rh.middletemporal_1)
4857	0.85266	(rh.fusiform_2)(rh.fusiform_3)(rh.fusiform_4)
4858	0.85266	(rh.fusiform_3)(rh.inferiorparietal_10)
4859	0.85266	(rh.fusiform_3)(rh.inferiortemporal_6)(rh.inferiortemporal_7)
4860	0.85266	(rh.inferiortemporal_5)(rh.inferiortemporal_6)(rh.inferiortemporal_7)
4861	0.85266	(rh.insula_2)(rh.precentral_13)(rh.precentral_7)
4862	0.85266	(rh.insula_7)(rh.parsopercularis_4)(rh.parstriangularis_1)
4863	0.85266	(rh.lateralorbitofrontal_6)(rh.lateralorbitofrontal_7)(rh.rostralmiddlefrontal_12)(rh.rostralmiddlefrontal_13)
4864	0.85266	(rh.lateralorbitofrontal_6)(rh.parstriangularis_1)(rh.rostralmiddlefrontal_3)
4865	0.85266	(rh.lateralorbitofrontal_7)(rh.medialorbitofrontal_2)(rh.rostralmiddlefrontal_12)(rh.superiorfrontal_2)
4866	0.85266	(rh.lateralorbitofrontal_7)(rh.parstriangularis_4)
4867	0.85266	(rh.parsopercularis_4)(rh.rostralmiddlefrontal_5)
4868	0.85266	(rh.parsopercularis_4)(rh.superiorfrontal_7)
4869	0.85266	(rh.parstriangularis_1)(rh.parstriangularis_2)(rh.parstriangularis_3)(rh.parstriangularis_4)
4870	0.85266	(rh.pericalcarine_2)(rh.pericalcarine_4)
4871	0.85266	(rh.postcentral_10)(rh.supramarginal_8)
4872	0.85266	(rh.postcentral_5)(rh.supramarginal_6)
4873	0.85266	(rh.precentral_12)(rh.precentral_7)
4874	0.85266	(rh.precuneus_1)(rh.precuneus_2)(rh.superiorparietal_11)
4875	0.85266	(rh.precuneus_8)(rh.superiorparietal_5)
4876	0.85266	(rh.superiorparietal_2)(rh.superiorparietal_7)
4877	0.85266	(Right-Amygdala)(Right-Hippocampus)(rh.fusiform_8)
4878	0.85266	(Right-Caudate)(rh.precentral_5)(rh.precentral_6)
4879	0.85266	(Right-Caudate)(rh.rostralmiddlefrontal_3)(rh.rostralmiddlefrontal_8)
4880	0.85266	(Right-Caudate)(Right-Pallidum)(rh.parsopercularis_4)
4881	0.85266	(Right-Caudate)(Right-Pallidum)(rh.posteriorcingulate_2)(rh.precentral_13)
4882	0.85266	(Right-Caudate)(Right-Pallidum)(Right-Putamen)(rh.parsopercularis_4)
4883	0.85266	(Right-Hippocampus)(rh.bankssts_1)(rh.middletemporal_4)
4884	0.85266	(Right-Pallidum)(rh.posteriorcingulate_2)(rh.precentral_13)
4885	0.85266	(Right-Putamen)(rh.caudalmiddlefrontal_5)(rh.superiorfrontal_7)
4886	0.85266	(Right-Putamen)(rh.insula_2)(rh.precentral_13)(rh.precentral_7)
4887	0.85266	(Right-Putamen)(rh.lateralorbitofrontal_2)(rh.parstriangularis_1)(rh.rostralmiddlefrontal_12)(rh.rostralmiddlefrontal_8)
4888	0.85266	(Right-Putamen)(rh.lateralorbitofrontal_7)(rh.parstriangularis_1)(rh.rostralmiddlefrontal_12)(rh.rostralmiddlefrontal_8)
4889	0.85266	(Right-Putamen)(rh.rostralmiddlefrontal_11)(rh.rostralmiddlefrontal_8)
4890	0.85266	(Right-Putamen)(rh.rostralmiddlefrontal_8)(rh.rostralmiddlefrontal_9)
4891	0.85266	(Right-Putamen)(rh.superiorparietal_1)(rh.supramarginal_8)(rh.supramarginal_9)
4892	0.85024	(Left-Accumbens-area)(Left-Caudate)(Left-Putamen)(lh.medialorbitofrontal_4)
4893	0.85024	(Left-Accumbens-area)(Left-Putamen)(lh.lateralorbitofrontal_3)
4894	0.85024	(Left-Caudate)(Left-Putamen)(lh.caudalanteriorcingulate_2)(lh.superiorfrontal_11)(lh.superiorfrontal_14)
4895	0.85024	(Left-Caudate)(Left-Putamen)(lh.lateralorbitofrontal_4)(lh.parstriangularis_3)(lh.rostralmiddlefrontal_9)
4896	0.85024	(Left-Caudate)(Left-Putamen)(lh.lateralorbitofrontal_6)(lh.rostralmiddlefrontal_1)
4897	0.85024	(Left-Caudate)(Left-Putamen)(lh.lateralorbitofrontal_7)(lh.medialorbitofrontal_2)(lh.rostralanteriorcingulate_1)(lh.rostralmiddlefrontal_12)
4898	0.85024	(Left-Caudate)(Left-Putamen)(lh.lateralorbitofrontal_7)(lh.medialorbitofrontal_2)(lh.rostralanteriorcingulate_1)(lh.rostralmiddlefrontal_9)
4899	0.85024	(Left-Caudate)(Left-Putamen)(lh.parstriangularis_3)(lh.rostralanteriorcingulate_2)
4900	0.85024	(Left-Caudate)(Left-Putamen)(lh.superiorfrontal_3)

4901	0.85024	(Left-Caudate)(Left-Thalamus-Proper)(lh.postcentral_3)
4902	0.85024	(Left-Caudate)(lh.caudalanteriorcingulate_2)(lh.posteriorcingulate_1)(lh.superiorfrontal_12)
4903	0.85024	(Left-Caudate)(lh.caudalanteriorcingulate_2)(lh.superiorfrontal_4)(lh.superiorfrontal_8)
4904	0.85024	(Left-Caudate)(lh.caudalmiddlefrontal_3)(lh.insula_6)(lh.rostralmiddlefrontal_4)
4905	0.85024	(Left-Caudate)(lh.caudalmiddlefrontal_3)(lh.parstriangularis_3)(lh.rostralmiddlefrontal_1)
4906	0.85024	(Left-Caudate)(lh.caudalmiddlefrontal_3)(lh.rostralmiddlefrontal_1)(lh.rostralmiddlefrontal_9)
4907	0.85024	(Left-Caudate)(lh.caudalmiddlefrontal_3)(lh.rostralmiddlefrontal_3)
4908	0.85024	(Left-Caudate)(lh.caudalmiddlefrontal_4)(lh.caudalmiddlefrontal_6)(lh.precentral_9)
4909	0.85024	(Left-Caudate)(lh.lateralorbitofrontal_6)(lh.lateralorbitofrontal_7)(lh.rostralmiddlefrontal_12)
4910	0.85024	(Left-Caudate)(lh.lateralorbitofrontal_6)(lh.lateralorbitofrontal_7)(lh.rostralmiddlefrontal_9)
4911	0.85024	(Left-Caudate)(lh.lateralorbitofrontal_7)(lh.parstriangularis_3)(lh.rostralmiddlefrontal_12)(lh.rostralmiddlefrontal_9)
4912	0.85024	(Left-Caudate)(lh.medialorbitofrontal_5)(lh.rostralanteriorcingulate_1)
4913	0.85024	(Left-Caudate)(lh.parsopercularis_1)(lh.parsopercularis_3)
4914	0.85024	(Left-Caudate)(lh.postcentral_8)(lh.precentral_14)
4915	0.85024	(Left-Caudate)(lh.posteriorcingulate_3)(lh.precentral_11)
4916	0.85024	(Left-Caudate)(lh.superiorfrontal_11)(lh.superiorfrontal_12)(lh.superiorfrontal_4)
4917	0.85024	(Left-Hippocampus)(lh.insula_1)(lh.supramarginal_1)
4918	0.85024	(Left-Hippocampus)(lh.superiortemporal_3)(lh.superiortemporal_6)(lh.superiortemporal_8)
4919	0.85024	(Left-Pallidum)(Left-Putamen)(Left-Thalamus-Proper)(lh.precentral_9)
4920	0.85024	(Left-Pallidum)(Left-Putamen)(lh.precentral_7)
4921	0.85024	(Left-Pallidum)(Left-Thalamus-Proper)(lh.precentral_9)
4922	0.85024	(Left-Putamen)(lh.caudalanteriorcingulate_1)(lh.rostralmiddlefrontal_4)
4923	0.85024	(Left-Putamen)(lh.caudalanteriorcingulate_2)(lh.rostralanteriorcingulate_2)(lh.superiorfrontal_11)
4924	0.85024	(Left-Putamen)(lh.lateralorbitofrontal_4)(lh.lateralorbitofrontal_7)(lh.parstriangularis_3)(lh.rostralmiddlefrontal_12)(lh.rostralmiddlefrontal_9)
4925	0.85024	(Left-Putamen)(lh.lateralorbitofrontal_4)(lh.parstriangularis_3)(lh.rostralanteriorcingulate_1)
4926	0.85024	(Left-Putamen)(lh.lateralorbitofrontal_4)(lh.rostralanteriorcingulate_1)(lh.rostralmiddlefrontal_12)
4927	0.85024	(Left-Putamen)(lh.medialorbitofrontal_2)(lh.rostralanteriorcingulate_1)(lh.rostralmiddlefrontal_12)(lh.superiorfrontal_3)
4928	0.85024	(Left-Putamen)(lh.medialorbitofrontal_2)(lh.rostralanteriorcingulate_1)(lh.rostralmiddlefrontal_9)(lh.superiorfrontal_3)
4929	0.85024	(Left-Putamen)(lh.parsopercularis_1)(lh.parsopercularis_3)(lh.parstriangularis_3)(lh.rostralmiddlefrontal_1)
4930	0.85024	(Left-Putamen)(lh.parstriangularis_3)(lh.rostralanteriorcingulate_1)(lh.rostralanteriorcingulate_2)
4931	0.85024	(Left-Putamen)(lh.postcentral_8)(lh.precentral_11)(lh.precentral_4)
4932	0.85024	(Left-Putamen)(lh.rostralanteriorcingulate_1)(lh.rostralmiddlefrontal_4)(lh.superiorfrontal_4)
4933	0.85024	(lh.caudalanteriorcingulate_1)(lh.caudalanteriorcingulate_2)(lh.superiorfrontal_8)
4934	0.85024	(lh.caudalanteriorcingulate_2)(lh.superiorfrontal_14)(lh.superiorfrontal_4)
4935	0.85024	(lh.cuneus_1)(lh.pericalcarine_1)(lh.superiorparietal_14)
4936	0.85024	(lh.cuneus_2)(lh.lateraloccipital_1)
4937	0.85024	(lh.fusiform_1)(lh.lateraloccipital_7)
4938	0.85024	(lh.fusiform_2)(lh.lateraloccipital_10)(lh.lateraloccipital_7)
4939	0.85024	(lh.fusiform_2)(lh.lateraloccipital_7)(lh.lateraloccipital_8)
4940	0.85024	(lh.inferiorparietal_4)(lh.inferiorparietal_6)
4941	0.85024	(lh.inferiorparietal_5)(lh.lingual_8)
4942	0.85024	(lh.insula_1)(lh.precentral_16)
4943	0.85024	(lh.insula_1)(lh.superiorparietal_5)(lh.supramarginal_9)
4944	0.85024	(lh.insula_1)(lh.supramarginal_1)(lh.supramarginal_2)(lh.supramarginal_4)
4945	0.85024	(lh.insula_1)(lh.supramarginal_6)(lh.supramarginal_9)
4946	0.85024	(lh.insula_6)(lh.rostralmiddlefrontal_4)(lh.rostralmiddlefrontal_9)
4947	0.85024	(lh.lateraloccipital_6)(lh.lateraloccipital_7)(lh.lateraloccipital_8)
4948	0.85024	(lh.lateraloccipital_6)(lh.lingual_6)
4949	0.85024	(lh.lateralorbitofrontal_4)(lh.lateralorbitofrontal_6)(lh.parstriangularis_1)
4950	0.85024	(lh.lateralorbitofrontal_5)(lh.parstriangularis_1)

4951	0.85024	(lh.lateralorbitofrontal_6)(lh.rostralmiddlefrontal_1)(lh.rostralmiddlefrontal_12)(lh.rostralmiddlefrontal_9)
4952	0.85024	(lh.lateralorbitofrontal_6)(lh.rostralmiddlefrontal_10)
4953	0.85024	(lh.lateralorbitofrontal_7)(lh.parstriangularis_3)(lh.rostralmiddlefrontal_12)(lh.rostralmiddlefrontal_6)
4954	0.85024	(lh.lateralorbitofrontal_7)(lh.rostralmiddlefrontal_1)
4955	0.85024	(lh.medialorbitofrontal_2)(lh.superiorfrontal_1)(lh.superiorfrontal_3)(lh.superiorfrontal_4)
4956	0.85024	(lh.medialorbitofrontal_2)(lh.superiorfrontal_1)(lh.superiorfrontal_4)
4957	0.85024	(lh.paracentral_5)(lh.posteriorcingulate_3)(lh.precentral_4)
4958	0.85024	(lh.pericalcarine_1)(lh.pericalcarine_2)(lh.superiorparietal_12)
4959	0.85024	(lh.posteriorcingulate_2)(lh.posteriorcingulate_3)
4960	0.85024	(lh.posteriorcingulate_3)(lh.precentral_4)(lh.superiorfrontal_14)
4961	0.85024	(lh.precuneus_3)(lh.precuneus_6)(lh.superiorparietal_7)
4962	0.85024	(lh.precuneus_5)(lh.precuneus_6)(lh.precuneus_8)
4963	0.85024	(lh.rostralanteriorcingulate_1)(lh.rostralmiddlefrontal_12)(lh.rostralmiddlefrontal_9)(lh.superiorfrontal_4)
4964	0.85024	(lh.rostralanteriorcingulate_1)(lh.rostralmiddlefrontal_12)(lh.superiorfrontal_3)(lh.superiorfrontal_4)
4965	0.85024	(lh.rostralmiddlefrontal_6)(lh.rostralmiddlefrontal_7)(lh.rostralmiddlefrontal_9)
4966	0.85024	(lh.superiorfrontal_14)(lh.superiorfrontal_15)(lh.superiorfrontal_16)(lh.superiorfrontal_17)
4967	0.85024	(lh.superiorfrontal_14)(lh.superiorfrontal_15)(lh.superiorfrontal_17)(lh.superiorfrontal_18)
4968	0.85024	(lh.superiorparietal_3)(lh.superiorparietal_5)(lh.supramarginal_9)
4969	0.85024	(lh.superiortemporal_10)(lh.superiortemporal_11)
4970	0.85024	(lh.supramarginal_1)(lh.supramarginal_4)(lh.supramarginal_9)
4971	0.85024	(lh.supramarginal_2)(lh.supramarginal_9)
4972	0.85024	(lh.supramarginal_4)(lh.supramarginal_6)
4973	0.85024	(rh.bankssts_1)(rh.inferiortemporal_6)(rh.middletemporal_1)
4974	0.85024	(rh.caudalmiddlefrontal_4)(rh.caudalmiddlefrontal_5)(rh.superiorfrontal_13)
4975	0.85024	(rh.caudalmiddlefrontal_4)(rh.precentral_13)(rh.precentral_7)
4976	0.85024	(rh.fusiform_6)(rh.middletemporal_1)
4977	0.85024	(rh.inferiorparietal_10)(rh.lateraloccipital_10)(rh.middletemporal_1)
4978	0.85024	(rh.inferiorparietal_12)(rh.precuneus_1)(rh.superiorparietal_11)
4979	0.85024	(rh.inferiorparietal_9)(rh.middletemporal_1)
4980	0.85024	(rh.inferiortemporal_4)(rh.inferiortemporal_6)
4981	0.85024	(rh.insula_7)(rh.lateralorbitofrontal_6)
4982	0.85024	(rh.isthmuscingulate_1)(rh.posteriorcingulate_4)
4983	0.85024	(rh.lateraloccipital_1)(rh.pericalcarine_2)(rh.superiorparietal_11)
4984	0.85024	(rh.lateraloccipital_4)(rh.lateraloccipital_5)(rh.lateraloccipital_7)(rh.lateraloccipital_8)
4985	0.85024	(rh.lateraloccipital_5)(rh.lateraloccipital_7)(rh.lingual_2)
4986	0.85024	(rh.lateralorbitofrontal_7)(rh.rostralmiddlefrontal_11)(rh.rostralmiddlefrontal_8)
4987	0.85024	(rh.lingual_2)(rh.lingual_4)(rh.pericalcarine_1)
4988	0.85024	(rh.parstriangularis_1)(rh.rostralmiddlefrontal_6)
4989	0.85024	(rh.postcentral_4)(rh.precentral_6)
4990	0.85024	(rh.precentral_11)(rh.precentral_13)(rh.precentral_15)
4991	0.85024	(rh.precuneus_10)(rh.superiorparietal_6)
4992	0.85024	(rh.precuneus_4)(rh.precuneus_7)(rh.superiorparietal_8)
4993	0.85024	(rh.rostralmiddlefrontal_1)(rh.rostralmiddlefrontal_2)(rh.rostralmiddlefrontal_3)
4994	0.85024	(rh.rostralmiddlefrontal_10)(rh.superiorfrontal_4)
4995	0.85024	(rh.rostralmiddlefrontal_12)(rh.rostralmiddlefrontal_3)(rh.rostralmiddlefrontal_5)
4996	0.85024	(rh.rostralmiddlefrontal_3)(rh.superiorfrontal_7)
4997	0.85024	(rh.rostralmiddlefrontal_8)(rh.superiorfrontal_1)(rh.superiorfrontal_2)
4998	0.85024	(rh.superiorfrontal_13)(rh.superiorfrontal_5)(rh.superiorfrontal_8)
4999	0.85024	(Right-Caudate)(rh.caudalanteriorcingulate_2)(rh.superiorfrontal_7)
5000	0.85024	(Right-Caudate)(rh.paracentral_4)

5001	0.85024	(Right-Caudate)(rh.rostralmiddlefrontal_12)(rh.rostralmiddlefrontal_8)
5002	0.85024	(Right-Caudate)(Right-Pallidum)(Right-Putamen)(rh.posteriorcingulate_2)(rh.precentral_13)
5003	0.85024	(Right-Caudate)(Right-Putamen)(rh.caudalanteriorcingulate_1)(rh.rostralanteriorcingulate_2)
5004	0.85024	(Right-Caudate)(Right-Putamen)(rh.lateralorbitofrontal_5)(rh.lateralorbitofrontal_7)(rh.medialorbitofrontal_2)
5005	0.85024	(Right-Caudate)(Right-Thalamus-Proper)(rh.insula_1)(rh.precentral_11)
5006	0.85024	(Right-Caudate)(Right-Thalamus-Proper)(rh.isthmuscingulate_1)
5007	0.85024	(Right-Hippocampus)(rh.bankssts_2)(rh.inferiorparietal_10)(rh.inferiorparietal_4)
5008	0.85024	(Right-Hippocampus)(rh.fusiform_8)(rh.superiortemporal_10)
5009	0.85024	(Right-Hippocampus)(rh.inferiorparietal_10)(rh.inferiorparietal_9)
5010	0.85024	(Right-Hippocampus)(rh.precuneus_2)(rh.precuneus_7)
5011	0.85024	(Right-Hippocampus)(Right-Thalamus-Proper)(rh.insula_1)(rh.supramarginal_9)
5012	0.85024	(Right-Pallidum)(Right-Putamen)(rh.posteriorcingulate_2)(rh.precentral_13)
5013	0.85024	(Right-Putamen)(rh.caudalanteriorcingulate_1)(rh.rostralanteriorcingulate_2)
5014	0.85024	(Right-Putamen)(rh.insula_3)(rh.precentral_5)(rh.precentral_6)
5015	0.85024	(Right-Putamen)(rh.insula_7)(rh.lateralorbitofrontal_6)
5016	0.85024	(Right-Putamen)(rh.lateralorbitofrontal_6)(rh.medialorbitofrontal_2)(rh.rostralmiddlefrontal_13)
5017	0.85024	(Right-Putamen)(rh.medialorbitofrontal_3)
5018	0.84783	(Left-Accumbens-area)(Left-Thalamus-Proper)(Right-Caudate)
5019	0.84783	(Left-Caudate)(Left-Putamen)(lh.caudalmiddlefrontal_3)(lh.insula_6)(lh.rostralmiddlefrontal_4)
5020	0.84783	(Left-Caudate)(Left-Putamen)(lh.caudalmiddlefrontal_4)(lh.superiorfrontal_12)
5021	0.84783	(Left-Caudate)(Left-Putamen)(lh.parstriangularis_3)(lh.rostralanteriorcingulate_1)(lh.rostralmiddlefrontal_12)(lh.rostralmiddlefrontal_9)
5022	0.84783	(Left-Caudate)(Left-Putamen)(lh.rostralanteriorcingulate_1)(lh.rostralmiddlefrontal_1)(lh.rostralmiddlefrontal_9)
5023	0.84783	(Left-Caudate)(lh.caudalmiddlefrontal_3)(lh.rostralmiddlefrontal_4)(lh.superiorfrontal_11)
5024	0.84783	(Left-Caudate)(lh.insula_6)(lh.rostralanteriorcingulate_1)
5025	0.84783	(Left-Caudate)(lh.lateralorbitofrontal_4)(lh.lateralorbitofrontal_6)(lh.parstriangularis_3)
5026	0.84783	(Left-Caudate)(lh.lateralorbitofrontal_4)(lh.lateralorbitofrontal_7)(lh.parstriangularis_3)
5027	0.84783	(Left-Caudate)(lh.lateralorbitofrontal_6)(lh.parstriangularis_3)(lh.rostralmiddlefrontal_12)
5028	0.84783	(Left-Caudate)(lh.paracentral_5)(lh.precentral_4)(lh.superiorfrontal_14)
5029	0.84783	(Left-Caudate)(lh.posteriorcingulate_2)(lh.posteriorcingulate_3)
5030	0.84783	(Left-Hippocampus)(lh.fusiform_4)(lh.fusiform_5)
5031	0.84783	(Left-Hippocampus)(lh.isthmuscingulate_3)(lh.parahippocampal_3)
5032	0.84783	(Left-Pallidum)(Left-Thalamus-Proper)(lh.precentral_11)(lh.precentral_4)
5033	0.84783	(Left-Putamen)(lh.caudalmiddlefrontal_1)(lh.caudalmiddlefrontal_3)(lh.rostralmiddlefrontal_4)
5034	0.84783	(Left-Putamen)(lh.insula_3)(lh.precentral_12)
5035	0.84783	(Left-Putamen)(lh.parstriangularis_3)(lh.rostralanteriorcingulate_1)(lh.rostralmiddlefrontal_4)
5036	0.84783	(Left-Putamen)(lh.precentral_4)(lh.superiorfrontal_14)
5037	0.84783	(lh.bankssts_2)(lh.inferiortemporal_6)(lh.inferiortemporal_7)
5038	0.84783	(lh.bankssts_3)(lh.inferiortemporal_8)
5039	0.84783	(lh.caudalanteriorcingulate_2)(lh.rostralanteriorcingulate_2)(lh.superiorfrontal_11)(lh.superiorfrontal_8)
5040	0.84783	(lh.caudalmiddlefrontal_3)(lh.insula_6)(lh.rostralmiddlefrontal_1)(lh.rostralmiddlefrontal_4)
5041	0.84783	(lh.caudalmiddlefrontal_4)(lh.caudalmiddlefrontal_6)(lh.precentral_11)(lh.precentral_9)
5042	0.84783	(lh.fusiform_2)(lh.lateraloccipital_10)(lh.lateraloccipital_9)
5043	0.84783	(lh.fusiform_2)(lh.lateraloccipital_11)(lh.lateraloccipital_6)(lh.pericalcarine_3)
5044	0.84783	(lh.fusiform_2)(lh.lingual_1)(lh.pericalcarine_2)
5045	0.84783	(lh.fusiform_4)(lh.fusiform_5)(lh.fusiform_7)
5046	0.84783	(lh.fusiform_8)(lh.inferiortemporal_1)
5047	0.84783	(lh.inferiortemporal_1)(lh.inferiortemporal_3)
5048	0.84783	(lh.insula_3)(lh.precentral_9)
5049	0.84783	(lh.insula_6)(lh.rostralanteriorcingulate_2)
5050	0.84783	(lh.isthmuscingulate_2)(lh.isthmuscingulate_3)(lh.precuneus_10)(lh.precuneus_6)

5051	0.84783	(lh.lateraloccipital_1)(lh.lateraloccipital_3)(lh.pericalcarine_1)(lh.pericalcarine_2)
5052	0.84783	(lh.lateraloccipital_11)(lh.lateraloccipital_3)(lh.lingual_1)(lh.pericalcarine_2)
5053	0.84783	(lh.lateraloccipital_2)(lh.pericalcarine_1)(lh.superiorparietal_12)
5054	0.84783	(lh.lateralorbitofrontal_2)(lh.lateralorbitofrontal_7)(lh.medialorbitofrontal_5)
5055	0.84783	(lh.lateralorbitofrontal_3)(lh.lateralorbitofrontal_5)
5056	0.84783	(lh.lateralorbitofrontal_6)(lh.medialorbitofrontal_2)(lh.rostralmiddlefrontal_9)
5057	0.84783	(lh.lateralorbitofrontal_6)(lh.parstriangularis_3)(lh.rostralmiddlefrontal_1)(lh.rostralmiddlefrontal_12)(lh.rostralmiddlefrontal_9)
5058	0.84783	(lh.lateralorbitofrontal_7)(lh.parstriangularis_3)(lh.rostralmiddlefrontal_6)(lh.rostralmiddlefrontal_9)
5059	0.84783	(lh.medialorbitofrontal_2)(lh.parstriangularis_3)(lh.rostralanteriorcingulate_1)(lh.rostralmiddlefrontal_9)
5060	0.84783	(lh.paracentral_4)(lh.paracentral_5)(lh.precentral_2)(lh.precentral_4)
5061	0.84783	(lh.postcentral_14)(lh.postcentral_8)
5062	0.84783	(lh.postcentral_14)(lh.supramarginal_3)
5063	0.84783	(lh.postcentral_4)(lh.posteriorcingulate_4)
5064	0.84783	(lh.postcentral_8)(lh.precentral_16)
5065	0.84783	(lh.precentral_11)(lh.precentral_5)(lh.precentral_7)
5066	0.84783	(lh.precuneus_10)(lh.superiorparietal_6)
5067	0.84783	(lh.precuneus_9)(lh.superiorparietal_13)
5068	0.84783	(lh.rostralanteriorcingulate_2)(lh.rostralmiddlefrontal_4)(lh.superiorfrontal_10)
5069	0.84783	(lh.rostralanteriorcingulate_2)(lh.rostralmiddlefrontal_8)(lh.rostralmiddlefrontal_9)
5070	0.84783	(lh.rostralmiddlefrontal_12)(lh.rostralmiddlefrontal_9)(lh.superiorfrontal_3)(lh.superiorfrontal_4)
5071	0.84783	(lh.rostralmiddlefrontal_4)(lh.rostralmiddlefrontal_7)(lh.rostralmiddlefrontal_9)
5072	0.84783	(lh.rostralmiddlefrontal_4)(lh.rostralmiddlefrontal_8)(lh.rostralmiddlefrontal_9)(lh.superiorfrontal_4)
5073	0.84783	(lh.rostralmiddlefrontal_8)(lh.superiorfrontal_1)
5074	0.84783	(lh.superiorparietal_12)(lh.superiorparietal_14)
5075	0.84783	(lh.supramarginal_10)(lh.supramarginal_7)
5076	0.84783	(rh.bankssts_1)(rh.inferiortemporal_6)(rh.inferiortemporal_7)
5077	0.84783	(rh.fusiform_1)(rh.lateraloccipital_5)(rh.lateraloccipital_7)
5078	0.84783	(rh.fusiform_1)(rh.lingual_2)(rh.pericalcarine_3)
5079	0.84783	(rh.fusiform_2)(rh.fusiform_3)(rh.lateraloccipital_8)
5080	0.84783	(rh.fusiform_5)(rh.inferiorparietal_10)(rh.lateraloccipital_10)
5081	0.84783	(rh.inferiorparietal_1)(rh.inferiorparietal_4)(rh.superiorparietal_1)
5082	0.84783	(rh.insula_1)(rh.superiorparietal_1)(rh.supramarginal_1)(rh.supramarginal_8)
5083	0.84783	(rh.insula_5)(rh.temporalpole_1)
5084	0.84783	(rh.lateraloccipital_4)(rh.lateraloccipital_8)(rh.pericalcarine_3)
5085	0.84783	(rh.lateraloccipital_8)(rh.lingual_7)(rh.pericalcarine_1)
5086	0.84783	(rh.lateralorbitofrontal_2)(rh.lateralorbitofrontal_4)(rh.lateralorbitofrontal_6)(rh.lateralorbitofrontal_7)
5087	0.84783	(rh.lateralorbitofrontal_6)(rh.parstriangularis_1)(rh.parstriangularis_3)(rh.parstriangularis_4)
5088	0.84783	(rh.medialorbitofrontal_2)(rh.rostralmiddlefrontal_12)(rh.rostralmiddlefrontal_13)
5089	0.84783	(rh.paracentral_4)(rh.precentral_13)(rh.superiorfrontal_16)
5090	0.84783	(rh.paracentral_4)(rh.superiorfrontal_14)(rh.superiorfrontal_16)
5091	0.84783	(rh.parstriangularis_1)(rh.parstriangularis_3)(rh.rostralmiddlefrontal_12)
5092	0.84783	(rh.postcentral_1)(rh.precentral_3)
5093	0.84783	(rh.postcentral_3)(rh.precentral_1)
5094	0.84783	(rh.precentral_13)(rh.precentral_7)(rh.precentral_8)
5095	0.84783	(rh.precuneus_1)(rh.precuneus_4)(rh.superiorparietal_10)(rh.superiorparietal_11)
5096	0.84783	(rh.rostralmiddlefrontal_9)(rh.superiorfrontal_2)(rh.superiorfrontal_5)(rh.superiorfrontal_7)
5097	0.84783	(rh.superiorfrontal_10)(rh.superiorfrontal_13)(rh.superiorfrontal_8)
5098	0.84783	(Right-Caudate)(rh.caudalanteriorcingulate_2)(rh.superiorfrontal_11)
5099	0.84783	(Right-Caudate)(rh.caudalmiddlefrontal_2)
5100	0.84783	(Right-Caudate)(rh.caudalmiddlefrontal_4)(rh.insula_7)(rh.parsopercularis_4)

5101	0.84783	(Right-Caudate)(rh.parstriangularis_1)(rh.rostralmiddlefrontal_3)
5102	0.84783	(Right-Caudate)(rh.superiorfrontal_11)(rh.superiorfrontal_13)(rh.superiorfrontal_9)
5103	0.84783	(Right-Caudate)(rh.superiorfrontal_13)(rh.superiorfrontal_7)
5104	0.84783	(Right-Caudate)(Right-Putamen)(rh.caudalmiddlefrontal_4)(rh.insula_7)(rh.parsopercularis_4)
5105	0.84783	(Right-Caudate)(Right-Putamen)(rh.medialorbitofrontal_3)
5106	0.84783	(Right-Caudate)(Right-Putamen)(rh.rostralmiddlefrontal_12)(rh.rostralmiddlefrontal_8)
5107	0.84783	(Right-Caudate)(Right-Putamen)(Right-Thalamus-Proper)(rh.insula_1)(rh.precentral_11)
5108	0.84783	(Right-Hippocampus)(rh.isthmuscingulate_2)(rh.precuneus_3)(rh.precuneus_4)
5109	0.84783	(Right-Hippocampus)(Right-Putamen)(Right-Thalamus-Proper)(rh.insula_1)(rh.supramarginal_9)
5110	0.84783	(Right-Putamen)(rh.caudalmiddlefrontal_4)(rh.precentral_13)(rh.precentral_7)
5111	0.84783	(Right-Putamen)(rh.insula_3)(rh.postcentral_1)
5112	0.84783	(Right-Putamen)(rh.insula_7)(rh.parsopercularis_4)(rh.parstriangularis_1)
5113	0.84783	(Right-Putamen)(rh.insula_7)(rh.rostralmiddlefrontal_5)
5114	0.84783	(Right-Putamen)(rh.precentral_11)(rh.precentral_12)
5115	0.84783	(Right-Putamen)(rh.precentral_13)(rh.superiorfrontal_14)
5116	0.84541	(Left-Accumbens-area)(Left-Caudate)(Left-Hippocampus)(Left-Thalamus-Proper)
5117	0.84541	(Left-Accumbens-area)(Left-Caudate)(Left-Hippocampus)
5118	0.84541	(Left-Caudate)(Left-Pallidum)(Left-Putamen)(Left-Thalamus-Proper)(lh.posteriorcingulate_3)
5119	0.84541	(Left-Caudate)(Left-Pallidum)(Left-Thalamus-Proper)(lh.precentral_11)(lh.precentral_4)
5120	0.84541	(Left-Caudate)(Left-Putamen)(Left-Thalamus-Proper)(lh.insula_1)(lh.precentral_11)(lh.precentral_7)
5121	0.84541	(Left-Caudate)(Left-Putamen)(Left-Thalamus-Proper)(lh.supramarginal_1)
5122	0.84541	(Left-Caudate)(Left-Putamen)(lh.caudalanteriorcingulate_2)(lh.rostralanteriorcingulate_2)(lh.superiorfrontal_11)
5123	0.84541	(Left-Caudate)(Left-Putamen)(lh.insula_6)(lh.rostralanteriorcingulate_1)
5124	0.84541	(Left-Caudate)(Left-Putamen)(lh.insula_6)(lh.rostralmiddlefrontal_1)(lh.rostralmiddlefrontal_9)
5125	0.84541	(Left-Caudate)(Left-Putamen)(lh.medialorbitofrontal_2)(lh.rostralanteriorcingulate_1)(lh.superiorfrontal_4)
5126	0.84541	(Left-Caudate)(Left-Putamen)(lh.precentral_7)(lh.precentral_9)
5127	0.84541	(Left-Caudate)(Left-Putamen)(lh.superiorfrontal_10)(lh.superiorfrontal_4)
5128	0.84541	(Left-Caudate)(Left-Thalamus-Proper)(lh.insula_1)(lh.precentral_11)(lh.precentral_7)
5129	0.84541	(Left-Caudate)(Left-Thalamus-Proper)(lh.insula_1)(lh.supramarginal_1)
5130	0.84541	(Left-Caudate)(lh.caudalanteriorcingulate_2)(lh.caudalmiddlefrontal_3)(lh.caudalmiddlefrontal_5)
5131	0.84541	(Left-Caudate)(lh.insula_6)(lh.parstriangularis_3)(lh.rostralmiddlefrontal_4)
5132	0.84541	(Left-Caudate)(lh.lateralorbitofrontal_4)(lh.lateralorbitofrontal_6)(lh.rostralmiddlefrontal_9)
5133	0.84541	(Left-Caudate)(lh.lateralorbitofrontal_4)(lh.rostralmiddlefrontal_12)(lh.rostralmiddlefrontal_9)
5134	0.84541	(Left-Caudate)(lh.medialorbitofrontal_2)(lh.rostralmiddlefrontal_9)(lh.superiorfrontal_3)
5135	0.84541	(Left-Caudate)(lh.parstriangularis_3)(lh.rostralanteriorcingulate_1)(lh.rostralmiddlefrontal_1)
5136	0.84541	(Left-Caudate)(lh.parstriangularis_3)(lh.rostralanteriorcingulate_1)(lh.rostralmiddlefrontal_4)
5137	0.84541	(Left-Caudate)(lh.precentral_11)(lh.precentral_7)(lh.precentral_9)
5138	0.84541	(Left-Caudate)(lh.rostralmiddlefrontal_1)(lh.rostralmiddlefrontal_12)(lh.rostralmiddlefrontal_9)
5139	0.84541	(Left-Pallidum)(Left-Putamen)(Left-Thalamus-Proper)(lh.posteriorcingulate_3)
5140	0.84541	(Left-Pallidum)(Left-Putamen)(Left-Thalamus-Proper)(lh.precentral_11)(lh.precentral_4)
5141	0.84541	(Left-Pallidum)(Left-Putamen)(Left-Thalamus-Proper)(lh.precentral_7)
5142	0.84541	(Left-Pallidum)(Left-Thalamus-Proper)(lh.precentral_7)
5143	0.84541	(Left-Putamen)(Left-Thalamus-Proper)(lh.postcentral_8)(lh.precentral_4)
5144	0.84541	(Left-Putamen)(lh.caudalmiddlefrontal_1)(lh.rostralmiddlefrontal_1)
5145	0.84541	(Left-Putamen)(lh.caudalmiddlefrontal_3)(lh.rostralmiddlefrontal_1)(lh.rostralmiddlefrontal_9)
5146	0.84541	(Left-Putamen)(lh.insula_3)(lh.precentral_9)
5147	0.84541	(Left-Putamen)(lh.insula_6)(lh.lateralorbitofrontal_6)(lh.parstriangularis_3)
5148	0.84541	(Left-Putamen)(lh.insula_6)(lh.parstriangularis_3)(lh.rostralmiddlefrontal_1)(lh.rostralmiddlefrontal_9)
5149	0.84541	(Left-Putamen)(lh.parstriangularis_3)(lh.rostralanteriorcingulate_1)(lh.rostralmiddlefrontal_1)
5150	0.84541	(Left-Putamen)(lh.parstriangularis_3)(lh.rostralmiddlefrontal_1)(lh.rostralmiddlefrontal_12)

5151	0.84541	(Left-Putamen)(lh.precentral_11)(lh.precentral_13)
5152	0.84541	(Left-Putamen)(lh.rostralmiddlefrontal_12)(lh.superiorfrontal_4)
5153	0.84541	(Left-Putamen)(lh.rostralmiddlefrontal_8)(lh.superiorfrontal_3)
5154	0.84541	(lh.bankssts_2)(lh.inferiortemporal_3)(lh.inferiortemporal_6)
5155	0.84541	(lh.bankssts_3)(lh.inferiorparietal_5)(lh.superiortemporal_2)
5156	0.84541	(lh.caudalanteriorcingulate_1)(lh.caudalanteriorcingulate_2)(lh.superiorfrontal_10)
5157	0.84541	(lh.caudalanteriorcingulate_2)(lh.rostralanteriorcingulate_1)(lh.rostralanteriorcingulate_2)(lh.superiorfrontal_4)
5158	0.84541	(lh.caudalanteriorcingulate_2)(lh.superiorfrontal_14)(lh.superiorfrontal_4)(lh.superiorfrontal_8)
5159	0.84541	(lh.caudalmiddlefrontal_3)(lh.parsopercularis_3)(lh.rostralmiddlefrontal_1)
5160	0.84541	(lh.fusiform_1)(lh.lateraloccipital_10)
5161	0.84541	(lh.fusiform_2)(lh.lateraloccipital_6)(lh.lingual_8)(lh.pericalcarine_3)
5162	0.84541	(lh.inferiorparietal_1)(lh.lateraloccipital_6)(lh.lateraloccipital_7)(lh.pericalcarine_3)
5163	0.84541	(lh.insula_1)(lh.postcentral_8)(lh.supramarginal_4)
5164	0.84541	(lh.insula_6)(lh.lateralorbitofrontal_6)(lh.rostralmiddlefrontal_1)
5165	0.84541	(lh.lateraloccipital_2)(lh.lateraloccipital_3)(lh.lateraloccipital_6)(lh.pericalcarine_2)
5166	0.84541	(lh.lateraloccipital_7)(lh.lingual_1)(lh.pericalcarine_3)
5167	0.84541	(lh.lateralorbitofrontal_2)(lh.medialorbitofrontal_4)
5168	0.84541	(lh.lateralorbitofrontal_4)(lh.rostralanteriorcingulate_1)(lh.rostralmiddlefrontal_12)(lh.rostralmiddlefrontal_9)
5169	0.84541	(lh.lateralorbitofrontal_6)(lh.lateralorbitofrontal_7)(lh.medialorbitofrontal_2)
5170	0.84541	(lh.medialorbitofrontal_2)(lh.medialorbitofrontal_4)(lh.rostralanteriorcingulate_1)
5171	0.84541	(lh.medialorbitofrontal_2)(lh.rostralmiddlefrontal_12)(lh.superiorfrontal_4)
5172	0.84541	(lh.parsorbitalis_1)(lh.rostralmiddlefrontal_9)
5173	0.84541	(lh.precentral_4)(lh.precentral_5)
5174	0.84541	(lh.precuneus_10)(lh.precuneus_11)(lh.precuneus_9)(lh.superiorparietal_11)
5175	0.84541	(lh.precuneus_10)(lh.precuneus_8)(lh.precuneus_9)(lh.superiorparietal_11)
5176	0.84541	(lh.rostralanteriorcingulate_1)(lh.rostralmiddlefrontal_8)(lh.rostralmiddlefrontal_9)
5177	0.84541	(lh.rostralanteriorcingulate_2)(lh.superiorfrontal_10)(lh.superiorfrontal_11)(lh.superiorfrontal_4)
5178	0.84541	(lh.rostralmiddlefrontal_1)(lh.rostralmiddlefrontal_5)(lh.rostralmiddlefrontal_9)
5179	0.84541	(lh.rostralmiddlefrontal_9)(lh.superiorfrontal_2)(lh.superiorfrontal_3)(lh.superiorfrontal_4)
5180	0.84541	(lh.superiorfrontal_10)(lh.superiorfrontal_14)(lh.superiorfrontal_7)
5181	0.84541	(lh.superiorfrontal_11)(lh.superiorfrontal_3)(lh.superiorfrontal_4)
5182	0.84541	(lh.superiorfrontal_11)(lh.superiorfrontal_3)
5183	0.84541	(lh.superiorfrontal_12)(lh.superiorfrontal_13)
5184	0.84541	(rh.bankssts_2)(rh.inferiorparietal_4)(rh.insula_2)
5185	0.84541	(rh.bankssts_2)(rh.inferiortemporal_2)
5186	0.84541	(rh.caudalanteriorcingulate_3)(rh.posteriorcingulate_1)(rh.posteriorcingulate_2)
5187	0.84541	(rh.fusiform_1)(rh.fusiform_2)(rh.lateraloccipital_8)(rh.lingual_7)
5188	0.84541	(rh.fusiform_1)(rh.lateraloccipital_4)(rh.lateraloccipital_7)(rh.lateraloccipital_8)(rh.lingual_2)
5189	0.84541	(rh.fusiform_1)(rh.lingual_4)(rh.pericalcarine_1)
5190	0.84541	(rh.fusiform_2)(rh.inferiorparietal_10)(rh.lateraloccipital_10)(rh.lateraloccipital_8)
5191	0.84541	(rh.fusiform_2)(rh.inferiorparietal_10)(rh.lingual_7)
5192	0.84541	(rh.fusiform_3)(rh.inferiorparietal_10)(rh.lateraloccipital_10)
5193	0.84541	(rh.fusiform_8)(rh.insula_5)(rh.superiortemporal_10)
5194	0.84541	(rh.inferiorparietal_12)(rh.lateraloccipital_8)(rh.lingual_7)
5195	0.84541	(rh.inferiorparietal_4)(rh.inferiorparietal_9)
5196	0.84541	(rh.inferiortemporal_2)(rh.superiortemporal_3)(rh.superiortemporal_6)
5197	0.84541	(rh.lateraloccipital_1)(rh.lateraloccipital_4)(rh.pericalcarine_4)
5198	0.84541	(rh.lateraloccipital_3)(rh.pericalcarine_3)(rh.superiorparietal_11)
5199	0.84541	(rh.lateraloccipital_7)(rh.lingual_1)(rh.lingual_2)
5200	0.84541	(rh.lateralorbitofrontal_6)(rh.parstriangularis_1)(rh.parstriangularis_2)

5201	0.84541	(rh.lateralorbitofrontal_6)(rh.parstriangularis_3)(rh.rostralmiddlefrontal_12)
5202	0.84541	(rh.lateralorbitofrontal_7)(rh.parstriangularis_4)(rh.rostralmiddlefrontal_12)
5203	0.84541	(rh.lingual_2)(rh.lingual_5)
5204	0.84541	(rh.middletemporal_4)(rh.middletemporal_8)
5205	0.84541	(rh.paracentral_4)(rh.precentral_13)(rh.precentral_14)(rh.precentral_15)
5206	0.84541	(rh.paracentral_6)(rh.supramarginal_8)
5207	0.84541	(rh.parstriangularis_1)(rh.parstriangularis_2)(rh.rostralmiddlefrontal_8)
5208	0.84541	(rh.parstriangularis_4)(rh.rostralmiddlefrontal_12)(rh.rostralmiddlefrontal_3)(rh.rostralmiddlefrontal_8)
5209	0.84541	(rh.parstriangularis_4)(rh.rostralmiddlefrontal_7)
5210	0.84541	(rh.precentral_10)(rh.precentral_9)
5211	0.84541	(rh.precuneus_10)(rh.superiorparietal_1)(rh.superiorparietal_8)
5212	0.84541	(rh.superiorfrontal_11)(rh.superiorfrontal_13)(rh.superiorfrontal_7)(rh.superiorfrontal_9)
5213	0.84541	(rh.superiorfrontal_12)(rh.superiorfrontal_13)(rh.superiorfrontal_16)
5214	0.84541	(rh.superiorfrontal_2)(rh.superiorfrontal_4)(rh.superiorfrontal_5)(rh.superiorfrontal_6)(rh.superiorfrontal_7)
5215	0.84541	(rh.superiortemporal_2)(rh.superiortemporal_3)
5216	0.84541	(Right-Caudate)(rh.caudalanteriorcingulate_3)(rh.posteriorcingulate_1)(rh.posteriorcingulate_2)
5217	0.84541	(Right-Caudate)(rh.lateralorbitofrontal_7)(rh.parstriangularis_1)
5218	0.84541	(Right-Caudate)(Right-Putamen)(rh.caudalmiddlefrontal_4)(rh.rostralmiddlefrontal_1)
5219	0.84541	(Right-Caudate)(Right-Putamen)(rh.precentral_5)(rh.precentral_6)
5220	0.84541	(Right-Hippocampus)(rh.inferiorparietal_4)(rh.superiortemporal_1)
5221	0.84541	(Right-Hippocampus)(rh.superiorparietal_1)(rh.supramarginal_9)
5222	0.84541	(Right-Hippocampus)(Right-Thalamus-Proper)(rh.isthmuscingulate_1)
5223	0.84541	(Right-Pallidum)(rh.insula_1)
5224	0.84541	(Right-Pallidum)(Right-Putamen)(rh.insula_1)
5225	0.84541	(Right-Putamen)(rh.lateralorbitofrontal_2)(rh.lateralorbitofrontal_4)(rh.lateralorbitofrontal_6)(rh.lateralorbitofrontal_7)
5226	0.84541	(Right-Putamen)(rh.lateralorbitofrontal_6)(rh.parstriangularis_1)(rh.rostralmiddlefrontal_12)(rh.rostralmiddlefrontal_8)
5227	0.84541	(Right-Putamen)(rh.lateralorbitofrontal_7)(rh.rostralmiddlefrontal_8)(rh.superiorfrontal_2)
5228	0.84541	(Right-Putamen)(rh.paracentral_6)
5229	0.84541	(Right-Putamen)(rh.parsopercularis_2)(rh.parstriangularis_1)
5230	0.84541	(Right-Putamen)(rh.parstriangularis_1)(rh.rostralmiddlefrontal_12)(rh.rostralmiddlefrontal_3)(rh.rostralmiddlefrontal_8)
5231	0.84541	(Right-Putamen)(rh.postcentral_5)(rh.supramarginal_1)
5232	0.84541	(Right-Putamen)(rh.precentral_6)(rh.precentral_8)
5233	0.84541	(Right-Putamen)(rh.rostralmiddlefrontal_12)(rh.rostralmiddlefrontal_13)
5234	0.84541	(Right-Putamen)(rh.rostralmiddlefrontal_8)(rh.superiorfrontal_7)
5235	0.84541	(Right-Putamen)(rh.superiorfrontal_9)
5236	0.843	(Left-Amygdala)(Left-Pallidum)(Left-Putamen)(lh.insula_7)
5237	0.843	(Left-Amygdala)(Left-Pallidum)(lh.insula_7)
5238	0.843	(Left-Caudate)(Left-Pallidum)(Left-Putamen)(Left-Thalamus-Proper)(lh.precentral_11)(lh.precentral_4)
5239	0.843	(Left-Caudate)(Left-Pallidum)(Left-Putamen)(Left-Thalamus-Proper)(lh.precentral_9)
5240	0.843	(Left-Caudate)(Left-Pallidum)(Left-Thalamus-Proper)(lh.precentral_9)
5241	0.843	(Left-Caudate)(Left-Putamen)(lh.lateralorbitofrontal_4)(lh.rostralmiddlefrontal_12)(lh.rostralmiddlefrontal_9)
5242	0.843	(Left-Caudate)(Left-Putamen)(lh.lateralorbitofrontal_7)(lh.medialorbitofrontal_2)(lh.medialorbitofrontal_4)
5243	0.843	(Left-Caudate)(Left-Putamen)(lh.medialorbitofrontal_2)(lh.parstriangularis_3)
5244	0.843	(Left-Caudate)(Left-Putamen)(lh.rostralmiddlefrontal_4)(lh.superiorfrontal_10)
5245	0.843	(Left-Caudate)(Left-Thalamus-Proper)(Right-Caudate)(Right-Thalamus-Proper)
5246	0.843	(Left-Caudate)(lh.caudalanteriorcingulate_2)(lh.superiorfrontal_10)(lh.superiorfrontal_11)(lh.superiorfrontal_8)
5247	0.843	(Left-Caudate)(lh.insula_6)(lh.parsopercularis_1)
5248	0.843	(Left-Caudate)(lh.medialorbitofrontal_2)(lh.medialorbitofrontal_4)(lh.rostralanteriorcingulate_1)
5249	0.843	(Left-Caudate)(lh.medialorbitofrontal_2)(lh.rostralanteriorcingulate_1)(lh.superiorfrontal_3)
5250	0.843	(Left-Caudate)(lh.parsopercularis_1)(lh.parstriangularis_3)

5251	0.843	(Left-Caudate)(lh.postcentral_8)(lh.precentral_4)(lh.precentral_7)
5252	0.843	(Left-Caudate)(lh.posteriorcingulate_3)(lh.posteriorcingulate_4)
5253	0.843	(Left-Caudate)(lh.rostralmiddlefrontal_1)(lh.rostralmiddlefrontal_3)
5254	0.843	(Left-Hippocampus)(Left-Putamen)(lh.supramarginal_1)
5255	0.843	(Left-Hippocampus)(lh.fusiform_5)(lh.inferiorparietal_5)
5256	0.843	(Left-Hippocampus)(lh.inferiorparietal_5)(lh.insula_2)
5257	0.843	(Left-Putamen)(Left-Thalamus-Proper)(lh.superiorparietal_3)
5258	0.843	(Left-Putamen)(lh.lateralorbitofrontal_4)(lh.rostralanteriorcingulate_1)(lh.rostralmiddlefrontal_12)(lh.rostralmiddlefrontal_9)
5259	0.843	(Left-Putamen)(lh.medialorbitofrontal_2)(lh.parstriangularis_3)(lh.rostralmiddlefrontal_12)(lh.rostralmiddlefrontal_9)
5260	0.843	(Left-Putamen)(lh.medialorbitofrontal_5)(lh.rostralanteriorcingulate_1)
5261	0.843	(Left-Putamen)(lh.parstriangularis_3)(lh.rostralanteriorcingulate_1)(lh.rostralmiddlefrontal_1)(lh.rostralmiddlefrontal_9)
5262	0.843	(Left-Putamen)(lh.parstriangularis_3)(lh.rostralanteriorcingulate_1)(lh.rostralmiddlefrontal_4)(lh.rostralmiddlefrontal_9)
5263	0.843	(Left-Putamen)(lh.parstriangularis_3)(lh.rostralmiddlefrontal_1)(lh.rostralmiddlefrontal_12)(lh.rostralmiddlefrontal_9)
5264	0.843	(Left-Putamen)(lh.postcentral_8)(lh.precentral_11)(lh.precentral_14)
5265	0.843	(Left-Putamen)(lh.rostralanteriorcingulate_2)(lh.rostralmiddlefrontal_1)
5266	0.843	(Left-Putamen)(lh.superiorparietal_5)
5267	0.843	(lh.bankssts_2)(lh.fusiform_7)(lh.inferiortemporal_3)
5268	0.843	(lh.caudalanteriorcingulate_1)(lh.rostralanteriorcingulate_2)(lh.rostralmiddlefrontal_4)
5269	0.843	(lh.caudalmiddlefrontal_4)(lh.caudalmiddlefrontal_6)(lh.superiorfrontal_12)
5270	0.843	(lh.entorhinal_1)(lh.insula_4)
5271	0.843	(lh.fusiform_1)(lh.lateraloccipital_6)
5272	0.843	(lh.fusiform_2)(lh.lateraloccipital_6)(lh.lingual_4)(lh.pericalcarine_3)
5273	0.843	(lh.fusiform_6)(lh.inferiortemporal_7)
5274	0.843	(lh.insula_1)(lh.postcentral_14)(lh.supramarginal_1)(lh.supramarginal_2)
5275	0.843	(lh.insula_3)(lh.precentral_14)
5276	0.843	(lh.isthmuscingulate_2)(lh.precuneus_11)(lh.precuneus_6)
5277	0.843	(lh.lateraloccipital_1)(lh.lateraloccipital_2)(lh.pericalcarine_1)(lh.pericalcarine_2)
5278	0.843	(lh.lateraloccipital_11)(lh.lingual_1)(lh.pericalcarine_2)(lh.pericalcarine_3)
5279	0.843	(lh.lateraloccipital_2)(lh.lateraloccipital_3)(lh.lateraloccipital_6)(lh.pericalcarine_3)
5280	0.843	(lh.lateralorbitofrontal_6)(lh.lateralorbitofrontal_7)(lh.parstriangularis_3)(lh.rostralmiddlefrontal_12)(lh.rostralmiddlefrontal_6)
5281	0.843	(lh.lateralorbitofrontal_6)(lh.medialorbitofrontal_2)(lh.rostralmiddlefrontal_12)
5282	0.843	(lh.lateralorbitofrontal_6)(lh.parstriangularis_2)(lh.rostralmiddlefrontal_1)
5283	0.843	(lh.lateralorbitofrontal_7)(lh.medialorbitofrontal_4)(lh.rostralanteriorcingulate_1)
5284	0.843	(lh.lateralorbitofrontal_7)(lh.parstriangularis_3)(lh.rostralmiddlefrontal_1)
5285	0.843	(lh.lateralorbitofrontal_7)(lh.rostralmiddlefrontal_9)(lh.superiorfrontal_3)
5286	0.843	(lh.lingual_8)(lh.precuneus_11)
5287	0.843	(lh.paracentral_5)(lh.superiorfrontal_12)(lh.superiorfrontal_14)(lh.superiorfrontal_15)
5288	0.843	(lh.parsopercularis_2)(lh.parsopercularis_3)(lh.parstriangularis_3)
5289	0.843	(lh.parstriangularis_2)(lh.rostralmiddlefrontal_1)(lh.rostralmiddlefrontal_9)
5290	0.843	(lh.parstriangularis_3)(lh.rostralanteriorcingulate_2)(lh.rostralmiddlefrontal_4)
5291	0.843	(lh.parstriangularis_3)(lh.rostralanteriorcingulate_2)(lh.rostralmiddlefrontal_9)
5292	0.843	(lh.postcentral_14)(lh.supramarginal_1)(lh.supramarginal_4)
5293	0.843	(lh.postcentral_3)(lh.postcentral_6)
5294	0.843	(lh.postcentral_5)(lh.postcentral_6)
5295	0.843	(lh.precentral_5)(lh.precentral_6)
5296	0.843	(lh.precuneus_11)(lh.precuneus_6)(lh.superiorparietal_11)
5297	0.843	(lh.precuneus_6)(lh.precuneus_8)(lh.superiorparietal_9)
5298	0.843	(lh.precuneus_8)(lh.superiorparietal_11)(lh.superiorparietal_6)
5299	0.843	(lh.rostralanteriorcingulate_1)(lh.superiorfrontal_1)
5300	0.843	(lh.rostralanteriorcingulate_1)(lh.superiorfrontal_2)

5301	0.843	(lh.rostralmiddlefrontal_11)(lh.rostralmiddlefrontal_6)
5302	0.843	(lh.rostralmiddlefrontal_4)(lh.rostralmiddlefrontal_8)(lh.superiorfrontal_3)(lh.superiorfrontal_4)
5303	0.843	(lh.rostralmiddlefrontal_4)(lh.rostralmiddlefrontal_9)(lh.superiorfrontal_3)(lh.superiorfrontal_4)
5304	0.843	(lh.rostralmiddlefrontal_8)(lh.superiorfrontal_1)(lh.superiorfrontal_3)
5305	0.843	(lh.superiorfrontal_10)(lh.superiorfrontal_14)(lh.superiorfrontal_7)(lh.superiorfrontal_8)
5306	0.843	(lh.superiorfrontal_10)(lh.superiorfrontal_4)(lh.superiorfrontal_7)(lh.superiorfrontal_9)
5307	0.843	(lh.superiorfrontal_10)(lh.superiorfrontal_4)(lh.superiorfrontal_9)
5308	0.843	(lh.superiorparietal_5)(lh.supramarginal_1)(lh.supramarginal_6)
5309	0.843	(lh.superiortemporal_6)(lh.superiortemporal_9)
5310	0.843	(rh.caudalmiddlefrontal_4)(rh.parsopercularis_2)
5311	0.843	(rh.caudalmiddlefrontal_5)(rh.superiorfrontal_11)
5312	0.843	(rh.cuneus_2)(rh.pericalcarine_2)(rh.superiorparietal_11)
5313	0.843	(rh.fusiform_1)(rh.fusiform_2)(rh.inferiorparietal_10)(rh.lateraloccipital_10)
5314	0.843	(rh.fusiform_1)(rh.inferiorparietal_10)(rh.lingual_7)
5315	0.843	(rh.fusiform_2)(rh.fusiform_3)(rh.lateraloccipital_10)(rh.lateraloccipital_8)
5316	0.843	(rh.fusiform_2)(rh.fusiform_5)
5317	0.843	(rh.inferiorparietal_10)(rh.inferiorparietal_6)
5318	0.843	(rh.inferiorparietal_10)(rh.lingual_5)(rh.lingual_7)
5319	0.843	(rh.inferiortemporal_5)(rh.middletemporal_1)
5320	0.843	(rh.insula_3)(rh.precentral_5)(rh.precentral_7)
5321	0.843	(rh.lateraloccipital_1)(rh.lateraloccipital_3)(rh.pericalcarine_3)
5322	0.843	(rh.lateraloccipital_3)(rh.superiorparietal_13)
5323	0.843	(rh.lateralorbitofrontal_6)(rh.lateralorbitofrontal_7)(rh.parstriangularis_4)
5324	0.843	(rh.lateralorbitofrontal_7)(rh.medialorbitofrontal_2)(rh.rostralmiddlefrontal_11)(rh.rostralmiddlefrontal_12)
5325	0.843	(rh.lingual_2)(rh.pericalcarine_1)(rh.pericalcarine_3)
5326	0.843	(rh.middletemporal_5)(rh.middletemporal_6)
5327	0.843	(rh.middletemporal_5)(rh.middletemporal_8)
5328	0.843	(rh.middletemporal_8)(rh.middletemporal_9)
5329	0.843	(rh.paracentral_3)(rh.precentral_15)(rh.precentral_16)
5330	0.843	(rh.paracentral_3)(rh.superiorfrontal_17)
5331	0.843	(rh.paracentral_4)(rh.precentral_16)
5332	0.843	(rh.paracentral_6)(rh.superiorparietal_7)
5333	0.843	(rh.pericalcarine_2)(rh.superiorparietal_11)(rh.superiorparietal_13)
5334	0.843	(rh.postcentral_1)(rh.postcentral_2)(rh.postcentral_3)
5335	0.843	(rh.posteriorcingulate_4)(rh.supramarginal_9)
5336	0.843	(rh.precentral_5)(rh.precentral_6)(rh.precentral_7)
5337	0.843	(rh.precuneus_1)(rh.superiorparietal_13)
5338	0.843	(rh.precuneus_7)(rh.superiorparietal_1)(rh.superiorparietal_10)
5339	0.843	(rh.rostralmiddlefrontal_12)(rh.rostralmiddlefrontal_3)(rh.rostralmiddlefrontal_5)(rh.rostralmiddlefrontal_8)
5340	0.843	(rh.rostralmiddlefrontal_9)(rh.superiorfrontal_4)
5341	0.843	(rh.rostralmiddlefrontal_9)(rh.superiorfrontal_6)
5342	0.843	(rh.superiorfrontal_10)(rh.superiorfrontal_11)(rh.superiorfrontal_13)(rh.superiorfrontal_8)
5343	0.843	(rh.superiorfrontal_11)(rh.superiorfrontal_13)(rh.superiorfrontal_5)(rh.superiorfrontal_9)
5344	0.843	(rh.superiorfrontal_12)(rh.superiorfrontal_13)(rh.superiorfrontal_15)
5345	0.843	(rh.superiorfrontal_2)(rh.superiorfrontal_3)(rh.superiorfrontal_4)(rh.superiorfrontal_5)
5346	0.843	(rh.superiorfrontal_4)(rh.superiorfrontal_8)
5347	0.843	(Right-Accumbens-area)(Right-Amygdala)(Right-Caudate)(Right-Pallidum)(Right-Thalamus-Proper)
5348	0.843	(Right-Accumbens-area)(Right-Amygdala)(Right-Caudate)(Right-Pallidum)
5349	0.843	(Right-Accumbens-area)(Right-Amygdala)(Right-Caudate)(Right-Thalamus-Proper)
5350	0.843	(Right-Accumbens-area)(Right-Amygdala)(Right-Caudate)

5351	0.843	(Right-Caudate)(rh.insula_1)(rh.supramarginal_8)
5352	0.843	(Right-Caudate)(rh.insula_7)(rh.parstriangularis_1)
5353	0.843	(Right-Caudate)(rh.lateralorbitofrontal_4)(rh.lateralorbitofrontal_5)(rh.lateralorbitofrontal_7)
5354	0.843	(Right-Caudate)(rh.lateralorbitofrontal_6)(rh.rostralmiddlefrontal_12)
5355	0.843	(Right-Caudate)(rh.rostralmiddlefrontal_1)(rh.superiorfrontal_7)
5356	0.843	(Right-Caudate)(rh.rostralmiddlefrontal_3)(rh.rostralmiddlefrontal_5)
5357	0.843	(Right-Caudate)(Right-Putamen)(rh.lateralorbitofrontal_6)(rh.rostralmiddlefrontal_12)
5358	0.843	(Right-Hippocampus)(rh.inferiorparietal_1)
5359	0.843	(Right-Hippocampus)(rh.inferiorparietal_10)(rh.lingual_5)
5360	0.843	(Right-Hippocampus)(rh.inferiorparietal_4)(rh.supramarginal_8)
5361	0.843	(Right-Hippocampus)(rh.superiortemporal_7)
5362	0.843	(Right-Putamen)(rh.caudalmiddlefrontal_4)(rh.insula_7)(rh.rostralmiddlefrontal_3)
5363	0.843	(Right-Putamen)(rh.insula_1)(rh.supramarginal_1)(rh.supramarginal_8)
5364	0.843	(Right-Putamen)(rh.insula_3)(rh.precentral_5)(rh.precentral_7)
5365	0.843	(Right-Putamen)(rh.insula_7)(rh.parstriangularis_1)(rh.rostralmiddlefrontal_3)
5366	0.843	(Right-Putamen)(rh.lateralorbitofrontal_6)(rh.lateralorbitofrontal_7)(rh.medialorbitofrontal_2)(rh.rostralmiddlefrontal_13)
5367	0.843	(Right-Putamen)(rh.rostralanteriorcingulate_2)(rh.rostralmiddlefrontal_8)
5368	0.843	(Right-Putamen)(rh.rostralmiddlefrontal_1)(rh.rostralmiddlefrontal_8)
5369	0.843	(Right-Putamen)(rh.rostralmiddlefrontal_10)(rh.rostralmiddlefrontal_8)(rh.superiorfrontal_2)
5370	0.843	(Right-Putamen)(rh.rostralmiddlefrontal_11)(rh.rostralmiddlefrontal_12)
5371	0.84058	(Left-Accumbens-area)(Left-Caudate)(Left-Hippocampus)(Left-Pallidum)(Left-Thalamus-Proper)
5372	0.84058	(Left-Accumbens-area)(Left-Caudate)(Left-Hippocampus)(Left-Pallidum)
5373	0.84058	(Left-Accumbens-area)(Left-Caudate)(Left-Hippocampus)(Left-Putamen)(Left-Thalamus-Proper)
5374	0.84058	(Left-Accumbens-area)(Left-Caudate)(Left-Hippocampus)(Left-Putamen)
5375	0.84058	(Left-Accumbens-area)(Left-Caudate)(Left-Thalamus-Proper)(Right-Caudate)
5376	0.84058	(Left-Accumbens-area)(Left-Caudate)(lh.lateralorbitofrontal_2)
5377	0.84058	(Left-Caudate)(Left-Pallidum)(lh.precentral_7)
5378	0.84058	(Left-Caudate)(Left-Putamen)(lh.caudalanteriorcingulate_1)(lh.rostralmiddlefrontal_4)
5379	0.84058	(Left-Caudate)(Left-Putamen)(lh.lateralorbitofrontal_6)(lh.lateralorbitofrontal_7)(lh.rostralmiddlefrontal_12)
5380	0.84058	(Left-Caudate)(Left-Putamen)(lh.postcentral_8)(lh.precentral_14)
5381	0.84058	(Left-Caudate)(Left-Putamen)(lh.rostralanteriorcingulate_2)(lh.rostralmiddlefrontal_4)(lh.rostralmiddlefrontal_9)
5382	0.84058	(Left-Caudate)(Left-Thalamus-Proper)(lh.postcentral_8)(lh.precentral_7)
5383	0.84058	(Left-Caudate)(lh.caudalanteriorcingulate_2)(lh.posteriorcingulate_1)(lh.superiorfrontal_14)
5384	0.84058	(Left-Caudate)(lh.caudalmiddlefrontal_1)(lh.caudalmiddlefrontal_3)(lh.rostralmiddlefrontal_1)
5385	0.84058	(Left-Caudate)(lh.caudalmiddlefrontal_1)(lh.caudalmiddlefrontal_3)(lh.superiorfrontal_11)
5386	0.84058	(Left-Caudate)(lh.lateralorbitofrontal_7)(lh.parstriangularis_3)(lh.rostralanteriorcingulate_1)(lh.rostralmiddlefrontal_9)
5387	0.84058	(Left-Caudate)(lh.lateralorbitofrontal_7)(lh.rostralanteriorcingulate_1)(lh.rostralmiddlefrontal_12)(lh.rostralmiddlefrontal_9)
5388	0.84058	(Left-Caudate)(lh.medialorbitofrontal_2)(lh.parstriangularis_3)(lh.rostralmiddlefrontal_9)
5389	0.84058	(Left-Caudate)(lh.rostralanteriorcingulate_2)(lh.rostralmiddlefrontal_9)(lh.superiorfrontal_4)
5390	0.84058	(Left-Putamen)(lh.caudalanteriorcingulate_2)(lh.superiorfrontal_11)(lh.superiorfrontal_4)
5391	0.84058	(Left-Putamen)(lh.caudalmiddlefrontal_3)(lh.rostralmiddlefrontal_3)
5392	0.84058	(Left-Putamen)(lh.insula_6)(lh.rostralmiddlefrontal_4)(lh.rostralmiddlefrontal_9)
5393	0.84058	(Left-Putamen)(lh.lateralorbitofrontal_2)(lh.lateralorbitofrontal_3)
5394	0.84058	(Left-Putamen)(lh.lateralorbitofrontal_4)(lh.lateralorbitofrontal_7)(lh.rostralanteriorcingulate_1)
5395	0.84058	(Left-Putamen)(lh.lateralorbitofrontal_7)(lh.medialorbitofrontal_2)(lh.parstriangularis_3)(lh.rostralmiddlefrontal_12)
5396	0.84058	(Left-Putamen)(lh.medialorbitofrontal_2)(lh.parstriangularis_3)(lh.rostralanteriorcingulate_1)
5397	0.84058	(Left-Putamen)(lh.postcentral_14)
5398	0.84058	(Left-Putamen)(lh.precentral_5)
5399	0.84058	(Left-Putamen)(lh.rostralmiddlefrontal_3)(lh.rostralmiddlefrontal_4)
5400	0.84058	(Left-Putamen)(lh.rostralmiddlefrontal_4)(lh.superiorfrontal_3)

5401	0.84058	(Left-Putamen)(lh.superiorfrontal_10)(lh.superiorfrontal_11)(lh.superiorfrontal_4)
5402	0.84058	(Left-Putamen)(lh.superiorfrontal_11)(lh.superiorfrontal_12)(lh.superiorfrontal_14)
5403	0.84058	(lh.caudalmiddlefrontal_3)(lh.parsopercularis_1)
5404	0.84058	(lh.caudalmiddlefrontal_3)(lh.rostralmiddlefrontal_4)(lh.rostralmiddlefrontal_9)
5405	0.84058	(lh.fusiform_1)(lh.fusiform_2)(lh.fusiform_5)(lh.lingual_8)
5406	0.84058	(lh.fusiform_1)(lh.fusiform_2)(lh.lateraloccipital_6)
5407	0.84058	(lh.fusiform_1)(lh.fusiform_2)(lh.lingual_8)(lh.pericalcarine_3)
5408	0.84058	(lh.fusiform_1)(lh.inferiortemporal_8)(lh.lateraloccipital_9)
5409	0.84058	(lh.fusiform_2)(lh.lingual_1)(lh.lingual_8)(lh.pericalcarine_3)
5410	0.84058	(lh.fusiform_6)(lh.inferiortemporal_8)
5411	0.84058	(lh.insula_6)(lh.rostralmiddlefrontal_1)(lh.rostralmiddlefrontal_4)(lh.rostralmiddlefrontal_9)
5412	0.84058	(lh.lateraloccipital_3)(lh.lateraloccipital_5)(lh.pericalcarine_2)
5413	0.84058	(lh.lateraloccipital_3)(lh.lateraloccipital_6)(lh.pericalcarine_2)(lh.pericalcarine_3)
5414	0.84058	(lh.lateralorbitofrontal_2)(lh.lateralorbitofrontal_3)
5415	0.84058	(lh.lateralorbitofrontal_4)(lh.lateralorbitofrontal_6)(lh.rostralmiddlefrontal_6)
5416	0.84058	(lh.lateralorbitofrontal_4)(lh.lateralorbitofrontal_7)(lh.rostralanteriorcingulate_1)
5417	0.84058	(lh.lateralorbitofrontal_4)(lh.medialorbitofrontal_2)
5418	0.84058	(lh.lateralorbitofrontal_6)(lh.lateralorbitofrontal_7)(lh.parstriangularis_3)(lh.rostralmiddlefrontal_6)(lh.rostralmiddlefrontal_9)
5419	0.84058	(lh.lateralorbitofrontal_6)(lh.parstriangularis_2)(lh.parstriangularis_3)(lh.rostralmiddlefrontal_1)
5420	0.84058	(lh.lateralorbitofrontal_7)(lh.medialorbitofrontal_3)(lh.medialorbitofrontal_4)
5421	0.84058	(lh.lateralorbitofrontal_7)(lh.rostralmiddlefrontal_12)(lh.rostralmiddlefrontal_6)(lh.rostralmiddlefrontal_9)
5422	0.84058	(lh.medialorbitofrontal_2)(lh.medialorbitofrontal_3)
5423	0.84058	(lh.paracentral_1)(lh.paracentral_4)(lh.superiorfrontal_16)
5424	0.84058	(lh.paracentral_3)(lh.postcentral_1)
5425	0.84058	(lh.parstriangularis_2)(lh.parstriangularis_3)(lh.rostralmiddlefrontal_1)(lh.rostralmiddlefrontal_9)
5426	0.84058	(lh.parstriangularis_3)(lh.rostralmiddlefrontal_8)(lh.rostralmiddlefrontal_9)
5427	0.84058	(lh.precentral_4)(lh.precentral_9)
5428	0.84058	(lh.precentral_8)(lh.precentral_9)
5429	0.84058	(lh.precuneus_2)(lh.superiorparietal_6)
5430	0.84058	(lh.precuneus_2)(lh.superiorparietal_7)
5431	0.84058	(lh.precuneus_3)(lh.precuneus_4)
5432	0.84058	(lh.precuneus_6)(lh.precuneus_9)(lh.superiorparietal_11)(lh.superiorparietal_6)
5433	0.84058	(lh.rostralanteriorcingulate_1)(lh.rostralmiddlefrontal_8)(lh.superiorfrontal_3)
5434	0.84058	(lh.rostralmiddlefrontal_1)(lh.rostralmiddlefrontal_8)(lh.rostralmiddlefrontal_9)
5435	0.84058	(lh.superiorfrontal_10)(lh.superiorfrontal_14)(lh.superiorfrontal_4)(lh.superiorfrontal_8)
5436	0.84058	(lh.superiorfrontal_10)(lh.superiorfrontal_14)(lh.superiorfrontal_4)
5437	0.84058	(lh.superiorfrontal_10)(lh.superiorfrontal_14)(lh.superiorfrontal_9)
5438	0.84058	(lh.superiorfrontal_11)(lh.superiorfrontal_4)(lh.superiorfrontal_6)(lh.superiorfrontal_7)(lh.superiorfrontal_8)
5439	0.84058	(lh.superiorfrontal_11)(lh.superiorfrontal_6)(lh.superiorfrontal_7)(lh.superiorfrontal_8)
5440	0.84058	(lh.superiorfrontal_12)(lh.superiorfrontal_13)(lh.superiorfrontal_14)
5441	0.84058	(lh.superiorfrontal_13)(lh.superiorfrontal_7)(lh.superiorfrontal_9)
5442	0.84058	(lh.superiorfrontal_13)(lh.superiorfrontal_7)
5443	0.84058	(lh.superiortemporal_11)(lh.superiortemporal_8)
5444	0.84058	(lh.supramarginal_1)(lh.supramarginal_2)(lh.supramarginal_6)
5445	0.84058	(rh.fusiform_1)(rh.lateraloccipital_4)(rh.lateraloccipital_8)(rh.pericalcarine_1)
5446	0.84058	(rh.fusiform_3)(rh.inferiortemporal_7)(rh.middletemporal_1)
5447	0.84058	(rh.inferiorparietal_12)(rh.inferiorparietal_4)
5448	0.84058	(rh.inferiortemporal_2)(rh.superiortemporal_10)
5449	0.84058	(rh.inferiortemporal_5)(rh.middletemporal_4)(rh.middletemporal_6)
5450	0.84058	(rh.insula_3)(rh.precentral_1)

5451	0.84058	(rh.lateralorbitofrontal_5)(rh.lateralorbitofrontal_6)
5452	0.84058	(rh.medialorbitofrontal_2)(rh.rostralmiddlefrontal_11)(rh.superiorfrontal_1)(rh.superiorfrontal_2)
5453	0.84058	(rh.middletemporal_4)(rh.middletemporal_5)(rh.middletemporal_7)
5454	0.84058	(rh.parsopercularis_2)(rh.parstriangularis_1)(rh.parstriangularis_2)
5455	0.84058	(rh.parsorbitalis_1)(rh.parsorbitalis_2)
5456	0.84058	(rh.precuneus_10)(rh.precuneus_7)(rh.superiorparietal_7)(rh.superiorparietal_8)
5457	0.84058	(rh.precuneus_2)(rh.superiorparietal_10)
5458	0.84058	(rh.precuneus_4)(rh.superiorparietal_10)(rh.superiorparietal_8)
5459	0.84058	(rh.precuneus_7)(rh.superiorparietal_11)
5460	0.84058	(rh.rostralmiddlefrontal_10)(rh.rostralmiddlefrontal_12)
5461	0.84058	(rh.superiorfrontal_1)(rh.superiorfrontal_3)(rh.superiorfrontal_4)
5462	0.84058	(rh.superiortemporal_1)(rh.superiortemporal_3)(rh.superiortemporal_9)
5463	0.84058	(rh.supramarginal_1)(rh.supramarginal_6)(rh.supramarginal_9)
5464	0.84058	(Right-Caudate)(rh.parstriangularis_1)(rh.rostralmiddlefrontal_12)
5465	0.84058	(Right-Caudate)(rh.precentral_10)(rh.precentral_13)
5466	0.84058	(Right-Caudate)(Right-Pallidum)(rh.insula_1)
5467	0.84058	(Right-Caudate)(Right-Pallidum)(Right-Putamen)(rh.insula_1)
5468	0.84058	(Right-Caudate)(Right-Putamen)(rh.insula_7)(rh.parstriangularis_1)
5469	0.84058	(Right-Caudate)(Right-Putamen)(rh.lateralorbitofrontal_7)(rh.parstriangularis_1)
5470	0.84058	(Right-Caudate)(Right-Putamen)(rh.supramarginal_8)
5471	0.84058	(Right-Hippocampus)(rh.fusiform_7)(rh.parahippocampal_2)
5472	0.84058	(Right-Hippocampus)(rh.insula_5)(rh.middletemporal_9)
5473	0.84058	(Right-Hippocampus)(rh.isthmuscingulate_2)(rh.precuneus_2)(rh.precuneus_4)
5474	0.84058	(Right-Pallidum)(rh.precentral_13)(rh.precentral_7)
5475	0.84058	(Right-Pallidum)(Right-Putamen)(rh.precentral_13)(rh.precentral_7)
5476	0.84058	(Right-Putamen)(rh.medialorbitofrontal_2)(rh.rostralmiddlefrontal_11)(rh.superiorfrontal_2)
5477	0.84058	(Right-Putamen)(rh.precentral_13)(rh.precentral_6)
5478	0.84058	(Right-Putamen)(rh.superiorfrontal_11)(rh.superiorfrontal_13)
5479	0.84058	(Right-Putamen)(rh.transversetemporal_1)
5480	0.83816	(Left-Accumbens-area)(Right-Accumbens-area)
5481	0.83816	(Left-Amygdala)(lh.insula_4)(lh.superiortemporal_11)
5482	0.83816	(Left-Caudate)(Left-Pallidum)(Left-Putamen)(Left-Thalamus-Proper)(lh.precentral_7)
5483	0.83816	(Left-Caudate)(Left-Pallidum)(Left-Putamen)(lh.precentral_11)(lh.precentral_9)
5484	0.83816	(Left-Caudate)(Left-Pallidum)(Left-Putamen)(lh.precentral_7)
5485	0.83816	(Left-Caudate)(Left-Pallidum)(Left-Thalamus-Proper)(lh.precentral_7)
5486	0.83816	(Left-Caudate)(Left-Pallidum)(lh.precentral_11)(lh.precentral_9)
5487	0.83816	(Left-Caudate)(Left-Putamen)(lh.caudalanteriorcingulate_2)(lh.rostralanteriorcingulate_2)(lh.superiorfrontal_4)
5488	0.83816	(Left-Caudate)(Left-Putamen)(lh.caudalmiddlefrontal_4)(lh.caudalmiddlefrontal_6)(lh.precentral_9)
5489	0.83816	(Left-Caudate)(Left-Putamen)(lh.caudalmiddlefrontal_5)(lh.precentral_9)
5490	0.83816	(Left-Caudate)(Left-Putamen)(lh.lateralorbitofrontal_4)(lh.lateralorbitofrontal_6)(lh.rostralmiddlefrontal_9)
5491	0.83816	(Left-Caudate)(Left-Putamen)(lh.lateralorbitofrontal_6)(lh.lateralorbitofrontal_7)(lh.parstriangularis_3)
5492	0.83816	(Left-Caudate)(Left-Putamen)(lh.lateralorbitofrontal_6)(lh.lateralorbitofrontal_7)(lh.rostralmiddlefrontal_9)
5493	0.83816	(Left-Caudate)(Left-Putamen)(lh.medialorbitofrontal_5)(lh.rostralanteriorcingulate_1)
5494	0.83816	(Left-Caudate)(Left-Putamen)(lh.parsopercularis_3)(lh.parstriangularis_3)(lh.rostralmiddlefrontal_1)
5495	0.83816	(Left-Caudate)(Left-Putamen)(lh.precentral_4)(lh.superiorfrontal_14)
5496	0.83816	(Left-Caudate)(Left-Putamen)(lh.rostralanteriorcingulate_1)(lh.rostralmiddlefrontal_4)(lh.superiorfrontal_4)
5497	0.83816	(Left-Caudate)(Left-Putamen)(lh.rostralmiddlefrontal_8)(lh.superiorfrontal_4)
5498	0.83816	(Left-Caudate)(lh.caudalmiddlefrontal_1)(lh.caudalmiddlefrontal_3)(lh.caudalmiddlefrontal_4)(lh.caudalmiddlefrontal_5)
5499	0.83816	(Left-Caudate)(lh.caudalmiddlefrontal_3)(lh.insula_6)(lh.parstriangularis_3)
5500	0.83816	(Left-Caudate)(lh.lateralorbitofrontal_6)(lh.parstriangularis_3)(lh.rostralmiddlefrontal_1)(lh.rostralmiddlefrontal_9)

5501	0.83816	(Left-Caudate)(lh.medialorbitofrontal_2)(lh.rostralanteriorcingulate_1)(lh.rostralmiddlefrontal_9)(lh.superiorfrontal_4)
5502	0.83816	(Left-Caudate)(lh.parstriangularis_3)(lh.rostralanteriorcingulate_1)(lh.rostralmiddlefrontal_1)(lh.rostralmiddlefrontal_9)
5503	0.83816	(Left-Caudate)(lh.postcentral_8)(lh.precentral_9)
5504	0.83816	(Left-Caudate)(lh.rostralanteriorcingulate_1)(lh.superiorfrontal_3)(lh.superiorfrontal_4)
5505	0.83816	(Left-Caudate)(lh.superiorfrontal_10)(lh.superiorfrontal_11)(lh.superiorfrontal_14)
5506	0.83816	(Left-Pallidum)(Left-Putamen)(lh.precentral_14)
5507	0.83816	(Left-Pallidum)(lh.precentral_14)
5508	0.83816	(Left-Putamen)(lh.caudalmiddlefrontal_1)(lh.superiorfrontal_11)
5509	0.83816	(Left-Putamen)(lh.caudalmiddlefrontal_4)(lh.insula_3)
5510	0.83816	(Left-Putamen)(lh.insula_6)(lh.parstriangularis_3)(lh.rostralmiddlefrontal_1)(lh.rostralmiddlefrontal_4)
5511	0.83816	(Left-Putamen)(lh.lateralorbitofrontal_2)(lh.lateralorbitofrontal_7)(lh.medialorbitofrontal_5)
5512	0.83816	(Left-Putamen)(lh.lateralorbitofrontal_7)(lh.parstriangularis_3)(lh.rostralanteriorcingulate_1)(lh.rostralmiddlefrontal_12)(lh.rostralmiddlefrontal_9)
5513	0.83816	(Left-Putamen)(lh.rostralmiddlefrontal_4)(lh.rostralmiddlefrontal_8)(lh.superiorfrontal_4)
5514	0.83816	(Left-Putamen)(lh.rostralmiddlefrontal_9)(lh.superiorfrontal_3)(lh.superiorfrontal_4)
5515	0.83816	(Left-Putamen)(lh.supramarginal_4)
5516	0.83816	(Left-Thalamus-Proper)(lh.insula_1)(lh.superiorparietal_3)
5517	0.83816	(Left-Thalamus-Proper)(lh.postcentral_8)(lh.precentral_11)
5518	0.83816	(lh.caudalanteriorcingulate_2)(lh.superiorfrontal_10)(lh.superiorfrontal_14)
5519	0.83816	(lh.caudalmiddlefrontal_4)(lh.insula_3)
5520	0.83816	(lh.cuneus_2)(lh.cuneus_3)
5521	0.83816	(lh.fusiform_1)(lh.fusiform_2)(lh.lateraloccipital_10)
5522	0.83816	(lh.fusiform_1)(lh.fusiform_2)(lh.lateraloccipital_7)
5523	0.83816	(lh.fusiform_2)(lh.fusiform_5)(lh.lateraloccipital_9)
5524	0.83816	(lh.fusiform_2)(lh.pericalcarine_2)(lh.pericalcarine_3)
5525	0.83816	(lh.inferiorparietal_4)(lh.lateraloccipital_6)
5526	0.83816	(lh.inferiorparietal_8)(lh.precuneus_9)(lh.superiorparietal_11)
5527	0.83816	(lh.insula_1)(lh.postcentral_14)(lh.supramarginal_4)
5528	0.83816	(lh.isthmuscingulate_2)(lh.precuneus_6)(lh.superiorparietal_5)
5529	0.83816	(lh.lateraloccipital_3)(lh.pericalcarine_1)(lh.pericalcarine_3)
5530	0.83816	(lh.lateralorbitofrontal_4)(lh.parstriangularis_1)(lh.parstriangularis_3)
5531	0.83816	(lh.lateralorbitofrontal_6)(lh.parsorbitalis_1)(lh.rostralmiddlefrontal_9)
5532	0.83816	(lh.lateralorbitofrontal_7)(lh.medialorbitofrontal_2)(lh.parstriangularis_3)(lh.rostralmiddlefrontal_12)(lh.rostralmiddlefrontal_9)
5533	0.83816	(lh.medialorbitofrontal_2)(lh.parstriangularis_3)(lh.rostralanteriorcingulate_1)(lh.rostralmiddlefrontal_12)
5534	0.83816	(lh.medialorbitofrontal_2)(lh.rostralanteriorcingulate_1)(lh.rostralmiddlefrontal_9)(lh.superiorfrontal_3)(lh.superiorfrontal_4)
5535	0.83816	(lh.medialorbitofrontal_2)(lh.rostralmiddlefrontal_12)(lh.superiorfrontal_3)(lh.superiorfrontal_4)
5536	0.83816	(lh.paracentral_1)(lh.paracentral_3)
5537	0.83816	(lh.postcentral_3)(lh.postcentral_8)
5538	0.83816	(lh.precentral_11)(lh.precentral_12)(lh.precentral_14)
5539	0.83816	(lh.precentral_4)(lh.superiorfrontal_14)(lh.superiorfrontal_15)
5540	0.83816	(lh.precentral_7)(lh.superiorparietal_3)
5541	0.83816	(lh.precentral_7)(lh.supramarginal_4)
5542	0.83816	(lh.rostralanteriorcingulate_1)(lh.rostralanteriorcingulate_2)(lh.rostralmiddlefrontal_8)
5543	0.83816	(lh.rostralanteriorcingulate_1)(lh.rostralmiddlefrontal_4)(lh.superiorfrontal_3)
5544	0.83816	(lh.rostralanteriorcingulate_2)(lh.rostralmiddlefrontal_4)(lh.superiorfrontal_11)
5545	0.83816	(lh.rostralmiddlefrontal_1)(lh.rostralmiddlefrontal_12)(lh.rostralmiddlefrontal_6)
5546	0.83816	(lh.rostralmiddlefrontal_1)(lh.superiorfrontal_10)
5547	0.83816	(lh.superiorfrontal_11)(lh.superiorfrontal_13)(lh.superiorfrontal_14)(lh.superiorfrontal_8)(lh.superiorfrontal_9)
5548	0.83816	(rh.bankssts_1)(rh.inferiortemporal_2)
5549	0.83816	(rh.caudalmiddlefrontal_4)(rh.precentral_10)
5550	0.83816	(rh.caudalmiddlefrontal_4)(rh.precentral_5)

5551	0.83816	(rh.caudalmiddlefrontal_5)(rh.rostralmiddlefrontal_5)
5552	0.83816	(rh.fusiform_1)(rh.inferiorparietal_10)(rh.lingual_5)
5553	0.83816	(rh.fusiform_1)(rh.lingual_2)(rh.lingual_5)
5554	0.83816	(rh.fusiform_5)(rh.fusiform_6)
5555	0.83816	(rh.inferiorparietal_10)(rh.inferiorparietal_12)(rh.lingual_7)
5556	0.83816	(rh.inferiorparietal_10)(rh.inferiortemporal_7)
5557	0.83816	(rh.inferiorparietal_10)(rh.lateraloccipital_10)(rh.lingual_7)
5558	0.83816	(rh.inferiorparietal_12)(rh.lateraloccipital_8)(rh.superiorparietal_11)
5559	0.83816	(rh.inferiorparietal_12)(rh.pericalcarine_1)(rh.superiorparietal_11)
5560	0.83816	(rh.inferiorparietal_4)(rh.inferiorparietal_6)(rh.inferiorparietal_8)
5561	0.83816	(rh.inferiorparietal_6)(rh.superiorparietal_11)
5562	0.83816	(rh.inferiortemporal_2)(rh.middletemporal_4)(rh.middletemporal_5)
5563	0.83816	(rh.isthmuscingulate_2)(rh.precuneus_2)(rh.precuneus_4)(rh.precuneus_7)
5564	0.83816	(rh.lateraloccipital_2)(rh.lateraloccipital_3)
5565	0.83816	(rh.lateralorbitofrontal_2)(rh.parstriangularis_4)
5566	0.83816	(rh.lateralorbitofrontal_6)(rh.parstriangularis_4)(rh.rostralmiddlefrontal_8)
5567	0.83816	(rh.lateralorbitofrontal_7)(rh.medialorbitofrontal_2)(rh.rostralmiddlefrontal_12)(rh.rostralmiddlefrontal_13)
5568	0.83816	(rh.medialorbitofrontal_1)(rh.superiorfrontal_1)
5569	0.83816	(rh.medialorbitofrontal_2)(rh.rostralmiddlefrontal_8)
5570	0.83816	(rh.paracentral_4)(rh.superiorfrontal_11)(rh.superiorfrontal_16)
5571	0.83816	(rh.parsopercularis_4)(rh.rostralmiddlefrontal_3)(rh.rostralmiddlefrontal_5)
5572	0.83816	(rh.parsorbitalis_1)(rh.rostralmiddlefrontal_12)
5573	0.83816	(rh.parstriangularis_1)(rh.rostralmiddlefrontal_6)(rh.rostralmiddlefrontal_8)
5574	0.83816	(rh.postcentral_1)(rh.supramarginal_6)
5575	0.83816	(rh.postcentral_2)(rh.precentral_3)
5576	0.83816	(rh.postcentral_3)(rh.supramarginal_7)
5577	0.83816	(rh.rostralmiddlefrontal_10)(rh.rostralmiddlefrontal_9)(rh.superiorfrontal_2)
5578	0.83816	(rh.superiorfrontal_2)(rh.superiorfrontal_3)(rh.superiorfrontal_6)
5579	0.83816	(rh.superiorfrontal_2)(rh.superiorfrontal_8)(rh.superiorfrontal_9)
5580	0.83816	(rh.superiorfrontal_4)(rh.superiorfrontal_5)(rh.superiorfrontal_8)
5581	0.83816	(rh.superiortemporal_10)(rh.superiortemporal_3)
5582	0.83816	(rh.superiortemporal_10)(rh.superiortemporal_6)(rh.superiortemporal_7)
5583	0.83816	(Right-Caudate)(rh.caudalmiddlefrontal_2)(rh.caudalmiddlefrontal_4)
5584	0.83816	(Right-Caudate)(rh.caudalmiddlefrontal_5)(rh.rostralmiddlefrontal_1)
5585	0.83816	(Right-Caudate)(rh.insula_1)(rh.precentral_11)(rh.precentral_7)
5586	0.83816	(Right-Caudate)(rh.superiorfrontal_5)(rh.superiorfrontal_9)
5587	0.83816	(Right-Caudate)(Right-Hippocampus)(rh.isthmuscingulate_1)
5588	0.83816	(Right-Caudate)(Right-Putamen)(rh.lateralorbitofrontal_7)(rh.superiorfrontal_2)
5589	0.83816	(Right-Hippocampus)(rh.inferiortemporal_2)(rh.superiortemporal_3)
5590	0.83816	(Right-Hippocampus)(rh.inferiortemporal_2)(rh.superiortemporal_6)
5591	0.83816	(Right-Hippocampus)(Right-Putamen)(rh.superiorparietal_1)
5592	0.83816	(Right-Pallidum)(rh.precentral_6)
5593	0.83816	(Right-Pallidum)(Right-Putamen)(rh.precentral_6)
5594	0.83816	(Right-Pallidum)(Right-Putamen)(Right-Thalamus-Proper)(rh.insula_1)
5595	0.83816	(Right-Pallidum)(Right-Thalamus-Proper)(rh.insula_1)
5596	0.83816	(Right-Putamen)(rh.caudalmiddlefrontal_4)(rh.caudalmiddlefrontal_5)(rh.rostralmiddlefrontal_1)
5597	0.83816	(Right-Putamen)(rh.caudalmiddlefrontal_4)(rh.precentral_5)
5598	0.83816	(Right-Putamen)(rh.lateralorbitofrontal_7)(rh.rostralmiddlefrontal_12)(rh.rostralmiddlefrontal_13)
5599	0.83816	(Right-Putamen)(rh.parsopercularis_4)(rh.parstriangularis_1)(rh.rostralmiddlefrontal_3)
5600	0.83816	(Right-Putamen)(rh.precentral_11)(rh.superiorparietal_1)

5601	0.83816	(Right-Putamen)(rh.precentral_12)(rh.precentral_13)
5602	0.83816	(Right-Thalamus-Proper)(rh.isthmuscingulate_1)(rh.isthmuscingulate_2)
5603	0.83575	(Left-Accumbens-area)(Left-Caudate)(Left-Hippocampus)(Left-Pallidum)(Left-Putamen)(Left-Thalamus-Proper)
5604	0.83575	(Left-Accumbens-area)(Left-Caudate)(Left-Hippocampus)(Left-Pallidum)(Left-Putamen)
5605	0.83575	(Left-Accumbens-area)(Left-Caudate)(Left-Putamen)(lh.lateralorbitofrontal_2)
5606	0.83575	(Left-Amygdala)(Left-Hippocampus)(lh.inferiortemporal_1)(lh.insula_4)
5607	0.83575	(Left-Caudate)(Left-Putamen)(Left-Thalamus-Proper)(lh.caudalmiddlefrontal_3)
5608	0.83575	(Left-Caudate)(Left-Putamen)(Left-Thalamus-Proper)(lh.postcentral_8)(lh.precentral_7)
5609	0.83575	(Left-Caudate)(Left-Putamen)(lh.caudalmiddlefrontal_1)(lh.caudalmiddlefrontal_3)(lh.rostralmiddlefrontal_4)
5610	0.83575	(Left-Caudate)(Left-Putamen)(lh.lateralorbitofrontal_4)(lh.lateralorbitofrontal_7)(lh.parstriangularis_3)
5611	0.83575	(Left-Caudate)(Left-Putamen)(lh.lateralorbitofrontal_7)(lh.rostralanteriorcingulate_1)(lh.rostralmiddlefrontal_12)(lh.rostralmiddlefrontal_9)
5612	0.83575	(Left-Caudate)(Left-Putamen)(lh.medialorbitofrontal_2)(lh.superiorfrontal_3)
5613	0.83575	(Left-Caudate)(Left-Putamen)(lh.rostralanteriorcingulate_1)(lh.superiorfrontal_3)
5614	0.83575	(Left-Caudate)(Left-Putamen)(lh.rostralanteriorcingulate_2)(lh.rostralmiddlefrontal_1)
5615	0.83575	(Left-Caudate)(Left-Putamen)(lh.rostralmiddlefrontal_1)(lh.rostralmiddlefrontal_12)
5616	0.83575	(Left-Caudate)(Left-Thalamus-Proper)(lh.caudalmiddlefrontal_3)
5617	0.83575	(Left-Caudate)(Left-Thalamus-Proper)(lh.postcentral_8)(lh.precentral_4)
5618	0.83575	(Left-Caudate)(lh.caudalanteriorcingulate_2)(lh.rostralanteriorcingulate_1)(lh.superiorfrontal_4)
5619	0.83575	(Left-Caudate)(lh.caudalanteriorcingulate_2)(lh.rostralanteriorcingulate_2)(lh.superiorfrontal_10)
5620	0.83575	(Left-Caudate)(lh.caudalanteriorcingulate_2)(lh.superiorfrontal_11)(lh.superiorfrontal_4)(lh.superiorfrontal_8)
5621	0.83575	(Left-Caudate)(lh.insula_1)(lh.postcentral_8)(lh.precentral_11)
5622	0.83575	(Left-Caudate)(lh.lateralorbitofrontal_4)(lh.lateralorbitofrontal_7)(lh.rostralmiddlefrontal_9)
5623	0.83575	(Left-Caudate)(lh.lateralorbitofrontal_6)(lh.parstriangularis_3)(lh.rostralmiddlefrontal_12)(lh.rostralmiddlefrontal_9)
5624	0.83575	(Left-Caudate)(lh.lateralorbitofrontal_7)(lh.medialorbitofrontal_2)(lh.parstriangularis_3)
5625	0.83575	(Left-Caudate)(lh.lateralorbitofrontal_7)(lh.medialorbitofrontal_4)(lh.medialorbitofrontal_5)
5626	0.83575	(Left-Caudate)(lh.parsopercularis_1)(lh.rostralmiddlefrontal_1)
5627	0.83575	(Left-Caudate)(lh.postcentral_8)(lh.precentral_11)(lh.precentral_4)
5628	0.83575	(Left-Caudate)(lh.rostralanteriorcingulate_1)(lh.rostralmiddlefrontal_9)(lh.superiorfrontal_3)
5629	0.83575	(Left-Caudate)(lh.rostralanteriorcingulate_2)(lh.superiorfrontal_10)(lh.superiorfrontal_11)
5630	0.83575	(Left-Caudate)(lh.rostralmiddlefrontal_12)(lh.superiorfrontal_4)
5631	0.83575	(Left-Hippocampus)(Left-Putamen)(lh.transversetemporal_2)
5632	0.83575	(Left-Hippocampus)(lh.fusiform_5)(lh.lingual_6)(lh.lingual_8)
5633	0.83575	(Left-Pallidum)(Left-Putamen)(Left-Thalamus-Proper)(lh.postcentral_8)
5634	0.83575	(Left-Pallidum)(Left-Thalamus-Proper)(lh.postcentral_8)
5635	0.83575	(Left-Putamen)(Left-Thalamus-Proper)(lh.caudalmiddlefrontal_3)
5636	0.83575	(Left-Putamen)(Left-Thalamus-Proper)(lh.paracentral_5)
5637	0.83575	(Left-Putamen)(Left-Thalamus-Proper)(lh.postcentral_8)(lh.precentral_11)
5638	0.83575	(Left-Putamen)(lh.caudalanteriorcingulate_2)(lh.superiorfrontal_10)
5639	0.83575	(Left-Putamen)(lh.caudalmiddlefrontal_4)(lh.caudalmiddlefrontal_6)(lh.precentral_11)(lh.precentral_9)
5640	0.83575	(Left-Putamen)(lh.caudalmiddlefrontal_5)(lh.caudalmiddlefrontal_6)(lh.precentral_9)
5641	0.83575	(Left-Putamen)(lh.insula_3)(lh.precentral_14)
5642	0.83575	(Left-Putamen)(lh.lateralorbitofrontal_4)(lh.lateralorbitofrontal_6)(lh.lateralorbitofrontal_7)(lh.parstriangularis_3)(lh.rostralmiddlefrontal_12)(lh.rostralmiddlefrontal_9)
5643	0.83575	(Left-Putamen)(lh.lateralorbitofrontal_4)(lh.parstriangularis_3)(lh.rostralanteriorcingulate_1)(lh.rostralmiddlefrontal_9)
5644	0.83575	(Left-Putamen)(lh.lateralorbitofrontal_6)(lh.medialorbitofrontal_2)
5645	0.83575	(Left-Putamen)(lh.lateralorbitofrontal_7)(lh.medialorbitofrontal_2)(lh.parstriangularis_3)(lh.rostralmiddlefrontal_9)
5646	0.83575	(Left-Putamen)(lh.rostralanteriorcingulate_1)(lh.rostralmiddlefrontal_12)(lh.superiorfrontal_4)
5647	0.83575	(Left-Putamen)(lh.rostralmiddlefrontal_1)(lh.rostralmiddlefrontal_3)
5648	0.83575	(Left-Putamen)(lh.rostralmiddlefrontal_4)(lh.rostralmiddlefrontal_8)(lh.rostralmiddlefrontal_9)
5649	0.83575	(Left-Thalamus-Proper)(lh.caudalmiddlefrontal_3)
5650	0.83575	(Left-Thalamus-Proper)(lh.precentral_14)

5651	0.83575	(lh.bankssts_2)(lh.fusiform_6)
5652	0.83575	(lh.bankssts_3)(lh.lateraloccipital_9)
5653	0.83575	(lh.caudalmiddlefrontal_1)(lh.caudalmiddlefrontal_6)
5654	0.83575	(lh.cuneus_2)(lh.pericalcarine_1)(lh.pericalcarine_2)
5655	0.83575	(lh.fusiform_2)(lh.lateraloccipital_6)(lh.lateraloccipital_7)(lh.lingual_1)
5656	0.83575	(lh.fusiform_2)(lh.lateraloccipital_7)(lh.lateraloccipital_9)(lh.lingual_8)
5657	0.83575	(lh.fusiform_2)(lh.lingual_1)(lh.lingual_4)(lh.lingual_8)
5658	0.83575	(lh.inferiorparietal_10)(lh.inferiorparietal_6)(lh.inferiorparietal_9)
5659	0.83575	(lh.inferiorparietal_3)(lh.inferiorparietal_8)
5660	0.83575	(lh.inferiorparietal_9)(lh.superiorparietal_6)
5661	0.83575	(lh.lateraloccipital_3)(lh.lateraloccipital_4)
5662	0.83575	(lh.lateraloccipital_3)(lh.lateraloccipital_6)(lh.lingual_1)(lh.pericalcarine_3)
5663	0.83575	(lh.lateralorbitofrontal_4)(lh.rostralmiddlefrontal_1)
5664	0.83575	(lh.lateralorbitofrontal_7)(lh.medialorbitofrontal_2)(lh.rostralmiddlefrontal_11)
5665	0.83575	(lh.lingual_7)(lh.parahippocampal_3)
5666	0.83575	(lh.paracentral_1)(lh.paracentral_5)(lh.precentral_2)
5667	0.83575	(lh.paracentral_4)(lh.superiorfrontal_14)(lh.superiorfrontal_15)
5668	0.83575	(lh.parsorbitalis_1)(lh.parstriangularis_3)(lh.rostralmiddlefrontal_9)
5669	0.83575	(lh.parstriangularis_3)(lh.rostralmiddlefrontal_12)(lh.rostralmiddlefrontal_4)
5670	0.83575	(lh.pericalcarine_2)(lh.superiorparietal_14)
5671	0.83575	(lh.precentral_11)(lh.precentral_4)(lh.precentral_9)
5672	0.83575	(lh.precuneus_10)(lh.precuneus_6)(lh.superiorparietal_6)
5673	0.83575	(lh.precuneus_10)(lh.superiorparietal_5)
5674	0.83575	(lh.rostralmiddlefrontal_1)(lh.rostralmiddlefrontal_12)(lh.rostralmiddlefrontal_6)(lh.rostralmiddlefrontal_9)
5675	0.83575	(lh.rostralmiddlefrontal_1)(lh.rostralmiddlefrontal_4)(lh.rostralmiddlefrontal_8)
5676	0.83575	(lh.rostralmiddlefrontal_9)(lh.superiorfrontal_1)(lh.superiorfrontal_4)
5677	0.83575	(lh.superiorfrontal_11)(lh.superiorfrontal_14)(lh.superiorfrontal_4)(lh.superiorfrontal_7)(lh.superiorfrontal_8)
5678	0.83575	(lh.superiorfrontal_11)(lh.superiorfrontal_14)(lh.superiorfrontal_4)(lh.superiorfrontal_7)
5679	0.83575	(lh.superiorfrontal_14)(lh.superiorfrontal_4)(lh.superiorfrontal_8)(lh.superiorfrontal_9)
5680	0.83575	(lh.superiorparietal_5)(lh.superiorparietal_6)(lh.superiorparietal_7)
5681	0.83575	(rh.bankssts_1)(rh.bankssts_2)(rh.inferiortemporal_6)
5682	0.83575	(rh.bankssts_1)(rh.inferiortemporal_6)(rh.middletemporal_2)
5683	0.83575	(rh.bankssts_1)(rh.superiortemporal_3)
5684	0.83575	(rh.bankssts_2)(rh.fusiform_7)
5685	0.83575	(rh.caudalmiddlefrontal_4)(rh.precentral_1)
5686	0.83575	(rh.caudalmiddlefrontal_4)(rh.rostralmiddlefrontal_3)(rh.rostralmiddlefrontal_8)
5687	0.83575	(rh.fusiform_2)(rh.lateraloccipital_8)(rh.lingual_5)(rh.lingual_7)
5688	0.83575	(rh.fusiform_5)(rh.inferiortemporal_7)
5689	0.83575	(rh.fusiform_8)(rh.superiortemporal_6)
5690	0.83575	(rh.inferiorparietal_4)(rh.insula_2)(rh.supramarginal_9)
5691	0.83575	(rh.inferiorparietal_8)(rh.superiorparietal_11)
5692	0.83575	(rh.inferiortemporal_1)(rh.superiortemporal_10)
5693	0.83575	(rh.inferiortemporal_5)(rh.inferiortemporal_7)(rh.middletemporal_2)
5694	0.83575	(rh.insula_2)(rh.transversetemporal_1)
5695	0.83575	(rh.insula_3)(rh.precentral_13)
5696	0.83575	(rh.lateraloccipital_3)(rh.lateraloccipital_7)
5697	0.83575	(rh.lateraloccipital_7)(rh.pericalcarine_1)
5698	0.83575	(rh.lateralorbitofrontal_2)(rh.lateralorbitofrontal_7)(rh.rostralmiddlefrontal_8)
5699	0.83575	(rh.lateralorbitofrontal_6)(rh.lateralorbitofrontal_7)(rh.rostralmiddlefrontal_8)
5700	0.83575	(rh.paracentral_4)(rh.superiorfrontal_13)

5701	0.83575	(rh.precentral_10)(rh.precentral_12)(rh.precentral_13)
5702	0.83575	(rh.precentral_11)(rh.precentral_7)(rh.supramarginal_1)
5703	0.83575	(rh.precentral_11)(rh.supramarginal_9)
5704	0.83575	(rh.precentral_13)(rh.superiorfrontal_14)(rh.superiorfrontal_17)
5705	0.83575	(rh.rostralmiddlefrontal_10)(rh.superiorfrontal_2)(rh.superiorfrontal_4)
5706	0.83575	(rh.superiorfrontal_2)(rh.superiorfrontal_6)(rh.superiorfrontal_9)
5707	0.83575	(rh.supramarginal_1)(rh.supramarginal_6)(rh.supramarginal_8)(rh.supramarginal_9)
5708	0.83575	(Right-Accumbens-area)(Right-Amygdala)(Right-Caudate)(Right-Pallidum)(Right-Putamen)(Right-Thalamus-Proper)
5709	0.83575	(Right-Accumbens-area)(Right-Amygdala)(Right-Caudate)(Right-Pallidum)(Right-Putamen)
5710	0.83575	(Right-Accumbens-area)(Right-Amygdala)(Right-Caudate)(Right-Putamen)(Right-Thalamus-Proper)
5711	0.83575	(Right-Accumbens-area)(Right-Amygdala)(Right-Caudate)(Right-Putamen)
5712	0.83575	(Right-Amygdala)(rh.insula_4)(rh.insula_5)(rh.insula_6)
5713	0.83575	(Right-Amygdala)(Right-Putamen)(rh.insula_4)(rh.insula_5)(rh.insula_6)
5714	0.83575	(Right-Caudate)(rh.superiorfrontal_2)(rh.superiorfrontal_5)(rh.superiorfrontal_7)
5715	0.83575	(Right-Caudate)(Right-Pallidum)(Right-Thalamus-Proper)(rh.posteriorcingulate_2)
5716	0.83575	(Right-Caudate)(Right-Putamen)(rh.lateralorbitofrontal_4)(rh.lateralorbitofrontal_5)(rh.lateralorbitofrontal_7)
5717	0.83575	(Right-Caudate)(Right-Putamen)(rh.parstriangularis_1)(rh.rostralmiddlefrontal_12)
5718	0.83575	(Right-Caudate)(Right-Putamen)(rh.precentral_10)(rh.precentral_13)
5719	0.83575	(Right-Caudate)(Right-Putamen)(rh.superiorfrontal_2)(rh.superiorfrontal_7)
5720	0.83575	(Right-Hippocampus)(rh.bankssts_2)(rh.inferiorparietal_4)(rh.insula_2)
5721	0.83575	(Right-Hippocampus)(rh.bankssts_2)(rh.insula_2)(rh.superiortemporal_1)
5722	0.83575	(Right-Hippocampus)(rh.isthmuscingulate_2)(rh.precuneus_2)(rh.precuneus_7)
5723	0.83575	(Right-Hippocampus)(Right-Putamen)(rh.supramarginal_8)
5724	0.83575	(Right-Pallidum)(Right-Thalamus-Proper)(rh.posteriorcingulate_2)
5725	0.83575	(Right-Putamen)(rh.caudalmiddlefrontal_3)
5726	0.83575	(Right-Putamen)(rh.lateralorbitofrontal_6)(rh.rostralmiddlefrontal_12)(rh.rostralmiddlefrontal_13)
5727	0.83575	(Right-Putamen)(rh.medialorbitofrontal_2)(rh.rostralmiddlefrontal_8)
5728	0.83575	(Right-Putamen)(rh.parsopercularis_4)(rh.rostralmiddlefrontal_5)
5729	0.83575	(Right-Putamen)(rh.superiorfrontal_2)(rh.superiorfrontal_5)
5730	0.83333	(Left-Accumbens-area)(Left-Amygdala)(Left-Hippocampus)(Left-Thalamus-Proper)
5731	0.83333	(Left-Accumbens-area)(Left-Amygdala)(Left-Hippocampus)
5732	0.83333	(Left-Caudate)(Left-Putamen)(Left-Thalamus-Proper)(lh.insula_1)(lh.supramarginal_1)
5733	0.83333	(Left-Caudate)(Left-Putamen)(lh.insula_1)(lh.postcentral_8)(lh.precentral_11)
5734	0.83333	(Left-Caudate)(Left-Putamen)(lh.insula_6)(lh.parstriangularis_3)(lh.rostralmiddlefrontal_9)
5735	0.83333	(Left-Caudate)(Left-Putamen)(lh.lateralorbitofrontal_4)(lh.lateralorbitofrontal_7)(lh.rostralmiddlefrontal_9)
5736	0.83333	(Left-Caudate)(Left-Putamen)(lh.lateralorbitofrontal_6)(lh.rostralmiddlefrontal_1)(lh.rostralmiddlefrontal_9)
5737	0.83333	(Left-Caudate)(Left-Putamen)(lh.precentral_11)(lh.precentral_7)(lh.precentral_9)
5738	0.83333	(Left-Caudate)(Left-Putamen)(lh.rostralmiddlefrontal_4)(lh.superiorfrontal_11)(lh.superiorfrontal_4)
5739	0.83333	(Left-Caudate)(Left-Putamen)(lh.rostralmiddlefrontal_9)(lh.superiorfrontal_3)
5740	0.83333	(Left-Caudate)(lh.caudalanteriorcingulate_2)(lh.superiorfrontal_10)(lh.superiorfrontal_4)
5741	0.83333	(Left-Caudate)(lh.caudalmiddlefrontal_5)(lh.caudalmiddlefrontal_6)(lh.precentral_9)
5742	0.83333	(Left-Caudate)(lh.insula_6)(lh.parsopercularis_1)(lh.parsopercularis_3)
5743	0.83333	(Left-Caudate)(lh.insula_6)(lh.parstriangularis_3)(lh.rostralmiddlefrontal_1)(lh.rostralmiddlefrontal_9)
5744	0.83333	(Left-Caudate)(lh.lateralorbitofrontal_4)(lh.lateralorbitofrontal_7)(lh.rostralmiddlefrontal_12)
5745	0.83333	(Left-Caudate)(lh.medialorbitofrontal_2)(lh.parstriangularis_3)(lh.rostralanteriorcingulate_1)
5746	0.83333	(Left-Caudate)(lh.posteriorcingulate_3)(lh.precentral_4)(lh.superiorfrontal_14)
5747	0.83333	(Left-Caudate)(lh.rostralmiddlefrontal_12)(lh.superiorfrontal_3)
5748	0.83333	(Left-Caudate)(lh.rostralmiddlefrontal_4)(lh.superiorfrontal_10)(lh.superiorfrontal_11)(lh.superiorfrontal_4)
5749	0.83333	(Left-Caudate)(lh.supramarginal_4)
5750	0.83333	(Left-Hippocampus)(lh.isthmuscingulate_3)(lh.lingual_7)

5751	0.83333	(Left-Putamen)(lh.caudalanteriorcingulate_2)(lh.caudalmiddlefrontal_3)(lh.caudalmiddlefrontal_5)
5752	0.83333	(Left-Putamen)(lh.caudalmiddlefrontal_3)(lh.superiorfrontal_11)(lh.superiorfrontal_12)
5753	0.83333	(Left-Putamen)(lh.lateralorbitofrontal_4)(lh.medialorbitofrontal_2)
5754	0.83333	(Left-Putamen)(lh.medialorbitofrontal_2)(lh.rostralanteriorcingulate_1)(lh.rostralmiddlefrontal_12)(lh.rostralmiddlefrontal_9)(lh.superiorfrontal_3)
5755	0.83333	(Left-Putamen)(lh.rostralmiddlefrontal_1)(lh.superiorfrontal_4)
5756	0.83333	(Left-Putamen)(lh.rostralmiddlefrontal_4)(lh.rostralmiddlefrontal_9)(lh.superiorfrontal_3)
5757	0.83333	(Left-Putamen)(lh.superiorparietal_3)(lh.supramarginal_1)
5758	0.83333	(Left-Putamen)(lh.supramarginal_1)(lh.supramarginal_9)
5759	0.83333	(lh.bankssts_2)(lh.inferiortemporal_6)(lh.inferiortemporal_8)
5760	0.83333	(lh.caudalanteriorcingulate_2)(lh.superiorfrontal_10)(lh.superiorfrontal_14)(lh.superiorfrontal_8)
5761	0.83333	(lh.caudalmiddlefrontal_2)(lh.rostralmiddlefrontal_3)
5762	0.83333	(lh.caudalmiddlefrontal_3)(lh.parstriangularis_3)(lh.rostralmiddlefrontal_4)
5763	0.83333	(lh.cuneus_1)(lh.pericalcarine_1)(lh.pericalcarine_3)(lh.superiorparietal_12)
5764	0.83333	(lh.fusiform_1)(lh.fusiform_3)(lh.fusiform_5)(lh.lateraloccipital_9)
5765	0.83333	(lh.fusiform_2)(lh.lateraloccipital_11)(lh.lingual_1)(lh.pericalcarine_3)
5766	0.83333	(lh.fusiform_7)(lh.inferiortemporal_3)(lh.middletemporal_3)
5767	0.83333	(lh.fusiform_7)(lh.middletemporal_5)
5768	0.83333	(lh.inferiortemporal_8)(lh.middletemporal_3)
5769	0.83333	(lh.lateraloccipital_2)(lh.pericalcarine_1)(lh.pericalcarine_2)(lh.pericalcarine_3)
5770	0.83333	(lh.lateralorbitofrontal_2)(lh.lateralorbitofrontal_7)(lh.medialorbitofrontal_4)
5771	0.83333	(lh.lateralorbitofrontal_4)(lh.rostralmiddlefrontal_6)(lh.rostralmiddlefrontal_9)
5772	0.83333	(lh.lateralorbitofrontal_7)(lh.medialorbitofrontal_1)(lh.medialorbitofrontal_3)
5773	0.83333	(lh.medialorbitofrontal_1)(lh.rostralmiddlefrontal_12)(lh.superiorfrontal_1)
5774	0.83333	(lh.paracentral_4)(lh.paracentral_5)(lh.superiorfrontal_14)
5775	0.83333	(lh.parsopercularis_4)(lh.parstriangularis_3)
5776	0.83333	(lh.parstriangularis_3)(lh.rostralmiddlefrontal_12)(lh.rostralmiddlefrontal_4)(lh.rostralmiddlefrontal_9)
5777	0.83333	(lh.posteriorcingulate_1)(lh.posteriorcingulate_2)(lh.posteriorcingulate_3)
5778	0.83333	(lh.precentral_11)(lh.precentral_16)
5779	0.83333	(lh.precuneus_9)(lh.superiorparietal_5)(lh.superiorparietal_6)
5780	0.83333	(lh.rostralanteriorcingulate_1)(lh.rostralmiddlefrontal_8)(lh.superiorfrontal_3)(lh.superiorfrontal_4)
5781	0.83333	(lh.rostralmiddlefrontal_1)(lh.rostralmiddlefrontal_9)(lh.superiorfrontal_4)
5782	0.83333	(lh.superiorfrontal_10)(lh.superiorfrontal_11)(lh.superiorfrontal_4)(lh.superiorfrontal_6)(lh.superiorfrontal_8)
5783	0.83333	(lh.superiorfrontal_10)(lh.superiorfrontal_11)(lh.superiorfrontal_6)(lh.superiorfrontal_8)
5784	0.83333	(lh.superiorfrontal_12)(lh.superiorfrontal_14)(lh.superiorfrontal_4)
5785	0.83333	(lh.superiorfrontal_3)(lh.superiorfrontal_4)(lh.superiorfrontal_5)(lh.superiorfrontal_7)
5786	0.83333	(lh.superiorfrontal_3)(lh.superiorfrontal_5)(lh.superiorfrontal_7)
5787	0.83333	(rh.bankssts_1)(rh.fusiform_7)
5788	0.83333	(rh.cuneus_4)(rh.superiorparietal_13)
5789	0.83333	(rh.fusiform_1)(rh.lateraloccipital_7)(rh.pericalcarine_3)
5790	0.83333	(rh.inferiorparietal_4)(rh.inferiorparietal_5)
5791	0.83333	(rh.inferiortemporal_3)(rh.inferiortemporal_4)(rh.middletemporal_7)
5792	0.83333	(rh.inferiortemporal_4)(rh.middletemporal_4)(rh.middletemporal_6)(rh.middletemporal_7)
5793	0.83333	(rh.insula_3)(rh.precentral_4)
5794	0.83333	(rh.lateraloccipital_3)(rh.lateraloccipital_4)(rh.lateraloccipital_7)
5795	0.83333	(rh.lateraloccipital_8)(rh.precuneus_2)
5796	0.83333	(rh.lateralorbitofrontal_4)(rh.medialorbitofrontal_2)
5797	0.83333	(rh.lateralorbitofrontal_5)(rh.lateralorbitofrontal_6)(rh.lateralorbitofrontal_7)
5798	0.83333	(rh.lateralorbitofrontal_6)(rh.parsorbitalis_2)(rh.parstriangularis_1)(rh.rostralmiddlefrontal_12)
5799	0.83333	(rh.paracentral_4)(rh.paracentral_6)(rh.precentral_15)
5800	0.83333	(rh.parsopercularis_2)(rh.parsopercularis_4)(rh.parstriangularis_1)

5801	0.83333	(rh.parsopercularis_4)(rh.rostralmiddlefrontal_1)
5802	0.83333	(rh.parsorbitalis_1)(rh.parstriangularis_1)(rh.parstriangularis_3)
5803	0.83333	(rh.parstriangularis_1)(rh.rostralmiddlefrontal_13)
5804	0.83333	(rh.postcentral_1)(rh.supramarginal_8)
5805	0.83333	(rh.postcentral_4)(rh.precentral_4)
5806	0.83333	(rh.postcentral_9)(rh.precentral_13)
5807	0.83333	(rh.precentral_5)(rh.precentral_7)(rh.precentral_8)
5808	0.83333	(rh.precuneus_10)(rh.supramarginal_9)
5809	0.83333	(rh.precuneus_4)(rh.precuneus_7)(rh.superiorparietal_11)
5810	0.83333	(rh.precuneus_7)(rh.precuneus_8)(rh.superiorparietal_8)
5811	0.83333	(rh.rostralmiddlefrontal_11)(rh.rostralmiddlefrontal_12)(rh.rostralmiddlefrontal_8)(rh.superiorfrontal_2)
5812	0.83333	(rh.rostralmiddlefrontal_3)(rh.rostralmiddlefrontal_7)
5813	0.83333	(rh.rostralmiddlefrontal_6)(rh.rostralmiddlefrontal_7)(rh.rostralmiddlefrontal_8)
5814	0.83333	(rh.superiorfrontal_2)(rh.superiorfrontal_5)(rh.superiorfrontal_6)(rh.superiorfrontal_9)
5815	0.83333	(rh.superiorfrontal_2)(rh.superiorfrontal_5)(rh.superiorfrontal_8)(rh.superiorfrontal_9)
5816	0.83333	(rh.superiorparietal_1)(rh.superiorparietal_2)(rh.superiorparietal_7)
5817	0.83333	(Right-Caudate)(rh.lateralorbitofrontal_6)(rh.lateralorbitofrontal_7)(rh.medialorbitofrontal_2)
5818	0.83333	(Right-Caudate)(rh.lateralorbitofrontal_7)(rh.medialorbitofrontal_2)(rh.rostralmiddlefrontal_12)
5819	0.83333	(Right-Caudate)(rh.paracentral_4)(rh.precentral_13)
5820	0.83333	(Right-Caudate)(rh.precentral_10)(rh.precentral_7)
5821	0.83333	(Right-Caudate)(Right-Pallidum)(Right-Putamen)(Right-Thalamus-Proper)(rh.insula_1)
5822	0.83333	(Right-Caudate)(Right-Pallidum)(Right-Putamen)(Right-Thalamus-Proper)(rh.posteriorcingulate_2)
5823	0.83333	(Right-Caudate)(Right-Pallidum)(Right-Thalamus-Proper)(rh.insula_1)
5824	0.83333	(Right-Caudate)(Right-Putamen)(rh.insula_1)(rh.precentral_11)(rh.precentral_7)
5825	0.83333	(Right-Caudate)(Right-Putamen)(rh.lateralorbitofrontal_7)(rh.medialorbitofrontal_2)(rh.rostralmiddlefrontal_12)
5826	0.83333	(Right-Pallidum)(Right-Putamen)(Right-Thalamus-Proper)(rh.posteriorcingulate_2)
5827	0.83333	(Right-Putamen)(rh.insula_3)(rh.precentral_13)
5828	0.83333	(Right-Putamen)(rh.lateralorbitofrontal_6)(rh.rostralmiddlefrontal_3)
5829	0.83333	(Right-Putamen)(rh.parstriangularis_1)(rh.rostralanteriorcingulate_2)
5830	0.83333	(Right-Putamen)(rh.precentral_7)(rh.precentral_8)
5831	0.83092	(Left-Caudate)(Left-Pallidum)(Left-Putamen)(Left-Thalamus-Proper)(lh.postcentral_8)
5832	0.83092	(Left-Caudate)(Left-Pallidum)(Left-Thalamus-Proper)(lh.postcentral_8)
5833	0.83092	(Left-Caudate)(Left-Putamen)(lh.caudalanteriorcingulate_2)(lh.superiorfrontal_11)(lh.superiorfrontal_4)
5834	0.83092	(Left-Caudate)(Left-Putamen)(lh.caudalmiddlefrontal_1)(lh.superiorfrontal_11)
5835	0.83092	(Left-Caudate)(Left-Putamen)(lh.caudalmiddlefrontal_3)(lh.insula_6)(lh.parstriangularis_3)
5836	0.83092	(Left-Caudate)(Left-Putamen)(lh.lateralorbitofrontal_1)
5837	0.83092	(Left-Caudate)(Left-Putamen)(lh.lateralorbitofrontal_4)(lh.lateralorbitofrontal_7)(lh.rostralmiddlefrontal_12)
5838	0.83092	(Left-Caudate)(Left-Putamen)(lh.lateralorbitofrontal_7)(lh.parstriangularis_3)(lh.rostralmiddlefrontal_12)(lh.rostralmiddlefrontal_9)
5839	0.83092	(Left-Caudate)(Left-Putamen)(lh.parstriangularis_3)(lh.rostralanteriorcingulate_1)(lh.rostralanteriorcingulate_2)
5840	0.83092	(Left-Caudate)(Left-Putamen)(lh.rostralmiddlefrontal_8)(lh.rostralmiddlefrontal_9)
5841	0.83092	(Left-Caudate)(Left-Thalamus-Proper)(lh.isthmuscingulate_2)
5842	0.83092	(Left-Caudate)(lh.caudalmiddlefrontal_1)(lh.rostralmiddlefrontal_1)(lh.rostralmiddlefrontal_4)
5843	0.83092	(Left-Caudate)(lh.caudalmiddlefrontal_4)(lh.caudalmiddlefrontal_6)(lh.superiorfrontal_12)
5844	0.83092	(Left-Caudate)(lh.insula_6)(lh.parsopercularis_3)(lh.parstriangularis_3)(lh.rostralmiddlefrontal_1)
5845	0.83092	(Left-Caudate)(lh.insula_6)(lh.rostralanteriorcingulate_2)
5846	0.83092	(Left-Caudate)(lh.lateralorbitofrontal_1)
5847	0.83092	(Left-Caudate)(lh.lateralorbitofrontal_2)(lh.medialorbitofrontal_4)
5848	0.83092	(Left-Caudate)(lh.lateralorbitofrontal_4)(lh.lateralorbitofrontal_6)(lh.lateralorbitofrontal_7)
5849	0.83092	(Left-Caudate)(lh.lateralorbitofrontal_4)(lh.rostralanteriorcingulate_1)(lh.rostralmiddlefrontal_9)
5850	0.83092	(Left-Caudate)(lh.lateralorbitofrontal_6)(lh.lateralorbitofrontal_7)(lh.parstriangularis_3)(lh.rostralmiddlefrontal_9)

5851	0.83092	(Left-Caudate)(lh.lateralorbitofrontal_7)(lh.medialorbitofrontal_4)(lh.rostralanteriorcingulate_1)
5852	0.83092	(Left-Caudate)(lh.lateralorbitofrontal_7)(lh.parstriangularis_3)(lh.rostralanteriorcingulate_1)(lh.rostralmiddlefrontal_12)
5853	0.83092	(Left-Caudate)(lh.medialorbitofrontal_2)(lh.rostralmiddlefrontal_12)(lh.superiorfrontal_3)
5854	0.83092	(Left-Caudate)(lh.paracentral_5)(lh.posteriorcingulate_3)(lh.precentral_4)
5855	0.83092	(Left-Caudate)(lh.posteriorcingulate_1)(lh.posteriorcingulate_2)(lh.posteriorcingulate_3)
5856	0.83092	(Left-Caudate)(lh.rostralmiddlefrontal_1)(lh.rostralmiddlefrontal_4)(lh.superiorfrontal_4)
5857	0.83092	(Left-Hippocampus)(lh.entorhinal_1)(lh.insula_4)
5858	0.83092	(Left-Hippocampus)(lh.inferiorparietal_5)(lh.lingual_8)
5859	0.83092	(Left-Putamen)(Left-Thalamus-Proper)(lh.precentral_14)
5860	0.83092	(Left-Putamen)(lh.caudalmiddlefrontal_1)(lh.caudalmiddlefrontal_3)(lh.rostralmiddlefrontal_1)
5861	0.83092	(Left-Putamen)(lh.caudalmiddlefrontal_3)(lh.parsopercularis_3)(lh.rostralmiddlefrontal_1)
5862	0.83092	(Left-Putamen)(lh.caudalmiddlefrontal_5)(lh.superiorfrontal_12)
5863	0.83092	(Left-Putamen)(lh.insula_6)(lh.rostralanteriorcingulate_2)
5864	0.83092	(Left-Putamen)(lh.lateralorbitofrontal_7)(lh.rostralmiddlefrontal_1)
5865	0.83092	(Left-Putamen)(lh.posteriorcingulate_1)(lh.superiorfrontal_12)
5866	0.83092	(Left-Putamen)(lh.precentral_4)(lh.precentral_9)
5867	0.83092	(Left-Putamen)(lh.superiorfrontal_14)(lh.superiorfrontal_4)
5868	0.83092	(lh.cuneus_1)(lh.pericalcarine_3)(lh.precuneus_9)
5869	0.83092	(lh.cuneus_2)(lh.pericalcarine_1)(lh.superiorparietal_12)
5870	0.83092	(lh.fusiform_1)(lh.fusiform_2)(lh.fusiform_5)(lh.lateraloccipital_9)
5871	0.83092	(lh.fusiform_2)(lh.lateraloccipital_6)(lh.lateraloccipital_7)(lh.lateraloccipital_9)
5872	0.83092	(lh.fusiform_2)(lh.lingual_6)(lh.lingual_8)(lh.pericalcarine_3)
5873	0.83092	(lh.fusiform_5)(lh.inferiorparietal_4)(lh.lingual_8)
5874	0.83092	(lh.fusiform_8)(lh.insula_4)
5875	0.83092	(lh.insula_6)(lh.precentral_16)
5876	0.83092	(lh.lateraloccipital_3)(lh.lateraloccipital_6)(lh.lingual_1)(lh.pericalcarine_2)
5877	0.83092	(lh.lateraloccipital_6)(lh.lateraloccipital_7)(lh.lingual_8)
5878	0.83092	(lh.lateraloccipital_6)(lh.lingual_1)(lh.lingual_3)
5879	0.83092	(lh.lateraloccipital_6)(lh.lingual_4)(lh.lingual_8)
5880	0.83092	(lh.lateralorbitofrontal_4)(lh.lateralorbitofrontal_6)(lh.parstriangularis_1)(lh.parstriangularis_3)
5881	0.83092	(lh.lateralorbitofrontal_4)(lh.parstriangularis_3)(lh.rostralanteriorcingulate_1)(lh.rostralmiddlefrontal_12)
5882	0.83092	(lh.lateralorbitofrontal_6)(lh.lateralorbitofrontal_7)(lh.medialorbitofrontal_2)(lh.rostralmiddlefrontal_12)
5883	0.83092	(lh.lateralorbitofrontal_6)(lh.lateralorbitofrontal_7)(lh.rostralmiddlefrontal_12)(lh.rostralmiddlefrontal_6)(lh.rostralmiddlefrontal_9)
5884	0.83092	(lh.lateralorbitofrontal_6)(lh.medialorbitofrontal_2)(lh.rostralmiddlefrontal_12)(lh.rostralmiddlefrontal_9)
5885	0.83092	(lh.lateralorbitofrontal_7)(lh.medialorbitofrontal_2)(lh.rostralmiddlefrontal_9)(lh.superiorfrontal_3)
5886	0.83092	(lh.lateralorbitofrontal_7)(lh.rostralanteriorcingulate_1)(lh.superiorfrontal_3)
5887	0.83092	(lh.lateralorbitofrontal_7)(lh.rostralmiddlefrontal_12)(lh.rostralmiddlefrontal_9)(lh.superiorfrontal_3)
5888	0.83092	(lh.lingual_6)(lh.lingual_8)(lh.precuneus_11)
5889	0.83092	(lh.parsopercularis_1)(lh.parsopercularis_4)(lh.parstriangularis_3)
5890	0.83092	(lh.posteriorcingulate_3)(lh.superiorfrontal_14)(lh.superiorfrontal_8)
5891	0.83092	(lh.precentral_5)(lh.precentral_6)(lh.precentral_7)
5892	0.83092	(lh.precuneus_3)(lh.superiorparietal_9)
5893	0.83092	(lh.rostralanteriorcingulate_1)(lh.superiorfrontal_2)(lh.superiorfrontal_4)
5894	0.83092	(lh.rostralmiddlefrontal_1)(lh.rostralmiddlefrontal_4)(lh.rostralmiddlefrontal_7)(lh.rostralmiddlefrontal_9)
5895	0.83092	(lh.rostralmiddlefrontal_4)(lh.rostralmiddlefrontal_5)(lh.rostralmiddlefrontal_9)
5896	0.83092	(lh.rostralmiddlefrontal_9)(lh.superiorfrontal_1)(lh.superiorfrontal_3)(lh.superiorfrontal_4)
5897	0.83092	(lh.superiorfrontal_10)(lh.superiorfrontal_3)(lh.superiorfrontal_4)(lh.superiorfrontal_7)
5898	0.83092	(lh.superiorfrontal_10)(lh.superiorfrontal_3)(lh.superiorfrontal_7)
5899	0.83092	(lh.superiorfrontal_10)(lh.superiorfrontal_5)(lh.superiorfrontal_6)(lh.superiorfrontal_7)
5900	0.83092	(lh.superiortemporal_2)(lh.superiortemporal_3)

5901	0.83092	(rh.bankssts_2)(rh.fusiform_5)
5902	0.83092	(rh.bankssts_2)(rh.inferiorparietal_10)(rh.inferiorparietal_9)
5903	0.83092	(rh.caudalmiddlefrontal_4)(rh.rostralmiddlefrontal_4)
5904	0.83092	(rh.caudalmiddlefrontal_4)(rh.superiorfrontal_13)(rh.superiorfrontal_7)(rh.superiorfrontal_9)
5905	0.83092	(rh.caudalmiddlefrontal_5)(rh.rostralmiddlefrontal_1)(rh.superiorfrontal_7)
5906	0.83092	(rh.fusiform_8)(rh.superiortemporal_7)
5907	0.83092	(rh.inferiorparietal_10)(rh.lateraloccipital_8)(rh.lingual_5)
5908	0.83092	(rh.inferiortemporal_2)(rh.superiortemporal_6)(rh.superiortemporal_7)
5909	0.83092	(rh.inferiortemporal_3)(rh.middletemporal_5)
5910	0.83092	(rh.lateraloccipital_3)(rh.lateraloccipital_4)(rh.lateraloccipital_5)
5911	0.83092	(rh.lateraloccipital_3)(rh.lateraloccipital_4)(rh.pericalcarine_1)(rh.superiorparietal_11)
5912	0.83092	(rh.lateraloccipital_5)(rh.lateraloccipital_6)(rh.lateraloccipital_7)
5913	0.83092	(rh.lateraloccipital_8)(rh.lingual_2)(rh.lingual_4)
5914	0.83092	(rh.lateralorbitofrontal_1)(rh.lateralorbitofrontal_2)
5915	0.83092	(rh.lateralorbitofrontal_6)(rh.lateralorbitofrontal_7)(rh.parstriangularis_4)(rh.rostralmiddlefrontal_12)
5916	0.83092	(rh.lateralorbitofrontal_6)(rh.parstriangularis_1)(rh.rostralmiddlefrontal_13)
5917	0.83092	(rh.lateralorbitofrontal_7)(rh.rostralmiddlefrontal_11)(rh.rostralmiddlefrontal_12)(rh.superiorfrontal_2)
5918	0.83092	(rh.lingual_5)(rh.lingual_6)(rh.lingual_7)
5919	0.83092	(rh.lingual_5)(rh.pericalcarine_1)
5920	0.83092	(rh.medialorbitofrontal_2)(rh.medialorbitofrontal_3)
5921	0.83092	(rh.parsopercularis_1)(rh.rostralmiddlefrontal_3)
5922	0.83092	(rh.parstriangularis_1)(rh.rostralmiddlefrontal_7)
5923	0.83092	(rh.postcentral_1)(rh.postcentral_3)(rh.precentral_1)
5924	0.83092	(rh.postcentral_9)(rh.supramarginal_1)
5925	0.83092	(rh.precentral_3)(rh.precentral_6)
5926	0.83092	(rh.precuneus_10)(rh.superiorparietal_1)(rh.superiorparietal_7)(rh.superiorparietal_8)
5927	0.83092	(rh.precuneus_5)(rh.precuneus_8)(rh.superiorparietal_8)
5928	0.83092	(rh.precuneus_6)(rh.precuneus_7)
5929	0.83092	(rh.precuneus_7)(rh.superiorparietal_1)(rh.superiorparietal_10)(rh.superiorparietal_7)
5930	0.83092	(rh.rostralanteriorcingulate_1)(rh.rostralmiddlefrontal_8)
5931	0.83092	(rh.rostralmiddlefrontal_1)(rh.rostralmiddlefrontal_3)(rh.rostralmiddlefrontal_8)
5932	0.83092	(rh.superiorfrontal_13)(rh.superiorfrontal_5)(rh.superiorfrontal_8)(rh.superiorfrontal_9)
5933	0.83092	(Right-Amygdala)(rh.temporalpole_1)
5934	0.83092	(Right-Caudate)(rh.caudalmiddlefrontal_4)(rh.superiorfrontal_7)(rh.superiorfrontal_9)
5935	0.83092	(Right-Caudate)(rh.insula_2)(rh.supramarginal_9)
5936	0.83092	(Right-Caudate)(rh.precentral_13)(rh.superiorfrontal_14)
5937	0.83092	(Right-Caudate)(rh.rostralmiddlefrontal_1)(rh.rostralmiddlefrontal_5)
5938	0.83092	(Right-Caudate)(Right-Putamen)(rh.caudalmiddlefrontal_4)(rh.parsopercularis_4)(rh.rostralmiddlefrontal_3)
5939	0.83092	(Right-Caudate)(Right-Putamen)(rh.insula_2)(rh.supramarginal_9)
5940	0.83092	(Right-Caudate)(Right-Putamen)(rh.lateralorbitofrontal_6)(rh.lateralorbitofrontal_7)(rh.medialorbitofrontal_2)
5941	0.83092	(Right-Caudate)(Right-Putamen)(rh.precentral_10)(rh.precentral_7)
5942	0.83092	(Right-Caudate)(Right-Putamen)(rh.precentral_8)
5943	0.83092	(Right-Caudate)(Right-Putamen)(rh.superiorfrontal_5)
5944	0.83092	(Right-Hippocampus)(rh.fusiform_7)(rh.middletemporal_4)
5945	0.83092	(Right-Putamen)(rh.insula_1)(rh.supramarginal_1)(rh.supramarginal_8)(rh.supramarginal_9)
5946	0.83092	(Right-Putamen)(rh.lateralorbitofrontal_5)(rh.lateralorbitofrontal_6)
5947	0.83092	(Right-Putamen)(rh.parsopercularis_2)(rh.precentral_1)
5948	0.8285	(Left-Caudate)(Left-Putamen)(Left-Thalamus-Proper)(lh.postcentral_8)(lh.precentral_4)
5949	0.8285	(Left-Caudate)(Left-Putamen)(lh.caudalanteriorcingulate_1)(lh.posteriorcingulate_1)
5950	0.8285	(Left-Caudate)(Left-Putamen)(lh.caudalmiddlefrontal_1)(lh.rostralmiddlefrontal_1)

5951	0.8285	(Left-Caudate)(Left-Putamen)(lh.caudalmiddlefrontal_3)(lh.parstriangularis_3)(lh.rostralmiddlefrontal_1)
5952	0.8285	(Left-Caudate)(Left-Putamen)(lh.caudalmiddlefrontal_3)(lh.rostralmiddlefrontal_1)(lh.rostralmiddlefrontal_9)
5953	0.8285	(Left-Caudate)(Left-Putamen)(lh.insula_6)(lh.parstriangularis_3)(lh.rostralmiddlefrontal_4)
5954	0.8285	(Left-Caudate)(Left-Putamen)(lh.lateralorbitofrontal_4)(lh.lateralorbitofrontal_6)(lh.lateralorbitofrontal_7)
5955	0.8285	(Left-Caudate)(Left-Putamen)(lh.lateralorbitofrontal_4)(lh.lateralorbitofrontal_6)(lh.parstriangularis_3)
5956	0.8285	(Left-Caudate)(Left-Putamen)(lh.lateralorbitofrontal_4)(lh.rostralanteriorcingulate_1)(lh.rostralmiddlefrontal_9)
5957	0.8285	(Left-Caudate)(Left-Putamen)(lh.posteriorcingulate_1)(lh.superiorfrontal_12)
5958	0.8285	(Left-Caudate)(Left-Putamen)(lh.rostralmiddlefrontal_1)(lh.rostralmiddlefrontal_12)(lh.rostralmiddlefrontal_9)
5959	0.8285	(Left-Caudate)(Left-Putamen)(lh.superiorfrontal_11)(lh.superiorfrontal_12)(lh.superiorfrontal_14)
5960	0.8285	(Left-Caudate)(lh.caudalanteriorcingulate_1)(lh.caudalanteriorcingulate_2)(lh.superiorfrontal_10)
5961	0.8285	(Left-Caudate)(lh.caudalanteriorcingulate_1)(lh.rostralanteriorcingulate_2)(lh.rostralmiddlefrontal_4)
5962	0.8285	(Left-Caudate)(lh.caudalmiddlefrontal_3)(lh.insula_6)(lh.parsopercularis_3)
5963	0.8285	(Left-Caudate)(lh.isthmuscingulate_1)(lh.posteriorcingulate_2)
5964	0.8285	(Left-Caudate)(lh.lateralorbitofrontal_7)(lh.rostralmiddlefrontal_1)
5965	0.8285	(Left-Caudate)(lh.paracentral_5)(lh.superiorfrontal_8)
5966	0.8285	(Left-Caudate)(lh.parstriangularis_3)(lh.rostralanteriorcingulate_1)(lh.rostralmiddlefrontal_4)(lh.rostralmiddlefrontal_9)
5967	0.8285	(Left-Caudate)(lh.rostralanteriorcingulate_1)(lh.rostralanteriorcingulate_2)(lh.rostralmiddlefrontal_4)(lh.superiorfrontal_4)
5968	0.8285	(Left-Putamen)(lh.caudalanteriorcingulate_1)(lh.posteriorcingulate_1)
5969	0.8285	(Left-Putamen)(lh.caudalmiddlefrontal_3)(lh.insula_6)(lh.rostralmiddlefrontal_1)(lh.rostralmiddlefrontal_4)
5970	0.8285	(Left-Putamen)(lh.caudalmiddlefrontal_3)(lh.rostralmiddlefrontal_4)(lh.superiorfrontal_11)
5971	0.8285	(Left-Putamen)(lh.lateralorbitofrontal_6)(lh.rostralmiddlefrontal_1)(lh.rostralmiddlefrontal_12)
5972	0.8285	(Left-Putamen)(lh.medialorbitofrontal_2)(lh.parstriangularis_3)(lh.rostralanteriorcingulate_1)(lh.rostralmiddlefrontal_9)
5973	0.8285	(Left-Putamen)(lh.medialorbitofrontal_2)(lh.rostralmiddlefrontal_9)(lh.superiorfrontal_4)
5974	0.8285	(Left-Thalamus-Proper)(lh.paracentral_5)(lh.postcentral_3)
5975	0.8285	(Left-Thalamus-Proper)(lh.precentral_7)(lh.precentral_9)
5976	0.8285	(lh.caudalanteriorcingulate_1)(lh.rostralanteriorcingulate_2)(lh.superiorfrontal_10)
5977	0.8285	(lh.caudalmiddlefrontal_3)(lh.caudalmiddlefrontal_4)(lh.superiorfrontal_12)
5978	0.8285	(lh.cuneus_1)(lh.lateraloccipital_2)(lh.superiorparietal_12)
5979	0.8285	(lh.cuneus_3)(lh.lateraloccipital_1)(lh.pericalcarine_1)
5980	0.8285	(lh.fusiform_1)(lh.fusiform_2)(lh.fusiform_3)(lh.lateraloccipital_9)
5981	0.8285	(lh.fusiform_1)(lh.fusiform_2)(lh.lingual_6)
5982	0.8285	(lh.fusiform_2)(lh.lateraloccipital_7)(lh.lingual_8)(lh.pericalcarine_3)
5983	0.8285	(lh.inferiorparietal_8)(lh.superiorparietal_11)(lh.superiorparietal_6)
5984	0.8285	(lh.insula_2)(lh.insula_4)
5985	0.8285	(lh.insula_6)(lh.lateralorbitofrontal_6)(lh.parstriangularis_3)(lh.rostralmiddlefrontal_1)
5986	0.8285	(lh.lateraloccipital_10)(lh.lateraloccipital_11)(lh.lateraloccipital_6)
5987	0.8285	(lh.lateraloccipital_9)(lh.middletemporal_1)
5988	0.8285	(lh.lateralorbitofrontal_4)(lh.medialorbitofrontal_2)(lh.rostralmiddlefrontal_9)
5989	0.8285	(lh.lateralorbitofrontal_4)(lh.parstriangularis_3)(lh.rostralmiddlefrontal_6)
5990	0.8285	(lh.lateralorbitofrontal_6)(lh.lateralorbitofrontal_7)(lh.rostralmiddlefrontal_1)
5991	0.8285	(lh.lateralorbitofrontal_6)(lh.parsorbitalis_1)(lh.parstriangularis_3)(lh.rostralmiddlefrontal_9)
5992	0.8285	(lh.lateralorbitofrontal_7)(lh.rostralmiddlefrontal_1)(lh.rostralmiddlefrontal_9)
5993	0.8285	(lh.medialorbitofrontal_2)(lh.rostralanteriorcingulate_1)(lh.superiorfrontal_1)
5994	0.8285	(lh.paracentral_4)(lh.superiorfrontal_15)(lh.superiorfrontal_16)(lh.superiorfrontal_18)
5995	0.8285	(lh.parsopercularis_1)(lh.parsopercularis_2)(lh.parsopercularis_3)(lh.parstriangularis_3)
5996	0.8285	(lh.precentral_11)(lh.precentral_13)(lh.precentral_9)
5997	0.8285	(lh.precentral_12)(lh.precentral_13)
5998	0.8285	(lh.precuneus_5)(lh.superiorparietal_9)
5999	0.8285	(lh.rostralanteriorcingulate_1)(lh.rostralmiddlefrontal_4)(lh.rostralmiddlefrontal_9)(lh.superiorfrontal_3)
6000	0.8285	(lh.rostralanteriorcingulate_2)(lh.rostralmiddlefrontal_4)(lh.rostralmiddlefrontal_8)(lh.superiorfrontal_4)

6001	0.8285	(lh.superiorfrontal_10)(lh.superiorfrontal_11)(lh.superiorfrontal_14)(lh.superiorfrontal_4)(lh.superiorfrontal_8)
6002	0.8285	(lh.superiorfrontal_10)(lh.superiorfrontal_11)(lh.superiorfrontal_14)(lh.superiorfrontal_4)
6003	0.8285	(lh.superiorfrontal_10)(lh.superiorfrontal_11)(lh.superiorfrontal_4)(lh.superiorfrontal_9)
6004	0.8285	(lh.superiorfrontal_10)(lh.superiorfrontal_12)
6005	0.8285	(lh.superiorfrontal_10)(lh.superiorfrontal_4)(lh.superiorfrontal_5)(lh.superiorfrontal_6)(lh.superiorfrontal_7)
6006	0.8285	(lh.superiorfrontal_12)(lh.superiorfrontal_14)(lh.superiorfrontal_4)(lh.superiorfrontal_8)
6007	0.8285	(lh.supramarginal_5)(lh.supramarginal_6)
6008	0.8285	(rh.caudalmiddlefrontal_5)(rh.parsopercularis_4)
6009	0.8285	(rh.caudalmiddlefrontal_5)(rh.precentral_13)
6010	0.8285	(rh.fusiform_1)(rh.fusiform_2)(rh.fusiform_3)(rh.lateraloccipital_8)
6011	0.8285	(rh.fusiform_4)(rh.fusiform_5)
6012	0.8285	(rh.inferiorparietal_10)(rh.lateraloccipital_10)(rh.lingual_5)
6013	0.8285	(rh.inferiortemporal_4)(rh.inferiortemporal_5)(rh.inferiortemporal_6)
6014	0.8285	(rh.inferiortemporal_4)(rh.middletemporal_2)
6015	0.8285	(rh.insula_1)(rh.superiorparietal_1)(rh.supramarginal_1)(rh.supramarginal_8)(rh.supramarginal_9)
6016	0.8285	(rh.lateraloccipital_1)(rh.lateraloccipital_3)(rh.pericalcarine_2)(rh.superiorparietal_11)
6017	0.8285	(rh.lateraloccipital_1)(rh.lateraloccipital_4)(rh.pericalcarine_2)
6018	0.8285	(rh.lateraloccipital_3)(rh.pericalcarine_2)(rh.pericalcarine_4)
6019	0.8285	(rh.lateraloccipital_4)(rh.lateraloccipital_8)(rh.lingual_2)(rh.pericalcarine_1)
6020	0.8285	(rh.lateralorbitofrontal_2)(rh.lateralorbitofrontal_6)(rh.rostralmiddlefrontal_8)
6021	0.8285	(rh.lateralorbitofrontal_6)(rh.lateralorbitofrontal_7)(rh.rostralmiddlefrontal_12)(rh.rostralmiddlefrontal_8)
6022	0.8285	(rh.lateralorbitofrontal_6)(rh.parstriangularis_4)(rh.rostralmiddlefrontal_3)
6023	0.8285	(rh.middletemporal_2)(rh.middletemporal_4)
6024	0.8285	(rh.paracentral_4)(rh.superiorfrontal_14)(rh.superiorfrontal_17)
6025	0.8285	(rh.parsorbitalis_2)(rh.parstriangularis_3)
6026	0.8285	(rh.pericalcarine_3)(rh.pericalcarine_4)
6027	0.8285	(rh.postcentral_10)(rh.posteriorcingulate_4)
6028	0.8285	(rh.postcentral_10)(rh.superiorparietal_1)(rh.supramarginal_1)
6029	0.8285	(rh.precentral_10)(rh.precentral_11)
6030	0.8285	(rh.precentral_14)(rh.superiorfrontal_16)
6031	0.8285	(rh.precuneus_7)(rh.superiorparietal_1)(rh.superiorparietal_8)
6032	0.8285	(rh.rostralmiddlefrontal_1)(rh.superiorfrontal_2)
6033	0.8285	(rh.superiorfrontal_10)(rh.superiorfrontal_13)(rh.superiorfrontal_8)(rh.superiorfrontal_9)
6034	0.8285	(rh.superiorfrontal_11)(rh.superiorfrontal_14)(rh.superiorfrontal_16)
6035	0.8285	(rh.superiorfrontal_12)(rh.superiorfrontal_17)
6036	0.8285	(rh.superiorfrontal_13)(rh.superiorfrontal_5)(rh.superiorfrontal_7)
6037	0.8285	(Right-Caudate)(rh.caudalmiddlefrontal_4)(rh.caudalmiddlefrontal_5)(rh.rostralmiddlefrontal_1)
6038	0.8285	(Right-Caudate)(rh.caudalmiddlefrontal_4)(rh.caudalmiddlefrontal_5)(rh.superiorfrontal_7)
6039	0.8285	(Right-Caudate)(rh.insula_7)(rh.lateralorbitofrontal_2)
6040	0.8285	(Right-Caudate)(rh.lateralorbitofrontal_2)(rh.rostralmiddlefrontal_12)
6041	0.8285	(Right-Caudate)(rh.lateralorbitofrontal_7)(rh.rostralmiddlefrontal_8)
6042	0.8285	(Right-Caudate)(rh.precentral_13)(rh.superiorfrontal_13)
6043	0.8285	(Right-Caudate)(rh.rostralanteriorcingulate_2)(rh.rostralmiddlefrontal_8)
6044	0.8285	(Right-Caudate)(Right-Putamen)(rh.insula_7)(rh.lateralorbitofrontal_2)
6045	0.8285	(Right-Caudate)(Right-Putamen)(rh.lateralorbitofrontal_2)(rh.rostralmiddlefrontal_12)
6046	0.8285	(Right-Hippocampus)(rh.insula_1)(rh.insula_2)(rh.supramarginal_9)
6047	0.8285	(Right-Hippocampus)(Right-Putamen)(rh.insula_1)(rh.insula_2)(rh.supramarginal_9)
6048	0.8285	(Right-Putamen)(rh.caudalmiddlefrontal_4)(rh.precentral_10)
6049	0.8285	(Right-Putamen)(rh.lateralorbitofrontal_2)(rh.lateralorbitofrontal_6)(rh.rostralmiddlefrontal_8)
6050	0.8285	(Right-Putamen)(rh.lateralorbitofrontal_4)(rh.medialorbitofrontal_2)

6051	0.8285	(Right-Putamen)(rh.lateralorbitofrontal_6)(rh.lateralorbitofrontal_7)(rh.rostralmiddlefrontal_8)
6052	0.8285	(Right-Putamen)(rh.rostralmiddlefrontal_1)(rh.superiorfrontal_7)
6053	0.82609	(Left-Accumbens-area)(Left-Amygdala)(Left-Hippocampus)(Left-Pallidum)(Left-Thalamus-Proper)
6054	0.82609	(Left-Accumbens-area)(Left-Amygdala)(Left-Hippocampus)(Left-Pallidum)
6055	0.82609	(Left-Accumbens-area)(Left-Amygdala)(Left-Hippocampus)(Left-Putamen)(Left-Thalamus-Proper)
6056	0.82609	(Left-Accumbens-area)(Left-Amygdala)(Left-Hippocampus)(Left-Putamen)
6057	0.82609	(Left-Amygdala)(Left-Hippocampus)(lh.superiortemporal_11)
6058	0.82609	(Left-Amygdala)(lh.entorhinal_1)(lh.insula_4)
6059	0.82609	(Left-Caudate)(Left-Putamen)(Left-Thalamus-Proper)(lh.paracentral_5)
6060	0.82609	(Left-Caudate)(Left-Putamen)(lh.caudalanteriorcingulate_2)(lh.caudalmiddlefrontal_3)(lh.caudalmiddlefrontal_5)
6061	0.82609	(Left-Caudate)(Left-Putamen)(lh.caudalanteriorcingulate_2)(lh.superiorfrontal_10)
6062	0.82609	(Left-Caudate)(Left-Putamen)(lh.caudalmiddlefrontal_3)(lh.insula_6)(lh.parsopercularis_3)
6063	0.82609	(Left-Caudate)(Left-Putamen)(lh.caudalmiddlefrontal_3)(lh.superiorfrontal_11)(lh.superiorfrontal_12)
6064	0.82609	(Left-Caudate)(Left-Putamen)(lh.lateralorbitofrontal_6)(lh.parstriangularis_3)(lh.rostralmiddlefrontal_12)
6065	0.82609	(Left-Caudate)(Left-Putamen)(lh.parstriangularis_3)(lh.rostralanteriorcingulate_1)(lh.rostralmiddlefrontal_4)
6066	0.82609	(Left-Caudate)(Left-Putamen)(lh.posteriorcingulate_2)
6067	0.82609	(Left-Caudate)(Left-Putamen)(lh.rostralmiddlefrontal_1)(lh.superiorfrontal_4)
6068	0.82609	(Left-Caudate)(Left-Thalamus-Proper)(lh.caudalmiddlefrontal_4)
6069	0.82609	(Left-Caudate)(lh.caudalmiddlefrontal_3)(lh.caudalmiddlefrontal_6)
6070	0.82609	(Left-Caudate)(lh.caudalmiddlefrontal_6)(lh.posteriorcingulate_1)
6071	0.82609	(Left-Caudate)(lh.insula_6)(lh.rostralmiddlefrontal_4)(lh.rostralmiddlefrontal_9)
6072	0.82609	(Left-Caudate)(lh.postcentral_8)(lh.precentral_11)(lh.precentral_7)
6073	0.82609	(Left-Caudate)(lh.rostralanteriorcingulate_1)(lh.rostralanteriorcingulate_2)(lh.rostralmiddlefrontal_9)(lh.superiorfrontal_4)
6074	0.82609	(Left-Caudate)(lh.rostralanteriorcingulate_1)(lh.rostralmiddlefrontal_12)(lh.superiorfrontal_4)
6075	0.82609	(Left-Caudate)(lh.rostralmiddlefrontal_4)(lh.rostralmiddlefrontal_8)(lh.superiorfrontal_4)
6076	0.82609	(Left-Caudate)(lh.superiorfrontal_11)(lh.superiorfrontal_14)(lh.superiorfrontal_4)(lh.superiorfrontal_8)
6077	0.82609	(Left-Hippocampus)(Left-Putamen)(Left-Thalamus-Proper)(lh.transversetemporal_2)
6078	0.82609	(Left-Hippocampus)(Left-Putamen)(lh.insula_1)(lh.supramarginal_1)
6079	0.82609	(Left-Putamen)(lh.caudalanteriorcingulate_2)(lh.posteriorcingulate_3)(lh.superiorfrontal_14)
6080	0.82609	(Left-Putamen)(lh.caudalmiddlefrontal_3)(lh.insula_6)(lh.parstriangularis_3)(lh.rostralmiddlefrontal_1)
6081	0.82609	(Left-Putamen)(lh.lateralorbitofrontal_6)(lh.medialorbitofrontal_2)(lh.rostralmiddlefrontal_9)
6082	0.82609	(Left-Putamen)(lh.posteriorcingulate_2)
6083	0.82609	(Left-Putamen)(lh.precentral_11)(lh.precentral_4)(lh.precentral_9)
6084	0.82609	(Left-Putamen)(lh.precentral_4)(lh.superiorfrontal_12)
6085	0.82609	(Left-Putamen)(lh.precentral_5)(lh.precentral_7)
6086	0.82609	(Left-Thalamus-Proper)(lh.caudalmiddlefrontal_4)
6087	0.82609	(lh.caudalanteriorcingulate_2)(lh.superiorfrontal_11)(lh.superiorfrontal_14)(lh.superiorfrontal_4)
6088	0.82609	(lh.caudalmiddlefrontal_3)(lh.caudalmiddlefrontal_6)
6089	0.82609	(lh.caudalmiddlefrontal_3)(lh.parsopercularis_1)(lh.rostralmiddlefrontal_1)
6090	0.82609	(lh.caudalmiddlefrontal_3)(lh.parstriangularis_3)(lh.rostralmiddlefrontal_1)(lh.rostralmiddlefrontal_4)
6091	0.82609	(lh.caudalmiddlefrontal_6)(lh.posteriorcingulate_1)
6092	0.82609	(lh.cuneus_1)(lh.lateraloccipital_2)(lh.pericalcarine_1)
6093	0.82609	(lh.fusiform_1)(lh.fusiform_5)(lh.inferiortemporal_8)(lh.lateraloccipital_9)
6094	0.82609	(lh.inferiorparietal_1)(lh.inferiorparietal_4)(lh.lateraloccipital_7)
6095	0.82609	(lh.inferiorparietal_10)(lh.supramarginal_10)
6096	0.82609	(lh.inferiortemporal_2)(lh.middletemporal_4)
6097	0.82609	(lh.inferiortemporal_5)(lh.inferiortemporal_6)(lh.middletemporal_3)
6098	0.82609	(lh.lateraloccipital_2)(lh.pericalcarine_2)(lh.superiorparietal_12)
6099	0.82609	(lh.lateraloccipital_6)(lh.lingual_3)(lh.pericalcarine_3)
6100	0.82609	(lh.lateralorbitofrontal_3)(lh.lateralorbitofrontal_7)

6101	0.82609	(lh.lateralorbitofrontal_4)(lh.parsorbitalis_1)
6102	0.82609	(lh.lateralorbitofrontal_6)(lh.rostralmiddlefrontal_10)(lh.rostralmiddlefrontal_6)
6103	0.82609	(lh.lateralorbitofrontal_7)(lh.parstriangularis_3)(lh.rostralmiddlefrontal_12)(lh.rostralmiddlefrontal_6)(lh.rostralmiddlefrontal_9)
6104	0.82609	(lh.lingual_4)(lh.lingual_6)
6105	0.82609	(lh.paracentral_5)(lh.superiorfrontal_12)(lh.superiorfrontal_8)
6106	0.82609	(lh.parsorbitalis_1)(lh.rostralmiddlefrontal_12)
6107	0.82609	(lh.rostralanteriorcingulate_1)(lh.superiorfrontal_2)(lh.superiorfrontal_3)
6108	0.82609	(lh.rostralanteriorcingulate_2)(lh.rostralmiddlefrontal_4)(lh.rostralmiddlefrontal_9)(lh.superiorfrontal_4)
6109	0.82609	(lh.rostralanteriorcingulate_2)(lh.rostralmiddlefrontal_4)(lh.superiorfrontal_10)(lh.superiorfrontal_4)
6110	0.82609	(lh.rostralmiddlefrontal_12)(lh.rostralmiddlefrontal_8)
6111	0.82609	(lh.superiorfrontal_10)(lh.superiorfrontal_12)(lh.superiorfrontal_8)
6112	0.82609	(lh.superiorfrontal_10)(lh.superiorfrontal_14)(lh.superiorfrontal_8)(lh.superiorfrontal_9)
6113	0.82609	(lh.superiorfrontal_10)(lh.superiorfrontal_4)(lh.superiorfrontal_8)(lh.superiorfrontal_9)
6114	0.82609	(rh.bankssts_1)(rh.inferiortemporal_5)(rh.middletemporal_4)
6115	0.82609	(rh.bankssts_2)(rh.inferiortemporal_5)
6116	0.82609	(rh.caudalanteriorcingulate_1)(rh.rostralanteriorcingulate_1)
6117	0.82609	(rh.caudalanteriorcingulate_2)(rh.parsopercularis_4)
6118	0.82609	(rh.caudalmiddlefrontal_1)(rh.superiorfrontal_13)
6119	0.82609	(rh.cuneus_3)(rh.cuneus_4)
6120	0.82609	(rh.cuneus_3)(rh.pericalcarine_2)(rh.superiorparietal_11)
6121	0.82609	(rh.fusiform_2)(rh.fusiform_4)(rh.lateraloccipital_10)
6122	0.82609	(rh.fusiform_2)(rh.inferiorparietal_10)(rh.lingual_5)
6123	0.82609	(rh.fusiform_2)(rh.lateraloccipital_10)(rh.lateraloccipital_8)(rh.lingual_5)
6124	0.82609	(rh.fusiform_8)(rh.superiortemporal_3)
6125	0.82609	(rh.insula_1)(rh.supramarginal_7)(rh.supramarginal_8)
6126	0.82609	(rh.lateraloccipital_2)(rh.lateraloccipital_4)
6127	0.82609	(rh.lateralorbitofrontal_6)(rh.parstriangularis_1)(rh.parstriangularis_3)(rh.rostralmiddlefrontal_12)
6128	0.82609	(rh.lateralorbitofrontal_6)(rh.parstriangularis_1)(rh.parstriangularis_4)(rh.rostralmiddlefrontal_8)
6129	0.82609	(rh.lateralorbitofrontal_6)(rh.parstriangularis_4)(rh.rostralmiddlefrontal_12)(rh.rostralmiddlefrontal_8)
6130	0.82609	(rh.lateralorbitofrontal_7)(rh.parstriangularis_1)(rh.parstriangularis_4)
6131	0.82609	(rh.medialorbitofrontal_2)(rh.rostralmiddlefrontal_11)(rh.rostralmiddlefrontal_12)(rh.superiorfrontal_2)
6132	0.82609	(rh.parsopercularis_1)(rh.parsopercularis_2)(rh.parsopercularis_4)
6133	0.82609	(rh.parsorbitalis_2)(rh.parstriangularis_4)
6134	0.82609	(rh.parsorbitalis_2)(rh.rostralmiddlefrontal_12)(rh.rostralmiddlefrontal_13)
6135	0.82609	(rh.parstriangularis_1)(rh.rostralmiddlefrontal_12)(rh.rostralmiddlefrontal_5)
6136	0.82609	(rh.parstriangularis_2)(rh.rostralmiddlefrontal_3)(rh.rostralmiddlefrontal_8)
6137	0.82609	(rh.postcentral_1)(rh.postcentral_2)(rh.postcentral_4)
6138	0.82609	(rh.postcentral_1)(rh.postcentral_4)(rh.precentral_6)
6139	0.82609	(rh.postcentral_1)(rh.precentral_1)(rh.precentral_4)
6140	0.82609	(rh.precuneus_10)(rh.superiorparietal_1)(rh.supramarginal_9)
6141	0.82609	(rh.rostralmiddlefrontal_10)(rh.rostralmiddlefrontal_11)(rh.rostralmiddlefrontal_8)
6142	0.82609	(rh.rostralmiddlefrontal_10)(rh.rostralmiddlefrontal_12)(rh.rostralmiddlefrontal_8)
6143	0.82609	(rh.superiorfrontal_12)(rh.superiorfrontal_14)
6144	0.82609	(rh.superiorfrontal_2)(rh.superiorfrontal_6)(rh.superiorfrontal_7)(rh.superiorfrontal_9)
6145	0.82609	(rh.superiorfrontal_2)(rh.superiorfrontal_7)(rh.superiorfrontal_8)(rh.superiorfrontal_9)
6146	0.82609	(rh.superiorfrontal_4)(rh.superiorfrontal_6)(rh.superiorfrontal_8)
6147	0.82609	(Right-Caudate)(rh.caudalanteriorcingulate_1)(rh.rostralanteriorcingulate_1)
6148	0.82609	(Right-Caudate)(rh.rostralmiddlefrontal_12)(rh.superiorfrontal_2)
6149	0.82609	(Right-Caudate)(rh.superiorfrontal_5)(rh.superiorfrontal_7)(rh.superiorfrontal_9)
6150	0.82609	(Right-Caudate)(Right-Pallidum)(rh.precentral_13)(rh.precentral_7)

6151	0.82609	(Right-Caudate)(Right-Pallidum)(Right-Putamen)(rh.precentral_13)(rh.precentral_7)
6152	0.82609	(Right-Caudate)(Right-Putamen)(rh.lateralorbitofrontal_7)(rh.rostralmiddlefrontal_8)
6153	0.82609	(Right-Hippocampus)(rh.bankssts_2)(rh.middletemporal_1)
6154	0.82609	(Right-Hippocampus)(rh.inferiorparietal_1)(rh.inferiorparietal_4)
6155	0.82609	(Right-Putamen)(rh.lateralorbitofrontal_6)(rh.lateralorbitofrontal_7)(rh.rostralmiddlefrontal_12)(rh.rostralmiddlefrontal_13)
6156	0.82609	(Right-Putamen)(rh.lateralorbitofrontal_7)(rh.rostralmiddlefrontal_11)(rh.superiorfrontal_2)
6157	0.82609	(Right-Putamen)(rh.paracentral_4)
6158	0.82609	(Right-Putamen)(rh.postcentral_1)(rh.precentral_6)(rh.precentral_7)
6159	0.82609	(Right-Putamen)(rh.posteriorcingulate_2)(rh.superiorfrontal_11)
6160	0.82609	(Right-Putamen)(rh.rostralmiddlefrontal_1)(rh.rostralmiddlefrontal_3)
6161	0.82367	(Left-Caudate)(Left-Putamen)(Left-Thalamus-Proper)(lh.caudalmiddlefrontal_4)
6162	0.82367	(Left-Caudate)(Left-Putamen)(lh.caudalanteriorcingulate_1)(lh.caudalanteriorcingulate_2)(lh.posteriorcingulate_1)
6163	0.82367	(Left-Caudate)(Left-Putamen)(lh.medialorbitofrontal_2)(lh.parstriangularis_3)(lh.rostralmiddlefrontal_9)
6164	0.82367	(Left-Caudate)(Left-Putamen)(lh.parstriangularis_3)(lh.rostralanteriorcingulate_1)(lh.rostralmiddlefrontal_1)
6165	0.82367	(Left-Caudate)(Left-Putamen)(lh.postcentral_8)(lh.precentral_11)(lh.precentral_4)
6166	0.82367	(Left-Caudate)(Left-Thalamus-Proper)(lh.posteriorcingulate_3)(lh.precentral_11)
6167	0.82367	(Left-Caudate)(lh.caudalanteriorcingulate_2)(lh.superiorfrontal_14)(lh.superiorfrontal_4)
6168	0.82367	(Left-Caudate)(lh.insula_6)(lh.lateralorbitofrontal_6)
6169	0.82367	(Left-Caudate)(lh.lateralorbitofrontal_3)(lh.lateralorbitofrontal_4)
6170	0.82367	(Left-Caudate)(lh.lateralorbitofrontal_6)(lh.lateralorbitofrontal_7)(lh.parstriangularis_3)(lh.rostralmiddlefrontal_12)
6171	0.82367	(Left-Caudate)(lh.lateralorbitofrontal_7)(lh.medialorbitofrontal_2)(lh.rostralanteriorcingulate_1)(lh.rostralmiddlefrontal_12)(lh.rostralmiddlefrontal_9)
6172	0.82367	(Left-Caudate)(lh.posteriorcingulate_3)(lh.superiorfrontal_8)
6173	0.82367	(Left-Caudate)(lh.rostralmiddlefrontal_12)(lh.rostralmiddlefrontal_9)(lh.superiorfrontal_3)
6174	0.82367	(Left-Caudate)(lh.superiorfrontal_12)(lh.superiorfrontal_4)(lh.superiorfrontal_8)
6175	0.82367	(Left-Hippocampus)(lh.lingual_6)(lh.parahippocampal_3)
6176	0.82367	(Left-Hippocampus)(lh.lingual_8)(lh.precuneus_11)
6177	0.82367	(Left-Hippocampus)(lh.precuneus_6)
6178	0.82367	(Left-Putamen)(Left-Thalamus-Proper)(lh.caudalmiddlefrontal_4)
6179	0.82367	(Left-Putamen)(Left-Thalamus-Proper)(lh.precentral_7)(lh.precentral_9)
6180	0.82367	(Left-Putamen)(lh.caudalanteriorcingulate_1)(lh.caudalanteriorcingulate_2)(lh.posteriorcingulate_1)
6181	0.82367	(Left-Putamen)(lh.insula_6)(lh.lateralorbitofrontal_6)(lh.rostralmiddlefrontal_1)
6182	0.82367	(Left-Putamen)(lh.lateralorbitofrontal_2)(lh.rostralanteriorcingulate_1)
6183	0.82367	(Left-Putamen)(lh.lateralorbitofrontal_6)(lh.rostralmiddlefrontal_1)(lh.rostralmiddlefrontal_12)(lh.rostralmiddlefrontal_9)
6184	0.82367	(Left-Putamen)(lh.lateralorbitofrontal_7)(lh.superiorfrontal_3)
6185	0.82367	(Left-Putamen)(lh.precentral_12)(lh.precentral_16)
6186	0.82367	(Left-Putamen)(lh.rostralanteriorcingulate_2)(lh.rostralmiddlefrontal_9)(lh.superiorfrontal_4)
6187	0.82367	(Left-Putamen)(lh.rostralmiddlefrontal_8)(lh.superiorfrontal_3)(lh.superiorfrontal_4)
6188	0.82367	(Left-Thalamus-Proper)(lh.posteriorcingulate_3)(lh.precentral_11)
6189	0.82367	(lh.caudalanteriorcingulate_2)(lh.caudalmiddlefrontal_1)(lh.superiorfrontal_11)
6190	0.82367	(lh.caudalmiddlefrontal_5)(lh.caudalmiddlefrontal_6)(lh.superiorfrontal_12)
6191	0.82367	(lh.caudalmiddlefrontal_6)(lh.precentral_11)(lh.precentral_13)
6192	0.82367	(lh.cuneus_1)(lh.superiorparietal_12)(lh.superiorparietal_14)
6193	0.82367	(lh.cuneus_2)(lh.pericalcarine_3)
6194	0.82367	(lh.fusiform_5)(lh.fusiform_6)(lh.inferiortemporal_8)
6195	0.82367	(lh.fusiform_7)(lh.inferiortemporal_2)(lh.inferiortemporal_3)
6196	0.82367	(lh.lateralorbitofrontal_2)(lh.rostralanteriorcingulate_1)
6197	0.82367	(lh.lateralorbitofrontal_4)(lh.lateralorbitofrontal_6)(lh.parstriangularis_3)(lh.rostralmiddlefrontal_6)
6198	0.82367	(lh.lateralorbitofrontal_4)(lh.lateralorbitofrontal_6)(lh.rostralmiddlefrontal_6)(lh.rostralmiddlefrontal_9)
6199	0.82367	(lh.lateralorbitofrontal_4)(lh.medialorbitofrontal_2)(lh.rostralmiddlefrontal_12)
6200	0.82367	(lh.lateralorbitofrontal_5)(lh.parstriangularis_3)

6201	0.82367	(lh.lateralorbitofrontal_7)(lh.medialorbitofrontal_2)(lh.parstriangularis_3)(lh.rostralanteriorcingulate_1)
6202	0.82367	(lh.lateralorbitofrontal_7)(lh.parstriangularis_3)(lh.rostralmiddlefrontal_1)(lh.rostralmiddlefrontal_9)
6203	0.82367	(lh.lingual_1)(lh.lingual_4)(lh.lingual_8)(lh.pericalcarine_3)
6204	0.82367	(lh.lingual_5)(lh.pericalcarine_3)
6205	0.82367	(lh.medialorbitofrontal_2)(lh.parstriangularis_3)(lh.rostralanteriorcingulate_1)(lh.rostralmiddlefrontal_12)(lh.rostralmiddlefrontal_9)
6206	0.82367	(lh.paracentral_1)(lh.paracentral_4)(lh.paracentral_5)(lh.precentral_2)
6207	0.82367	(lh.pericalcarine_3)(lh.superiorparietal_11)
6208	0.82367	(lh.posteriorcingulate_3)(lh.precentral_11)(lh.precentral_4)
6209	0.82367	(lh.precuneus_2)(lh.precuneus_3)(lh.precuneus_6)
6210	0.82367	(lh.precuneus_8)(lh.precuneus_9)(lh.superiorparietal_6)
6211	0.82367	(lh.rostralanteriorcingulate_1)(lh.rostralanteriorcingulate_2)(lh.rostralmiddlefrontal_4)(lh.rostralmiddlefrontal_9)
6212	0.82367	(lh.rostralanteriorcingulate_1)(lh.rostralmiddlefrontal_4)(lh.superiorfrontal_3)(lh.superiorfrontal_4)
6213	0.82367	(lh.superiorfrontal_10)(lh.superiorfrontal_4)(lh.superiorfrontal_7)(lh.superiorfrontal_8)(lh.superiorfrontal_9)
6214	0.82367	(rh.fusiform_1)(rh.fusiform_2)(rh.fusiform_3)(rh.lateraloccipital_10)(rh.lateraloccipital_8)
6215	0.82367	(rh.fusiform_1)(rh.lateraloccipital_5)(rh.lateraloccipital_8)
6216	0.82367	(rh.fusiform_1)(rh.lateraloccipital_8)(rh.lingual_2)(rh.pericalcarine_1)
6217	0.82367	(rh.fusiform_8)(rh.inferiortemporal_1)(rh.inferiortemporal_2)
6218	0.82367	(rh.inferiorparietal_1)(rh.superiorparietal_10)(rh.superiorparietal_7)
6219	0.82367	(rh.inferiortemporal_2)(rh.inferiortemporal_3)(rh.inferiortemporal_4)
6220	0.82367	(rh.insula_3)(rh.precentral_6)(rh.precentral_7)
6221	0.82367	(rh.lateraloccipital_4)(rh.lateraloccipital_5)(rh.pericalcarine_4)
6222	0.82367	(rh.lateraloccipital_4)(rh.pericalcarine_3)(rh.superiorparietal_11)
6223	0.82367	(rh.lateralorbitofrontal_6)(rh.rostralmiddlefrontal_12)(rh.rostralmiddlefrontal_3)
6224	0.82367	(rh.parstriangularis_1)(rh.parstriangularis_4)(rh.rostralmiddlefrontal_12)(rh.rostralmiddlefrontal_3)(rh.rostralmiddlefrontal_8)
6225	0.82367	(rh.parstriangularis_1)(rh.rostralmiddlefrontal_12)(rh.rostralmiddlefrontal_5)(rh.rostralmiddlefrontal_8)
6226	0.82367	(rh.pericalcarine_2)(rh.pericalcarine_3)
6227	0.82367	(rh.precentral_10)(rh.precentral_6)(rh.precentral_8)
6228	0.82367	(rh.precuneus_4)(rh.precuneus_5)(rh.precuneus_8)
6229	0.82367	(rh.rostralmiddlefrontal_10)(rh.rostralmiddlefrontal_12)(rh.superiorfrontal_2)
6230	0.82367	(rh.superiorfrontal_1)(rh.superiorfrontal_2)(rh.superiorfrontal_3)
6231	0.82367	(rh.superiorfrontal_4)(rh.superiorfrontal_5)(rh.superiorfrontal_6)(rh.superiorfrontal_8)
6232	0.82367	(rh.superiorparietal_11)(rh.superiorparietal_7)
6233	0.82367	(Right-Accumbens-area)(rh.lateralorbitofrontal_4)
6234	0.82367	(Right-Caudate)(rh.caudalmiddlefrontal_4)(rh.superiorfrontal_11)(rh.superiorfrontal_9)
6235	0.82367	(Right-Caudate)(rh.insula_1)(rh.supramarginal_8)(rh.supramarginal_9)
6236	0.82367	(Right-Caudate)(rh.rostralmiddlefrontal_5)(rh.superiorfrontal_2)
6237	0.82367	(Right-Caudate)(Right-Putamen)(rh.parsopercularis_2)
6238	0.82367	(Right-Caudate)(Right-Putamen)(rh.rostralmiddlefrontal_3)(rh.rostralmiddlefrontal_8)
6239	0.82367	(Right-Caudate)(Right-Putamen)(rh.supramarginal_8)(rh.supramarginal_9)
6240	0.82367	(Right-Hippocampus)(rh.superiortemporal_3)(rh.transversetemporal_1)
6241	0.82367	(Right-Putamen)(rh.caudalmiddlefrontal_3)(rh.caudalmiddlefrontal_4)
6242	0.82367	(Right-Putamen)(rh.lateralorbitofrontal_7)(rh.medialorbitofrontal_2)(rh.rostralmiddlefrontal_12)(rh.superiorfrontal_2)
6243	0.82367	(Right-Putamen)(rh.precentral_5)(rh.precentral_6)(rh.precentral_7)
6244	0.82367	(Right-Putamen)(rh.rostralmiddlefrontal_11)(rh.rostralmiddlefrontal_8)(rh.superiorfrontal_2)
6245	0.82367	(Right-Putamen)(Right-Thalamus-Proper)(rh.precentral_7)
6246	0.82367	(Right-Thalamus-Proper)(rh.precentral_7)
6247	0.82126	(Left-Caudate)(Left-Putamen)(lh.caudalanteriorcingulate_2)(lh.posteriorcingulate_3)(lh.superiorfrontal_14)
6248	0.82126	(Left-Caudate)(Left-Putamen)(lh.lateralorbitofrontal_6)(lh.parstriangularis_3)(lh.rostralmiddlefrontal_1)
6249	0.82126	(Left-Caudate)(Left-Putamen)(lh.lateralorbitofrontal_7)(lh.medialorbitofrontal_2)(lh.parstriangularis_3)
6250	0.82126	(Left-Caudate)(Left-Putamen)(lh.lateralorbitofrontal_7)(lh.parstriangularis_3)(lh.rostralanteriorcingulate_1)(lh.rostralmiddlefrontal_9)

6251	0.82126	(Left-Caudate)(Left-Putamen)(lh.rostralmiddlefrontal_4)(lh.rostralmiddlefrontal_8)
6252	0.82126	(Left-Caudate)(Left-Putamen)(lh.superiorfrontal_10)(lh.superiorfrontal_11)(lh.superiorfrontal_4)
6253	0.82126	(Left-Caudate)(Left-Putamen)(lh.superiorfrontal_14)(lh.superiorfrontal_4)
6254	0.82126	(Left-Caudate)(Left-Thalamus-Proper)(lh.postcentral_8)(lh.precentral_11)
6255	0.82126	(Left-Caudate)(lh.caudalmiddlefrontal_1)(lh.caudalmiddlefrontal_6)
6256	0.82126	(Left-Caudate)(lh.lateralorbitofrontal_4)(lh.lateralorbitofrontal_6)(lh.parstriangularis_3)(lh.rostralmiddlefrontal_9)
6257	0.82126	(Left-Caudate)(lh.parstriangularis_3)(lh.rostralmiddlefrontal_1)(lh.rostralmiddlefrontal_12)
6258	0.82126	(Left-Caudate)(lh.rostralanteriorcingulate_1)(lh.rostralmiddlefrontal_4)(lh.rostralmiddlefrontal_9)(lh.superiorfrontal_4)
6259	0.82126	(Left-Caudate)(lh.rostralanteriorcingulate_2)(lh.rostralmiddlefrontal_4)(lh.superiorfrontal_10)
6260	0.82126	(Left-Caudate)(lh.rostralanteriorcingulate_2)(lh.rostralmiddlefrontal_4)(lh.superiorfrontal_11)
6261	0.82126	(Left-Caudate)(lh.rostralanteriorcingulate_2)(lh.rostralmiddlefrontal_8)(lh.superiorfrontal_4)
6262	0.82126	(Left-Hippocampus)(lh.fusiform_5)(lh.inferiorparietal_4)
6263	0.82126	(Left-Hippocampus)(lh.inferiorparietal_4)(lh.lingual_8)
6264	0.82126	(Left-Hippocampus)(lh.temporalpole_1)
6265	0.82126	(Left-Pallidum)(lh.posteriorcingulate_3)(lh.precentral_4)
6266	0.82126	(Left-Putamen)(lh.lateralorbitofrontal_3)(lh.lateralorbitofrontal_7)
6267	0.82126	(Left-Putamen)(lh.lateralorbitofrontal_4)(lh.medialorbitofrontal_2)(lh.rostralmiddlefrontal_9)
6268	0.82126	(Left-Putamen)(lh.lateralorbitofrontal_4)(lh.rostralmiddlefrontal_1)
6269	0.82126	(Left-Putamen)(lh.lateralorbitofrontal_7)(lh.medialorbitofrontal_2)(lh.parstriangularis_3)(lh.rostralmiddlefrontal_12)(lh.rostralmiddlefrontal_9)
6270	0.82126	(Left-Putamen)(lh.parstriangularis_3)(lh.rostralanteriorcingulate_2)(lh.rostralmiddlefrontal_9)
6271	0.82126	(Left-Putamen)(lh.rostralanteriorcingulate_2)(lh.superiorfrontal_10)
6272	0.82126	(Left-Putamen)(lh.rostralmiddlefrontal_1)(lh.rostralmiddlefrontal_3)(lh.rostralmiddlefrontal_4)
6273	0.82126	(Left-Thalamus-Proper)(lh.paracentral_5)(lh.posteriorcingulate_3)
6274	0.82126	(lh.bankssts_1)(lh.superiortemporal_2)
6275	0.82126	(lh.bankssts_3)(lh.fusiform_4)(lh.fusiform_5)
6276	0.82126	(lh.caudalanteriorcingulate_2)(lh.superiorfrontal_10)(lh.superiorfrontal_11)(lh.superiorfrontal_14)
6277	0.82126	(lh.caudalanteriorcingulate_2)(lh.superiorfrontal_11)(lh.superiorfrontal_14)(lh.superiorfrontal_4)(lh.superiorfrontal_8)
6278	0.82126	(lh.fusiform_7)(lh.inferiortemporal_6)(lh.inferiortemporal_7)
6279	0.82126	(lh.inferiorparietal_1)(lh.inferiorparietal_6)(lh.inferiorparietal_8)
6280	0.82126	(lh.inferiortemporal_3)(lh.inferiortemporal_4)(lh.middletemporal_4)
6281	0.82126	(lh.inferiortemporal_5)(lh.middletemporal_4)
6282	0.82126	(lh.insula_6)(lh.parstriangularis_3)(lh.rostralmiddlefrontal_4)(lh.rostralmiddlefrontal_9)
6283	0.82126	(lh.lateraloccipital_1)(lh.pericalcarine_1)(lh.superiorparietal_14)
6284	0.82126	(lh.lateraloccipital_11)(lh.lateraloccipital_3)(lh.pericalcarine_3)
6285	0.82126	(lh.lateraloccipital_6)(lh.lateraloccipital_7)(lh.lingual_1)(lh.pericalcarine_3)
6286	0.82126	(lh.lateraloccipital_6)(lh.lingual_6)(lh.pericalcarine_3)
6287	0.82126	(lh.lateraloccipital_6)(lh.precuneus_11)
6288	0.82126	(lh.lateraloccipital_7)(lh.lingual_6)(lh.lingual_8)
6289	0.82126	(lh.lateralorbitofrontal_4)(lh.lateralorbitofrontal_5)(lh.lateralorbitofrontal_6)
6290	0.82126	(lh.lateralorbitofrontal_4)(lh.lateralorbitofrontal_7)(lh.medialorbitofrontal_2)
6291	0.82126	(lh.lateralorbitofrontal_4)(lh.parstriangularis_3)(lh.rostralanteriorcingulate_1)(lh.rostralmiddlefrontal_12)(lh.rostralmiddlefrontal_9)
6292	0.82126	(lh.lateralorbitofrontal_4)(lh.parstriangularis_3)(lh.rostralmiddlefrontal_1)
6293	0.82126	(lh.lateralorbitofrontal_4)(lh.rostralmiddlefrontal_1)(lh.rostralmiddlefrontal_9)
6294	0.82126	(lh.lateralorbitofrontal_6)(lh.lateralorbitofrontal_7)(lh.parstriangularis_3)(lh.rostralmiddlefrontal_1)
6295	0.82126	(lh.lateralorbitofrontal_6)(lh.rostralmiddlefrontal_11)(lh.rostralmiddlefrontal_6)
6296	0.82126	(lh.lateralorbitofrontal_7)(lh.medialorbitofrontal_2)(lh.rostralanteriorcingulate_1)(lh.superiorfrontal_3)
6297	0.82126	(lh.lateralorbitofrontal_7)(lh.medialorbitofrontal_2)(lh.rostralmiddlefrontal_12)(lh.rostralmiddlefrontal_9)(lh.superiorfrontal_3)
6298	0.82126	(lh.paracentral_1)(lh.paracentral_5)(lh.precentral_3)
6299	0.82126	(lh.paracentral_5)(lh.postcentral_3)(lh.precentral_4)
6300	0.82126	(lh.paracentral_5)(lh.posteriorcingulate_3)(lh.superiorfrontal_14)

6301	0.82126	(lh.parsopercularis_1)(lh.parstriangularis_3)(lh.rostralmiddlefrontal_9)
6302	0.82126	(lh.parsopercularis_1)(lh.rostralmiddlefrontal_9)
6303	0.82126	(lh.parstriangularis_1)(lh.rostralmiddlefrontal_6)
6304	0.82126	(lh.parstriangularis_3)(lh.rostralmiddlefrontal_1)(lh.rostralmiddlefrontal_12)(lh.rostralmiddlefrontal_6)
6305	0.82126	(lh.rostralanteriorcingulate_1)(lh.rostralmiddlefrontal_1)(lh.rostralmiddlefrontal_4)
6306	0.82126	(lh.rostralanteriorcingulate_1)(lh.superiorfrontal_1)(lh.superiorfrontal_3)
6307	0.82126	(lh.rostralanteriorcingulate_2)(lh.superiorfrontal_3)
6308	0.82126	(lh.rostralmiddlefrontal_1)(lh.rostralmiddlefrontal_4)(lh.rostralmiddlefrontal_5)(lh.rostralmiddlefrontal_9)
6309	0.82126	(lh.superiorfrontal_10)(lh.superiorfrontal_11)(lh.superiorfrontal_4)(lh.superiorfrontal_6)(lh.superiorfrontal_7)(lh.superiorfrontal_8)
6310	0.82126	(lh.superiorfrontal_10)(lh.superiorfrontal_11)(lh.superiorfrontal_6)(lh.superiorfrontal_7)(lh.superiorfrontal_8)
6311	0.82126	(lh.superiorfrontal_13)(lh.superiorfrontal_7)(lh.superiorfrontal_8)
6312	0.82126	(lh.superiortemporal_2)(lh.supramarginal_6)
6313	0.82126	(rh.bankssts_2)(rh.bankssts_3)(rh.superiortemporal_1)
6314	0.82126	(rh.bankssts_2)(rh.inferiorparietal_4)(rh.superiortemporal_1)
6315	0.82126	(rh.caudalmiddlefrontal_3)(rh.precentral_9)
6316	0.82126	(rh.caudalmiddlefrontal_4)(rh.insula_7)(rh.parsopercularis_4)(rh.rostralmiddlefrontal_3)
6317	0.82126	(rh.caudalmiddlefrontal_4)(rh.parsopercularis_2)(rh.parsopercularis_4)
6318	0.82126	(rh.fusiform_1)(rh.lingual_7)(rh.pericalcarine_1)
6319	0.82126	(rh.fusiform_2)(rh.lateraloccipital_10)(rh.lingual_5)(rh.lingual_7)
6320	0.82126	(rh.fusiform_3)(rh.lateraloccipital_10)(rh.middletemporal_1)
6321	0.82126	(rh.inferiorparietal_1)(rh.inferiorparietal_4)(rh.supramarginal_8)
6322	0.82126	(rh.inferiorparietal_10)(rh.inferiorparietal_11)(rh.lateraloccipital_10)(rh.lateraloccipital_8)
6323	0.82126	(rh.inferiorparietal_4)(rh.superiorparietal_1)(rh.supramarginal_9)
6324	0.82126	(rh.inferiorparietal_8)(rh.inferiorparietal_9)
6325	0.82126	(rh.inferiortemporal_3)(rh.inferiortemporal_5)(rh.middletemporal_6)
6326	0.82126	(rh.inferiortemporal_5)(rh.middletemporal_2)(rh.middletemporal_6)
6327	0.82126	(rh.inferiortemporal_6)(rh.inferiortemporal_7)(rh.middletemporal_1)(rh.middletemporal_2)
6328	0.82126	(rh.lateraloccipital_1)(rh.lateraloccipital_3)(rh.lateraloccipital_4)(rh.pericalcarine_4)
6329	0.82126	(rh.lateraloccipital_1)(rh.lateraloccipital_4)(rh.superiorparietal_11)
6330	0.82126	(rh.lateraloccipital_10)(rh.lateraloccipital_4)
6331	0.82126	(rh.lateraloccipital_4)(rh.lateraloccipital_5)(rh.pericalcarine_3)
6332	0.82126	(rh.lateraloccipital_4)(rh.lingual_7)
6333	0.82126	(rh.lateralorbitofrontal_2)(rh.parstriangularis_1)(rh.parstriangularis_4)
6334	0.82126	(rh.lateralorbitofrontal_6)(rh.parstriangularis_2)(rh.parstriangularis_4)
6335	0.82126	(rh.lateralorbitofrontal_7)(rh.rostralmiddlefrontal_11)(rh.rostralmiddlefrontal_8)(rh.superiorfrontal_2)
6336	0.82126	(rh.paracentral_4)(rh.precentral_14)(rh.superiorfrontal_17)
6337	0.82126	(rh.parsopercularis_2)(rh.parstriangularis_2)(rh.rostralmiddlefrontal_3)
6338	0.82126	(rh.parsopercularis_4)(rh.parstriangularis_1)(rh.parstriangularis_2)(rh.rostralmiddlefrontal_3)
6339	0.82126	(rh.parsorbitalis_2)(rh.rostralmiddlefrontal_12)(rh.rostralmiddlefrontal_7)
6340	0.82126	(rh.parstriangularis_1)(rh.rostralmiddlefrontal_3)(rh.rostralmiddlefrontal_6)
6341	0.82126	(rh.pericalcarine_1)(rh.precuneus_1)(rh.superiorparietal_11)
6342	0.82126	(rh.postcentral_3)(rh.precentral_4)
6343	0.82126	(rh.precentral_10)(rh.precentral_5)
6344	0.82126	(rh.precuneus_4)(rh.precuneus_7)(rh.superiorparietal_7)(rh.superiorparietal_8)
6345	0.82126	(rh.rostralmiddlefrontal_1)(rh.rostralmiddlefrontal_2)(rh.rostralmiddlefrontal_5)
6346	0.82126	(rh.rostralmiddlefrontal_4)(rh.rostralmiddlefrontal_6)
6347	0.82126	(rh.rostralmiddlefrontal_5)(rh.rostralmiddlefrontal_9)(rh.superiorfrontal_2)
6348	0.82126	(rh.superiorfrontal_10)(rh.superiorfrontal_11)(rh.superiorfrontal_13)(rh.superiorfrontal_8)(rh.superiorfrontal_9)
6349	0.82126	(rh.superiorfrontal_11)(rh.superiorfrontal_13)(rh.superiorfrontal_5)(rh.superiorfrontal_8)
6350	0.82126	(rh.superiorfrontal_2)(rh.superiorfrontal_5)(rh.superiorfrontal_6)(rh.superiorfrontal_7)(rh.superiorfrontal_9)

6351	0.82126	(rh.superiortemporal_4)(rh.superiortemporal_6)
6352	0.82126	(Right-Amygdala)(rh.fusiform_8)(rh.insula_5)
6353	0.82126	(Right-Caudate)(rh.caudalanteriorcingulate_2)(rh.parsopercularis_4)
6354	0.82126	(Right-Caudate)(rh.caudalmiddlefrontal_4)(rh.superiorfrontal_11)(rh.superiorfrontal_7)
6355	0.82126	(Right-Caudate)(rh.insula_7)(rh.rostralmiddlefrontal_3)
6356	0.82126	(Right-Caudate)(Right-Putamen)(rh.parstriangularis_1)(rh.rostralmiddlefrontal_3)
6357	0.82126	(Right-Hippocampus)(Right-Putamen)(rh.superiorparietal_1)(rh.supramarginal_9)
6358	0.82126	(Right-Putamen)(rh.caudalmiddlefrontal_4)(rh.caudalmiddlefrontal_5)(rh.superiorfrontal_7)
6359	0.82126	(Right-Putamen)(rh.caudalmiddlefrontal_4)(rh.superiorfrontal_11)(rh.superiorfrontal_13)
6360	0.82126	(Right-Putamen)(rh.lateralorbitofrontal_7)(rh.rostralmiddlefrontal_12)(rh.rostralmiddlefrontal_8)(rh.superiorfrontal_2)
6361	0.81884	(Left-Accumbens-area)(Left-Amygdala)(Left-Hippocampus)(Left-Pallidum)(Left-Putamen)(Left-Thalamus-Proper)
6362	0.81884	(Left-Accumbens-area)(Left-Amygdala)(Left-Hippocampus)(Left-Pallidum)(Left-Putamen)
6363	0.81884	(Left-Accumbens-area)(lh.lateralorbitofrontal_7)
6364	0.81884	(Left-Caudate)(Left-Putamen)(Left-Thalamus-Proper)(lh.postcentral_8)(lh.precentral_11)
6365	0.81884	(Left-Caudate)(Left-Putamen)(lh.insula_6)(lh.lateralorbitofrontal_6)
6366	0.81884	(Left-Caudate)(Left-Putamen)(lh.medialorbitofrontal_2)(lh.parstriangularis_3)(lh.rostralanteriorcingulate_1)
6367	0.81884	(Left-Caudate)(Left-Putamen)(lh.precentral_4)(lh.superiorfrontal_12)
6368	0.81884	(Left-Caudate)(lh.caudalmiddlefrontal_4)(lh.caudalmiddlefrontal_6)(lh.precentral_11)(lh.precentral_9)
6369	0.81884	(Left-Caudate)(lh.lateralorbitofrontal_2)(lh.lateralorbitofrontal_7)(lh.medialorbitofrontal_5)
6370	0.81884	(Left-Caudate)(lh.posteriorcingulate_3)(lh.precentral_11)(lh.precentral_4)
6371	0.81884	(Left-Caudate)(lh.rostralmiddlefrontal_1)(lh.superiorfrontal_10)
6372	0.81884	(Left-Hippocampus)(lh.insula_2)(lh.insula_4)
6373	0.81884	(Left-Hippocampus)(lh.isthmuscingulate_3)(lh.precuneus_6)
6374	0.81884	(Left-Putamen)(lh.lateralorbitofrontal_2)(lh.medialorbitofrontal_4)
6375	0.81884	(Left-Putamen)(lh.lateralorbitofrontal_4)(lh.lateralorbitofrontal_7)(lh.medialorbitofrontal_2)
6376	0.81884	(Left-Putamen)(lh.medialorbitofrontal_2)(lh.parstriangularis_3)(lh.rostralanteriorcingulate_1)(lh.rostralmiddlefrontal_12)
6377	0.81884	(Left-Putamen)(lh.posteriorcingulate_3)(lh.precentral_11)
6378	0.81884	(Left-Putamen)(lh.precentral_13)(lh.precentral_14)
6379	0.81884	(Left-Putamen)(lh.rostralanteriorcingulate_2)(lh.superiorfrontal_11)(lh.superiorfrontal_4)
6380	0.81884	(Left-Putamen)(lh.rostralmiddlefrontal_12)(lh.rostralmiddlefrontal_9)(lh.superiorfrontal_4)
6381	0.81884	(Left-Putamen)(lh.rostralmiddlefrontal_4)(lh.superiorfrontal_10)(lh.superiorfrontal_4)
6382	0.81884	(Left-Putamen)(lh.superiorfrontal_12)(lh.superiorfrontal_4)
6383	0.81884	(lh.bankssts_2)(lh.inferiortemporal_7)(lh.inferiortemporal_8)
6384	0.81884	(lh.caudalanteriorcingulate_1)(lh.superiorfrontal_4)
6385	0.81884	(lh.caudalmiddlefrontal_3)(lh.insula_6)(lh.parsopercularis_1)
6386	0.81884	(lh.caudalmiddlefrontal_3)(lh.insula_6)(lh.parsopercularis_3)(lh.rostralmiddlefrontal_1)
6387	0.81884	(lh.caudalmiddlefrontal_5)(lh.superiorfrontal_11)
6388	0.81884	(lh.fusiform_1)(lh.fusiform_2)(lh.lingual_6)(lh.lingual_8)
6389	0.81884	(lh.fusiform_2)(lh.lateraloccipital_6)(lh.lingual_1)(lh.lingual_4)(lh.pericalcarine_3)
6390	0.81884	(lh.fusiform_4)(lh.inferiortemporal_7)(lh.inferiortemporal_8)
6391	0.81884	(lh.fusiform_7)(lh.middletemporal_3)(lh.middletemporal_4)
6392	0.81884	(lh.inferiorparietal_1)(lh.lingual_6)
6393	0.81884	(lh.inferiorparietal_5)(lh.inferiorparietal_9)(lh.superiortemporal_2)
6394	0.81884	(lh.insula_1)(lh.precentral_11)(lh.supramarginal_9)
6395	0.81884	(lh.insula_6)(lh.lateralorbitofrontal_6)(lh.rostralmiddlefrontal_9)
6396	0.81884	(lh.lingual_1)(lh.lingual_3)(lh.pericalcarine_2)(lh.pericalcarine_3)
6397	0.81884	(lh.paracentral_5)(lh.precentral_7)
6398	0.81884	(lh.paracentral_5)(lh.superiorfrontal_12)(lh.superiorfrontal_14)(lh.superiorfrontal_8)
6399	0.81884	(lh.parsopercularis_2)(lh.parsopercularis_3)(lh.rostralmiddlefrontal_1)
6400	0.81884	(lh.parstriangularis_3)(lh.rostralanteriorcingulate_1)(lh.rostralanteriorcingulate_2)(lh.rostralmiddlefrontal_9)

6401	0.81884	(lh.parstriangularis_3)(lh.rostralmiddlefrontal_1)(lh.rostralmiddlefrontal_12)(lh.rostralmiddlefrontal_6)(lh.rostralmiddlefrontal_9)
6402	0.81884	(lh.parstriangularis_3)(lh.rostralmiddlefrontal_4)(lh.rostralmiddlefrontal_8)
6403	0.81884	(lh.parstriangularis_3)(lh.rostralmiddlefrontal_7)
6404	0.81884	(lh.precentral_16)(lh.supramarginal_1)
6405	0.81884	(lh.precuneus_10)(lh.precuneus_6)(lh.precuneus_8)(lh.superiorparietal_11)
6406	0.81884	(lh.rostralanteriorcingulate_1)(lh.rostralmiddlefrontal_12)(lh.rostralmiddlefrontal_4)
6407	0.81884	(lh.rostralanteriorcingulate_2)(lh.rostralmiddlefrontal_1)(lh.rostralmiddlefrontal_9)
6408	0.81884	(lh.rostralmiddlefrontal_4)(lh.superiorfrontal_10)(lh.superiorfrontal_3)
6409	0.81884	(lh.superiorfrontal_10)(lh.superiorfrontal_11)(lh.superiorfrontal_12)
6410	0.81884	(lh.superiorfrontal_10)(lh.superiorfrontal_11)(lh.superiorfrontal_14)(lh.superiorfrontal_7)
6411	0.81884	(lh.superiorfrontal_10)(lh.superiorfrontal_11)(lh.superiorfrontal_14)(lh.superiorfrontal_9)
6412	0.81884	(lh.superiorfrontal_11)(lh.superiorfrontal_14)(lh.superiorfrontal_4)(lh.superiorfrontal_9)
6413	0.81884	(lh.superiortemporal_6)(lh.transversetemporal_1)
6414	0.81884	(rh.fusiform_6)(rh.lateraloccipital_10)
6415	0.81884	(rh.fusiform_8)(rh.inferiortemporal_2)(rh.middletemporal_9)
6416	0.81884	(rh.inferiorparietal_12)(rh.inferiorparietal_4)(rh.inferiorparietal_8)
6417	0.81884	(rh.inferiorparietal_12)(rh.lateraloccipital_4)(rh.lateraloccipital_8)(rh.pericalcarine_1)
6418	0.81884	(rh.insula_1)(rh.precentral_13)
6419	0.81884	(rh.lateraloccipital_10)(rh.lateraloccipital_4)(rh.lateraloccipital_8)
6420	0.81884	(rh.medialorbitofrontal_1)(rh.medialorbitofrontal_2)
6421	0.81884	(rh.medialorbitofrontal_2)(rh.rostralmiddlefrontal_8)(rh.superiorfrontal_2)
6422	0.81884	(rh.parsorbitalis_1)(rh.parstriangularis_1)(rh.rostralmiddlefrontal_12)
6423	0.81884	(rh.parstriangularis_1)(rh.superiorfrontal_2)
6424	0.81884	(rh.precentral_11)(rh.superiorparietal_1)(rh.supramarginal_1)
6425	0.81884	(rh.rostralmiddlefrontal_1)(rh.rostralmiddlefrontal_3)(rh.rostralmiddlefrontal_5)(rh.rostralmiddlefrontal_8)
6426	0.81884	(rh.rostralmiddlefrontal_10)(rh.rostralmiddlefrontal_8)(rh.rostralmiddlefrontal_9)
6427	0.81884	(rh.rostralmiddlefrontal_12)(rh.rostralmiddlefrontal_3)(rh.rostralmiddlefrontal_6)
6428	0.81884	(rh.superiorfrontal_2)(rh.superiorfrontal_5)(rh.superiorfrontal_7)(rh.superiorfrontal_8)(rh.superiorfrontal_9)
6429	0.81884	(Right-Accumbens-area)(Right-Putamen)(rh.lateralorbitofrontal_4)
6430	0.81884	(Right-Amygdala)(rh.insula_2)(rh.insula_4)
6431	0.81884	(Right-Amygdala)(Right-Putamen)(rh.insula_2)(rh.insula_4)
6432	0.81884	(Right-Caudate)(rh.lateralorbitofrontal_2)(rh.rostralmiddlefrontal_8)
6433	0.81884	(Right-Caudate)(rh.lateralorbitofrontal_4)(rh.lateralorbitofrontal_6)(rh.lateralorbitofrontal_7)
6434	0.81884	(Right-Caudate)(rh.lateralorbitofrontal_6)(rh.lateralorbitofrontal_7)(rh.rostralmiddlefrontal_12)
6435	0.81884	(Right-Caudate)(rh.superiorfrontal_13)(rh.superiorfrontal_14)
6436	0.81884	(Right-Caudate)(Right-Putamen)(rh.lateralorbitofrontal_2)(rh.rostralmiddlefrontal_8)
6437	0.81884	(Right-Caudate)(Right-Putamen)(rh.lateralorbitofrontal_4)(rh.lateralorbitofrontal_6)(rh.lateralorbitofrontal_7)
6438	0.81884	(Right-Caudate)(Right-Putamen)(rh.lateralorbitofrontal_6)(rh.lateralorbitofrontal_7)(rh.rostralmiddlefrontal_12)
6439	0.81884	(Right-Hippocampus)(rh.superiortemporal_1)(rh.superiortemporal_3)(rh.superiortemporal_9)
6440	0.81884	(Right-Putamen)(rh.insula_1)(rh.precentral_13)
6441	0.81884	(Right-Putamen)(rh.lateralorbitofrontal_5)(rh.lateralorbitofrontal_6)(rh.lateralorbitofrontal_7)
6442	0.81884	(Right-Putamen)(rh.lateralorbitofrontal_6)(rh.lateralorbitofrontal_7)(rh.rostralmiddlefrontal_12)(rh.rostralmiddlefrontal_8)
6443	0.81884	(Right-Putamen)(rh.lateralorbitofrontal_7)(rh.rostralmiddlefrontal_11)(rh.rostralmiddlefrontal_12)
6444	0.81884	(Right-Putamen)(rh.medialorbitofrontal_2)(rh.rostralmiddlefrontal_12)(rh.rostralmiddlefrontal_13)
6445	0.81884	(Right-Putamen)(rh.rostralanteriorcingulate_1)(rh.rostralmiddlefrontal_8)
6446	0.81643	(Left-Amygdala)(Left-Hippocampus)(lh.entorhinal_1)(lh.insula_4)
6447	0.81643	(Left-Amygdala)(Left-Hippocampus)(lh.temporalpole_1)
6448	0.81643	(Left-Caudate)(Left-Putamen)(lh.insula_6)(lh.rostralanteriorcingulate_2)
6449	0.81643	(Left-Caudate)(Left-Putamen)(lh.insula_6)(lh.rostralmiddlefrontal_4)(lh.rostralmiddlefrontal_9)
6450	0.81643	(Left-Caudate)(Left-Putamen)(lh.lateralorbitofrontal_6)(lh.parstriangularis_3)(lh.rostralmiddlefrontal_12)(lh.rostralmiddlefrontal_9)

6451	0.81643	(Left-Caudate)(Left-Putamen)(lh.posteriorcingulate_3)(lh.precentral_11)
6452	0.81643	(Left-Caudate)(lh.caudalanteriorcingulate_1)(lh.rostralmiddlefrontal_1)
6453	0.81643	(Left-Caudate)(lh.caudalanteriorcingulate_1)(lh.superiorfrontal_8)
6454	0.81643	(Left-Caudate)(lh.lateralorbitofrontal_6)(lh.lateralorbitofrontal_7)(lh.rostralmiddlefrontal_12)(lh.rostralmiddlefrontal_9)
6455	0.81643	(Left-Caudate)(lh.postcentral_3)(lh.posteriorcingulate_4)
6456	0.81643	(Left-Hippocampus)(lh.superiortemporal_2)(lh.superiortemporal_3)
6457	0.81643	(Left-Pallidum)(lh.precentral_11)(lh.precentral_7)
6458	0.81643	(Left-Putamen)(lh.caudalmiddlefrontal_1)(lh.rostralmiddlefrontal_1)(lh.rostralmiddlefrontal_4)
6459	0.81643	(Left-Putamen)(lh.insula_1)(lh.supramarginal_4)
6460	0.81643	(Left-Putamen)(lh.lateralorbitofrontal_4)(lh.medialorbitofrontal_2)(lh.rostralmiddlefrontal_12)
6461	0.81643	(Left-Putamen)(lh.rostralanteriorcingulate_2)(lh.rostralmiddlefrontal_8)(lh.superiorfrontal_4)
6462	0.81643	(Left-Putamen)(lh.rostralmiddlefrontal_1)(lh.rostralmiddlefrontal_4)(lh.superiorfrontal_4)
6463	0.81643	(lh.caudalanteriorcingulate_1)(lh.rostralmiddlefrontal_1)
6464	0.81643	(lh.caudalanteriorcingulate_2)(lh.superiorfrontal_10)(lh.superiorfrontal_11)(lh.superiorfrontal_14)(lh.superiorfrontal_8)
6465	0.81643	(lh.caudalmiddlefrontal_3)(lh.caudalmiddlefrontal_4)(lh.caudalmiddlefrontal_6)
6466	0.81643	(lh.fusiform_1)(lh.fusiform_4)(lh.lateraloccipital_9)
6467	0.81643	(lh.fusiform_2)(lh.fusiform_5)(lh.lateraloccipital_9)(lh.lingual_8)
6468	0.81643	(lh.fusiform_2)(lh.lateraloccipital_11)(lh.pericalcarine_2)
6469	0.81643	(lh.inferiorparietal_8)(lh.superiorparietal_11)(lh.superiorparietal_5)
6470	0.81643	(lh.isthmuscingulate_2)(lh.precuneus_3)(lh.precuneus_6)
6471	0.81643	(lh.lateraloccipital_11)(lh.lateraloccipital_7)
6472	0.81643	(lh.lateraloccipital_7)(lh.lateraloccipital_8)(lh.lateraloccipital_9)
6473	0.81643	(lh.lateralorbitofrontal_4)(lh.lateralorbitofrontal_7)(lh.parstriangularis_3)(lh.rostralanteriorcingulate_1)
6474	0.81643	(lh.lateralorbitofrontal_4)(lh.parstriangularis_3)(lh.rostralmiddlefrontal_6)(lh.rostralmiddlefrontal_9)
6475	0.81643	(lh.lateralorbitofrontal_6)(lh.lateralorbitofrontal_7)(lh.medialorbitofrontal_2)(lh.rostralmiddlefrontal_9)
6476	0.81643	(lh.lateralorbitofrontal_6)(lh.lateralorbitofrontal_7)(lh.parstriangularis_3)(lh.rostralmiddlefrontal_12)(lh.rostralmiddlefrontal_6)(lh.rostralmiddlefrontal_9)
6477	0.81643	(lh.lateralorbitofrontal_7)(lh.rostralanteriorcingulate_1)(lh.rostralmiddlefrontal_12)(lh.superiorfrontal_3)
6478	0.81643	(lh.medialorbitofrontal_2)(lh.superiorfrontal_2)(lh.superiorfrontal_3)
6479	0.81643	(lh.medialorbitofrontal_2)(lh.superiorfrontal_2)
6480	0.81643	(lh.paracentral_3)(lh.superiorparietal_1)
6481	0.81643	(lh.paracentral_5)(lh.precentral_4)(lh.superiorfrontal_15)
6482	0.81643	(lh.paracentral_5)(lh.superiorfrontal_16)
6483	0.81643	(lh.paracentral_5)(lh.superiorfrontal_17)
6484	0.81643	(lh.parstriangularis_3)(lh.rostralanteriorcingulate_2)(lh.rostralmiddlefrontal_1)
6485	0.81643	(lh.postcentral_3)(lh.supramarginal_4)
6486	0.81643	(lh.precuneus_6)(lh.precuneus_7)(lh.precuneus_8)
6487	0.81643	(lh.rostralanteriorcingulate_1)(lh.rostralmiddlefrontal_1)(lh.rostralmiddlefrontal_4)(lh.rostralmiddlefrontal_9)
6488	0.81643	(lh.rostralanteriorcingulate_1)(lh.rostralmiddlefrontal_12)(lh.rostralmiddlefrontal_9)(lh.superiorfrontal_3)(lh.superiorfrontal_4)
6489	0.81643	(lh.rostralanteriorcingulate_2)(lh.rostralmiddlefrontal_4)(lh.superiorfrontal_11)(lh.superiorfrontal_4)
6490	0.81643	(lh.rostralmiddlefrontal_1)(lh.rostralmiddlefrontal_4)(lh.rostralmiddlefrontal_9)(lh.superiorfrontal_4)
6491	0.81643	(lh.rostralmiddlefrontal_4)(lh.rostralmiddlefrontal_8)(lh.rostralmiddlefrontal_9)(lh.superiorfrontal_3)
6492	0.81643	(lh.superiorfrontal_10)(lh.superiorfrontal_11)(lh.superiorfrontal_12)(lh.superiorfrontal_8)
6493	0.81643	(lh.superiorfrontal_10)(lh.superiorfrontal_11)(lh.superiorfrontal_14)(lh.superiorfrontal_7)(lh.superiorfrontal_8)
6494	0.81643	(lh.superiorfrontal_10)(lh.superiorfrontal_11)(lh.superiorfrontal_4)(lh.superiorfrontal_7)(lh.superiorfrontal_9)
6495	0.81643	(lh.superiortemporal_11)(lh.superiortemporal_7)
6496	0.81643	(rh.bankssts_2)(rh.supramarginal_8)
6497	0.81643	(rh.caudalmiddlefrontal_1)(rh.precentral_13)
6498	0.81643	(rh.caudalmiddlefrontal_4)(rh.superiorfrontal_11)(rh.superiorfrontal_7)(rh.superiorfrontal_9)
6499	0.81643	(rh.fusiform_4)(rh.inferiortemporal_6)
6500	0.81643	(rh.inferiortemporal_3)(rh.middletemporal_4)(rh.middletemporal_7)

6501	0.81643	(rh.insula_1)(rh.precentral_11)(rh.superiorparietal_1)
6502	0.81643	(rh.lateraloccipital_1)(rh.lateraloccipital_4)(rh.pericalcarine_3)
6503	0.81643	(rh.lateraloccipital_4)(rh.lateraloccipital_6)(rh.lateraloccipital_7)
6504	0.81643	(rh.lateraloccipital_5)(rh.lateraloccipital_7)(rh.pericalcarine_4)
6505	0.81643	(rh.lateralorbitofrontal_2)(rh.lateralorbitofrontal_6)(rh.parstriangularis_4)
6506	0.81643	(rh.lateralorbitofrontal_7)(rh.parstriangularis_1)(rh.parstriangularis_4)(rh.rostralmiddlefrontal_12)
6507	0.81643	(rh.lateralorbitofrontal_7)(rh.parstriangularis_1)(rh.rostralmiddlefrontal_13)
6508	0.81643	(rh.medialorbitofrontal_2)(rh.rostralmiddlefrontal_12)(rh.rostralmiddlefrontal_8)
6509	0.81643	(rh.middletemporal_8)(rh.superiortemporal_7)
6510	0.81643	(rh.paracentral_4)(rh.precentral_14)(rh.superiorfrontal_16)
6511	0.81643	(rh.paracentral_6)(rh.precuneus_10)(rh.superiorparietal_2)
6512	0.81643	(rh.parsopercularis_4)(rh.rostralmiddlefrontal_8)
6513	0.81643	(rh.precuneus_1)(rh.superiorparietal_11)(rh.superiorparietal_13)
6514	0.81643	(rh.precuneus_4)(rh.superiorparietal_1)(rh.superiorparietal_11)
6515	0.81643	(rh.precuneus_5)(rh.precuneus_7)(rh.superiorparietal_10)
6516	0.81643	(rh.superiorfrontal_11)(rh.superiorfrontal_13)(rh.superiorfrontal_15)
6517	0.81643	(rh.superiortemporal_4)(rh.superiortemporal_5)
6518	0.81643	(Right-Caudate)(lh.posteriorcingulate_2)
6519	0.81643	(Right-Caudate)(rh.caudalmiddlefrontal_4)(rh.rostralmiddlefrontal_8)
6520	0.81643	(Right-Caudate)(rh.caudalmiddlefrontal_4)(rh.superiorfrontal_13)(rh.superiorfrontal_7)
6521	0.81643	(Right-Caudate)(Right-Putamen)(rh.caudalmiddlefrontal_4)(rh.rostralmiddlefrontal_8)
6522	0.81643	(Right-Caudate)(Right-Putamen)(rh.parsopercularis_2)(rh.parsopercularis_4)
6523	0.81643	(Right-Putamen)(rh.caudalmiddlefrontal_1)(rh.caudalmiddlefrontal_2)
6524	0.81643	(Right-Putamen)(rh.caudalmiddlefrontal_4)(rh.superiorfrontal_9)
6525	0.81643	(Right-Putamen)(rh.lateralorbitofrontal_6)(rh.parstriangularis_1)(rh.rostralmiddlefrontal_3)
6526	0.81643	(Right-Putamen)(rh.rostralmiddlefrontal_9)(rh.superiorfrontal_7)
6527	0.81643	(Right-Thalamus-Proper)(rh.transversetemporal_1)
6528	0.81401	(Left-Accumbens-area)(Right-Caudate)(lh.rostralanteriorcingulate_1)
6529	0.81401	(Left-Caudate)(Left-Putamen)(lh.caudalmiddlefrontal_1)(lh.caudalmiddlefrontal_3)(lh.rostralmiddlefrontal_1)
6530	0.81401	(Left-Caudate)(Left-Putamen)(lh.lateralorbitofrontal_7)(lh.medialorbitofrontal_2)(lh.rostralanteriorcingulate_1)(lh.rostralmiddlefrontal_12)(lh.rostralmiddlefrontal_9)
6531	0.81401	(Left-Caudate)(Left-Putamen)(lh.postcentral_8)(lh.precentral_11)(lh.precentral_7)
6532	0.81401	(Left-Caudate)(lh.caudalanteriorcingulate_2)(lh.caudalmiddlefrontal_1)(lh.superiorfrontal_11)
6533	0.81401	(Left-Caudate)(lh.lateralorbitofrontal_4)(lh.lateralorbitofrontal_6)(lh.rostralmiddlefrontal_12)
6534	0.81401	(Left-Caudate)(lh.parstriangularis_3)(lh.rostralmiddlefrontal_1)(lh.rostralmiddlefrontal_12)(lh.rostralmiddlefrontal_9)
6535	0.81401	(Left-Caudate)(lh.precentral_4)(lh.precentral_9)
6536	0.81401	(Left-Caudate)(lh.superiorfrontal_11)(lh.superiorfrontal_12)(lh.superiorfrontal_4)(lh.superiorfrontal_8)
6537	0.81401	(Left-Caudate)(Right-Caudate)(lh.posteriorcingulate_2)
6538	0.81401	(Left-Hippocampus)(lh.fusiform_5)(lh.fusiform_6)(lh.fusiform_7)
6539	0.81401	(Left-Hippocampus)(lh.isthmuscingulate_3)(lh.precuneus_10)
6540	0.81401	(Left-Hippocampus)(lh.precuneus_10)
6541	0.81401	(Left-Putamen)(lh.caudalanteriorcingulate_2)(lh.caudalmiddlefrontal_3)(lh.superiorfrontal_11)(lh.superiorfrontal_12)
6542	0.81401	(Left-Putamen)(lh.insula_1)(lh.supramarginal_1)(lh.supramarginal_9)
6543	0.81401	(Left-Putamen)(lh.insula_6)(lh.rostralmiddlefrontal_1)(lh.rostralmiddlefrontal_4)(lh.rostralmiddlefrontal_9)
6544	0.81401	(Left-Putamen)(lh.lateralorbitofrontal_6)(lh.parstriangularis_3)(lh.rostralmiddlefrontal_1)(lh.rostralmiddlefrontal_12)
6545	0.81401	(Left-Putamen)(lh.lateralorbitofrontal_7)(lh.medialorbitofrontal_4)(lh.medialorbitofrontal_5)
6546	0.81401	(Left-Putamen)(lh.lateralorbitofrontal_7)(lh.parstriangularis_3)(lh.rostralmiddlefrontal_1)
6547	0.81401	(Left-Putamen)(lh.parstriangularis_3)(lh.rostralanteriorcingulate_2)(lh.rostralmiddlefrontal_4)
6548	0.81401	(lh.caudalmiddlefrontal_1)(lh.rostralmiddlefrontal_1)(lh.rostralmiddlefrontal_3)
6549	0.81401	(lh.caudalmiddlefrontal_1)(lh.rostralmiddlefrontal_4)(lh.superiorfrontal_11)
6550	0.81401	(lh.fusiform_2)(lh.lateraloccipital_6)(lh.lateraloccipital_8)

6551	0.81401	(lh.inferiorparietal_1)(lh.lingual_8)
6552	0.81401	(lh.inferiorparietal_1)(lh.precuneus_11)(lh.precuneus_9)
6553	0.81401	(lh.inferiorparietal_4)(lh.pericalcarine_3)
6554	0.81401	(lh.isthmuscingulate_3)(lh.lingual_6)(lh.lingual_7)
6555	0.81401	(lh.lateraloccipital_3)(lh.pericalcarine_1)(lh.pericalcarine_2)(lh.pericalcarine_3)
6556	0.81401	(lh.lateraloccipital_5)(lh.lateraloccipital_6)
6557	0.81401	(lh.lateraloccipital_7)(lh.lingual_6)(lh.pericalcarine_3)
6558	0.81401	(lh.paracentral_3)(lh.precentral_2)
6559	0.81401	(lh.postcentral_8)(lh.precentral_7)(lh.supramarginal_9)
6560	0.81401	(lh.precuneus_2)(lh.precuneus_3)(lh.superiorparietal_5)
6561	0.81401	(lh.rostralanteriorcingulate_1)(lh.superiorfrontal_2)(lh.superiorfrontal_3)(lh.superiorfrontal_4)
6562	0.81401	(lh.rostralmiddlefrontal_1)(lh.rostralmiddlefrontal_4)(lh.rostralmiddlefrontal_8)(lh.rostralmiddlefrontal_9)
6563	0.81401	(lh.superiorfrontal_10)(lh.superiorfrontal_11)(lh.superiorfrontal_3)(lh.superiorfrontal_4)
6564	0.81401	(lh.superiorfrontal_10)(lh.superiorfrontal_11)(lh.superiorfrontal_3)
6565	0.81401	(rh.bankssts_3)(rh.superiortemporal_1)(rh.superiortemporal_3)
6566	0.81401	(rh.caudalmiddlefrontal_4)(rh.superiorfrontal_13)(rh.superiorfrontal_14)
6567	0.81401	(rh.cuneus_2)(rh.cuneus_3)(rh.pericalcarine_2)
6568	0.81401	(rh.cuneus_4)(rh.lateraloccipital_1)(rh.lateraloccipital_3)
6569	0.81401	(rh.cuneus_4)(rh.pericalcarine_4)
6570	0.81401	(rh.fusiform_1)(rh.inferiorparietal_12)
6571	0.81401	(rh.fusiform_1)(rh.lateraloccipital_4)(rh.pericalcarine_3)
6572	0.81401	(rh.fusiform_1)(rh.lateraloccipital_7)(rh.lingual_2)(rh.pericalcarine_3)
6573	0.81401	(rh.inferiortemporal_4)(rh.inferiortemporal_5)(rh.middletemporal_4)(rh.middletemporal_6)
6574	0.81401	(rh.insula_3)(rh.postcentral_1)(rh.precentral_7)
6575	0.81401	(rh.lateraloccipital_5)(rh.lateraloccipital_7)(rh.pericalcarine_3)
6576	0.81401	(rh.lateraloccipital_7)(rh.lateraloccipital_8)(rh.pericalcarine_3)
6577	0.81401	(rh.lateraloccipital_8)(rh.lingual_2)(rh.pericalcarine_3)
6578	0.81401	(rh.lateralorbitofrontal_6)(rh.lateralorbitofrontal_7)(rh.parstriangularis_1)(rh.rostralmiddlefrontal_13)
6579	0.81401	(rh.paracentral_4)(rh.precentral_13)(rh.superiorfrontal_14)
6580	0.81401	(rh.parstriangularis_1)(rh.rostralmiddlefrontal_8)(rh.superiorfrontal_2)
6581	0.81401	(rh.parstriangularis_4)(rh.rostralmiddlefrontal_6)
6582	0.81401	(rh.precentral_11)(rh.supramarginal_8)
6583	0.81401	(rh.precuneus_10)(rh.precuneus_4)
6584	0.81401	(rh.precuneus_2)(rh.precuneus_3)
6585	0.81401	(rh.precuneus_7)(rh.superiorparietal_1)(rh.superiorparietal_7)(rh.superiorparietal_8)
6586	0.81401	(rh.rostralanteriorcingulate_2)(rh.rostralmiddlefrontal_3)
6587	0.81401	(rh.superiorfrontal_11)(rh.superiorfrontal_15)(rh.superiorfrontal_16)
6588	0.81401	(Right-Accumbens-area)(rh.medialorbitofrontal_5)
6589	0.81401	(Right-Caudate)(rh.insula_1)(rh.precentral_13)
6590	0.81401	(Right-Caudate)(rh.lateralorbitofrontal_2)(rh.lateralorbitofrontal_6)(rh.lateralorbitofrontal_7)
6591	0.81401	(Right-Caudate)(rh.lateralorbitofrontal_4)(rh.medialorbitofrontal_2)
6592	0.81401	(Right-Caudate)(Right-Putamen)(rh.insula_1)(rh.precentral_13)
6593	0.81401	(Right-Caudate)(Right-Putamen)(rh.lateralorbitofrontal_2)(rh.lateralorbitofrontal_6)(rh.lateralorbitofrontal_7)
6594	0.81401	(Right-Hippocampus)(rh.inferiorparietal_10)(rh.middletemporal_1)
6595	0.81401	(Right-Hippocampus)(rh.middletemporal_4)(rh.superiortemporal_6)
6596	0.81401	(Right-Putamen)(rh.lateralorbitofrontal_1)(rh.lateralorbitofrontal_2)
6597	0.81401	(Right-Putamen)(rh.medialorbitofrontal_2)(rh.rostralmiddlefrontal_11)(rh.rostralmiddlefrontal_12)
6598	0.81401	(Right-Putamen)(rh.parsopercularis_4)(rh.rostralmiddlefrontal_8)
6599	0.81159	(Left-Caudate)(Left-Putamen)(lh.caudalmiddlefrontal_3)(lh.caudalmiddlefrontal_6)
6600	0.81159	(Left-Caudate)(Left-Putamen)(lh.lateralorbitofrontal_7)(lh.parstriangularis_3)(lh.rostralanteriorcingulate_1)(lh.rostralmiddlefrontal_12)

6601	0.81159	(Left-Caudate)(Left-Putamen)(lh.parstriangularis_3)(lh.rostralanteriorcingulate_1)(lh.rostralmiddlefrontal_4)(lh.rostralmiddlefrontal_9)
6602	0.81159	(Left-Caudate)(Left-Putamen)(lh.posteriorcingulate_1)(lh.posteriorcingulate_2)
6603	0.81159	(Left-Caudate)(lh.caudalmiddlefrontal_3)(lh.caudalmiddlefrontal_4)(lh.superiorfrontal_12)
6604	0.81159	(Left-Caudate)(lh.caudalmiddlefrontal_3)(lh.parsopercularis_3)(lh.rostralmiddlefrontal_1)
6605	0.81159	(Left-Caudate)(lh.medialorbitofrontal_2)(lh.parstriangularis_3)(lh.rostralanteriorcingulate_1)(lh.rostralmiddlefrontal_9)
6606	0.81159	(Left-Caudate)(lh.medialorbitofrontal_2)(lh.rostralmiddlefrontal_12)(lh.rostralmiddlefrontal_9)(lh.superiorfrontal_3)
6607	0.81159	(Left-Caudate)(lh.paracentral_5)(lh.superiorfrontal_14)(lh.superiorfrontal_8)
6608	0.81159	(Left-Caudate)(lh.rostralanteriorcingulate_1)(lh.rostralmiddlefrontal_12)(lh.superiorfrontal_3)
6609	0.81159	(Left-Caudate)(lh.rostralmiddlefrontal_1)(lh.rostralmiddlefrontal_9)(lh.superiorfrontal_4)
6610	0.81159	(Left-Putamen)(lh.caudalmiddlefrontal_3)(lh.caudalmiddlefrontal_6)
6611	0.81159	(Left-Putamen)(lh.caudalmiddlefrontal_3)(lh.rostralmiddlefrontal_1)(lh.rostralmiddlefrontal_3)
6612	0.81159	(Left-Putamen)(lh.caudalmiddlefrontal_4)(lh.insula_6)
6613	0.81159	(Left-Putamen)(lh.insula_1)(lh.superiorparietal_3)(lh.supramarginal_1)
6614	0.81159	(Left-Putamen)(lh.lateralorbitofrontal_4)(lh.parstriangularis_3)(lh.rostralanteriorcingulate_1)(lh.rostralmiddlefrontal_12)
6615	0.81159	(Left-Putamen)(lh.lateralorbitofrontal_7)(lh.rostralmiddlefrontal_1)(lh.rostralmiddlefrontal_9)
6616	0.81159	(Left-Putamen)(lh.posteriorcingulate_1)(lh.posteriorcingulate_2)
6617	0.81159	(Left-Putamen)(lh.rostralanteriorcingulate_1)(lh.rostralmiddlefrontal_8)
6618	0.81159	(Left-Putamen)(lh.rostralanteriorcingulate_1)(lh.rostralmiddlefrontal_9)(lh.superiorfrontal_3)(lh.superiorfrontal_4)
6619	0.81159	(Left-Putamen)(lh.rostralmiddlefrontal_8)(lh.rostralmiddlefrontal_9)(lh.superiorfrontal_3)
6620	0.81159	(Left-Putamen)(lh.rostralmiddlefrontal_8)(lh.rostralmiddlefrontal_9)(lh.superiorfrontal_4)
6621	0.81159	(Left-Putamen)(lh.superiorfrontal_8)
6622	0.81159	(Left-Thalamus-Proper)(lh.superiortemporal_3)
6623	0.81159	(lh.caudalmiddlefrontal_4)(lh.caudalmiddlefrontal_5)(lh.precentral_9)
6624	0.81159	(lh.caudalmiddlefrontal_4)(lh.insula_6)
6625	0.81159	(lh.fusiform_4)(lh.inferiortemporal_8)(lh.lateraloccipital_9)
6626	0.81159	(lh.inferiorparietal_5)(lh.supramarginal_6)
6627	0.81159	(lh.lateraloccipital_11)(lh.lateraloccipital_3)(lh.lateraloccipital_6)(lh.pericalcarine_2)
6628	0.81159	(lh.lateraloccipital_11)(lh.lateraloccipital_6)(lh.lateraloccipital_7)
6629	0.81159	(lh.lateraloccipital_11)(lh.lateraloccipital_6)(lh.pericalcarine_2)(lh.pericalcarine_3)
6630	0.81159	(lh.lateraloccipital_4)(lh.pericalcarine_2)
6631	0.81159	(lh.lateralorbitofrontal_4)(lh.medialorbitofrontal_2)(lh.rostralmiddlefrontal_12)(lh.rostralmiddlefrontal_9)
6632	0.81159	(lh.medialorbitofrontal_2)(lh.rostralanteriorcingulate_1)(lh.superiorfrontal_1)(lh.superiorfrontal_3)
6633	0.81159	(lh.paracentral_4)(lh.superiorfrontal_14)(lh.superiorfrontal_16)
6634	0.81159	(lh.parstriangularis_3)(lh.rostralmiddlefrontal_9)(lh.superiorfrontal_3)
6635	0.81159	(lh.parstriangularis_3)(lh.superiorfrontal_3)
6636	0.81159	(lh.postcentral_14)(lh.supramarginal_1)(lh.supramarginal_2)(lh.supramarginal_4)
6637	0.81159	(lh.precuneus_6)(lh.superiorparietal_6)(lh.superiorparietal_7)
6638	0.81159	(lh.rostralanteriorcingulate_2)(lh.superiorfrontal_3)(lh.superiorfrontal_4)
6639	0.81159	(lh.rostralmiddlefrontal_1)(lh.rostralmiddlefrontal_4)(lh.superiorfrontal_10)
6640	0.81159	(lh.rostralmiddlefrontal_11)(lh.rostralmiddlefrontal_9)
6641	0.81159	(lh.rostralmiddlefrontal_8)(lh.superiorfrontal_10)(lh.superiorfrontal_3)
6642	0.81159	(lh.superiorfrontal_10)(lh.superiorfrontal_11)(lh.superiorfrontal_4)(lh.superiorfrontal_8)(lh.superiorfrontal_9)
6643	0.81159	(rh.bankssts_1)(rh.bankssts_2)(rh.superiortemporal_3)
6644	0.81159	(rh.bankssts_2)(rh.middletemporal_2)
6645	0.81159	(rh.caudalmiddlefrontal_4)(rh.parsopercularis_4)(rh.rostralmiddlefrontal_1)
6646	0.81159	(rh.inferiorparietal_10)(rh.inferiorparietal_6)(rh.inferiorparietal_8)
6647	0.81159	(rh.inferiorparietal_11)(rh.inferiorparietal_8)
6648	0.81159	(rh.inferiorparietal_12)(rh.lateraloccipital_10)
6649	0.81159	(rh.insula_7)(rh.rostralmiddlefrontal_3)(rh.rostralmiddlefrontal_8)
6650	0.81159	(rh.lateraloccipital_3)(rh.pericalcarine_1)(rh.pericalcarine_2)

6651	0.81159	(rh.lateraloccipital_4)(rh.precuneus_1)
6652	0.81159	(rh.lateralorbitofrontal_6)(rh.parstriangularis_1)(rh.parstriangularis_4)(rh.rostralmiddlefrontal_12)(rh.rostralmiddlefrontal_8)
6653	0.81159	(rh.lateralorbitofrontal_6)(rh.superiorfrontal_2)
6654	0.81159	(rh.middletemporal_9)(rh.superiortemporal_6)
6655	0.81159	(rh.postcentral_1)(rh.postcentral_3)(rh.supramarginal_7)
6656	0.81159	(rh.precentral_5)(rh.precentral_9)
6657	0.81159	(rh.rostralmiddlefrontal_10)(rh.rostralmiddlefrontal_11)(rh.rostralmiddlefrontal_8)(rh.superiorfrontal_2)
6658	0.81159	(Right-Accumbens-area)(Right-Amygdala)(Right-Caudate)(Right-Hippocampus)(Right-Pallidum)(Right-Thalamus-Proper)
6659	0.81159	(Right-Accumbens-area)(Right-Amygdala)(Right-Caudate)(Right-Hippocampus)(Right-Pallidum)
6660	0.81159	(Right-Accumbens-area)(Right-Amygdala)(Right-Caudate)(Right-Hippocampus)(Right-Thalamus-Proper)
6661	0.81159	(Right-Accumbens-area)(Right-Amygdala)(Right-Caudate)(Right-Hippocampus)
6662	0.81159	(Right-Caudate)(rh.caudalmiddlefrontal_4)(rh.superiorfrontal_13)(rh.superiorfrontal_9)
6663	0.81159	(Right-Caudate)(rh.lateralorbitofrontal_2)(rh.lateralorbitofrontal_6)(rh.parstriangularis_1)
6664	0.81159	(Right-Caudate)(rh.rostralmiddlefrontal_11)
6665	0.81159	(Right-Caudate)(Right-Putamen)(rh.lateralorbitofrontal_4)(rh.medialorbitofrontal_2)
6666	0.81159	(Right-Hippocampus)(rh.inferiortemporal_7)
6667	0.81159	(Right-Putamen)(rh.lateralorbitofrontal_7)(rh.medialorbitofrontal_2)(rh.rostralmiddlefrontal_12)(rh.rostralmiddlefrontal_13)
6668	0.81159	(Right-Putamen)(rh.medialorbitofrontal_2)(rh.rostralmiddlefrontal_12)(rh.rostralmiddlefrontal_8)
6669	0.81159	(Right-Putamen)(rh.rostralmiddlefrontal_5)(rh.rostralmiddlefrontal_9)
6670	0.81159	(Right-Putamen)(rh.rostralmiddlefrontal_8)(rh.rostralmiddlefrontal_9)(rh.superiorfrontal_2)
6671	0.81159	(Right-Putamen)(rh.rostralmiddlefrontal_8)(rh.superiorfrontal_2)(rh.superiorfrontal_7)
6672	0.80918	(Left-Accumbens-area)(Left-Caudate)(Right-Caudate)(lh.rostralanteriorcingulate_1)
6673	0.80918	(Left-Caudate)(Left-Pallidum)(Left-Putamen)(lh.precentral_14)
6674	0.80918	(Left-Caudate)(Left-Pallidum)(lh.precentral_14)
6675	0.80918	(Left-Caudate)(Left-Putamen)(lh.caudalanteriorcingulate_2)(lh.caudalmiddlefrontal_3)(lh.superiorfrontal_11)(lh.superiorfrontal_12)
6676	0.80918	(Left-Caudate)(Left-Putamen)(lh.lateralorbitofrontal_2)(lh.lateralorbitofrontal_7)(lh.medialorbitofrontal_5)
6677	0.80918	(Left-Caudate)(Left-Putamen)(lh.lateralorbitofrontal_4)(lh.lateralorbitofrontal_6)(lh.rostralmiddlefrontal_12)
6678	0.80918	(Left-Caudate)(Left-Putamen)(lh.lateralorbitofrontal_6)(lh.lateralorbitofrontal_7)(lh.rostralmiddlefrontal_12)(lh.rostralmiddlefrontal_9)
6679	0.80918	(Left-Caudate)(Left-Putamen)(lh.rostralanteriorcingulate_2)(lh.superiorfrontal_10)
6680	0.80918	(Left-Caudate)(Left-Putamen)(lh.rostralmiddlefrontal_12)(lh.superiorfrontal_4)
6681	0.80918	(Left-Caudate)(Left-Putamen)(lh.rostralmiddlefrontal_3)
6682	0.80918	(Left-Caudate)(Left-Thalamus-Proper)(lh.precentral_7)(lh.precentral_9)
6683	0.80918	(Left-Caudate)(lh.lateralorbitofrontal_6)(lh.medialorbitofrontal_2)
6684	0.80918	(Left-Caudate)(lh.superiorfrontal_10)(lh.superiorfrontal_12)
6685	0.80918	(Left-Caudate)(lh.superiorfrontal_12)(lh.superiorfrontal_14)(lh.superiorfrontal_4)
6686	0.80918	(Left-Pallidum)(Left-Putamen)(Left-Thalamus-Proper)(lh.precentral_11)(lh.precentral_9)
6687	0.80918	(Left-Pallidum)(Left-Thalamus-Proper)(lh.precentral_11)(lh.precentral_9)
6688	0.80918	(Left-Putamen)(lh.lateralorbitofrontal_6)(lh.parstriangularis_3)(lh.rostralmiddlefrontal_1)(lh.rostralmiddlefrontal_12)(lh.rostralmiddlefrontal_9)
6689	0.80918	(Left-Putamen)(lh.parstriangularis_3)(lh.rostralmiddlefrontal_8)
6690	0.80918	(Left-Putamen)(lh.rostralanteriorcingulate_1)(lh.rostralanteriorcingulate_2)(lh.rostralmiddlefrontal_9)(lh.superiorfrontal_4)
6691	0.80918	(Left-Thalamus-Proper)(lh.isthmuscingulate_2)(lh.posteriorcingulate_4)
6692	0.80918	(lh.bankssts_3)(lh.inferiortemporal_7)
6693	0.80918	(lh.caudalmiddlefrontal_1)(lh.caudalmiddlefrontal_4)(lh.caudalmiddlefrontal_6)
6694	0.80918	(lh.cuneus_1)(lh.inferiorparietal_1)(lh.pericalcarine_3)
6695	0.80918	(lh.fusiform_2)(lh.inferiorparietal_4)
6696	0.80918	(lh.fusiform_2)(lh.lateraloccipital_11)(lh.lateraloccipital_6)(lh.lingual_1)(lh.pericalcarine_3)
6697	0.80918	(lh.fusiform_5)(lh.inferiortemporal_7)(lh.inferiortemporal_8)
6698	0.80918	(lh.fusiform_7)(lh.inferiortemporal_8)
6699	0.80918	(lh.inferiorparietal_9)(lh.precuneus_9)
6700	0.80918	(lh.inferiortemporal_6)(lh.middletemporal_4)

6701	0.80918	(lh.lateralorbitofrontal_4)(lh.lateralorbitofrontal_7)(lh.medialorbitofrontal_2)(lh.rostralmiddlefrontal_12)
6702	0.80918	(lh.lateralorbitofrontal_4)(lh.parstriangularis_3)(lh.rostralmiddlefrontal_1)(lh.rostralmiddlefrontal_9)
6703	0.80918	(lh.lateralorbitofrontal_4)(lh.rostralmiddlefrontal_12)(lh.rostralmiddlefrontal_6)
6704	0.80918	(lh.paracentral_1)(lh.paracentral_4)(lh.paracentral_5)(lh.precentral_3)
6705	0.80918	(lh.paracentral_5)(lh.precentral_4)(lh.superiorfrontal_12)
6706	0.80918	(lh.paracentral_5)(lh.superiorparietal_1)
6707	0.80918	(lh.parsorbitalis_1)(lh.rostralmiddlefrontal_11)
6708	0.80918	(lh.precuneus_10)(lh.precuneus_6)(lh.superiorparietal_5)
6709	0.80918	(lh.precuneus_3)(lh.superiorparietal_6)(lh.superiorparietal_7)
6710	0.80918	(lh.rostralanteriorcingulate_1)(lh.rostralmiddlefrontal_8)(lh.rostralmiddlefrontal_9)(lh.superiorfrontal_3)
6711	0.80918	(lh.rostralmiddlefrontal_12)(lh.rostralmiddlefrontal_8)(lh.rostralmiddlefrontal_9)
6712	0.80918	(lh.rostralmiddlefrontal_8)(lh.superiorfrontal_10)(lh.superiorfrontal_3)(lh.superiorfrontal_4)
6713	0.80918	(lh.superiorfrontal_11)(lh.superiorfrontal_12)(lh.superiorfrontal_14)(lh.superiorfrontal_4)
6714	0.80918	(lh.superiorfrontal_12)(lh.superiorfrontal_14)(lh.superiorfrontal_15)(lh.superiorfrontal_17)
6715	0.80918	(lh.superiorfrontal_12)(lh.superiorfrontal_15)(lh.superiorfrontal_17)
6716	0.80918	(rh.fusiform_1)(rh.lingual_5)(rh.lingual_6)
6717	0.80918	(rh.lateraloccipital_8)(rh.pericalcarine_1)(rh.superiorparietal_11)
6718	0.80918	(rh.parstriangularis_1)(rh.rostralmiddlefrontal_12)(rh.rostralmiddlefrontal_7)
6719	0.80918	(rh.parstriangularis_1)(rh.rostralmiddlefrontal_3)(rh.rostralmiddlefrontal_6)(rh.rostralmiddlefrontal_8)
6720	0.80918	(rh.parstriangularis_2)(rh.parstriangularis_4)(rh.rostralmiddlefrontal_8)
6721	0.80918	(rh.precentral_7)(rh.superiorparietal_1)
6722	0.80918	(rh.precuneus_4)(rh.precuneus_7)(rh.superiorparietal_1)(rh.superiorparietal_10)
6723	0.80918	(rh.superiorparietal_1)(rh.superiorparietal_10)(rh.superiorparietal_11)
6724	0.80918	(rh.superiorparietal_2)(rh.supramarginal_8)
6725	0.80918	(Right-Caudate)(rh.caudalmiddlefrontal_4)(rh.precentral_13)(rh.precentral_7)
6726	0.80918	(Right-Caudate)(rh.caudalmiddlefrontal_5)(rh.precentral_13)
6727	0.80918	(Right-Caudate)(rh.isthmuscingulate_1)(rh.posteriorcingulate_3)
6728	0.80918	(Right-Caudate)(rh.precentral_10)(rh.precentral_13)(rh.precentral_7)
6729	0.80918	(Right-Caudate)(Right-Putamen)(rh.rostralmiddlefrontal_12)(rh.superiorfrontal_2)
6730	0.80918	(Right-Caudate)(Right-Putamen)(rh.rostralmiddlefrontal_3)(rh.rostralmiddlefrontal_5)
6731	0.80918	(Right-Caudate)(Right-Putamen)(rh.superiorfrontal_14)
6732	0.80918	(Right-Hippocampus)(rh.inferiortemporal_6)
6733	0.80918	(Right-Putamen)(rh.caudalmiddlefrontal_5)(rh.parsopercularis_4)
6734	0.80918	(Right-Putamen)(rh.parstriangularis_1)(rh.superiorfrontal_2)
6735	0.80918	(Right-Putamen)(rh.rostralmiddlefrontal_2)
6736	0.80918	(Right-Putamen)(Right-Thalamus-Proper)(rh.insula_2)(rh.precentral_7)
6737	0.80918	(Right-Thalamus-Proper)(rh.insula_2)(rh.precentral_7)
6738	0.80676	(Left-Caudate)(Left-Pallidum)(Left-Putamen)(Left-Thalamus-Proper)(lh.precentral_11)(lh.precentral_9)
6739	0.80676	(Left-Caudate)(Left-Pallidum)(Left-Thalamus-Proper)(lh.precentral_11)(lh.precentral_9)
6740	0.80676	(Left-Caudate)(Left-Putamen)(lh.medialorbitofrontal_2)(lh.medialorbitofrontal_4)(lh.rostralanteriorcingulate_1)
6741	0.80676	(Left-Caudate)(Left-Putamen)(lh.precentral_4)(lh.precentral_9)
6742	0.80676	(Left-Caudate)(Left-Thalamus-Proper)(lh.paracentral_5)(lh.posteriorcingulate_3)
6743	0.80676	(Left-Caudate)(lh.lateralorbitofrontal_4)(lh.parstriangularis_3)(lh.rostralanteriorcingulate_1)
6744	0.80676	(Left-Caudate)(lh.rostralanteriorcingulate_1)(lh.rostralanteriorcingulate_2)(lh.rostralmiddlefrontal_4)(lh.rostralmiddlefrontal_9)
6745	0.80676	(Left-Putamen)(lh.lateralorbitofrontal_4)(lh.lateralorbitofrontal_7)(lh.medialorbitofrontal_2)(lh.rostralmiddlefrontal_12)
6746	0.80676	(Left-Putamen)(lh.medialorbitofrontal_2)(lh.medialorbitofrontal_4)(lh.rostralanteriorcingulate_1)
6747	0.80676	(Left-Putamen)(lh.medialorbitofrontal_2)(lh.parstriangularis_3)(lh.rostralanteriorcingulate_1)(lh.rostralmiddlefrontal_12)(lh.rostralmiddlefrontal_9)
6748	0.80676	(Left-Putamen)(lh.rostralanteriorcingulate_1)(lh.rostralmiddlefrontal_12)(lh.rostralmiddlefrontal_9)(lh.superiorfrontal_4)
6749	0.80676	(Left-Putamen)(lh.rostralanteriorcingulate_1)(lh.rostralmiddlefrontal_4)(lh.rostralmiddlefrontal_9)(lh.superiorfrontal_4)
6750	0.80676	(lh.inferiorparietal_4)(lh.lateraloccipital_7)(lh.lateraloccipital_9)

6751	0.80676	(lh.rostralmiddlefrontal_4)(lh.rostralmiddlefrontal_5)(lh.rostralmiddlefrontal_7)
6752	0.80676	(lh.superiorfrontal_12)(lh.superiorfrontal_14)(lh.superiorfrontal_15)(lh.superiorfrontal_8)
6753	0.80676	(lh.superiorfrontal_12)(lh.superiorfrontal_15)(lh.superiorfrontal_8)
6754	0.80676	(lh.superiorfrontal_4)(lh.superiorfrontal_6)(lh.superiorfrontal_9)
6755	0.80676	(lh.supramarginal_1)(lh.supramarginal_3)
6756	0.80676	(rh.bankssts_1)(rh.fusiform_6)
6757	0.80676	(rh.caudalmiddlefrontal_4)(rh.precentral_10)(rh.precentral_7)
6758	0.80676	(rh.inferiorparietal_11)(rh.inferiorparietal_12)(rh.inferiorparietal_8)
6759	0.80676	(rh.insula_1)(rh.supramarginal_7)(rh.supramarginal_8)(rh.supramarginal_9)
6760	0.80676	(rh.superiorfrontal_13)(rh.superiorfrontal_5)(rh.superiorfrontal_7)(rh.superiorfrontal_9)
6761	0.80676	(Right-Amygdala)(Right-Hippocampus)(rh.insula_2)(rh.insula_4)
6762	0.80676	(Right-Amygdala)(Right-Hippocampus)(Right-Putamen)(rh.insula_2)(rh.insula_4)
6763	0.80676	(Right-Caudate)(rh.parstriangularis_1)(rh.rostralmiddlefrontal_5)
6764	0.80676	(Right-Caudate)(rh.precentral_13)(rh.precentral_8)
6765	0.80676	(Right-Caudate)(Right-Pallidum)(rh.caudalmiddlefrontal_4)(rh.parsopercularis_4)
6766	0.80676	(Right-Caudate)(Right-Pallidum)(Right-Putamen)(rh.caudalmiddlefrontal_4)(rh.parsopercularis_4)
6767	0.80676	(Right-Caudate)(Right-Putamen)(rh.caudalmiddlefrontal_4)(rh.precentral_13)(rh.precentral_7)
6768	0.80676	(Right-Caudate)(Right-Putamen)(rh.caudalmiddlefrontal_5)(rh.superiorfrontal_7)
6769	0.80676	(Right-Caudate)(Right-Putamen)(rh.lateralorbitofrontal_2)(rh.lateralorbitofrontal_6)(rh.parstriangularis_1)
6770	0.80676	(Right-Hippocampus)(rh.bankssts_2)(rh.inferiortemporal_2)
6771	0.80676	(Right-Hippocampus)(rh.fusiform_8)(rh.middletemporal_9)
6772	0.80676	(Right-Pallidum)(rh.caudalmiddlefrontal_4)(rh.parsopercularis_4)
6773	0.80676	(Right-Pallidum)(Right-Putamen)(rh.caudalmiddlefrontal_4)(rh.parsopercularis_4)
6774	0.80676	(Right-Putamen)(rh.rostralmiddlefrontal_5)(rh.superiorfrontal_7)
6775	0.80435	(Left-Caudate)(Left-Putamen)(Left-Thalamus-Proper)(lh.precentral_7)(lh.precentral_9)
6776	0.80435	(Left-Caudate)(Left-Putamen)(lh.rostralanteriorcingulate_2)(lh.rostralmiddlefrontal_9)(lh.superiorfrontal_4)
6777	0.80435	(Left-Caudate)(lh.caudalanteriorcingulate_2)(lh.superiorfrontal_10)(lh.superiorfrontal_14)
6778	0.80435	(Left-Caudate)(lh.caudalmiddlefrontal_3)(lh.caudalmiddlefrontal_4)(lh.caudalmiddlefrontal_6)
6779	0.80435	(Left-Caudate)(lh.precentral_11)(lh.precentral_4)(lh.precentral_9)
6780	0.80435	(Left-Hippocampus)(Left-Thalamus-Proper)(lh.superiortemporal_3)
6781	0.80435	(Left-Putamen)(lh.caudalmiddlefrontal_3)(lh.insula_6)(lh.parsopercularis_3)(lh.rostralmiddlefrontal_1)
6782	0.80435	(Left-Putamen)(lh.insula_1)(lh.insula_2)(lh.insula_3)
6783	0.80435	(Left-Putamen)(lh.insula_6)(lh.parsopercularis_1)(lh.parsopercularis_3)(lh.parstriangularis_3)(lh.rostralmiddlefrontal_1)
6784	0.80435	(Left-Putamen)(lh.lateralorbitofrontal_4)(lh.lateralorbitofrontal_7)(lh.parstriangularis_3)(lh.rostralanteriorcingulate_1)
6785	0.80435	(Left-Putamen)(lh.lateralorbitofrontal_4)(lh.parstriangularis_3)(lh.rostralanteriorcingulate_1)(lh.rostralmiddlefrontal_12)(lh.rostralmiddlefrontal_9)
6786	0.80435	(lh.bankssts_2)(lh.bankssts_3)(lh.fusiform_5)
6787	0.80435	(lh.cuneus_1)(lh.cuneus_2)(lh.pericalcarine_1)(lh.superiorparietal_14)
6788	0.80435	(lh.insula_1)(lh.insula_2)(lh.insula_3)
6789	0.80435	(lh.lateralorbitofrontal_4)(lh.lateralorbitofrontal_6)(lh.rostralmiddlefrontal_12)(lh.rostralmiddlefrontal_6)
6790	0.80435	(lh.parstriangularis_3)(lh.rostralmiddlefrontal_1)(lh.rostralmiddlefrontal_7)
6791	0.80435	(lh.precuneus_6)(lh.precuneus_8)(lh.precuneus_9)(lh.superiorparietal_6)
6792	0.80435	(lh.superiorfrontal_11)(lh.superiorfrontal_12)(lh.superiorfrontal_14)(lh.superiorfrontal_4)(lh.superiorfrontal_8)
6793	0.80435	(lh.superiorfrontal_4)(lh.superiorfrontal_6)(lh.superiorfrontal_7)(lh.superiorfrontal_9)
6794	0.80435	(rh.inferiorparietal_10)(rh.inferiorparietal_4)(rh.insula_2)
6795	0.80435	(rh.inferiortemporal_3)(rh.middletemporal_6)(rh.middletemporal_7)
6796	0.80435	(rh.insula_1)(rh.precentral_13)(rh.precentral_7)
6797	0.80435	(rh.lateralorbitofrontal_4)(rh.lateralorbitofrontal_7)(rh.medialorbitofrontal_2)
6798	0.80435	(rh.paracentral_6)(rh.precentral_13)(rh.superiorparietal_2)
6799	0.80435	(rh.rostralanteriorcingulate_2)(rh.rostralmiddlefrontal_5)
6800	0.80435	(rh.rostralmiddlefrontal_12)(rh.rostralmiddlefrontal_3)(rh.rostralmiddlefrontal_6)(rh.rostralmiddlefrontal_8)

6801	0.80435	(rh.rostralmiddlefrontal_5)(rh.superiorfrontal_2)(rh.superiorfrontal_7)
6802	0.80435	(rh.superiorfrontal_11)(rh.superiorfrontal_13)(rh.superiorfrontal_7)(rh.superiorfrontal_8)
6803	0.80435	(Right-Putamen)(rh.caudalmiddlefrontal_5)(rh.precentral_13)
6804	0.80435	(Right-Putamen)(rh.insula_1)(rh.precentral_13)(rh.precentral_7)
6805	0.80193	(Left-Amygdala)(lh.fusiform_8)(lh.insula_4)
6806	0.80193	(Left-Caudate)(lh.medialorbitofrontal_2)(lh.superiorfrontal_3)(lh.superiorfrontal_4)
6807	0.80193	(Left-Putamen)(lh.caudalanteriorcingulate_2)(lh.superiorfrontal_8)
6808	0.80193	(lh.bankssts_2)(lh.fusiform_7)(lh.middletemporal_4)
6809	0.80193	(lh.caudalanteriorcingulate_1)(lh.rostralanteriorcingulate_2)(lh.superiorfrontal_11)
6810	0.80193	(lh.precentral_3)(lh.precentral_4)
6811	0.80193	(lh.precuneus_6)(lh.precuneus_9)(lh.superiorparietal_5)(lh.superiorparietal_6)
6812	0.80193	(rh.caudalanteriorcingulate_2)(rh.superiorfrontal_9)
6813	0.80193	(rh.inferiorparietal_10)(rh.lateraloccipital_10)(rh.lateraloccipital_8)(rh.lingual_7)
6814	0.80193	(rh.postcentral_5)(rh.precentral_11)
6815	0.80193	(Right-Caudate)(rh.medialorbitofrontal_2)(rh.rostralmiddlefrontal_11)
