# Supplementary material for: The frequent complete subgraphs in the human connectome
Source: PLoS One. 2020 Aug 20;15(8):e0236883. doi: 10.1371/journal.pone.0236883 (PMC7444532; doi:10.1371/journal.pone.0236883)
Supplement: S2 Table — (PDF) [file pone.0236883.s002.pdf]

p-value	Holm-Bonferroni	frequency_male	frequency_female	
0	1.00E-05	0.65143	0.89958	(Right-Caudate)(Right-Putamen)(rh.superiorparietal_1)(rh.supramarginal_9)
0	1.00E-05	0.58857	0.85356	(Right-Putamen)(Right-Thalamus-Proper)(rh.precentral_6)
0	1.00E-05	0.62286	0.87448	(Right-Caudate)(Right-Putamen)(rh.insula_1)(rh.superiorparietal_1)(rh.supramarginal_9)
0	1.00E-05	0.63429	0.87448	(Right-Caudate)(rh.insula_1)(rh.superiorparietal_1)(rh.supramarginal_9)
0	1.00E-05	0.70286	0.91632	(Right-Caudate)(Right-Putamen)(rh.superiorparietal_1)
0	1.00E-05	0.61538	0.85356	(Right-Thalamus-Proper)(rh.precentral_6)
0	1.00E-05	0.68	0.8954	(Right-Thalamus-Proper)(rh.insula_1)(rh.precentral_7)
0	1.00E-05	0.68	0.8954	(Right-Putamen)(Right-Thalamus-Proper)(rh.insula_1)(rh.precentral_7)
0	1.00E-05	0.67429	0.89121	(Right-Caudate)(Right-Putamen)(rh.insula_1)(rh.superiorparietal_1)
0	1.00E-05	0.57143	0.8159	(Right-Pallidum)(Right-Thalamus-Proper)(rh.precentral_6)
0	1.00E-05	0.57143	0.8159	(Right-Pallidum)(Right-Putamen)(Right-Thalamus-Proper)(rh.precentral_6)
0	1.00E-05	0.58286	0.82427	(Right-Caudate)(Right-Putamen)(Right-Thalamus-Proper)(rh.precentral_6)
0	1.00E-05	0.62637	0.85356	(Left-Thalamus-Proper)(lh.superiorparietal_5)
0	1.00E-05	0.58857	0.82427	(Right-Caudate)(Right-Thalamus-Proper)(rh.precentral_6)
0	1.00E-05	0.73714	0.92469	(Left-Caudate)(Left-Putamen)(Left-Thalamus-Proper)(lh.supramarginal_1)
0	1.00E-05	0.68571	0.89121	(Right-Caudate)(rh.insula_1)(rh.superiorparietal_1)
0	1.00E-05	0.66857	0.87866	(Right-Caudate)(Right-Thalamus-Proper)(rh.insula_1)(rh.precentral_7)
0	1.00E-05	0.66857	0.87866	(Right-Caudate)(Right-Putamen)(Right-Thalamus-Proper)(rh.insula_1)(rh.precentral_7)
0	1.00E-05	0.68132	0.88703	(lh.inferiorparietal_5)(lh.inferiorparietal_8)
0	1.00E-05	0.70286	0.89958	(Right-Caudate)(Right-Thalamus-Proper)(rh.precentral_7)
0	1.00E-05	0.70286	0.89958	(Right-Caudate)(Right-Putamen)(Right-Thalamus-Proper)(rh.precentral_7)
0	1.00E-05	0.62286	0.841	(Right-Caudate)(Right-Thalamus-Proper)(rh.precentral_11)(rh.precentral_7)
0	1.00E-05	0.63429	0.84937	(Right-Caudate)(Right-Putamen)(rh.precentral_13)(rh.superiorparietal_1)
0	1.00E-05	0.71429	0.90377	(Right-Putamen)(Right-Thalamus-Proper)(rh.precentral_7)
0	1.00E-05	0.6044	0.82427	(Right-Caudate)(rh.superiorparietal_1)(rh.supramarginal_8)(rh.supramarginal_9)
0	1.00E-05	0.61538	0.83264	(Right-Caudate)(rh.superiorparietal_1)(rh.supramarginal_8)
0	1.00E-05	0.69714	0.89121	(Right-Thalamus-Proper)(rh.insula_2)(rh.precentral_7)
0	1.00E-05	0.69714	0.89121	(Right-Putamen)(Right-Thalamus-Proper)(rh.insula_2)(rh.precentral_7)
0	1.00E-05	0.81714	0.96234	(Left-Caudate)(Left-Putamen)(lh.supramarginal_1)
0	1.00E-05	0.61538	0.82845	(Left-Thalamus-Proper)(lh.superiorparietal_2)
0	1.00E-05	0.61538	0.82845	(Left-Thalamus-Proper)(lh.postcentral_8)(lh.precentral_9)
0	1.00E-05	0.61714	0.82845	(Right-Caudate)(Right-Putamen)(Right-Thalamus-Proper)(rh.precentral_11)(rh.precentral_7)
0	1.00E-05	0.65143	0.85356	(Right-Thalamus-Proper)(rh.precentral_11)(rh.precentral_7)
0	1.00E-05	0.66286	0.86192	(Right-Putamen)(Right-Thalamus-Proper)(rh.insula_1)(rh.insula_2)(rh.precentral_7)
0	1.00E-05	0.76923	0.93305	(Left-Caudate)(Left-Thalamus-Proper)(lh.supramarginal_1)
0	1.00E-05	0.73143	0.90795	(Left-Caudate)(Left-Putamen)(Left-Thalamus-Proper)(lh.insula_1)(lh.supramarginal_1)
0	1.00E-05	0.72527	0.90377	(Right-Thalamus-Proper)(rh.precentral_7)
0	1.00E-05	0.67033	0.86192	(Right-Thalamus-Proper)(rh.insula_1)(rh.insula_2)(rh.precentral_7)
0	1.00E-05	0.73626	0.90795	(Right-Caudate)(rh.superiorparietal_1)(rh.supramarginal_9)
0	1.00E-05	0.72527	0.89958	(Left-Thalamus-Proper)(lh.caudalmiddlefrontal_3)
0	1.00E-05	0.64571	0.841	(Right-Putamen)(Right-Thalamus-Proper)(rh.precentral_11)(rh.precentral_7)
0	1.00E-05	0.7033	0.88285	(rh.bankssts_2)(rh.fusiform_5)
1.00E-05	1.00E-05	0.60571	0.80753	(Right-Caudate)(Right-Hippocampus)(rh.superiorparietal_1)
1.00E-05	1.00E-05	0.62857	0.82427	(Right-Putamen)(Right-Thalamus-Proper)(rh.insula_1)(rh.superiorparietal_1)
1.00E-05	1.00E-05	0.63429	0.82845	(Right-Thalamus-Proper)(rh.insula_1)(rh.superiorparietal_1)
1.00E-05	1.00E-05	0.71429	0.88703	(Left-Caudate)(lh.postcentral_8)(lh.precentral_9)
1.00E-05	1.00E-05	0.68571	0.86611	(Right-Caudate)(Right-Thalamus-Proper)(rh.precentral_13)(rh.precentral_7)
1.00E-05	1.00E-05	0.68571	0.86611	(Right-Caudate)(Right-Putamen)(Right-Thalamus-Proper)(rh.precentral_13)(rh.precentral_7)
1.00E-05	1.00E-05	0.85143	0.97071	(Left-Caudate)(lh.supramarginal_1)

1.00E-05	1.00E-05	0.63429	0.82427	(Right-Thalamus-Proper)(rh.precentral_11)(rh.precentral_13)(rh.precentral_7)
1.00E-05	1.00E-05	0.65143	0.83682	(Right-Caudate)(Right-Pallidum)(Right-Thalamus-Proper)(rh.precentral_7)
1.00E-05	1.00E-05	0.65143	0.83682	(Right-Caudate)(Right-Pallidum)(Right-Putamen)(Right-Thalamus-Proper)(rh.precentral_7)
1.00E-05	1.00E-05	0.65714	0.841	(Right-Putamen)(Right-Thalamus-Proper)(rh.superiorparietal_1)
2.00E-05	1.00E-05	0.69714	0.87029	(Right-Thalamus-Proper)(rh.precentral_13)(rh.precentral_7)
2.00E-05	1.00E-05	0.69714	0.87029	(Left-Pallidum)(Left-Thalamus-Proper)(lh.caudalmiddlefrontal_3)
2.00E-05	1.00E-05	0.69714	0.87029	(Left-Pallidum)(Left-Putamen)(Left-Thalamus-Proper)(lh.caudalmiddlefrontal_3)
2.00E-05	1.00E-05	0.69714	0.87029	(Left-Caudate)(Left-Pallidum)(Left-Thalamus-Proper)(lh.caudalmiddlefrontal_3)
2.00E-05	1.00E-05	0.69714	0.87029	(Right-Putamen)(Right-Thalamus-Proper)(rh.precentral_13)(rh.precentral_7)
2.00E-05	1.00E-05	0.69714	0.87029	(Left-Caudate)(Left-Pallidum)(Left-Putamen)(Left-Thalamus-Proper)(lh.caudalmiddlefrontal_3)
2.00E-05	1.00E-05	0.62637	0.8159	(lh.postcentral_14)(lh.precentral_14)
2.00E-05	1.00E-05	0.63736	0.82427	(rh.precentral_1)(rh.supramarginal_9)
2.00E-05	1.00E-05	0.81143	0.94561	(Left-Caudate)(Left-Putamen)(lh.insula_1)(lh.supramarginal_1)
2.00E-05	1.00E-05	0.65714	0.83682	(Right-Pallidum)(Right-Thalamus-Proper)(rh.precentral_7)
2.00E-05	1.00E-05	0.65714	0.83682	(Right-Pallidum)(Right-Putamen)(Right-Thalamus-Proper)(rh.precentral_7)
2.00E-05	1.00E-05	0.78022	0.92469	(Right-Caudate)(rh.superiorparietal_1)
3.00E-05	1.00E-05	0.79429	0.93305	(Right-Caudate)(rh.precentral_15)
3.00E-05	1.00E-05	0.76923	0.91632	(Left-Caudate)(Left-Thalamus-Proper)(lh.insula_1)(lh.supramarginal_1)
3.00E-05	1.00E-05	0.65934	0.83682	(lh.paracentral_3)(lh.precentral_4)
4.00E-05	1.00E-05	0.74725	0.89958	(lh.precuneus_10)(lh.superiorparietal_5)
4.00E-05	1.00E-05	0.74857	0.89958	(Left-Caudate)(Left-Thalamus-Proper)(lh.caudalmiddlefrontal_3)
4.00E-05	1.00E-05	0.74857	0.89958	(Left-Putamen)(Left-Thalamus-Proper)(lh.caudalmiddlefrontal_3)
4.00E-05	1.00E-05	0.74857	0.89958	(Left-Caudate)(Left-Putamen)(Left-Thalamus-Proper)(lh.caudalmiddlefrontal_3)
5.00E-05	1.00E-05	0.69231	0.85774	(Left-Thalamus-Proper)(lh.supramarginal_9)
7.00E-05	1.00E-05	0.67429	0.841	(Left-Thalamus-Proper)(lh.insula_1)(lh.supramarginal_9)
9.00E-05	1.00E-05	0.65143	0.82008	(Left-Caudate)(Left-Hippocampus)(Left-Putamen)(lh.supramarginal_1)
0.0001	1.00E-05	0.66286	0.82845	(Left-Putamen)(Left-Thalamus-Proper)(lh.postcentral_8)(lh.precentral_9)
0.00012	1.00E-05	0.77143	0.90795	(Left-Caudate)(Left-Thalamus-Proper)(lh.postcentral_3)
0.00013	1.00E-05	0.7033	0.85774	(Right-Caudate)(rh.precentral_13)(rh.superiorparietal_1)
0.00014	1.00E-05	0.79121	0.9205	(Right-Caudate)(rh.precentral_13)(rh.precentral_15)
0.00016	1.00E-05	0.84571	0.95397	(Left-Caudate)(lh.insula_1)(lh.supramarginal_1)
0.00018	1.00E-05	0.94286	1	(Left-Thalamus-Proper)(lh.precentral_11)
0.00018	1.00E-05	0.94286	1	(Left-Caudate)(Left-Thalamus-Proper)(lh.precentral_11)
0.00018	1.00E-05	0.94286	1	(Left-Putamen)(Left-Thalamus-Proper)(lh.precentral_11)
0.00018	1.00E-05	0.94286	1	(Left-Caudate)(Left-Putamen)(Left-Thalamus-Proper)(lh.precentral_11)
0.00019	1.00E-05	0.75824	0.8954	(lh.fusiform_2)(lh.fusiform_5)(lh.lingual_8)
0.00019	1.00E-05	0.84	0.94979	(Left-Caudate)(Left-Putamen)(Left-Thalamus-Proper)(lh.postcentral_8)
0.0002	1.00E-05	0.64	0.80335	(Left-Putamen)(lh.precentral_16)(lh.supramarginal_1)
0.0002	1.00E-05	0.69231	0.84519	(Left-Thalamus-Proper)(lh.supramarginal_4)
0.0003	1.00E-05	0.67033	0.82427	(Right-Thalamus-Proper)(rh.superiorparietal_1)(rh.supramarginal_9)
0.00033	1.00E-05	0.70857	0.85356	(lh.inferiorparietal_5)(lh.inferiorparietal_8)(lh.inferiorparietal_9)
0.00034	1.00E-05	0.71429	0.85774	(lh.bankssts_2)(lh.fusiform_7)(lh.middletemporal_4)
0.00034	1.00E-05	0.84571	0.94979	(Left-Caudate)(Left-Thalamus-Proper)(lh.postcentral_8)
0.00036	1.00E-05	0.65714	0.81172	(Left-Caudate)(lh.postcentral_3)(lh.postcentral_8)
0.00039	1.00E-05	0.92571	0.99163	(Left-Caudate)(Left-Putamen)(lh.postcentral_8)
0.00039	1.00E-05	0.94857	1	(lh.paracentral_5)(lh.superiorfrontal_14)
0.00042	1.00E-05	0.83429	0.94142	(Left-Putamen)(Left-Thalamus-Proper)(lh.supramarginal_1)
0.00042	1.00E-05	0.83429	0.94142	(Left-Putamen)(Left-Thalamus-Proper)(lh.insula_1)(lh.supramarginal_1)
0.00044	1.00E-05	0.79121	0.91213	(Left-Putamen)(lh.postcentral_8)(lh.precentral_9)
0.00044	1.00E-05	0.68571	0.83264	(Left-Hippocampus)(lh.bankssts_3)(lh.insula_2)

0.00048	1.00E-05	0.78022	0.90377	(lh.fusiform_2)(lh.fusiform_5)
0.00053	1.00E-05	0.71429	0.85356	(Right-Thalamus-Proper)(rh.superiorparietal_1)
0.00053	1.00E-05	0.71429	0.85356	(Left-Putamen)(lh.postcentral_14)(lh.supramarginal_1)
0.00054	1.00E-05	0.72527	0.86192	(Left-Putamen)(lh.postcentral_14)(lh.precentral_16)
0.00055	1.00E-05	0.85714	0.95397	(Left-Thalamus-Proper)(lh.supramarginal_1)
0.00055	1.00E-05	0.85714	0.95397	(Left-Thalamus-Proper)(lh.insula_1)(lh.supramarginal_1)
0.00058	1.00E-05	0.73714	0.87029	(rh.caudalmiddlefrontal_4)(rh.rostralmiddlefrontal_1)(rh.rostralmiddlefrontal_2)(rh.rostralmiddlefrontal_3)
0.00059	1.00E-05	0.74286	0.87448	(Left-Caudate)(Left-Putamen)(Left-Thalamus-Proper)(lh.postcentral_8)(lh.precentral_11)
0.00061	1.00E-05	0.76	0.88703	(Left-Caudate)(Left-Putamen)(lh.postcentral_8)(lh.precentral_9)
0.00062	1.00E-05	0.85143	0.94979	(Left-Putamen)(lh.precentral_16)
0.00079	2.00E-05	0.7033	0.841	(lh.postcentral_14)(lh.supramarginal_9)
0.00079	2.00E-05	0.7033	0.841	(Left-Putamen)(lh.parsopercularis_1)(lh.precentral_16)
0.00079	2.00E-05	0.70857	0.84519	(lh.inferiorparietal_5)(lh.inferiorparietal_6)(lh.inferiorparietal_8)
0.0008	2.00E-05	0.78022	0.89958	(lh.fusiform_1)(lh.fusiform_2)(lh.fusiform_5)
0.00084	2.00E-05	0.72527	0.85774	(lh.paracentral_5)(lh.superiorfrontal_14)(lh.superiorfrontal_17)
0.00085	2.00E-05	0.75824	0.88285	(Left-Thalamus-Proper)(lh.postcentral_8)(lh.precentral_11)
0.00085	2.00E-05	0.73626	0.86611	(rh.postcentral_1)(rh.supramarginal_8)
0.00086	2.00E-05	0.74725	0.87448	(lh.fusiform_1)(lh.fusiform_2)(lh.fusiform_5)(lh.lateraloccipital_9)
0.00094	2.00E-05	0.81143	0.9205	(Left-Thalamus-Proper)(lh.superiorparietal_3)
0.00096	2.00E-05	0.74857	0.87448	(Left-Caudate)(Left-Thalamus-Proper)(lh.postcentral_8)(lh.precentral_11)
0.00099	2.00E-05	0.86286	0.95397	(Left-Caudate)(lh.postcentral_3)
0.00101	2.00E-05	0.78857	0.90377	(Right-Pallidum)(Right-Putamen)(rh.parsopercularis_4)
0.00101	2.00E-05	0.77143	0.89121	(Left-Putamen)(lh.postcentral_14)
0.00119	2.00E-05	0.85143	0.94561	(rh.caudalanteriorcingulate_3)(rh.caudalmiddlefrontal_4)
0.00119	2.00E-05	0.85143	0.94561	(Right-Caudate)(rh.caudalanteriorcingulate_3)(rh.caudalmiddlefrontal_4)
0.00126	2.00E-05	0.79121	0.90377	(Right-Pallidum)(rh.parsopercularis_4)
0.00126	2.00E-05	0.79121	0.90377	(Right-Putamen)(rh.precentral_5)(rh.precentral_8)
0.00128	2.00E-05	0.84571	0.94142	(Left-Thalamus-Proper)(lh.precentral_11)(lh.precentral_9)
0.00131	2.00E-05	0.78022	0.8954	(Left-Hippocampus)(lh.parahippocampal_1)(lh.parahippocampal_2)
0.00134	2.00E-05	0.76923	0.88703	(rh.fusiform_1)(rh.fusiform_2)(rh.inferiorparietal_10)
0.00134	2.00E-05	0.74725	0.87029	(lh.lateraloccipital_3)(lh.lateraloccipital_4)
0.00135	2.00E-05	0.75824	0.87866	(lh.fusiform_2)(lh.fusiform_5)(lh.lateraloccipital_9)
0.00141	2.00E-05	0.88	0.96234	(Left-Caudate)(lh.caudalanteriorcingulate_2)(lh.posteriorcingulate_1)(lh.posteriorcingulate_2)
0.00142	2.00E-05	0.68	0.8159	(Left-Thalamus-Proper)(lh.insula_1)(lh.superiorparietal_5)
0.00146	2.00E-05	0.67033	0.80753	(lh.isthmuscingulate_2)(lh.precuneus_10)(lh.superiorparietal_5)
0.0016	2.00E-05	0.87429	0.95816	(Left-Putamen)(Left-Thalamus-Proper)(lh.postcentral_8)
0.00163	2.00E-05	0.69714	0.82845	(lh.paracentral_3)(lh.paracentral_5)(lh.precentral_4)
0.00164	2.00E-05	0.78857	0.89958	(Right-Caudate)(Right-Pallidum)(rh.parsopercularis_4)
0.00166	2.00E-05	0.69231	0.82427	(Right-Putamen)(rh.postcentral_8)(rh.supramarginal_1)
0.00169	2.00E-05	0.93714	0.99163	(Left-Caudate)(lh.postcentral_8)
0.00169	2.00E-05	0.93714	0.99163	(Left-Thalamus-Proper)(lh.insula_1)(lh.precentral_11)
0.00169	2.00E-05	0.93714	0.99163	(Left-Putamen)(Left-Thalamus-Proper)(lh.insula_1)(lh.precentral_11)
0.00182	2.00E-05	0.96	1	(rh.fusiform_1)(rh.fusiform_2)
0.00185	2.00E-05	0.71429	0.841	(lh.isthmuscingulate_2)(lh.superiorparietal_6)
0.00185	2.00E-05	0.71429	0.841	(Left-Putamen)(lh.insula_3)(lh.precentral_16)
0.00191	2.00E-05	0.89714	0.97071	(Left-Hippocampus)(lh.bankssts_2)
0.002	2.00E-05	0.73626	0.85774	(lh.fusiform_2)(lh.fusiform_5)(lh.lateraloccipital_9)(lh.lingual_8)
0.00205	2.00E-05	0.79121	0.89958	(lh.precuneus_9)(lh.superiorparietal_11)(lh.superiorparietal_6)
0.00205	2.00E-05	0.79121	0.89958	(Right-Caudate)(Right-Pallidum)(Right-Putamen)(rh.parsopercularis_4)
0.00209	2.00E-05	0.75824	0.87448	(lh.fusiform_1)(lh.fusiform_2)(lh.fusiform_5)(lh.lingual_8)

0.00209	2.00E-05	0.78022	0.89121	(Left-Caudate)(lh.caudalmiddlefrontal_1)(lh.rostralmiddlefrontal_1)
0.00214	2.00E-05	0.73714	0.85774	(Right-Pallidum)(rh.caudalmiddlefrontal_4)(rh.parsopercularis_4)
0.00214	2.00E-05	0.73714	0.85774	(Right-Pallidum)(Right-Putamen)(rh.caudalmiddlefrontal_4)(rh.parsopercularis_4)
0.00214	2.00E-05	0.73714	0.85774	(Right-Caudate)(Right-Pallidum)(rh.caudalmiddlefrontal_4)(rh.parsopercularis_4)
0.00214	2.00E-05	0.73714	0.85774	(Right-Caudate)(Right-Pallidum)(Right-Putamen)(rh.caudalmiddlefrontal_4)(rh.parsopercularis_4)
0.00231	2.00E-05	0.84571	0.93724	(Left-Putamen)(Left-Thalamus-Proper)(lh.precentral_11)(lh.precentral_9)
0.00231	2.00E-05	0.84571	0.93724	(Right-Caudate)(Right-Hippocampus)(Right-Putamen)(Right-Thalamus-Proper)(rh.supramarginal_9)
0.00251	2.00E-05	0.77143	0.88285	(Left-Putamen)(Left-Thalamus-Proper)(lh.postcentral_8)(lh.precentral_11)
0.00262	2.00E-05	0.78857	0.8954	(lh.inferiorparietal_5)(lh.lingual_8)
0.00272	3.00E-05	0.71429	0.83682	(rh.bankssts_2)(rh.supramarginal_8)
0.00284	3.00E-05	0.88	0.95816	(Left-Thalamus-Proper)(lh.postcentral_8)
0.00286	3.00E-05	0.72527	0.84519	(rh.lateraloccipital_2)(rh.pericalcarine_4)
0.00307	3.00E-05	0.73143	0.84937	(Left-Putamen)(lh.insula_3)(lh.supramarginal_1)
0.00317	3.00E-05	0.75824	0.87029	(Left-Caudate)(lh.parsopercularis_1)(lh.rostralmiddlefrontal_1)
0.00323	3.00E-05	0.76923	0.87866	(Left-Putamen)(lh.superiorparietal_5)
0.00323	3.00E-05	0.76923	0.87866	(Right-Hippocampus)(rh.superiorparietal_1)(rh.supramarginal_9)
0.0033	3.00E-05	0.74286	0.85774	(lh.precuneus_10)(lh.precuneus_6)(lh.superiorparietal_5)
0.00373	3.00E-05	0.85714	0.94142	(rh.caudalmiddlefrontal_4)(rh.rostralmiddlefrontal_1)(rh.rostralmiddlefrontal_2)
0.00388	3.00E-05	0.85143	0.93724	(Right-Caudate)(Right-Hippocampus)(Right-Thalamus-Proper)(rh.supramarginal_9)
0.00393	3.00E-05	0.71429	0.83264	(Left-Thalamus-Proper)(lh.posteriorcingulate_1)(lh.posteriorcingulate_2)
0.00393	3.00E-05	0.71429	0.83264	(Left-Caudate)(Left-Thalamus-Proper)(lh.posteriorcingulate_1)(lh.posteriorcingulate_2)
0.00393	3.00E-05	0.96571	1	(lh.posteriorcingulate_1)(lh.posteriorcingulate_3)
0.00393	3.00E-05	0.96571	1	(Left-Caudate)(lh.posteriorcingulate_1)(lh.posteriorcingulate_3)
0.00401	3.00E-05	0.84571	0.93305	(Left-Caudate)(Left-Thalamus-Proper)(lh.precentral_11)(lh.precentral_9)
0.00419	3.00E-05	0.8	0.89958	(rh.inferiorparietal_1)(rh.supramarginal_8)
0.0042	3.00E-05	0.95429	0.99582	(rh.fusiform_1)(rh.fusiform_2)(rh.lateraloccipital_10)
0.00427	3.00E-05	0.81714	0.91213	(Left-Caudate)(lh.paracentral_5)(lh.postcentral_3)
0.00436	4.00E-05	0.73626	0.84937	(Left-Putamen)(lh.insula_1)(lh.postcentral_14)
0.00436	4.00E-05	0.73626	0.84937	(rh.caudalmiddlefrontal_5)(rh.rostralmiddlefrontal_1)(rh.rostralmiddlefrontal_2)
0.00453	4.00E-05	0.89143	0.96234	(lh.caudalanteriorcingulate_2)(lh.posteriorcingulate_1)(lh.posteriorcingulate_2)
0.00473	4.00E-05	0.75824	0.86611	(lh.caudalmiddlefrontal_3)(lh.caudalmiddlefrontal_6)
0.00486	4.00E-05	0.76923	0.87448	(lh.rostralanteriorcingulate_2)(lh.rostralmiddlefrontal_4)(lh.superiorfrontal_10)
0.00487	4.00E-05	0.69231	0.81172	(Left-Thalamus-Proper)(lh.postcentral_8)(lh.precentral_11)(lh.precentral_4)
0.00497	4.00E-05	0.88571	0.95816	(Right-Caudate)(Right-Hippocampus)(Right-Putamen)(rh.supramarginal_9)
0.005	4.00E-05	0.79121	0.89121	(rh.rostralmiddlefrontal_1)(rh.rostralmiddlefrontal_2)(rh.rostralmiddlefrontal_3)
0.005	4.00E-05	0.79121	0.89121	(Left-Caudate)(lh.rostralanteriorcingulate_2)(lh.superiorfrontal_10)(lh.superiorfrontal_4)
0.00533	4.00E-05	0.70857	0.82427	(lh.precuneus_10)(lh.precuneus_9)(lh.superiorparietal_5)
0.00538	5.00E-05	0.76	0.86611	(rh.inferiorparietal_1)(rh.inferiorparietal_4)(rh.supramarginal_8)
0.00559	5.00E-05	0.71429	0.82845	(lh.precuneus_6)(lh.superiorparietal_8)
0.00559	5.00E-05	0.71429	0.82845	(rh.lateraloccipital_6)(rh.pericalcarine_4)
0.00571	5.00E-05	0.87429	0.94979	(rh.bankssts_2)(rh.insula_2)
0.00594	5.00E-05	0.72527	0.83682	(lh.precentral_11)(lh.superiorparietal_3)
0.00594	5.00E-05	0.72527	0.83682	(lh.precentral_3)(lh.superiorfrontal_15)
0.00594	5.00E-05	0.72527	0.83682	(Right-Thalamus-Proper)(rh.precentral_15)
0.00594	5.00E-05	0.72527	0.83682	(Right-Putamen)(rh.precentral_4)
0.00594	5.00E-05	0.72527	0.83682	(rh.lateraloccipital_8)(rh.lingual_2)(rh.lingual_4)
0.00594	5.00E-05	0.72527	0.83682	(rh.fusiform_1)(rh.fusiform_2)(rh.inferiorparietal_10)(rh.lateraloccipital_8)
0.00594	5.00E-05	0.72527	0.83682	(lh.fusiform_1)(lh.fusiform_2)(lh.fusiform_5)(lh.lateraloccipital_9)(lh.lingual_8)
0.00643	6.00E-05	0.85714	0.93724	(Left-Pallidum)(lh.caudalmiddlefrontal_3)
0.00643	6.00E-05	0.85714	0.93724	(Left-Caudate)(Left-Pallidum)(lh.caudalmiddlefrontal_3)

0.00643	6.00E-05	0.85714	0.93724	(Left-Pallidum)(Left-Putamen)(lh.caudalmiddlefrontal_3)
0.00643	6.00E-05	0.85714	0.93724	(Left-Caudate)(Left-Pallidum)(Left-Putamen)(lh.caudalmiddlefrontal_3)
0.00665	7.00E-05	0.81143	0.90377	(rh.isthmuscingulate_1)(rh.superiorparietal_1)
0.00669	7.00E-05	0.84571	0.92887	(Left-Caudate)(Left-Putamen)(Left-Thalamus-Proper)(lh.precentral_11)(lh.precentral_9)
0.00679	8.00E-05	0.83429	0.9205	(lh.lateraloccipital_3)(lh.lateraloccipital_5)
0.00687	8.00E-05	0.93143	0.98326	(Left-Caudate)(lh.precentral_11)(lh.precentral_9)
0.0069	8.00E-05	0.75824	0.86192	(lh.paracentral_5)(lh.superiorfrontal_17)
0.0071	9.00E-05	0.70286	0.8159	(Left-Caudate)(Left-Putamen)(Left-Thalamus-Proper)(lh.insula_1)(lh.postcentral_8)(lh.precentral_11)
0.00717	9.00E-05	0.76923	0.87029	(Left-Caudate)(Left-Thalamus-Proper)(lh.postcentral_8)(lh.precentral_4)
0.00784	0.00013	0.71429	0.82427	(Right-Thalamus-Proper)(rh.supramarginal_8)
0.00784	0.00013	0.71429	0.82427	(Right-Caudate)(rh.superiorfrontal_11)(rh.superiorfrontal_14)
0.0081	0.00014	0.76571	0.86611	(Left-Caudate)(Left-Thalamus-Proper)(lh.posteriorcingulate_3)(lh.precentral_11)
0.00856	0.00018	0.97143	1	(Left-Caudate)(lh.posteriorcingulate_1)(lh.posteriorcingulate_2)
0.00859	0.00018	0.89143	0.95816	(Right-Caudate)(Right-Hippocampus)(rh.supramarginal_9)
0.0089	0.00022	0.73626	0.841	(lh.bankssts_3)(lh.insula_2)
0.0089	0.00022	0.73626	0.841	(Right-Hippocampus)(rh.fusiform_8)(rh.middletemporal_9)
0.0089	0.00023	0.73626	0.841	(Left-Caudate)(lh.lateralorbitofrontal_6)(lh.medialorbitofrontal_2)
0.00892	0.00023	0.96	0.99582	(rh.lateraloccipital_8)(rh.lateraloccipital_9)
0.00893	0.00024	0.78286	0.87866	(Left-Thalamus-Proper)(lh.insula_1)(lh.superiorparietal_3)
0.00944	0.0004	0.73714	0.841	(Left-Putamen)(Left-Thalamus-Proper)(lh.insula_1)(lh.postcentral_8)(lh.precentral_11)
0.0099	0.00143	0.75824	0.85774	(lh.inferiorparietal_1)(lh.lingual_6)
0.0099	0.00167	0.75824	0.85774	(Right-Putamen)(rh.parsopercularis_2)(rh.precentral_1)
0.0099	0.002	0.75824	0.85774	(Left-Caudate)(lh.postcentral_8)(lh.precentral_11)(lh.precentral_4)
0.00997	0.0025	0.87429	0.94561	(Right-Hippocampus)(rh.bankssts_2)(rh.insula_2)
0.00997	0.00333	0.87429	0.94561	(Right-Caudate)(Right-Putamen)(Right-Thalamus-Proper)(rh.supramarginal_9)
