# Supplementary material for: The frequent complete subgraphs in the human connectome
Source: PLoS One. 2020 Aug 20;15(8):e0236883. doi: 10.1371/journal.pone.0236883 (PMC7444532; doi:10.1371/journal.pone.0236883)
Supplement: S3 Table — In both S2 and S3 Tables a frequency cut-off 80% is applied to the larger frequency of the appearance in the sexes: only those significant differences are listed, where the larger of the frequencies of males and females are at least 80%. (PDF) [file pone.0236883.s003.pdf]

p-value	Holm-Bonferroni	frequency_male	frequency_female	
0	1.00E-05	0.91429	0.66154	(Left-Putamen)(lh.posteriorcingulate_4)
0	1.00E-05	0.81714	0.53556	(Right-Amygdala)(Right-Caudate)(Right-Pallidum)(rh.insula_2)
0	1.00E-05	0.81714	0.53556	(Right-Amygdala)(Right-Caudate)(Right-Pallidum)(Right-Thalamus-Proper)(rh.insula_2)
0	1.00E-05	0.81714	0.53556	(Right-Amygdala)(Right-Caudate)(Right-Pallidum)(Right-Putamen)(rh.insula_2)
0	1.00E-05	0.81714	0.53556	(Right-Amygdala)(Right-Caudate)(Right-Pallidum)(Right-Putamen)(Right-Thalamus-Proper)(rh.insula_2)
0	1.00E-05	0.81143	0.53138	(Right-Amygdala)(Right-Caudate)(Right-Hippocampus)(Right-Pallidum)(rh.insula_2)
0	1.00E-05	0.81143	0.53138	(Right-Amygdala)(Right-Caudate)(Right-Hippocampus)(Right-Pallidum)(Right-Putamen)(rh.insula_2)
0	1.00E-05	0.81143	0.53138	(Right-Amygdala)(Right-Caudate)(Right-Hippocampus)(Right-Pallidum)(Right-Thalamus-Proper)(rh.insula_2)
0	1.00E-05	0.81143	0.53138	(Right-Amygdala)(Right-Caudate)(Right-Hippocampus)(Right-Pallidum)(Right-Putamen)(Right-Thalamus-Proper)(rh.insula_2)
0	1.00E-05	0.86857	0.61506	(Right-Amygdala)(Right-Caudate)(Right-Putamen)(rh.insula_2)
0	1.00E-05	0.86857	0.61506	(Right-Amygdala)(Right-Caudate)(Right-Putamen)(Right-Thalamus-Proper)(rh.insula_2)
0	1.00E-05	0.82857	0.56485	(Right-Caudate)(Right-Pallidum)(rh.insula_2)(rh.precentral_13)
0	1.00E-05	0.82857	0.56485	(Right-Caudate)(Right-Pallidum)(Right-Putamen)(rh.insula_2)(rh.precentral_13)
0	1.00E-05	0.91429	0.68201	(Left-Caudate)(Left-Putamen)(lh.posteriorcingulate_4)
0	1.00E-05	0.86286	0.61088	(Right-Amygdala)(Right-Caudate)(Right-Hippocampus)(rh.insula_2)
0	1.00E-05	0.86286	0.61088	(Right-Amygdala)(Right-Caudate)(Right-Hippocampus)(Right-Thalamus-Proper)(rh.insula_2)
0	1.00E-05	0.86286	0.61088	(Right-Amygdala)(Right-Caudate)(Right-Hippocampus)(Right-Putamen)(rh.insula_2)
0	1.00E-05	0.86286	0.61088	(Right-Amygdala)(Right-Caudate)(Right-Hippocampus)(Right-Putamen)(Right-Thalamus-Proper)(rh.insula_2)
0	1.00E-05	0.94857	0.75385	(Left-Putamen)(lh.paracentral_5)
0	1.00E-05	0.89714	0.67782	(Right-Caudate)(Right-Hippocampus)(Right-Pallidum)(rh.insula_2)
0	1.00E-05	0.89714	0.67782	(Right-Caudate)(Right-Hippocampus)(Right-Pallidum)(Right-Thalamus-Proper)(rh.insula_2)
0	1.00E-05	0.89714	0.67782	(Right-Caudate)(Right-Hippocampus)(Right-Pallidum)(Right-Putamen)(rh.insula_2)
0	1.00E-05	0.89714	0.67782	(Right-Caudate)(Right-Hippocampus)(Right-Pallidum)(Right-Putamen)(Right-Thalamus-Proper)(rh.insula_2)
0	1.00E-05	0.93714	0.73846	(rh.bankssts_2)(rh.bankssts_3)(rh.superiortemporal_1)
0	1.00E-05	0.90857	0.69874	(Right-Caudate)(Right-Pallidum)(Right-Putamen)(rh.insula_2)
0	1.00E-05	0.90857	0.69874	(Right-Caudate)(Right-Pallidum)(Right-Putamen)(Right-Thalamus-Proper)(rh.insula_2)
0	1.00E-05	0.85714	0.6318	(lh.insula_6)(lh.lateralorbitofrontal_4)(lh.lateralorbitofrontal_6)
0	1.00E-05	0.84571	0.61925	(Left-Hippocampus)(lh.isthmuscingulate_2)(lh.precuneus_10)(lh.precuneus_11)
0	1.00E-05	0.84571	0.61925	(Left-Hippocampus)(lh.isthmuscingulate_2)(lh.isthmuscingulate_3)(lh.precuneus_10)(lh.precuneus_11)
0	1.00E-05	0.85143	0.62762	(Left-Putamen)(lh.insula_6)(lh.lateralorbitofrontal_4)(lh.lateralorbitofrontal_6)
0	1.00E-05	0.96571	0.80335	(Right-Caudate)(rh.insula_1)(rh.insula_2)
0	1.00E-05	0.96571	0.80335	(Right-Caudate)(Right-Putamen)(rh.insula_1)(rh.insula_2)
0	1.00E-05	0.81714	0.59231	(lh.cuneus_3)(lh.lateraloccipital_2)
0	1.00E-05	0.92571	0.73846	(Left-Putamen)(lh.paracentral_5)(lh.precentral_4)
0	1.00E-05	0.83429	0.61506	(lh.insula_6)(lh.lateralorbitofrontal_4)(lh.lateralorbitofrontal_6)(lh.parstriangularis_3)
0	1.00E-05	0.90857	0.71548	(Right-Caudate)(Right-Putamen)(rh.insula_2)(rh.precentral_13)
0	1.00E-05	0.86857	0.66154	(Right-Amygdala)(Right-Caudate)(rh.insula_2)
0	1.00E-05	0.86857	0.66154	(Right-Amygdala)(Right-Caudate)(Right-Thalamus-Proper)(rh.insula_2)
0	1.00E-05	0.87429	0.66946	(Left-Caudate)(Left-Putamen)(Left-Thalamus-Proper)(lh.posteriorcingulate_4)
0	1.00E-05	0.82286	0.60669	(lh.inferiorparietal_3)(lh.inferiorparietal_6)(lh.inferiorparietal_8)
0	1.00E-05	0.97714	0.83264	(Right-Caudate)(rh.insula_2)
0	1.00E-05	0.97714	0.83264	(Right-Caudate)(Right-Thalamus-Proper)(rh.insula_2)
0	1.00E-05	0.97714	0.83264	(Right-Caudate)(Right-Putamen)(rh.insula_2)
0	1.00E-05	0.97714	0.83264	(Right-Caudate)(Right-Putamen)(Right-Thalamus-Proper)(rh.insula_2)
0	1.00E-05	0.87429	0.67364	(Left-Putamen)(Left-Thalamus-Proper)(lh.posteriorcingulate_4)
0	1.00E-05	0.96571	0.81172	(Right-Caudate)(Right-Hippocampus)(rh.insula_2)
0	1.00E-05	0.96571	0.81172	(Right-Caudate)(Right-Hippocampus)(Right-Putamen)(rh.insula_2)
0	1.00E-05	0.96571	0.81172	(Right-Caudate)(Right-Hippocampus)(Right-Thalamus-Proper)(rh.insula_2)
0	1.00E-05	0.96571	0.81172	(Right-Caudate)(Right-Hippocampus)(Right-Putamen)(Right-Thalamus-Proper)(rh.insula_2)

0	1.00E-05	0.84571	0.63846	(lh.fusiform_4)(lh.lingual_8)
0	1.00E-05	0.84	0.63598	(lh.lateraloccipital_2)(lh.lateraloccipital_6)(lh.superiorparietal_12)
1.00E-05	1.00E-05	0.95429	0.79916	(Right-Caudate)(Right-Thalamus-Proper)(rh.insula_1)(rh.insula_2)
1.00E-05	1.00E-05	0.95429	0.79916	(Right-Caudate)(Right-Putamen)(Right-Thalamus-Proper)(rh.insula_1)(rh.insula_2)
1.00E-05	1.00E-05	0.90857	0.73077	(Right-Caudate)(Right-Pallidum)(rh.insula_2)
1.00E-05	1.00E-05	0.90857	0.73077	(Right-Caudate)(Right-Pallidum)(Right-Thalamus-Proper)(rh.insula_2)
1.00E-05	1.00E-05	0.81714	0.61088	(rh.precuneus_5)(rh.superiorparietal_10)(rh.superiorparietal_9)
1.00E-05	1.00E-05	0.89714	0.71548	(lh.fusiform_2)(lh.lateraloccipital_6)(lh.lateraloccipital_7)(lh.lingual_1)(lh.pericalcarine_3)
1.00E-05	1.00E-05	0.87429	0.68462	(Left-Putamen)(lh.paracentral_5)(lh.superiorfrontal_14)
1.00E-05	1.00E-05	0.88571	0.70293	(Right-Caudate)(Right-Putamen)(Right-Thalamus-Proper)(rh.insula_2)(rh.precentral_13)
1.00E-05	1.00E-05	0.81714	0.61506	(lh.fusiform_5)(lh.fusiform_7)(lh.inferiortemporal_6)
1.00E-05	1.00E-05	0.90857	0.73846	(lh.precentral_11)(lh.precentral_16)
1.00E-05	1.00E-05	0.90857	0.73846	(Right-Caudate)(rh.insula_2)(rh.precentral_13)
1.00E-05	1.00E-05	0.81714	0.61925	(lh.bankssts_3)(lh.inferiortemporal_7)(lh.inferiortemporal_8)
1.00E-05	1.00E-05	0.85714	0.66946	(lh.rostralmiddlefrontal_12)(lh.rostralmiddlefrontal_9)(lh.superiorfrontal_2)(lh.superiorfrontal_3)
1.00E-05	1.00E-05	0.85714	0.66946	(rh.bankssts_2)(rh.bankssts_3)(rh.superiortemporal_1)(rh.superiortemporal_3)
1.00E-05	1.00E-05	0.89714	0.72308	(lh.caudalmiddlefrontal_3)(lh.caudalmiddlefrontal_4)(lh.insula_6)
1.00E-05	1.00E-05	0.88	0.7	(lh.lateralorbitofrontal_6)(lh.rostralmiddlefrontal_11)(lh.rostralmiddlefrontal_9)
2.00E-05	1.00E-05	0.90857	0.74059	(Left-Putamen)(lh.caudalmiddlefrontal_4)(lh.insula_6)
2.00E-05	1.00E-05	0.86857	0.68619	(rh.paracentral_6)(rh.precuneus_10)(rh.superiorparietal_7)
2.00E-05	1.00E-05	0.93714	0.78462	(Left-Hippocampus)(lh.fusiform_4)
2.00E-05	1.00E-05	0.89143	0.71967	(Left-Putamen)(lh.caudalmiddlefrontal_3)(lh.caudalmiddlefrontal_4)(lh.insula_6)
2.00E-05	1.00E-05	0.93143	0.77824	(lh.lateraloccipital_7)(lh.precuneus_11)
3.00E-05	1.00E-05	0.87429	0.69874	(lh.rostralmiddlefrontal_12)(lh.superiorfrontal_1)(lh.superiorfrontal_2)(lh.superiorfrontal_3)
3.00E-05	1.00E-05	0.90857	0.74615	(lh.superiortemporal_2)(lh.supramarginal_6)
3.00E-05	1.00E-05	0.90857	0.74615	(lh.caudalmiddlefrontal_4)(lh.insula_6)
3.00E-05	1.00E-05	0.85714	0.67692	(lh.lateralorbitofrontal_6)(lh.parstriangularis_2)(lh.rostralmiddlefrontal_12)
3.00E-05	1.00E-05	0.89714	0.73077	(Left-Pallidum)(lh.posteriorcingulate_3)(lh.precentral_4)
3.00E-05	1.00E-05	0.85714	0.67782	(lh.lateralorbitofrontal_6)(lh.parstriangularis_2)(lh.parstriangularis_3)(lh.rostralmiddlefrontal_12)
3.00E-05	1.00E-05	0.88571	0.71548	(Left-Putamen)(lh.paracentral_5)(lh.postcentral_3)
3.00E-05	1.00E-05	0.88571	0.71548	(Left-Hippocampus)(lh.precuneus_10)(lh.precuneus_11)
3.00E-05	1.00E-05	0.88571	0.71548	(Left-Hippocampus)(lh.isthmuscingulate_3)(lh.precuneus_10)(lh.precuneus_11)
3.00E-05	1.00E-05	0.89143	0.72385	(Left-Putamen)(lh.insula_6)(lh.lateralorbitofrontal_4)
3.00E-05	1.00E-05	0.84	0.6569	(rh.paracentral_6)(rh.superiorparietal_2)(rh.superiorparietal_7)
3.00E-05	1.00E-05	0.94286	0.79916	(rh.rostralmiddlefrontal_11)(rh.rostralmiddlefrontal_12)(rh.rostralmiddlefrontal_8)
4.00E-05	1.00E-05	0.87429	0.70293	(lh.rostralmiddlefrontal_12)(lh.superiorfrontal_1)(lh.superiorfrontal_2)
4.00E-05	1.00E-05	0.85143	0.67364	(lh.parstriangularis_2)(lh.rostralmiddlefrontal_12)(lh.rostralmiddlefrontal_9)
4.00E-05	1.00E-05	0.85143	0.67364	(lh.parstriangularis_2)(lh.parstriangularis_3)(lh.rostralmiddlefrontal_12)(lh.rostralmiddlefrontal_9)
4.00E-05	1.00E-05	0.88	0.7113	(rh.rostralmiddlefrontal_11)(rh.rostralmiddlefrontal_8)(rh.superiorfrontal_1)(rh.superiorfrontal_2)
4.00E-05	1.00E-05	0.81714	0.6318	(Right-Caudate)(rh.lateralorbitofrontal_4)(rh.lateralorbitofrontal_5)(rh.lateralorbitofrontal_7)(rh.medialorbitofrontal_2)
4.00E-05	1.00E-05	0.85714	0.68201	(Left-Putamen)(lh.paracentral_5)(lh.posteriorcingulate_3)
4.00E-05	1.00E-05	0.88571	0.71967	(Left-Caudate)(lh.caudalmiddlefrontal_4)(lh.insula_6)
4.00E-05	1.00E-05	0.88571	0.71967	(Left-Caudate)(Left-Putamen)(lh.caudalmiddlefrontal_4)(lh.insula_6)
5.00E-05	1.00E-05	0.86286	0.69038	(lh.insula_6)(lh.lateralorbitofrontal_4)(lh.parstriangularis_3)
5.00E-05	1.00E-05	0.84571	0.66923	(rh.inferiorparietal_1)(rh.superiorparietal_1)(rh.superiorparietal_10)(rh.superiorparietal_7)
5.00E-05	1.00E-05	0.90286	0.74477	(lh.rostralmiddlefrontal_12)(lh.superiorfrontal_2)(lh.superiorfrontal_3)
5.00E-05	1.00E-05	0.92	0.76923	(lh.bankssts_3)(lh.inferiortemporal_8)
5.00E-05	1.00E-05	0.97714	0.86192	(rh.inferiorparietal_12)(rh.inferiorparietal_7)
5.00E-05	1.00E-05	0.86857	0.69874	(Left-Caudate)(lh.caudalmiddlefrontal_3)(lh.caudalmiddlefrontal_4)(lh.insula_6)
5.00E-05	1.00E-05	0.86857	0.69874	(rh.rostralmiddlefrontal_10)(rh.rostralmiddlefrontal_8)(rh.superiorfrontal_1)(rh.superiorfrontal_2)

5.00E-05	1.00E-05	0.86857	0.69874	(Left-Caudate)(Left-Putamen)(lh.caudalmiddlefrontal_3)(lh.caudalmiddlefrontal_4)(lh.insula_6)
5.00E-05	1.00E-05	0.86286	0.69231	(Left-Putamen)(lh.paracentral_5)(lh.superiorfrontal_12)
5.00E-05	1.00E-05	0.86286	0.69231	(rh.inferiortemporal_5)(rh.middletemporal_2)(rh.middletemporal_4)
5.00E-05	1.00E-05	0.86857	0.7	(lh.parstriangularis_2)(lh.rostralmiddlefrontal_12)
6.00E-05	1.00E-05	0.89714	0.73846	(rh.rostralmiddlefrontal_11)(rh.rostralmiddlefrontal_8)(rh.superiorfrontal_1)
6.00E-05	1.00E-05	0.89143	0.73077	(Left-Hippocampus)(lh.isthmuscingulate_2)(lh.precuneus_10)
6.00E-05	1.00E-05	0.89143	0.73077	(Left-Hippocampus)(lh.isthmuscingulate_2)(lh.isthmuscingulate_3)(lh.precuneus_10)
6.00E-05	1.00E-05	0.88571	0.72308	(Right-Caudate)(Right-Thalamus-Proper)(rh.insula_2)(rh.precentral_13)
6.00E-05	1.00E-05	0.83429	0.6569	(Left-Putamen)(lh.insula_1)(lh.insula_2)(lh.insula_3)(lh.precentral_11)
6.00E-05	1.00E-05	0.84	0.66527	(lh.lingual_6)(lh.lingual_8)(lh.pericalcarine_3)(lh.precuneus_11)
7.00E-05	1.00E-05	0.93714	0.79916	(lh.postcentral_8)(lh.postcentral_9)
8.00E-05	1.00E-05	0.92	0.77406	(lh.inferiorparietal_3)(lh.inferiorparietal_8)
8.00E-05	1.00E-05	0.85143	0.68201	(Right-Caudate)(rh.lateralorbitofrontal_4)(rh.lateralorbitofrontal_5)(rh.medialorbitofrontal_2)
8.00E-05	1.00E-05	0.82857	0.65385	(lh.cuneus_1)(lh.lateraloccipital_6)
8.00E-05	1.00E-05	0.83429	0.66109	(rh.inferiorparietal_6)(rh.inferiorparietal_7)(rh.inferiorparietal_8)
8.00E-05	1.00E-05	0.81714	0.64017	(rh.supramarginal_5)(rh.supramarginal_8)(rh.supramarginal_9)
8.00E-05	1.00E-05	0.83429	0.66154	(lh.insula_1)(lh.insula_2)(lh.insula_3)(lh.precentral_11)
8.00E-05	1.00E-05	0.83429	0.66154	(Right-Pallidum)(Right-Thalamus-Proper)(rh.insula_2)(rh.precentral_13)
8.00E-05	1.00E-05	0.83429	0.66154	(Right-Pallidum)(Right-Putamen)(Right-Thalamus-Proper)(rh.insula_2)(rh.precentral_13)
9.00E-05	1.00E-05	0.85714	0.69038	(lh.medialorbitofrontal_1)(lh.superiorfrontal_1)(lh.superiorfrontal_3)
9.00E-05	1.00E-05	0.97143	0.85774	(Left-Accumbens-area)(Left-Caudate)(lh.medialorbitofrontal_5)
9.00E-05	1.00E-05	0.92571	0.78462	(lh.parstriangularis_2)(lh.rostralmiddlefrontal_6)
9.00E-05	1.00E-05	0.89143	0.7364	(Left-Hippocampus)(lh.lingual_6)(lh.lingual_8)(lh.precuneus_11)
9.00E-05	1.00E-05	0.84571	0.67692	(Right-Pallidum)(rh.insula_2)(rh.precentral_13)
9.00E-05	1.00E-05	0.84571	0.67692	(Right-Pallidum)(Right-Putamen)(rh.insula_2)(rh.precentral_13)
9.00E-05	1.00E-05	0.86286	0.69874	(lh.lateralorbitofrontal_6)(lh.parstriangularis_2)(lh.parstriangularis_3)(lh.rostralmiddlefrontal_6)(lh.rostralmiddlefrontal_9)
0.0001	1.00E-05	0.89714	0.74477	(lh.precentral_11)(lh.precentral_14)(lh.precentral_16)
0.0001	1.00E-05	0.92	0.77692	(lh.fusiform_2)(lh.lateraloccipital_6)(lh.lateraloccipital_7)(lh.lingual_1)
0.0001	1.00E-05	0.85714	0.69231	(lh.inferiorparietal_2)(lh.inferiorparietal_3)(lh.inferiorparietal_8)
0.0001	1.00E-05	0.86857	0.70711	(lh.parstriangularis_2)(lh.parstriangularis_3)(lh.rostralmiddlefrontal_12)
0.00011	1.00E-05	0.93714	0.80335	(rh.cuneus_2)(rh.cuneus_3)
0.00011	1.00E-05	0.96571	0.84937	(Right-Caudate)(rh.lateralorbitofrontal_4)(rh.lateralorbitofrontal_5)
0.00011	1.00E-05	0.90286	0.75385	(Right-Accumbens-area)(rh.medialorbitofrontal_5)
0.00011	1.00E-05	0.87429	0.71548	(Right-Accumbens-area)(Right-Caudate)(rh.medialorbitofrontal_5)
0.00011	1.00E-05	0.89714	0.74615	(lh.lateralorbitofrontal_6)(lh.parstriangularis_2)(lh.rostralmiddlefrontal_6)
0.00011	1.00E-05	0.88571	0.73077	(lh.insula_1)(lh.insula_2)(lh.insula_3)
0.00011	1.00E-05	0.88571	0.73077	(rh.lateralorbitofrontal_7)(rh.rostralmiddlefrontal_11)(rh.rostralmiddlefrontal_12)(rh.rostralmiddlefrontal_8)
0.00011	1.00E-05	0.89143	0.73846	(lh.insula_6)(lh.lateralorbitofrontal_4)
0.00011	1.00E-05	0.83429	0.66527	(rh.supramarginal_3)(rh.supramarginal_5)(rh.supramarginal_8)
0.00012	1.00E-05	0.81143	0.63846	(rh.bankssts_3)(rh.superiortemporal_1)(rh.superiortemporal_2)
0.00012	1.00E-05	0.88571	0.73222	(lh.parstriangularis_2)(lh.parstriangularis_3)(lh.rostralmiddlefrontal_6)(lh.rostralmiddlefrontal_9)
0.00013	1.00E-05	0.82286	0.65272	(rh.insula_2)(rh.insula_3)(rh.insula_7)
0.00013	1.00E-05	0.82286	0.65272	(Right-Putamen)(rh.insula_2)(rh.insula_3)(rh.insula_7)
0.00013	1.00E-05	0.98286	0.88285	(rh.inferiorparietal_1)(rh.superiorparietal_1)(rh.superiorparietal_7)
0.00013	1.00E-05	0.95429	0.83264	(rh.inferiorparietal_12)(rh.inferiorparietal_7)(rh.inferiorparietal_8)
0.00014	1.00E-05	0.86857	0.7113	(lh.cuneus_2)(lh.pericalcarine_1)(lh.pericalcarine_3)
0.00015	1.00E-05	0.94286	0.8159	(Right-Putamen)(rh.precentral_12)
0.00016	1.00E-05	0.92571	0.79079	(Left-Hippocampus)(lh.fusiform_4)(lh.fusiform_5)
0.00016	1.00E-05	0.81714	0.64854	(lh.medialorbitofrontal_1)(lh.rostralmiddlefrontal_12)(lh.superiorfrontal_1)(lh.superiorfrontal_3)
0.00017	1.00E-05	0.88	0.72803	(lh.lateraloccipital_7)(lh.pericalcarine_3)(lh.precuneus_11)

0.00017	1.00E-05	0.97714	0.87448	(lh.lateraloccipital_2)(lh.superiorparietal_12)
0.00017	1.00E-05	0.84571	0.68462	(rh.precentral_1)(rh.precentral_5)
0.00018	1.00E-05	0.84	0.67782	(Left-Caudate)(Left-Putamen)(lh.paracentral_5)(lh.posteriorcingulate_3)
0.00018	1.00E-05	0.84	0.67782	(Left-Putamen)(lh.insula_6)(lh.lateralorbitofrontal_4)(lh.parstriangularis_3)
0.00018	1.00E-05	0.82286	0.6569	(Left-Putamen)(lh.insula_6)(lh.lateralorbitofrontal_4)(lh.rostralmiddlefrontal_9)
0.00018	1.00E-05	0.85143	0.69231	(rh.fusiform_1)(rh.pericalcarine_1)(rh.pericalcarine_3)
0.00018	1.00E-05	0.92571	0.79231	(rh.lateraloccipital_3)(rh.precuneus_1)(rh.superiorparietal_11)
0.00019	1.00E-05	0.86286	0.70711	(lh.lingual_8)(lh.pericalcarine_3)(lh.precuneus_11)
0.00019	1.00E-05	0.86286	0.70711	(Right-Hippocampus)(rh.isthmuscingulate_2)(rh.precuneus_2)(rh.precuneus_3)
0.00019	1.00E-05	0.86286	0.70711	(rh.lateralorbitofrontal_4)(rh.lateralorbitofrontal_5)(rh.lateralorbitofrontal_7)(rh.medialorbitofrontal_2)
0.0002	1.00E-05	0.86857	0.71538	(lh.fusiform_5)(lh.inferiortemporal_6)
0.0002	1.00E-05	0.86857	0.71538	(lh.inferiorparietal_6)(lh.superiortemporal_2)
0.00021	1.00E-05	0.90857	0.76923	(lh.lateraloccipital_6)(lh.lateraloccipital_7)(lh.lingual_1)(lh.pericalcarine_3)
0.00021	1.00E-05	0.87429	0.72308	(rh.superiorparietal_10)(rh.superiorparietal_9)
0.00021	1.00E-05	0.87429	0.72308	(Left-Putamen)(lh.postcentral_3)(lh.precentral_4)
0.00021	1.00E-05	0.88	0.73077	(rh.rostralmiddlefrontal_10)(rh.rostralmiddlefrontal_8)(rh.superiorfrontal_1)
0.00021	1.00E-05	0.88	0.73077	(rh.isthmuscingulate_2)(rh.precuneus_2)(rh.precuneus_3)
0.00021	1.00E-05	0.88	0.73077	(Left-Putamen)(lh.postcentral_3)(lh.superiorparietal_3)
0.00021	1.00E-05	0.89714	0.75385	(rh.precuneus_1)(rh.superiorparietal_11)(rh.superiorparietal_13)
0.00022	1.00E-05	0.90857	0.76987	(Left-Caudate)(Left-Putamen)(lh.paracentral_5)(lh.precentral_4)
0.00022	1.00E-05	0.91429	0.77824	(Left-Putamen)(Left-Thalamus-Proper)(lh.paracentral_5)
0.00023	1.00E-05	0.83429	0.67364	(lh.rostralmiddlefrontal_12)(lh.superiorfrontal_2)(lh.superiorfrontal_3)(lh.superiorfrontal_4)
0.00023	1.00E-05	0.92	0.78661	(rh.precuneus_1)(rh.superiorparietal_13)
0.00023	1.00E-05	0.93143	0.80335	(rh.middletemporal_2)(rh.middletemporal_6)
0.00023	1.00E-05	0.93143	0.80335	(Left-Caudate)(Left-Putamen)(lh.paracentral_5)
0.00024	1.00E-05	0.85714	0.70293	(lh.fusiform_2)(lh.lateraloccipital_6)(lh.lingual_1)(lh.pericalcarine_2)(lh.pericalcarine_3)
0.00025	1.00E-05	0.84	0.68201	(rh.lateralorbitofrontal_2)(rh.lateralorbitofrontal_6)(rh.parsorbitalis_1)(rh.parstriangularis_1)
0.00025	1.00E-05	0.82286	0.66109	(lh.insula_6)(lh.lateralorbitofrontal_4)(lh.rostralmiddlefrontal_9)
0.00028	1.00E-05	0.82857	0.66946	(Left-Caudate)(lh.lateralorbitofrontal_2)(lh.lateralorbitofrontal_7)(lh.medialorbitofrontal_2)(lh.rostralanteriorcingulate_1)
0.00028	1.00E-05	0.89714	0.75732	(Left-Caudate)(Left-Pallidum)(lh.posteriorcingulate_3)(lh.precentral_4)
0.00028	1.00E-05	0.86857	0.71967	(rh.rostralmiddlefrontal_10)(rh.rostralmiddlefrontal_11)(rh.superiorfrontal_1)(rh.superiorfrontal_2)
0.00029	1.00E-05	0.95429	0.841	(Left-Accumbens-area)(Left-Caudate)(Left-Putamen)(lh.medialorbitofrontal_5)
0.00029	1.00E-05	0.83429	0.67692	(rh.fusiform_7)(rh.inferiortemporal_5)(rh.middletemporal_4)
0.00029	1.00E-05	0.83429	0.67692	(lh.lateralorbitofrontal_7)(lh.rostralmiddlefrontal_11)(lh.rostralmiddlefrontal_9)
0.0003	1.00E-05	0.90286	0.76569	(lh.rostralmiddlefrontal_9)(lh.superiorfrontal_1)(lh.superiorfrontal_2)
0.0003	1.00E-05	0.90286	0.76569	(Left-Hippocampus)(lh.lingual_8)(lh.precuneus_11)
0.00031	1.00E-05	0.94857	0.83264	(lh.fusiform_1)(lh.lingual_6)
0.00031	1.00E-05	0.83429	0.67782	(Left-Caudate)(lh.insula_6)(lh.lateralorbitofrontal_4)
0.00031	1.00E-05	0.83429	0.67782	(lh.rostralmiddlefrontal_12)(lh.superiorfrontal_2)(lh.superiorfrontal_4)
0.00031	1.00E-05	0.83429	0.67782	(Left-Caudate)(Left-Putamen)(lh.insula_6)(lh.lateralorbitofrontal_4)
0.00031	1.00E-05	0.83429	0.67782	(Right-Caudate)(Right-Putamen)(rh.lateralorbitofrontal_4)(rh.lateralorbitofrontal_5)(rh.medialorbitofrontal_2)
0.00032	1.00E-05	0.91429	0.78243	(rh.medialorbitofrontal_1)(rh.superiorfrontal_1)
0.00032	1.00E-05	0.91429	0.78243	(Right-Putamen)(rh.precentral_12)(rh.precentral_13)
0.00032	1.00E-05	0.94286	0.82427	(rh.lateralorbitofrontal_7)(rh.rostralmiddlefrontal_11)(rh.rostralmiddlefrontal_12)
0.00032	1.00E-05	0.94286	0.82427	(lh.fusiform_1)(lh.lingual_6)(lh.lingual_8)
0.00032	1.00E-05	0.84571	0.69231	(Left-Putamen)(lh.superiorparietal_2)
0.00033	1.00E-05	0.92571	0.79916	(lh.parstriangularis_2)(lh.parstriangularis_3)(lh.rostralmiddlefrontal_6)
0.00034	1.00E-05	0.85714	0.70711	(Left-Caudate)(Left-Pallidum)(lh.posteriorcingulate_1)
0.00034	1.00E-05	0.85143	0.7	(lh.fusiform_1)(lh.fusiform_4)(lh.inferiortemporal_8)
0.00035	1.00E-05	0.84	0.68619	(rh.lateralorbitofrontal_7)(rh.rostralmiddlefrontal_11)(rh.rostralmiddlefrontal_12)(rh.rostralmiddlefrontal_8)(rh.superiorfrontal_2)

0.00035	1.00E-05	0.85714	0.70769	(lh.postcentral_8)(lh.precentral_14)(lh.precentral_16)
0.00036	1.00E-05	0.88571	0.74477	(Left-Putamen)(lh.insula_1)(lh.insula_2)(lh.insula_3)
0.00037	1.00E-05	0.86286	0.71548	(lh.parstriangularis_2)(lh.parstriangularis_3)(lh.rostralmiddlefrontal_1)(lh.rostralmiddlefrontal_6)
0.00037	1.00E-05	0.92	0.79231	(Left-Putamen)(lh.caudalanteriorcingulate_2)(lh.superiorfrontal_12)(lh.superiorfrontal_14)
0.00037	1.00E-05	0.92	0.79231	(rh.rostralmiddlefrontal_11)(rh.rostralmiddlefrontal_12)(rh.rostralmiddlefrontal_8)(rh.superiorfrontal_2)
0.00037	1.00E-05	0.92	0.79231	(Right-Caudate)(rh.lateralorbitofrontal_4)(rh.lateralorbitofrontal_5)(rh.lateralorbitofrontal_7)
0.00037	1.00E-05	0.86857	0.72308	(lh.rostralmiddlefrontal_12)(lh.rostralmiddlefrontal_9)(lh.superiorfrontal_2)
0.00038	1.00E-05	0.87429	0.73077	(lh.inferiorparietal_5)(lh.superiortemporal_3)
0.00038	1.00E-05	0.87429	0.73077	(lh.precuneus_10)(lh.precuneus_6)(lh.precuneus_7)
0.00039	1.00E-05	0.84571	0.69456	(rh.precuneus_10)(rh.precuneus_8)(rh.superiorparietal_7)
0.00039	1.00E-05	0.84571	0.69456	(Right-Putamen)(rh.lateralorbitofrontal_4)(rh.lateralorbitofrontal_5)(rh.lateralorbitofrontal_7)(rh.medialorbitofrontal_2)
0.00039	1.00E-05	0.88	0.73846	(rh.middletemporal_3)(rh.middletemporal_6)
0.00039	1.00E-05	0.88	0.73846	(rh.inferiorparietal_11)(rh.inferiorparietal_8)
0.00039	1.00E-05	0.88	0.73846	(rh.precuneus_4)(rh.precuneus_6)(rh.precuneus_7)
0.00039	1.00E-05	0.96	0.85356	(Right-Amygdala)(Right-Caudate)
0.00039	1.00E-05	0.96	0.85356	(rh.paracentral_6)(rh.precuneus_10)
0.00039	1.00E-05	0.96	0.85356	(Right-Amygdala)(Right-Caudate)(Right-Thalamus-Proper)
0.00039	1.00E-05	0.96	0.85356	(Right-Amygdala)(Right-Caudate)(Right-Putamen)
0.00039	1.00E-05	0.96	0.85356	(Right-Amygdala)(Right-Caudate)(Right-Pallidum)
0.00039	1.00E-05	0.96	0.85356	(Right-Amygdala)(Right-Caudate)(Right-Pallidum)(Right-Putamen)
0.00039	1.00E-05	0.96	0.85356	(Right-Amygdala)(Right-Caudate)(Right-Pallidum)(Right-Thalamus-Proper)
0.00039	1.00E-05	0.96	0.85356	(Right-Amygdala)(Right-Caudate)(Right-Putamen)(Right-Thalamus-Proper)
0.00039	1.00E-05	0.96	0.85356	(Right-Amygdala)(Right-Caudate)(Right-Pallidum)(Right-Putamen)(Right-Thalamus-Proper)
0.0004	1.00E-05	0.88571	0.74615	(lh.rostralmiddlefrontal_11)(lh.rostralmiddlefrontal_9)
0.0004	1.00E-05	0.86857	0.72385	(Right-Hippocampus)(rh.precuneus_2)(rh.precuneus_3)
0.0004	1.00E-05	0.90286	0.76923	(lh.fusiform_2)(lh.lateraloccipital_7)(lh.lingual_1)(lh.pericalcarine_3)
0.0004	1.00E-05	0.89714	0.76151	(lh.lateralorbitofrontal_6)(lh.parstriangularis_2)(lh.parstriangularis_3)(lh.rostralmiddlefrontal_6)
0.0004	1.00E-05	0.89714	0.76154	(rh.paracentral_6)(rh.superiorparietal_7)
0.0004	1.00E-05	0.89714	0.76154	(rh.cuneus_2)(rh.cuneus_3)(rh.pericalcarine_2)
0.00043	1.00E-05	0.85143	0.70293	(Right-Caudate)(Right-Hippocampus)(Right-Putamen)(Right-Thalamus-Proper)(rh.insula_1)(rh.insula_2)
0.00043	1.00E-05	0.95429	0.84519	(Right-Caudate)(Right-Putamen)(rh.lateralorbitofrontal_4)(rh.lateralorbitofrontal_5)
0.00044	1.00E-05	0.90857	0.77824	(lh.medialorbitofrontal_1)(lh.rostralmiddlefrontal_12)(lh.superiorfrontal_1)
0.00044	1.00E-05	0.81714	0.66154	(lh.superiorparietal_2)(lh.superiorparietal_7)
0.00044	1.00E-05	0.81714	0.66154	(rh.parsopercularis_1)(rh.parstriangularis_4)
0.00045	1.00E-05	0.94857	0.83682	(rh.rostralmiddlefrontal_11)(rh.rostralmiddlefrontal_12)(rh.superiorfrontal_2)
0.00046	1.00E-05	0.91429	0.78661	(Left-Putamen)(lh.precentral_5)
0.00047	1.00E-05	0.80571	0.64854	(rh.precuneus_10)(rh.precuneus_8)(rh.superiorparietal_7)(rh.superiorparietal_8)
0.00047	1.00E-05	0.85714	0.7113	(rh.inferiorparietal_10)(rh.inferiorparietal_11)(rh.inferiorparietal_8)
0.00047	1.00E-05	0.82286	0.66923	(rh.supramarginal_5)(rh.supramarginal_9)
0.00048	1.00E-05	0.82286	0.66946	(lh.fusiform_1)(lh.fusiform_2)(lh.lingual_6)(lh.pericalcarine_3)
0.00048	1.00E-05	0.82286	0.66946	(lh.medialorbitofrontal_1)(lh.medialorbitofrontal_2)(lh.superiorfrontal_1)(lh.superiorfrontal_3)
0.0005	1.00E-05	0.82857	0.67692	(rh.bankssts_1)(rh.inferiortemporal_7)(rh.middletemporal_1)(rh.middletemporal_2)
0.00051	1.00E-05	0.86286	0.71967	(lh.lateralorbitofrontal_6)(lh.parstriangularis_2)(lh.rostralmiddlefrontal_6)(lh.rostralmiddlefrontal_9)
0.00053	1.00E-05	0.84571	0.69874	(rh.bankssts_2)(rh.inferiorparietal_4)(rh.inferiorparietal_9)
0.00053	1.00E-05	0.84571	0.69874	(lh.medialorbitofrontal_1)(lh.rostralmiddlefrontal_12)(lh.superiorfrontal_3)
0.00058	1.00E-05	0.97714	0.88703	(Left-Accumbens-area)(lh.medialorbitofrontal_5)
0.00058	1.00E-05	0.97714	0.88703	(rh.rostralmiddlefrontal_11)(rh.rostralmiddlefrontal_8)
0.00059	1.00E-05	0.85143	0.70711	(Left-Pallidum)(Left-Putamen)(lh.posteriorcingulate_1)
0.00059	1.00E-05	0.85143	0.70711	(Left-Caudate)(Left-Pallidum)(Left-Putamen)(lh.posteriorcingulate_1)
0.00059	1.00E-05	0.85143	0.70711	(Left-Caudate)(lh.lateralorbitofrontal_2)(lh.lateralorbitofrontal_7)(lh.rostralanteriorcingulate_1)

0.00059	1.00E-05	0.85143	0.70711	(Right-Caudate)(Right-Hippocampus)(Right-Putamen)(rh.insula_1)(rh.insula_2)
0.00059	1.00E-05	0.90286	0.77406	(lh.supramarginal_5)(lh.supramarginal_6)
0.00061	1.00E-05	0.85143	0.70769	(lh.supramarginal_3)(lh.supramarginal_4)
0.00061	1.00E-05	0.85143	0.70769	(lh.supramarginal_5)(lh.supramarginal_6)(lh.supramarginal_9)
0.00061	1.00E-05	0.85143	0.70769	(lh.fusiform_1)(lh.fusiform_3)(lh.fusiform_4)
0.00062	1.00E-05	0.95429	0.84937	(lh.medialorbitofrontal_1)(lh.superiorfrontal_1)
0.00064	1.00E-05	0.80571	0.65272	(lh.fusiform_1)(lh.fusiform_2)(lh.lingual_6)(lh.lingual_8)(lh.pericalcarine_3)
0.00064	1.00E-05	0.85714	0.71538	(lh.medialorbitofrontal_2)(lh.rostralmiddlefrontal_11)(lh.rostralmiddlefrontal_9)
0.00064	1.00E-05	0.85714	0.71538	(lh.rostralmiddlefrontal_1)(lh.rostralmiddlefrontal_4)(lh.rostralmiddlefrontal_5)(lh.rostralmiddlefrontal_7)
0.00065	1.00E-05	0.88	0.74477	(Left-Putamen)(Left-Thalamus-Proper)(lh.paracentral_5)(lh.precentral_4)
0.00066	1.00E-05	0.92	0.79916	(Left-Hippocampus)(lh.superiortemporal_3)(lh.superiortemporal_6)(lh.superiortemporal_8)
0.00066	1.00E-05	0.86286	0.72308	(rh.insula_5)(rh.superiortemporal_7)
0.00066	2.00E-05	0.86286	0.72308	(Right-Pallidum)(rh.precentral_11)
0.00068	2.00E-05	0.92571	0.80753	(lh.rostralmiddlefrontal_1)(lh.rostralmiddlefrontal_5)(lh.rostralmiddlefrontal_7)
0.00068	2.00E-05	0.92571	0.80753	(lh.fusiform_2)(lh.lateraloccipital_7)(lh.lingual_1)
0.00068	2.00E-05	0.93714	0.82427	(Left-Pallidum)(lh.posteriorcingulate_3)
0.00068	2.00E-05	0.93714	0.82427	(Left-Caudate)(Left-Pallidum)(lh.posteriorcingulate_3)
0.00068	2.00E-05	0.93714	0.82427	(lh.lateralorbitofrontal_7)(lh.medialorbitofrontal_1)(lh.rostralmiddlefrontal_12)
0.00069	2.00E-05	0.86857	0.73077	(rh.inferiorparietal_12)(rh.inferiorparietal_6)(rh.inferiorparietal_8)
0.00069	2.00E-05	0.80571	0.65385	(lh.lateraloccipital_7)(lh.lingual_1)(lh.lingual_8)
0.00069	2.00E-05	0.80571	0.65385	(rh.bankssts_1)(rh.inferiortemporal_6)(rh.inferiortemporal_7)(rh.middletemporal_2)
0.00071	2.00E-05	0.86286	0.72385	(rh.parstriangularis_4)(rh.rostralmiddlefrontal_3)(rh.rostralmiddlefrontal_6)
0.00071	2.00E-05	0.87429	0.73846	(rh.cuneus_3)(rh.precuneus_1)
0.00071	2.00E-05	0.87429	0.73846	(rh.inferiorparietal_10)(rh.inferiorparietal_11)(rh.lateraloccipital_10)(rh.lateraloccipital_8)
0.00072	2.00E-05	0.88	0.74615	(lh.inferiorparietal_3)(lh.lateraloccipital_6)
0.00072	2.00E-05	0.88	0.74615	(Right-Putamen)(rh.rostralmiddlefrontal_5)(rh.rostralmiddlefrontal_9)
0.00073	2.00E-05	0.91429	0.79231	(lh.cuneus_2)(lh.pericalcarine_3)
0.00073	2.00E-05	0.91429	0.79231	(Left-Pallidum)(Left-Putamen)(lh.posteriorcingulate_3)
0.00073	2.00E-05	0.91429	0.79231	(Left-Caudate)(Left-Pallidum)(Left-Putamen)(lh.posteriorcingulate_3)
0.00073	2.00E-05	0.84571	0.70293	(rh.lateralorbitofrontal_2)(rh.lateralorbitofrontal_6)(rh.parsorbitalis_1)
0.00073	2.00E-05	0.84571	0.70293	(lh.lateralorbitofrontal_2)(lh.lateralorbitofrontal_7)(lh.medialorbitofrontal_2)(lh.rostralanteriorcingulate_1)
0.00073	2.00E-05	0.84571	0.70293	(Left-Caudate)(Left-Putamen)(lh.lateralorbitofrontal_2)(lh.lateralorbitofrontal_7)(lh.rostralanteriorcingulate_1)
0.00074	2.00E-05	0.88571	0.75385	(lh.cuneus_3)(lh.pericalcarine_2)
0.00074	2.00E-05	0.88571	0.75385	(rh.precuneus_2)(rh.precuneus_3)
0.00074	2.00E-05	0.88571	0.75385	(lh.precuneus_2)(lh.precuneus_3)(lh.superiorparietal_2)
0.00074	2.00E-05	0.88571	0.75385	(lh.lateraloccipital_6)(lh.lingual_1)(lh.lingual_3)
0.00074	2.00E-05	0.82857	0.68201	(lh.bankssts_2)(lh.bankssts_3)(lh.inferiortemporal_7)
0.00074	2.00E-05	0.90857	0.78462	(lh.fusiform_1)(lh.fusiform_2)(lh.lingual_6)
0.00077	2.00E-05	0.86857	0.73222	(lh.lateralorbitofrontal_6)(lh.parstriangularis_3)(lh.rostralmiddlefrontal_1)(lh.rostralmiddlefrontal_12)(lh.rostralmiddlefrontal_6)(lh.rostralmiddlefront
0.0008	2.00E-05	0.98857	0.91213	(rh.bankssts_2)(rh.superiortemporal_1)
0.00081	2.00E-05	0.81714	0.66946	(lh.inferiorparietal_3)(lh.lateraloccipital_6)(lh.lateraloccipital_7)
0.00081	2.00E-05	0.85143	0.7113	(rh.lateralorbitofrontal_2)(rh.parsorbitalis_1)(rh.parstriangularis_1)
0.00081	2.00E-05	0.85143	0.7113	(Left-Hippocampus)(lh.inferiorparietal_5)(lh.superiortemporal_3)
0.00083	2.00E-05	0.90286	0.77824	(Left-Caudate)(lh.lateralorbitofrontal_1)
0.00083	2.00E-05	0.90286	0.77824	(Left-Caudate)(Left-Putamen)(lh.lateralorbitofrontal_1)
0.00083	2.00E-05	0.90286	0.77824	(lh.lingual_6)(lh.lingual_8)(lh.precuneus_11)
0.00083	2.00E-05	0.87429	0.74059	(Left-Caudate)(Left-Pallidum)(Left-Putamen)(lh.posteriorcingulate_3)(lh.precentral_4)
0.00085	2.00E-05	0.96	0.86192	(rh.rostralmiddlefrontal_11)(rh.rostralmiddlefrontal_12)
0.00087	2.00E-05	0.80571	0.6569	(rh.precuneus_4)(rh.precuneus_8)(rh.superiorparietal_7)
0.00089	2.00E-05	0.85714	0.71967	(Left-Accumbens-area)(Left-Caudate)(lh.lateralorbitofrontal_2)(lh.medialorbitofrontal_5)

0.00091	2.00E-05	0.91429	0.79498	(lh.lateraloccipital_7)(lh.lingual_1)(lh.pericalcarine_3)
0.00094	2.00E-05	0.92	0.80335	(lh.cuneus_1)(lh.inferiorparietal_1)
0.00094	2.00E-05	0.92	0.80335	(lh.lateralorbitofrontal_7)(lh.medialorbitofrontal_1)(lh.medialorbitofrontal_2)(lh.rostralmiddlefrontal_12)
0.00094	2.00E-05	0.94857	0.84519	(rh.bankssts_3)(rh.superiortemporal_1)
0.00094	2.00E-05	0.94857	0.84519	(rh.inferiortemporal_2)(rh.inferiortemporal_3)
0.00094	2.00E-05	0.94857	0.84519	(rh.rostralmiddlefrontal_10)(rh.superiorfrontal_1)
0.00094	2.00E-05	0.94857	0.84519	(Right-Caudate)(rh.lateralorbitofrontal_5)(rh.medialorbitofrontal_2)
0.00096	2.00E-05	0.92571	0.81172	(Right-Caudate)(rh.lateralorbitofrontal_5)(rh.lateralorbitofrontal_7)(rh.medialorbitofrontal_2)
0.00097	2.00E-05	0.88571	0.75732	(lh.parstriangularis_2)(lh.rostralmiddlefrontal_6)(lh.rostralmiddlefrontal_9)
0.00098	2.00E-05	0.93714	0.82845	(Left-Caudate)(lh.lateralorbitofrontal_2)(lh.lateralorbitofrontal_7)(lh.medialorbitofrontal_2)
0.001	2.00E-05	0.82857	0.68619	(lh.fusiform_1)(lh.fusiform_4)(lh.fusiform_5)(lh.inferiortemporal_8)
0.001	2.00E-05	0.82857	0.68619	(lh.medialorbitofrontal_1)(lh.medialorbitofrontal_2)(lh.rostralmiddlefrontal_12)(lh.superiorfrontal_3)
0.00101	2.00E-05	0.84571	0.70711	(rh.medialorbitofrontal_1)(rh.medialorbitofrontal_2)(rh.rostralmiddlefrontal_11)
0.00101	2.00E-05	0.84571	0.70711	(lh.fusiform_1)(lh.lingual_6)(lh.lingual_8)(lh.pericalcarine_3)
0.00103	2.00E-05	0.89143	0.76569	(lh.rostralmiddlefrontal_9)(lh.superiorfrontal_1)(lh.superiorfrontal_2)(lh.superiorfrontal_3)
0.00105	2.00E-05	0.84571	0.70769	(rh.lateralorbitofrontal_6)(rh.parsorbitalis_1)(rh.parsorbitalis_2)
0.00105	2.00E-05	0.84571	0.70769	(lh.fusiform_4)(lh.fusiform_5)(lh.inferiortemporal_7)
0.00109	2.00E-05	0.81714	0.67364	(Right-Caudate)(rh.insula_2)(rh.precentral_13)(rh.precentral_7)
0.00109	2.00E-05	0.81714	0.67364	(Right-Caudate)(rh.lateralorbitofrontal_7)(rh.medialorbitofrontal_2)(rh.rostralmiddlefrontal_12)(rh.superiorfrontal_2)
0.00109	2.00E-05	0.81714	0.67364	(Right-Caudate)(Right-Putamen)(rh.insula_2)(rh.precentral_13)(rh.precentral_7)
0.0011	2.00E-05	0.89714	0.77406	(Left-Caudate)(Left-Putamen)(Left-Thalamus-Proper)(lh.paracentral_5)
0.0011	2.00E-05	0.89714	0.77406	(rh.medialorbitofrontal_2)(rh.rostralmiddlefrontal_11)(rh.rostralmiddlefrontal_12)(rh.superiorfrontal_2)
0.0011	2.00E-05	0.85143	0.71538	(rh.lingual_4)(rh.pericalcarine_1)(rh.pericalcarine_3)
0.00111	2.00E-05	0.85143	0.71548	(Right-Caudate)(rh.lateralorbitofrontal_7)(rh.rostralmiddlefrontal_12)(rh.superiorfrontal_2)
0.00114	2.00E-05	0.96571	0.87448	(Left-Caudate)(lh.lateralorbitofrontal_2)(lh.lateralorbitofrontal_7)
0.00115	2.00E-05	0.85714	0.72308	(Left-Pallidum)(lh.posteriorcingulate_1)
0.00119	2.00E-05	0.86286	0.73077	(lh.medialorbitofrontal_1)(lh.superiorfrontal_3)
0.00121	2.00E-05	0.98857	0.91632	(lh.isthmuscingulate_2)(lh.isthmuscingulate_3)(lh.precuneus_10)
0.00122	2.00E-05	0.90857	0.79079	(rh.inferiortemporal_5)(rh.middletemporal_4)(rh.middletemporal_6)
0.00124	2.00E-05	0.84	0.70293	(rh.fusiform_2)(rh.fusiform_3)(rh.lingual_5)
0.00124	2.00E-05	0.88	0.75314	(rh.inferiorparietal_11)(rh.inferiorparietal_12)(rh.inferiorparietal_8)
0.00124	2.00E-05	0.88	0.75314	(Left-Putamen)(lh.caudalmiddlefrontal_3)(lh.caudalmiddlefrontal_4)(lh.superiorfrontal_12)
0.00127	2.00E-05	0.87429	0.74615	(rh.inferiorparietal_6)(rh.inferiorparietal_7)
0.00127	2.00E-05	0.87429	0.74615	(rh.precuneus_5)(rh.superiorparietal_10)(rh.superiorparietal_8)
0.00127	2.00E-05	0.87429	0.74615	(lh.lateralorbitofrontal_6)(lh.parstriangularis_3)(lh.rostralmiddlefrontal_1)(lh.rostralmiddlefrontal_12)(lh.rostralmiddlefrontal_6)
0.00128	2.00E-05	0.91429	0.79916	(lh.superiorparietal_12)(lh.superiorparietal_14)
0.00128	2.00E-05	0.91429	0.79916	(rh.parstriangularis_1)(rh.parstriangularis_3)(rh.rostralmiddlefrontal_12)
0.00128	2.00E-05	0.91429	0.79916	(Right-Putamen)(rh.precentral_11)(rh.precentral_12)
0.00128	2.00E-05	0.91429	0.79916	(lh.fusiform_2)(lh.lingual_1)(lh.pericalcarine_2)
0.00131	2.00E-05	0.88	0.75385	(lh.inferiorparietal_3)(lh.inferiorparietal_6)
0.00131	2.00E-05	0.88	0.75385	(rh.inferiorparietal_1)(rh.superiorparietal_10)(rh.superiorparietal_7)
0.00132	2.00E-05	0.92	0.80753	(rh.supramarginal_6)(rh.supramarginal_7)(rh.supramarginal_8)(rh.supramarginal_9)
0.00136	2.00E-05	0.92571	0.8159	(Right-Hippocampus)(Right-Pallidum)(rh.insula_2)
0.00136	2.00E-05	0.92571	0.8159	(lh.lateralorbitofrontal_6)(lh.parstriangularis_2)(lh.parstriangularis_3)(lh.rostralmiddlefrontal_9)
0.00136	2.00E-05	0.92571	0.8159	(Right-Hippocampus)(Right-Pallidum)(Right-Putamen)(rh.insula_2)
0.00136	2.00E-05	0.92571	0.8159	(Right-Hippocampus)(Right-Pallidum)(Right-Thalamus-Proper)(rh.insula_2)
0.00136	2.00E-05	0.92571	0.8159	(Right-Hippocampus)(Right-Pallidum)(Right-Putamen)(Right-Thalamus-Proper)(rh.insula_2)
0.00136	2.00E-05	0.92571	0.8159	(Left-Caudate)(Left-Putamen)(lh.lateralorbitofrontal_2)(lh.lateralorbitofrontal_7)(lh.medialorbitofrontal_2)
0.00136	2.00E-05	0.94857	0.84937	(Right-Amygdala)(Right-Caudate)(Right-Hippocampus)
0.00136	2.00E-05	0.94857	0.84937	(Right-Amygdala)(Right-Caudate)(Right-Hippocampus)(Right-Pallidum)

0.00136	2.00E-05	0.94857	0.84937	(Right-Amygdala)(Right-Caudate)(Right-Hippocampus)(Right-Putamen)
0.00136	2.00E-05	0.94857	0.84937	(Right-Amygdala)(Right-Caudate)(Right-Hippocampus)(Right-Thalamus-Proper)
0.00136	2.00E-05	0.94857	0.84937	(Right-Amygdala)(Right-Caudate)(Right-Hippocampus)(Right-Putamen)(Right-Thalamus-Proper)
0.00136	2.00E-05	0.94857	0.84937	(Right-Amygdala)(Right-Caudate)(Right-Hippocampus)(Right-Pallidum)(Right-Thalamus-Proper)
0.00136	2.00E-05	0.94857	0.84937	(Right-Amygdala)(Right-Caudate)(Right-Hippocampus)(Right-Pallidum)(Right-Putamen)
0.00136	2.00E-05	0.94857	0.84937	(Right-Amygdala)(Right-Caudate)(Right-Hippocampus)(Right-Pallidum)(Right-Putamen)(Right-Thalamus-Proper)
0.00136	2.00E-05	0.89143	0.76923	(lh.precentral_5)(lh.precentral_6)
0.00137	2.00E-05	0.84571	0.7113	(Left-Accumbens-area)(Left-Caudate)(Left-Putamen)(lh.lateralorbitofrontal_2)(lh.medialorbitofrontal_5)
0.00137	2.00E-05	0.89714	0.77692	(rh.precuneus_6)(rh.precuneus_7)
0.00137	2.00E-05	0.89714	0.77692	(Right-Putamen)(rh.paracentral_4)
0.00137	2.00E-05	0.89714	0.77692	(lh.fusiform_1)(lh.fusiform_2)(lh.lingual_6)(lh.lingual_8)
0.00138	2.00E-05	0.90857	0.79231	(lh.lingual_8)(lh.precuneus_11)
0.00138	2.00E-05	0.90857	0.79231	(Right-Caudate)(Right-Putamen)(rh.lateralorbitofrontal_4)(rh.lateralorbitofrontal_5)(rh.lateralorbitofrontal_7)
0.00138	2.00E-05	0.90286	0.78462	(Right-Caudate)(rh.rostralmiddlefrontal_12)(rh.superiorfrontal_2)
0.00138	2.00E-05	0.90286	0.78462	(rh.lateralorbitofrontal_7)(rh.rostralmiddlefrontal_11)(rh.rostralmiddlefrontal_12)(rh.superiorfrontal_2)
0.00138	2.00E-05	0.93143	0.82427	(rh.supramarginal_4)(rh.supramarginal_8)
0.00139	2.00E-05	0.94286	0.841	(rh.rostralmiddlefrontal_10)(rh.superiorfrontal_1)(rh.superiorfrontal_2)
0.00145	2.00E-05	0.86857	0.74059	(Right-Hippocampus)(rh.bankssts_2)(rh.inferiorparietal_4)(rh.superiortemporal_1)
0.00147	2.00E-05	0.82286	0.68462	(rh.bankssts_1)(rh.inferiortemporal_6)(rh.middletemporal_1)(rh.middletemporal_2)
0.00149	2.00E-05	0.97143	0.88703	(Right-Hippocampus)(rh.bankssts_2)(rh.superiortemporal_1)
0.00151	2.00E-05	0.85143	0.71967	(Right-Hippocampus)(rh.bankssts_2)(rh.superiortemporal_1)(rh.superiortemporal_9)
0.00151	2.00E-05	0.83429	0.69874	(Left-Putamen)(lh.precentral_11)(lh.precentral_5)(lh.precentral_7)
0.00156	2.00E-05	0.82857	0.69231	(rh.insula_6)(rh.lateralorbitofrontal_4)
0.00157	2.00E-05	0.87429	0.74895	(Left-Pallidum)(Left-Putamen)(lh.posteriorcingulate_3)(lh.precentral_4)
0.00157	2.00E-05	0.98286	0.90795	(lh.fusiform_2)(lh.lateraloccipital_6)(lh.lingual_1)(lh.pericalcarine_3)
0.00161	2.00E-05	0.90286	0.78661	(Right-Accumbens-area)(Right-Amygdala)(Right-Caudate)(Right-Putamen)
0.00161	2.00E-05	0.90286	0.78661	(Right-Accumbens-area)(Right-Amygdala)(Right-Caudate)(Right-Putamen)(Right-Thalamus-Proper)
0.00161	2.00E-05	0.90286	0.78661	(Right-Accumbens-area)(Right-Amygdala)(Right-Caudate)(Right-Pallidum)(Right-Putamen)
0.00161	2.00E-05	0.90286	0.78661	(Right-Accumbens-area)(Right-Amygdala)(Right-Caudate)(Right-Pallidum)(Right-Putamen)(Right-Thalamus-Proper)
0.00166	2.00E-05	0.96571	0.87866	(Left-Caudate)(Left-Pallidum)(lh.precentral_4)
0.00168	2.00E-05	0.84	0.70711	(Right-Caudate)(rh.lateralorbitofrontal_4)(rh.lateralorbitofrontal_7)(rh.medialorbitofrontal_2)
0.00175	2.00E-05	0.84	0.70769	(rh.inferiortemporal_2)(rh.superiortemporal_10)(rh.superiortemporal_7)
0.00175	2.00E-05	0.84	0.70769	(Left-Putamen)(lh.postcentral_3)(lh.supramarginal_9)
0.00177	2.00E-05	0.81143	0.67364	(rh.parstriangularis_4)(rh.rostralmiddlefrontal_3)(rh.rostralmiddlefrontal_6)(rh.rostralmiddlefrontal_8)
0.00179	2.00E-05	0.96	0.87029	(lh.inferiorparietal_2)(lh.inferiorparietal_3)
0.00179	2.00E-05	0.96	0.87029	(Left-Accumbens-area)(Left-Putamen)(lh.medialorbitofrontal_5)
0.00179	2.00E-05	0.96	0.87029	(Left-Caudate)(Left-Putamen)(lh.lateralorbitofrontal_2)(lh.lateralorbitofrontal_7)
0.00181	2.00E-05	0.86286	0.7364	(rh.fusiform_1)(rh.fusiform_3)(rh.lingual_5)
0.00181	2.00E-05	0.86286	0.7364	(Left-Caudate)(Left-Putamen)(Left-Thalamus-Proper)(lh.paracentral_5)(lh.precentral_4)
0.00183	2.00E-05	0.88571	0.76569	(Right-Putamen)(rh.precentral_11)(rh.precentral_12)(rh.precentral_13)
0.00183	2.00E-05	0.82857	0.69456	(rh.lateralorbitofrontal_4)(rh.lateralorbitofrontal_6)(rh.lateralorbitofrontal_7)(rh.medialorbitofrontal_2)
0.00184	2.00E-05	0.84571	0.71538	(rh.insula_7)(rh.lateralorbitofrontal_2)(rh.rostralmiddlefrontal_8)
0.00184	2.00E-05	0.84571	0.71538	(rh.caudalmiddlefrontal_1)(rh.caudalmiddlefrontal_3)(rh.precentral_10)
0.00184	2.00E-05	0.84571	0.71538	(Right-Caudate)(Right-Pallidum)(Right-Putamen)(rh.precentral_11)
0.00185	2.00E-05	0.92	0.81172	(lh.lateraloccipital_6)(lh.lateraloccipital_7)(lh.lingual_1)
0.00185	2.00E-05	0.92	0.81172	(Right-Caudate)(rh.lateralorbitofrontal_7)(rh.rostralmiddlefrontal_12)
0.00185	2.00E-05	0.92	0.81172	(Right-Caudate)(Right-Putamen)(rh.lateralorbitofrontal_7)(rh.rostralmiddlefrontal_12)
0.00185	2.00E-05	0.84571	0.71548	(Right-Amygdala)(Right-Hippocampus)(Right-Pallidum)(rh.insula_2)
0.00185	2.00E-05	0.84571	0.71548	(Right-Amygdala)(Right-Hippocampus)(Right-Pallidum)(Right-Thalamus-Proper)(rh.insula_2)
0.00185	2.00E-05	0.84571	0.71548	(Right-Amygdala)(Right-Hippocampus)(Right-Pallidum)(Right-Putamen)(rh.insula_2)

0.00185	2.00E-05	0.84571	0.71548	(Right-Amygdala)(Right-Hippocampus)(Right-Pallidum)(Right-Putamen)(Right-Thalamus-Proper)(rh.insula_2)
0.00191	2.00E-05	0.97714	0.89958	(rh.precuneus_5)(rh.superiorparietal_10)
0.00191	2.00E-05	0.92571	0.82008	(lh.lateralorbitofrontal_6)(lh.parstriangularis_2)(lh.rostralmiddlefrontal_9)
0.00193	2.00E-05	0.85143	0.72308	(Right-Amygdala)(Right-Pallidum)(rh.insula_2)
0.00193	2.00E-05	0.85143	0.72308	(lh.lateraloccipital_2)(lh.lateraloccipital_3)(lh.superiorparietal_12)
0.00193	2.00E-05	0.85143	0.72308	(Right-Amygdala)(Right-Pallidum)(Right-Putamen)(rh.insula_2)
0.00193	2.00E-05	0.85143	0.72308	(Right-Amygdala)(Right-Pallidum)(Right-Thalamus-Proper)(rh.insula_2)
0.00193	2.00E-05	0.85143	0.72308	(Right-Amygdala)(Right-Pallidum)(Right-Putamen)(Right-Thalamus-Proper)(rh.insula_2)
0.00194	2.00E-05	0.94857	0.85356	(Right-Caudate)(rh.lateralorbitofrontal_5)(rh.lateralorbitofrontal_7)
0.00194	2.00E-05	0.94857	0.85356	(Right-Pallidum)(Right-Putamen)(rh.posteriorcingulate_2)
0.00194	2.00E-05	0.94857	0.85356	(Right-Caudate)(Right-Pallidum)(Right-Putamen)(rh.posteriorcingulate_2)
0.00198	2.00E-05	0.81714	0.68201	(lh.lateralorbitofrontal_2)(lh.lateralorbitofrontal_7)(lh.medialorbitofrontal_2)(lh.rostralmiddlefrontal_12)
0.00202	2.00E-05	0.85714	0.73077	(rh.precuneus_8)(rh.superiorparietal_10)
0.00202	2.00E-05	0.85714	0.73077	(rh.supramarginal_5)(rh.supramarginal_6)
0.00202	2.00E-05	0.85714	0.73077	(rh.precuneus_10)(rh.superiorparietal_2)(rh.superiorparietal_7)
0.00204	2.00E-05	0.83429	0.70293	(Right-Pallidum)(rh.precentral_10)(rh.precentral_13)
0.00204	2.00E-05	0.83429	0.70293	(rh.lateraloccipital_10)(rh.lateraloccipital_8)(rh.pericalcarine_1)
0.00204	2.00E-05	0.83429	0.70293	(Right-Pallidum)(Right-Putamen)(rh.precentral_10)(rh.precentral_13)
0.0021	2.00E-05	0.86286	0.73846	(Left-Hippocampus)(lh.inferiortemporal_6)
0.0021	2.00E-05	0.86286	0.73846	(lh.lingual_4)(lh.lingual_5)(lh.lingual_8)
0.00218	2.00E-05	0.86857	0.74615	(lh.lateraloccipital_6)(lh.superiorparietal_12)
0.00218	2.00E-05	0.86857	0.74615	(rh.insula_7)(rh.parstriangularis_1)(rh.rostralmiddlefrontal_8)
0.00218	2.00E-05	0.86857	0.74615	(rh.inferiortemporal_2)(rh.inferiortemporal_3)(rh.middletemporal_7)
0.00219	2.00E-05	0.97143	0.89121	(Right-Caudate)(rh.lateralorbitofrontal_5)
0.00221	2.00E-05	0.82286	0.69038	(Right-Putamen)(rh.lateralorbitofrontal_4)(rh.lateralorbitofrontal_6)(rh.lateralorbitofrontal_7)(rh.medialorbitofrontal_2)
0.00222	2.00E-05	0.81143	0.67692	(rh.fusiform_5)(rh.inferiortemporal_6)
0.00223	2.00E-05	0.90286	0.79079	(lh.fusiform_2)(lh.pericalcarine_2)(lh.pericalcarine_3)
0.00224	2.00E-05	0.85714	0.73222	(rh.inferiorparietal_10)(rh.inferiorparietal_4)(rh.inferiorparietal_9)
0.00224	2.00E-05	0.85714	0.73222	(Right-Putamen)(rh.precentral_10)(rh.precentral_11)(rh.precentral_13)
0.00226	2.00E-05	0.84	0.7113	(rh.parstriangularis_4)(rh.rostralmiddlefrontal_6)(rh.rostralmiddlefrontal_8)
0.00226	2.00E-05	0.87429	0.75385	(rh.rostralmiddlefrontal_10)(rh.rostralmiddlefrontal_11)(rh.superiorfrontal_1)
0.00226	2.00E-05	0.87429	0.75385	(lh.rostralmiddlefrontal_4)(lh.rostralmiddlefrontal_5)(lh.rostralmiddlefrontal_7)
0.00226	2.00E-05	0.87429	0.75385	(lh.fusiform_1)(lh.fusiform_5)(lh.lingual_6)
0.00226	2.00E-05	0.87429	0.75385	(Right-Putamen)(rh.lateralorbitofrontal_4)(rh.lateralorbitofrontal_7)(rh.medialorbitofrontal_2)
0.00232	2.00E-05	0.88	0.76151	(rh.rostralmiddlefrontal_10)(rh.rostralmiddlefrontal_11)(rh.rostralmiddlefrontal_8)(rh.superiorfrontal_2)
0.00233	2.00E-05	0.88	0.76154	(rh.lateralorbitofrontal_4)(rh.lateralorbitofrontal_7)(rh.medialorbitofrontal_2)
0.00235	2.00E-05	0.90857	0.79916	(rh.fusiform_1)(rh.lingual_4)(rh.pericalcarine_1)
0.00235	2.00E-05	0.98286	0.91213	(rh.rostralmiddlefrontal_11)(rh.superiorfrontal_1)
0.00235	2.00E-05	0.98286	0.91213	(lh.lateralorbitofrontal_7)(lh.parstriangularis_3)(lh.rostralmiddlefrontal_12)
0.00239	2.00E-05	0.88571	0.76923	(lh.postcentral_14)(lh.postcentral_8)
0.00239	2.00E-05	0.88571	0.76923	(rh.precentral_10)(rh.precentral_11)
0.00239	2.00E-05	0.88571	0.76923	(rh.parstriangularis_4)(rh.rostralmiddlefrontal_6)
0.00239	2.00E-05	0.88571	0.76923	(lh.rostralmiddlefrontal_1)(lh.rostralmiddlefrontal_6)(lh.rostralmiddlefrontal_7)
0.00239	2.00E-05	0.88571	0.76923	(Left-Caudate)(lh.lateralorbitofrontal_2)(lh.lateralorbitofrontal_7)(lh.medialorbitofrontal_5)
0.00242	2.00E-05	0.96571	0.88285	(Left-Hippocampus)(lh.superiortemporal_3)(lh.superiortemporal_8)
0.00244	2.00E-05	0.89143	0.77692	(rh.inferiortemporal_5)(rh.middletemporal_2)(rh.middletemporal_6)
0.00245	2.00E-05	0.86286	0.74059	(lh.lateralorbitofrontal_2)(lh.lateralorbitofrontal_7)(lh.rostralanteriorcingulate_1)
0.00245	2.00E-05	0.86286	0.74059	(lh.parstriangularis_2)(lh.rostralmiddlefrontal_1)(lh.rostralmiddlefrontal_6)
0.00246	2.00E-05	0.82857	0.69874	(Right-Putamen)(rh.insula_2)(rh.insula_7)
0.00246	2.00E-05	0.82857	0.69874	(rh.fusiform_1)(rh.fusiform_3)(rh.lateraloccipital_10)(rh.lingual_5)

0.00247	2.00E-05	0.89714	0.78462	(rh.inferiorparietal_9)(rh.middletemporal_1)
0.00247	2.00E-05	0.89714	0.78462	(rh.bankssts_2)(rh.inferiorparietal_4)(rh.superiortemporal_1)
0.00249	2.00E-05	0.84571	0.71967	(lh.medialorbitofrontal_1)(lh.medialorbitofrontal_2)(lh.superiorfrontal_3)
0.00249	2.00E-05	0.84571	0.71967	(rh.fusiform_1)(rh.lateraloccipital_4)(rh.lateraloccipital_7)(rh.pericalcarine_3)
0.0025	2.00E-05	0.90286	0.79231	(lh.rostralmiddlefrontal_12)(lh.superiorfrontal_2)
0.0025	2.00E-05	0.90286	0.79231	(rh.inferiorparietal_4)(rh.inferiorparietal_9)
0.0025	2.00E-05	0.90286	0.79231	(lh.insula_1)(lh.postcentral_8)(lh.precentral_7)
0.0025	2.00E-05	0.90286	0.79231	(lh.lateraloccipital_7)(lh.lingual_8)(lh.pericalcarine_3)
0.0025	2.00E-05	0.90286	0.79231	(lh.fusiform_1)(lh.inferiortemporal_8)(lh.lateraloccipital_9)
0.0025	2.00E-05	0.90286	0.79231	(Left-Hippocampus)(Left-Thalamus-Proper)(lh.insula_1)(lh.insula_2)
0.0025	2.00E-05	0.90286	0.79231	(Left-Pallidum)(Left-Putamen)(Left-Thalamus-Proper)(lh.posteriorcingulate_3)
0.0025	2.00E-05	0.90286	0.79231	(Right-Pallidum)(Right-Putamen)(rh.posteriorcingulate_2)(rh.precentral_13)
0.0025	2.00E-05	0.90286	0.79231	(Right-Caudate)(Right-Pallidum)(Right-Putamen)(rh.posteriorcingulate_2)(rh.precentral_13)
0.0025	2.00E-05	0.90286	0.79231	(Left-Caudate)(Left-Pallidum)(Left-Putamen)(Left-Thalamus-Proper)(lh.posteriorcingulate_3)
0.0025	2.00E-05	0.88571	0.76987	(Right-Putamen)(rh.lateralorbitofrontal_7)(rh.rostralmiddlefrontal_11)(rh.rostralmiddlefrontal_12)
0.0025	2.00E-05	0.88571	0.76987	(lh.parstriangularis_3)(lh.rostralmiddlefrontal_1)(lh.rostralmiddlefrontal_12)(lh.rostralmiddlefrontal_6)(lh.rostralmiddlefrontal_9)
0.00253	2.00E-05	0.82286	0.69231	(lh.caudalmiddlefrontal_3)(lh.parsopercularis_3)(lh.parstriangularis_3)
0.00253	2.00E-05	0.82286	0.69231	(rh.bankssts_1)(rh.inferiortemporal_6)(rh.inferiortemporal_7)(rh.middletemporal_1)
0.00257	2.00E-05	0.92	0.8159	(rh.rostralmiddlefrontal_8)(rh.superiorfrontal_1)
0.00257	2.00E-05	0.92	0.8159	(rh.paracentral_3)(rh.precentral_14)(rh.precentral_15)
0.00258	2.00E-05	0.96	0.87448	(rh.precuneus_5)(rh.superiorparietal_9)
0.00258	2.00E-05	0.96	0.87448	(rh.lateralorbitofrontal_4)(rh.lateralorbitofrontal_5)(rh.lateralorbitofrontal_7)
0.00263	2.00E-05	1	0.94979	(lh.fusiform_2)(lh.lateraloccipital_6)(lh.lingual_1)
0.00266	2.00E-05	0.92571	0.82427	(lh.fusiform_1)(lh.inferiortemporal_8)
0.00266	2.00E-05	0.92571	0.82427	(Right-Pallidum)(rh.insula_2)
0.00266	2.00E-05	0.92571	0.82427	(rh.caudalmiddlefrontal_1)(rh.caudalmiddlefrontal_2)(rh.caudalmiddlefrontal_3)
0.00266	2.00E-05	0.92571	0.82427	(Right-Pallidum)(Right-Thalamus-Proper)(rh.insula_2)
0.00266	2.00E-05	0.92571	0.82427	(Right-Pallidum)(Right-Putamen)(rh.insula_2)
0.00266	2.00E-05	0.92571	0.82427	(Left-Caudate)(lh.lateralorbitofrontal_7)(lh.medialorbitofrontal_5)
0.00266	3.00E-05	0.92571	0.82427	(Right-Pallidum)(Right-Putamen)(Right-Thalamus-Proper)(rh.insula_2)
0.00267	3.00E-05	0.86857	0.74895	(Left-Putamen)(lh.insula_2)(lh.insula_3)(lh.precentral_11)
0.00267	3.00E-05	0.86857	0.74895	(Left-Caudate)(Left-Pallidum)(Left-Thalamus-Proper)(lh.posteriorcingulate_3)(lh.precentral_4)
0.0027	3.00E-05	0.95429	0.86611	(lh.lateralorbitofrontal_2)(lh.lateralorbitofrontal_7)(lh.medialorbitofrontal_2)
0.00273	3.00E-05	0.83429	0.70711	(Left-Hippocampus)(lh.isthmuscingulate_3)(lh.lingual_8)
0.00273	3.00E-05	0.83429	0.70711	(Right-Caudate)(Right-Putamen)(rh.lateralorbitofrontal_4)(rh.lateralorbitofrontal_7)(rh.medialorbitofrontal_2)
0.00273	3.00E-05	0.93143	0.83264	(lh.precuneus_10)(lh.precuneus_7)
0.00273	3.00E-05	0.93143	0.83264	(rh.caudalmiddlefrontal_1)(rh.caudalmiddlefrontal_2)(rh.caudalmiddlefrontal_4)
0.00274	3.00E-05	0.85143	0.72803	(lh.bankssts_2)(lh.bankssts_3)(lh.inferiortemporal_8)
0.00277	3.00E-05	0.94857	0.85774	(Right-Pallidum)(rh.posteriorcingulate_2)
0.00277	3.00E-05	0.94857	0.85774	(Right-Caudate)(Right-Pallidum)(rh.posteriorcingulate_2)
0.00277	3.00E-05	0.94857	0.85774	(Right-Putamen)(rh.lateralorbitofrontal_4)(rh.lateralorbitofrontal_5)(rh.lateralorbitofrontal_7)
0.00279	3.00E-05	0.94286	0.84937	(lh.inferiorparietal_2)(lh.inferiorparietal_8)
0.00281	3.00E-05	0.80571	0.67364	(Left-Caudate)(lh.parsopercularis_3)(lh.rostralmiddlefrontal_4)
0.00284	3.00E-05	0.83429	0.70769	(Left-Hippocampus)(lh.fusiform_7)(lh.inferiortemporal_6)
0.00287	3.00E-05	0.89714	0.78661	(rh.parsorbitalis_1)(rh.parstriangularis_1)(rh.parstriangularis_3)
0.00287	3.00E-05	0.89714	0.78661	(lh.parstriangularis_3)(lh.rostralmiddlefrontal_12)(lh.rostralmiddlefrontal_4)(lh.rostralmiddlefrontal_9)
0.00291	3.00E-05	0.87429	0.75732	(Right-Putamen)(rh.precentral_10)(rh.precentral_11)
0.003	3.00E-05	0.84	0.71538	(rh.precentral_1)(rh.precentral_7)
0.003	3.00E-05	0.84	0.71538	(lh.inferiorparietal_1)(lh.inferiorparietal_3)(lh.lateraloccipital_6)
0.003	3.00E-05	0.84	0.71538	(rh.inferiortemporal_2)(rh.inferiortemporal_3)(rh.middletemporal_5)

0.00301	3.00E-05	0.85714	0.7364	(Left-Putamen)(lh.lateralorbitofrontal_2)(lh.lateralorbitofrontal_7)(lh.rostralanteriorcingulate_1)
0.00306	3.00E-05	0.90286	0.79498	(lh.precuneus_2)(lh.superiorparietal_7)
0.00306	3.00E-05	0.90286	0.79498	(rh.insula_3)(rh.precentral_1)
0.00316	3.00E-05	0.84571	0.72308	(Left-Putamen)(lh.precentral_11)(lh.precentral_5)
0.0032	3.00E-05	0.97143	0.8954	(rh.supramarginal_6)(rh.supramarginal_7)(rh.supramarginal_8)
0.00323	3.00E-05	0.90857	0.80335	(lh.isthmuscingulate_2)(lh.isthmuscingulate_3)(lh.precuneus_10)(lh.precuneus_6)
0.00328	3.00E-05	0.82857	0.70293	(Right-Putamen)(rh.insula_6)(rh.lateralorbitofrontal_4)
0.00328	3.00E-05	0.82857	0.70293	(rh.lateralorbitofrontal_7)(rh.parstriangularis_1)(rh.rostralmiddlefrontal_12)(rh.rostralmiddlefrontal_13)
0.00332	3.00E-05	0.85143	0.73077	(rh.inferiorparietal_1)(rh.superiorparietal_1)(rh.superiorparietal_10)
0.00332	3.00E-05	0.85143	0.73077	(rh.lateraloccipital_3)(rh.pericalcarine_2)(rh.precuneus_1)
0.00332	3.00E-05	0.85143	0.73077	(rh.lateralorbitofrontal_7)(rh.medialorbitofrontal_2)(rh.rostralmiddlefrontal_11)(rh.rostralmiddlefrontal_12)(rh.superiorfrontal_2)
0.00348	3.00E-05	0.85714	0.73846	(lh.fusiform_1)(lh.fusiform_5)(lh.lingual_6)(lh.lingual_8)
0.0035	3.00E-05	0.96571	0.88703	(Left-Pallidum)(lh.precentral_4)
0.0035	3.00E-05	0.96571	0.88703	(lh.precuneus_6)(lh.precuneus_8)(lh.superiorparietal_11)
0.0035	3.00E-05	0.98286	0.91632	(rh.middletemporal_4)(rh.middletemporal_6)
0.00351	3.00E-05	0.81714	0.69038	(rh.fusiform_7)(rh.inferiortemporal_4)(rh.middletemporal_4)
0.00356	3.00E-05	0.92	0.82008	(Left-Pallidum)(Left-Thalamus-Proper)(lh.posteriorcingulate_3)
0.00356	3.00E-05	0.92	0.82008	(Left-Caudate)(Left-Pallidum)(Left-Thalamus-Proper)(lh.posteriorcingulate_3)
0.00356	3.00E-05	0.92	0.82008	(lh.medialorbitofrontal_1)(lh.medialorbitofrontal_2)(lh.superiorfrontal_1)
0.0036	3.00E-05	0.86857	0.75314	(Left-Pallidum)(Left-Thalamus-Proper)(lh.posteriorcingulate_3)(lh.precentral_4)
0.00364	3.00E-05	0.86286	0.74615	(rh.fusiform_3)(rh.lingual_5)
0.00367	3.00E-05	0.85143	0.73222	(Left-Caudate)(Left-Pallidum)(Left-Putamen)(Left-Thalamus-Proper)(lh.posteriorcingulate_3)(lh.precentral_4)
0.0037	3.00E-05	0.92571	0.82845	(rh.lateraloccipital_3)(rh.precuneus_1)
0.00371	3.00E-05	0.80571	0.67782	(lh.fusiform_1)(lh.fusiform_2)(lh.lateraloccipital_7)(lh.pericalcarine_3)
0.00371	3.00E-05	0.96	0.87866	(Right-Caudate)(Right-Putamen)(rh.lateralorbitofrontal_5)
0.00371	3.00E-05	0.96	0.87866	(Left-Caudate)(Left-Pallidum)(Left-Putamen)(lh.precentral_4)
0.00378	3.00E-05	0.86857	0.75385	(lh.precuneus_6)(lh.precuneus_8)(lh.superiorparietal_11)(lh.superiorparietal_6)
0.00381	3.00E-05	0.93143	0.83682	(lh.precuneus_6)(lh.precuneus_7)
0.00381	3.00E-05	0.93143	0.83682	(rh.inferiorparietal_10)(rh.inferiorparietal_11)(rh.lateraloccipital_10)
0.00381	3.00E-05	0.93143	0.83682	(Right-Caudate)(Right-Putamen)(rh.lateralorbitofrontal_5)(rh.medialorbitofrontal_2)
0.00389	3.00E-05	0.93714	0.84519	(lh.insula_6)(lh.lateralorbitofrontal_6)
0.00389	3.00E-05	0.93714	0.84519	(lh.isthmuscingulate_2)(lh.isthmuscingulate_3)(lh.precuneus_10)(lh.precuneus_11)
0.00391	3.00E-05	0.82286	0.69874	(rh.postcentral_1)(rh.precentral_1)(rh.precentral_7)
0.00391	3.00E-05	0.82286	0.69874	(lh.fusiform_2)(lh.lateraloccipital_6)(lh.lingual_6)(lh.pericalcarine_3)
0.00391	3.00E-05	0.89714	0.79079	(lh.parstriangularis_3)(lh.rostralmiddlefrontal_12)(lh.rostralmiddlefrontal_4)
0.00391	3.00E-05	0.89714	0.79079	(Left-Caudate)(lh.lateralorbitofrontal_7)(lh.medialorbitofrontal_4)(lh.medialorbitofrontal_5)
0.00392	3.00E-05	0.87429	0.76151	(Left-Putamen)(lh.caudalanteriorcingulate_2)(lh.superiorfrontal_11)(lh.superiorfrontal_12)(lh.superiorfrontal_14)
0.00392	3.00E-05	0.87429	0.76151	(Left-Caudate)(Left-Putamen)(lh.lateralorbitofrontal_2)(lh.lateralorbitofrontal_7)(lh.medialorbitofrontal_5)
0.00392	3.00E-05	0.94857	0.86192	(lh.precuneus_6)(lh.precuneus_8)(lh.precuneus_9)(lh.superiorparietal_11)
0.00393	3.00E-05	0.87429	0.76154	(Right-Putamen)(rh.paracentral_4)(rh.precentral_13)
0.00393	3.00E-05	0.87429	0.76154	(lh.fusiform_2)(lh.lateraloccipital_6)(lh.lingual_1)(lh.pericalcarine_2)
0.00393	3.00E-05	0.94286	0.85356	(Right-Caudate)(rh.rostralmiddlefrontal_12)
0.00393	3.00E-05	0.94286	0.85356	(lh.caudalmiddlefrontal_4)(lh.superiorfrontal_12)
0.00393	3.00E-05	0.94286	0.85356	(lh.lateralorbitofrontal_7)(lh.medialorbitofrontal_3)
0.00393	3.00E-05	0.94286	0.85356	(rh.inferiortemporal_5)(rh.middletemporal_4)
0.00393	3.00E-05	0.94286	0.85356	(rh.rostralmiddlefrontal_6)(rh.rostralmiddlefrontal_7)
0.00393	3.00E-05	0.94286	0.85356	(Right-Caudate)(Right-Putamen)(rh.rostralmiddlefrontal_12)
0.00393	3.00E-05	0.94286	0.85356	(lh.isthmuscingulate_2)(lh.precuneus_10)(lh.precuneus_11)
0.00399	3.00E-05	0.81714	0.69231	(rh.precentral_10)(rh.precentral_8)(rh.precentral_9)
0.00399	3.00E-05	0.81714	0.69231	(Right-Pallidum)(rh.insula_1)(rh.insula_2)

0.00403	3.00E-05	0.85714	0.74059	(rh.supramarginal_5)(rh.supramarginal_6)(rh.supramarginal_8)
0.00403	3.00E-05	0.85714	0.74059	(Left-Caudate)(Left-Putamen)(lh.caudalmiddlefrontal_3)(lh.caudalmiddlefrontal_4)(lh.superiorfrontal_12)
0.00406	3.00E-05	0.88	0.76923	(rh.superiorparietal_11)(rh.superiorparietal_7)
0.00406	3.00E-05	0.88	0.76923	(rh.precentral_11)(rh.supramarginal_9)
0.00406	3.00E-05	0.88	0.76923	(lh.cuneus_1)(lh.lateraloccipital_2)(lh.superiorparietal_12)
0.00406	3.00E-05	0.88	0.76923	(lh.caudalmiddlefrontal_3)(lh.parstriangularis_3)(lh.rostralmiddlefrontal_1)(lh.rostralmiddlefrontal_4)
0.00406	3.00E-05	0.88	0.76923	(Right-Putamen)(rh.rostralmiddlefrontal_11)(rh.rostralmiddlefrontal_12)(rh.rostralmiddlefrontal_8)
0.00417	3.00E-05	0.90286	0.79916	(Right-Caudate)(rh.lateralorbitofrontal_6)(rh.rostralmiddlefrontal_12)
0.00417	3.00E-05	0.90286	0.79916	(Right-Accumbens-area)(Right-Amygdala)(Right-Caudate)
0.00417	3.00E-05	0.90286	0.79916	(Right-Accumbens-area)(Right-Amygdala)(Right-Caudate)(Right-Pallidum)
0.00417	3.00E-05	0.90286	0.79916	(Right-Accumbens-area)(Right-Amygdala)(Right-Caudate)(Right-Thalamus-Proper)
0.00417	3.00E-05	0.90286	0.79916	(Right-Caudate)(Right-Putamen)(rh.lateralorbitofrontal_6)(rh.rostralmiddlefrontal_12)
0.00417	3.00E-05	0.90286	0.79916	(Right-Accumbens-area)(Right-Amygdala)(Right-Caudate)(Right-Pallidum)(Right-Thalamus-Proper)
0.00418	3.00E-05	0.88571	0.77692	(rh.fusiform_4)(rh.fusiform_5)
0.00418	3.00E-05	0.88571	0.77692	(lh.caudalmiddlefrontal_3)(lh.parstriangularis_3)(lh.rostralmiddlefrontal_4)
0.00418	3.00E-05	0.88571	0.77692	(rh.fusiform_2)(rh.lateraloccipital_8)(rh.lingual_5)(rh.lingual_7)
0.00418	3.00E-05	0.88571	0.77692	(rh.inferiortemporal_6)(rh.inferiortemporal_7)(rh.middletemporal_1)(rh.middletemporal_2)
0.00425	3.00E-05	0.82286	0.7	(rh.insula_7)(rh.rostralmiddlefrontal_5)(rh.rostralmiddlefrontal_8)
0.00425	3.00E-05	0.82286	0.7	(rh.insula_3)(rh.postcentral_1)(rh.precentral_1)
0.00425	3.00E-05	0.88	0.76987	(lh.lateralorbitofrontal_6)(lh.parsorbitalis_1)(lh.rostralmiddlefrontal_12)
0.00425	3.00E-05	0.88	0.76987	(lh.inferiorparietal_8)(lh.superiorparietal_11)(lh.superiorparietal_5)
0.00425	3.00E-05	0.88	0.76987	(Left-Hippocampus)(lh.superiortemporal_2)(lh.superiortemporal_3)
0.00428	3.00E-05	0.89143	0.78462	(rh.middletemporal_2)(rh.middletemporal_4)
0.00428	3.00E-05	0.89143	0.78462	(rh.bankssts_1)(rh.inferiortemporal_6)(rh.middletemporal_2)
0.00428	3.00E-05	0.89143	0.78462	(lh.cuneus_1)(lh.superiorparietal_12)(lh.superiorparietal_14)
0.00434	3.00E-05	0.82857	0.70711	(lh.lateralorbitofrontal_2)(lh.lateralorbitofrontal_7)(lh.rostralmiddlefrontal_12)
0.00437	4.00E-05	0.89714	0.79231	(rh.rostralmiddlefrontal_10)(rh.rostralmiddlefrontal_11)(rh.rostralmiddlefrontal_8)
0.00437	4.00E-05	0.89714	0.79231	(rh.paracentral_6)(rh.precuneus_10)(rh.superiorparietal_2)
0.00437	4.00E-05	0.89714	0.79231	(rh.bankssts_2)(rh.inferiorparietal_10)(rh.inferiorparietal_9)
0.00437	4.00E-05	0.89714	0.79231	(Left-Putamen)(lh.precentral_5)(lh.precentral_7)
0.00437	4.00E-05	0.89714	0.79231	(lh.fusiform_2)(lh.lingual_1)(lh.pericalcarine_2)(lh.pericalcarine_3)
0.00443	4.00E-05	0.90857	0.80753	(rh.lateralorbitofrontal_7)(rh.rostralmiddlefrontal_11)(rh.rostralmiddlefrontal_8)
0.00443	4.00E-05	0.90857	0.80753	(rh.rostralmiddlefrontal_8)(rh.superiorfrontal_1)(rh.superiorfrontal_2)
0.00443	4.00E-05	0.90857	0.80753	(Right-Caudate)(Right-Putamen)(rh.lateralorbitofrontal_5)(rh.lateralorbitofrontal_7)(rh.medialorbitofrontal_2)
0.00444	4.00E-05	0.84571	0.72803	(Left-Caudate)(Left-Putamen)(lh.lateralorbitofrontal_2)(lh.lateralorbitofrontal_7)(lh.medialorbitofrontal_4)
0.00446	4.00E-05	0.99429	0.94142	(lh.lateraloccipital_6)(lh.lingual_1)(lh.pericalcarine_3)
0.00451	4.00E-05	0.82857	0.70769	(lh.insula_1)(lh.precentral_4)
0.00451	4.00E-05	0.82857	0.70769	(rh.insula_2)(rh.insula_7)
0.00451	4.00E-05	0.82857	0.70769	(rh.lingual_6)(rh.pericalcarine_1)
0.00451	4.00E-05	0.82857	0.70769	(rh.bankssts_1)(rh.inferiortemporal_5)(rh.inferiortemporal_6)(rh.middletemporal_2)
0.00466	4.00E-05	0.97143	0.89958	(lh.lateralorbitofrontal_6)(lh.lateralorbitofrontal_7)(lh.parstriangularis_3)(lh.rostralmiddlefrontal_12)
0.00467	4.00E-05	0.91429	0.8159	(lh.fusiform_2)(lh.pericalcarine_2)
0.00467	4.00E-05	0.91429	0.8159	(lh.lateralorbitofrontal_5)(lh.lateralorbitofrontal_6)
0.00478	4.00E-05	0.83429	0.71538	(rh.lateraloccipital_8)(rh.pericalcarine_1)(rh.pericalcarine_3)
0.00478	4.00E-05	0.83429	0.71538	(rh.fusiform_3)(rh.fusiform_4)(rh.fusiform_5)
0.00478	4.00E-05	0.83429	0.71538	(Left-Hippocampus)(lh.isthmuscingulate_2)(lh.isthmuscingulate_3)(lh.precuneus_6)
0.00478	4.00E-05	0.83429	0.71538	(Right-Putamen)(rh.precentral_10)(rh.precentral_11)(rh.precentral_7)
0.00482	4.00E-05	0.86857	0.75732	(rh.lateralorbitofrontal_3)(rh.lateralorbitofrontal_6)
0.00482	4.00E-05	0.86857	0.75732	(rh.precuneus_8)(rh.superiorparietal_7)(rh.superiorparietal_8)
0.00482	4.00E-05	0.86857	0.75732	(lh.lateralorbitofrontal_6)(lh.rostralmiddlefrontal_1)(lh.rostralmiddlefrontal_12)(lh.rostralmiddlefrontal_6)(lh.rostralmiddlefrontal_9)

0.00488	4.00E-05	0.80571	0.68201	(rh.precentral_11)(rh.superiorparietal_1)(rh.supramarginal_9)
0.00488	4.00E-05	0.80571	0.68201	(lh.lateralorbitofrontal_6)(lh.parsorbitalis_1)(lh.parstriangularis_3)(lh.rostralmiddlefrontal_12)(lh.rostralmiddlefrontal_9)
0.00489	4.00E-05	0.85143	0.7364	(lh.medialorbitofrontal_2)(lh.superiorfrontal_1)(lh.superiorfrontal_2)
0.00489	4.00E-05	0.85143	0.7364	(Left-Caudate)(Left-Putamen)(lh.paracentral_5)(lh.superiorfrontal_14)
0.00489	4.00E-05	0.85143	0.7364	(lh.medialorbitofrontal_2)(lh.superiorfrontal_1)(lh.superiorfrontal_2)(lh.superiorfrontal_3)
0.00489	4.00E-05	0.85143	0.7364	(Left-Pallidum)(Left-Putamen)(Left-Thalamus-Proper)(lh.posteriorcingulate_3)(lh.precentral_4)
0.0049	4.00E-05	0.92	0.82427	(Left-Putamen)(lh.caudalmiddlefrontal_4)(lh.superiorfrontal_12)
0.0049	4.00E-05	0.92	0.82427	(lh.fusiform_2)(lh.lingual_6)(lh.pericalcarine_3)
0.0049	4.00E-05	0.92	0.82427	(lh.insula_6)(lh.lateralorbitofrontal_6)(lh.parstriangularis_3)
0.00494	4.00E-05	0.89143	0.78661	(rh.inferiortemporal_3)(rh.middletemporal_5)
0.00494	4.00E-05	0.89143	0.78661	(Right-Amygdala)(rh.temporalpole_1)
0.00494	4.00E-05	0.89143	0.78661	(lh.rostralmiddlefrontal_1)(lh.rostralmiddlefrontal_4)(lh.rostralmiddlefrontal_7)(lh.rostralmiddlefrontal_9)
0.00505	4.00E-05	0.96571	0.89121	(lh.lateralorbitofrontal_7)(lh.medialorbitofrontal_1)
0.00505	4.00E-05	0.96571	0.89121	(lh.lateralorbitofrontal_6)(lh.rostralmiddlefrontal_11)
0.00505	4.00E-05	0.96571	0.89121	(Left-Caudate)(lh.lateralorbitofrontal_7)(lh.medialorbitofrontal_4)
0.00506	4.00E-05	0.84	0.72308	(Left-Hippocampus)(lh.isthmuscingulate_2)(lh.precuneus_6)
0.00506	4.00E-05	0.84	0.72308	(Left-Caudate)(Left-Putamen)(lh.rostralmiddlefrontal_3)(lh.rostralmiddlefrontal_4)
0.00511	4.00E-05	0.92571	0.83264	(lh.inferiorparietal_1)(lh.precuneus_9)
0.00511	4.00E-05	0.92571	0.83264	(lh.fusiform_1)(lh.fusiform_3)(lh.lateraloccipital_9)
0.00515	4.00E-05	0.82286	0.70293	(Left-Putamen)(lh.lateralorbitofrontal_2)(lh.lateralorbitofrontal_7)(lh.rostralmiddlefrontal_12)
0.00525	4.00E-05	0.87429	0.76569	(Right-Caudate)(Right-Putamen)(rh.lateralorbitofrontal_4)(rh.medialorbitofrontal_2)
0.00529	4.00E-05	0.93143	0.841	(rh.insula_2)(rh.precentral_13)
0.00529	4.00E-05	0.93143	0.841	(Right-Putamen)(rh.insula_2)(rh.precentral_13)
0.00529	4.00E-05	0.93143	0.841	(Left-Putamen)(lh.insula_6)(lh.lateralorbitofrontal_6)
0.00531	4.00E-05	0.96	0.88285	(lh.lateralorbitofrontal_7)(lh.parstriangularis_3)(lh.rostralmiddlefrontal_12)(lh.rostralmiddlefrontal_9)
0.00533	4.00E-05	0.84571	0.73077	(rh.superiortemporal_1)(rh.superiortemporal_2)
0.00533	4.00E-05	0.84571	0.73077	(rh.precentral_7)(rh.supramarginal_9)
0.00533	4.00E-05	0.84571	0.73077	(lh.precuneus_2)(lh.precuneus_3)(lh.superiorparietal_7)
0.00537	4.00E-05	0.85714	0.74477	(lh.lateralorbitofrontal_2)(lh.medialorbitofrontal_2)(lh.rostralanteriorcingulate_1)
0.00537	5.00E-05	0.85714	0.74477	(lh.fusiform_2)(lh.lateraloccipital_11)(lh.lingual_1)(lh.pericalcarine_2)
0.00542	5.00E-05	0.93714	0.84937	(Right-Caudate)(Right-Putamen)(rh.lateralorbitofrontal_5)(rh.lateralorbitofrontal_7)
0.00542	5.00E-05	0.93714	0.84937	(rh.lateralorbitofrontal_6)(rh.parsorbitalis_1)(rh.parstriangularis_1)
0.00542	5.00E-05	0.93714	0.84937	(Left-Putamen)(lh.lateralorbitofrontal_2)(lh.lateralorbitofrontal_7)(lh.medialorbitofrontal_2)
0.00546	5.00E-05	0.95429	0.87448	(rh.rostralmiddlefrontal_12)(rh.rostralmiddlefrontal_7)(rh.rostralmiddlefrontal_8)
0.00546	5.00E-05	0.95429	0.87448	(rh.precuneus_4)(rh.precuneus_5)(rh.superiorparietal_10)
0.00546	5.00E-05	0.95429	0.87448	(Left-Hippocampus)(lh.superiortemporal_3)(lh.superiortemporal_6)
0.00546	5.00E-05	0.95429	0.87448	(Left-Putamen)(lh.insula_1)(lh.precentral_11)(lh.precentral_7)
0.0055	5.00E-05	0.94286	0.85774	(lh.rostralmiddlefrontal_9)(lh.superiorfrontal_2)
0.0055	5.00E-05	0.94286	0.85774	(Right-Putamen)(rh.lateralorbitofrontal_4)(rh.lateralorbitofrontal_6)(rh.lateralorbitofrontal_7)
0.00552	5.00E-05	0.94857	0.86611	(lh.middletemporal_7)(lh.superiortemporal_11)
0.00552	5.00E-05	0.94857	0.86611	(lh.lateralorbitofrontal_7)(lh.medialorbitofrontal_1)(lh.medialorbitofrontal_2)
0.00552	5.00E-05	0.94857	0.86611	(lh.lateralorbitofrontal_6)(lh.lateralorbitofrontal_7)(lh.parstriangularis_3)(lh.rostralmiddlefrontal_12)(lh.rostralmiddlefrontal_9)
0.00561	5.00E-05	0.85143	0.73846	(rh.precuneus_10)(rh.superiorparietal_1)(rh.superiorparietal_6)
0.00561	5.00E-05	0.85143	0.73846	(Right-Caudate)(Right-Hippocampus)(rh.insula_1)(rh.insula_2)
0.00561	5.00E-05	0.85143	0.73846	(Right-Caudate)(Right-Putamen)(rh.lateralorbitofrontal_7)(rh.rostralmiddlefrontal_12)(rh.rostralmiddlefrontal_8)
0.00561	5.00E-05	0.85143	0.73846	(Right-Caudate)(Right-Hippocampus)(Right-Thalamus-Proper)(rh.insula_1)(rh.insula_2)
0.00566	5.00E-05	0.90286	0.80335	(Right-Putamen)(rh.lateralorbitofrontal_2)(rh.lateralorbitofrontal_4)(rh.lateralorbitofrontal_6)(rh.lateralorbitofrontal_7)
0.0057	5.00E-05	0.88	0.77406	(rh.lateraloccipital_1)(rh.superiorparietal_11)(rh.superiorparietal_13)
0.0057	5.00E-05	0.88	0.77406	(Right-Caudate)(rh.lateralorbitofrontal_6)(rh.lateralorbitofrontal_7)(rh.rostralmiddlefrontal_12)
0.0057	5.00E-05	0.88	0.77406	(Right-Caudate)(Right-Putamen)(rh.lateralorbitofrontal_6)(rh.lateralorbitofrontal_7)(rh.rostralmiddlefrontal_12)

0.00572	5.00E-05	0.82857	0.7113	(lh.parstriangularis_1)(lh.parstriangularis_2)(lh.parstriangularis_3)
0.00577	5.00E-05	0.80571	0.68462	(lh.lateralorbitofrontal_4)(lh.parsorbitalis_1)(lh.parstriangularis_1)
0.00577	5.00E-05	0.80571	0.68462	(lh.parsopercularis_3)(lh.parstriangularis_3)(lh.rostralmiddlefrontal_4)
0.00588	5.00E-05	0.86286	0.75314	(rh.medialorbitofrontal_2)(rh.rostralmiddlefrontal_11)(rh.rostralmiddlefrontal_8)
0.00588	5.00E-05	0.86286	0.75314	(Left-Caudate)(Left-Putamen)(lh.caudalanteriorcingulate_2)(lh.superiorfrontal_11)(lh.superiorfrontal_12)(lh.superiorfrontal_14)
0.00589	5.00E-05	0.85714	0.74615	(rh.insula_7)(rh.rostralmiddlefrontal_3)(rh.rostralmiddlefrontal_8)
0.00589	5.00E-05	0.85714	0.74615	(rh.bankssts_2)(rh.superiortemporal_1)(rh.superiortemporal_9)
0.00589	5.00E-05	0.85714	0.74615	(lh.inferiorparietal_8)(lh.inferiorparietal_9)(lh.superiorparietal_5)
0.00602	5.00E-05	0.90857	0.81172	(Left-Caudate)(Left-Putamen)(lh.lateralorbitofrontal_7)(lh.medialorbitofrontal_5)
0.00607	6.00E-05	0.81714	0.69874	(Right-Pallidum)(Right-Putamen)(rh.insula_1)(rh.insula_2)
0.00607	6.00E-05	0.81714	0.69874	(Right-Putamen)(rh.precentral_10)(rh.precentral_12)(rh.precentral_13)
0.00609	6.00E-05	0.97714	0.91213	(Left-Caudate)(lh.caudalanteriorcingulate_2)(lh.caudalmiddlefrontal_3)(lh.superiorfrontal_11)
0.00616	6.00E-05	0.88571	0.78243	(lh.parsorbitalis_1)(lh.rostralmiddlefrontal_12)
0.00616	6.00E-05	0.88571	0.78243	(rh.lateralorbitofrontal_6)(rh.parstriangularis_1)(rh.parstriangularis_3)(rh.rostralmiddlefrontal_12)
0.00616	6.00E-05	0.88571	0.78243	(lh.lateralorbitofrontal_7)(lh.parstriangularis_3)(lh.rostralmiddlefrontal_12)(lh.rostralmiddlefrontal_6)(lh.rostralmiddlefrontal_9)
0.00617	6.00E-05	0.86286	0.75385	(lh.fusiform_1)(lh.lingual_6)(lh.pericalcarine_3)
0.00617	6.00E-05	0.86286	0.75385	(Left-Putamen)(lh.caudalmiddlefrontal_4)(lh.caudalmiddlefrontal_6)(lh.superiorfrontal_12)
0.00621	6.00E-05	0.98857	0.93305	(lh.inferiortemporal_8)(lh.lateraloccipital_9)
0.00621	6.00E-05	0.98857	0.93305	(lh.isthmuscingulate_2)(lh.precuneus_10)
0.00621	6.00E-05	0.98857	0.93305	(rh.isthmuscingulate_2)(rh.precuneus_4)(rh.precuneus_7)
0.00621	6.00E-05	0.98857	0.93305	(Right-Accumbens-area)(Right-Caudate)(Right-Putamen)
0.00621	6.00E-05	0.98857	0.93305	(Right-Accumbens-area)(Right-Caudate)(Right-Pallidum)(Right-Putamen)
0.00634	6.00E-05	0.83429	0.71967	(rh.inferiorparietal_10)(rh.inferiorparietal_9)(rh.middletemporal_1)
0.00637	6.00E-05	0.91429	0.82008	(Left-Putamen)(lh.posteriorcingulate_3)(lh.superiorfrontal_14)
0.00637	6.00E-05	0.91429	0.82008	(lh.fusiform_1)(lh.lingual_8)(lh.pericalcarine_3)
0.00643	6.00E-05	0.86857	0.76154	(lh.middletemporal_4)(lh.superiortemporal_6)
0.00643	6.00E-05	0.86857	0.76154	(lh.parsorbitalis_1)(lh.parsorbitalis_2)
0.00643	6.00E-05	0.86857	0.76154	(rh.precentral_11)(rh.superiorparietal_1)(rh.supramarginal_1)
0.00643	6.00E-05	0.86857	0.76154	(rh.caudalmiddlefrontal_1)(rh.caudalmiddlefrontal_2)(rh.caudalmiddlefrontal_3)(rh.caudalmiddlefrontal_4)
0.00643	6.00E-05	0.86857	0.76154	(Right-Caudate)(rh.medialorbitofrontal_2)(rh.rostralmiddlefrontal_12)(rh.superiorfrontal_2)
0.00647	6.00E-05	0.85143	0.74059	(Left-Accumbens-area)(Left-Putamen)(lh.lateralorbitofrontal_2)(lh.medialorbitofrontal_5)
0.00658	6.00E-05	0.81714	0.7	(lh.parsopercularis_3)(lh.rostralmiddlefrontal_4)
0.00658	6.00E-05	0.81714	0.7	(Left-Pallidum)(lh.insula_1)(lh.precentral_7)
0.00658	7.00E-05	0.81714	0.7	(Right-Putamen)(rh.insula_7)(rh.rostralmiddlefrontal_5)(rh.rostralmiddlefrontal_8)
0.00665	7.00E-05	0.89143	0.79079	(rh.lateralorbitofrontal_4)(rh.medialorbitofrontal_2)
0.00665	7.00E-05	0.89143	0.79079	(lh.lateralorbitofrontal_7)(lh.medialorbitofrontal_1)(lh.medialorbitofrontal_3)
0.00669	7.00E-05	0.87429	0.76923	(rh.posteriorcingulate_4)(rh.precentral_11)
0.00669	7.00E-05	0.87429	0.76923	(rh.parsopercularis_4)(rh.rostralmiddlefrontal_8)
0.00669	7.00E-05	0.87429	0.76923	(rh.medialorbitofrontal_1)(rh.rostralmiddlefrontal_11)(rh.superiorfrontal_1)
0.00669	7.00E-05	0.87429	0.76923	(rh.insula_3)(rh.precentral_1)(rh.precentral_6)
0.00669	7.00E-05	0.87429	0.76923	(lh.bankssts_2)(lh.inferiortemporal_7)(lh.inferiortemporal_8)
0.00669	7.00E-05	0.87429	0.76923	(Right-Caudate)(Right-Putamen)(rh.rostralmiddlefrontal_12)(rh.superiorfrontal_2)
0.00669	7.00E-05	0.87429	0.76923	(Left-Putamen)(lh.caudalanteriorcingulate_2)(lh.caudalmiddlefrontal_3)(lh.superiorfrontal_11)(lh.superiorfrontal_12)
0.00675	7.00E-05	0.82286	0.70711	(lh.lateraloccipital_6)(lh.lingual_1)(lh.lingual_3)(lh.lingual_4)
0.00675	7.00E-05	0.82286	0.70711	(Right-Caudate)(Right-Putamen)(rh.lateralorbitofrontal_7)(rh.rostralmiddlefrontal_12)(rh.superiorfrontal_2)
0.00676	7.00E-05	0.97143	0.90377	(lh.precentral_15)(lh.precentral_16)
0.00676	7.00E-05	0.97143	0.90377	(lh.isthmuscingulate_3)(lh.precuneus_10)(lh.precuneus_6)
0.00679	7.00E-05	0.99429	0.94561	(lh.inferiortemporal_7)(lh.inferiortemporal_8)
0.00679	7.00E-05	0.99429	0.94561	(rh.insula_3)(rh.insula_7)
0.00679	7.00E-05	0.99429	0.94561	(rh.fusiform_2)(rh.lingual_7)

0.00679	7.00E-05	0.99429	0.94561	(rh.precentral_10)(rh.precentral_8)
0.00679	8.00E-05	0.99429	0.94561	(rh.caudalmiddlefrontal_1)(rh.caudalmiddlefrontal_3)
0.00679	8.00E-05	0.99429	0.94561	(Right-Putamen)(rh.insula_3)(rh.insula_7)
0.00694	8.00E-05	0.88	0.77692	(lh.fusiform_8)(lh.insula_4)
0.00694	8.00E-05	0.88	0.77692	(lh.insula_1)(lh.postcentral_3)
0.00694	8.00E-05	0.88	0.77692	(rh.precentral_3)(rh.precentral_6)
0.00699	8.00E-05	0.87429	0.76987	(Right-Caudate)(Right-Putamen)(rh.insula_1)(rh.insula_2)(rh.supramarginal_9)
0.00701	8.00E-05	0.84	0.72803	(lh.lateraloccipital_6)(lh.pericalcarine_3)(lh.superiorparietal_12)
0.00701	8.00E-05	0.84	0.72803	(Left-Caudate)(Left-Putamen)(lh.paracentral_5)(lh.superiorfrontal_12)
0.00701	8.00E-05	0.84	0.72803	(Left-Caudate)(lh.lateralorbitofrontal_2)(lh.medialorbitofrontal_2)(lh.rostralanteriorcingulate_1)
0.00701	8.00E-05	0.82286	0.70769	(rh.parsorbitalis_2)(rh.parstriangularis_3)(rh.rostralmiddlefrontal_12)
0.00701	8.00E-05	0.82286	0.70769	(rh.fusiform_2)(rh.fusiform_3)(rh.fusiform_5)
0.00702	8.00E-05	0.92571	0.83682	(lh.superiortemporal_11)(lh.superiortemporal_9)
0.00702	8.00E-05	0.92571	0.83682	(rh.parsorbitalis_1)(rh.parstriangularis_3)
0.0071	9.00E-05	0.85714	0.74895	(Right-Pallidum)(rh.precentral_11)(rh.precentral_13)
0.0071	9.00E-05	0.85714	0.74895	(Right-Pallidum)(Right-Putamen)(rh.precentral_11)(rh.precentral_13)
0.0071	9.00E-05	0.85714	0.74895	(Left-Putamen)(lh.insula_6)(lh.lateralorbitofrontal_6)(lh.parstriangularis_3)(lh.rostralmiddlefrontal_1)
0.00711	9.00E-05	0.81143	0.69456	(lh.parsorbitalis_1)(lh.parstriangularis_3)(lh.rostralmiddlefrontal_12)(lh.rostralmiddlefrontal_9)
0.00711	9.00E-05	0.81143	0.69456	(rh.fusiform_1)(rh.fusiform_2)(rh.fusiform_3)(rh.lingual_5)
0.00714	9.00E-05	0.89714	0.79916	(rh.parsorbitalis_1)(rh.parsorbitalis_2)
0.00717	9.00E-05	0.88571	0.78462	(lh.parstriangularis_3)(lh.rostralmiddlefrontal_1)(lh.rostralmiddlefrontal_12)(lh.rostralmiddlefrontal_6)
0.00717	9.00E-05	0.88571	0.78462	(lh.fusiform_1)(lh.fusiform_3)(lh.fusiform_5)(lh.lateraloccipital_9)
0.00717	9.00E-05	0.88571	0.78462	(rh.lateralorbitofrontal_6)(rh.lateralorbitofrontal_7)(rh.parstriangularis_4)(rh.rostralmiddlefrontal_12)
0.00725	9.00E-05	0.96571	0.8954	(rh.lateralorbitofrontal_6)(rh.parsorbitalis_2)
0.00729	0.0001	0.93143	0.84519	(rh.lateralorbitofrontal_2)(rh.lateralorbitofrontal_7)(rh.parstriangularis_1)(rh.rostralmiddlefrontal_12)
0.00739	0.0001	0.89143	0.79231	(Left-Hippocampus)(lh.precuneus_10)
0.00739	0.0001	0.89143	0.79231	(rh.precuneus_8)(rh.superiorparietal_7)
0.00739	0.0001	0.89143	0.79231	(lh.caudalmiddlefrontal_3)(lh.caudalmiddlefrontal_4)(lh.superiorfrontal_12)
0.00739	0.0001	0.89143	0.79231	(Left-Hippocampus)(lh.isthmuscingulate_3)(lh.precuneus_10)
0.00739	0.0001	0.89143	0.79231	(Left-Putamen)(lh.superiorfrontal_11)(lh.superiorfrontal_12)(lh.superiorfrontal_14)
0.00745	0.0001	0.82857	0.71538	(rh.fusiform_3)(rh.lateraloccipital_10)(rh.lingual_5)
0.00745	0.0001	0.82857	0.71538	(Left-Hippocampus)(lh.fusiform_5)(lh.inferiortemporal_8)
0.00751	0.0001	0.93714	0.85356	(lh.inferiorparietal_4)(lh.lateraloccipital_7)
0.00751	0.0001	0.93714	0.85356	(lh.medialorbitofrontal_2)(lh.rostralmiddlefrontal_11)
0.00751	0.00011	0.93714	0.85356	(lh.medialorbitofrontal_1)(lh.medialorbitofrontal_2)(lh.rostralmiddlefrontal_12)
0.00755	0.00011	0.96	0.88703	(rh.postcentral_10)(rh.postcentral_8)
0.00755	0.00011	0.96	0.88703	(Left-Pallidum)(Left-Putamen)(lh.precentral_4)
0.0076	0.00011	0.88	0.77824	(lh.lateralorbitofrontal_4)(lh.lateralorbitofrontal_5)(lh.lateralorbitofrontal_6)
0.00764	0.00011	0.90286	0.80753	(Right-Caudate)(Right-Putamen)(rh.rostralmiddlefrontal_12)(rh.rostralmiddlefrontal_8)
0.00764	0.00011	0.90286	0.80753	(rh.lateralorbitofrontal_2)(rh.lateralorbitofrontal_4)(rh.lateralorbitofrontal_6)(rh.lateralorbitofrontal_7)
0.00767	0.00011	0.94286	0.86192	(rh.medialorbitofrontal_1)(rh.rostralmiddlefrontal_11)
0.00767	0.00011	0.94286	0.86192	(rh.lateralorbitofrontal_4)(rh.lateralorbitofrontal_6)(rh.lateralorbitofrontal_7)
0.00771	0.00011	0.98286	0.92469	(rh.lateralorbitofrontal_4)(rh.lateralorbitofrontal_5)
0.00771	0.00012	0.95429	0.87866	(lh.medialorbitofrontal_1)(lh.rostralmiddlefrontal_12)
0.00771	0.00012	0.95429	0.87866	(rh.rostralmiddlefrontal_11)(rh.rostralmiddlefrontal_8)(rh.superiorfrontal_2)
0.00771	0.00012	0.95429	0.87866	(lh.insula_1)(lh.precentral_11)(lh.precentral_7)
0.00774	0.00012	0.94857	0.87029	(rh.inferiortemporal_7)(rh.middletemporal_1)(rh.middletemporal_2)
0.00774	0.00012	0.94857	0.87029	(rh.isthmuscingulate_2)(rh.precuneus_3)(rh.precuneus_4)(rh.precuneus_7)
0.00774	0.00012	0.94857	0.87029	(Left-Caudate)(Left-Putamen)(lh.caudalanteriorcingulate_2)(lh.caudalmiddlefrontal_3)(lh.superiorfrontal_11)
0.00778	0.00013	0.86286	0.75732	(rh.lateralorbitofrontal_6)(rh.lateralorbitofrontal_7)(rh.parstriangularis_1)(rh.parstriangularis_4)(rh.rostralmiddlefrontal_12)

0.0079	0.00013	0.83429	0.72308	(rh.precuneus_10)(rh.superiorparietal_1)(rh.superiorparietal_2)(rh.superiorparietal_7)
0.00792	0.00013	0.81714	0.70293	(rh.lateralorbitofrontal_6)(rh.lateralorbitofrontal_7)(rh.parstriangularis_1)(rh.rostralmiddlefrontal_12)(rh.rostralmiddlefrontal_13)
0.00792	0.00013	0.81714	0.70293	(Right-Putamen)(rh.insula_7)(rh.lateralorbitofrontal_2)(rh.lateralorbitofrontal_6)(rh.parstriangularis_1)
0.00815	0.00014	0.90857	0.8159	(Left-Caudate)(Left-Putamen)(lh.posteriorcingulate_3)(lh.superiorfrontal_14)
0.00815	0.00014	0.90857	0.8159	(lh.rostralmiddlefrontal_1)(lh.rostralmiddlefrontal_4)(lh.rostralmiddlefrontal_7)
0.00815	0.00014	0.90857	0.8159	(lh.rostralmiddlefrontal_8)(lh.superiorfrontal_3)(lh.superiorfrontal_5)
0.00815	0.00014	0.90857	0.8159	(lh.rostralmiddlefrontal_8)(lh.superiorfrontal_3)(lh.superiorfrontal_4)(lh.superiorfrontal_5)
0.00815	0.00014	0.90857	0.8159	(Left-Caudate)(Left-Putamen)(lh.caudalanteriorcingulate_2)(lh.superiorfrontal_12)(lh.superiorfrontal_14)
0.00823	0.00015	0.88571	0.78661	(Right-Putamen)(rh.lateralorbitofrontal_4)(rh.medialorbitofrontal_2)
0.00823	0.00015	0.88571	0.78661	(lh.insula_6)(lh.lateralorbitofrontal_6)(lh.parstriangularis_3)(lh.rostralmiddlefrontal_1)
0.0083	0.00015	0.83429	0.72385	(lh.parstriangularis_3)(lh.rostralmiddlefrontal_1)(lh.rostralmiddlefrontal_12)(lh.rostralmiddlefrontal_4)(lh.rostralmiddlefrontal_9)
0.00836	0.00015	0.84	0.73077	(lh.bankssts_2)(lh.middletemporal_2)
0.00836	0.00016	0.84	0.73077	(rh.inferiorparietal_12)(rh.lateraloccipital_8)(rh.precuneus_2)
0.00836	0.00016	0.84	0.73077	(lh.fusiform_2)(lh.lateraloccipital_6)(lh.lingual_6)
0.0085	0.00016	0.86857	0.76569	(rh.precentral_10)(rh.precentral_11)(rh.precentral_13)
0.0085	0.00016	0.86857	0.76569	(lh.rostralmiddlefrontal_12)(lh.rostralmiddlefrontal_8)(lh.superiorfrontal_3)
0.0085	0.00017	0.86857	0.76569	(Left-Caudate)(Left-Putamen)(lh.caudalanteriorcingulate_2)(lh.caudalmiddlefrontal_3)(lh.superiorfrontal_11)(lh.superiorfrontal_12)
0.0085	0.00017	0.85143	0.74477	(lh.caudalmiddlefrontal_2)(lh.caudalmiddlefrontal_3)(lh.rostralmiddlefrontal_3)
0.0085	0.00017	0.85143	0.74477	(Left-Putamen)(lh.lateralorbitofrontal_2)(lh.lateralorbitofrontal_4)(lh.lateralorbitofrontal_7)
0.00864	0.00018	0.91429	0.82427	(lh.inferiorparietal_4)(lh.inferiorparietal_5)
0.00864	0.00019	0.91429	0.82427	(rh.lateralorbitofrontal_6)(rh.parsorbitalis_2)(rh.rostralmiddlefrontal_13)
0.00864	0.00019	0.91429	0.82427	(Left-Caudate)(lh.caudalanteriorcingulate_2)(lh.caudalmiddlefrontal_3)(lh.superiorfrontal_11)(lh.superiorfrontal_12)
0.00883	0.00019	0.84571	0.73846	(lh.superiortemporal_11)(lh.temporalpole_1)
0.00883	0.0002	0.84571	0.73846	(lh.lateralorbitofrontal_5)(lh.lateralorbitofrontal_6)(lh.parstriangularis_1)
0.00883	0.0002	0.84571	0.73846	(Left-Pallidum)(lh.precentral_4)(lh.precentral_7)
0.00888	0.0002	0.89143	0.79498	(lh.lateralorbitofrontal_7)(lh.medialorbitofrontal_2)(lh.rostralmiddlefrontal_11)
0.00889	0.00021	0.97714	0.91632	(lh.lateralorbitofrontal_2)(lh.lateralorbitofrontal_7)
0.00889	0.00021	0.97714	0.91632	(Left-Caudate)(lh.lateralorbitofrontal_7)(lh.medialorbitofrontal_2)
0.00912	0.00024	0.92	0.83264	(lh.rostralmiddlefrontal_8)(lh.superiorfrontal_5)
0.00912	0.00025	0.92	0.83264	(lh.insula_2)(lh.insula_3)
0.00912	0.00026	0.92	0.83264	(rh.lateraloccipital_1)(rh.pericalcarine_3)
0.00912	0.00026	0.92	0.83264	(Left-Putamen)(lh.insula_2)(lh.insula_3)
0.00912	0.00027	0.92	0.83264	(lh.caudalanteriorcingulate_2)(lh.caudalmiddlefrontal_3)(lh.superiorfrontal_11)(lh.superiorfrontal_12)
0.00912	0.00028	0.92	0.83264	(Left-Putamen)(lh.insula_6)(lh.parsopercularis_1)(lh.parstriangularis_3)(lh.rostralmiddlefrontal_1)
0.00912	0.00029	0.92	0.83264	(rh.parstriangularis_3)(rh.rostralmiddlefrontal_12)
0.00926	0.00029	0.87429	0.77406	(lh.fusiform_2)(lh.lateraloccipital_6)(lh.pericalcarine_2)
0.00926	0.0003	0.87429	0.77406	(Left-Caudate)(Left-Putamen)(lh.caudalmiddlefrontal_3)(lh.rostralmiddlefrontal_4)(lh.superiorfrontal_11)
0.0093	0.00031	0.85143	0.74615	(Right-Putamen)(rh.caudalanteriorcingulate_1)(rh.caudalanteriorcingulate_2)
0.0093	0.00032	0.85143	0.74615	(lh.rostralanteriorcingulate_2)(lh.superiorfrontal_4)(lh.superiorfrontal_5)
0.0093	0.00033	0.85143	0.74615	(Right-Caudate)(Right-Putamen)(rh.caudalanteriorcingulate_1)(rh.caudalanteriorcingulate_2)
0.0093	0.00034	0.85143	0.74615	(Right-Caudate)(rh.lateralorbitofrontal_7)(rh.rostralmiddlefrontal_12)(rh.rostralmiddlefrontal_8)
0.00932	0.00036	0.98857	0.93724	(lh.precuneus_6)(lh.superiorparietal_11)
0.00932	0.00037	0.98857	0.93724	(rh.lateralorbitofrontal_2)(rh.lateralorbitofrontal_6)(rh.parstriangularis_1)
0.00932	0.00038	0.98857	0.93724	(lh.lingual_1)(lh.pericalcarine_2)(lh.pericalcarine_3)
0.00956	0.00042	0.89714	0.80335	(rh.lateralorbitofrontal_6)(rh.lateralorbitofrontal_7)(rh.parstriangularis_4)
0.00956	0.00043	0.89714	0.80335	(rh.lateraloccipital_1)(rh.lateraloccipital_3)(rh.pericalcarine_3)
0.00956	0.00045	0.89714	0.80335	(Right-Putamen)(rh.rostralmiddlefrontal_11)(rh.rostralmiddlefrontal_12)
0.00956	0.00048	0.89714	0.80335	(rh.lateralorbitofrontal_7)(rh.medialorbitofrontal_2)(rh.rostralmiddlefrontal_11)(rh.rostralmiddlefrontal_12)
0.00958	0.0005	0.92571	0.841	(lh.lateraloccipital_7)(lh.lingual_1)
0.00958	0.00053	0.92571	0.841	(rh.precuneus_5)(rh.superiorparietal_8)

0.00958	0.00056	0.92571	0.841	(rh.supramarginal_6)(rh.supramarginal_7)(rh.supramarginal_9)
0.00976	0.00059	0.82857	0.71967	(lh.fusiform_1)(lh.fusiform_3)(lh.lingual_8)
0.00976	0.00063	0.82857	0.71967	(Left-Putamen)(lh.lateralorbitofrontal_2)(lh.lateralorbitofrontal_6)(lh.lateralorbitofrontal_7)
0.00976	0.00067	0.97143	0.90795	(rh.lateralorbitofrontal_7)(rh.medialorbitofrontal_2)(rh.rostralmiddlefrontal_13)
0.00976	0.00071	0.97143	0.90795	(rh.rostralmiddlefrontal_11)(rh.superiorfrontal_1)(rh.superiorfrontal_2)
0.00976	0.00077	0.97143	0.90795	(lh.parstriangularis_3)(lh.rostralmiddlefrontal_1)(lh.rostralmiddlefrontal_6)
0.00978	0.00083	0.85714	0.75385	(lh.insula_2)(lh.postcentral_8)
0.00978	0.00091	0.85714	0.75385	(rh.cuneus_2)(rh.lateraloccipital_1)
0.00978	0.001	0.85714	0.75385	(rh.fusiform_1)(rh.lateraloccipital_8)(rh.pericalcarine_3)
0.00978	0.00111	0.85714	0.75385	(Left-Caudate)(Left-Putamen)(lh.rostralmiddlefrontal_3)
0.00978	0.00125	0.85714	0.75385	(lh.lateralorbitofrontal_6)(lh.parstriangularis_2)(lh.parstriangularis_3)(lh.rostralmiddlefrontal_1)(lh.rostralmiddlefrontal_9)
0.00999	0.005	0.93143	0.84937	(lh.lateralorbitofrontal_7)(lh.parstriangularis_3)(lh.rostralanteriorcingulate_1)(lh.rostralmiddlefrontal_12)
0.00999	0.01	0.93143	0.84937	(lh.rostralmiddlefrontal_9)(lh.superiorfrontal_2)(lh.superiorfrontal_3)

:al_9)
