# Supplementary material for: The frequent complete subgraphs in the human connectome
Source: PLoS One. 2020 Aug 20;15(8):e0236883. doi: 10.1371/journal.pone.0236883 (PMC7444532; doi:10.1371/journal.pone.0236883)
Supplement: S4 Table — In S4 Table the results of 20 runs are presented: the female plus and male plus rows show the number of frequent complete subgraphs with significantly higher frequencies (p = 0.01) in females and males, respectively. (PDF) [file pone.0236883.s004.pdf]

clique size	2	3	4	5	6	7	8	9	10

run #1

male:	2515	3806	2365	752	111	5	0	0	0
female:	2445	3513	2283	828	189	31	2	0	0

male plus: 472
female plus: 264

run #2

male:	2514	3868	2508	812	113	4	0	0	0
female:	2396	3418	2232	844	195	21	1	0	0

male plus: 422
female plus: 163

run #3

male:	2468	3657	2285	702	91	5	0	0	0
female:	2409	3511	2376	977	273	48	2	0	0

male plus: 603
female plus: 233

run #4

male:	2518	3723	2344	735	94	3	0	0	0
female:	2392	3338	2084	714	139	18	1	0	0

male plus: 686
female plus: 232

run #5

male:	2520	3915	2546	840	123	4	0	0	0
female:	2444	3427	2171	748	136	15	1	0	0

male plus: 697
female plus: 136

run #6

male:	2552	3809	2287	682	94	4	0	0	0
female:	2436	3517	2188	792	167	25	2	0	0

male plus: 466
female plus: 194

run #7

male:	2449	3561	2075	569	62	1	0	0	0
female:	2330	3152	1896	643	128	16	0	0	0

male plus: 345
female plus: 157

run #8

male:	2484	3659	2244	684	93	3	0	0	0
female:	2348	3209	1969	671	134	22	2	0	0

male plus: 554
female plus: 165

run #9

male:	2513	3885	2599	989	199	21	0	0	0
female:	2419	3484	2217	745	129	2	0	0	0

male plus: 706
female plus: 126

run #10

male:	2535	3820	2464	873	142	5	0	0	0
female:	2357	3345	2111	761	146	13	0	0	0

male plus: 743
female plus: 224

run #11

male:	2563	3863	2547	908	168	15	0	0	0
female:	2383	3409	2262	808	182	25	1	0	0

male plus: 695
female plus: 178

run #12

male:	2470	3703	2380	744	100	4	0	0	0
female:	2369	3353	2115	729	141	14	0	0	0

male plus: 552
female plus: 159

run #13

male:	2556	4030	2666	917	139	5	0	0	0
female:	2414	3505	2232	752	116	10	0	0	0

male plus: 750
female plus: 166

run #14

male:	2537	3796	2394	794	118	6	0	0	0
female:	2394	3402	2143	715	132	9	0	0	0

male plus: 644
female plus: 187

run #15

male:	2529	3880	2419	739	94	4	0	0	0
female:	2430	3426	2050	704	150	23	1	0	0

male plus: 568
female plus: 203

run #16

male:	2475	3605	2193	684	104	4	0	0	0
female:	2369	3325	2098	663	116	13	0	0	0

male plus: 483
female plus: 181

run #17

male:	2538	3891	2517	907	168	16	0	0	0

female:	2455	3516	2266	742	107	2	0	0	0

male plus: 884
female plus: 153

run #18

male:	2496	3774	2417	808	118	5	0	0	0
female:	2409	3404	2152	756	179	29	1	0	0

male plus: 781
female plus: 116

run #19

male:	2428	3531	2093	648	108	2	0	0	0
female:	2391	3342	2134	730	130	15	0	0	0

male plus: 408
female plus: 207

run #20

male:	2537	3985	2643	929	153	5	0	0	0
female:	2372	3313	2129	791	183	26	1	0	0

male plus: 694
female plus: 198
